# Characterisation of the Proteome of *Leptospira interrogans* Serovar Canicola as a Resource for the Identification of Common Serovar Immunogenic Proteins

**DOI:** 10.1155/2014/572901

**Published:** 2014-05-27

**Authors:** P. C. Humphryes, M. E. Weeks, N. G. Coldham

**Affiliations:** ^1^Animal Health and Veterinary Laboratories Agency, Addlestone, New Haw, Surrey KT15 3NB, UK; ^2^UCL Institute of Child Health, 30 Guilford Street, London WC1N 1EH, UK

## Abstract

Over 230 serovars of *Leptospira interrogans* have been identified; however few have been completely characterised. The aim of this study was to characterise the proteome of serovar Canicola and to compare this against the serovars of Copenhageni and Pomona. 2D-LC/MS analysis identified 1653 *Leptospira* proteins in serovar Canicola; 60 of these proteins were common to Copenhageni and Pomona, 16 of which are known to be immunogenic. This study provides the first reported proteome for serovar Canicola and suggests that proteomic comparison of different serovars could be used as a tool for identification of novel target molecules for vaccine development.

## 1. Introduction

*L. interrogans* is a spirochete responsible for leptospirosis and Weil's disease. Over 230 pathogenic serovars have been identified [1], each potentially fatal if left untreated. Leptospirosis continues to be a significant threat to food producing animals; in 2010 of the 8,681 suspected serum samples examined in the UK by the Animal Health and Veterinary Laboratories Agencies (AHVLA) 2,946 [2] were identified as being seropositive for* Leptospira*. Approximately 59% (1736) of these seropositive samples were derived from dogs and the majority of these (69%) were positive for serovar Canicola, which to date has not been fully characterised using genomics or proteomics. Whilst the mortality rate associated with leptospirosis remains low, due to its susceptibility to antibiotics [3] and the routine vaccination of domestic and farm animals, initial clinical signs such as cessation of milk production and miscarriage [4] can be commercially damaging to the dairy farming industry. Vaccination represents an effective treatment strategy for prevention of the disease; however the vaccines currently available are all serovar specific.

Serological methods for identification of* Leptospira* serovars, such as the microscopic agglutination test (MAT), are well established. Whilst being effective these are extremely time consuming and require access to a large* Leptospira* strain/antiserum collection [5], to which many third world countries do not necessarily have access; this in conjunction with the limited public profile of leptospirosis often leads to misdiagnosis and general under reporting of infection. In addition false positive results using the MAT have been reported due to the prior vaccination of test subjects [6]. Genetic classification systems for* L. interrogans* are not routinely utilised as genetically different species are often found to be serologically identical [5] which leads to poor reproducibility with the MAT [5, 7]. Protein based diagnostic ELISAs for the detection of* Leptospira* have been reported previously [8–10]; however the development of a routine protein based test for the taxonomic classification of the various serovars has yet to be developed. It is conceivable that characterisation and cross comparison of the protein content of the different serovars might unveil serovar specific protein markers which could be developed into a routine diagnostic test (such as an ELISA) to replace the MAT and track the epidemiology of the bacteria more accurately. However to date only serovars Copenhageni [11–15], Pomona [16], and Lai [17, 18] have had their proteomes characterised. In addition improved epidemiology of the bacteria, through the development of a routine protein based ELISA, would enable prophylactic treatment strategies, such as vaccination [19], to be more appropriately implemented for control purposes. Improved characterisation of the serovars could also aid in the identification of novel multiserovar drug and vaccine targets.

The aim of the present study was to characterise the proteome of* L. interrogans* serovar Canicola and to compare this against the published proteomes of serovars Copenhageni and Pomona to identify proteins common to each and determine functionally important differences. In addition characterisation of the serovar Canicola proteome would provide a valuable resource for future research into the treatment and prevention of leptospirosis.

## 2. Results and Discussion

**Figure 1:**
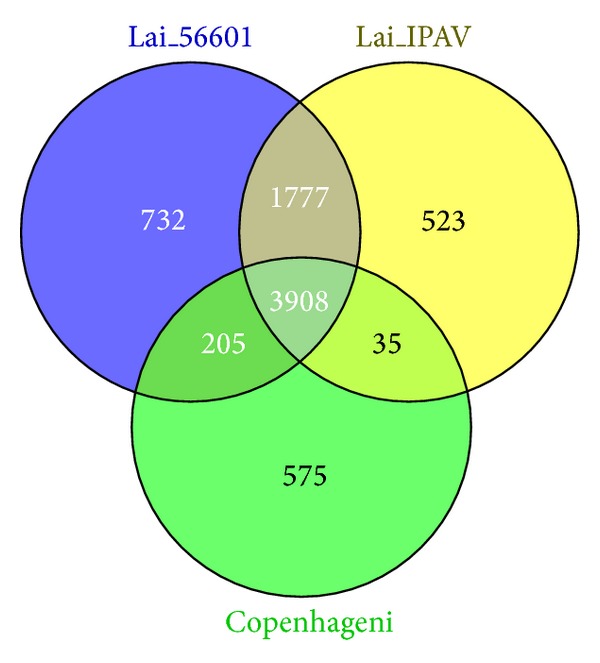
Venn analysis of total number of peptides identified in serovar Canicola using three different protein databases of serovars Copenhageni (strain Fiocruz L1-130), Lai (strain IPAV) and Lai (strain 56601).

Analysis of serovar Canicola using 2D-LC/MS identified 2961 unique* Leptospira* accession numbers across the three protein databases used; 1653 of these had unique protein identifications which represents the detected proteome (Table 1, Appendix 1 in the Supplementary Material available online at http://dx.doi.org/10.1155/2014/572901) for the serovar (peptide identifications are given in Appendix 2 in the Supplementary Material). This represents a substantial improvement in coverage over the serovar Copenhageni and Pomona proteomes previously determined by Eshghi et al. [11] and Vieira et al. [16], respectively (Table 1). Whilst the studies performed by Malmström et al. [12], Cao et al. [17], and Zhong at al. [18] all identified a larger number of proteins (Table 1) it is important to note that all of these studies benefited from a serovar specific protein database; in addition the studies by Malmström et al. [12] and Cao et al. [17] did not report the use of biological replicates. In the absence of a Canicola specific genome sequence for data interrogation the genome for serovar Copenhageni was used in the first instance as a proxy, as previously reported [16], resulting in the identification of 1015* Leptospira* proteins. Further data interrogation using protein databases derived from two serovar Lai genome sequences enabled an additional 638 proteins to be identified. A comparison of the peptides identified in serovar Canicola using the three different databases (Figure 1) demonstrated considerable variation between genomes, particularly for serovar Lai which only had 1777 peptides conserved between the two genomes (Figure 1). This clearly demonstrates the inter- and intraheterogeneity of different serovars of* L. interrogans* at the peptide level and the need for future* Leptospira* proteomic studies to search their data against multiple genomes where serovar/strain specific databases are unavailable.

**Figure 2:**
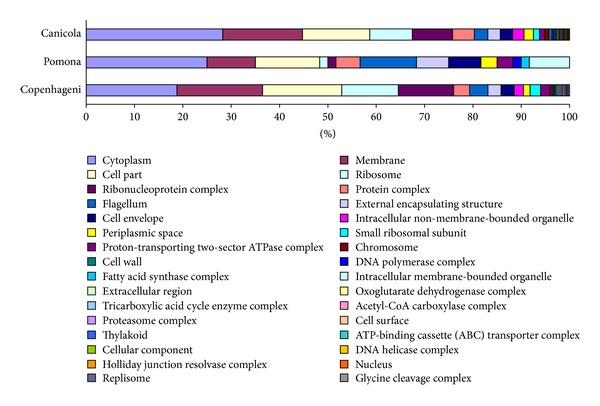
Proteins identified in different serovars of* L. interrogans* grouped by their cellular content. Note legend reads left to right.

**Figure 3:**
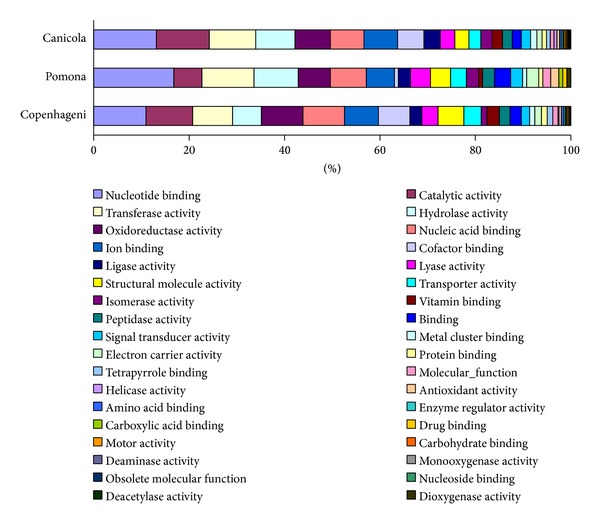
Proteins identified in different serovars of* L. interrogans* grouped by functional annotation. Note legend reads left to right.

Proteins identified in serovars Copenhageni [11] and Pomona [16] were selected for further comparison against serovar Canicola as their proteomes are freely accessible online. The cellular contents of the proteins identified in the different studies were determined and compared (Figure 2) and analysis of the different functional groups of proteins identified in the three studies (Figure 3) was also performed to establish if there were any biological differences between serovars. Additional investigation, taking into account differences in the extraction and/or processing methodologies used in the different studies, is required to validate these observations; however this does demonstrate the value of cross serovar analysis as an investigative tool.

A conserved proteome for the three serovars was subsequently determined (Appendix 3 in the Supplementary Material); 60 proteins were revealed to be conserved (Appendices 3–5 in the Supplementary Material), sixteen of which have been previously reported in the literature as immunogenic (Table 2) through immune studies in the hamster [20] and mice models [23] and immunoblotting with serum from infected humans [22] and mice [21]. Further work is required to determine if any of the identified conserved proteins could be used as viable targets for therapeutic drugs, antimicrobials, and/or vaccines; however this does suggest that proteomic comparison of serovars could also be used as an effective screening tool; further refinement of the conserved proteome presented herein is suggested as the proteomes of additional serovars are published.

In conclusion this study provides the first reported proteome for* L. interrogans* serovar Canicola, which is of particular importance due to its high frequency of infection in dogs. The identified protein/peptide lists available in the appendices in the Supplementary Material also provide a valuable resource for future research into both serovar Canicola and* Leptospira* in general.

## 3. Materials and Methods

Starter cultures of* L. interrogans* serovar Canicola (strain Hond Utrecht IV; from AHVLA, UK) were prepared by inoculation of 20 mL EMJH (Becton Dickinson, USA) media with 1 mL pure culture (passage number 1) and incubated for 7 days at 30°C with orbital agitation at 50 rpm. Larger working cultures (*n* = 3) for proteome extraction were initiated by inoculation of 400 mL EMJH media with 10 mL of starter culture and incubated at 30°C (50 rpm). Bacteria were harvested during the logarithmic growth phase (~5∗10^8^ cells/mL) by cooling the cultures on ice for 30 minutes and collection of cells by centrifugation at 4000 ×g for 20 minutes at 4°C. The number of bacterial cells was assessed by dark field microscopy using a Thoma counting chamber (0.1 mm depth, 1/400 m^2^). The bacterial cells were washed by suspension in 100 mL chilled phosphate buffered saline (PBS; 200 mM, pH 7.2) and pelleted (4000 ×g; 20 min, 4°C). Bacterial cell pellets were suspended in PBS (10 mL) containing PMSF (100 *μ*M) and lysed by 6-second pulses of probe sonication (amplitude 60) using a Vibra-Cell ultrasonic processor (Sonics and Materials, USA) for 3 minutes on ice. Cell debris was removed by centrifugation at 3000 ×g and the supernatant retained. A low speed cytosolic extract was produced from the supernatant by centrifugation at 32000 ×g for 30 minutes. The pellet was retained and the supernatant (cytosol extract) was then desalted by dilution with ammonium bicarbonate (2.5 mM; pH 8.0), concentrated to 0.5 mL by centrifugation in 5 kDa molecular weight cut-off concentrators (Sartorius Stedim, France), and stored at −20°C.

The retained pellet was then washed by suspension in chilled phosphate buffered saline (PBS; 200 mM, pH 7.2) and collected by centrifugation (32000 ×g). The washed pellet was redissolved in 3 mL lysis buffer (Urea 5 M, Thiourea 2 M, DTT 100 mM, CHAPS 2%, 3-(decyldimethylammonio)propanesulfonate inner salt 2%, Tris base 0.48%) and centrifuged at 32000 ×g for 30 minutes. The protein extract was precipitated in a 4-fold excess of ice cold acetone and incubated at −20°C for 48 hours prior to centrifugation (3000 ×g for 30 minutes). The resulting pellet (precipitated extract) was desalted and concentrated as before using ammonium bicarbonate and vivaspin centrifugal filters, respectively. Estimation of protein concentration for both extracts was then determined using the Bradford method (Sigma-Aldrich, UK), with bovine serum albumin as the calibration standard (0.05–1.0 mg/mL). Three replicates of each bacterial extract, normalised by dilution in 2.5 mM ammonium bicarbonate (pH 8.0) to 100 *μ*g of protein, were heat denatured at 95°C for 5 minutes and then digested overnight with 2 *μ*g sequencing grade trypsin (Promega) [24]; digestion was terminated by the addition of 1 *μ*L of 25.2 M formic acid (Fluka). Tryptic peptides (50 *μ*g) were fractionated on a Biobasic SCX HPLC (2.1 × 100 mm) column (Thermo Scientific, UK) using a Hewlett-Packard 1100 HPLC system, as previously described [25], at a flow rate of 0.25 mL/min. Mobile phases used were 75 : 25 2.5 mM ammonium acetate : acetonitrile pH 4.5 (A) and 75 : 25 250 mM ammonium acetate : acetonitrile pH 4.5 (B) with a binary gradient (*t* = 0 min, A 100%; *t* = 5 min, A 100%; *t* = 18 min, 65% A; *t* = 20 min, B 100%; *t* = 22 min, A 100%; *t* = 32 min, A 100%). Eluted peptides were monitored at 280 nm and 15 fractions (1 mL) were collected between 8 and 23 min. The SCX fractions were taken to dryness at 60°C under vacuum using an Eppendorf 5301 centrifugal concentrator (Eppendorf, UK).

Dried SCX fractions were resuspended in 0.1% v/v formic acid (20 *μ*L) and analyzed on an Agilent 6520 quadrupole time-of-flight (Q-TOF) mass spectrometer (Agilent Technologies, UK) with an HPLC chip cube source. The chip consisted of a 40 nL enrichment column (Zorbax 300 SB- C18; 5 *μ*m) and a 75 *μ*m × 150 mm analytical column (Zorbax 300 SB- C18; 5 *μ*m) driven by the Agilent Technologies 1200 series nano/capillary HPLC system. Both systems were controlled by Masshunter Workstation Data Acquisition for Q-TOF (Version B.02.00, Patches 1, 2; Agilent Technologies). Tryptic peptides (1 *μ*L injection volume) were loaded onto the enrichment column of the chip and washed with eight column volumes of 0.1% v/v trifluoroacetic acid (TFA). Tryptic peptides were separated on the analytical column and eluted directly into the mass spectrometer. Mobile phases used were 0.1% v/v TFA (A) and 90 : 10 acetonitrile: 0.1% v/v TFA (B) with a binary gradient (*t* = 0 min, A 95%; *t* = 5 min, A 95%; *t* = 40 min, A 60%; *t* = 41 min, A 20%; *t* = 45 min, A 20%; *t* = 47 min, A 95%) at a flow rate of 0.6 *μ*L/min. The mass spectrometer was run in positive ion mode, and MS survey scans were run over a range of *m*/*z* 250 to 3000 and at five spectra per second. Precursor ions were selected for auto MS/MS at an absolute threshold of 2000 and a relative threshold of 0.01, with a maximum of 5 precursors per cycle, and active exclusion set at 1 spectra and released after 3 minutes. Precursor charge state selection and preference were set to 2+ and then 3+.

The search engine Spectrum Mill (Agilent, UK) was used to extract MS/MS data from Masshunter acquisition files and proteins were subsequently identified by comparison of tryptic peptide product ion mass spectra against those generated* in silico* from a protein database. Search parameters included selection of trypsin as the proteolytic enzyme with up to two missed cleavage sites and a variable modification for oxidation of methionine residues; precursor and product mass tolerances were set to 20 and 50 ppm, respectively. Identified protein lists (and associated information) with a Spectrum Mill protein score higher than 11 were exported as tab separated files for bioinformatics analysis.

To date, only three genome sequences for* Leptospira interrogans*, corresponding to serovars Copenhageni (strain Fiocruz L1-130), Lai (strain IPAV), and Lai (strain 56601), have been determined and published in peer-reviewed journals. To identify* L. interrogans* proteins present in serovar Canicola, three custom protein databases derived from these published genomes were used for database interrogation; proteins identified had at least two distinct tryptic peptides and were present in all three technical replicates. Proteins identified in the precipitated and cytosol extracts using the three databases were reassembled into a single proteome (Appendix 1 in the Supplementary Material) using Access (Microsoft, USA) and duplicate identifications were removed based on protein name; duplicate peptide identifications were removed based on amino acid sequence (Appendix 2 in the Supplementary Material) and a Venn diagram (Figure 1) was generated using Venny [26] to compare peptide identifications between the three databases. Functional annotation of proteins present in serovars Canicola, Copenhageni, and Pomona was determined using the Protein Information Resource (PIR; http://pir.georgetown.edu/ accessed on 12/12/12) and proteins conserved between the three serovars were determined using Access (Appendix 3 in the Supplementary Material).

## Supplementary Material

Supplementary materials contain five appendices with additional protein and peptide data/comparisons generated during this study.

## Figures and Tables

**Table 1:** Comparison of the total number of *Leptospira* proteins identified in this project with previous studies.

Reference	Species	Strain	*Leptospira* proteins identified
[11]	*L. interrogans* serovar Copenhageni	Fiocruz L1-130	563
[16]	*L. interrogans* serovar Pomona	LPF	108
[12]	*L. interrogans* serovar Copenhageni	Fiocruz L1-130	2221
[17]	*L. interrogans* serovar Lai	56601	2540
[18]	*L. interrogans* serovar Lai	IPAV	2608
[18]	*L. interrogans* serovar Lai	56601	2673
This study	*L. interrogans* serovar Canicola	Hond Utrecht IV	1653

**Table 2:** Immunogenic proteins found to be conserved between three serovars of *Leptospira interrogans*.

Reference	Accession number	Protein identification	Function
[20]	Q72N71	LipL41	Binding
[21]	Q72PA2	Succinyl-CoA synthetase beta subunit	Catalytic activity; nucleotide binding; ligase activity; ion binding
[21]	Q72PR0	Putative glutamine synthetase protein	Catalytic activity; ligase activity
[22]	Q72Q79	Fructose-bisphosphate aldolase	Lyase activity
[21]	Q72R58	Flagellin protein	Structural molecule activity
[21]	Q72RU5	LipL45	Unknown
[21]	Q72S54	Flagellin protein	Structural molecule activity
[21]	Q72S55	Flagellin protein	Structural molecule activity
[21]	Q72SG6	ATP-dependent Clp protease, proteolytic subunit	Peptidase activity; nucleotide binding; hydrolase activity
[23]	Q72SM7	LipL32	Unknown
[21]	Q72T03	Peroxiredoxin	Antioxidant activity; oxidoreductase activity
[22]	Q72T27	Putative citrate lyase	Catalytic activity; lyase activity; ion binding
[21]	Q72U13	Elongation factor Ts	Nucleic acid binding
[21]	Q72V20	Hypothetical protein LIC10483	Unknown
[21]	Q72VD7	Electron transport flavoprotein beta subunit	Electron carrier activity
[21]	Q72WD5	DNA polymerase III beta subunit	Nucleic acid binding; transferase activity; hydrolase activity

Total conserved proteins			16
