# Supplementary material for: Characterisation of the Proteome of Leptospira interrogans Serovar Canicola as a Resource for the Identification of Common Serovar Immunogenic Proteins
Source: Int J Proteomics. 2014 May 27;2014:572901. doi: 10.1155/2014/572901 (PMC4058600; doi:10.1155/2014/572901)
Supplement: Supplementary file 1 — Supplementary materials contain five appendices with additional protein and peptide data/comparisons generated during this study. [file 572901.f1.pdf]

**Appendix 1**: *Leptospira* proteins identified in *Leptospira in*

| Accession Number | Protein identification | Protein Length (Amino Acids) |
|---|---|---|
| 24212702 | DNA polymerase III subunit beta | 373 |
| 24212705 | DNA gyrase subunit B | 639 |
| 24212706 | DNA gyrase subunit A | 834 |
| 24212709 | hypothetical protein LA_0009 | 245 |
| 24212720 | short chain dehydrogenase | 248 |
| 24212721 | translation factor Sua5 | 203 |
| 24212731 | hypothetical protein LA_0031 | 234 |
| 24212733 | inositol monophosphatase family protein | 283 |
| 24212740 | ribonuclease R | 748 |
| 24212743 | TPR-repeat-containing protein | 502 |
| 24212745 | 3-dehydroquinate dehydratase | 234 |
| 24212751 | nucleoside-diphosphate-sugar epimerase | 312 |
| 24212752 | NAD(P)(+) transhydrogenase subunit alpha | 389 |
| 24212754 | histidyl-tRNA synthetase | 439 |
| 24212756 | OmpA family protein | 687 |
| 24212772 | deoxycytidine triphosphate deaminase | 174 |
| 24212783 | diaminopimelate epimerase | 281 |
| 24212807 | hypothetical protein LA_0107 | 118 |
| 24212813 | cystathionine gamma-synthase | 500 |
| 24212824 | imidazoleglycerol-phosphate dehydratase/histidinol-phosphatase | 206 |
| 24212826 | phosphoribosylformimino-5-aminoimidazole carboxamide ribotide isomerase | 241 |
| 24212827 | hypothetical protein LA_0127 | 404 |

| | | |
|---|---|---|
| 24212830 | phosphomethylpyrimidine kinase protein | 274 |
| 24212834 | ribosome-associated GTPase | 318 |
| 24212835 | hypothetical protein LA_0135 | 433 |
| 24212841 | metal-dependent hydrolase of the beta-lactamase superfamily I | 312 |
| 24212846 | transcriptional regulator | 699 |
| 24212849 | SET domain-containing protein | 133 |
| 24212853 | hypothetical protein LA_0153 | 489 |
| 24212857 | chorismate synthase | 380 |
| 24212860 | NADH dehydrogenase (ubiquinone) chain G | 482 |
| 24212878 | preprotein translocase subunit YidC | 627 |
| 24212879 | Jag-like RNA-binding protein | 236 |
| 24212884 | hypothetical protein LA_0184 | 692 |
| 24212885 | fumarate hydratase | 464 |
| 24212918 | hypothetical protein LA_0218 | 510 |
| 24212922 | OmpA family lipoprotein | 195 |
| 24212943 | cytochrome c oxidase polypeptide I | 534 |
| 24212950 | integral membrane protein with PIN domain | 343 |
| 24212951 | phosphoenolpyruvate carboxykinase | 530 |
| 24212956 | aspartyl/glutamyl-tRNA amidotransferase subunit B | 486 |
| 24212963 | hypothetical protein LA_0263 | 242 |
| 24212972 | hypothetical protein LA_0272 | 337 |
| 24212978 | hypothetical protein LA_0278 | 94 |
| 24212980 | cAMP-binding protein | 405 |

| | | |
|---|---|---|
| 24212984 | hypothetical protein LA_0284 | 231 |
| 24213000 | ADP-ribose pyrophosphatase | 182 |
| 24213011 | metal-dependent hydrolase | 416 |
| 24213013 | elongation factor G | 706 |
| 24213035 | NAD(P)(+) transhydrogenase subunit beta | 466 |
| 24213060 | glycine dehydrogenase | 964 |
| 24213065 | hypothetical protein LA_0365 | 553 |
| 24213069 | ribose-5-phosphate isomerase | 145 |
| 24213091 | ATP-dependent protease ClpA | 846 |
| 24213101 | phosphohistidine phosphatase | 160 |
| 24213102 | ABC transporter ATP-binding protein | 304 |
| 24213108 | tryptophanyl-tRNA synthetase | 322 |
| 24213111 | electron transfer flavoprotein subunit alpha | 319 |
| 24213112 | electron transfer flavoprotein subunit beta | 254 |
| 24213114 | isovaleryl-CoA dehydrogenase | 394 |
| 24213119 | hypothetical protein LA_0419 | 182 |
| 24213138 | acyl-CoA dehydrogenase | 565 |
| 24213147 | hypothetical protein LA_0447 | 56 |
| 24213157 | acetyl-CoA acetyltransferase | 393 |
| 24213163 | riboflavin synthase subunit beta | 151 |
| 24213169 | isopropylmalate/homocitrate/citramalate synthase | 428 |
| 24213171 | hypothetical protein LA_0471 | 157 |
| 24213194 | hypothetical protein LA_0494 | 231 |

| | | |
|---|---|---|
| 24213205 | hypothetical protein LA_0505 | 366 |
| 24213213 | UDP-3-O-[3-hydroxymyristoyl] glucosamine N-acyltransferase | 340 |
| 24213233 | pyridoxal phosphate-dependent aminotransferase | 369 |
| 24213244 | hypothetical protein LA_0544 | 391 |
| 24213246 | hypothetical protein LA_0546 | 841 |
| 24213252 | periplasmic solute-binding protein | 338 |
| 24213295 | glutathione S-transferase-like protein | 209 |
| 24213311 | cell division protein FtsA | 409 |
| 24213338 | shikimate 5-dehydrogenase | 313 |
| 24213346 | 30S ribosomal protein S1 | 384 |
| 24213348 | anti-sigma regulatory factor | 222 |
| 24213349 | excinuclease ABC subunit B | 666 |
| 24213356 | mannose-6-phosphate isomerase | 330 |
| 24213365 | metallo-beta-lactamase superfamily protein | 321 |
| 24213371 | citrate synthase | 426 |
| 24213374 | oxidoreductase | 514 |
| 24213393 | aspartate kinase | 405 |
| 24213420 | hypothetical protein LA_0720 | 249 |
| 24213427 | carbamoyl phosphate synthase large subunit | 1106 |
| 24213434 | peroxiredoxin-like protein | 183 |
| 24213437 | elongation factor Tu | 401 |
| 24213438 | 30S ribosomal protein S10 | 102 |
| 24213439 | 50S ribosomal protein L3 | 206 |
| 24213440 | 50S ribosomal protein L4 | 211 |

| | | |
|---|---|---|
| 24213441 | 50S ribosomal protein L23 | 104 |
| 24213442 | 50S ribosomal protein L2 | 279 |
| 24213444 | 50S ribosomal protein L22 | 110 |
| 24213445 | 30S ribosomal protein S3 | 225 |
| 24213446 | 50S ribosomal protein L16 | 137 |
| 24213451 | 50S ribosomal protein L5 | 182 |
| 24213452 | 30S ribosomal protein S14 | 61 |
| 24213453 | 30S ribosomal protein S8 | 133 |
| 24213454 | 50S ribosomal protein L6 | 179 |
| 24213455 | 50S ribosomal protein L18 | 122 |
| 24213458 | 50S ribosomal protein L15 | 180 |
| 24213460 | adenylate kinase | 187 |
| 24213462 | 30S ribosomal protein S13 | 125 |
| 24213464 | 30S ribosomal protein S4 | 207 |
| 24213465 | RNA polymerase subunit alpha | 325 |
| 24213466 | 50S ribosomal protein L17 | 171 |
| 24213476 | L,L-diaminopimelate aminotransferase | 408 |
| 24213478 | aspartate carbamoyltransferase catalytic subunit | 320 |
| 24213485 | Zn-dependent hydrolase | 347 |
| 24213488 | SAM-dependent methyltransferase | 315 |
| 24213499 | cell shape determination protein | 146 |
| 24213500 | pyridoxal phosphate biosynthesis protein | 352 |
| 24213509 | trypsin-like serine protease | 388 |
| 24213510 | Holliday junction DNA helicase B | 341 |
| 24213527 | thioesterase | 286 |

| | | |
|---|---|---|
| 24213533 | pantothenate kinase | 257 |
| 24213538 | UDP-N-acetylmuramoylalanine--D-glutamate ligase | 463 |
| 24213539 | anti-sigma factor antagonist | 110 |
| 24213540 | indole-3-glycerol-phosphate synthase | 252 |
| 24213545 | 2-isopropylmalate synthase | 298 |
| 24213549 | 50S ribosomal protein L21 | 104 |
| 24213551 | 50S ribosomal protein L27 | 84 |
| 24213552 | GTPase ObgE | 357 |
| 24213553 | gamma-glutamyl kinase | 290 |
| 24213554 | gamma-glutamyl phosphate reductase | 416 |
| 24213555 | nicotinate-nucleotide adenylyltransferase | 199 |
| 24213562 | thiol peroxidase | 171 |
| 24213573 | cytoplasmic membrane protein | 423 |
| 24213579 | hypothetical protein LA_0879 | 156 |
| 24213590 | NADH dehydrogenase (ubiquinone) chain F | 443 |
| 24213592 | NADH dehydrogenase (ubiquinone) chain D | 405 |
| 24213593 | NADH dehydrogenase (ubiquinone) chain C | 178 |
| 24213599 | MaoC-like acyl dehydratase | 154 |
| 24213600 | MarR family transcriptional regulator | 151 |
| 24213603 | methylase | 543 |
| 24213609 | methylglyoxal synthase | 149 |
| 24213613 | NHL repeat protein | 676 |
| 24213618 | enoyl-CoA hydratase | 266 |
| 24213629 | enolase-phosphatase | 234 |
| 24213640 | hypothetical protein LA_0940 | 392 |
| 24213642 | transcription elongation factor NusA | 460 |
| 24213647 | polynucleotide phosphorylase | 699 |

| | | |
|---|---|---|
| 24213654 | hypothetical protein LA_0954 | 441 |
| 24213656 | glutamate synthase subunit alpha | 1498 |
| 24213680 | thiamine biosynthesis protein ThiC | 495 |
| 24213707 | glutathione peroxidase | 189 |
| 24213721 | transcription termination factor Rho | 482 |
| 24213747 | diaminopimelate decarboxylase | 422 |
| 24213753 | hypothetical protein LA_1053 | 136 |
| 24213780 | aminopeptidase N | 884 |
| 24213792 | hypothetical protein LA_1092 | 160 |
| 24213799 | hypothetical protein LA_1099 | 477 |
| 24213801 | succinyl-CoA synthetase subunit alpha | 291 |
| 24213802 | succinyl-CoA synthetase subunit beta | 390 |
| 24213807 | hypothetical protein LA_1107 | 278 |
| 24213810 | adenylosuccinate synthase | 425 |
| 24213811 | ATP phosphoribosyltransferas e regulatory subunit | 344 |
| 24213812 | glycerol-3-phosphate dehydrogenase | 669 |
| 24213819 | dUTP diphosphatase | 145 |
| 24213832 | hypothetical protein LA_1132 | 80 |
| 24213836 | outer membrane lipoprotein-sorting protein | 257 |
| 24213837 | hypothetical protein LA_1137 | 371 |
| 24213838 | 2-methylthioadenine synthetase | 437 |
| 24213840 | anthranilate phosphoribosyl transferase | 336 |

| | | |
|---|---|---|
| 24213844 | pyrimidine deaminase, riboflavin biosynthesis | 466 |
| 24213847 | bifunctional 3,4-dihydroxy-2-butanone 4-phosphate synthase/GTP cyclohydrolase II | 402 |
| 24213855 | sulfate ABC transporter substrate-binding protein | 335 |
| 24213873 | acetyl-CoA synthetase | 660 |
| 24213874 | ABC transporter substrate-binding protein | 330 |
| 24213881 | histidinol-phosphate aminotransferase | 370 |
| 24213885 | signal transduction protein | 569 |
| 24213890 | hypothetical protein LA_1190 | 313 |
| 24213898 | 3-hydroxybutyryl-CoA dehydratase | 257 |
| 24213907 | thioesterase superfamily protein | 140 |
| 24213922 | dihydrolipoamide acetyltransferase | 419 |
| 24213923 | dihydrolipoamide dehydrogenase | 467 |
| 24213924 | alpha-ketoglutarate decarboxylase | 920 |
| 24213940 | threonyl-tRNA synthetase | 639 |
| 24213942 | translation initiation factor IF-3 | 179 |
| 24213943 | 50S ribosomal protein L35 | 67 |
| 24213944 | 50S ribosomal protein L20 | 117 |
| 24213945 | hypothetical protein LA_1245 | 213 |
| 24213947 | 5-formyltetrahydrofolate cyclo-ligase | 176 |
| 24213951 | chemotaxis protein histidine kinase CheA | 1070 |
| 24213952 | chemotaxis-specific methylesterase CheB | 357 |

| | | |
|---|---|---|
| 24213953 | two-component response regulator receiver protein | 120 |
| 24213955 | segregation and condensation protein B | 187 |
| 24213958 | 3-phosphoshikimate 1-carboxyvinyltransferase | 440 |
| 24213964 | fatty acid/phospholipid synthesis protein | 335 |
| 24213993 | aspartate/tyrosine/aromatic aminotransferase | 366 |
| 24214013 | glutamine synthetase | 473 |
| 24214023 | phosphoribosylformylglycinamidine (FGAM) synthase | 745 |
| 24214025 | isoleucyl-tRNA synthetase | 914 |
| 24214029 | hypothetical protein LA_1329 | 239 |
| 24214051 | capsule biosynthesis protein CapA | 342 |
| 24214059 | hypothetical protein LA_1359 | 150 |
| 24214078 | GTP-binding protein BipA | 606 |
| 24214088 | glycyl-tRNA synthetase | 464 |
| 24214091 | ribosomal protein L11 methylase | 300 |
| 24214092 | ribokinase | 328 |
| 24214098 | hypothetical protein LA_1398 | 218 |
| 24214109 | serine hydroxymethyltransferase | 415 |
| 24214117 | lactoylglutathione lyase | 152 |
| 24214120 | hypothetical protein LA_1420 | 125 |
| 24214122 | serine/threonine kinase | 1780 |
| 24214137 | glucose kinase | 298 |
| 24214149 | carboxy-terminal processing protease | 462 |
| 24214159 | UDP-glucose 6-dehydrogenase | 436 |

| | | |
|---|---|---|
| 24214180 | PLP dependent enzyme class III | 222 |
| 24214188 | glycolate oxidase | 760 |
| 24214195 | hypothetical protein LA_1495 | 331 |
| 24214211 | L-aspartate oxidase | 532 |
| 24214214 | pyridoxal phosphate biosynthetic protein PdxJ | 250 |
| 24214216 | tRNA isopentenyltransferase | 297 |
| 24214218 | mannose-1-phosphate guanylyltransferase | 353 |
| 24214232 | fructose-bisphosphate aldolase | 351 |
| 24214263 | Hsp20/alpha crystallin molecular chaperone | 130 |
| 24214278 | FAD dependent oxidoreductase | 365 |
| 24214279 | phosphoheptose isomerase | 186 |
| 24214287 | hypothetical protein LA_1587 | 264 |
| 24214305 | CMP-N-acetylneuraminic acid synthetase | 228 |
| 24214310 | UDP-N-acetylglucosamine 2-epimerase | 387 |
| 24214311 | nucleotidyl transferase | 351 |
| 24214313 | N-acetylneuraminic (sialic) acid synthetase | 753 |
| 24214316 | alcohol dehydrogenase | 355 |
| 24214317 | molybdenum cofactor biosynthesis protein | 325 |
| 24214319 | carbamoyl transferase | 602 |
| 24214325 | 3-deoxy-manno-octulosonate cytidylyltransferase | 251 |
| 24214326 | oxidoreductase family protein | 342 |

| | | |
|---|---|---|
| 24214329 | phosphoglycerate dehydrogenase and related dehydrogenase | 332 |
| 24214332 | CDP-glucose 4,6-dehydratase | 364 |
| 24214343 | polysaccharide biosynthesis protein | 344 |
| 24214354 | FdtA-like protein | 139 |
| 24214359 | dTDP-4-dehydrorhamnose 3,5-epimerase | 186 |
| 24214360 | dTDP-4-dehydrorhamnose reductase | 306 |
| 24214362 | glucose-1-phosphate thymidylyltransferase | 294 |
| 24214375 | 30S ribosomal protein S6 | 91 |
| 24214376 | single-stranded DNA-binding protein | 150 |
| 24214377 | 30S ribosomal protein S18 | 103 |
| 24214378 | 50S ribosomal protein L9 | 149 |
| 24214380 | aspartyl-tRNA synthetase | 601 |
| 24214389 | competence-damage inducible protein CinA | 418 |
| 24214396 | triosephosphate isomerase | 250 |
| 24214403 | phosphoglycerate kinase | 396 |
| 24214404 | glyceraldehyde 3-phosphate dehydrogenase | 335 |
| 24214415 | hypothetical protein LA_1715 | 357 |
| 24214419 | cysteine synthase | 309 |
| 24214425 | HD family protein | 525 |
| 24214428 | hypothetical protein LA_1728 | 282 |
| 24214553 | hypothetical protein LA_1853 | 146 |
| 24214563 | cysteinyl-tRNA synthetase | 472 |
| 24214564 | acetyl esterase | 321 |

| | | |
|---|---|---|
| 24214565 | bifunctional 5,10-methylene-tetrahydrofolate dehydrogenase/ 5,10-methylene-tetrahydrofolate cyclohydrolase | 285 |
| 24214566 | asparaginyl-tRNA synthetase | 435 |
| 24214575 | ATP-dependent helicase | 658 |
| 24214583 | hypothetical protein LA_1883 | 505 |
| 24214588 | GTP-binding protein LepA | 600 |
| 24214597 | succinate dehydrogenase flavoprotein subunit | 637 |
| 24214599 | aldo/keto oxidoreductase | 508 |
| 24214620 | RNA-binding protein | 92 |
| 24214628 | thioredoxin | 119 |
| 24214629 | CAP family transcription factor | 719 |
| 24214635 | hypothetical protein LA_1935 | 190 |
| 24214636 | hydrolase/acyltransferase | 623 |
| 24214639 | hypothetical protein LA_1939 | 159 |
| 24214647 | hypothetical protein LA_1947 | 402 |
| 24214651 | enolase | 432 |
| 24214676 | short-chain dehydrogenase | 246 |
| 24214686 | inosine-5'-monophosphate dehydrogenase | 508 |
| 24214688 | hypothetical protein LA_1988 | 230 |
| 24214695 | lysyl-tRNA synthetase | 495 |
| 24214709 | pyruvate dehydrogenase subunit beta | 324 |
| 24214710 | pyruvate dehydrogenase subunit alpha | 327 |
| 24214717 | endoflagellar filament core protein | 282 |
| 24214719 | flagellin protein | 284 |

| | | |
|---|---|---|
| 24214724 | hypothetical protein LA_2024 | 412 |
| 24214742 | methylase of chemotaxis methyl-accepting protein | 303 |
| 24214750 | undecaprenyldiphospho-muramoylpentapeptide beta- N-acetylglucosaminyltransferase | 358 |
| 24214759 | D-alanine--D-alanine ligase | 351 |
| 24214761 | homoserine O-acetyltransferase | 366 |
| 24214762 | O-acetylhomoserine/O-acetylserine sulfhydrylase | 434 |
| 24214766 | hypothetical protein LA_2066 | 276 |
| 24214771 | HflC membrane associated protease | 310 |
| 24214777 | peptidyl-prolyl cis-trans isomerase | 291 |
| 24214795 | isopropylmalate isomerase large subunit | 465 |
| 24214796 | 3-isopropylmalate dehydratase small subunit | 206 |
| 24214807 | ATP-grasp enzyme | 343 |
| 24214809 | glutaredoxin-like protein | 107 |
| 24214819 | glycerol kinase | 496 |
| 24214825 | periplasmic serine protease | 328 |
| 24214829 | DNA or RNA helicase | 569 |
| 24214830 | aspartate aminotransferase | 404 |
| 24214832 | hydrolase | 277 |
| 24214839 | malate dehydrogenase | 326 |
| 24214841 | dethiobiotin synthetase | 221 |
| 24214842 | adenosylmethionine-8-amino-7-oxononanoate aminotransferase | 433 |

| | | |
|---|---|---|
| 24214846 | DNA mismatch repair protein MutS | 848 |
| 24214851 | two-component system signal receiver | 135 |
| 24214852 | isocitrate/isopropylmalate dehydrogenase | 358 |
| 24214853 | acetylornithine aminotransferase | 406 |
| 24214864 | acyl-CoA hydrolase | 140 |
| 24214878 | N-acetyl-gamma-glutamyl-phosphate reductase | 338 |
| 24214879 | recombinase A | 366 |
| 24214884 | Zn-dependent protease | 461 |
| 24214886 | hypothetical protein LA_2186 | 1934 |
| 24214897 | 2,4-dienoyl-CoA reductase | 669 |
| 24214912 | excinuclease ABC subunit A | 948 |
| 24214922 | two component system sensor histidine kinase\response regulator | 832 |
| 24214926 | fructose-1,6-bisphosphatase | 374 |
| 24214930 | hypothetical protein LA_2230 | 148 |
| 24214931 | DNA primase | 598 |
| 24214932 | RNA polymerase sigma-70 factor | 585 |
| 24214952 | DJ-1/PfpI family intracellular protease | 181 |
| 24214980 | endoflagellar biosynthesis chaperone | 150 |
| 24214983 | bifunctional phosphoribosylaminoimidazolecarboxamide formyltransferase/IMP cyclohydrolase | 511 |
| 24214986 | translaldolase | 214 |
| 24214997 | hypothetical protein LA_2297 | 436 |
| 24215009 | long-chain-fatty-acid CoA ligase | 681 |

| | | |
|---|---|---|
| 24215023 | hypothetical protein LA_2323 | 234 |
| 24215024 | 3-ketoacyl-(acyl-carrier-protein) reductase | 256 |
| 24215029 | GTP-dependent nucleic acid-binding protein EngD | 365 |
| 24215035 | 3-polyprenyl-4-hydroxybenzoate decarboxylase | 204 |
| 24215045 | ATP-dependent protease ATP-binding subunit | 479 |
| 24215049 | peptidyl-tRNA hydrolase | 190 |
| 24215052 | hypothetical protein LA_2352 | 519 |
| 24215057 | exodeoxyribonuclease VII small subunit | 95 |
| 24215074 | type II secretory pathway ATPase protein E | 557 |
| 24215083 | response regulator with HD-GYP domain | 394 |
| 24215087 | 50S ribosomal protein L19 | 138 |
| 24215088 | tRNA (guanine-N1)-methyltransferase | 231 |
| 24215094 | ribulose-5-phosphate 3-epimerase | 215 |
| 24215095 | hypothetical protein LA_2395 | 331 |
| 24215099 | hypothetical protein LA_2399 | 283 |
| 24215103 | HPr kinase/phosphorylase | 321 |
| 24215104 | DNA-directed RNA polymerase sigma-54 subunit | 473 |
| 24215111 | ADP-heptose synthetase | 337 |
| 24215113 | cell wall-associated hydrolase/lipoprotein | 423 |
| 24215132 | biotin carboxylase-like protein | 914 |
| 24215133 | acetyl-CoA carboxylase carboxyltransferase component | 555 |

| | | |
|---|---|---|
| 24215138 | peptide deformylase | 178 |
| 24215168 | hypothetical protein LA_2468 | 444 |
| 24215169 | MCP methylation inhibitor | 154 |
| 24215183 | integrase/recombinase XerD | 298 |
| 24215194 | thioredoxin reductase | 316 |
| 24215206 | imidazoleglycerol-phosphate synthase | 256 |
| 24215207 | aspartyl/glutamyl-tRNA amidotransferase subunit A | 487 |
| 24215212 | cytoplasmic membrane lipoprotein LipL31 | 241 |
| 24215213 | transcription-repair coupling factor | 1186 |
| 24215228 | GGDEF domain receiver component of a two component response regulator | 330 |
| 24215239 | ABC transporter ATPase | 623 |
| 24215250 | FKBP-type peptidyl-prolyl cis-trans isomerase | 129 |
| 24215259 | ATP-dependent protease ClpP | 198 |
| 24215262 | glycosylhydrolase | 619 |
| 24215269 | acetolactate synthase small subunit | 161 |
| 24215270 | acetolactate synthase large subunit | 592 |
| 24215275 | hypothetical protein LA_2575 | 89 |
| 24215276 | hypothetical protein LA_2576 | 667 |
| 24215290 | flagellar switch complex protein | 323 |
| 24215323 | glutathione transferase | 218 |
| 24215325 | oligopeptidase A | 651 |
| 24215327 | MaoC family protein | 392 |
| 24215332 | hypothetical protein LA_2632 | 320 |
| 24215337 | hypothetical protein LA_2637 | 272 |
| 24215354 | co-chaperonin GroES | 96 |

| | | |
|---|---|---|
| 24215355 | chaperonin GroEL | 546 |
| 24215374 | metal-dependent amidase/aminoacylase/carboxypeptidase | 393 |
| 24215387 | aminopeptidase P | 429 |
| 24215391 | N6-adenine-specific DNA methylase | 416 |
| 24215402 | phosphoglycolate phosphatase | 224 |
| 24215406 | thiamine biosynthesis enzyme | 367 |
| 24215426 | dioxygenase | 315 |
| 24215433 | peptide chain release factor 3 | 590 |
| 24215438 | hypothetical protein LA_2738 | 369 |
| 24215459 | rod shape-determining protein MreB | 340 |
| 24215461 | exopolyphosphatase | 521 |
| 24215480 | F0F1 ATP synthase subunit delta | 186 |
| 24215508 | ABC transporter permease | 471 |
| 24215509 | peroxiredoxin | 193 |
| 24215521 | hypothetical protein LA_2821 | 134 |
| 24215534 | adenylate/guanylate cyclase | 423 |
| 24215536 | hypothetical protein LA_2836 | 155 |
| 24215540 | ornithine carbamoyltransferase | 311 |
| 24215548 | flagellar hook protein FlgE | 464 |
| 24215568 | translation initiation factor 2B | 365 |
| 24215603 | Holliday junction DNA helicase RuvA | 212 |
| 24215605 | flavin reductase-like protein | 159 |
| 24215616 | ribonuclease D | 212 |
| 24215620 | two-component response regulator | 369 |
| 24215628 | GGDEF family protein | 317 |
| 24215648 | serine protease | 484 |
| 24215650 | two-component hybrid sensor and regulator | 304 |
| 24215654 | acyltransferase | 265 |

| | | |
|---|---|---|
| 24215655 | methylmalonyl-CoA mutase | 1125 |
| 24215658 | dihydroxy-acid dehydratase | 560 |
| 24215666 | hypothetical protein LA_2967 | 104 |
| 24215687 | uridine 5-monophosphate synthase | 194 |
| 24215689 | NH(3)-dependent NAD(+) synthetase | 642 |
| 24215694 | hypothetical protein LA_2995 | 107 |
| 24215698 | single-stranded DNA exonuclease | 643 |
| 24215701 | tRNA nucleotidyltransferase/poly A polymerase | 487 |
| 24215727 | hypothetical protein LA_3028 | 685 |
| 24215732 | molybdate metabolism regulator | 276 |
| 24215738 | hypothetical protein LA_3039 | 390 |
| 24215751 | aminotransferase | 434 |
| 24215769 | anti-sigma-factor antagonist | 113 |
| 24215779 | adenylosuccinate lyase | 433 |
| 24215785 | nicotinate-nucleotide pyrophosphorylase | 304 |
| 24215793 | ferric uptake regulator | 160 |
| 24215796 | hypothetical protein LA_3097 | 555 |
| 24215817 | hypothetical protein LA_3118 | 353 |
| 24215818 | hypothetical protein LA_3119 | 322 |
| 24215843 | acyl-CoA transferase/carnitine dehydratase | 390 |
| 24215859 | 1-hydroxy-2-methyl-2-(E)-butenyl 4-diphosphate synthase | 663 |
| 24215871 | aldo/keto reductase | 278 |
| 24215891 | fruiting body developmental protein R | 279 |

| | | |
|---|---|---|
| 24215962 | hypothetical protein LA_3263 | 407 |
| 24215963 | hypothetical protein LA_3264 | 193 |
| 24215964 | hypothetical protein LA_3265 | 183 |
| 24215966 | molybdopterin oxidoreductase iron-sulfur binding subunit | 1035 |
| 24215985 | adenylate cyclase related protein | 770 |
| 24215987 | tryptophan synthase subunit alpha | 264 |
| 24215988 | tryptophan synthase subunit beta | 397 |
| 24215989 | prolyl-tRNA synthetase | 576 |
| 24215994 | ribosome recycling factor | 184 |
| 24215995 | uridylate kinase | 248 |
| 24215996 | elongation factor Ts | 199 |
| 24216004 | dihydrodipicolinate reductase | 270 |
| 24216005 | dihydrodipicolinate synthase | 307 |
| 24216011 | glyoxalase | 141 |
| 24216019 | hypothetical protein LA_3320 | 377 |
| 24216054 | hypothetical protein LA_3355 | 191 |
| 24216064 | hydroxyethylthiazole kinase | 265 |
| 24216069 | surface antigen OrfC lipoprotein | 456 |
| 24216079 | endoflagellar filament sheath protein | 239 |
| 24216098 | metallo-beta-lactamase | 275 |
| 24216099 | Zn-dependent peptidase | 557 |
| 24216105 | nucleotide-binding protein | 163 |
| 24216106 | alanyl-tRNA synthetase | 977 |
| 24216108 | 50S ribosomal protein L13 | 149 |
| 24216109 | hypothetical protein LA_3410 | 209 |

| | | |
|---|---|---|
| 24216115 | 30S ribosomal protein S7 | 157 |
| 24216116 | 30S ribosomal protein S12 | 124 |
| 24216118 | DNA-directed RNA polymerase subunit beta' | 1404 |
| 24216119 | DNA-directed RNA polymerase subunit beta | 1226 |
| 24216120 | 50S ribosomal protein L7/L12 | 127 |
| 24216121 | 50S ribosomal protein L10 | 177 |
| 24216122 | 50S ribosomal protein L1 | 230 |
| 24216123 | 50S ribosomal protein L11 | 142 |
| 24216124 | transcription antiterminator | 181 |
| 24216134 | DEAD/DEAH box helicase | 540 |
| 24216152 | hypothetical protein LA_3453 | 318 |
| 24216159 | polyphosphate kinase | 712 |
| 24216167 | outer membrane receptor for Fe3+-dicitrate/TonB-dependent receptor | 825 |
| 24216170 | hypothetical protein LA_3471 | 404 |
| 24216184 | esterase | 235 |
| 24216196 | hypothetical protein LA_3497 | 740 |
| 24216212 | phosphoribosylaminoimidazole-succinocarboxamide synthase | 285 |
| 24216213 | serine/threonine phosphatase | 377 |
| 24216221 | hypothetical protein LA_3522 | 229 |
| 24216232 | hypothetical protein LA_3533 | 162 |
| 24216256 | ribosomal protein serine acetyltransferase | 263 |
| 24216280 | dioxygenase-like protein | 257 |

| | | |
|---|---|---|
| 24216295 | ATP-dependent Lon protease | 839 |
| 24216305 | hypothetical protein LA_3606 | 150 |
| 24216324 | DNA polymerase I | 917 |
| 24216327 | anthranilate synthase component I | 473 |
| 24216331 | FOG:HEAT repeat protein | 486 |
| 24216335 | cyclic amidohydrolase | 424 |
| 24216337 | homoserine dehydrogenase | 429 |
| 24216403 | heat shock protein GrpE | 212 |
| 24216404 | chaperone protein DnaK | 646 |
| 24216406 | heat shock protein DnaJ | 372 |
| 24216443 | hypothetical protein LA_3744 | 357 |
| 24216446 | threonine synthase | 449 |
| 24216447 | hypothetical protein LA_3748 | 330 |
| 24216448 | hypothetical protein LA_3749 | 194 |
| 24216481 | glyoxalase/bleomycin resistance protein/dioxygenase | 134 |
| 24216482 | adenosine deaminase | 442 |
| 24216500 | glucosamine 6-phosphate synthetase | 610 |
| 24216506 | nitrogen regulatory protein P-II | 114 |
| 24216515 | hypothetical protein LA_3816 | 129 |
| 24216517 | hypothetical protein LA_3818 | 157 |
| 24216518 | ATP-dependent Zn protease | 655 |
| 24216520 | 50S ribosomal protein L25 | 212 |
| 24216521 | phosphoribosyl pyrophosphate synthetase | 312 |
| 24216522 | UDP-N-acetylglucosamine diphosphorylase | 252 |
| 24216538 | phospholipid binding protein | 181 |

| | | |
|---|---|---|
| 24216545 | cation/multidrug efflux pump | 1083 |
| 24216568 | response regulator containing a signal receiver domain and a DNA-binding domain | 223 |
| 24216573 | acid phosphatase | 250 |
| 24216587 | glucose-6-phosphate isomerase | 445 |
| 24216591 | nucleoside-diphosphate-sugar pyrophosphorylase | 242 |
| 24216593 | histidine kinase/response regulator hybrid protein | 507 |
| 24216602 | metallo-beta-lactamase superfamily hydrolase | 288 |
| 24216609 | hypothetical protein LA_3910 | 290 |
| 24216611 | hypothetical protein LA_3912 | 291 |
| 24216616 | hypothetical protein LA_3917 | 169 |
| 24216617 | methionyl-tRNA synthetase | 715 |
| 24216621 | hypothetical protein LA_3922 | 181 |
| 24216635 | flavoprotein | 180 |
| 24216637 | hypothetical protein LA_3938 | 861 |
| 24216646 | D-alanyl-D-alanine carboxypeptidase | 244 |
| 24216647 | UDP-glucose 4-epimerase | 323 |
| 24216648 | UDP-N-acetylglucosamine acyltransferase | 259 |
| 24216656 | 3-deoxy-D-arabino-heptulosonate 7-phosphate synthase | 373 |
| 24216657 | 3-methyl-2-oxobutanoate hydroxymethyltransferase | 265 |
| 24216658 | 2-amino-4-hydroxy-6-hydroxymethyldihydropteridine pyrophosphokinase | 149 |

| | | |
|---|---|---|
| 24216663 | GDP-mannose 4,6-dehydratase | 340 |
| 24216668 | adenosine/AMP deaminase | 442 |
| 24216676 | NTP pyrophosphohydrolase | 162 |
| 24216681 | hypothetical protein LA_3982 | 127 |
| 24216697 | cholesterol oxidase | 1135 |
| 24216734 | phenylalanyl-tRNA synthetase subunit beta | 808 |
| 24216739 | inorganic pyrophosphatase | 179 |
| 24216757 | lysine decarboxylase-like protein | 185 |
| 24216766 | isocitrate dehydrogenase | 398 |
| 24216778 | UDP-N-acetylglucosamine 1-carboxyvinyltransferase | 432 |
| 24216784 | Zn-dependent alcohol dehydrogenase | 340 |
| 24216785 | ferredoxin | 108 |
| 24216786 | hypothetical protein LA_4087 | 164 |
| 24216792 | MutT/nudix family protein | 223 |
| 24216799 | hypothetical protein LA_4100 | 148 |
| 24216802 | response regulator | 233 |
| 24216808 | hypothetical protein LA_4109 | 374 |
| 24216818 | NAD dependent DNA ligase | 681 |
| 24216830 | peptidase-like protein | 659 |
| 24216837 | 3-hydroxyacyl-CoA dehydrogenase | 436 |
| 24216849 | phenylalanine-tRNA synthetase subunit alpha | 341 |
| 24216858 | fatty acid synthase subunit beta | 385 |
| 24216865 | argininosuccinate synthase | 403 |

| | | |
|---|---|---|
| 24216866 | pantetheine-phosphate adenylyltransferase | 160 |
| 24216867 | nucleoside-diphosphate kinase | 137 |
| 24216889 | bifunctional glutamate-tRNA ligase/glutamine-tRNA ligase | 521 |
| 24216899 | L-amino acid oxidase | 447 |
| 24216918 | sulfate adenylyltransferase subunit 1 | 416 |
| 24216919 | sulfate adenylyltransferase subunit 2 | 305 |
| 24216938 | polysaccharide deacetylase | 540 |
| 24216947 | purine nucleoside phosphorylase | 287 |
| 24216954 | GTP cyclohydrolase I | 183 |
| 24216957 | hypothetical protein LA_4258 | 162 |
| 24216965 | phosphoribosylglycinamide formyltransferase 2 | 387 |
| 24216976 | metal (Ni/Fe) hydrogenase large subunit | 466 |
| 24216992 | hypothetical protein LA_4293 | 224 |
| 24216994 | hypoxanthine phosphoribosyltransferase | 183 |
| 24217038 | seryl-tRNA synthetase | 417 |
| 24217045 | ParB-like protein | 291 |
| 24217047 | glucose-inhibited division protein B | 254 |
| 24217054 | hypothetical protein LA_4355 | 276 |
| 24217060 | DNA polymerase III gamma and tau subunits | 310 |
| 161484946 | valyl-tRNA synthetase | 882 |
| 161484947 | leucyl-tRNA synthetase | 864 |
| 161484948 | F0F1 ATP synthase subunit alpha | 503 |

| | | |
|---|---|---|
| 161484952 | bifunctional GMP synthase/glutamine amidotransferase | 603 |
| 161484957 | valine--pyruvate aminotransferase | 417 |
| 229020021 | F0F1 ATP synthase subunit gamma | 286 |
| 294827557 | hypothetical protein LA_0018 | 298 |
| 294827567 | hypothetical protein LA_0071 | 156 |
| 294827570 | dioxygenase superfamily protein | 123 |
| 294827583 | hypothetical protein LA_0136 | 309 |
| 294827584 | hypothetical protein LA_0137 | 537 |
| 294827589 | malic enzyme | 420 |
| 294827600 | cytochrome c oxidase subunit II | 321 |
| 294827605 | RNA polymerase ECF-type sigma factor | 301 |
| 294827606 | hypothetical protein LA_0268 | 105 |
| 294827608 | lipoprotein releasing system permease | 457 |
| 294827612 | exonuclease | 362 |
| 294827620 | hypothetical protein LA_0323 | 142 |
| 294827621 | geranylgeranyl pyrophosphate synthase | 313 |
| 294827627 | glycine cleavage system aminomethyltransferase T | 396 |
| 294827630 | phosphoserine aminotransferase | 375 |
| 294827632 | hypothetical protein LA_0370 | 577 |
| 294827635 | hypothetical protein LA_0379 | 511 |
| 294827652 | phosphoglyceromutase | 548 |
| 294827680 | bifunctional permease/carbonic anhydrase | 741 |
| 294827690 | hypothetical protein LA_0616 | 355 |

| | | |
|---|---|---|
| 294827692 | quinolinate synthetase | 325 |
| 294827703 | hypothetical protein LA_0654 | 232 |
| 294827726 | 30S ribosomal protein S17 | 67 |
| 294827727 | 30S ribosomal protein S5 | 168 |
| 294827741 | methionine sulfoxide reductase B | 131 |
| 294827742 | biotin--[acetyl-CoA-carboxylase] ligase | 253 |
| 294827752 | NADH dehydrogenase (ubiquinone) chain E | 159 |
| 294827765 | translation initiation factor IF-2 | 863 |
| 294827769 | hypothetical protein LA_0958 | 714 |
| 294827771 | exodeoxyribonuclease V subunit alpha | 612 |
| 294827823 | carbamoyl phosphate synthase small subunit | 362 |
| 294827827 | bifunctional prephenate dehydratase/chorismate mutase | 363 |
| 294827829 | ATP phosphoribosyltransferase | 206 |
| 294827834 | hypothetical protein LA_1295 | 1010 |
| 294827842 | hypothetical protein LA_1328 | 612 |
| 294827856 | pirin-like protein | 285 |
| 294827865 | hypothetical protein LA_1412 | 334 |
| 294827875 | transcriptional repressor LexA | 203 |
| 294827885 | membrane-bound proton-translocating pyrophosphatase | 695 |
| 294827905 | thymidylate synthase | 524 |
| 294827913 | hypothetical protein LA_1586 | 317 |
| 294827920 | dTDP-4-dehydrorhamnose epimerase | 150 |
| 294827924 | dTDP-D-glucose 4,6-dehydratase | 349 |

| | | |
|---|---|---|
| 294827929 | arginyl-tRNA synthetase | 586 |
| 294827985 | hypothetical protein LA_1950 | 164 |
| 294827988 | preprotein translocase subunit SecA | 909 |
| 294827997 | hypothetical protein LA_1999 | 169 |
| 294828003 | hypothetical protein LA_2025 | 420 |
| 294828010 | UDP-N-acetylmuramoylalanyl-D-glutamate--2,6-diaminopimelate ligase | 504 |
| 294828023 | 7-cyano-7-deazaguanine reductase | 133 |
| 294828030 | gamma-glutamylcysteine synthetase | 520 |
| 294828041 | endonuclease III | 239 |
| 294828046 | hypothetical protein LA_2174 | 198 |
| 294828071 | deoxyguanosinetriphosphate triphosphohydrolase | 374 |
| 294828077 | hypothetical protein LA_2295 | 388 |
| 294828080 | UDP-3-O-[3-hydroxymyristoyl] N-acetylglucosamine deacetylase | 337 |
| 294828087 | hypothetical protein LA_2353 | 190 |
| 294828088 | ribonucleotide reductase subunit alpha | 1201 |
| 294828097 | 30S ribosomal protein S16 | 114 |
| 294828098 | methionyl-tRNA formyltransferase | 315 |
| 294828100 | 2-dehydro-3-deoxyphosphooctonate aldolase | 287 |
| 294828101 | CTP synthetase | 538 |
| 294828103 | endoflagellar hook-filament protein | 284 |

| | | |
|---|---|---|
| 294828105 | penicillin tolerance protein | 312 |
| 294828110 | hypothetical protein LA_2436 | 102 |
| 294828116 | methionyl aminopeptidase | 240 |
| 294828117 | hypothetical protein LA_2458 | 64 |
| 294828118 | polymerase | 572 |
| 294828130 | hypothetical protein LA_2531 | 143 |
| 294828134 | phosphate sodium symporter | 763 |
| 294828136 | acyl-CoA thioesterase | 204 |
| 294828147 | S-adenosylmethionine synthetase | 412 |
| 294828160 | bacterioferritin | 155 |
| 294828162 | Fe-S oxidoreductase | 696 |
| 294828167 | hypothetical protein LA_2726 | 119 |
| 294828170 | acetyl-CoA carboxylase biotin carboxyl carrier protein subunit | 166 |
| 294828180 | F0F1 ATP synthase subunit beta | 467 |
| 294828187 | hypothetical protein LA_2800 | 368 |
| 294828188 | methyl-accepting chemotaxis protein | 557 |
| 294828193 | citrate lyase | 330 |
| 294828212 | pyruvate kinase | 475 |
| 294828215 | phosphoribosylaminoimi dazole synthetase | 370 |
| 294828218 | thiosulfate sulfurtransferase | 285 |
| 294828227 | Fe-S metabolism protein | 132 |
| 294828235 | nickel ABC transporter periplasmic protein OppA | 599 |
| 294828236 | guanosine polyphosphate pyrophosphohydrolase/s ynthetase | 674 |
| 294828237 | hypothetical protein LA_3091 | 245 |
| 294828265 | hypothetical protein LA_3200b | 497 |

| | | |
|---|---|---|
| 294828269 | hypothetical protein LA_3219 | 274 |
| 294828285 | 1-deoxy-D-xylulose 5-phosphate reductoisomerase | 389 |
| 294828286 | 30S ribosomal protein S2 | 289 |
| 294828301 | transcript cleavage factor/unknown domain fusion protein | 926 |
| 294828302 | flagellar filament sheath protein | 315 |
| 294828310 | hypothetical protein LA_3405 | 129 |
| 294828311 | 30S ribosomal protein S9 | 132 |
| 294828319 | riboflavin biosynthesis protein | 318 |
| 294828320 | FAD/FMN-containing dehydrogenase | 474 |
| 294828339 | hypothetical protein LA_3534 | 158 |
| 294828340 | activator of Hsp90 ATPase 1 family protein | 153 |
| 294828347 | selenocysteine lyase | 413 |
| 294828352 | DNA-binding ferritin-like protein | 160 |
| 294828362 | anthranilate synthase component II | 192 |
| 294828371 | uracil-DNA glycosylase | 218 |
| 294828376 | hypothetical protein LA_3697 | 674 |
| 294828378 | antimicrobial peptide ABC transporter ATPase | 260 |
| 294828390 | phosphoribosylamine--glycine ligase | 422 |
| 294828422 | hypothetical protein LA_3911 | 186 |
| 294828432 | hypothetical protein LA_3961 | 303 |
| 294828446 | hypothetical protein LA_4025 | 264 |
| 294828448 | organic radical activating enzyme | 237 |
| 294828454 | ankyrin repeat-containing protein | 338 |

| | | |
|---|---|---|
| 294828460 | apolipoprotein n-acyltransferase | 609 |
| 294828463 | amidophosphoribosyltransferase | 446 |
| 294828465 | bifunctional ornithine acetyltransferase/N-acetylglutamate synthase | 383 |
| 294828475 | hypothetical protein LA_4154 | 672 |
| 294828487 | hypothetical protein LA_4185 | 365 |
| 294828493 | ferredoxin-like sulfite reductase | 561 |
| 294828498 | ketol-acid reductoisomerase | 333 |
| 294828513 | carboxypeptidase I | 514 |
| 294828514 | ferredoxin--NADP reductase | 310 |
| 294828515 | DNA polymerase III subunit gamma | 479 |
| 294828518 | branched-chain amino acid aminotransferase | 307 |
| 24212703 | recombination protein RecF | 365 |
| 24212711 | outer membrane lipoprotein LipL21 | 186 |
| 24212742 | glucose-methanol-choline oxidoreductase | 537 |
| 24212767 | hypothetical protein LA_0067 | 1061 |
| 24212817 | hypothetical protein LA_0117 | 340 |
| 24212848 | hypothetical protein LA_0147 | 408 |
| 24212861 | NADH dehydrogenase (ubiquinone) chain I | 175 |
| 24212866 | hypothetical protein LA_0166 | 457 |
| 24212974 | lipoprotein releasing system LolD | 225 |
| 24213010 | regulatory-like cAMP-binding protein | 351 |
| 24213030 | penicillin G acylase precursor | 838 |
| 24213074 | hypothetical protein LA_0374 | 696 |
| 24213099 | paraoxonase | 353 |

| | | |
|---|---|---|
| 24213133 | hypothetical protein LA_0433 | 181 |
| 24213141 | hypothetical protein LA_0441 | 672 |
| 24213162 | hypothetical protein LA_0462 | 268 |
| 24213275 | hypothetical protein LA_0575 | 335 |
| 24213450 | 50S ribosomal protein L24 | 119 |
| 24213463 | 30S ribosomal protein S11 | 136 |
| 24213492 | hypothetical protein LA_0792 | 437 |
| 24213503 | hypothetical protein LA_0803 | 128 |
| 24213523 | alpha/beta hydrolase-like protein | 303 |
| 24213578 | hypothetical protein LA_0878 | 350 |
| 24213589 | NADH dehydrogenase (ubiquinone) chain H | 346 |
| 24213651 | hypothetical protein LA_0951 | 287 |
| 24213657 | TolC-like outer membrane efflux protein | 557 |
| 24213745 | membrane carboxypeptidase/penicillin-binding protein | 827 |
| 24213752 | hypothetical protein LA_1052 | 459 |
| 24213841 | preprotein translocase subunit YajC | 108 |
| 24213892 | hypothetical protein LA_1192 | 523 |
| 24213906 | hypothetical protein LA_1206 | 195 |
| 24213961 | hypothetical protein LA_1261 | 247 |
| 24213980 | phosphoglycerol transferase-like protein | 662 |
| 24214003 | GTP-binding protein EngA | 489 |
| 24214026 | hypothetical protein LA_1326 | 246 |

| | | |
|---|---|---|
| 24214048 | histidine kinase sensor protein | 462 |
| 24214084 | hypothetical protein LA_1384 | 306 |
| 24214097 | export protein | 984 |
| 24214140 | hypothetical protein LA_1440 | 519 |
| 24214146 | heavy metal efflux pump | 393 |
| 24214221 | chemotaxis protein CheW | 154 |
| 24214253 | hypothetical protein LA_1553 | 673 |
| 24214303 | glycosyltransferase | 316 |
| 24214352 | hypothetical protein LA_1652 | 297 |
| 24214374 | poly-gamma-glutamate synthesis protein | 409 |
| 24214433 | hypothetical protein LA_1733 | 387 |
| 24214434 | anti-sigma factor antagonist-like protein | 310 |
| 24214596 | succinate dehydrogenase/fumarate reductase iron-sulfur subunit | 243 |
| 24214598 | succinate:quinone oxidoreductase (SQR)-like protein | 226 |
| 24214627 | hypothetical protein LA_1927 | 269 |
| 24214657 | hypothetical protein LA_1957 | 191 |
| 24214677 | FAD-binding oxidoreductase | 500 |
| 24214681 | hypothetical protein LA_1981 | 540 |
| 24214696 | hypothetical protein LA_1996 | 216 |
| 24214753 | hypothetical protein LA_2053 | 182 |
| 24214805 | gamma-glutamyltranspeptidase | 577 |
| 24214854 | phosphomannomutase | 587 |

| | | |
|---|---|---|
| 24214873 | lipopolysaccharide core biosynthesis protein | 353 |
| 24214944 | hypothetical protein LA_2244 | 166 |
| 24214955 | hypothetical protein LA_2255 | 356 |
| 24214962 | alkylglycerone-phosphate synthase | 563 |
| 24214968 | hypothetical protein LA_2268 | 426 |
| 24215000 | hypothetical protein LA_2300 | 226 |
| 24215001 | hypothetical protein LA_2301 | 224 |
| 24215016 | hypothetical protein LA_2316 | 146 |
| 24215020 | biopolymer transport protein TolQ | 201 |
| 24215037 | chromosome partitioning ATPase | 347 |
| 24215067 | actin-like ATPase involved in cell division | 540 |
| 24215076 | type II secretory pathway component protein C | 303 |
| 24215156 | hypothetical protein LA_2456 | 305 |
| 24215210 | hypothetical protein LA_2510 | 991 |
| 24215215 | histidinol dehydrogenase | 427 |
| 24215237 | hypothetical protein LA_2537 | 705 |
| 24215284 | hypothetical protein LA_2584 | 353 |
| 24215291 | flagellar MS-ring protein | 565 |
| 24215313 | endoflagellar motor switch protein | 354 |
| 24215364 | flagellar basal body P-ring protein | 366 |
| 24215372 | hypothetical protein LA_2672 | 338 |
| 24215419 | hypothetical protein LA_2719 | 768 |
| 24215455 | transpeptidase/penicillin binding protein | 647 |

| | | |
|---|---|---|
| 24215481 | F0F1 ATP synthase subunit B | 173 |
| 24215559 | hypothetical protein LA_2859 | 173 |
| 24215617 | alpha-galactosidase | 646 |
| 24215703 | prolipoprotein diacylglyceryl transferase | 318 |
| 24215819 | zinc-bindin carboxypeptidase | 506 |
| 24215865 | metallophosphoesterase | 280 |
| 24215961 | hypothetical protein LA_3262 | 159 |
| 24215967 | hypothetical protein LA_3268 | 168 |
| 24215990 | zinc metalloprotease | 575 |
| 24216002 | hypothetical protein LA_3303 | 351 |
| 24216140 | leucyl aminopeptidase | 495 |
| 24216231 | hypothetical protein LA_3532 | 130 |
| 24216234 | hypothetical protein LA_3535 | 182 |
| 24216270 | hypothetical protein LA_3571 | 139 |
| 24216274 | flagellar basal body-associated protein FliL | 174 |
| 24216312 | 2-dehydropantoate 2-reductase | 348 |
| 24216330 | receiver component of a two-component response regulator | 137 |
| 24216411 | lysophospholipase L1 biosynthesis ABC transporter permease | 845 |
| 24216451 | hypothetical protein LA_3752 | 511 |
| 24216453 | signal peptidase I | 198 |
| 24216461 | hypothetical protein LA_3762 | 142 |
| 24216464 | hypothetical protein LA_3765 | 281 |
| 24216569 | hypothetical protein LA_3870 | 554 |
| 24216612 | hypothetical protein LA_3913 | 188 |
| 24216623 | NAD(P)H steroid dehydrogenase | 321 |

| | | |
|---|---|---|
| 24216636 | hemolysin-like protein | 444 |
| 24216698 | choline dehydrogenase | 522 |
| 24216850 | UDP-N-acetylmuramate--L-alanine ligase | 465 |
| 24216852 | hypothetical protein LA_4153 | 367 |
| 24216877 | GMC oxidoreductase | 518 |
| 24216971 | hypothetical protein LA_4272 | 650 |
| 24216977 | metal (Ni/Fe) hydrogenase small subunit | 273 |
| 24217048 | metallopeptidase | 482 |
| 161484954 | 30S ribosomal protein S15 | 88 |
| 294827558 | glutaredoxin family protein | 118 |
| 294827566 | hypothetical protein LA_0070 | 987 |
| 294827572 | 1-acylglycerol-3-phosphate O-acyltransferase | 234 |
| 294827581 | hypothetical protein LA_0129 | 607 |
| 294827588 | hypothetical protein LA_0172 | 780 |
| 294827598 | hypothetical protein LA_0234 | 334 |
| 294827624 | glycerophosphoryl diester phosphodiesterase | 323 |
| 294827639 | hypothetical protein LA_0389 | 304 |
| 294827654 | hypothetical protein LA_0444 | 349 |
| 294827739 | hypothetical protein LA_0818 | 334 |
| 294827744 | dolichyl-phosphate-mannose-protein mannosyltransferase | 578 |
| 294827748 | aldehyde dehydrogenase | 487 |
| 294827762 | 4-hydroxybenzoate transporter transmembrane protein | 460 |

| | | |
|---|---|---|
| 294827801 | preprotein translocase subunit SecD | 642 |
| 294827802 | preprotein translocase subunit SecF | 312 |
| 294827804 | sulfate ABC transporter permease | 276 |
| 294827812 | hypothetical protein LA_1196 | 341 |
| 294827859 | hypothetical protein LA_1396 | 177 |
| 294827896 | hypothetical protein LA_1507 | 527 |
| 294827907 | sodium/hydrogen antiporter | 741 |
| 294827987 | hypothetical protein LA_1958 | 203 |
| 294827993 | cation efflux system protein | 1109 |
| 294828020 | hypothetical protein LA_2080 | 325 |
| 294828035 | SGNH-hydrolase | 248 |
| 294828058 | endoflagellar motor protein B | 336 |
| 294828059 | magnesium transporter | 463 |
| 294828084 | glucose/sorbosone dehydrogenase | 457 |
| 294828092 | type II secretory pathway component protein D | 596 |
| 294828123 | hypothetical protein LA_2497 | 484 |
| 294828143 | sodium:alanine symporter/phosphatidylserine decarboxylase | 741 |
| 294828152 | biotin carboxylase | 410 |
| 294828153 | flagellar basal body rod protein FlgG | 264 |
| 294828159 | lipopolysaccharide heptosyltransferase II | 343 |
| 294828172 | hypothetical protein LA_2746 | 609 |
| 294828177 | cGMP-specific phosphodiesterase | 718 |
| 294828195 | MhpC-like protein | 335 |
| 294828247 | OmpL1 | 320 |

| | | |
|---|---|---|
| 294828249 | alpha/beta hydrolase superfamily protein | 277 |
| 294828279 | Fe-S-cluster-containing hydrogenase | 455 |
| 294828309 | hypothetical protein LA_3397 | 259 |
| 294828315 | hypothetical protein LA_3433 | 261 |
| 294828363 | multidrug ABC transporter ATPase/permease | 606 |
| 294828368 | ketosteroid isomerase-like protein | 156 |
| 294828369 | hypothetical protein LA_3664 | 368 |
| 294828439 | hypothetical protein LA_4000 | 236 |
| 294828449 | lauroyl/myristoyl acyltransferase | 300 |
| 294828461 | hypothetical protein LA_4084 | 183 |
| 45655587 | 5,10 methylene tetrahydrofolate reductase | 310 |
| 45655588 | hypothetical protein LIC20005 | 274 |
| 45655592 | porphobilinogen deaminase | 547 |
| 45655593 | delta-aminolevulinic acid dehydratase | 317 |
| 45655594 | glutamate-1-semialdehyde aminotransferase | 443 |
| 45655597 | uroporphyrinogen decarboxylase | 340 |
| 45655598 | coproporphyrinogen III oxidase | 443 |
| 45655605 | azoreductase | 200 |
| 45655611 | NADH dehydrogenase | 422 |
| 45655613 | hypothetical protein LIC20030 | 202 |
| 45655615 | hypothetical protein LIC20032 | 140 |
| 45655617 | hypothetical protein LIC20034 | 356 |

| | | |
|---|---|---|
| 45655618 | hypothetical protein LIC20035 | 440 |
| 45655620 | methanol dehydrogenase regulator | 357 |
| 45655627 | heat shock protein 90 | 607 |
| 45655628 | hypothetical protein LIC20045 | 189 |
| 45655645 | phosphoribosyl-ATP pyrophosphatase | 92 |
| 45655647 | 3-oxoacyl-(acyl-carrier protein) reductase | 254 |
| 45655648 | acyl carrier protein | 77 |
| 45655661 | hypothetical protein LIC20078 | 171 |
| 45655663 | 4-hydroxybenzoyl-CoA thioesterase | 142 |
| 45655666 | S-adenosyl-L-homocysteine hydrolase | 436 |
| 45655668 | B12-dependent methionine synthase | 1247 |
| 45655671 | diphosphate--fructose-6-phosphate 1-phosphotransferase | 431 |
| 45655676 | bacterioferritin comigratory protein | 163 |
| 45655679 | hypothetical protein LIC20096 | 365 |
| 45655683 | hypothetical protein LIC20100 | 283 |
| 45655685 | pheromone shutdown protein | 408 |
| 45655697 | LipL45 homologue | 363 |
| 45655720 | capsular polysaccharide biosynthesis protein | 629 |
| 45655723 | heat shock protein HtpX | 295 |
| 45655724 | adenine phosphoribosyltransferase | 177 |
| 45655725 | HtrA1 | 504 |
| 45655726 | HtrA1-like protein | 527 |
| 45655728 | hypothetical protein LIC20146 | 525 |

| | | |
|---|---|---|
| 45655740 | ATP-dependent protease | 540 |
| 45655747 | hypothetical protein LIC20165 | 386 |
| 45655749 | hypothetical protein LIC20167 | 356 |
| 45655754 | lipoprotein | 717 |
| 45655757 | dnaK suppressor | 150 |
| 45655767 | hypothetical protein LIC20185 | 233 |
| 45655772 | hypothetical protein LIC20190 | 485 |
| 45655775 | 4-hydroxybutyrate CoA transferase | 429 |
| 45655776 | hypothetical protein LIC20196 | 172 |
| 45655777 | cysteine protease | 799 |
| 45655779 | UTP-glucose-1-phosphate uridyltransferase | 472 |
| 45655784 | cysteine desulfurase | 383 |
| 45655785 | hypothetical protein LIC20205 | 289 |
| 45655788 | methylmalonyl-COA mutase small subunit | 626 |
| 45655794 | hypothetical protein LIC20214 | 843 |
| 45655796 | ExbB | 204 |
| 45655803 | hypothetical protein LIC20223 | 143 |
| 45655805 | 3-oxoacyl-[acyl-carrier protein] reductase | 232 |
| 45655807 | acireductone dioxygenase enzyme ARD and ARD' | 177 |
| 45655808 | phosphate ABC transporter periplasmic phosphate-binding protein | 299 |
| 45655814 | S-adenosylmethionine decarboxylase like protein | 288 |
| 45655816 | hypothetical protein LIC20240 | 180 |
| 45655817 | alanine racemase | 389 |
| 45655818 | putative acyltransferase | 381 |

| | | |
|---|---|---|
| 45655819 | hypothetical protein LIC20243 | 303 |
| 45655824 | aconitate hydratase | 757 |
| 45655825 | peptidoglycan-associated cytoplasmic membrane protein | 383 |
| 45655841 | aspartate-semialdehyde dehydrogenase | 349 |
| 45655847 | hypothetical protein LIC20272 | 357 |
| 45655849 | hypothetical protein LIC20274 | 250 |
| 45655850 | hypothetical protein LIC20275 | 281 |
| 45655851 | hypothetical protein LIC20276 | 312 |
| 45655915 | chromosomal replication initiator protein | 443 |
| 45655916 | DNA polymerase III beta subunit | 373 |
| 45655923 | putative lipoprotein | 245 |
| 45655925 | LipL21 | 186 |
| 45655931 | hypothetical protein LIC10017 | 301 |
| 45655933 | 3-oxoacyl-acyl carrier protein reductase | 247 |
| 45655934 | hypothetical protein LIC10020 | 202 |
| 45655936 | arsenate reductase | 120 |
| 45655941 | hypothetical protein LIC10027 | 234 |
| 45655943 | inositol monophosphatase | 282 |
| 45655952 | hypothetical protein LIC10038 | 502 |
| 45655959 | dTDP-glucose 4-6-dehydratase | 312 |
| 45655960 | proton-translocating transhydrogenase, subunit alpha part 1 | 389 |
| 45655974 | hypothetical protein LIC10060 | 1061 |
| 45655978 | hypothetical protein LIC10064 | 160 |

| | | |
|---|---|---|
| 45655980 | enoyl-CoA hydratase/isomerase family protein | 260 |
| 45655983 | glyoxylase I | 132 |
| 45655987 | 3-demethylubiquinone-9 3-methyltransferase | 283 |
| 45655997 | RNA methyltransferase | 415 |
| 45656006 | hypothetical protein LIC10095 | 118 |
| 45656016 | hypothetical protein LIC10105 | 340 |
| 45656022 | imidazoleglycerol-phosphate dehydratase | 206 |
| 45656024 | phosphoribosylformimino-5-aminoimidazole carboxamide ribotide isomerase protein | 241 |
| 45656025 | hypothetical protein LIC10115 | 404 |
| 45656027 | hypothetical protein LIC10117 | 616 |
| 45656028 | phoshomethylpyrimidine kinase protein | 273 |
| 45656032 | hypothetical protein LIC10122 | 433 |
| 45656033 | LipL45-like protein | 330 |
| 45656034 | hypothetical protein LIC10124 | 551 |
| 45656035 | hypothetical protein LIC10125 | 1197 |
| 45656037 | hypothetical protein LIC10127 | 312 |
| 45656042 | sigma 54 activator | 699 |
| 45656044 | hypothetical protein LIC10134 | 408 |
| 45656048 | HD-GYP hydrolase domain-containing protein | 489 |
| 45656051 | NADH dehydrogenase I G subunit | 482 |
| 45656052 | NADH dehydrogenase I I subunit | 175 |
| 45656055 | hypothetical protein LIC10145 | 457 |

| | | |
|---|---|---|
| 45656063 | hypothetical protein LIC10157 | 627 |
| 45656064 | hypothetical protein LIC10158 | 236 |
| 45656067 | hypothetical protein LIC10161 | 699 |
| 45656074 | hypothetical protein LIC10168 | 132 |
| 45656081 | hypothetical protein LIC10175 | 495 |
| 45656096 | peptidoglycan associated cytoplasmic membrane protein | 195 |
| 45656113 | cytochrome c oxidase polypeptide II | 333 |
| 45656116 | hypothetical protein LIC10211 | 99 |
| 45656119 | CarD transcriptional regulator | 197 |
| 45656120 | hypothetical protein LIC10215 | 343 |
| 45656131 | hypothetical protein LIC10226 | 242 |
| 45656132 | hypothetical protein LIC10227 | 609 |
| 45656136 | hypothetical protein LIC10231 | 337 |
| 45656137 | lipoprotein releasing system transmembrane protein | 458 |
| 45656140 | hypothetical protein LIC10235 | 94 |
| 45656142 | cyclic nucleotide binding protein | 405 |
| 45656146 | hypothetical protein LIC10241 | 231 |
| 45656174 | chlorohydrolase family protein | 416 |
| 45656186 | zinc-binding dehydrogenase family protein | 340 |
| 45656187 | farnesyl-diphosphate synthase | 332 |
| 45656194 | H+-translocating transhydrogenase subunit beta | 466 |
| 45656206 | hypothetical protein LIC10302 | 349 |

| | | |
|---|---|---|
| 45656218 | hypothetical protein LIC10314 | 557 |
| 45656221 | ribose 5-phosphate isomerase B | 145 |
| 45656222 | hypothetical protein LIC10318 | 640 |
| 45656225 | hypothetical protein LIC10321 | 696 |
| 45656229 | hemolysin | 378 |
| 45656230 | hypothetical protein LIC10326 | 524 |
| 45656241 | hypothetical protein LIC10337 | 369 |
| 45656243 | hemolysin B | 846 |
| 45656253 | hypothetical protein LIC10350 | 160 |
| 45656256 | hypothetical protein LIC10353 | 503 |
| 45656263 | electron transfer flavoprotein alpha-subunit | 319 |
| 45656264 | electron transport flavoprotein beta subunit | 253 |
| 45656280 | hypothetical protein LIC10380 | 181 |
| 45656284 | hypothetical protein LIC10384 | 672 |
| 45656302 | hypothetical protein LIC10402 | 268 |
| 45656303 | riboflavin synthase beta chain | 151 |
| 45656305 | hypothetical protein LIC10405 | 688 |
| 45656306 | hypothetical protein LIC10406 | 438 |
| 45656311 | hypothetical protein LIC10411 | 157 |
| 45656319 | hypothetical protein LIC10419 | 318 |
| 45656325 | hypothetical protein LIC10425 | 314 |
| 45656327 | UDP-N-acetylglucosamine pyrophosphorylase | 252 |
| 45656329 | 50S ribosomal protein L25/general stress protein Ctc | 212 |

| | | |
|---|---|---|
| 45656331 | cell division protein | 655 |
| 45656333 | hypothetical protein LIC10434 | 121 |
| 45656339 | nitrogen regulatory protein PII | 114 |
| 45656342 | 3-methylcrotonoyl-CoA carboxylase beta subunit | 536 |
| 45656344 | glutamine-fructose-6-phosphate transaminase | 610 |
| 45656358 | hypothetical protein LIC10460 | 134 |
| 45656368 | peptide chain release factor 2 | 367 |
| 45656372 | hypothetical protein LIC10474 | 142 |
| 45656378 | hypothetical protein LIC10480 | 511 |
| 45656380 | hypothetical protein LIC10482 | 193 |
| 45656381 | hypothetical protein LIC10483 | 330 |
| 45656384 | hypothetical protein LIC10486 | 357 |
| 45656416 | ABC transporter, atp-binding protein | 263 |
| 45656417 | hypothetical protein LIC10519 | 845 |
| 45656419 | hypothetical protein LIC10521 | 182 |
| 45656421 | hypothetical protein LIC10523 | 372 |
| 45656422 | hypothetical protein LIC10524 | 646 |
| 45656423 | hypothetical protein LIC10525 | 212 |
| 45656427 | hypothetical protein LIC10529 | 714 |
| 45656444 | lipase | 357 |
| 45656449 | hypothetical protein LIC10551 | 371 |
| 45656452 | hypothetical protein LIC10554 | 164 |
| 45656476 | hypothetical protein LIC10578 | 486 |
| 45656499 | hypothetical protein LIC10601 | 150 |

| | | |
|---|---|---|
| 45656504 | DNA-binding stress protein | 172 |
| 45656506 | ATP-dependent protease La | 839 |
| 45656522 | flagellar protein | 174 |
| 45656532 | selenocysteine reductase | 416 |
| 45656538 | hypothetical protein LIC10640 | 334 |
| 45656560 | hypothetical protein LIC10662 | 182 |
| 45656561 | hypothetical protein LIC10663 | 159 |
| 45656562 | hypothetical protein LIC10664 | 162 |
| 45656563 | hypothetical protein LIC10665 | 130 |
| 45656570 | hypothetical protein LIC10672 | 229 |
| 45656579 | phosphoribosylformylglycinamidine synthase | 219 |
| 45656588 | hypothetical protein LIC10690 | 740 |
| 45656597 | hypothetical protein LIC10699 | 235 |
| 45656598 | 3-oxoacyl-[acyl-carrier-protein] reductase oxidoreductase | 259 |
| 45656612 | outer membrane receptor protein | 825 |
| 45656616 | hypothetical protein LIC10718 | 481 |
| 45656622 | hypothetical protein LIC10724 | 318 |
| 45656629 | cytosol aminopeptidase protein | 495 |
| 45656634 | ATP-dependent RNA helicase | 540 |
| 45656636 | hypothetical protein LIC10740 | 263 |
| 45656642 | transcription antitermination protein | 181 |
| 45656654 | hypothetical protein LIC10760 | 209 |
| 45656659 | hypothetical protein LIC10765 | 146 |

| | | |
|---|---|---|
| 45656660 | hypothetical protein LIC10766 | 144 |
| 45656662 | metalloprotease | 542 |
| 45656663 | processing metalloprotease | 557 |
| 45656664 | hypothetical protein LIC10770 | 275 |
| 45656682 | fused transcript cleavage factor/uncharacterized domain-containing protein | 926 |
| 45656686 | surface antigen | 456 |
| 45656698 | glutathione S-transferase | 164 |
| 45656699 | hypothetical protein LIC10808 | 191 |
| 45656700 | zinc binding dehydrogenase | 348 |
| 45656719 | hypothetical protein LIC10828 | 378 |
| 45656724 | hypothetical protein LIC10833 | 170 |
| 45656736 | hypothetical protein LIC10845 | 351 |
| 45656739 | hypothetical protein LIC10848 | 125 |
| 45656748 | integral membrane zinc metalloprotease | 575 |
| 45656764 | hypothetical protein LIC10873 | 168 |
| 45656765 | molybdopterin oxidoreductase | 1035 |
| 45656766 | molybdopterin oxidoredutase membrane subunit | 457 |
| 45656767 | hypothetical protein LIC10876 | 183 |
| 45656768 | hypothetical protein LIC10877 | 162 |
| 45656769 | hypothetical protein LIC10878 | 407 |
| 45656802 | transketolase alpha subunit protein | 288 |
| 45656824 | fruiting body developmental protein | 279 |
| 45656838 | sigma factor regulatory protein | 569 |

| | | |
|---|---|---|
| 45656857 | alpha-methylacyl-CoA racemase | 390 |
| 45656861 | outer membrane protein | 320 |
| 45656872 | hypothetical protein LIC10984 | 322 |
| 45656873 | hypothetical protein LIC10985 | 353 |
| 45656890 | LipL71 | 555 |
| 45656896 | hypothetical protein LIC11009 | 249 |
| 45656899 | hypothetical protein LIC11013 | 599 |
| 45656903 | hypothetical protein LIC11017 | 260 |
| 45656930 | hypothetical protein LIC11046 | 400 |
| 45656935 | hypothetical protein LIC11051 | 685 |
| 45656949 | poly A polymerase | 450 |
| 45656955 | hypothetical protein LIC11071 | 107 |
| 45656994 | histidine kinase response regulator hybrid protein | 304 |
| 45657031 | nitrilotriacetate monooxygenase component B | 159 |
| 45657032 | DNA helicase subunit A | 212 |
| 45657057 | hypothetical protein LIC11173 | 499 |
| 45657059 | translation initiation factor | 364 |
| 45657066 | hypothetical protein LIC11182 | 173 |
| 45657067 | hypothetical protein LIC11183 | 348 |
| 45657073 | hypothetical protein LIC11189 | 660 |
| 45657078 | putative citrate lyase | 330 |
| 45657080 | hypothetical protein LIC11196 | 155 |
| 45657092 | hypothetical protein LIC11209 | 134 |
| 45657099 | methyl-accepting chemotaxis transducer transmembrane protein | 574 |

| | | |
|---|---|---|
| 45657107 | hypothetical protein LIC11224 | 383 |
| 45657121 | ATP synthase C chain | 108 |
| 45657141 | hypothetical protein LIC11258 | 340 |
| 45657144 | penicillin-binding protein | 647 |
| 45657150 | periplasmic sulfate-binding protein | 334 |
| 45657151 | hypothetical protein LIC11268 | 609 |
| 45657153 | hypothetical protein LIC11270 | 429 |
| 45657156 | hypothetical protein LIC11274 | 369 |
| 45657166 | hypothetical protein LIC11284 | 120 |
| 45657167 | hypothetical protein LIC11285 | 315 |
| 45657174 | hypothetical protein LIC11294 | 699 |
| 45657178 | hypothetical protein LIC11298 | 367 |
| 45657189 | hypothetical protein LIC11309 | 416 |
| 45657191 | acetyl-CoA C-acyltransferase | 385 |
| 45657198 | N-acyl-L-amino acid amidohydrolase | 393 |
| 45657209 | ribonuclease BN transmembrane protein | 749 |
| 45657210 | hypothetical protein LIC11332 | 422 |
| 45657222 | hypothetical protein LIC11344 | 222 |
| 45657230 | LipL32 | 272 |
| 45657233 | transketolase | 320 |
| 45657237 | hypothetical protein LIC11359 | 392 |
| 45657246 | sodium/alanine symporter family protein | 713 |
| 45657247 | hypothetical protein LIC11369 | 108 |
| 45657248 | flagellar motor switch protein | 354 |
| 45657275 | hypothetical protein LIC11398 | 353 |

| | | |
|---|---|---|
| 45657282 | hypothetical protein LIC11405 | 667 |
| 45657285 | hypothetical protein LIC11408 | 369 |
| 45657287 | acetolactate synthase 3 regulatory subunit | 161 |
| 45657290 | glycosyl hydrolase | 605 |
| 45657293 | ATP-dependent Clp protease, proteolytic subunit | 198 |
| 45657299 | glycine rich RNA-binding protein | 131 |
| 45657312 | hypothetical protein LIC11436 | 705 |
| 45657317 | hypothetical protein LIC11441 | 158 |
| 45657331 | LipL31 | 241 |
| 45657336 | glutamyl-tRNA(Gln) amidotransferase subunit A | 487 |
| 45657337 | hypothetical protein LIC11462 | 256 |
| 45657343 | hypothetical protein LIC11468 | 489 |
| 45657363 | chemotaxis protein | 154 |
| 45657364 | hypothetical protein LIC11489 | 444 |
| 45657365 | hypothetical protein LIC11490 | 249 |
| 45657371 | hypothetical protein LIC11496 | 98 |
| 45657373 | hypothetical protein LIC11498 | 1137 |
| 45657374 | hypothetical protein LIC11499 | 80 |
| 45657375 | methionine aminopeptidase | 252 |
| 45657385 | putative polypeptide deformylase protein | 178 |
| 45657387 | hypothetical protein LIC11514 | 122 |
| 45657388 | hypothetical protein LIC11515 | 938 |
| 45657390 | acetyl-CoA carboxylase alpha subunit | 555 |

| | | |
|---|---|---|
| 45657391 | biotin carboxylase subunit of acetyl CoA carboxylase | 914 |
| 45657409 | hypothetical protein LIC11536 | 423 |
| 45657411 | ADP-heptose synthase | 337 |
| 45657423 | hypothetical protein LIC11550 | 283 |
| 45657436 | hypothetical protein LIC11563 | 394 |
| 45657442 | hypothetical protein LIC11569 | 302 |
| 45657443 | general secretory pathway protein D | 596 |
| 45657444 | general secretory pathway protein E | 557 |
| 45657446 | general secretory pathway protein G | 158 |
| 45657451 | hypothetical protein LIC11578 | 540 |
| 45657453 | hypothetical protein LIC11580 | 313 |
| 45657460 | ribonucleotide-diphosphate reductase subunit alpha | 1201 |
| 45657462 | exodeoxyribonuclease small subunit | 95 |
| 45657467 | hypothetical protein LIC11595 | 519 |
| 45657469 | 2-isopropylmalate synthase 2 | 516 |
| 45657472 | ATP-dependent protease peptidase subunit | 180 |
| 45657474 | hypothetical protein LIC11602 | 501 |
| 45657479 | cell division ATPase | 347 |
| 45657481 | 3-octaprenyl-4-hydroxybenzoate carboxylase | 204 |
| 45657485 | translation-associated GTPase | 365 |
| 45657488 | ADP-L-glycero-D-mannoheptose-6-epimerase | 326 |

| | | |
|---|---|---|
| 45657489 | ArsR family transcriptional regulator | 99 |
| 45657491 | hypothetical protein LIC11619 | 234 |
| 45657493 | biopolymer transport protein | 201 |
| 45657494 | biopolymer transport exbd-related transmembrane protein | 149 |
| 45657497 | hypothetical protein LIC11625 | 146 |
| 45657506 | CsgA | 222 |
| 45657508 | hypothetical protein LIC11636 | 224 |
| 45657509 | hypothetical protein LIC11637 | 226 |
| 45657513 | hypothetical protein LIC11641 | 436 |
| 45657515 | LipL45 | 390 |
| 45657519 | pseudouridylate synthase | 246 |
| 45657520 | hypothetical protein LIC11650 | 341 |
| 45657522 | putative translaldolase | 214 |
| 45657527 | hypothetical protein LIC11657 | 150 |
| 45657544 | TetR family transcriptional regulator | 217 |
| 45657548 | hypothetical protein LIC11682 | 356 |
| 45657551 | hypothetical protein LIC11685 | 181 |
| 45657553 | endonuclease | 308 |
| 45657559 | hypothetical protein LIC11693 | 166 |
| 45657579 | motility protein B | 266 |
| 45657583 | nucleotide excision repair subunit A UvrABC | 948 |
| 45657584 | protease IV | 579 |
| 45657605 | hypothetical protein LIC11739 | 1934 |
| 45657606 | hypothetical protein LIC11740 | 444 |
| 45657607 | hypothetical protein LIC11741 | 461 |

| | | |
|---|---|---|
| 45657615 | hypothetical protein LIC11749 | 593 |
| 45657616 | hypothetical protein LIC11750 | 215 |
| 45657634 | 3-isopropylmalate dehydrogenase | 358 |
| 45657636 | hypothetical protein LIC11770 | 181 |
| 45657640 | DNA mismatch repair protein | 848 |
| 45657642 | hypothetical protein LIC11776 | 248 |
| 45657648 | hypothetical protein LIC11782 | 348 |
| 45657654 | alpha/beta hydrolase | 277 |
| 45657657 | ATP-dependent DNA helicase | 569 |
| 45657666 | hypothetical protein LIC11800 | 318 |
| 45657674 | glutaredoxin-related protein | 107 |
| 45657676 | hypothetical protein LIC11811 | 343 |
| 45657686 | isopropylmalate isomerase small subunit | 206 |
| 45657694 | bifunctional GMP synthase/glutamine amidotransferase protein | 603 |
| 45657707 | hypothetical protein LIC11844 | 315 |
| 45657708 | hypothetical protein LIC11845 | 310 |
| 45657711 | hypothetical protein LIC11848 | 276 |
| 45657715 | O-acetylhomoserine (thiol) lyase | 434 |
| 45657718 | d-alanine-d-alanine ligase | 351 |
| 45657722 | hypothetical protein LIC11859 | 251 |
| 45657726 | UDP-N-acetylmuramate-alanine ligase | 490 |

| | | |
|---|---|---|
| 45657727 | UDP-N-acetylglucosamine:LPS N-acetylglucosamine transferase | 358 |
| 45657730 | UDP-N-acetylmuramoylalanyl-D-glutamate--2,6-diaminopimelate ligase | 506 |
| 45657738 | putative hydrolase | 412 |
| 45657747 | hypothetical protein LIC11884 | 432 |
| 45657760 | pyruvate dehydrogenase alpha2 subunit protein | 327 |
| 45657769 | hypothetical protein LIC11909 | 156 |
| 45657774 | udp-n-acetyl-d-mannosamine transferase | 275 |
| 45657775 | thiamine phosphate pyrophosphorylase | 214 |
| 45657779 | IMP dehydrogenase | 508 |
| 45657780 | hypothetical protein LIC11920 | 389 |
| 45657805 | hypothetical protein LIC11946 | 223 |
| 45657810 | ATP-dependent Clp protease-like, proteolytic subunit | 197 |
| 45657814 | hypothetical protein LIC11955 | 186 |
| 45657817 | ARM repeat-containing protein | 402 |
| 45657824 | hypothetical protein LIC11970 | 623 |
| 45657825 | hypothetical protein LIC11971 | 190 |
| 45657845 | D-3-phosphoglycerate dehydrogenase | 226 |
| 45657854 | succinate dehydrogenase cytochrome B subunit | 226 |
| 45657868 | hypothetical protein LIC12016 | 135 |
| 45657878 | methylenetetrahydrofolate dehydrogenase | 284 |
| 45657879 | hypothetical protein LIC12027 | 321 |

| | | |
|---|---|---|
| 45657889 | hypothetical protein LIC12040 | 146 |
| 45657912 | hypothetical protein LIC12064 | 332 |
| 45657918 | hypothetical protein LIC12070 | 310 |
| 45657919 | hypothetical protein LIC12071 | 387 |
| 45657923 | hypothetical protein LIC12075 | 282 |
| 45657926 | hypothetical protein LIC12078 | 525 |
| 45657932 | hypothetical protein LIC12084 | 357 |
| 45657938 | glyceraldehyde-3-phosphate dehydrogenase | 335 |
| 45657949 | hypothetical protein LIC12101 | 418 |
| 45657960 | single-stranded DNA binding protein | 150 |
| 45657972 | dTDP-glucose 4,6-dehydratase | 349 |
| 45657976 | glycosyl transferase | 273 |
| 45657979 | hypothetical protein LIC12131 | 139 |
| 45657981 | hypothetical protein LIC12133 | 297 |
| 45658012 | hypothetical protein LIC12163 | 602 |
| 45658014 | putative molybdenum cofactor biosynthesis protein | 325 |
| 45658018 | sialic acid synthase | 753 |
| 45658021 | putative UDP-N-acetylglucosamine-2-epimerase | 387 |
| 45658026 | CMP-N-acetlyneuraminic acid synthetase | 228 |
| 45658028 | sugar transferase | 315 |
| 45658042 | hypothetical protein LIC12193 | 264 |
| 45658043 | transketolase beta subunit | 310 |
| 45658045 | hypothetical protein LIC12196 | 544 |

| | | |
|---|---|---|
| 45658053 | FAD-dependent oxidoreductase family protein | 365 |
| 45658054 | GDP-l-fucose synthetase | 314 |
| 45658060 | Hsp15 | 130 |
| 45658070 | cation:proton antiporter | 750 |
| 45658094 | host factor-1 | 85 |
| 45658096 | hypothetical protein LIC12247 | 171 |
| 45658112 | hypothetical protein LIC12263 | 331 |
| 45658117 | putative glycolate oxidase | 760 |
| 45658124 | hypothetical protein LIC12276 | 222 |
| 45658142 | udp-glucose dehydrogenase | 436 |
| 45658153 | LexA family transcriptional repressor | 203 |
| 45658160 | glucokinase | 298 |
| 45658172 | serine/threonine kinase protein | 1780 |
| 45658174 | hypothetical protein LIC12326 | 123 |
| 45658176 | putative lactoylglutathione lyase | 152 |
| 45658177 | hypothetical protein LIC12331 | 281 |
| 45658179 | hypothetical protein LIC12333 | 340 |
| 45658187 | hypothetical protein LIC12341 | 218 |
| 45658189 | hypothetical protein LIC12343 | 177 |
| 45658199 | hypothetical protein LIC12353 | 306 |
| 45658203 | elongation factor EF-G | 606 |
| 45658214 | hypothetical protein LIC12368 | 398 |
| 45658217 | hypothetical protein LIC12371 | 150 |
| 45658223 | capsule biosynthesis protein | 342 |

| | | |
|---|---|---|
| 45658242 | hypothetical protein LIC12396 | 239 |
| 45658243 | hypothetical protein LIC12397 | 614 |
| 45658248 | phosphoribosylformylglycinamide synthetase | 745 |
| 45658253 | putative glutamine synthetase protein | 473 |
| 45658266 | hypothetical protein LIC12421 | 1010 |
| 45658267 | aspartate aminotransferase a | 366 |
| 45658278 | phosphoglycerol transferase | 662 |
| 45658287 | putative glycerol-3-phosphate acyltransferase PlsX | 335 |
| 45658289 | ATP phosphoribosyltransferase catalytic subunit | 206 |
| 45658290 | hypothetical protein LIC12446 | 247 |
| 45658294 | chorismate mutase and prephenate dehydratase | 368 |
| 45658295 | hypothetical protein LIC12452 | 187 |
| 45658298 | protein-glutamate methylesterase | 357 |
| 45658302 | hypothetical protein LIC12460 | 212 |
| 45658318 | dihydrolipoamide succinyltransferase | 421 |
| 45658319 | Pbp1A | 812 |
| 45658330 | hypothetical protein LIC12488 | 140 |
| 45658331 | hypothetical protein LIC12489 | 187 |
| 45658341 | hypothetical protein LIC12499 | 523 |
| 45658342 | chemoreceptor (methyl-accepting chemotaxis) transmembrane protein | 691 |
| 45658343 | hypothetical protein LIC12501 | 313 |

| | | |
|---|---|---|
| 45658357 | cystine-binding periplasmic protein precursor | 330 |
| 45658358 | acetoacetyl-CoA synthetase | 660 |
| 45658371 | sulfate ABC transporter periplasmic sulphate-binding protein precursor | 335 |
| 45658376 | GTP cyclohydrolase 2 | 401 |
| 45658381 | hypothetical protein LIC12539 | 248 |
| 45658382 | hypothetical protein LIC12540 | 108 |
| 45658383 | anthranilate phosphoribosyltransferase | 336 |
| 45658385 | hypothetical protein LIC12543 | 437 |
| 45658386 | DNA binding protein | 371 |
| 45658387 | outer membrane lipoprotein carrier protein | 257 |
| 45658390 | hypothetical protein LIC12548 | 80 |
| 45658401 | deoxyuridine 5'triphosphate nucleotidohydrolase | 145 |
| 45658402 | hypothetical protein LIC12561 | 316 |
| 45658406 | adenylosuccinate synthetase | 425 |
| 45658407 | hypothetical protein LIC12568 | 278 |
| 45658411 | succinyl-CoA synthetase beta subunit | 390 |
| 45658412 | succinyl-CoA synthetase alpha subunit | 291 |
| 45658414 | hypothetical protein LIC12576 | 477 |
| 45658419 | hypothetical protein LIC12581 | 160 |
| 45658452 | hypothetical protein LIC12615 | 356 |
| 45658457 | membrane carboxypeptidase | 827 |

| | | |
|---|---|---|
| 45658458 | hypothetical protein LIC12621 | 136 |
| 45658472 | hypothetical protein LIC12635 | 387 |
| 45658521 | exodeoxyribonuclease v alpha chain | 617 |
| 45658527 | hypothetical protein LIC12692 | 716 |
| 45658529 | glutamate synthase (NADPH) alpha chain precursor | 1498 |
| 45658530 | hypothetical protein LIC12695 | 441 |
| 45658536 | polynucleotide phosphorylase/polyade nylase | 698 |
| 45658543 | hypothetical protein LIC12708 | 392 |
| 45658552 | putative 4-hydroxybenzoate transporter transmembrane protein | 465 |
| 45658553 | putative enolase-phosphatase E-1 | 234 |
| 45658564 | hypothetical protein LIC12730 | 676 |
| 45658565 | hypothetical protein LIC12731 | 665 |
| 45658571 | site-specific modification DNA-methyltransferase | 543 |
| 45658577 | NADH dehydrogenase I subunit C | 178 |
| 45658578 | NADH dehydrogenase I D subunit | 405 |
| 45658579 | NADH dehydrogenase I E subunit | 161 |
| 45658580 | NADH dehydrogenase I F subunit | 443 |
| 45658581 | NADH dehydrogenase I H subunit | 346 |
| 45658589 | hypothetical protein LIC12755 | 156 |

| | | |
|---|---|---|
| 45658590 | DshA | 350 |
| 45658603 | putative nicotinate-nucleotide adenylyltransferase | 199 |
| 45658613 | hydroxymethylglutaryl-CoA lyase | 298 |
| 45658624 | hypothetical protein LIC12793 | 254 |
| 45658627 | hypothetical protein LIC12796 | 286 |
| 45658632 | hypothetical protein LIC12801 | 303 |
| 45658636 | hypothetical protein LIC12805 | 335 |
| 45658643 | serine protease MucD precursor | 388 |
| 45658651 | hypothetical protein LIC12821 | 146 |
| 45658652 | hypothetical protein LIC12822 | 255 |
| 45658661 | SAM dependent methyltransferase | 315 |
| 45658664 | hypothetical protein LIC12834 | 347 |
| 45658676 | DNA-directed RNA polymerase subunit alpha | 325 |
| 45658685 | 50S ribosomal protein L30 | 59 |
| 45658708 | hypothetical protein LIC12878 | 183 |
| 45658716 | hypothetical protein LIC12886 | 249 |
| 45658752 | hypothetical protein LIC12923 | 514 |
| 45658756 | beta-lactamase related protein | 321 |
| 45658759 | chemotaxis motility protein A | 252 |
| 45658764 | hypothetical protein LIC12936 | 237 |
| 45658770 | hypothetical protein LIC12942 | 222 |
| 45658793 | LipL41 | 355 |
| 45658797 | hypothetical protein LIC12970 | 409 |
| 45658801 | hypothetical protein LIC12974 | 187 |

| | | |
|---|---|---|
| 45658807 | hypothetical protein LIC12980 | 151 |
| 45658819 | sulfate permease | 750 |
| 45658822 | hypothetical protein LIC12995 | 335 |
| 45658842 | hypothetical protein LIC13015 | 346 |
| 45658844 | acriflavine resistance | 1103 |
| 45658848 | hypothetical protein LIC13021 | 857 |
| 45658849 | hypothetical protein LIC13022 | 391 |
| 45658853 | ThiJ/PfpI family protein | 234 |
| 45658872 | UDP glucosamine N-acyltransferase | 340 |
| 45658876 | hypothetical protein LIC13050 | 366 |
| 45658897 | hypothetical protein LIC13071 | 181 |
| 45658900 | acriflavin resistance | 1083 |
| 45658915 | hypothetical protein LIC13089 | 554 |
| 45658920 | survival protein | 250 |
| 45658931 | sugar-phosphate nucleotidyl transferase | 242 |
| 45658932 | hypothetical protein LIC13109 | 331 |
| 45658944 | hypothetical protein LIC13121 | 290 |
| 45658946 | hypothetical protein LIC13123 | 291 |
| 45658947 | hypothetical protein LIC13125 | 188 |
| 45658950 | hypothetical protein LIC13128 | 183 |
| 45658953 | hypothetical protein LIC13131 | 181 |
| 45658964 | phosphopantothenoylcysteine decarboxylase | 180 |
| 45658966 | hypothetical protein LIC13144 | 861 |
| 45658977 | hypothetical protein LIC13155 | 115 |
| 45658984 | phospho-2-dehydro-3-deoxyheptonate aldolase chorismate mutase | 380 |

| | | |
|---|---|---|
| 45658988 | hypothetical protein LIC13166 | 306 |
| 45658994 | hypothetical protein LIC13172 | 419 |
| 45658997 | potassium uptake system protein | 236 |
| 45659002 | (di)nucleoside polyphosphate hydrolase | 162 |
| 45659005 | methyl-accepting chemotaxis-like | 127 |
| 45659034 | hypothetical protein LIC13212 | 278 |
| 45659036 | putative cytochrome oxidase subunit | 194 |
| 45659041 | phenylalanyl-tRNA synthetase beta chain | 801 |
| 45659057 | ankyrin-like protein | 338 |
| 45659058 | hypothetical protein LIC13237 | 185 |
| 45659078 | NADH oxidoreductase | 340 |
| 45659083 | glutamine phosphoribosylpyrophosphate amidotransferase | 490 |
| 45659085 | MutT nudix family protein | 223 |
| 45659092 | bifunctional ornithine acetyltransferase/N-acetylglutamate synthase protein | 385 |
| 45659096 | hypothetical protein LIC13275 | 374 |
| 45659114 | hypothetical protein LIC13293 | 659 |
| 45659129 | phenylalanyl-tRNA synthetase alpha chain | 341 |
| 45659133 | hypothetical protein LIC13314 | 675 |
| 45659144 | phosphopantetheine adenylyltransferase | 160 |
| 45659145 | nucleoside diphosphate kinase | 137 |
| 45659164 | glutamyl-tRNA synthetase | 521 |
| 45659179 | hypothetical protein LIC13360 | 611 |

| | | |
|---|---|---|
| 45659182 | hypothetical protein LIC13363 | 258 |
| 45659186 | sulfite reductase subunit beta | 561 |
| 45659216 | 5'-methylthioadenosine phosphorylase | 287 |
| 45659224 | hypothetical protein LIC13407 | 162 |
| 45659234 | hypothetical protein LIC13417 | 283 |
| 45659235 | hypothetical protein LIC13418 | 341 |
| 45659240 | hydrogenase subunit | 466 |
| 45659253 | hypothetical protein LIC13436 | 224 |
| 45659255 | hypoxanthine-guanine-xanthine phosphoribosyltransferase | 183 |
| 45659289 | DNA polymerase III gamma subunit | 479 |
| 45659297 | hypothetical protein LIC13482 | 339 |
| 45659301 | hypothetical protein LIC13488 | 291 |
| 45659303 | glucose inhibited division protein B | 254 |
| 45659305 | hypothetical protein LIC13494 | 276 |
| 45659308 | DNA polymerase III | 310 |
| 161621772 | 50S ribosomal protein L14 | 130 |
| 161621780 | valine--pyruvate transaminase | 417 |
| 229259640 | F0F1 ATP synthase subunit A | 350 |
| 386072505 | hypothetical protein LIF_A0017 | 298 |
| 386072508 | putative translation factor Sua5 | 203 |
| 386072515 | hypothetical protein LIF_A0027 | 234 |
| 386072526 | hypothetical protein LIF_A0038 | 502 |
| 386072534 | NAD(P)(+) transhydrogenase alpha subunit | 389 |

| | | |
|---|---|---|
| 386072551 | hypothetical protein LIF_A0063 | 156 |
| 386072581 | hypothetical protein LIF_A0096 | 118 |
| 386072596 | imidazoleglycerol-phosphate dehydratase or histidinol-phosphatase | 206 |
| 386072599 | hypothetical protein LIF_A0115 | 404 |
| 386072607 | hypothetical protein LIF_A0123 | 433 |
| 386072608 | LipL45-like lipoprotein | 309 |
| 386072610 | hypothetical protein LIF_A0126 | 1197 |
| 386072623 | hypothetical protein LIF_A0139 | 489 |
| 386072640 | hypothetical protein LIF_A0158 | 692 |
| 386072668 | hypothetical protein LIF_A0189 | 510 |
| 386072704 | hypothetical protein LIF_A0225 | 242 |
| 386072708 | hypothetical protein LIF_A0229 | 105 |
| 386072711 | hypothetical protein LIF_A0232 | 337 |
| 386072712 | permease component of lipoprotein releasing system | 457 |
| 386072715 | hypothetical protein LIF_A0235a | 94 |
| 386072720 | hypothetical protein LIF_A0240 | 231 |
| 386072755 | hypothetical protein LIF_A0276 | 142 |
| 386072764 | NAD(P)(+) transhydrogenase beta component | 466 |
| 386072783 | glycine cleavage T protein | 396 |
| 386072786 | hypothetical protein LIF_A0308 | 553 |
| 386072790 | hypothetical protein LIF_A0312 | 577 |
| 386072799 | hypothetical protein LIF_A0321 | 511 |

| | | |
|---|---|---|
| 386072822 | ATP-binding protein of an ABC transporter complex | 304 |
| 386072860 | hypothetical protein LIF_A0383 | 56 |
| 386072879 | hypothetical protein LIF_A0402 | 157 |
| 386072898 | hypothetical protein LIF_A0422 | 366 |
| 386072924 | hypothetical protein LIF_A0449 | 841 |
| 386072930 | putative periplasmic solute-binding protein | 338 |
| 386072978 | protein FtsA | 409 |
| 386073010 | hypothetical protein LIF_A0534 | 232 |
| 386073060 | hypothetical protein LIF_A0586 | 249 |
| 386073102 | RNA polymerase alpha subunit | 325 |
| 386073114 | putative Zn-dependent hydrolase | 347 |
| 386073126 | putative protein involved in cell shape determination | 146 |
| 386073152 | putative thioesterase | 286 |
| 386073161 | Indole-3-glycerol phosphate synthase | 252 |
| 386073191 | hypothetical protein LIF_A0718 | 156 |
| 386073215 | hypothetical protein LIF_A0742 | 665 |
| 386073237 | hypothetical protein LIF_A0764 | 392 |
| 386073249 | hypothetical protein LIF_A0776 | 441 |
| 386073252 | hypothetical protein LIF_A0779 | 714 |
| 386073324 | hypothetical protein LIF_A0851 | 136 |
| 386073355 | hypothetical protein LIF_A0883 | 160 |
| 386073362 | hypothetical protein LIF_A0890 | 477 |
| 386073370 | hypothetical protein LIF_A0898 | 278 |
| 386073388 | hypothetical protein LIF_A0919 | 80 |

| | | |
|---|---|---|
| 386073393 | hypothetical protein LIF_A0924 | 371 |
| 386073405 | substrate-binding protein of ABC-type sulfate transport system | 335 |
| 386073419 | substrate binding protein of an ABC transporter complex | 330 |
| 386073430 | signal transduction protein containing REC,GGDEF and EAL domains | 569 |
| 386073434 | hypothetical protein LIF_A0965 | 313 |
| 386073478 | hypothetical protein LIF_A1010 | 213 |
| 386073512 | hypothetical protein LIF_A1044 | 1010 |
| 386073534 | hypothetical protein LIF_A1067 | 612 |
| 386073535 | hypothetical protein LIF_A1068 | 239 |
| 386073549 | signal receiver component of two-component system | 151 |
| 386073553 | capsule biosynthesis protein CapA (poly-gamma-glutamate biosynthesis protein) | 342 |
| 386073559 | hypothetical protein LIF_A1093 | 150 |
| 386073581 | 50S ribosomal protein L11 methylase | 300 |
| 386073586 | hypothetical protein LIF_A1120 | 218 |
| 386073593 | glycine hydroxymethyltransferase | 415 |
| 386073595 | hypothetical protein LIF_A1129 | 334 |
| 386073600 | hypothetical protein LIF_A1134 | 125 |
| 386073602 | signal transduction protein containing GAF and PP2C domains | 930 |
| 386073667 | hypothetical protein LIF_A1202 | 331 |

| | | |
|---|---|---|
| 386073694 | putative signal transduction protein containing CBS domains | 146 |
| 386073739 | hypothetical protein LIF_A1274 | 264 |
| 386073771 | putative carbamoyl transferase | 602 |
| 386073794 | FnlA | 344 |
| 386073836 | CinA | 418 |
| 386073856 | hypothetical protein LIF_A1390 | 357 |
| 386073863 | hypothetical protein LIF_A1397 | 282 |
| 386073957 | hypothetical protein LIF_A1488 | 146 |
| 386073968 | bifunctional folate pathway enzyme | 285 |
| 386074021 | hypothetical protein LIF_A1555 | 190 |
| 386074033 | hypothetical protein LIF_A1568 | 402 |
| 386074036 | hypothetical protein LIF_A1571 | 164 |
| 386074072 | hypothetical protein LIF_A1607 | 230 |
| 386074080 | hypothetical protein LIF_A1614 | 169 |
| 386074100 | hypothetical protein LIF_A1636 | 412 |
| 386074101 | hypothetical protein LIF_A1637 | 420 |
| 386074136 | hypothetical protein LIF_A1672 | 276 |
| 386074232 | hypothetical protein LIF_A1771 | 198 |
| 386074241 | putative Zn-dependent protease | 461 |
| 386074243 | hypothetical protein LIF_A1782 | 1934 |
| 386074273 | sensor histidine kinase and response regulator of a two component complex | 832 |
| 386074280 | Yqey-like protein | 148 |
| 386074323 | PurH | 511 |
| 386074336 | hypothetical protein LIF_A1875 | 436 |
| 386074358 | hypothetical protein LIF_A1897 | 234 |

| | | |
|---|---|---|
| 386074382 | hypothetical protein LIF_A1921 | 519 |
| 386074384 | hypothetical protein LIF_A1923 | 190 |
| 386074418 | hypothetical protein LIF_A1958 | 331 |
| 386074421 | hypothetical protein LIF_A1961 | 283 |
| 386074457 | hypothetical protein LIF_A1997 | 102 |
| 386074474 | hypothetical protein LIF_A2016 | 64 |
| 386074484 | hypothetical protein LIF_A2026 | 444 |
| 386074485 | inhibitor of MCP methylation | 154 |
| 386074490 | putative polymerase | 572 |
| 386074511 | amidase | 487 |
| 386074516 | hypothetical protein LIF_A2057 | 241 |
| 386074526 | diguanylate cyclase | 330 |
| 386074529 | hypothetical protein LIF_A2070 | 143 |
| 386074561 | hypothetical protein LIF_A2103 | 369 |
| 386074563 | hypothetical protein LIF_A2105 | 89 |
| 386074564 | hypothetical protein LIF_A2106 | 667 |
| 386074657 | putative N6-adenine-specific DNA methylase | 416 |
| 386074682 | hypothetical protein LIF_A2227 | 119 |
| 386074688 | putative acetyl-CoA carboxylase/pyruvate carboxylase | 166 |
| 386074691 | hypothetical protein LIF_A2236 | 369 |
| 386074706 | actin-like ATPase | 340 |
| 386074719 | ATP synthase subunit gamma | 286 |
| 386074721 | ATP synthase F1 subunit delta | 186 |
| 386074750 | hypothetical protein LIF_A2300 | 134 |
| 386074762 | hypothetical protein LIF_A2313 | 155 |

| | | |
|---|---|---|
| 386074807 | Holliday junction ATP-dependent DNA helicase RuvA | 212 |
| 386074808 | flavin reductase-related protein | 159 |
| 386074881 | hypothetical protein LIF_A2434 | 107 |
| 386074885 | ssDNA-specific exonuclease RecJ | 643 |
| 386074902 | leucine-rich repeat containing protein | 685 |
| 386074907 | hypothetical protein LIF_A2460 | 390 |
| 386074938 | OppA | 599 |
| 386074941 | hypothetical protein LIF_A2494 | 245 |
| 386074967 | hypothetical protein LIF_A2522 | 322 |
| 386074997 | serine/threonine phosphatase containing GAF domains | 588 |
| 386075055 | hypothetical protein LIF_A2615 | 407 |
| 386075057 | hypothetical protein LIF_A2617 | 183 |
| 386075073 | adenylate cyclase-like protein | 770 |
| 386075100 | leucine-rich repeat protein | 377 |
| 386075127 | hypothetical protein LIF_A2689 | 191 |
| 386075144 | transcript cleavage factor/hypothetical protein | 926 |
| 386075163 | hypothetical protein LIF_A2728 | 129 |
| 386075168 | hypothetical protein LIF_A2733 | 209 |
| 386075201 | hypothetical protein LIF_A2768 | 318 |
| 386075215 | putative periplasmic lipoprotein | 404 |
| 386075230 | putative esterase | 235 |
| 386075237 | hypothetical protein LIF_A2804 | 740 |
| 386075253 | phosphoribosylaminoimidazolesuccinocarboxamide synthase | 285 |

| | | |
|---|---|---|
| 386075254 | serine/threonine phosphatase containing signal receiver domain REC | 377 |
| 386075260 | hypothetical protein LIF_A2828 | 229 |
| 386075268 | hypothetical protein LIF_A2836 | 162 |
| 386075269 | hypothetical protein LIF_A2837 | 158 |
| 386075291 | hypothetical protein LIF_A2858 | 320 |
| 386075301 | ATP-binding component of an ABC transporter complex | 255 |
| 386075330 | hypothetical protein LIF_A2897 | 150 |
| 386075355 | HEAT repeat containing protein | 486 |
| 386075400 | hypothetical protein LIF_A2966 | 674 |
| 386075405 | GrpE | 212 |
| 386075406 | DnaK protein | 646 |
| 386075409 | putative transcriptional regulator | 182 |
| 386075412 | ATPase component of ABC-type antimicrobial peptide transport system | 260 |
| 386075437 | hypothetical protein LIF_A3003 | 357 |
| 386075440 | hypothetical protein LIF_A3006 | 330 |
| 386075441 | hypothetical protein LIF_A3007 | 194 |
| 386075452 | peptidyl-tRNA hydrolase involved in peptide chain release | 367 |
| 386075477 | 3-methylcrotonoyl-CoA carboxylase subunit beta | 536 |
| 386075485 | hypothetical protein LIF_A3053 | 129 |
| 386075486 | hypothetical protein LIF_A3054 | 157 |
| 386075541 | histidine kinase and response regulator hybrid protein | 507 |

| | | |
|---|---|---|
| 386075551 | hypothetical protein LIF_A3122 | 290 |
| 386075552 | hypothetical protein LIF_A3123 | 186 |
| 386075553 | hypothetical protein LIF_A3124 | 291 |
| 386075557 | hypothetical protein LIF_A3129 | 169 |
| 386075560 | hypothetical protein LIF_A3132 | 181 |
| 386075573 | hypothetical protein LIF_A3145 | 861 |
| 386075590 | 3-deoxy-D-arabinoheptulosonate-7-phosphate synthase | 373 |
| 386075594 | hypothetical protein LIF_A3166 | 303 |
| 386075610 | hypothetical protein LIF_A3182 | 127 |
| 386075640 | hypothetical protein LIF_A3211 | 264 |
| 386075661 | lysine decarboxylase-related protein | 185 |
| 386075694 | hypothetical protein LIF_A3266 | 148 |
| 386075702 | hypothetical protein LIF_A3274 | 374 |
| 386075706 | putative acetyl-CoA acetyltransferase | 507 |
| 386075735 | phenylalanine-tRNA synthetase alpha subunit | 341 |
| 386075739 | hypothetical protein LIF_A3311 | 672 |
| 386075765 | hypothetical protein LIF_A3337 | 365 |
| 386075826 | hypothetical protein LIF_A3398 | 162 |
| 386075854 | hypothetical protein LIF_A3426 | 224 |
| 386075903 | hypothetical protein LIF_A3479 | 276 |
| 386075905 | putative branched-chain amino acid aminotransferase | 307 |
| 386075906 | DNA polymerase III subunits gamma and tau | 310 |
| 386072548 | hypothetical protein LIF_A0060 | 1061 |

| | | |
|---|---|---|
| 386072550 | hypothetical protein LIF_A0062 | 987 |
| 386072591 | hypothetical protein LIF_A0106 | 340 |
| 386072601 | hypothetical protein LIF_A0117 | 607 |
| 386072630 | hypothetical protein LIF_A0146 | 457 |
| 386072713 | lipoprotein releasing system, LolD ATPase component | 225 |
| 386072742 | regulatory-related cAMP-binding protein | 351 |
| 386072775 | hypothetical protein LIF_A0297 | 349 |
| 386072798 | hypothetical protein LIF_A0320 | 378 |
| 386072809 | hypothetical protein LIF_A0331 | 304 |
| 386072851 | hypothetical protein LIF_A0374 | 181 |
| 386072855 | hypothetical protein LIF_A0378 | 672 |
| 386072872 | hypothetical protein LIF_A0395 | 268 |
| 386073121 | hypothetical protein LIF_A0647 | 437 |
| 386073144 | hypothetical protein LIF_A0671 | 334 |
| 386073148 | alpha/beta hydrolase-related protein | 303 |
| 386073190 | hypothetical protein LIF_A0717 | 350 |
| 386073323 | hypothetical protein LIF_A0850 | 459 |
| 386073397 | Preprotein translocase YajC subunit | 108 |
| 386073406 | sulfate ABC transport system permease protein | 276 |
| 386073447 | hypothetical protein LIF_A0978 | 195 |
| 386073492 | hypothetical protein LIF_A1024 | 247 |
| 386073501 | phosphoglycerol transferase-related protein | 662 |
| 386073676 | hypothetical protein LIF_A1211 | 527 |

| | | |
|---|---|---|
| 386073807 | glycosyl transferase family protein | 273 |
| 386073867 | hypothetical protein LIF_A1401 | 387 |
| 386074013 | hypothetical protein LIF_A1547 | 269 |
| 386074044 | hypothetical protein LIF_A1579 | 203 |
| 386074065 | hypothetical protein LIF_A1600 | 540 |
| 386074123 | hypothetical protein LIF_A1659 | 182 |
| 386074146 | hypothetical protein LIF_A1682 | 325 |
| 386074289 | hypothetical protein LIF_A1828 | 166 |
| 386074298 | hypothetical protein LIF_A1837 | 356 |
| 386074339 | hypothetical protein LIF_A1878 | 226 |
| 386074340 | hypothetical protein LIF_A1879 | 224 |
| 386074472 | hypothetical protein LIF_A2014 | 305 |
| 386074534 | LipL45-related protein | 705 |
| 386074571 | hypothetical protein LIF_A2113 | 353 |
| 386074696 | hypothetical protein LIF_A2242 | 609 |
| 386074736 | hypothetical protein LIF_A2285 | 368 |
| 386074774 | MhpC-related protein | 335 |
| 386074776 | hypothetical protein LIF_A2328 | 173 |
| 386075060 | hypothetical protein LIF_A2620 | 168 |
| 386075089 | hypothetical protein LIF_A2650 | 351 |
| 386075267 | hypothetical protein LIF_A2835 | 130 |
| 386075270 | hypothetical protein LIF_A2838 | 182 |
| 386075304 | hypothetical protein LIF_A2871 | 139 |
| 386075353 | ATPase and permease components of ABC-type multidrug transport system | 606 |
| 386075448 | hypothetical protein LIF_A3015 | 142 |

| | | |
|---|---|---|
| 386075523 | hypothetical protein LIF_A3092 | 554 |
| 386075836 | hypothetical protein LIF_A3408 | 650 |
| Total Conserved Proteins | | |

*terrogans* serovar Canicola

| Mean No. of peptides | Mean % Coverage |
|---|---|
| 24.00 | 30.67 |
| 31.67 | 35.23 |
| 18.67 | 19.17 |
| 4.33 | 14.50 |
| 10.33 | 27.50 |
| 4.33 | 19.40 |
| 9.00 | 8.07 |
| 9.33 | 27.87 |
| 3.67 | 5.13 |
| 3.67 | 6.63 |
| 2.33 | 11.50 |
| 14.33 | 26.90 |
| 15.33 | 31.67 |
| 19.00 | 17.03 |
| 2.00 | 1.60 |
| 5.33 | 34.43 |
| 15.67 | 29.27 |
| 14.33 | 16.33 |
| 11.00 | 17.47 |
| 4.67 | 21.03 |
| 6.67 | 29.13 |
| 2.67 | 7.60 |

| | |
|---|---|
| 10.33 | 25.00 |
| 2.67 | 9.60 |
| 12.67 | 14.57 |
| 10.33 | 18.43 |
| 14.67 | 17.20 |
| 11.33 | 26.97 |
| 12.00 | 19.43 |
| 10.00 | 27.83 |
| 5.33 | 10.33 |
| 4.00 | 6.93 |
| 12.33 | 27.93 |
| 3.33 | 5.33 |
| 21.00 | 36.10 |
| 2.33 | 6.93 |
| 9.00 | 30.37 |
| 4.67 | 6.87 |
| 20.00 | 17.07 |
| 77.67 | 36.03 |
| 11.00 | 18.00 |
| 12.00 | 25.13 |
| 2.33 | 7.97 |
| 3.67 | 13.80 |
| 4.67 | 11.70 |

| | |
|---|---|
| 11.33 | 29.40 |
| 3.33 | 14.20 |
| 4.67 | 16.60 |
| 98.00 | 43.23 |
| 4.33 | 9.60 |
| 12.00 | 10.13 |
| 46.33 | 29.20 |
| 5.33 | 34.43 |
| 29.67 | 22.17 |
| 3.33 | 16.87 |
| 2.67 | 10.47 |
| 5.67 | 14.73 |
| 82.00 | 45.20 |
| 22.33 | 29.23 |
| 19.00 | 20.77 |
| 2.33 | 18.60 |
| 18.67 | 23.60 |
| 11.00 | 39.20 |
| 27.00 | 41.00 |
| 41.33 | 44.10 |
| 5.00 | 11.50 |
| 39.33 | 48.57 |
| 9.67 | 22.03 |

| | |
|---|---|
| 5.67 | 11.87 |
| 5.33 | 8.70 |
| 7.00 | 9.60 |
| 2.67 | 5.50 |
| 12.33 | 9.70 |
| 3.33 | 9.13 |
| 16.00 | 27.37 |
| 5.67 | 13.07 |
| 4.33 | 10.30 |
| 4.67 | 11.20 |
| 5.00 | 15.13 |
| 13.00 | 17.63 |
| 7.00 | 16.93 |
| 2.33 | 5.57 |
| 27.33 | 28.97 |
| 8.00 | 11.10 |
| 10.67 | 29.67 |
| 2.67 | 9.73 |
| 31.67 | 20.40 |
| 3.33 | 19.60 |
| 240.33 | 65.30 |
| 7.00 | 26.40 |
| 3.67 | 10.60 |
| 10.33 | 20.97 |

| | |
|---|---|
| 2.67 | 11.50 |
| 18.00 | 37.93 |
| 5.33 | 34.20 |
| 7.33 | 24.87 |
| 5.00 | 29.17 |
| 14.67 | 46.47 |
| 2.67 | 19.60 |
| 7.33 | 23.77 |
| 4.67 | 15.23 |
| 2.00 | 10.60 |
| 14.00 | 35.17 |
| 19.67 | 58.77 |
| 6.67 | 30.93 |
| 15.67 | 41.53 |
| 61.33 | 56.60 |
| 12.00 | 20.60 |
| 54.33 | 40.23 |
| 17.33 | 39.33 |
| 4.00 | 14.77 |
| 2.00 | 3.40 |
| 16.00 | 38.07 |
| 2.00 | 7.60 |
| 34.67 | 34.57 |
| 2.67 | 11.07 |
| 2.67 | 9.37 |

| | |
|---|---|
| 6.67 | 17.57 |
| 2.67 | 6.33 |
| 5.33 | 39.67 |
| 3.00 | 7.37 |
| 5.33 | 17.40 |
| 5.67 | 27.87 |
| 3.00 | 38.43 |
| 6.33 | 15.13 |
| 8.00 | 21.57 |
| 30.00 | 39.07 |
| 2.67 | 20.03 |
| 17.33 | 31.53 |
| 2.67 | 4.40 |
| 5.00 | 28.17 |
| 5.33 | 12.20 |
| 3.00 | 10.93 |
| 7.33 | 40.40 |
| 17.33 | 49.10 |
| 3.67 | 29.10 |
| 8.33 | 11.07 |
| 3.67 | 20.43 |
| 15.67 | 20.40 |
| 13.33 | 37.83 |
| 4.00 | 15.77 |
| 5.67 | 11.07 |
| 14.00 | 24.30 |
| 51.67 | 35.07 |

| | |
|---|---|
| 4.67 | 8.87 |
| 71.33 | 32.47 |
| 8.67 | 19.20 |
| 4.33 | 20.77 |
| 52.33 | 28.93 |
| 17.00 | 27.77 |
| 2.00 | 14.70 |
| 9.00 | 4.40 |
| 3.33 | 37.47 |
| 3.00 | 5.20 |
| 38.67 | 48.17 |
| 34.00 | 37.53 |
| 7.33 | 16.63 |
| 9.67 | 11.97 |
| 7.67 | 21.17 |
| 28.00 | 22.50 |
| 7.00 | 33.50 |
| 6.33 | 33.73 |
| 3.00 | 12.40 |
| 2.67 | 10.70 |
| 5.67 | 11.27 |
| 18.33 | 33.97 |

| | |
|---|---|
| 2.00 | 2.33 |
| 10.00 | 22.57 |
| 23.67 | 40.53 |
| 4.67 | 5.57 |
| 4.00 | 12.40 |
| 5.67 | 19.23 |
| 2.67 | 7.63 |
| 3.67 | 10.30 |
| 17.67 | 31.73 |
| 3.00 | 30.00 |
| 24.33 | 22.73 |
| 21.00 | 31.30 |
| 47.33 | 26.53 |
| 26.33 | 22.43 |
| 3.33 | 23.03 |
| 2.00 | 16.40 |
| 6.67 | 35.57 |
| 5.33 | 12.40 |
| 5.33 | 22.10 |
| 2.67 | 2.83 |
| 3.33 | 11.93 |

| | |
|---|---|
| 11.67 | 35.23 |
| 6.67 | 28.47 |
| 3.67 | 8.70 |
| 2.00 | 5.30 |
| 6.33 | 15.27 |
| 55.00 | 38.43 |
| 17.67 | 16.83 |
| 25.00 | 17.17 |
| 3.33 | 14.33 |
| 11.00 | 26.17 |
| 8.67 | 19.73 |
| 22.33 | 29.27 |
| 14.00 | 14.10 |
| 2.00 | 5.30 |
| 12.00 | 17.83 |
| 7.00 | 18.47 |
| 35.00 | 41.17 |
| 22.67 | 41.63 |
| 5.00 | 20.90 |
| 8.33 | 5.57 |
| 5.33 | 9.13 |
| 4.33 | 12.07 |
| 25.33 | 31.90 |

| | |
|---|---|
| 3.00 | 19.93 |
| 9.00 | 12.30 |
| 10.67 | 24.10 |
| 3.33 | 7.67 |
| 4.33 | 7.33 |
| 4.00 | 11.20 |
| 4.67 | 15.07 |
| 29.67 | 34.63 |
| 6.00 | 33.80 |
| 4.67 | 13.33 |
| 6.33 | 18.90 |
| 4.67 | 19.00 |
| 3.33 | 7.97 |
| 5.33 | 12.93 |
| 2.67 | 11.07 |
| 9.67 | 10.73 |
| 17.00 | 22.00 |
| 15.00 | 26.50 |
| 70.67 | 31.93 |
| 2.67 | 10.30 |
| 2.67 | 10.07 |

| | |
|---|---|
| 6.00 | 15.90 |
| 3.00 | 8.30 |
| 20.00 | 33.57 |
| 4.00 | 24.67 |
| 6.67 | 28.47 |
| 2.33 | 3.57 |
| 4.00 | 13.80 |
| 7.00 | 53.07 |
| 5.33 | 26.17 |
| 4.00 | 29.70 |
| 11.00 | 29.27 |
| 40.33 | 29.17 |
| 6.67 | 8.47 |
| 6.33 | 18.53 |
| 50.00 | 50.73 |
| 56.00 | 39.67 |
| 16.67 | 28.43 |
| 106.67 | 59.70 |
| 7.67 | 13.50 |
| 8.00 | 26.10 |
| 9.00 | 27.13 |
| 7.33 | 13.60 |
| 9.67 | 22.03 |

| | |
|---|---|
| 8.33 | 21.67 |
| 15.00 | 16.63 |
| 6.00 | 13.13 |
| 11.00 | 18.63 |
| 6.33 | 13.23 |
| 14.67 | 22.37 |
| 5.00 | 6.83 |
| 24.67 | 72.87 |
| 43.33 | 42.80 |
| 56.00 | 34.03 |
| 8.00 | 26.80 |
| 4.33 | 7.53 |
| 3.00 | 16.33 |
| 5.00 | 7.90 |
| 14.67 | 23.37 |
| 4.00 | 21.37 |
| 24.67 | 25.37 |
| 3.00 | 14.30 |
| 20.00 | 15.37 |
| 3.33 | 12.63 |
| 5.00 | 14.80 |
| 54.00 | 47.53 |
| 9.67 | 32.37 |

| | |
|---|---|
| 13.33 | 25.27 |
| 2.67 | 8.20 |
| 2.33 | 4.53 |
| 3.33 | 10.60 |
| 2.67 | 8.77 |
| 48.33 | 38.60 |
| 23.33 | 47.67 |
| 4.67 | 12.00 |
| 9.67 | 12.67 |
| 28.00 | 30.93 |
| 10.67 | 40.27 |
| 4.33 | 16.97 |
| 12.67 | 22.40 |
| 2.00 | 3.40 |
| 2.33 | 8.60 |
| 22.00 | 22.87 |
| 48.00 | 24.30 |
| 5.67 | 17.63 |
| 85.00 | 54.47 |
| 3.00 | 11.30 |
| 3.33 | 12.53 |

| | |
|---|---|
| 4.00 | 5.37 |
| 7.67 | 52.27 |
| 31.00 | 30.43 |
| 13.33 | 23.20 |
| 11.67 | 20.70 |
| 7.33 | 23.83 |
| 46.33 | 33.67 |
| 5.33 | 9.37 |
| 11.67 | 6.20 |
| 29.67 | 33.97 |
| 7.33 | 9.80 |
| 10.33 | 11.90 |
| 10.33 | 30.10 |
| 2.33 | 8.27 |
| 4.33 | 8.97 |
| 13.00 | 19.83 |
| 4.67 | 23.00 |
| 2.67 | 12.63 |
| 17.67 | 23.57 |
| 17.33 | 28.47 |
| 3.67 | 11.03 |
| 23.67 | 15.43 |

| | |
|---|---|
| 3.00 | 10.37 |
| 12.00 | 23.00 |
| 31.00 | 24.27 |
| 6.00 | 27.90 |
| 6.33 | 12.93 |
| 3.00 | 27.13 |
| 7.00 | 13.13 |
| 13.00 | 19.60 |
| 2.00 | 6.57 |
| 8.67 | 19.07 |
| 10.67 | 37.40 |
| 5.67 | 20.30 |
| 5.33 | 17.97 |
| 4.33 | 12.03 |
| 3.67 | 7.97 |
| 3.00 | 10.00 |
| 2.00 | 5.47 |
| 4.33 | 13.50 |
| 4.33 | 5.80 |
| 51.67 | 39.30 |
| 23.67 | 28.53 |

| | |
|---|---|
| 6.00 | 27.87 |
| 11.00 | 7.70 |
| 21.67 | 24.83 |
| 2.33 | 10.80 |
| 6.33 | 18.20 |
| 11.00 | 31.60 |
| 11.33 | 21.30 |
| 6.00 | 16.00 |
| 5.67 | 4.50 |
| 6.33 | 14.50 |
| 2.33 | 3.23 |
| 27.00 | 53.17 |
| 10.00 | 21.17 |
| 5.33 | 7.60 |
| 3.33 | 22.53 |
| 14.33 | 20.83 |
| 3.67 | 23.13 |
| 5.00 | 7.27 |
| 3.00 | 6.80 |
| 3.33 | 13.87 |
| 4.67 | 5.47 |
| 54.67 | 38.67 |
| 8.00 | 21.87 |
| 49.67 | 44.93 |
| 13.67 | 47.87 |

| | |
|---|---|
| 373.33 | 66.63 |
| 4.67 | 15.43 |
| 6.00 | 16.97 |
| 6.33 | 14.50 |
| 20.67 | 33.73 |
| 3.33 | 7.43 |
| 4.67 | 15.43 |
| 8.67 | 12.37 |
| 9.33 | 17.67 |
| 8.33 | 14.57 |
| 7.00 | 14.33 |
| 7.67 | 18.93 |
| 3.00 | 6.10 |
| 20.33 | 39.33 |
| 12.33 | 33.80 |
| 7.67 | 10.93 |
| 13.33 | 35.87 |
| 3.00 | 10.13 |
| 5.67 | 10.57 |
| 18.67 | 31.90 |
| 3.33 | 19.27 |
| 5.00 | 36.20 |
| 5.33 | 14.60 |
| 2.00 | 5.07 |
| 2.67 | 9.83 |
| 2.33 | 7.00 |
| 9.67 | 17.50 |
| 4.33 | 18.07 |

| | |
|---|---|
| 8.67 | 9.33 |
| 15.33 | 16.17 |
| 2.33 | 17.30 |
| 3.33 | 10.93 |
| 3.67 | 6.47 |
| 5.00 | 16.77 |
| 4.00 | 6.43 |
| 9.33 | 14.37 |
| 22.00 | 13.77 |
| 3.67 | 10.43 |
| 3.67 | 7.13 |
| 9.33 | 14.70 |
| 7.00 | 29.73 |
| 14.67 | 27.17 |
| 6.33 | 26.93 |
| 3.00 | 23.07 |
| 3.67 | 5.30 |
| 4.33 | 13.03 |
| 3.67 | 12.57 |
| 15.33 | 19.80 |
| 14.33 | 12.37 |
| 6.33 | 9.80 |
| 2.67 | 9.17 |

| | |
|---|---|
| 3.00 | 5.10 |
| 3.33 | 24.80 |
| 11.00 | 26.57 |
| 38.33 | 20.83 |
| 5.67 | 6.63 |
| 6.00 | 22.53 |
| 4.33 | 18.27 |
| 10.00 | 14.63 |
| 8.67 | 26.40 |
| 7.33 | 20.77 |
| 8.00 | 19.07 |
| 7.33 | 31.43 |
| 15.00 | 29.07 |
| 4.67 | 24.80 |
| 2.00 | 4.70 |
| 7.00 | 14.43 |
| 17.33 | 32.67 |
| 3.33 | 9.10 |
| 13.00 | 23.37 |
| 5.00 | 18.37 |
| 13.00 | 14.90 |
| 6.00 | 21.60 |
| 15.67 | 13.00 |
| 2.00 | 12.70 |
| 11.67 | 40.63 |

| | |
|---|---|
| 22.33 | 46.83 |
| 2.67 | 21.70 |
| 144.00 | 40.80 |
| 103.67 | 39.40 |
| 15.00 | 29.37 |
| 23.67 | 32.93 |
| 9.00 | 22.10 |
| 5.00 | 19.20 |
| 22.00 | 33.10 |
| 5.67 | 3.57 |
| 5.33 | 9.07 |
| 24.33 | 27.07 |
| 3.67 | 5.10 |
| 4.00 | 8.40 |
| 4.00 | 15.70 |
| 4.33 | 8.50 |
| 5.67 | 17.87 |
| 4.33 | 5.73 |
| 5.00 | 14.63 |
| 2.67 | 17.67 |
| 2.67 | 10.23 |
| 4.33 | 17.87 |

| | |
|---|---|
| 11.67 | 11.97 |
| 13.00 | 33.73 |
| 8.00 | 8.10 |
| 11.33 | 16.53 |
| 3.33 | 8.17 |
| 2.33 | 7.57 |
| 8.33 | 13.33 |
| 9.00 | 26.07 |
| 52.67 | 33.13 |
| 6.33 | 15.27 |
| 3.67 | 9.03 |
| 27.33 | 39.73 |
| 19.00 | 36.93 |
| 4.00 | 25.87 |
| 2.33 | 19.13 |
| 8.67 | 19.80 |
| 14.67 | 20.83 |
| 5.00 | 40.87 |
| 4.00 | 13.13 |
| 3.00 | 22.80 |
| 3.00 | 4.03 |
| 18.67 | 39.13 |
| 17.00 | 40.43 |
| 10.33 | 36.57 |
| 9.67 | 23.33 |

| | |
|---|---|
| 5.67 | 7.10 |
| 8.33 | 23.43 |
| 9.67 | 30.67 |
| 7.67 | 18.77 |
| 2.00 | 14.40 |
| 2.00 | 3.57 |
| 3.67 | 17.43 |
| 2.00 | 7.50 |
| 13.67 | 23.80 |
| 13.00 | 13.57 |
| 33.00 | 19.73 |
| 4.00 | 18.73 |
| 4.33 | 19.20 |
| 6.33 | 7.73 |
| 4.67 | 14.27 |
| 22.00 | 21.20 |
| 9.67 | 17.87 |
| 15.00 | 29.90 |
| 15.67 | 17.07 |
| 7.67 | 10.70 |

| | |
|---|---|
| 18.00 | 41.03 |
| 3.67 | 12.27 |
| 4.67 | 19.70 |
| 17.00 | 29.07 |
| 4.00 | 3.53 |
| 32.67 | 28.10 |
| 11.33 | 30.47 |
| 3.33 | 14.70 |
| 73.67 | 43.93 |
| 7.67 | 18.10 |
| 3.00 | 10.43 |
| 2.33 | 15.70 |
| 2.00 | 10.30 |
| 3.33 | 17.13 |
| 2.33 | 14.80 |
| 3.67 | 10.40 |
| 11.00 | 19.10 |
| 20.00 | 20.13 |
| 9.33 | 13.40 |
| 61.67 | 54.40 |
| 22.67 | 11.97 |
| 28.67 | 34.83 |
| 42.33 | 40.90 |

| | |
|---|---|
| 4.67 | 26.20 |
| 26.33 | 44.00 |
| 55.33 | 28.63 |
| 5.33 | 16.70 |
| 10.00 | 15.27 |
| 18.33 | 34.80 |
| 4.67 | 9.27 |
| 11.33 | 29.13 |
| 4.33 | 21.27 |
| 2.00 | 9.20 |
| 8.00 | 14.73 |
| 3.67 | 4.47 |
| 4.00 | 10.03 |
| 2.33 | 10.70 |
| 5.33 | 12.83 |
| 4.00 | 14.37 |
| 3.33 | 14.63 |
| 3.33 | 15.57 |
| 2.33 | 9.67 |
| 19.33 | 19.03 |
| 25.67 | 14.97 |
| 17.00 | 21.20 |

| | |
|---|---|
| 9.67 | 14.67 |
| 12.67 | 25.03 |
| 4.00 | 13.80 |
| 7.67 | 17.63 |
| 5.33 | 12.17 |
| 53.00 | 60.37 |
| 3.00 | 10.93 |
| 19.67 | 16.13 |
| 13.00 | 24.10 |
| 9.33 | 19.17 |
| 5.33 | 10.67 |
| 4.00 | 30.13 |
| 4.33 | 7.03 |
| 2.33 | 8.17 |
| 3.33 | 14.77 |
| 5.00 | 9.53 |
| 20.33 | 40.97 |
| 8.33 | 22.00 |
| 8.33 | 14.57 |
| 4.67 | 8.43 |
| 12.33 | 24.83 |
| 2.33 | 4.23 |
| 47.00 | 34.90 |

| | |
|---|---|
| 7.00 | 15.50 |
| 2.67 | 9.90 |
| 3.33 | 25.33 |
| 8.00 | 33.90 |
| 2.00 | 20.60 |
| 8.67 | 23.10 |
| 4.33 | 15.87 |
| 30.00 | 21.80 |
| 13.33 | 7.37 |
| 3.00 | 3.20 |
| 4.67 | 17.27 |
| 11.67 | 25.93 |
| 4.67 | 22.07 |
| 22.67 | 16.37 |
| 11.00 | 16.00 |
| 5.33 | 16.93 |
| 2.00 | 13.10 |
| 3.33 | 13.27 |
| 2.00 | 3.50 |
| 8.33 | 16.17 |
| 13.00 | 19.70 |
| 5.67 | 39.30 |
| 4.00 | 6.27 |

| | |
|---|---|
| 12.33 | 15.47 |
| 7.00 | 19.00 |
| 21.33 | 17.03 |
| 6.00 | 28.13 |
| 12.33 | 20.20 |
| 6.00 | 11.30 |
| 11.00 | 30.50 |
| 7.33 | 10.73 |
| 2.67 | 11.53 |
| 7.00 | 32.93 |
| 4.33 | 7.40 |
| 15.33 | 24.50 |
| 3.00 | 11.43 |
| 2.00 | 10.50 |
| 39.00 | 18.87 |
| 2.33 | 26.27 |
| 9.00 | 24.07 |
| 11.33 | 20.20 |
| 21.33 | 22.53 |
| 4.67 | 23.63 |

| | |
|---|---|
| 4.67 | 18.90 |
| 3.33 | 20.50 |
| 7.33 | 30.40 |
| 8.33 | 60.37 |
| 2.33 | 5.47 |
| 3.00 | 25.80 |
| 3.00 | 4.03 |
| 3.33 | 13.70 |
| 35.00 | 32.70 |
| 5.67 | 23.83 |
| 9.67 | 17.27 |
| 4.67 | 26.53 |
| 2.67 | 20.40 |
| 34.67 | 42.33 |
| 2.00 | 7.60 |
| 3.33 | 7.40 |
| 31.00 | 42.40 |
| 7.33 | 16.73 |
| 6.33 | 17.63 |
| 19.67 | 37.83 |
| 2.00 | 14.30 |
| 8.00 | 15.00 |
| 3.33 | 5.50 |
| 11.33 | 27.00 |
| 3.67 | 6.93 |

| | |
|---|---|
| 9.67 | 33.17 |
| 5.00 | 13.57 |
| 13.67 | 34.20 |
| 51.00 | 37.23 |
| 10.67 | 19.43 |
| 4.67 | 29.43 |
| 4.67 | 27.97 |
| 3.67 | 12.23 |
| 7.00 | 13.33 |
| 8.67 | 40.50 |
| 2.33 | 20.43 |
| 8.67 | 23.33 |
| 6.67 | 25.17 |
| 5.33 | 17.33 |
| 8.33 | 19.57 |
| 5.67 | 6.63 |
| 3.33 | 8.53 |
| 6.33 | 13.47 |
| 3.33 | 16.07 |
| 20.00 | 32.93 |
| 4.67 | 15.00 |
| 4.33 | 11.77 |
| 2.67 | 8.80 |

| | |
|---|---|
| 5.67 | 7.13 |
| 5.00 | 12.07 |
| 10.67 | 17.03 |
| 7.00 | 9.70 |
| 7.67 | 17.93 |
| 49.67 | 33.87 |
| 32.67 | 40.00 |
| 11.67 | 15.00 |
| 31.67 | 38.57 |
| 12.67 | 21.53 |
| 10.67 | 22.10 |
| 2.00 | 4.57 |
| 3.00 | 15.70 |
| 4.33 | 8.33 |
| 6.67 | 6.87 |
| 5.67 | 19.77 |
| 5.33 | 16.43 |
| 4.33 | 17.87 |
| 4.33 | 7.10 |
| 4.33 | 16.97 |
| 7.00 | 19.20 |
| 3.67 | 5.63 |
| 3.67 | 5.07 |
| 3.00 | 6.87 |

| | |
|---|---|
| 4.00 | 25.00 |
| 5.67 | 5.83 |
| 5.33 | 15.37 |
| 2.67 | 8.90 |
| 3.67 | 17.90 |
| 2.67 | 11.97 |
| 3.33 | 10.40 |
| 2.00 | 9.30 |
| 3.00 | 9.30 |
| 4.33 | 11.70 |
| 3.00 | 4.30 |
| 2.67 | 5.50 |
| 2.33 | 4.87 |
| 4.00 | 7.23 |
| 7.00 | 12.77 |
| 4.67 | 37.30 |
| 3.67 | 7.53 |
| 4.33 | 18.23 |
| 4.67 | 15.60 |
| 10.00 | 15.97 |
| 2.33 | 6.10 |
| 5.00 | 29.50 |

| | |
|---|---|
| 3.67 | 8.23 |
| 6.67 | 22.73 |
| 7.00 | 7.03 |
| 3.00 | 6.63 |
| 5.00 | 18.57 |
| 7.33 | 25.07 |
| 2.00 | 4.17 |
| 2.67 | 8.97 |
| 4.00 | 14.83 |
| 7.00 | 17.43 |
| 4.67 | 12.67 |
| 5.67 | 21.07 |
| 9.67 | 20.80 |
| 2.67 | 8.33 |
| 2.33 | 10.13 |
| 2.67 | 14.60 |
| 6.33 | 13.33 |
| 2.00 | 5.13 |
| 2.67 | 11.53 |
| 2.00 | 12.00 |
| 4.67 | 5.60 |
| 9.33 | 12.27 |

| | |
|---|---|
| 2.33 | 6.73 |
| 7.33 | 39.30 |
| 5.33 | 13.53 |
| 14.00 | 19.87 |
| 4.00 | 8.63 |
| 4.00 | 22.67 |
| 4.33 | 22.70 |
| 3.67 | 20.07 |
| 7.33 | 25.80 |
| 2.67 | 8.20 |
| 10.00 | 18.30 |
| 4.67 | 13.07 |
| 2.33 | 9.13 |
| 2.33 | 2.60 |
| 4.33 | 11.93 |
| 4.00 | 6.27 |
| 3.00 | 8.80 |
| 4.33 | 8.40 |
| 5.33 | 12.93 |
| 4.00 | 17.00 |
| 2.67 | 9.00 |
| 4.00 | 4.10 |
| 3.33 | 9.30 |

| | |
|---|---|
| 5.00 | 19.23 |
| 2.67 | 4.00 |
| 10.00 | 11.27 |
| 5.33 | 13.13 |
| 2.33 | 6.03 |
| 4.33 | 11.53 |
| 8.00 | 27.83 |
| 3.33 | 21.97 |
| 7.00 | 15.13 |
| 6.00 | 16.20 |
| 7.00 | 14.37 |
| 5.33 | 22.27 |
| 5.67 | 39.00 |
| 7.00 | 40.93 |
| 3.00 | 20.60 |
| 2.33 | 7.70 |
| 2.00 | 9.40 |
| 3.00 | 3.37 |
| 3.00 | 7.43 |
| 4.00 | 26.53 |
| 4.67 | 12.40 |
| 2.33 | 11.30 |
| 10.67 | 21.80 |
| 2.67 | 12.33 |
| 4.67 | 14.90 |

| | |
|---|---|
| 2.00 | 8.50 |
| 2.00 | 4.17 |
| 2.67 | 5.30 |
| 2.67 | 7.30 |
| 2.67 | 8.27 |
| 9.67 | 16.43 |
| 2.67 | 15.00 |
| 5.67 | 15.00 |
| 3.00 | 34.03 |
| 2.67 | 21.70 |
| 3.00 | 2.83 |
| 4.00 | 17.60 |
| 6.67 | 12.80 |
| 6.67 | 9.00 |
| 4.67 | 9.60 |
| 6.33 | 17.40 |
| 3.33 | 15.73 |
| 3.33 | 8.63 |
| 5.00 | 11.50 |
| 2.00 | 2.90 |
| 11.00 | 14.23 |
| 4.00 | 6.00 |

| | |
|---|---|
| 14.00 | 19.77 |
| 3.33 | 6.13 |
| 2.00 | 11.20 |
| 2.00 | 7.30 |
| 4.00 | 16.13 |
| 7.33 | 15.50 |
| 3.00 | 3.00 |
| 3.00 | 16.07 |
| 7.00 | 6.77 |
| 2.33 | 5.00 |
| 5.00 | 14.77 |
| 5.33 | 14.10 |
| 6.00 | 13.60 |
| 4.67 | 10.63 |
| 11.67 | 16.93 |
| 7.33 | 16.53 |
| 2.67 | 3.30 |
| 6.33 | 15.63 |
| 2.33 | 9.17 |
| 3.67 | 10.27 |
| 3.33 | 4.33 |
| 4.00 | 7.03 |
| 3.33 | 8.60 |
| 30.33 | 41.33 |

| | |
|---|---|
| 3.67 | 16.90 |
| 8.00 | 13.27 |
| 2.00 | 11.27 |
| 4.00 | 8.13 |
| 3.67 | 6.90 |
| 3.33 | 15.37 |
| 5.33 | 14.53 |
| 2.67 | 15.77 |
| 4.33 | 14.07 |
| 3.00 | 9.07 |
| 7.67 | 11.33 |
| 4.33 | 20.00 |
| 4.33 | 7.67 |
| 15.00 | 20.33 |
| 24.00 | 24.33 |
| 2.67 | 5.67 |
| 5.00 | 12.33 |
| 5.33 | 18.00 |
| 3.67 | 11.00 |
| 2.33 | 15.67 |
| 4.33 | 15.67 |
| 2.33 | 8.33 |

| | |
|---|---|
| 8.17 | 14.67 |
| 7.83 | 19.17 |
| 9.33 | 14.83 |
| 2.33 | 8.33 |
| 10.67 | 13.00 |
| 4.00 | 17.00 |
| 3.67 | 9.00 |
| 5.00 | 26.00 |
| 3.33 | 11.67 |
| 13.00 | 19.67 |
| 42.67 | 21.67 |
| 3.33 | 7.33 |
| 5.00 | 23.67 |
| 6.33 | 13.33 |
| 2.00 | 11.67 |
| 5.67 | 15.67 |
| 4.00 | 9.67 |
| 3.83 | 7.33 |
| 4.33 | 13.67 |
| 13.33 | 21.00 |
| 4.67 | 8.50 |
| 4.33 | 10.67 |
| 2.67 | 5.33 |

| | |
|---|---|
| 3.33 | 6.00 |
| 5.83 | 11.83 |
| 7.00 | 14.00 |
| 2.67 | 3.33 |
| 7.67 | 29.00 |
| 10.33 | 33.00 |
| 4.00 | 8.67 |
| 3.00 | 8.67 |
| 5.33 | 16.67 |
| 25.67 | 22.33 |
| 4.67 | 9.67 |
| 10.00 | 11.00 |
| 4.33 | 13.33 |
| 12.33 | 18.33 |
| 3.00 | 6.00 |
| 3.33 | 14.17 |
| 17.00 | 11.00 |
| 5.67 | 17.33 |
| 9.33 | 36.00 |
| 2.33 | 9.67 |
| 3.67 | 15.00 |
| 4.67 | 19.33 |
| 13.67 | 28.33 |
| 6.33 | 14.33 |

| | |
|---|---|
| 6.50 | 18.83 |
| 19.00 | 16.00 |
| 6.00 | 18.33 |
| 7.50 | 17.00 |
| 5.17 | 13.50 |
| 3.50 | 16.17 |
| 5.67 | 19.33 |
| 2.67 | 4.00 |
| 2.33 | 4.67 |
| 16.00 | 24.33 |
| 7.33 | 31.00 |
| 3.00 | 11.00 |
| 4.50 | 10.00 |
| 9.67 | 29.33 |
| 3.67 | 19.00 |
| 3.00 | 23.00 |
| 13.50 | 13.83 |
| 7.33 | 20.00 |
| 3.83 | 7.00 |
| 13.33 | 27.67 |
| 15.33 | 36.17 |
| 4.00 | 4.00 |
| 4.33 | 9.00 |

| | |
|---|---|
| 4.33 | 22.00 |
| 14.00 | 38.33 |
| 3.67 | 17.00 |
| 2.00 | 5.33 |
| 12.83 | 17.00 |
| 6.33 | 23.33 |
| 2.33 | 8.67 |
| 4.67 | 23.33 |
| 5.17 | 15.00 |
| 5.33 | 9.33 |
| 8.00 | 17.67 |
| 11.33 | 13.67 |
| 4.00 | 10.33 |
| 22.00 | 21.50 |
| 10.67 | 8.67 |
| 4.33 | 9.33 |
| 7.83 | 9.83 |
| 3.50 | 9.17 |
| 5.17 | 8.33 |
| 4.67 | 10.00 |
| 4.00 | 16.00 |
| 4.00 | 6.33 |

| | |
|---|---|
| 6.33 | 8.17 |
| 7.17 | 17.17 |
| 3.50 | 4.67 |
| 2.67 | 18.67 |
| 3.00 | 5.33 |
| 14.00 | 46.00 |
| 11.50 | 23.83 |
| 3.00 | 24.67 |
| 3.67 | 18.33 |
| 11.50 | 22.00 |
| 8.67 | 23.67 |
| 3.00 | 5.00 |
| 2.83 | 10.33 |
| 3.33 | 6.67 |
| 3.00 | 13.00 |
| 6.17 | 16.17 |
| 9.67 | 19.33 |
| 2.67 | 9.33 |
| 7.67 | 25.67 |
| 3.33 | 8.67 |
| 6.00 | 12.00 |
| 2.67 | 8.33 |

| | |
|---|---|
| 22.83 | 20.33 |
| 5.00 | 31.00 |
| 3.50 | 4.67 |
| 2.67 | 3.67 |
| 3.33 | 11.33 |
| 3.67 | 8.00 |
| 2.67 | 11.00 |
| 17.50 | 18.17 |
| 3.00 | 15.00 |
| 3.00 | 4.33 |
| 37.00 | 38.83 |
| 10.83 | 20.17 |
| 3.33 | 20.67 |
| 3.33 | 5.33 |
| 4.33 | 13.67 |
| 16.33 | 41.33 |
| 4.67 | 8.33 |
| 2.00 | 4.67 |
| 42.67 | 57.50 |
| 2.67 | 8.67 |
| 7.00 | 15.33 |
| 4.33 | 21.67 |
| 15.17 | 35.83 |

| | |
|---|---|
| 11.67 | 18.33 |
| 3.50 | 21.67 |
| 3.83 | 34.00 |
| 8.67 | 14.67 |
| 6.17 | 10.33 |
| 2.67 | 18.33 |
| 3.00 | 10.33 |
| 4.67 | 11.67 |
| 2.00 | 5.33 |
| 3.17 | 15.33 |
| 13.00 | 29.00 |
| 3.17 | 8.17 |
| 5.17 | 14.00 |
| 3.00 | 2.67 |
| 6.67 | 30.50 |
| 5.33 | 12.67 |
| 26.17 | 22.50 |
| 8.67 | 25.67 |
| 4.00 | 3.67 |
| 4.67 | 17.00 |
| 3.67 | 10.00 |
| 3.67 | 16.00 |
| 3.67 | 8.33 |
| 12.67 | 35.33 |

| | |
|---|---|
| 5.00 | 21.00 |
| 7.50 | 8.33 |
| 2.33 | 20.00 |
| 5.33 | 16.00 |
| 2.33 | 6.00 |
| 4.00 | 35.00 |
| 4.67 | 19.00 |
| 2.33 | 14.00 |
| 4.67 | 22.00 |
| 3.50 | 11.33 |
| 3.33 | 12.33 |
| 4.50 | 6.83 |
| 2.33 | 11.67 |
| 16.33 | 34.00 |
| 5.00 | 7.00 |
| 7.00 | 11.67 |
| 5.67 | 8.67 |
| 7.00 | 16.00 |
| 6.50 | 5.67 |
| 2.67 | 7.33 |
| 20.67 | 34.00 |
| 7.00 | 32.67 |
| 4.33 | 25.33 |

| | |
|---|---|
| 2.00 | 19.00 |
| 19.67 | 21.00 |
| 7.33 | 12.00 |
| 4.00 | 13.67 |
| 32.00 | 28.17 |
| 4.50 | 10.00 |
| 3.00 | 19.33 |
| 3.67 | 14.33 |
| 2.67 | 7.00 |
| 5.00 | 9.67 |
| 2.00 | 13.67 |
| 3.67 | 8.67 |
| 3.00 | 28.00 |
| 6.33 | 11.33 |
| 3.00 | 17.00 |
| 45.17 | 22.33 |
| 3.50 | 7.00 |
| 14.83 | 29.67 |
| 4.67 | 25.33 |
| 7.33 | 12.33 |
| 4.67 | 17.33 |
| 2.67 | 8.33 |
| 8.33 | 11.33 |

| | |
|---|---|
| 13.33 | 18.33 |
| 12.33 | 14.67 |
| 4.00 | 10.67 |
| 5.83 | 18.00 |
| 12.50 | 15.00 |
| 8.00 | 22.67 |
| 9.83 | 15.17 |
| 4.67 | 11.33 |
| 2.67 | 5.00 |
| 21.33 | 17.67 |
| 5.33 | 9.50 |
| 4.33 | 16.33 |
| 8.33 | 14.67 |
| 4.33 | 33.33 |
| 2.50 | 13.00 |
| 3.00 | 4.67 |
| 13.67 | 22.00 |
| 2.00 | 4.00 |
| 2.67 | 8.33 |
| 4.67 | 8.33 |
| 15.33 | 26.33 |
| 8.00 | 26.00 |
| 7.33 | 28.50 |
| 3.50 | 6.50 |

| | |
|---|---|
| 2.33 | 6.67 |
| 2.00 | 10.00 |
| 6.33 | 17.33 |
| 2.00 | 5.33 |
| 11.00 | 21.67 |
| 2.67 | 4.33 |
| 2.00 | 6.00 |
| 8.33 | 15.33 |
| 3.67 | 25.00 |
| 2.33 | 6.33 |
| 8.50 | 13.50 |
| 3.33 | 6.67 |
| 3.00 | 8.33 |
| 7.67 | 26.67 |
| 2.33 | 5.00 |
| 2.00 | 4.00 |
| 4.00 | 9.67 |
| 3.50 | 9.33 |
| 49.17 | 48.50 |
| 8.67 | 22.33 |
| 29.17 | 32.33 |
| 2.00 | 2.00 |
| 9.67 | 16.00 |
| 3.33 | 9.33 |
| 2.00 | 5.33 |

| | |
|---|---|
| 8.17 | 11.50 |
| 6.17 | 12.67 |
| 3.00 | 20.00 |
| 4.17 | 5.83 |
| 10.33 | 24.33 |
| 2.33 | 17.67 |
| 3.33 | 4.33 |
| 2.50 | 19.67 |
| 9.33 | 29.33 |
| 8.67 | 17.67 |
| 9.67 | 22.00 |
| 4.67 | 11.33 |
| 20.00 | 22.17 |
| 9.50 | 15.17 |
| 2.67 | 14.67 |
| 2.67 | 24.00 |
| 2.33 | 1.67 |
| 6.67 | 49.00 |
| 5.67 | 25.00 |
| 5.67 | 27.33 |
| 3.00 | 17.00 |
| 2.33 | 2.67 |
| 11.83 | 19.17 |

| | |
|---|---|
| 20.17 | 18.50 |
| 3.67 | 3.67 |
| 4.33 | 14.00 |
| 3.67 | 7.33 |
| 6.67 | 15.00 |
| 4.00 | 12.00 |
| 10.33 | 16.33 |
| 4.00 | 8.33 |
| 2.00 | 13.00 |
| 5.67 | 12.67 |
| 2.00 | 5.33 |
| 22.00 | 12.83 |
| 9.33 | 19.00 |
| 4.67 | 8.00 |
| 8.00 | 10.00 |
| 3.33 | 13.33 |
| 2.67 | 6.67 |
| 2.33 | 7.00 |
| 5.67 | 32.00 |
| 18.83 | 12.67 |
| 7.00 | 18.67 |

| | |
|---|---|
| 12.33 | 47.17 |
| 4.00 | 10.67 |
| 3.00 | 11.33 |
| 2.67 | 16.00 |
| 3.67 | 21.00 |
| 2.67 | 9.33 |
| 4.00 | 20.00 |
| 3.00 | 16.33 |
| 2.00 | 7.33 |
| 17.50 | 32.00 |
| 3.00 | 9.67 |
| 2.33 | 5.67 |
| 14.33 | 30.00 |
| 2.67 | 12.33 |
| 2.67 | 13.50 |
| 3.33 | 9.67 |
| 3.00 | 17.00 |
| 2.00 | 6.67 |
| 5.67 | 32.67 |
| 2.33 | 12.33 |
| 6.83 | 8.33 |
| 6.33 | 12.00 |
| 10.67 | 5.33 |
| 7.67 | 15.33 |
| 3.67 | 6.67 |

| | |
|---|---|
| 2.33 | 4.33 |
| 4.33 | 15.33 |
| 16.33 | 17.00 |
| 3.00 | 5.00 |
| 3.33 | 2.83 |
| 4.00 | 14.33 |
| 13.83 | 20.00 |
| 4.00 | 13.00 |
| 11.83 | 15.33 |
| 2.67 | 9.67 |
| 10.33 | 22.00 |
| 3.00 | 12.00 |
| 8.33 | 33.00 |
| 6.00 | 9.00 |
| 10.00 | 10.67 |
| 4.83 | 12.33 |
| 14.33 | 27.67 |
| 41.00 | 40.00 |
| 3.00 | 9.00 |
| 2.67 | 7.00 |
| 3.00 | 7.67 |

| | |
|---|---|
| 2.67 | 4.33 |
| 4.00 | 8.33 |
| 2.00 | 3.67 |
| 7.50 | 18.00 |
| 2.67 | 6.00 |
| 4.00 | 21.33 |
| 10.67 | 13.67 |
| 2.00 | 6.00 |
| 22.33 | 23.00 |
| 2.00 | 5.67 |
| 2.50 | 12.33 |
| 6.83 | 27.67 |
| 4.33 | 18.00 |
| 3.00 | 8.33 |
| 4.17 | 7.67 |
| 8.33 | 26.33 |
| 5.83 | 17.00 |
| 3.00 | 8.33 |
| 2.00 | 12.33 |
| 6.67 | 16.00 |
| 6.67 | 15.67 |

| | |
|---|---|
| 8.00 | 25.00 |
| 2.00 | 7.00 |
| 3.33 | 12.00 |
| 3.00 | 7.33 |
| 7.00 | 22.00 |
| 5.00 | 8.17 |
| 9.33 | 20.67 |
| 19.50 | 23.67 |
| 5.00 | 5.67 |
| 5.33 | 27.33 |
| 6.00 | 10.33 |
| 2.00 | 7.67 |
| 2.67 | 16.00 |
| 2.67 | 10.67 |
| 29.00 | 17.33 |
| 7.50 | 20.17 |
| 6.00 | 7.50 |
| 3.67 | 11.00 |
| 3.00 | 7.67 |
| 2.00 | 7.67 |
| 3.00 | 12.33 |
| 15.00 | 30.67 |
| 6.33 | 10.33 |

| | |
|---|---|
| 4.00 | 10.00 |
| 3.67 | 15.33 |
| 4.67 | 26.00 |
| 3.33 | 3.00 |
| 2.67 | 29.67 |
| 2.00 | 6.00 |
| 7.83 | 21.50 |
| 7.33 | 7.67 |
| 2.33 | 12.67 |
| 14.17 | 23.17 |
| 3.67 | 15.00 |
| 5.33 | 8.00 |
| 5.67 | 3.33 |
| 4.33 | 19.33 |
| 19.33 | 40.33 |
| 2.67 | 5.67 |
| 3.67 | 12.00 |
| 7.33 | 17.00 |
| 3.00 | 9.00 |
| 5.00 | 19.67 |
| 10.67 | 17.33 |
| 5.00 | 12.67 |
| 6.33 | 14.00 |
| 7.67 | 18.67 |

| | |
|---|---|
| 3.33 | 14.00 |
| 6.50 | 9.17 |
| 8.83 | 11.67 |
| 21.50 | 24.67 |
| 10.83 | 8.00 |
| 4.00 | 8.33 |
| 7.00 | 13.33 |
| 2.00 | 4.67 |
| 2.67 | 14.00 |
| 3.83 | 9.50 |
| 9.00 | 24.00 |
| 4.67 | 18.33 |
| 3.00 | 10.33 |
| 5.17 | 11.83 |
| 8.33 | 13.00 |
| 4.33 | 6.00 |
| 2.67 | 30.00 |
| 2.33 | 5.00 |
| 2.33 | 4.33 |
| 2.67 | 3.33 |
| 3.33 | 9.00 |

| | |
|---|---|
| 2.33 | 8.33 |
| 3.67 | 3.67 |
| 18.00 | 35.33 |
| 6.67 | 15.67 |
| 2.00 | 6.00 |
| 4.00 | 36.00 |
| 14.33 | 22.67 |
| 4.33 | 9.67 |
| 5.83 | 16.50 |
| 2.33 | 7.67 |
| 4.67 | 33.67 |
| 4.00 | 24.00 |
| 3.00 | 9.67 |
| 8.67 | 14.67 |
| 4.17 | 12.33 |
| 17.00 | 22.67 |
| 20.50 | 35.50 |
| 5.67 | 11.67 |
| 3.33 | 37.00 |
| 2.00 | 4.67 |
| 2.67 | 4.67 |

| | |
|---|---|
| 7.17 | 26.00 |
| 3.67 | 11.67 |
| 2.67 | 2.33 |
| 7.00 | 6.00 |
| 33.00 | 17.67 |
| 2.33 | 5.00 |
| 25.33 | 22.00 |
| 5.83 | 12.50 |
| 3.33 | 6.00 |
| 3.67 | 16.33 |
| 11.33 | 17.33 |
| 9.00 | 12.33 |
| 7.00 | 8.67 |
| 3.67 | 24.67 |
| 2.83 | 8.67 |
| 4.67 | 17.67 |
| 3.33 | 10.33 |
| 2.00 | 4.00 |
| 4.33 | 27.33 |

| | |
|---|---|
| 3.00 | 9.00 |
| 2.00 | 14.00 |
| 5.00 | 17.00 |
| 4.33 | 14.67 |
| 2.33 | 7.67 |
| 2.67 | 4.67 |
| 4.33 | 11.00 |
| 26.67 | 36.17 |
| 8.50 | 27.17 |
| 2.33 | 5.00 |
| 2.00 | 3.00 |
| 2.50 | 9.00 |
| 32.00 | 40.50 |
| 3.00 | 25.00 |
| 3.33 | 18.00 |
| 2.33 | 5.67 |
| 8.33 | 11.33 |
| 2.67 | 6.00 |
| 2.00 | 11.67 |
| 2.83 | 11.83 |
| 3.67 | 7.67 |
| 55.83 | 45.67 |
| 5.67 | 8.67 |
| 2.33 | 13.33 |

| | |
|---|---|
| 7.00 | 31.00 |
| 10.00 | 14.67 |
| 3.67 | 8.00 |
| 4.33 | 11.83 |
| 6.17 | 4.50 |
| 8.00 | 7.33 |
| 2.33 | 10.67 |
| 3.33 | 12.33 |
| 5.00 | 10.33 |
| 3.33 | 8.00 |
| 9.33 | 26.00 |
| 13.67 | 10.50 |
| 7.00 | 12.67 |
| 8.33 | 29.00 |
| 2.00 | 13.33 |
| 2.67 | 8.00 |
| 2.67 | 7.00 |
| 4.83 | 16.67 |
| 3.00 | 13.00 |
| 11.00 | 9.00 |
| 3.00 | 16.00 |
| 4.33 | 18.67 |
| 3.67 | 3.00 |
| 3.00 | 14.33 |
| 7.50 | 16.50 |

| | |
|---|---|
| 17.67 | 32.33 |
| 2.00 | 6.00 |
| 2.00 | 10.00 |
| 3.67 | 15.00 |
| 16.00 | 41.33 |
| 3.33 | 12.33 |
| 2.00 | 8.00 |
| 22.67 | 15.33 |
| 2.67 | 8.33 |
| 3.33 | 14.33 |
| 3.33 | 13.50 |
| 4.67 | 11.33 |
| 2.67 | 13.33 |
| 9.67 | 14.67 |
| 15.67 | 17.00 |
| 7.67 | 11.67 |
| 16.67 | 9.00 |
| 9.50 | 13.83 |
| 4.00 | 18.33 |
| 22.67 | 44.33 |
| 24.17 | 16.00 |
| 2.00 | 3.67 |

| | |
|---|---|
| 2.33 | 11.00 |
| 41.00 | 30.67 |
| 7.67 | 28.33 |
| 3.00 | 19.67 |
| 5.33 | 14.00 |
| 3.00 | 15.00 |
| 6.00 | 9.67 |
| 3.67 | 9.67 |
| 2.33 | 9.33 |
| 9.00 | 19.00 |
| 2.33 | 7.67 |
| 3.33 | 12.67 |
| 2.67 | 11.67 |
| 2.33 | 9.00 |
| 10.83 | 15.17 |
| 2.00 | 18.00 |
| 8.67 | 21.33 |
| 3.67 | 3.00 |
| 7.67 | 17.63 |
| 4.33 | 19.40 |
| 9.00 | 8.07 |
| 3.67 | 6.63 |
| 15.00 | 31.67 |

| | |
|---|---|
| 5.33 | 12.17 |
| 14.00 | 16.33 |
| 4.67 | 21.03 |
| 2.67 | 7.60 |
| 12.67 | 14.57 |
| 3.00 | 10.93 |
| 8.33 | 8.93 |
| 12.00 | 19.43 |
| 3.33 | 5.33 |
| 2.33 | 6.93 |
| 11.67 | 25.13 |
| 4.00 | 30.13 |
| 2.33 | 7.97 |
| 3.33 | 4.80 |
| 3.67 | 13.80 |
| 11.33 | 29.40 |
| 3.33 | 14.77 |
| 4.33 | 9.60 |
| 20.33 | 40.97 |
| 46.33 | 29.20 |
| 8.33 | 14.57 |
| 4.67 | 8.43 |

| | |
|---|---|
| 2.67 | 10.47 |
| 10.67 | 39.20 |
| 39.00 | 48.57 |
| 5.33 | 11.87 |
| 12.00 | 9.40 |
| 3.33 | 9.13 |
| 5.00 | 10.13 |
| 2.67 | 9.90 |
| 2.67 | 9.73 |
| 59.67 | 53.50 |
| 4.00 | 14.77 |
| 16.00 | 38.07 |
| 2.67 | 9.37 |
| 2.67 | 6.70 |
| 4.67 | 28.17 |
| 8.00 | 11.00 |
| 5.67 | 11.07 |
| 4.33 | 8.87 |
| 13.33 | 7.37 |
| 2.00 | 14.70 |
| 3.33 | 37.47 |
| 3.00 | 5.20 |
| 7.33 | 16.63 |
| 6.33 | 33.73 |

| | |
|---|---|
| 2.67 | 10.70 |
| 23.67 | 40.53 |
| 4.00 | 12.40 |
| 2.67 | 7.63 |
| 3.67 | 10.30 |
| 5.00 | 12.40 |
| 22.33 | 15.87 |
| 10.67 | 15.60 |
| 3.33 | 14.33 |
| 4.67 | 25.10 |
| 10.67 | 24.90 |
| 8.67 | 19.73 |
| 2.00 | 5.30 |
| 7.00 | 18.47 |
| 34.67 | 41.17 |
| 2.00 | 13.10 |
| 5.00 | 20.90 |
| 3.33 | 3.70 |
| 10.67 | 24.10 |

| | |
|---|---|
| 6.67 | 14.30 |
| 4.67 | 19.00 |
| 70.33 | 31.33 |
| 20.00 | 33.57 |
| 6.33 | 8.47 |
| 16.67 | 28.43 |
| 8.00 | 26.10 |
| 9.00 | 27.13 |
| 8.33 | 21.67 |
| 8.00 | 26.80 |
| 4.67 | 7.07 |
| 7.00 | 19.00 |
| 3.00 | 14.30 |
| 6.00 | 28.13 |
| 13.33 | 25.27 |
| 12.00 | 19.63 |
| 23.00 | 46.70 |
| 7.00 | 32.93 |
| 5.33 | 9.37 |
| 11.67 | 6.20 |
| 10.00 | 11.23 |
| 2.33 | 8.27 |
| 17.67 | 23.57 |
| 3.33 | 9.80 |
| 2.67 | 9.23 |

| | |
|---|---|
| 7.00 | 13.13 |
| 2.00 | 10.50 |
| 3.67 | 10.60 |
| 3.67 | 7.97 |
| 3.33 | 20.50 |
| 8.33 | 60.37 |
| 11.00 | 7.70 |
| 21.67 | 24.83 |
| 2.33 | 5.47 |
| 11.00 | 20.97 |
| 6.00 | 16.00 |
| 6.33 | 14.50 |
| 3.00 | 25.80 |
| 14.67 | 25.70 |
| 3.67 | 23.13 |
| 5.00 | 7.27 |
| 6.33 | 14.50 |
| 4.67 | 26.53 |
| 2.67 | 20.40 |
| 9.33 | 17.67 |
| 8.33 | 14.57 |
| 4.00 | 13.80 |
| 7.67 | 18.93 |
| 12.33 | 33.80 |
| 13.33 | 35.87 |

| | |
|---|---|
| 3.33 | 19.27 |
| 5.00 | 36.20 |
| 4.67 | 16.77 |
| 4.00 | 6.43 |
| 22.00 | 13.77 |
| 3.67 | 7.13 |
| 7.67 | 15.00 |
| 11.33 | 27.00 |
| 3.33 | 11.13 |
| 11.67 | 11.60 |
| 3.00 | 5.10 |
| 11.00 | 26.57 |
| 5.67 | 6.63 |
| 2.00 | 4.70 |
| 7.00 | 14.43 |
| 51.00 | 37.23 |
| 4.67 | 29.43 |
| 11.33 | 40.63 |
| 5.33 | 9.07 |
| 4.00 | 8.40 |
| 4.00 | 15.70 |
| 4.33 | 8.50 |
| 5.33 | 16.70 |

| | |
|---|---|
| 4.33 | 5.73 |
| 5.00 | 14.63 |
| 2.67 | 17.67 |
| 8.67 | 40.50 |
| 4.33 | 14.83 |
| 3.33 | 11.17 |
| 13.00 | 33.73 |
| 3.33 | 8.17 |
| 5.67 | 6.63 |
| 9.00 | 26.07 |
| 52.67 | 33.13 |
| 9.00 | 32.20 |
| 3.33 | 8.53 |
| 3.67 | 9.03 |
| 19.00 | 36.93 |
| 4.00 | 25.87 |
| 6.00 | 14.40 |
| 7.33 | 13.77 |
| 4.00 | 13.13 |
| 3.00 | 22.80 |
| 2.00 | 3.57 |

| | |
|---|---|
| 2.00 | 7.50 |
| 3.33 | 16.07 |
| 13.67 | 23.80 |
| 13.00 | 13.57 |
| 4.00 | 18.73 |
| 6.33 | 7.73 |
| 15.00 | 29.90 |
| 19.67 | 32.93 |
| 17.00 | 29.07 |
| 4.67 | 15.00 |
| 3.00 | 12.57 |
| 2.33 | 14.80 |
| 10.33 | 18.20 |
| 3.33 | 7.47 |
| 22.67 | 11.97 |
| 7.00 | 9.70 |
| 7.67 | 17.93 |
| 2.00 | 9.20 |
| 4.00 | 10.03 |
| 3.33 | 15.57 |
| 10.67 | 22.10 |
| 2.33 | 9.67 |
| 6.33 | 6.53 |

| | |
|---|---|
| 3.00 | 2.83 |
| 5.33 | 18.87 |
| 5.67 | 10.40 |
| 3.00 | 5.37 |
| 4.00 | 14.30 |
| 5.67 | 16.07 |
| 2.67 | 10.27 |
| 4.67 | 14.50 |
| 2.67 | 14.10 |
| 3.67 | 23.17 |
| 4.33 | 5.83 |
| 4.00 | 13.73 |
| 3.00 | 9.70 |
| 3.67 | 9.80 |
| 3.00 | 9.30 |
| 3.67 | 10.93 |
| 5.67 | 10.93 |
| 4.00 | 33.00 |
| 2.00 | 11.20 |
| 4.00 | 18.23 |
| 4.33 | 14.67 |
| 9.00 | 15.07 |
| 5.67 | 12.33 |

| | |
|---|---|
| 2.33 | 6.43 |
| 4.33 | 12.17 |
| 2.00 | 8.27 |
| 3.00 | 16.07 |
| 2.00 | 5.13 |
| 2.00 | 12.00 |
| 2.33 | 5.00 |
| 6.00 | 36.30 |
| 4.67 | 11.37 |
| 3.67 | 20.60 |
| 3.67 | 19.43 |
| 2.33 | 9.13 |
| 4.00 | 6.27 |
| 3.00 | 8.80 |
| 3.00 | 3.90 |
| 2.33 | 5.60 |
| 2.67 | 6.80 |
| 2.33 | 4.00 |
| 3.00 | 20.57 |
| 4.67 | 16.20 |
| 4.33 | 18.43 |
| 5.00 | 39.00 |
| 6.33 | 39.00 |
| 3.67 | 6.90 |
| 4.00 | 9.80 |

| | |
|---|---|
| 10.00 | 21.13 |
| 7.67 | 12.63 |

**Appendix 2:** Conserv

| Accession Number |
|---|
| 24212701 |
| 24212701 |
| 24212702 |
| 24212702 |
| 24212702 |
| 24212702 |
| 24212702 |
| 24212702 |
| 24212702 |
| 24212702 |
| 24212702 |
| 24212702 |
| 24212703 |
| 24212705 |
| 24212705 |
| 24212705 |
| 24212705 |
| 24212705 |
| 24212705 |
| 24212705 |
| 24212705 |
| 24212705 |
| 24212705 |
| 24212705 |
| 24212705 |
| 24212705 |
| 24212705 |
| 24212705 |
| 24212705 |
| 24212705 |
| 24212705 |
| 24212706 |
| 24212706 |
| 24212706 |
| 24212706 |
| 24212706 |
| 24212706 |
| 24212706 |
| 24212706 |
| 24212706 |
| 24212706 |
| 24212706 |
| 24212706 |
| 24212706 |
| 24212709 |
| 24212709 |

24212709
24212709
24212709
24212709
24212709
24212709
24212711
24212711
24212714
24212720
24212720
24212720
24212720
24212720
24212720
24212720
24212720
24212721
24212721
24212721
24212731
24212731
24212731
24212731
24212731
24212731
24212731
24212732
24212733
24212733
24212733
24212733
24212733
24212733
24212733
24212735
24212737
24212737
24212740
24212740
24212740
24212742
24212742
24212743
24212743
24212743
24212743
24212743

24212745
24212749
24212749
24212751
24212751
24212751
24212751
24212751
24212751
24212751
24212751
24212751
24212751
24212751
24212752
24212752
24212752
24212752
24212752
24212752
24212752
24212752
24212752
24212752
24212752
24212752
24212752
24212752
24212752
24212752
24212752
24212752
24212752
24212752
24212754
24212754
24212754
24212754
24212754
24212756
24212756
24212756
24212761
24212761
24212762
24212762
24212762
24212767

24212767
24212767
24212767
24212772
24212772
24212772
24212772
24212772
24212773
24212773
24212773
24212773
24212773
24212781
24212782
24212782
24212782
24212783
24212783
24212783
24212783
24212783
24212783
24212783
24212783
24212783
24212783
24212783
24212785
24212793
24212798
24212802
24212807
24212807
24212807
24212808
24212810
24212813
24212813
24212813
24212813
24212813
24212813
24212813
24212813
24212814
24212815
24212817

24212817
24212817
24212817
24212817
24212817
24212818
24212824
24212824
24212824
24212825
24212826
24212826
24212826
24212826
24212827
24212827
24212827
24212827
24212827
24212830
24212830
24212830
24212830
24212834
24212834
24212835
24212835
24212835
24212835
24212838
24212838
24212838
24212838
24212838
24212838
24212838
24212838
24212838
24212838
24212838
24212838
24212838
24212838
24212838
24212841
24212841
24212841
24212841

24212841
24212841
24212841
24212844
24212846
24212846
24212846
24212846
24212846
24212846
24212846
24212846
24212848
24212848
24212848
24212848
24212848
24212848
24212849
24212853
24212853
24212853
24212853
24212853
24212853
24212853
24212853
24212857
24212857
24212857
24212857
24212857
24212857
24212857
24212857
24212860
24212860
24212860
24212860
24212860
24212860
24212860
24212861
24212861
24212861
24212861
24212861
24212861

24212866
24212866
24212866
24212866
24212878
24212878
24212878
24212878
24212878
24212878
24212878
24212878
24212878
24212879
24212879
24212879
24212879
24212879
24212879
24212879
24212884
24212884
24212884
24212884
24212884
24212884
24212884
24212884
24212885
24212885
24212885
24212885
24212885
24212885
24212885
24212885
24212885
24212885
24212885
24212885
24212885
24212905
24212905
24212905
24212905
24212906
24212918
24212918

24212918
24212922
24212922
24212922
24212922
24212922
24212922
24212922
24212922
24212922
24212922
24212922
24212922
24212922
24212922
24212922
24212925
24212925
24212929
24212929
24212929
24212941
24212943
24212943
24212943
24212947
24212947
24212950
24212950
24212950
24212950
24212950
24212950
24212950
24212951
24212951
24212951
24212951
24212951
24212951
24212951
24212951
24212951
24212951
24212951
24212951
24212951
24212951

24212951
24212951
24212951
24212951
24212951
24212951
24212951
24212951
24212951
24212951
24212953
24212955
24212955
24212956
24212956
24212956
24212956
24212956
24212956
24212963
24212963
24212963
24212963
24212964
24212964
24212971
24212971
24212972
24212972
24212972
24212972
24212974
24212974
24212974
24212976
24212976
24212976
24212978
24212980
24212980
24212980
24212980
24212980
24212980
24212980
24212980
24212980
24212984

24212984
24212984
24212984
24212998
24212999
24213000
24213000
24213000
24213009
24213009
24213009
24213009
24213010
24213010
24213010
24213010
24213011
24213011
24213011
24213013
24213013
24213013
24213013
24213013
24213013
24213013
24213013
24213013
24213013
24213013
24213013
24213013
24213013
24213013
24213013
24213013
24213013
24213013
24213013
24213013
24213013
24213013
24213013
24213013
24213013
24213013
24213013
24213013

24213013
24213013
24213020
24213020
24213025
24213025
24213025
24213025
24213025
24213030
24213030
24213032
24213032
24213035
24213035
24213035
24213035
24213035
24213035
24213035
24213035
24213035
24213045
24213050
24213050
24213051
24213051
24213051
24213051
24213060
24213060
24213060
24213060
24213060
24213060
24213060
24213060
24213060
24213065
24213065
24213065
24213065
24213065
24213065
24213065
24213065
24213065
24213065

24213065
24213065
24213065
24213065
24213065
24213069
24213069
24213069
24213069
24213074
24213074
24213075
24213080
24213091
24213091
24213091
24213091
24213091
24213091
24213091
24213091
24213091
24213091
24213091
24213091
24213091
24213091
24213091
24213091
24213091
24213099
24213099
24213101
24213101
24213102
24213102
24213102
24213102
24213102
24213103
24213106
24213108
24213108
24213108
24213108
24213108
24213109
24213110

24213110
24213110
24213111
24213111
24213111
24213111
24213111
24213111
24213111
24213111
24213111
24213111
24213111
24213111
24213111
24213111
24213112
24213112
24213112
24213112
24213112
24213112
24213112
24213112
24213114
24213114
24213114
24213114
24213114
24213114
24213114
24213114
24213114
24213114
24213115
24213119
24213119
24213128
24213133
24213133
24213133
24213138
24213138
24213138
24213138
24213138
24213138
24213138

24213138
24213138
24213138
24213138
24213138
24213138
24213141
24213141
24213143
24213147
24213147
24213147
24213157
24213157
24213157
24213157
24213157
24213157
24213157
24213157
24213157
24213157
24213157
24213157
24213157
24213159
24213159
24213162
24213162
24213162
24213163
24213163
24213163
24213163
24213164
24213165
24213165
24213166
24213169
24213169
24213169
24213169
24213169
24213171
24213171
24213171
24213171
24213171

24213171
24213171
24213171
24213171
24213171
24213188
24213194
24213194
24213194
24213194
24213194
24213194
24213194
24213198
24213198
24213205
24213205
24213205
24213205
24213205
24213213
24213213
24213232
24213233
24213233
24213233
24213237
24213238
24213238
24213238
24213243
24213244
24213244
24213244
24213246
24213246
24213246
24213246
24213246
24213246
24213246
24213246
24213246
24213246
24213246
24213247
24213247
24213251

24213252
24213252
24213252
24213252
24213252
24213252
24213252
24213252
24213260
24213260
24213260
24213260
24213260
24213260
24213260
24213260
24213260
24213260
24213260
24213260
24213260
24213260
24213260
24213260
24213260
24213260
24213260
24213260
24213260
24213266
24213268
24213275
24213275
24213295
24213295
24213295
24213295
24213311
24213311
24213311
24213312
24213338
24213346
24213346
24213346
24213346
24213346
24213346

24213346
24213346
24213348
24213348
24213348
24213349
24213349
24213349
24213349
24213349
24213349
24213349
24213349
24213356
24213356
24213356
24213356
24213356
24213365
24213365
24213371
24213371
24213371
24213371
24213371
24213371
24213371
24213371
24213371
24213371
24213374
24213374
24213378
24213378
24213378
24213378
24213393
24213393
24213393
24213393
24213393
24213393
24213393
24213399
24213401
24213403
24213420
24213420

24213427
24213427
24213427
24213427
24213427
24213427
24213427
24213427
24213427
24213427
24213427
24213427
24213427
24213427
24213427
24213427
24213427
24213427
24213427
24213434
24213434
24213436
24213436
24213436
24213437
24213437
24213437
24213437
24213437
24213437
24213437
24213437
24213437
24213437
24213437
24213437
24213437
24213437
24213437
24213437
24213437
24213437
24213437
24213437
24213437
24213437
24213437
24213437

24213437
24213437
24213437
24213437
24213438
24213438
24213438
24213438
24213439
24213439
24213439
24213440
24213440
24213440
24213440
24213440
24213440
24213440
24213440
24213440
24213441
24213441
24213442
24213442
24213442
24213442
24213442
24213442
24213442
24213442
24213442
24213442
24213442
24213442
24213442
24213442
24213442
24213442
24213444
24213444
24213444
24213444
24213444
24213444
24213445
24213445
24213445
24213445

24213445
24213445
24213445
24213445
24213445
24213446
24213446
24213446
24213446
24213447
24213447
24213449
24213449
24213450
24213450
24213450
24213451
24213451
24213451
24213451
24213451
24213451
24213451
24213451
24213451
24213451
24213452
24213452
24213452
24213453
24213453
24213453
24213453
24213453
24213453
24213454
24213454
24213454
24213454
24213454
24213454
24213454
24213455
24213455
24213457
24213457
24213458
24213458

24213458
24213458
24213458
24213458
24213458
24213458
24213459
24213460
24213460
24213460
24213460
24213460
24213460
24213460
24213460
24213460
24213460
24213460
24213460
24213460
24213461
24213461
24213462
24213462
24213462
24213463
24213463
24213464
24213464
24213464
24213464
24213464
24213464
24213464
24213464
24213464
24213464
24213464
24213464
24213464
24213464
24213464
24213465
24213465
24213465
24213465
24213465
24213465

24213465
24213465
24213465
24213465
24213465
24213465
24213465
24213465
24213465
24213465
24213465
24213465
24213465
24213465
24213465
24213465
24213466
24213466
24213466
24213466
24213476
24213476
24213476
24213476
24213476
24213476
24213476
24213476
24213476
24213476
24213476
24213476
24213478
24213478
24213478
24213478
24213478
24213478
24213478
24213478
24213478
24213485
24213485
24213485
24213485
24213485
24213485
24213488

24213490
24213490
24213490
24213490
24213490
24213490
24213490
24213490
24213490
24213490
24213490
24213490
24213490
24213491
24213491
24213492
24213492
24213492
24213498
24213499
24213499
24213499
24213499
24213499
24213499
24213500
24213500
24213500
24213500
24213500
24213503
24213509
24213509
24213509
24213509
24213509
24213509
24213509
24213509
24213509
24213509
24213509
24213509
24213510
24213510
24213510
24213523
24213523

24213523
24213525
24213525
24213527
24213527
24213527
24213527
24213528
24213528
24213528
24213528
24213528
24213528
24213528
24213528
24213528
24213528
24213528
24213528
24213528
24213528
24213528
24213528
24213533
24213533
24213533
24213533
24213538
24213538
24213539
24213539
24213539
24213539
24213539
24213540
24213540
24213541
24213545
24213545
24213545
24213545
24213547
24213547
24213549
24213549
24213549
24213549
24213549

24213551
24213551
24213551
24213552
24213552
24213552
24213553
24213553
24213553
24213553
24213553
24213553
24213553
24213554
24213554
24213554
24213554
24213554
24213554
24213554
24213554
24213554
24213554
24213554
24213555
24213555
24213562
24213562
24213562
24213562
24213573
24213573
24213578
24213578
24213579
24213579
24213588
24213589
24213590
24213590
24213590
24213590
24213590
24213590
24213590
24213590
24213590
24213590

24213592
24213592
24213592
24213592
24213592
24213592
24213593
24213593
24213593
24213593
24213593
24213593
24213593
24213599
24213599
24213599
24213599
24213599
24213600
24213600
24213600
24213603
24213603
24213603
24213608
24213609
24213609
24213609
24213609
24213612
24213612
24213612
24213612
24213612
24213612
24213612
24213612
24213613
24213613
24213613
24213613
24213613
24213613
24213613
24213613
24213613
24213618
24213618

24213618
24213618
24213618
24213618
24213618
24213618
24213629
24213629
24213629
24213640
24213640
24213640
24213640
24213640
24213640
24213640
24213642
24213642
24213642
24213642
24213642
24213642
24213642
24213642
24213642
24213647
24213647
24213647
24213647
24213647
24213647
24213647
24213647
24213647
24213647
24213647
24213647
24213647
24213647
24213647
24213647
24213647
24213647
24213647
24213647
24213651
24213651
24213654

24213654
24213654
24213654
24213654
24213654
24213656
24213656
24213656
24213656
24213656
24213656
24213656
24213656
24213656
24213656
24213656
24213656
24213656
24213656
24213656
24213656
24213656
24213656
24213656
24213656
24213656
24213656
24213656
24213656
24213656
24213656
24213656
24213656
24213656
24213656
24213656
24213656
24213656
24213656
24213656
24213656
24213656
24213657
24213657
24213657
24213664
24213665
24213666

24213669
24213669
24213669
24213669
24213669
24213669
24213669
24213680
24213680
24213680
24213694
24213694
24213699
24213707
24213707
24213708
24213719
24213721
24213721
24213721
24213721
24213721
24213721
24213721
24213721
24213721
24213721
24213721
24213721
24213721
24213721
24213721
24213721
24213721
24213721
24213721
24213722
24213722
24213722
24213732
24213732
24213732
24213732
24213732
24213732
24213732
24213732
24213732

24213736
24213736
24213736
24213736
24213736
24213736
24213744
24213744
24213744
24213744
24213744
24213745
24213745
24213745
24213745
24213745
24213747
24213747
24213747
24213747
24213747
24213747
24213747
24213747
24213747
24213747
24213747
24213747
24213752
24213752
24213752
24213752
24213752
24213753
24213753
24213780
24213780
24213780
24213782
24213786
24213787
24213787
24213787
24213787
24213788
24213792
24213792
24213792

24213797
24213799
24213799
24213799
24213799
24213799
24213799
24213801
24213801
24213801
24213801
24213801
24213801
24213801
24213801
24213801
24213801
24213801
24213801
24213801
24213801
24213801
24213801
24213802
24213802
24213802
24213802
24213802
24213802
24213802
24213802
24213802
24213802
24213802
24213802
24213802
24213802
24213807
24213807
24213807
24213807
24213807
24213807
24213807
24213810
24213810
24213810
24213810

24213810
24213810
24213811
24213811
24213811
24213811
24213811
24213812
24213812
24213812
24213812
24213812
24213812
24213812
24213812
24213812
24213812
24213812
24213812
24213812
24213812
24213817
24213817
24213817
24213819
24213819
24213819
24213826
24213826
24213826
24213830
24213830
24213830
24213830
24213830
24213830
24213830
24213832
24213832
24213832
24213836
24213836
24213836
24213836
24213837
24213837
24213837
24213837

24213837
24213837
24213837
24213837
24213838
24213838
24213838
24213838
24213840
24213840
24213840
24213840
24213840
24213840
24213840
24213841
24213841
24213841
24213841
24213844
24213847
24213847
24213847
24213847
24213847
24213847
24213847
24213847
24213847
24213855
24213855
24213855
24213855
24213855
24213855
24213855
24213873
24213873
24213873
24213874
24213874
24213874
24213879
24213879
24213881
24213881
24213881
24213881

24213881
24213881
24213884
24213884
24213885
24213885
24213885
24213885
24213889
24213890
24213890
24213890
24213891
24213891
24213891
24213891
24213891
24213891
24213892
24213892
24213892
24213898
24213898
24213898
24213898
24213898
24213898
24213905
24213905
24213906
24213906
24213906
24213907
24213907
24213909
24213914
24213920
24213920
24213920
24213921
24213921
24213922
24213922
24213922
24213922
24213922
24213922
24213922

24213922
24213922
24213923
24213923
24213923
24213923
24213923
24213923
24213923
24213923
24213923
24213923
24213924
24213924
24213924
24213924
24213924
24213924
24213924
24213924
24213924
24213924
24213924
24213924
24213924
24213924
24213924
24213924
24213924
24213924
24213924
24213940
24213940
24213940
24213940
24213940
24213940
24213940
24213940
24213940
24213940
24213940
24213940
24213940
24213940
24213940
24213940
24213942

24213942
24213943
24213943
24213943
24213944
24213944
24213944
24213944
24213945
24213945
24213945
24213945
24213947
24213947
24213951
24213951
24213951
24213951
24213951
24213951
24213951
24213951
24213951
24213951
24213952
24213952
24213953
24213953
24213953
24213953
24213954
24213955
24213955
24213955
24213957
24213957
24213958
24213958
24213958
24213958
24213960
24213960
24213960
24213960
24213960
24213960
24213960
24213960

24213960
24213960
24213960
24213960
24213960
24213960
24213960
24213960
24213960
24213960
24213960
24213960
24213961
24213961
24213961
24213961
24213963
24213964
24213964
24213973
24213980
24213980
24213980
24213980
24213980
24213980
24213980
24213980
24213980
24213993
24213993
24213993
24213993
24214003
24214003
24214013
24214013
24214013
24214013
24214013
24214013
24214013
24214013
24214013
24214013
24214013
24214013
24214013

24214013
24214013
24214013
24214023
24214023
24214023
24214023
24214023
24214023
24214023
24214023
24214023
24214025
24214025
24214025
24214025
24214025
24214025
24214025
24214025
24214025
24214025
24214025
24214026
24214026
24214026
24214026
24214029
24214029
24214029
24214029
24214034
24214036
24214037
24214037
24214048
24214048
24214048
24214048
24214051
24214051
24214051
24214051
24214051
24214059
24214059
24214059
24214078

24214078
24214078
24214078
24214078
24214078
24214078
24214078
24214078
24214078
24214078
24214078
24214078
24214084
24214084
24214084
24214084
24214088
24214088
24214088
24214088
24214088
24214088
24214090
24214090
24214091
24214091
24214092
24214092
24214092
24214092
24214097
24214097
24214097
24214097
24214097
24214097
24214098
24214098
24214098
24214098
24214109
24214109
24214109
24214109
24214109
24214109
24214109
24214109

24214109
24214109
24214109
24214109
24214109
24214109
24214109
24214109
24214109
24214109
24214109
24214109
24214110
24214114
24214116
24214117
24214117
24214117
24214117
24214117
24214117
24214120
24214120
24214120
24214122
24214122
24214122
24214122
24214122
24214122
24214122
24214129
24214137
24214137
24214137
24214138
24214140
24214140
24214140
24214146
24214146
24214146
24214146
24214149
24214149
24214149
24214149
24214158

24214159
24214159
24214159
24214159
24214159
24214159
24214159
24214159
24214159
24214163
24214164
24214164
24214179
24214180
24214180
24214180
24214188
24214188
24214188
24214188
24214188
24214188
24214195
24214195
24214195
24214195
24214195
24214195
24214211
24214211
24214214
24214214
24214216
24214216
24214217
24214218
24214218
24214218
24214218
24214218
24214221
24214221
24214226
24214230
24214230
24214232
24214232
24214232

24214232
24214232
24214232
24214232
24214232
24214232
24214232
24214232
24214232
24214235
24214235
24214243
24214243
24214243
24214243
24214246
24214246
24214253
24214253
24214263
24214263
24214263
24214264
24214277
24214277
24214277
24214278
24214278
24214278
24214278
24214279
24214279
24214279
24214280
24214280
24214280
24214280
24214280
24214280
24214280
24214280
24214280
24214280
24214280
24214280
24214283
24214283
24214283

24214283
24214283
24214283
24214283
24214283
24214283
24214283
24214283
24214287
24214287
24214287
24214287
24214287
24214290
24214290
24214290
24214290
24214290
24214290
24214300
24214300
24214300
24214300
24214303
24214303
24214304
24214304
24214305
24214306
24214306
24214306
24214306
24214307
24214307
24214310
24214310
24214310
24214311
24214311
24214313
24214313
24214313
24214313
24214313
24214313
24214313
24214313
24214313

24214313
24214314
24214314
24214314
24214314
24214314
24214314
24214314
24214316
24214316
24214316
24214316
24214316
24214316
24214316
24214317
24214317
24214317
24214317
24214317
24214317
24214317
24214318
24214319
24214319
24214319
24214319
24214319
24214319
24214319
24214319
24214319
24214319
24214319
24214319
24214319
24214319
24214319
24214319
24214322
24214322
24214324
24214324
24214325
24214325
24214325
24214325
24214326

24214327
24214327
24214327
24214329
24214329
24214329
24214329
24214329
24214329
24214331
24214332
24214332
24214335
24214338
24214343
24214343
24214343
24214343
24214343
24214343
24214343
24214343
24214343
24214343
24214343
24214344
24214344
24214344
24214344
24214344
24214344
24214345
24214345
24214345
24214345
24214345
24214345
24214345
24214345
24214345
24214347
24214347
24214347
24214348
24214350
24214352
24214352
24214352

24214352
24214354
24214354
24214355
24214355
24214355
24214355
24214357
24214357
24214358
24214358
24214359
24214359
24214359
24214359
24214359
24214360
24214362
24214362
24214362
24214362
24214366
24214374
24214374
24214374
24214374
24214374
24214375
24214375
24214375
24214375
24214375
24214375
24214376
24214376
24214377
24214377
24214377
24214378
24214378
24214378
24214378
24214378
24214380
24214380
24214380
24214380
24214380

24214380
24214380
24214380
24214380
24214380
24214380
24214380
24214380
24214380
24214383
24214383
24214389
24214389
24214389
24214390
24214390
24214396
24214396
24214396
24214403
24214403
24214403
24214403
24214403
24214403
24214403
24214403
24214403
24214403
24214403
24214403
24214403
24214403
24214403
24214403
24214403
24214404
24214404
24214404
24214404
24214404
24214404
24214404
24214404
24214404
24214404
24214404
24214404

24214404
24214415
24214415
24214415
24214415
24214415
24214415
24214415
24214415
24214419
24214419
24214419
24214419
24214419
24214419
24214419
24214419
24214419
24214419
24214419
24214419
24214419
24214419
24214419
24214419
24214419
24214419
24214424
24214424
24214425
24214425
24214425
24214425
24214425
24214425
24214425
24214425
24214428
24214428
24214428
24214428
24214428
24214428
24214428
24214428
24214428
24214428
24214433

24214433
24214433
24214434
24214434
24214434
24214440
24214440
24214440
24214441
24214446
24214481
24214493
24214493
24214553
24214553
24214553
24214553
24214557
24214557
24214561
24214563
24214563
24214563
24214563
24214563
24214564
24214564
24214564
24214564
24214564
24214564
24214564
24214565
24214565
24214565
24214565
24214565
24214566
24214566
24214566
24214566
24214566
24214566
24214566
24214567
24214567
24214575
24214575

24214575
24214575
24214579
24214579
24214579
24214579
24214579
24214579
24214579
24214579
24214579
24214579
24214579
24214579
24214579
24214579
24214579
24214579
24214579
24214583
24214583
24214583
24214583
24214583
24214583
24214583
24214583
24214583
24214583
24214583
24214583
24214583
24214583
24214583
24214583
24214583
24214583
24214583
24214586
24214588
24214588
24214588
24214588
24214588
24214588
24214596
24214596
24214596

24214596
24214597
24214597
24214597
24214597
24214597
24214597
24214597
24214597
24214597
24214597
24214597
24214597
24214597
24214598
24214599
24214599
24214599
24214611
24214611
24214611
24214611
24214611
24214611
24214611
24214611
24214611
24214611
24214611
24214611
24214611
24214615
24214615
24214615
24214617
24214620
24214620
24214620
24214620
24214620
24214620
24214624
24214624
24214627
24214627
24214627
24214628
24214628

24214628
24214629
24214629
24214629
24214629
24214629
24214629
24214629
24214629
24214629
24214629
24214629
24214629
24214629
24214629
24214629
24214629
24214629
24214629
24214629
24214629
24214629
24214629
24214630
24214630
24214630
24214630
24214630
24214635
24214635
24214635
24214635
24214636
24214636
24214636
24214636
24214636
24214636
24214636
24214636
24214636
24214639
24214639
24214639
24214646
24214647
24214647
24214647

24214647
24214647
24214647
24214648
24214648
24214651
24214651
24214651
24214651
24214651
24214651
24214651
24214651
24214651
24214657
24214657
24214657
24214676
24214676
24214676
24214676
24214676
24214676
24214676
24214676
24214677
24214677
24214677
24214677
24214678
24214678
24214678
24214679
24214679
24214681
24214681
24214685
24214685
24214686
24214686
24214686
24214686
24214686
24214686
24214686
24214686
24214686
24214686

24214686
24214686
24214688
24214688
24214691
24214691
24214692
24214692
24214692
24214695
24214695
24214695
24214695
24214695
24214695
24214695
24214695
24214696
24214696
24214707
24214707
24214709
24214709
24214709
24214710
24214710
24214710
24214710
24214711
24214717
24214717
24214717
24214717
24214717
24214717
24214717
24214717
24214717
24214717
24214717
24214717
24214717
24214717
24214717
24214719
24214719
24214719
24214719

24214719
24214719
24214724
24214724
24214724
24214724
24214724
24214724
24214724
24214724
24214724
24214724
24214724
24214724
24214724
24214724
24214724
24214724
24214724
24214724
24214724
24214727
24214734
24214742
24214742
24214742
24214745
24214750
24214750
24214750
24214750
24214750
24214753
24214753
24214759
24214759
24214760
24214761
24214762
24214762
24214762
24214762
24214762
24214762
24214762
24214762
24214762
24214762

24214762
24214762
24214762
24214762
24214762
24214762
24214763
24214763
24214763
24214766
24214766
24214766
24214766
24214766
24214766
24214766
24214766
24214766
24214766
24214766
24214766
24214766
24214766
24214771
24214771
24214771
24214771
24214771
24214772
24214772
24214777
24214777
24214777
24214777
24214777
24214777
24214777
24214777
24214778
24214781
24214790
24214790
24214793
24214795
24214795
24214795
24214795
24214795

24214795
24214795
24214795
24214795
24214795
24214796
24214796
24214796
24214796
24214796
24214796
24214796
24214797
24214797
24214797
24214797
24214797
24214797
24214797
24214805
24214805
24214805
24214805
24214807
24214807
24214807
24214807
24214809
24214809
24214812
24214814
24214814
24214815
24214818
24214818
24214819
24214819
24214819
24214819
24214819
24214819
24214821
24214825
24214825
24214825
24214827
24214829
24214829

24214829
24214829
24214829
24214829
24214829
24214829
24214829
24214829
24214829
24214829
24214829
24214830
24214830
24214830
24214830
24214830
24214830
24214830
24214830
24214832
24214832
24214832
24214836
24214838
24214838
24214838
24214838
24214838
24214838
24214838
24214838
24214838
24214838
24214838
24214838
24214838
24214839
24214839
24214839
24214839
24214839
24214839
24214839
24214839
24214839
24214839
24214839
24214839

24214839
24214839
24214839
24214839
24214840
24214840
24214841
24214841
24214842
24214842
24214842
24214842
24214846
24214846
24214846
24214846
24214846
24214846
24214846
24214850
24214851
24214851
24214851
24214851
24214852
24214852
24214852
24214852
24214852
24214852
24214853
24214853
24214853
24214853
24214853
24214853
24214853
24214854
24214854
24214854
24214854
24214862
24214862
24214862
24214864
24214864
24214872
24214872

24214873
24214873
24214877
24214877
24214877
24214878
24214878
24214878
24214878
24214878
24214879
24214879
24214879
24214879
24214879
24214879
24214879
24214879
24214879
24214879
24214879
24214884
24214884
24214884
24214884
24214884
24214886
24214886
24214886
24214886
24214886
24214886
24214886
24214886
24214886
24214893
24214897
24214897
24214897
24214897
24214897
24214897
24214897
24214897
24214897
24214897
24214897
24214897

24214897
24214897
24214902
24214902
24214902
24214902
24214902
24214902
24214902
24214902
24214902
24214902
24214902
24214902
24214902
24214911
24214911
24214911
24214911
24214911
24214911
24214911
24214912
24214912
24214912
24214912
24214912
24214912
24214912
24214912
24214912
24214922
24214922
24214922
24214922
24214922
24214922
24214922
24214922
24214922
24214922
24214923
24214923
24214926
24214926
24214926
24214926
24214926

24214926
24214926
24214926
24214930
24214931
24214931
24214931
24214932
24214932
24214932
24214932
24214932
24214932
24214932
24214932
24214932
24214932
24214932
24214932
24214932
24214936
24214936
24214936
24214936
24214936
24214936
24214936
24214936
24214937
24214944
24214944
24214944
24214944
24214944
24214944
24214944
24214952
24214952
24214955
24214955
24214955
24214955
24214962
24214962
24214962
24214962
24214962
24214962

24214962
24214965
24214965
24214965
24214965
24214965
24214966
24214967
24214968
24214968
24214969
24214977
24214980
24214982
24214983
24214983
24214983
24214983
24214983
24214983
24214983
24214983
24214983
24214986
24214986
24214986
24214986
24214986
24214986
24214986
24214986
24214986
24214991
24214997
24214997
24214997
24214997
24215000
24215000
24215000
24215000
24215001
24215001
24215001
24215001
24215001
24215002
24215002

24215002
24215003
24215003
24215009
24215009
24215009
24215009
24215009
24215009
24215009
24215009
24215009
24215009
24215009
24215009
24215009
24215009
24215009
24215009
24215014
24215015
24215015
24215016
24215016
24215016
24215016
24215019
24215020
24215020
24215020
24215020
24215023
24215023
24215023
24215024
24215024
24215024
24215024
24215024
24215024
24215025
24215025
24215025
24215025
24215025
24215025
24215025
24215025

24215025
24215025
24215025
24215027
24215027
24215027
24215028
24215028
24215029
24215029
24215029
24215029
24215029
24215029
24215035
24215035
24215035
24215036
24215037
24215037
24215037
24215037
24215037
24215039
24215040
24215045
24215045
24215045
24215045
24215045
24215045
24215045
24215046
24215046
24215046
24215046
24215049
24215049
24215049
24215050
24215050
24215050
24215050
24215050
24215052
24215052
24215052
24215052

24215055
24215057
24215057
24215065
24215065
24215066
24215067
24215067
24215067
24215067
24215067
24215067
24215067
24215067
24215072
24215073
24215073
24215074
24215074
24215074
24215074
24215074
24215074
24215076
24215076
24215076
24215076
24215076
24215083
24215083
24215083
24215083
24215083
24215083
24215083
24215083
24215083
24215087
24215087
24215087
24215087
24215087
24215087
24215088
24215088
24215088
24215090
24215090

24215094
24215094
24215094
24215095
24215095
24215095
24215095
24215095
24215095
24215095
24215095
24215098
24215098
24215099
24215099
24215100
24215100
24215103
24215103
24215103
24215104
24215104
24215104
24215104
24215106
24215106
24215110
24215111
24215111
24215111
24215111
24215111
24215113
24215113
24215115
24215118
24215118
24215118
24215118
24215118
24215118
24215118
24215118
24215132
24215132
24215132
24215132
24215132

24215132
24215132
24215132
24215132
24215132
24215132
24215132
24215132
24215132
24215132
24215132
24215132
24215132
24215132
24215132
24215132
24215132
24215132
24215132
24215132
24215132
24215132
24215133
24215133
24215133
24215133
24215133
24215133
24215133
24215133
24215133
24215133
24215133
24215133
24215133
24215133
24215133
24215133
24215134
24215135
24215138
24215138
24215138
24215138
24215138
24215139
24215139
24215139

24215139
24215139
24215139
24215139
24215156
24215159
24215161
24215165
24215166
24215167
24215167
24215167
24215168
24215168
24215168
24215168
24215168
24215168
24215168
24215169
24215169
24215169
24215183
24215183
24215184
24215184
24215194
24215194
24215194
24215206
24215206
24215206
24215206
24215206
24215206
24215206
24215207
24215207
24215207
24215207
24215207
24215207
24215207
24215210
24215210
24215212
24215212
24215212

24215212
24215212
24215212
24215212
24215212
24215212
24215213
24215213
24215213
24215213
24215214
24215215
24215215
24215215
24215215
24215215
24215226
24215226
24215226
24215228
24215228
24215228
24215230
24215235
24215235
24215235
24215235
24215235
24215235
24215236
24215236
24215236
24215236
24215236
24215236
24215236
24215237
24215237
24215239
24215239
24215242
24215242
24215242
24215242
24215242
24215242
24215242
24215244

24215250
24215250
24215250
24215250
24215250
24215250
24215251
24215251
24215258
24215258
24215258
24215258
24215258
24215258
24215258
24215258
24215258
24215259
24215259
24215259
24215259
24215259
24215259
24215259
24215260
24215260
24215260
24215260
24215260
24215260
24215260
24215260
24215260
24215260
24215260
24215260
24215260
24215260
24215260
24215262
24215262
24215262
24215262
24215262
24215262
24215262
24215269
24215269

24215269
24215270
24215270
24215270
24215270
24215270
24215270
24215270
24215270
24215270
24215270
24215272
24215272
24215272
24215272
24215272
24215272
24215272
24215272
24215275
24215275
24215275
24215276
24215276
24215276
24215276
24215276
24215276
24215276
24215276
24215276
24215276
24215276
24215276
24215276
24215276
24215279
24215281
24215284
24215284
24215289
24215290
24215290
24215291
24215291
24215291
24215294
24215296

24215302
24215302
24215303
24215312
24215313
24215313
24215313
24215323
24215323
24215325
24215325
24215327
24215327
24215327
24215327
24215327
24215327
24215327
24215327
24215327
24215327
24215327
24215329
24215332
24215332
24215332
24215332
24215332
24215332
24215337
24215337
24215337
24215337
24215337
24215337
24215337
24215337
24215337
24215337
24215337
24215337
24215337
24215337
24215337
24215337
24215337
24215337
24215339

24215339
24215339
24215343
24215343
24215343
24215343
24215348
24215354
24215354
24215354
24215354
24215354
24215354
24215354
24215354
24215355
24215355
24215355
24215355
24215355
24215355
24215355
24215355
24215355
24215355
24215355
24215355
24215355
24215355
24215355
24215355
24215355
24215355
24215355
24215355
24215355
24215355
24215355
24215355
24215355
24215355
24215355
24215355
24215355
24215355
24215355
24215355
24215355

24215355
24215355
24215355
24215355
24215355
24215355
24215355
24215355
24215355
24215362
24215362
24215362
24215364
24215364
24215364
24215364
24215372
24215372
24215374
24215374
24215374
24215374
24215387
24215387
24215387
24215387
24215387
24215389
24215389
24215389
24215389
24215389
24215389
24215389
24215389
24215391
24215391
24215391
24215391
24215391
24215391
24215391
24215392
24215399
24215400
24215402
24215402
24215402

24215402
24215402
24215402
24215406
24215406
24215406
24215419
24215419
24215419
24215426
24215426
24215433
24215433
24215433
24215433
24215433
24215437
24215437
24215437
24215438
24215438
24215438
24215438
24215438
24215438
24215438
24215438
24215438
24215438
24215438
24215440
24215441
24215448
24215448
24215448
24215448
24215448
24215448
24215449
24215449
24215449
24215455
24215455
24215455
24215459
24215459
24215459
24215459

24215459
24215459
24215459
24215461
24215461
24215461
24215461
24215461
24215461
24215475
24215475
24215475
24215480
24215480
24215480
24215480
24215480
24215480
24215481
24215481
24215488
24215490
24215508
24215508
24215509
24215509
24215509
24215509
24215509
24215509
24215521
24215521
24215521
24215526
24215527
24215529
24215529
24215534
24215534
24215534
24215534
24215534
24215534
24215536
24215536
24215536
24215536
24215536

24215540
24215540
24215540
24215547
24215547
24215547
24215547
24215548
24215548
24215548
24215548
24215548
24215559
24215562
24215562
24215568
24215568
24215568
24215568
24215568
24215568
24215571
24215577
24215594
24215594
24215594
24215603
24215603
24215603
24215603
24215605
24215605
24215605
24215616
24215616
24215616
24215617
24215617
24215617
24215617
24215617
24215617
24215617
24215620
24215620
24215628
24215628
24215640

24215648
24215648
24215648
24215648
24215648
24215649
24215649
24215650
24215650
24215650
24215654
24215654
24215654
24215655
24215655
24215655
24215655
24215655
24215658
24215658
24215658
24215658
24215658
24215658
24215658
24215658
24215658
24215666
24215666
24215667
24215667
24215667
24215667
24215667
24215667
24215667
24215667
24215672
24215672
24215685
24215687
24215687
24215689
24215689
24215689
24215691
24215691
24215694

24215695
24215698
24215698
24215698
24215701
24215701
24215701
24215701
24215701
24215701
24215701
24215702
24215702
24215703
24215703
24215703
24215703
24215727
24215727
24215727
24215727
24215727
24215727
24215727
24215727
24215727
24215732
24215732
24215732
24215738
24215738
24215738
24215738
24215740
24215740
24215750
24215751
24215751
24215751
24215751
24215751
24215751
24215753
24215754
24215768
24215768
24215768
24215769

24215769
24215769
24215769
24215770
24215770
24215778
24215778
24215779
24215779
24215779
24215779
24215779
24215779
24215779
24215779
24215779
24215785
24215785
24215785
24215785
24215785
24215793
24215793
24215795
24215795
24215795
24215795
24215795
24215795
24215796
24215796
24215796
24215796
24215796
24215796
24215796
24215796
24215796
24215796
24215796
24215796
24215796
24215796
24215796
24215796
24215796
24215796
24215796

24215812
24215812
24215812
24215817
24215817
24215817
24215817
24215817
24215817
24215817
24215817
24215818
24215818
24215818
24215818
24215819
24215819
24215819
24215834
24215834
24215834
24215834
24215834
24215834
24215834
24215834
24215834
24215834
24215834
24215834
24215842
24215842
24215842
24215842
24215842
24215842
24215843
24215843
24215843
24215843
24215843
24215857
24215859
24215859
24215859
24215859
24215859
24215859

24215859
24215865
24215871
24215871
24215872
24215890
24215891
24215891
24215896
24215903
24215909
24215913
24215913
24215915
24215915
24215954
24215961
24215961
24215961
24215961
24215962
24215962
24215962
24215962
24215962
24215962
24215962
24215962
24215963
24215963
24215963
24215963
24215963
24215963
24215964
24215964
24215964
24215964
24215964
24215964
24215964
24215966
24215966
24215966
24215966
24215966
24215966
24215966

24215966
24215966
24215966
24215966
24215966
24215966
24215966
24215966
24215966
24215966
24215966
24215966
24215966
24215966
24215966
24215966
24215966
24215966
24215966
24215966
24215966
24215967
24215967
24215967
24215984
24215984
24215985
24215985
24215985
24215985
24215985
24215985
24215985
24215985
24215985
24215987
24215987
24215987
24215987
24215988
24215988
24215988
24215988
24215988
24215988
24215988
24215989
24215989

24215989
24215989
24215989
24215989
24215989
24215990
24215990
24215990
24215990
24215990
24215990
24215992
24215993
24215993
24215994
24215994
24215994
24215994
24215994
24215995
24215995
24215995
24215995
24215996
24215996
24215996
24215996
24215999
24215999
24216000
24216000
24216002
24216002
24216002
24216002
24216004
24216004
24216004
24216004
24216004
24216004
24216004
24216005
24216005
24216005
24216005
24216005
24216011

24216011
24216016
24216019
24216022
24216049
24216049
24216050
24216051
24216054
24216055
24216055
24216055
24216055
24216055
24216055
24216060
24216061
24216061
24216061
24216062
24216062
24216064
24216064
24216064
24216064
24216064
24216069
24216069
24216069
24216069
24216069
24216069
24216069
24216069
24216069
24216071
24216071
24216071
24216071
24216071
24216079
24216079
24216079
24216079
24216079
24216079
24216079
24216098

24216098
24216098
24216098
24216099
24216099
24216099
24216099
24216099
24216099
24216099
24216099
24216100
24216100
24216100
24216100
24216100
24216100
24216100
24216100
24216100
24216100
24216102
24216102
24216105
24216105
24216105
24216106
24216106
24216106
24216106
24216106
24216106
24216106
24216106
24216106
24216106
24216106
24216108
24216108
24216108
24216109
24216109
24216109
24216109
24216109
24216109
24216109
24216109

24216115
24216115
24216115
24216115
24216115
24216115
24216115
24216116
24216118
24216118
24216118
24216118
24216118
24216118
24216118
24216118
24216118
24216118
24216118
24216118
24216118
24216118
24216118
24216118
24216118
24216118
24216118
24216118
24216118
24216118
24216118
24216118
24216118
24216118
24216118
24216118
24216118
24216118
24216118
24216118
24216118
24216118
24216118
24216118
24216118
24216118
24216118
24216118

24216118
24216118
24216118
24216118
24216118
24216118
24216118
24216118
24216118
24216118
24216118
24216118
24216118
24216118
24216118
24216119
24216119
24216119
24216119
24216119
24216119
24216119
24216119
24216119
24216119
24216119
24216119
24216119
24216119
24216119
24216119
24216119
24216119
24216119
24216119
24216119
24216119
24216119
24216119
24216119
24216119
24216119
24216119
24216119
24216119
24216119
24216119
24216119

24216119
24216119
24216119
24216119
24216119
24216119
24216119
24216119
24216119
24216119
24216119
24216119
24216120
24216120
24216120
24216120
24216120
24216120
24216120
24216121
24216121
24216121
24216121
24216121
24216121
24216121
24216121
24216122
24216122
24216122
24216122
24216122
24216122
24216122
24216122
24216123
24216123
24216123
24216123
24216124
24216124
24216124
24216124
24216124
24216124
24216124
24216134
24216134

24216134
24216140
24216140
24216141
24216141
24216141
24216152
24216152
24216152
24216154
24216159
24216159
24216159
24216159
24216159
24216159
24216159
24216159
24216159
24216159
24216159
24216159
24216159
24216159
24216159
24216159
24216167
24216167
24216167
24216167
24216167
24216167
24216169
24216169
24216170
24216170
24216184
24216184
24216184
24216184
24216196
24216196
24216196
24216196
24216196
24216210
24216210
24216212

24216212
24216212
24216212
24216213
24216213
24216221
24216221
24216221
24216221
24216222
24216223
24216223
24216231
24216231
24216231
24216231
24216231
24216232
24216232
24216234
24216234
24216234
24216234
24216238
24216238
24216256
24216256
24216262
24216262
24216262
24216270
24216270
24216270
24216274
24216274
24216275
24216278
24216278
24216280
24216280
24216281
24216295
24216295
24216295
24216295
24216295
24216295
24216295

24216295
24216295
24216296
24216296
24216296
24216296
24216296
24216296
24216296
24216305
24216305
24216305
24216305
24216306
24216306
24216306
24216306
24216306
24216306
24216312
24216312
24216323
24216324
24216324
24216324
24216324
24216324
24216324
24216326
24216326
24216326
24216326
24216326
24216326
24216326
24216326
24216326
24216327
24216327
24216327
24216327
24216327
24216327
24216330
24216331
24216331
24216335
24216335

24216337
24216337
24216337
24216337
24216337
24216337
24216337
24216338
24216343
24216343
24216343
24216343
24216343
24216356
24216356
24216356
24216356
24216356
24216356
24216356
24216360
24216371
24216371
24216371
24216371
24216371
24216375
24216375
24216375
24216383
24216383
24216383
24216383
24216383
24216383
24216383
24216383
24216383
24216383
24216391
24216391
24216391
24216391
24216403
24216403
24216403
24216403
24216404

24216404
24216404
24216404
24216404
24216404
24216404
24216404
24216404
24216404
24216404
24216404
24216404
24216404
24216404
24216404
24216404
24216404
24216406
24216406
24216406
24216406
24216406
24216406
24216406
24216408
24216408
24216408
24216408
24216408
24216408
24216408
24216408
24216408
24216408
24216409
24216409
24216409
24216409
24216409
24216411
24216411
24216411
24216439
24216443
24216443
24216443
24216446
24216446

24216446
24216446
24216446
24216446
24216446
24216446
24216446
24216446
24216446
24216446
24216446
24216446
24216446
24216447
24216447
24216447
24216447
24216447
24216447
24216447
24216447
24216447
24216447
24216447
24216447
24216447
24216448
24216448
24216448
24216448
24216450
24216451
24216451
24216451
24216453
24216453
24216453
24216453
24216457
24216457
24216461
24216461
24216461
24216461
24216461
24216461
24216464
24216464

24216466
24216466
24216468
24216468
24216481
24216481
24216482
24216482
24216482
24216482
24216482
24216482
24216482
24216482
24216487
24216487
24216494
24216494
24216494
24216495
24216498
24216499
24216499
24216499
24216500
24216500
24216500
24216500
24216500
24216500
24216500
24216500
24216500
24216500
24216500
24216502
24216502
24216502
24216502
24216502
24216502
24216506
24216506
24216506
24216515
24216515
24216515
24216517

24216517
24216518
24216518
24216518
24216518
24216518
24216518
24216518
24216518
24216518
24216518
24216518
24216518
24216518
24216518
24216518
24216519
24216519
24216519
24216519
24216519
24216520
24216520
24216520
24216520
24216520
24216520
24216521
24216521
24216521
24216521
24216521
24216521
24216522
24216522
24216522
24216522
24216522
24216522
24216522
24216532
24216532
24216537
24216538
24216538
24216538
24216538
24216545

24216545
24216545
24216545
24216545
24216545
24216545
24216545
24216545
24216545
24216545
24216545
24216545
24216545
24216545
24216545
24216545
24216545
24216545
24216545
24216548
24216560
24216560
24216568
24216568
24216568
24216568
24216568
24216568
24216569
24216569
24216569
24216569
24216569
24216569
24216569
24216569
24216569
24216571
24216571
24216571
24216571
24216573
24216573
24216573
24216587
24216587
24216587
24216587

24216587
24216587
24216591
24216591
24216592
24216592
24216593
24216593
24216593
24216593
24216596
24216602
24216602
24216602
24216602
24216609
24216609
24216611
24216611
24216611
24216611
24216611
24216611
24216611
24216612
24216612
24216616
24216616
24216617
24216617
24216617
24216617
24216617
24216617
24216617
24216617
24216617
24216621
24216621
24216621
24216623
24216623
24216623
24216623
24216628
24216628
24216633
24216635

24216635
24216635
24216636
24216636
24216637
24216637
24216637
24216637
24216637
24216637
24216637
24216638
24216638
24216638
24216638
24216638
24216638
24216644
24216646
24216646
24216646
24216646
24216647
24216647
24216647
24216647
24216648
24216648
24216648
24216648
24216649
24216649
24216656
24216656
24216656
24216656
24216656
24216656
24216656
24216656
24216656
24216657
24216657
24216657
24216657
24216658
24216659
24216663

24216663
24216663
24216663
24216663
24216663
24216663
24216663
24216663
24216663
24216663
24216663
24216668
24216668
24216668
24216671
24216671
24216671
24216676
24216676
24216676
24216677
24216681
24216681
24216681
24216681
24216681
24216681
24216681
24216681
24216681
24216681
24216681
24216696
24216697
24216697
24216697
24216697
24216697
24216697
24216697
24216698
24216698
24216707
24216707
24216707
24216707
24216707
24216726

24216734
24216734
24216734
24216734
24216734
24216734
24216734
24216734
24216734
24216734
24216734
24216734
24216734
24216734
24216734
24216734
24216734
24216739
24216739
24216739
24216739
24216745
24216751
24216751
24216757
24216757
24216764
24216766
24216766
24216766
24216766
24216766
24216766
24216766
24216766
24216766
24216766
24216766
24216766
24216766
24216766
24216766
24216766
24216766
24216766
24216766
24216766
24216767

24216778
24216778
24216778
24216778
24216778
24216778
24216784
24216784
24216784
24216784
24216784
24216784
24216785
24216785
24216786
24216792
24216792
24216796
24216799
24216799
24216802
24216802
24216806
24216808
24216808
24216808
24216808
24216808
24216808
24216808
24216813
24216813
24216814
24216816
24216818
24216818
24216818
24216818
24216818
24216818
24216818
24216818
24216818
24216818
24216830
24216830
24216830
24216830

24216830
24216830
24216836
24216837
24216837
24216837
24216837
24216837
24216837
24216837
24216837
24216837
24216837
24216837
24216837
24216837
24216837
24216837
24216837
24216838
24216838
24216838
24216838
24216838
24216838
24216838
24216838
24216838
24216838
24216849
24216849
24216849
24216850
24216850
24216850
24216852
24216852
24216855
24216855
24216855
24216855
24216855
24216855
24216855
24216858
24216858
24216858
24216858

24216858
24216858
24216858
24216858
24216858
24216858
24216858
24216858
24216858
24216859
24216865
24216865
24216865
24216865
24216865
24216865
24216865
24216865
24216865
24216865
24216865
24216865
24216865
24216865
24216865
24216865
24216866
24216866
24216866
24216867
24216867
24216867
24216867
24216867
24216867
24216867
24216870
24216870
24216870
24216870
24216870
24216870
24216870
24216870
24216870
24216870
24216877
24216877

24216881
24216889
24216889
24216889
24216889
24216889
24216889
24216889
24216889
24216889
24216889
24216889
24216889
24216889
24216889
24216892
24216892
24216892
24216892
24216892
24216892
24216892
24216892
24216892
24216893
24216893
24216893
24216893
24216893
24216893
24216893
24216893
24216893
24216893
24216893
24216893
24216893
24216893
24216893
24216899
24216899
24216899
24216899
24216899
24216907
24216907
24216916
24216918

24216918
24216918
24216918
24216918
24216919
24216919
24216919
24216919
24216919
24216919
24216919
24216920
24216935
24216938
24216938
24216938
24216938
24216938
24216938
24216942
24216942
24216947
24216947
24216947
24216947
24216947
24216947
24216947
24216947
24216948
24216948
24216953
24216953
24216953
24216953
24216953
24216953
24216953
24216954
24216954
24216954
24216956
24216956
24216957
24216965
24216965
24216965
24216965

24216965
24216971
24216971
24216971
24216971
24216971
24216971
24216971
24216973
24216976
24216976
24216976
24216976
24216976
24216976
24216976
24216977
24216977
24216977
24216987
24216990
24216992
24216992
24216992
24216994
24216994
24216995
24216998
24216998
24216999
24216999
24216999
24217006
24217008
24217013
24217013
24217013
24217031
24217034
24217034
24217035
24217038
24217038
24217038
24217038
24217038
24217039
24217040

24217040
24217040
24217045
24217045
24217045
24217045
24217045
24217045
24217047
24217047
24217047
24217048
24217048
24217048
24217048
24217048
24217054
24217054
24217054
24217054
24217058
24217058
24217060
24217060
24217060
45655587
45655587
45655588
45655588
45655588
45655592
45655592
45655593
45655593
45655593
45655593
45655593
45655594
45655594
45655594
45655594
45655594
45655594
45655594
45655596
45655597
45655598
45655598

45655598
45655605
45655605
45655605
45655609
45655609
45655611
45655611
45655611
45655611
45655611
45655613
45655615
45655617
45655617
45655618
45655618
45655618
45655618
45655618
45655618
45655618
45655619
45655620
45655620
45655620
45655620
45655620
45655620
45655627
45655627
45655627
45655627
45655627
45655627
45655628
45655631
45655631
45655632
45655635
45655640
45655641
45655641
45655641
45655641
45655641
45655641
45655641

45655641
45655644
45655645
45655645
45655645
45655647
45655647
45655647
45655648
45655648
45655648
45655661
45655661
45655661
45655662
45655663
45655663
45655666
45655666
45655666
45655666
45655666
45655666
45655666
45655666
45655666
45655666
45655668
45655668
45655668
45655668
45655668
45655668
45655668
45655668
45655668
45655668
45655668
45655668
45655668
45655668
45655668
45655668
45655668
45655671
45655671
45655672
45655676

45655676
45655677
45655679
45655679
45655679
45655680
45655680
45655682
45655683
45655685
45655685
45655685
45655691
45655691
45655691
45655691
45655697
45655698
45655718
45655720
45655720
45655721
45655722
45655723
45655723
45655724
45655724
45655724
45655725
45655725
45655726
45655726
45655728
45655735
45655740
45655742
45655743
45655744
45655744
45655747
45655747
45655749
45655749
45655752
45655754
45655757
45655757
45655757

45655757
45655767
45655767
45655767
45655767
45655767
45655767
45655767
45655767
45655768
45655772
45655772
45655772
45655775
45655775
45655775
45655776
45655776
45655777
45655777
45655777
45655777
45655777
45655777
45655777
45655777
45655777
45655777
45655779
45655779
45655779
45655784
45655784
45655784
45655785
45655785
45655785
45655785
45655785
45655788
45655788
45655788
45655788
45655788
45655788
45655788
45655789
45655789

45655789
45655789
45655789
45655789
45655789
45655789
45655794
45655794
45655796
45655796
45655796
45655803
45655805
45655805
45655805
45655807
45655807
45655807
45655807
45655807
45655808
45655814
45655814
45655814
45655815
45655816
45655817
45655817
45655817
45655817
45655817
45655817
45655817
45655817
45655818
45655818
45655818
45655818
45655819
45655819
45655819
45655819
45655819
45655820
45655822
45655822
45655822
45655822

45655824
45655824
45655824
45655824
45655824
45655824
45655824
45655824
45655824
45655824
45655824
45655824
45655824
45655824
45655824
45655824
45655825
45655829
45655829
45655829
45655829
45655829
45655829
45655831
45655832
45655837
45655839
45655839
45655839
45655839
45655839
45655840
45655841
45655841
45655841
45655841
45655841
45655841
45655847
45655847
45655847
45655847
45655847
45655848
45655849
45655849
45655849
45655850

45655850
45655850
45656005
45656005
45656025
45656028
45656028
45656048
45656121
45656195
45656218
45656218
45656241
45656243
45656252
45656328
45656328
45656419
45656444
45656463
45656479
45656481
45656484
45656487
45656504
45656516
45656524
45656538
45656541
45656590
45656610
45656617
45656617
45656620
45656650
45656664
45656690
45656715
45656719
45656719
45656802
45656847
45656861
45656893
45656903
45656920
45656921
45656935

45656959
45656960
45656995
45657021
45657061
45657067
45657099
45657102
45657125
45657127
45657127
45657150
45657182
45657193
45657198
45657213
45657218
45657233
45657237
45657237
45657247
45657247
45657259
45657267
45657285
45657299
45657309
45657313
45657343
45657345
45657390
45657391
45657391
45657399
45657411
45657419
45657515
45657518
45657520
45657523
45657553
45657595
45657612
45657612
45657634
45657635
45657656
45657668

45657673
45657673
45657687
45657688
45657717
45657743
45657769
45657814
45657824
45657830
45657855
45657884
45657930
45657939
45657939
45657957
45657972
45657985
45658000
45658012
45658015
45658015
45658015
45658043
45658043
45658044
45658044
45658044
45658044
45658044
45658044
45658044
45658044
45658045
45658045
45658045
45658045
45658045
45658046
45658046
45658046
45658046
45658046
45658046
45658047
45658047
45658047
45658047

45658047
45658047
45658047
45658047
45658047
45658047
45658048
45658073
45658092
45658112
45658140
45658165
45658178
45658187
45658223
45658223
45658246
45658248
45658253
45658254
45658258
45658278
45658305
45658318
45658390
45658398
45658407
45658476
45658482
45658486
45658489
45658521
45658529
45658529
45658529
45658529
45658535
45658535
45658536
45658536
45658565
45658571
45658603
45658604
45658604
45658618
45658626
45658626

45658659
45658683
45658689
45658696
45658699
45658705
45658729
45658729
45658752
45658782
45658797
45658801
45658807
45658849
45658900
45658907
45658909
45658936
45658941
45659008
45659032
45659041
45659041
45659042
45659066
45659069
45659083
45659096
45659103
45659114
45659118
45659120
45659135
45659137
45659147
45659156
45659186
45659194
45659230
45659237
45659268
45659287
45659297
45659308
45659308
45659308
45659308
45659308

45659308
45659308
45659308
45659308
45659308
45659308
161484946
161484946
161484946
161484946
161484946
161484946
161484946
161484946
161484946
161484946
161484946
161484947
161484947
161484947
161484947
161484947
161484947
161484947
161484947
161484947
161484947
161484947
161484948
161484948
161484948
161484948
161484948
161484948
161484948
161484948
161484948
161484948
161484948
161484948
161484948
161484948
161484948
161484948
161484948
161484948
161484948
161484948

161484948
161484948
161484948
161484948
161484952
161484952
161484952
161484952
161484952
161484952
161484952
161484952
161484953
161484953
161484953
161484953
161484953
161484953
161484953
161484953
161484954
161484954
161484954
161484954
161484957
161484957
161484957
161484957
161484957
161484957
161621776
229020021
229020021
229020021
229020021
229020021
229020021
229020021
229020021
294827557
294827557
294827557
294827557
294827557
294827558
294827562
294827566
294827566

294827567
294827567
294827567
294827570
294827570
294827570
294827570
294827570
294827572
294827572
294827574
294827574
294827577
294827577
294827577
294827577
294827577
294827577
294827577
294827577
294827577
294827577
294827577
294827581
294827581
294827581
294827581
294827581
294827581
294827583
294827583
294827583
294827583
294827584
294827584
294827584
294827584
294827584
294827584
294827584
294827584
294827584
294827584
294827584
294827584
294827584
294827584
294827588

294827588
294827588
294827588
294827588
294827589
294827589
294827589
294827589
294827592
294827592
294827598
294827600
294827600
294827600
294827600
294827600
294827600
294827600
294827600
294827600
294827601
294827601
294827601
294827601
294827601
294827601
294827602
294827603
294827603
294827603
294827605
294827605
294827606
294827606
294827606
294827606
294827608
294827608
294827608
294827608
294827608
294827608
294827608
294827608
294827612
294827612
294827612
294827613

294827613
294827613
294827613
294827613
294827613
294827613
294827613
294827613
294827613
294827613
294827613
294827613
294827620
294827621
294827621
294827624
294827624
294827624
294827627
294827627
294827627
294827627
294827627
294827627
294827627
294827627
294827627
294827627
294827627
294827630
294827630
294827630
294827630
294827630
294827630
294827630
294827630
294827632
294827632
294827632
294827632
294827632
294827632
294827634
294827634
294827634
294827634
294827634

294827634
294827635
294827635
294827635
294827635
294827635
294827635
294827635
294827639
294827639
294827639
294827643
294827643
294827652
294827652
294827652
294827652
294827652
294827652
294827652
294827652
294827652
294827652
294827654
294827654
294827657
294827658
294827660
294827662
294827662
294827662
294827670
294827670
294827670
294827670
294827670
294827670
294827670
294827670
294827670
294827670
294827670
294827670
294827677
294827680
294827680
294827680
294827680

294827680
294827680
294827680
294827680
294827685
294827685
294827685
294827685
294827685
294827685
294827685
294827685
294827686
294827690
294827690
294827690
294827690
294827690
294827690
294827690
294827690
294827690
294827690
294827690
294827690
294827690
294827690
294827690
294827690
294827690
294827690
294827690
294827690
294827690
294827690
294827690
294827690
294827690
294827690
294827690
294827690
294827690
294827690
294827690
294827690
294827692
294827692
294827692

294827692
294827692
294827693
294827694
294827695
294827697
294827699
294827699
294827702
294827702
294827702
294827702
294827703
294827703
294827703
294827703
294827704
294827709
294827709
294827709
294827709
294827709
294827714
294827719
294827726
294827726
294827727
294827727
294827727
294827727
294827727
294827727
294827727
294827727
294827727
294827731
294827733
294827733
294827739
294827739
294827741
294827741
294827742
294827742
294827742
294827742
294827742
294827744

294827744
294827747
294827748
294827748
294827748
294827748
294827748
294827748
294827748
294827748
294827752
294827752
294827762
294827762
294827764
294827765
294827765
294827765
294827765
294827765
294827765
294827765
294827765
294827765
294827765
294827765
294827765
294827765
294827765
294827765
294827765
294827765
294827765
294827765
294827765
294827765
294827765
294827766
294827766
294827766
294827769
294827769
294827769
294827770
294827770
294827771
294827787
294827788

294827791
294827792
294827792
294827792
294827792
294827793
294827799
294827800
294827800
294827800
294827801
294827801
294827801
294827801
294827801
294827801
294827801
294827801
294827801
294827801
294827801
294827801
294827801
294827802
294827802
294827804
294827804
294827805
294827805
294827805
294827812
294827812
294827815
294827819
294827823
294827823
294827823
294827823
294827823
294827827
294827827
294827827
294827827
294827827
294827827
294827827
294827829
294827829

294827829
294827829
294827830
294827830
294827830
294827830
294827834
294827834
294827834
294827834
294827834
294827834
294827834
294827834
294827834
294827834
294827834
294827834
294827836
294827838
294827841
294827842
294827842
294827842
294827842
294827842
294827842
294827842
294827843
294827848
294827848
294827848
294827854
294827854
294827855
294827856
294827856
294827856
294827858
294827858
294827859
294827859
294827859
294827865
294827865
294827865
294827865
294827865

294827865
294827866
294827866
294827866
294827866
294827866
294827866
294827868
294827868
294827875
294827875
294827881
294827885
294827888
294827890
294827890
294827896
294827896
294827896
294827896
294827896
294827896
294827898
294827898
294827898
294827898
294827898
294827898
294827898
294827898
294827898
294827898
294827898
294827898
294827898
294827898
294827898
294827899
294827899
294827900
294827905
294827905
294827905
294827905
294827905
294827905
294827905
294827907

294827907
294827907
294827907
294827912
294827912
294827912
294827913
294827913
294827913
294827913
294827913
294827917
294827920
294827920
294827920
294827920
294827922
294827922
294827924
294827924
294827924
294827924
294827928
294827929
294827929
294827929
294827929
294827929
294827929
294827932
294827932
294827932
294827932
294827933
294827940
294827940
294827940
294827965
294827965
294827970
294827970
294827975
294827985
294827985
294827987
294827987
294827987
294827988

294827988
294827988
294827988
294827988
294827988
294827988
294827988
294827988
294827988
294827988
294827988
294827988
294827988
294827988
294827988
294827988
294827988
294827988
294827988
294827988
294827992
294827993
294827993
294827993
294827993
294827993
294827993
294827993
294827994
294827994
294827994
294827994
294827997
294827997
294827997
294827997
294827997
294827998
294828003
294828003
294828003
294828003
294828003
294828003
294828003
294828003
294828004
294828005

294828005
294828006
294828006
294828008
294828008
294828008
294828009
294828009
294828009
294828010
294828010
294828010
294828010
294828013
294828013
294828013
294828013
294828020
294828022
294828022
294828022
294828023
294828023
294828023
294828026
294828029
294828029
294828030
294828030
294828030
294828030
294828034
294828035
294828035
294828035
294828041
294828041
294828041
294828041
294828045
294828046
294828046
294828046
294828046
294828046
294828046
294828046
294828047

294828050
294828050
294828050
294828050
294828050
294828053
294828053
294828056
294828056
294828058
294828058
294828058
294828058
294828059
294828059
294828059
294828059
294828059
294828059
294828063
294828063
294828063
294828065
294828068
294828068
294828068
294828071
294828071
294828072
294828074
294828075
294828077
294828077
294828077
294828077
294828077
294828077
294828077
294828077
294828077
294828077
294828077
294828077
294828077
294828077
294828077
294828077
294828077

294828080
294828080
294828080
294828080
294828080
294828082
294828082
294828082
294828082
294828082
294828082
294828082
294828082
294828084
294828084
294828084
294828084
294828087
294828087
294828087
294828087
294828088
294828088
294828088
294828088
294828088
294828088
294828088
294828088
294828088
294828088
294828088
294828088
294828088
294828088
294828088
294828088
294828088
294828092
294828092
294828092
294828092
294828092
294828092
294828092
294828094
294828096
294828097

294828097
294828098
294828098
294828098
294828098
294828098
294828099
294828099
294828099
294828100
294828100
294828100
294828100
294828100
294828100
294828101
294828101
294828101
294828101
294828101
294828101
294828101
294828101
294828101
294828103
294828103
294828103
294828103
294828103
294828105
294828105
294828105
294828106
294828110
294828110
294828111
294828116
294828116
294828116
294828116
294828116
294828117
294828117
294828117
294828117
294828118
294828118
294828123

294828123
294828123
294828123
294828123
294828123
294828123
294828123
294828125
294828125
294828126
294828126
294828130
294828130
294828130
294828130
294828134
294828134
294828134
294828134
294828134
294828134
294828134
294828134
294828134
294828136
294828136
294828137
294828143
294828143
294828143
294828144
294828144
294828144
294828144
294828144
294828144
294828144
294828144
294828147
294828147
294828147
294828147
294828147
294828147
294828147
294828147
294828147
294828147

294828147
294828147
294828147
294828147
294828147
294828149
294828152
294828152
294828152
294828152
294828153
294828153
294828159
294828159
294828160
294828160
294828160
294828162
294828162
294828162
294828162
294828162
294828162
294828162
294828162
294828162
294828162
294828162
294828162
294828162
294828162
294828167
294828167
294828167
294828170
294828170
294828171
294828172
294828172
294828172
294828174
294828174
294828175
294828177
294828177
294828177
294828177
294828180

294828180
294828180
294828180
294828180
294828180
294828180
294828180
294828180
294828180
294828180
294828180
294828180
294828180
294828180
294828180
294828180
294828180
294828180
294828180
294828181
294828182
294828182
294828187
294828187
294828187
294828188
294828188
294828188
294828188
294828188
294828188
294828192
294828193
294828193
294828193
294828193
294828193
294828193
294828193
294828193
294828193
294828193
294828193
294828193
294828195
294828195
294828195
294828195

294828205
294828212
294828212
294828212
294828212
294828212
294828212
294828215
294828215
294828215
294828215
294828215
294828216
294828216
294828218
294828218
294828218
294828218
294828218
294828218
294828218
294828218
294828218
294828218
294828222
294828222
294828226
294828227
294828227
294828235
294828235
294828235
294828235
294828235
294828235
294828235
294828235
294828235
294828235
294828235
294828235
294828236
294828236
294828237
294828237
294828237
294828237
294828237

294828247
294828247
294828247
294828247
294828247
294828247
294828247
294828247
294828247
294828247
294828249
294828249
294828249
294828254
294828258
294828258
294828258
294828258
294828258
294828258
294828265
294828265
294828269
294828269
294828269
294828269
294828269
294828269
294828269
294828269
294828269
294828279
294828279
294828279
294828279
294828279
294828279
294828279
294828285
294828285
294828285
294828286
294828286
294828286
294828286
294828286
294828286
294828286

294828286
294828286
294828286
294828286
294828286
294828286
294828286
294828289
294828297
294828297
294828297
294828298
294828298
294828298
294828301
294828301
294828301
294828301
294828301
294828301
294828301
294828301
294828301
294828301
294828301
294828301
294828301
294828301
294828301
294828301
294828301
294828301
294828301
294828301
294828301
294828301
294828301
294828301
294828301
294828301
294828301
294828301
294828301
294828301
294828301
294828301
294828301
294828301

294828301
294828301
294828301
294828301
294828301
294828301
294828301
294828301
294828301
294828301
294828301
294828301
294828302
294828302
294828302
294828302
294828302
294828302
294828309
294828309
294828309
294828309
294828310
294828310
294828310
294828311
294828311
294828311
294828311
294828311
294828315
294828315
294828315
294828319
294828319
294828319
294828320
294828320
294828320
294828327
294828327
294828327
294828327
294828327
294828327
294828327
294828327
294828329

294828334
294828338
294828339
294828339
294828339
294828339
294828340
294828340
294828347
294828347
294828347
294828347
294828347
294828347
294828348
294828348
294828351
294828352
294828352
294828352
294828354
294828354
294828362
294828362
294828363
294828363
294828363
294828368
294828368
294828368
294828369
294828369
294828369
294828371
294828371
294828371
294828372
294828375
294828375
294828376
294828376
294828378
294828378
294828378
294828390
294828390
294828390
294828390

294828390
294828390
294828402
294828402
294828402
294828402
294828411
294828411
294828411
294828411
294828411
294828411
294828411
294828415
294828415
294828415
294828422
294828422
294828422
294828426
294828426
294828426
294828426
294828426
294828427
294828427
294828427
294828432
294828432
294828432
294828432
294828432
294828432
294828432
294828432
294828432
294828432
294828432
294828432
294828432
294828432
294828432
294828432
294828432
294828432
294828432
294828433
294828436

294828436
294828439
294828439
294828439
294828446
294828446
294828446
294828446
294828446
294828448
294828449
294828449
294828449
294828452
294828452
294828452
294828452
294828452
294828452
294828452
294828452
294828452
294828454
294828454
294828460
294828460
294828460
294828460
294828460
294828460
294828461
294828461
294828463
294828463
294828463
294828463
294828463
294828464
294828464
294828464
294828464
294828464
294828464
294828465
294828465
294828465
294828465
294828465

294828465
294828469
294828469
294828474
294828474
294828475
294828475
294828475
294828475
294828475
294828475
294828475
294828475
294828475
294828475
294828475
294828475
294828475
294828475
294828475
294828486
294828487
294828487
294828487
294828487
294828487
294828487
294828487
294828487
294828491
294828493
294828493
294828493
294828493
294828493
294828493
294828493
294828493
294828493
294828493
294828493
294828493
294828493
294828493
294828498
294828498
294828498
294828498

294828498
294828498
294828498
294828498
294828498
294828498
294828499
294828499
294828504
294828511
294828512
294828513
294828513
294828513
294828513
294828513
294828514
294828514
294828514
294828514
294828514
294828514
294828514
294828514
294828514
294828514
294828514
294828514
294828515
294828515
294828515
294828515
294828515
294828515
294828515
294828515
294828518
294828518
294828518
294828518
294828518
294828518
386072525
386072526
386072526
386072534
386072534
386072534

386072538
386072548
386072548
386072548
386072548
386072553
386072580
386072591
386072591
386072599
386072599
386072601
386072601
386072608
386072609
386072609
386072609
386072609
386072610
386072610
386072610
386072610
386072617
386072623
386072626
386072626
386072630
386072636
386072636
386072688
386072688
386072694
386072694
386072708
386072711
386072711
386072713
386072713
386072741
386072756
386072764
386072764
386072767
386072774
386072775
386072775
386072799
386072809

386072811
386072832
386072839
386072839
386072851
386072851
386072855
386072873
386072875
386072875
386072875
386072875
386072879
386072879
386072879
386072879
386072889
386072924
386072928
386072930
386072930
386072954
386072954
386072954
386072954
386072981
386072981
386072988
386072988
386073010
386073024
386073024
386073024
386073025
386073025
386073025
386073073
386073080
386073081
386073081
386073081
386073082
386073085
386073089
386073089
386073089
386073090
386073092

386073092
386073094
386073096
386073107
386073114
386073121
386073121
386073135
386073144
386073164
386073185
386073185
386073190
386073190
386073198
386073239
386073240
386073240
386073243
386073249
386073260
386073318
386073318
386073359
386073359
386073359
386073370
386073370
386073393
386073393
386073393
386073393
386073399
386073399
386073400
386073408
386073408
386073435
386073447
386073447
386073447
386073447
386073447
386073458
386073458
386073460
386073461
386073477

386073478
386073478
386073478
386073482
386073482
386073482
386073482
386073491
386073492
386073497
386073501
386073501
386073525
386073529
386073529
386073532
386073534
386073550
386073550
386073562
386073573
386073576
386073576
386073585
386073596
386073596
386073596
386073599
386073636
386073636
386073636
386073667
386073676
386073676
386073680
386073680
386073690
386073690
386073707
386073707
386073707
386073720
386073752
386073752
386073765
386073765
386073812
386073825

386073825
386073854
386073860
386073863
386073867
386073867
386073868
386073868
386073868
386073979
386073981
386073981
386073981
386073981
386073981
386073981
386073981
386073981
386073992
386073992
386073992
386073992
386073994
386074013
386074015
386074016
386074022
386074033
386074039
386074046
386074046
386074052
386074060
386074060
386074060
386074061
386074061
386074065
386074069
386074075
386074100
386074101
386074105
386074109
386074109
386074120
386074123
386074136

386074139
386074140
386074144
386074146
386074147
386074147
386074170
386074183
386074199
386074199
386074199
386074205
386074207
386074212
386074233
386074233
386074233
386074233
386074235
386074235
386074235
386074235
386074264
386074264
386074264
386074265
386074268
386074269
386074273
386074273
386074273
386074273
386074282
386074282
386074282
386074283
386074283
386074283
386074283
386074289
386074290
386074298
386074305
386074305
386074305
386074305
386074323
386074334

386074334
386074334
386074339
386074346
386074346
386074346
386074360
386074362
386074362
386074375
386074375
386074393
386074393
386074398
386074398
386074401
386074401
386074412
386074418
386074439
386074460
386074460
386074460
386074460
386074472
386074474
386074475
386074475
386074484
386074484
386074484
386074504
386074511
386074516
386074516
386074529
386074529
386074533
386074533
386074534
386074534
386074556
386074564
386074564
386074564
386074564
386074564
386074571

386074576
386074576
386074602
386074614
386074614
386074614
386074614
386074621
386074621
386074628
386074635
386074635
386074671
386074671
386074671
386074671
386074690
386074690
386074695
386074705
386074705
386074706
386074706
386074706
386074706
386074714
386074718
386074721
386074721
386074744
386074750
386074818
386074859
386074888
386074926
386074928
386074938
386074938
386074938
386074939
386074939
386074947
386074948
386074948
386074948
386074948
386074966
386074982

386074982
386074982
386074994
386074994
386074994
386075054
386075054
386075054
386075056
386075057
386075058
386075059
386075059
386075060
386075073
386075073
386075073
386075076
386075078
386075078
386075078
386075089
386075128
386075128
386075128
386075140
386075140
386075144
386075144
386075144
386075144
386075144
386075144
386075167
386075172
386075173
386075176
386075178
386075188
386075211
386075235
386075237
386075237
386075237
386075260
386075291
386075291
386075291

386075304
386075323
386075323
386075331
386075337
386075337
386075337
386075353
386075360
386075361
386075365
386075383
386075383
386075395
386075397
386075397
386075397
386075406
386075406
386075407
386075407
386075408
386075412
386075442
386075464
386075469
386075473
386075485
386075487
386075487
386075487
386075489
386075493
386075507
386075507
386075523
386075523
386075523
386075526
386075526
386075562
386075562
386075594
386075594
386075610
386075629
386075629
386075629

| |
|---|
| 386075642 |
| 386075675 |
| 386075675 |
| 386075675 |
| 386075675 |
| 386075681 |
| 386075682 |
| 386075682 |
| 386075699 |
| 386075725 |
| 386075739 |
| 386075739 |
| 386075739 |
| 386075741 |
| 386075741 |
| 386075758 |
| 386075758 |
| 386075765 |
| 386075771 |
| 386075772 |
| 386075772 |
| 386075772 |
| 386075811 |
| 386075819 |
| 386075823 |
| 386075823 |
| 386075836 |
| 386075857 |
| 386075901 |

ed *Leptospira* peptides identified in *Leptospira interrogans* serovar Canicola

| **Protein identification** |
|---|
| chromosomal replication initiation protein A |
| chromosomal replication initiation protein A |
| DNA polymerase III subunit beta |
| DNA polymerase III subunit beta |
| DNA polymerase III subunit beta |
| DNA polymerase III subunit beta |
| DNA polymerase III subunit beta |
| DNA polymerase III subunit beta |
| DNA polymerase III subunit beta |
| DNA polymerase III subunit beta |
| DNA polymerase III subunit beta |
| DNA polymerase III subunit beta |
| recombination protein RecF |
| DNA gyrase subunit B |
| DNA gyrase subunit B |
| DNA gyrase subunit B |
| DNA gyrase subunit B |
| DNA gyrase subunit B |
| DNA gyrase subunit B |
| DNA gyrase subunit B |
| DNA gyrase subunit B |
| DNA gyrase subunit B |
| DNA gyrase subunit B |
| DNA gyrase subunit B |
| DNA gyrase subunit B |
| DNA gyrase subunit B |
| DNA gyrase subunit B |
| DNA gyrase subunit B |
| DNA gyrase subunit B |
| DNA gyrase subunit B |
| DNA gyrase subunit B |
| DNA gyrase subunit A |
| DNA gyrase subunit A |
| DNA gyrase subunit A |
| DNA gyrase subunit A |
| DNA gyrase subunit A |
| DNA gyrase subunit A |
| DNA gyrase subunit A |
| DNA gyrase subunit A |
| DNA gyrase subunit A |
| DNA gyrase subunit A |
| DNA gyrase subunit A |
| DNA gyrase subunit A |
| DNA gyrase subunit A |
| hypothetical protein LA_0009 |
| hypothetical protein LA_0009 |

hypothetical protein LA_0009
hypothetical protein LA_0009
hypothetical protein LA_0009
hypothetical protein LA_0009
hypothetical protein LA_0009
hypothetical protein LA_0009
outer membrane lipoprotein LipL21
outer membrane lipoprotein LipL21
DNA-directed DNA polymerase subunit delta
short chain dehydrogenase
short chain dehydrogenase
short chain dehydrogenase
short chain dehydrogenase
short chain dehydrogenase
short chain dehydrogenase
short chain dehydrogenase
short chain dehydrogenase
translation factor Sua5
translation factor Sua5
translation factor Sua5
hypothetical protein LA_0031
hypothetical protein LA_0031
hypothetical protein LA_0031
hypothetical protein LA_0031
hypothetical protein LA_0031
hypothetical protein LA_0031
hypothetical protein LA_0031
hypothetical protein LA_0032
inositol monophosphatase family protein
inositol monophosphatase family protein
inositol monophosphatase family protein
inositol monophosphatase family protein
inositol monophosphatase family protein
inositol monophosphatase family protein
inositol monophosphatase family protein
beta-galactosidase
HSP33-family chaperone
HSP33-family chaperone
ribonuclease R
ribonuclease R
ribonuclease R
glucose-methanol-choline oxidoreductase
glucose-methanol-choline oxidoreductase
TPR-repeat-containing protein
TPR-repeat-containing protein
TPR-repeat-containing protein
TPR-repeat-containing protein
TPR-repeat-containing protein

3-dehydroquinate dehydratase
methyl-accepting chemotaxis protein
methyl-accepting chemotaxis protein
nucleoside-diphosphate-sugar epimerase
nucleoside-diphosphate-sugar epimerase
nucleoside-diphosphate-sugar epimerase
nucleoside-diphosphate-sugar epimerase
nucleoside-diphosphate-sugar epimerase
nucleoside-diphosphate-sugar epimerase
nucleoside-diphosphate-sugar epimerase
nucleoside-diphosphate-sugar epimerase
nucleoside-diphosphate-sugar epimerase
nucleoside-diphosphate-sugar epimerase
nucleoside-diphosphate-sugar epimerase
NAD(P)(+) transhydrogenase subunit alpha
NAD(P)(+) transhydrogenase subunit alpha
NAD(P)(+) transhydrogenase subunit alpha
NAD(P)(+) transhydrogenase subunit alpha
NAD(P)(+) transhydrogenase subunit alpha
NAD(P)(+) transhydrogenase subunit alpha
NAD(P)(+) transhydrogenase subunit alpha
NAD(P)(+) transhydrogenase subunit alpha
NAD(P)(+) transhydrogenase subunit alpha
NAD(P)(+) transhydrogenase subunit alpha
NAD(P)(+) transhydrogenase subunit alpha
NAD(P)(+) transhydrogenase subunit alpha
NAD(P)(+) transhydrogenase subunit alpha
NAD(P)(+) transhydrogenase subunit alpha
NAD(P)(+) transhydrogenase subunit alpha
NAD(P)(+) transhydrogenase subunit alpha
NAD(P)(+) transhydrogenase subunit alpha
NAD(P)(+) transhydrogenase subunit alpha
NAD(P)(+) transhydrogenase subunit alpha
NAD(P)(+) transhydrogenase subunit alpha
histidyl-tRNA synthetase
histidyl-tRNA synthetase
histidyl-tRNA synthetase
histidyl-tRNA synthetase
histidyl-tRNA synthetase
OmpA family protein
OmpA family protein
OmpA family protein
RlpA-like lipoprotein
RlpA-like lipoprotein
serine acetyltransferase
serine acetyltransferase
serine acetyltransferase
hypothetical protein LA_0067

hypothetical protein LA_0067
hypothetical protein LA_0067
hypothetical protein LA_0067
deoxycytidine triphosphate deaminase
deoxycytidine triphosphate deaminase
deoxycytidine triphosphate deaminase
deoxycytidine triphosphate deaminase
deoxycytidine triphosphate deaminase
enoyl-CoA hydratase
enoyl-CoA hydratase
enoyl-CoA hydratase
enoyl-CoA hydratase
enoyl-CoA hydratase
hydrolase
SAM-dependent methyltransferase
SAM-dependent methyltransferase
SAM-dependent methyltransferase
diaminopimelate epimerase
diaminopimelate epimerase
diaminopimelate epimerase
diaminopimelate epimerase
diaminopimelate epimerase
diaminopimelate epimerase
diaminopimelate epimerase
diaminopimelate epimerase
diaminopimelate epimerase
diaminopimelate epimerase
diaminopimelate epimerase
hypothetical protein LA_0085
hypothetical protein LA_0093
tRNA (uracil-5-)-methyltransferase
hypothetical protein LA_0102
hypothetical protein LA_0107
hypothetical protein LA_0107
hypothetical protein LA_0107
leucyl/phenylalanyl-tRNA--protein transferase
para-aminobenzoate synthase component I
cystathionine gamma-synthase
cystathionine gamma-synthase
cystathionine gamma-synthase
cystathionine gamma-synthase
cystathionine gamma-synthase
cystathionine gamma-synthase
cystathionine gamma-synthase
cystathionine gamma-synthase
peptide chain release factor 1
hypothetical protein LA_0115
hypothetical protein LA_0117

hypothetical protein LA_0117
hypothetical protein LA_0117
hypothetical protein LA_0117
hypothetical protein LA_0117
hypothetical protein LA_0117
nudix hydrolase
imidazoleglycerol-phosphate dehydratase/histidinol-phosphatase
imidazoleglycerol-phosphate dehydratase/histidinol-phosphatase
imidazoleglycerol-phosphate dehydratase/histidinol-phosphatase
imidazole glycerol phosphate synthase subunit HisH
phosphoribosylformimino-5-aminoimidazole carboxamide ribotide isomerase
phosphoribosylformimino-5-aminoimidazole carboxamide ribotide isomerase
phosphoribosylformimino-5-aminoimidazole carboxamide ribotide isomerase
phosphoribosylformimino-5-aminoimidazole carboxamide ribotide isomerase
hypothetical protein LA_0127
hypothetical protein LA_0127
hypothetical protein LA_0127
hypothetical protein LA_0127
hypothetical protein LA_0127
phosphomethylpyrimidine kinase protein
phosphomethylpyrimidine kinase protein
phosphomethylpyrimidine kinase protein
phosphomethylpyrimidine kinase protein
ribosome-associated GTPase
ribosome-associated GTPase
hypothetical protein LA_0135
hypothetical protein LA_0135
hypothetical protein LA_0135
hypothetical protein LA_0135
TPR-repeat-containing protein
TPR-repeat-containing protein
TPR-repeat-containing protein
TPR-repeat-containing protein
TPR-repeat-containing protein
TPR-repeat-containing protein
TPR-repeat-containing protein
TPR-repeat-containing protein
TPR-repeat-containing protein
TPR-repeat-containing protein
TPR-repeat-containing protein
TPR-repeat-containing protein
TPR-repeat-containing protein
TPR-repeat-containing protein
TPR-repeat-containing protein
metal-dependent hydrolase of the beta-lactamase superfamily I
metal-dependent hydrolase of the beta-lactamase superfamily I
metal-dependent hydrolase of the beta-lactamase superfamily I
metal-dependent hydrolase of the beta-lactamase superfamily I

metal-dependent hydrolase of the beta-lactamase superfamily I
metal-dependent hydrolase of the beta-lactamase superfamily I
metal-dependent hydrolase of the beta-lactamase superfamily I
ribosome biogenesis GTP-binding protein YsxC
transcriptional regulator
transcriptional regulator
transcriptional regulator
transcriptional regulator
transcriptional regulator
transcriptional regulator
transcriptional regulator
transcriptional regulator
hypothetical protein LA_0147
hypothetical protein LA_0147
hypothetical protein LA_0147
hypothetical protein LA_0147
hypothetical protein LA_0147
hypothetical protein LA_0147
SET domain-containing protein
hypothetical protein LA_0153
hypothetical protein LA_0153
hypothetical protein LA_0153
hypothetical protein LA_0153
hypothetical protein LA_0153
hypothetical protein LA_0153
hypothetical protein LA_0153
hypothetical protein LA_0153
chorismate synthase
chorismate synthase
chorismate synthase
chorismate synthase
chorismate synthase
chorismate synthase
chorismate synthase
chorismate synthase
NADH dehydrogenase (ubiquinone) chain G
NADH dehydrogenase (ubiquinone) chain G
NADH dehydrogenase (ubiquinone) chain G
NADH dehydrogenase (ubiquinone) chain G
NADH dehydrogenase (ubiquinone) chain G
NADH dehydrogenase (ubiquinone) chain G
NADH dehydrogenase (ubiquinone) chain G
NADH dehydrogenase (ubiquinone) chain I
NADH dehydrogenase (ubiquinone) chain I
NADH dehydrogenase (ubiquinone) chain I
NADH dehydrogenase (ubiquinone) chain I
NADH dehydrogenase (ubiquinone) chain I
NADH dehydrogenase (ubiquinone) chain I

hypothetical protein LA_0166
hypothetical protein LA_0166
hypothetical protein LA_0166
hypothetical protein LA_0166
preprotein translocase subunit YidC
preprotein translocase subunit YidC
preprotein translocase subunit YidC
preprotein translocase subunit YidC
preprotein translocase subunit YidC
preprotein translocase subunit YidC
preprotein translocase subunit YidC
preprotein translocase subunit YidC
preprotein translocase subunit YidC
Jag-like RNA-binding protein
Jag-like RNA-binding protein
Jag-like RNA-binding protein
Jag-like RNA-binding protein
Jag-like RNA-binding protein
Jag-like RNA-binding protein
Jag-like RNA-binding protein
hypothetical protein LA_0184
hypothetical protein LA_0184
hypothetical protein LA_0184
hypothetical protein LA_0184
hypothetical protein LA_0184
hypothetical protein LA_0184
hypothetical protein LA_0184
hypothetical protein LA_0184
fumarate hydratase
fumarate hydratase
fumarate hydratase
fumarate hydratase
fumarate hydratase
fumarate hydratase
fumarate hydratase
fumarate hydratase
fumarate hydratase
fumarate hydratase
fumarate hydratase
fumarate hydratase
fumarate hydratase
hypothetical protein LA_0205
hypothetical protein LA_0205
hypothetical protein LA_0205
hypothetical protein LA_0205
hypothetical protein LA_0206
hypothetical protein LA_0218
hypothetical protein LA_0218

hypothetical protein LA_0218
OmpA family lipoprotein
OmpA family lipoprotein
OmpA family lipoprotein
OmpA family lipoprotein
OmpA family lipoprotein
OmpA family lipoprotein
OmpA family lipoprotein
OmpA family lipoprotein
OmpA family lipoprotein
OmpA family lipoprotein
OmpA family lipoprotein
OmpA family lipoprotein
OmpA family lipoprotein
OmpA family lipoprotein
OmpA family lipoprotein
short-chain dehydrogenase
short-chain dehydrogenase
inositol monophosphatase family protein
inositol monophosphatase family protein
inositol monophosphatase family protein
SCO1/SenC/PrrC family protein
cytochrome c oxidase polypeptide I
cytochrome c oxidase polypeptide I
cytochrome c oxidase polypeptide I
hypothetical protein LA_0247
hypothetical protein LA_0247
integral membrane protein with PIN domain
integral membrane protein with PIN domain
integral membrane protein with PIN domain
integral membrane protein with PIN domain
integral membrane protein with PIN domain
integral membrane protein with PIN domain
integral membrane protein with PIN domain
phosphoenolpyruvate carboxykinase
phosphoenolpyruvate carboxykinase
phosphoenolpyruvate carboxykinase
phosphoenolpyruvate carboxykinase
phosphoenolpyruvate carboxykinase
phosphoenolpyruvate carboxykinase
phosphoenolpyruvate carboxykinase
phosphoenolpyruvate carboxykinase
phosphoenolpyruvate carboxykinase
phosphoenolpyruvate carboxykinase
phosphoenolpyruvate carboxykinase
phosphoenolpyruvate carboxykinase
phosphoenolpyruvate carboxykinase
phosphoenolpyruvate carboxykinase

phosphoenolpyruvate carboxykinase
phosphoenolpyruvate carboxykinase
phosphoenolpyruvate carboxykinase
phosphoenolpyruvate carboxykinase
phosphoenolpyruvate carboxykinase
phosphoenolpyruvate carboxykinase
phosphoenolpyruvate carboxykinase
phosphoenolpyruvate carboxykinase
phosphoenolpyruvate carboxykinase
phosphoenolpyruvate carboxykinase
hypothetical protein LA_0253
ParA
ParA
aspartyl/glutamyl-tRNA amidotransferase subunit B
aspartyl/glutamyl-tRNA amidotransferase subunit B
aspartyl/glutamyl-tRNA amidotransferase subunit B
aspartyl/glutamyl-tRNA amidotransferase subunit B
aspartyl/glutamyl-tRNA amidotransferase subunit B
aspartyl/glutamyl-tRNA amidotransferase subunit B
hypothetical protein LA_0263
hypothetical protein LA_0263
hypothetical protein LA_0263
hypothetical protein LA_0263
hypothetical protein LA_0264
hypothetical protein LA_0264
hypothetical protein LA_0271
hypothetical protein LA_0271
hypothetical protein LA_0272
hypothetical protein LA_0272
hypothetical protein LA_0272
hypothetical protein LA_0272
lipoprotein releasing system LolD
lipoprotein releasing system LolD
lipoprotein releasing system LolD
hypothetical protein LA_0276
hypothetical protein LA_0276
hypothetical protein LA_0276
hypothetical protein LA_0278
cAMP-binding protein
cAMP-binding protein
cAMP-binding protein
cAMP-binding protein
cAMP-binding protein
cAMP-binding protein
cAMP-binding protein
cAMP-binding protein
cAMP-binding protein
hypothetical protein LA_0284

hypothetical protein LA_0284
hypothetical protein LA_0284
hypothetical protein LA_0284
hypothetical protein LA_0298
carbon starvation protein A
ADP-ribose pyrophosphatase
ADP-ribose pyrophosphatase
ADP-ribose pyrophosphatase
cAMP-binding protein
cAMP-binding protein
cAMP-binding protein
cAMP-binding protein
regulatory-like cAMP-binding protein
regulatory-like cAMP-binding protein
regulatory-like cAMP-binding protein
regulatory-like cAMP-binding protein
metal-dependent hydrolase
metal-dependent hydrolase
metal-dependent hydrolase
elongation factor G
elongation factor G
elongation factor G
elongation factor G
elongation factor G
elongation factor G
elongation factor G
elongation factor G
elongation factor G
elongation factor G
elongation factor G
elongation factor G
elongation factor G
elongation factor G
elongation factor G
elongation factor G
elongation factor G
elongation factor G
elongation factor G
elongation factor G
elongation factor G
elongation factor G
elongation factor G
elongation factor G
elongation factor G
elongation factor G
elongation factor G
elongation factor G
elongation factor G

elongation factor G
elongation factor G
thioredoxin
thioredoxin
Zn-dependent alcohol dehydrogenase
Zn-dependent alcohol dehydrogenase
Zn-dependent alcohol dehydrogenase
Zn-dependent alcohol dehydrogenase
Zn-dependent alcohol dehydrogenase
penicillin G acylase precursor
penicillin G acylase precursor
6-pyruvoyltetrahydropterin synthase
6-pyruvoyltetrahydropterin synthase
NAD(P)(+) transhydrogenase subunit beta
NAD(P)(+) transhydrogenase subunit beta
NAD(P)(+) transhydrogenase subunit beta
NAD(P)(+) transhydrogenase subunit beta
NAD(P)(+) transhydrogenase subunit beta
NAD(P)(+) transhydrogenase subunit beta
NAD(P)(+) transhydrogenase subunit beta
NAD(P)(+) transhydrogenase subunit beta
NAD(P)(+) transhydrogenase subunit beta
flagellar hook-basal body protein FliE
hypothetical protein LA_0350
hypothetical protein LA_0350
TPR-repeat-containing protein
TPR-repeat-containing protein
TPR-repeat-containing protein
TPR-repeat-containing protein
glycine dehydrogenase
glycine dehydrogenase
glycine dehydrogenase
glycine dehydrogenase
glycine dehydrogenase
glycine dehydrogenase
glycine dehydrogenase
glycine dehydrogenase
glycine dehydrogenase
hypothetical protein LA_0365
hypothetical protein LA_0365
hypothetical protein LA_0365
hypothetical protein LA_0365
hypothetical protein LA_0365
hypothetical protein LA_0365
hypothetical protein LA_0365
hypothetical protein LA_0365
hypothetical protein LA_0365
hypothetical protein LA_0365

hypothetical protein LA_0365
hypothetical protein LA_0365
hypothetical protein LA_0365
hypothetical protein LA_0365
hypothetical protein LA_0365
ribose-5-phosphate isomerase
ribose-5-phosphate isomerase
ribose-5-phosphate isomerase
ribose-5-phosphate isomerase
hypothetical protein LA_0374
hypothetical protein LA_0374
hypothetical protein LA_0375
methyltransferase
ATP-dependent protease ClpA
ATP-dependent protease ClpA
ATP-dependent protease ClpA
ATP-dependent protease ClpA
ATP-dependent protease ClpA
ATP-dependent protease ClpA
ATP-dependent protease ClpA
ATP-dependent protease ClpA
ATP-dependent protease ClpA
ATP-dependent protease ClpA
ATP-dependent protease ClpA
ATP-dependent protease ClpA
ATP-dependent protease ClpA
ATP-dependent protease ClpA
ATP-dependent protease ClpA
ATP-dependent protease ClpA
ATP-dependent protease ClpA
paraoxonase
paraoxonase
phosphohistidine phosphatase
phosphohistidine phosphatase
ABC transporter ATP-binding protein
ABC transporter ATP-binding protein
ABC transporter ATP-binding protein
ABC transporter ATP-binding protein
ABC transporter ATP-binding protein
ABC transporter permease
hypothetical protein LA_0406
tryptophanyl-tRNA synthetase
tryptophanyl-tRNA synthetase
tryptophanyl-tRNA synthetase
tryptophanyl-tRNA synthetase
tryptophanyl-tRNA synthetase
hypothetical protein LA_0409
outer membrane lipoprotein-sorting protein

outer membrane lipoprotein-sorting protein
outer membrane lipoprotein-sorting protein
electron transfer flavoprotein subunit alpha
electron transfer flavoprotein subunit alpha
electron transfer flavoprotein subunit alpha
electron transfer flavoprotein subunit alpha
electron transfer flavoprotein subunit alpha
electron transfer flavoprotein subunit alpha
electron transfer flavoprotein subunit alpha
electron transfer flavoprotein subunit alpha
electron transfer flavoprotein subunit alpha
electron transfer flavoprotein subunit alpha
electron transfer flavoprotein subunit alpha
electron transfer flavoprotein subunit alpha
electron transfer flavoprotein subunit alpha
electron transfer flavoprotein subunit alpha
electron transfer flavoprotein subunit beta
electron transfer flavoprotein subunit beta
electron transfer flavoprotein subunit beta
electron transfer flavoprotein subunit beta
electron transfer flavoprotein subunit beta
electron transfer flavoprotein subunit beta
electron transfer flavoprotein subunit beta
electron transfer flavoprotein subunit beta
isovaleryl-CoA dehydrogenase
isovaleryl-CoA dehydrogenase
isovaleryl-CoA dehydrogenase
isovaleryl-CoA dehydrogenase
isovaleryl-CoA dehydrogenase
isovaleryl-CoA dehydrogenase
isovaleryl-CoA dehydrogenase
isovaleryl-CoA dehydrogenase
isovaleryl-CoA dehydrogenase
isovaleryl-CoA dehydrogenase
SAM-dependent O-methyltransferase
hypothetical protein LA_0419
hypothetical protein LA_0419
transcriptional regulator
hypothetical protein LA_0433
hypothetical protein LA_0433
hypothetical protein LA_0433
acyl-CoA dehydrogenase
acyl-CoA dehydrogenase
acyl-CoA dehydrogenase
acyl-CoA dehydrogenase
acyl-CoA dehydrogenase
acyl-CoA dehydrogenase
acyl-CoA dehydrogenase

acyl-CoA dehydrogenase
acyl-CoA dehydrogenase
acyl-CoA dehydrogenase
acyl-CoA dehydrogenase
acyl-CoA dehydrogenase
acyl-CoA dehydrogenase
hypothetical protein LA_0441
hypothetical protein LA_0441
RNA polymerase sigma subunit
hypothetical protein LA_0447
hypothetical protein LA_0447
hypothetical protein LA_0447
acetyl-CoA acetyltransferase
acetyl-CoA acetyltransferase
acetyl-CoA acetyltransferase
acetyl-CoA acetyltransferase
acetyl-CoA acetyltransferase
acetyl-CoA acetyltransferase
acetyl-CoA acetyltransferase
acetyl-CoA acetyltransferase
acetyl-CoA acetyltransferase
acetyl-CoA acetyltransferase
acetyl-CoA acetyltransferase
acetyl-CoA acetyltransferase
acetyl-CoA acetyltransferase
N-acetylmuramoyl-L-alanine amidase
N-acetylmuramoyl-L-alanine amidase
hypothetical protein LA_0462
hypothetical protein LA_0462
hypothetical protein LA_0462
riboflavin synthase subunit beta
riboflavin synthase subunit beta
riboflavin synthase subunit beta
riboflavin synthase subunit beta
transcription antitermination protein NusB
TPR-repeat-containing protein
TPR-repeat-containing protein
FOG:HEAT repeat protein
isopropylmalate/homocitrate/citramalate synthase
isopropylmalate/homocitrate/citramalate synthase
isopropylmalate/homocitrate/citramalate synthase
isopropylmalate/homocitrate/citramalate synthase
isopropylmalate/homocitrate/citramalate synthase
hypothetical protein LA_0471
hypothetical protein LA_0471
hypothetical protein LA_0471
hypothetical protein LA_0471
hypothetical protein LA_0471

hypothetical protein LA_0471
hypothetical protein LA_0471
hypothetical protein LA_0471
hypothetical protein LA_0471
hypothetical protein LA_0471
DNA helicase UvrD
hypothetical protein LA_0494
hypothetical protein LA_0494
hypothetical protein LA_0494
hypothetical protein LA_0494
hypothetical protein LA_0494
hypothetical protein LA_0494
hypothetical protein LA_0494
flagellar motor protein
flagellar motor protein
hypothetical protein LA_0505
hypothetical protein LA_0505
hypothetical protein LA_0505
hypothetical protein LA_0505
hypothetical protein LA_0505
UDP-3-O-[3-hydroxymyristoyl] glucosamine N-acyltransferase
UDP-3-O-[3-hydroxymyristoyl] glucosamine N-acyltransferase
hypothetical protein LA_0532
pyridoxal phosphate-dependent aminotransferase
pyridoxal phosphate-dependent aminotransferase
pyridoxal phosphate-dependent aminotransferase
hydrolase
transcriptional regulator
transcriptional regulator
transcriptional regulator
hypothetical protein LA_0543
hypothetical protein LA_0544
hypothetical protein LA_0544
hypothetical protein LA_0544
hypothetical protein LA_0546
hypothetical protein LA_0546
hypothetical protein LA_0546
hypothetical protein LA_0546
hypothetical protein LA_0546
hypothetical protein LA_0546
hypothetical protein LA_0546
hypothetical protein LA_0546
hypothetical protein LA_0546
hypothetical protein LA_0546
hypothetical protein LA_0546
hypothetical protein LA_0547
hypothetical protein LA_0547
phosphoribosyl-AMP cyclohydrolase protein

periplasmic solute-binding protein
periplasmic solute-binding protein
periplasmic solute-binding protein
periplasmic solute-binding protein
periplasmic solute-binding protein
periplasmic solute-binding protein
periplasmic solute-binding protein
periplasmic solute-binding protein
acyl-CoA dehydrogenase
acyl-CoA dehydrogenase
acyl-CoA dehydrogenase
acyl-CoA dehydrogenase
acyl-CoA dehydrogenase
acyl-CoA dehydrogenase
acyl-CoA dehydrogenase
acyl-CoA dehydrogenase
acyl-CoA dehydrogenase
acyl-CoA dehydrogenase
acyl-CoA dehydrogenase
acyl-CoA dehydrogenase
acyl-CoA dehydrogenase
acyl-CoA dehydrogenase
acyl-CoA dehydrogenase
acyl-CoA dehydrogenase
acyl-CoA dehydrogenase
acyl-CoA dehydrogenase
acyl-CoA dehydrogenase
acyl-CoA dehydrogenase
acyl-CoA dehydrogenase
short-chain dehydrogenase
fatty acid transport protein
hypothetical protein LA_0575
hypothetical protein LA_0575
glutathione S-transferase-like protein
glutathione S-transferase-like protein
glutathione S-transferase-like protein
glutathione S-transferase-like protein
cell division protein FtsA
cell division protein FtsA
cell division protein FtsA
cell division protein FtsZ
shikimate 5-dehydrogenase
30S ribosomal protein S1
30S ribosomal protein S1
30S ribosomal protein S1
30S ribosomal protein S1
30S ribosomal protein S1
30S ribosomal protein S1

30S ribosomal protein S1
30S ribosomal protein S1
anti-sigma regulatory factor
anti-sigma regulatory factor
anti-sigma regulatory factor
excinuclease ABC subunit B
excinuclease ABC subunit B
excinuclease ABC subunit B
excinuclease ABC subunit B
excinuclease ABC subunit B
excinuclease ABC subunit B
excinuclease ABC subunit B
excinuclease ABC subunit B
mannose-6-phosphate isomerase
mannose-6-phosphate isomerase
mannose-6-phosphate isomerase
mannose-6-phosphate isomerase
mannose-6-phosphate isomerase
metallo-beta-lactamase superfamily protein
metallo-beta-lactamase superfamily protein
citrate synthase
citrate synthase
citrate synthase
citrate synthase
citrate synthase
citrate synthase
citrate synthase
citrate synthase
citrate synthase
citrate synthase
oxidoreductase
oxidoreductase
methyl-accepting chemotaxis protein
methyl-accepting chemotaxis protein
methyl-accepting chemotaxis protein
methyl-accepting chemotaxis protein
aspartate kinase
aspartate kinase
aspartate kinase
aspartate kinase
aspartate kinase
aspartate kinase
aspartate kinase
hypothetical protein LA_0699
molybdate metabolism regulator
molybdate metabolism regulator
hypothetical protein LA_0720
hypothetical protein LA_0720

carbamoyl phosphate synthase large subunit
carbamoyl phosphate synthase large subunit
carbamoyl phosphate synthase large subunit
carbamoyl phosphate synthase large subunit
carbamoyl phosphate synthase large subunit
carbamoyl phosphate synthase large subunit
carbamoyl phosphate synthase large subunit
carbamoyl phosphate synthase large subunit
carbamoyl phosphate synthase large subunit
carbamoyl phosphate synthase large subunit
carbamoyl phosphate synthase large subunit
carbamoyl phosphate synthase large subunit
carbamoyl phosphate synthase large subunit
carbamoyl phosphate synthase large subunit
carbamoyl phosphate synthase large subunit
carbamoyl phosphate synthase large subunit
carbamoyl phosphate synthase large subunit
carbamoyl phosphate synthase large subunit
carbamoyl phosphate synthase large subunit
peroxiredoxin-like protein
peroxiredoxin-like protein
elongation factor G
elongation factor G
elongation factor G
elongation factor Tu
elongation factor Tu
elongation factor Tu
elongation factor Tu
elongation factor Tu
elongation factor Tu
elongation factor Tu
elongation factor Tu
elongation factor Tu
elongation factor Tu
elongation factor Tu
elongation factor Tu
elongation factor Tu
elongation factor Tu
elongation factor Tu
elongation factor Tu
elongation factor Tu
elongation factor Tu
elongation factor Tu
elongation factor Tu
elongation factor Tu
elongation factor Tu
elongation factor Tu
elongation factor Tu

elongation factor Tu
elongation factor Tu
elongation factor Tu
elongation factor Tu
30S ribosomal protein S10
30S ribosomal protein S10
30S ribosomal protein S10
30S ribosomal protein S10
50S ribosomal protein L3
50S ribosomal protein L3
50S ribosomal protein L3
50S ribosomal protein L4
50S ribosomal protein L4
50S ribosomal protein L4
50S ribosomal protein L4
50S ribosomal protein L4
50S ribosomal protein L4
50S ribosomal protein L4
50S ribosomal protein L4
50S ribosomal protein L4
50S ribosomal protein L23
50S ribosomal protein L23
50S ribosomal protein L2
50S ribosomal protein L2
50S ribosomal protein L2
50S ribosomal protein L2
50S ribosomal protein L2
50S ribosomal protein L2
50S ribosomal protein L2
50S ribosomal protein L2
50S ribosomal protein L2
50S ribosomal protein L2
50S ribosomal protein L2
50S ribosomal protein L2
50S ribosomal protein L2
50S ribosomal protein L2
50S ribosomal protein L2
50S ribosomal protein L2
50S ribosomal protein L22
50S ribosomal protein L22
50S ribosomal protein L22
50S ribosomal protein L22
50S ribosomal protein L22
50S ribosomal protein L22
30S ribosomal protein S3
30S ribosomal protein S3
30S ribosomal protein S3
30S ribosomal protein S3

30S ribosomal protein S3
30S ribosomal protein S3
30S ribosomal protein S3
30S ribosomal protein S3
30S ribosomal protein S3
50S ribosomal protein L16
50S ribosomal protein L16
50S ribosomal protein L16
50S ribosomal protein L16
50S ribosomal protein L29
50S ribosomal protein L29
50S ribosomal protein L14
50S ribosomal protein L14
50S ribosomal protein L24
50S ribosomal protein L24
50S ribosomal protein L24
50S ribosomal protein L5
50S ribosomal protein L5
50S ribosomal protein L5
50S ribosomal protein L5
50S ribosomal protein L5
50S ribosomal protein L5
50S ribosomal protein L5
50S ribosomal protein L5
50S ribosomal protein L5
50S ribosomal protein L5
30S ribosomal protein S14
30S ribosomal protein S14
30S ribosomal protein S14
30S ribosomal protein S8
30S ribosomal protein S8
30S ribosomal protein S8
30S ribosomal protein S8
30S ribosomal protein S8
30S ribosomal protein S8
50S ribosomal protein L6
50S ribosomal protein L6
50S ribosomal protein L6
50S ribosomal protein L6
50S ribosomal protein L6
50S ribosomal protein L6
50S ribosomal protein L6
50S ribosomal protein L18
50S ribosomal protein L18
50S ribosomal protein L30
50S ribosomal protein L30
50S ribosomal protein L15
50S ribosomal protein L15

50S ribosomal protein L15
50S ribosomal protein L15
50S ribosomal protein L15
50S ribosomal protein L15
50S ribosomal protein L15
50S ribosomal protein L15
preprotein translocase subunit SecY
adenylate kinase
adenylate kinase
adenylate kinase
adenylate kinase
adenylate kinase
adenylate kinase
adenylate kinase
adenylate kinase
adenylate kinase
adenylate kinase
adenylate kinase
adenylate kinase
adenylate kinase
translation initiation factor IF-1
translation initiation factor IF-1
30S ribosomal protein S13
30S ribosomal protein S13
30S ribosomal protein S13
30S ribosomal protein S11
30S ribosomal protein S11
30S ribosomal protein S4
30S ribosomal protein S4
30S ribosomal protein S4
30S ribosomal protein S4
30S ribosomal protein S4
30S ribosomal protein S4
30S ribosomal protein S4
30S ribosomal protein S4
30S ribosomal protein S4
30S ribosomal protein S4
30S ribosomal protein S4
30S ribosomal protein S4
30S ribosomal protein S4
30S ribosomal protein S4
30S ribosomal protein S4
RNA polymerase subunit alpha
RNA polymerase subunit alpha
RNA polymerase subunit alpha
RNA polymerase subunit alpha
RNA polymerase subunit alpha
RNA polymerase subunit alpha

RNA polymerase subunit alpha
RNA polymerase subunit alpha
RNA polymerase subunit alpha
RNA polymerase subunit alpha
RNA polymerase subunit alpha
RNA polymerase subunit alpha
RNA polymerase subunit alpha
RNA polymerase subunit alpha
RNA polymerase subunit alpha
RNA polymerase subunit alpha
RNA polymerase subunit alpha
RNA polymerase subunit alpha
RNA polymerase subunit alpha
RNA polymerase subunit alpha
RNA polymerase subunit alpha
RNA polymerase subunit alpha
50S ribosomal protein L17
50S ribosomal protein L17
50S ribosomal protein L17
50S ribosomal protein L17
L,L-diaminopimelate aminotransferase
L,L-diaminopimelate aminotransferase
L,L-diaminopimelate aminotransferase
L,L-diaminopimelate aminotransferase
L,L-diaminopimelate aminotransferase
L,L-diaminopimelate aminotransferase
L,L-diaminopimelate aminotransferase
L,L-diaminopimelate aminotransferase
L,L-diaminopimelate aminotransferase
L,L-diaminopimelate aminotransferase
L,L-diaminopimelate aminotransferase
L,L-diaminopimelate aminotransferase
aspartate carbamoyltransferase catalytic subunit
aspartate carbamoyltransferase catalytic subunit
aspartate carbamoyltransferase catalytic subunit
aspartate carbamoyltransferase catalytic subunit
aspartate carbamoyltransferase catalytic subunit
aspartate carbamoyltransferase catalytic subunit
aspartate carbamoyltransferase catalytic subunit
aspartate carbamoyltransferase catalytic subunit
aspartate carbamoyltransferase catalytic subunit
Zn-dependent hydrolase
Zn-dependent hydrolase
Zn-dependent hydrolase
Zn-dependent hydrolase
Zn-dependent hydrolase
Zn-dependent hydrolase
SAM-dependent methyltransferase

citrate synthase
citrate synthase
citrate synthase
citrate synthase
citrate synthase
citrate synthase
citrate synthase
citrate synthase
citrate synthase
citrate synthase
citrate synthase
citrate synthase
citrate synthase
transglycosylase
transglycosylase
hypothetical protein LA_0792
hypothetical protein LA_0792
hypothetical protein LA_0792
hypothetical protein LA_0798
cell shape determination protein
cell shape determination protein
cell shape determination protein
cell shape determination protein
cell shape determination protein
cell shape determination protein
pyridoxal phosphate biosynthesis protein
pyridoxal phosphate biosynthesis protein
pyridoxal phosphate biosynthesis protein
pyridoxal phosphate biosynthesis protein
pyridoxal phosphate biosynthesis protein
hypothetical protein LA_0803
trypsin-like serine protease
trypsin-like serine protease
trypsin-like serine protease
trypsin-like serine protease
trypsin-like serine protease
trypsin-like serine protease
trypsin-like serine protease
trypsin-like serine protease
trypsin-like serine protease
trypsin-like serine protease
trypsin-like serine protease
trypsin-like serine protease
Holliday junction DNA helicase B
Holliday junction DNA helicase B
Holliday junction DNA helicase B
alpha/beta hydrolase-like protein
alpha/beta hydrolase-like protein

alpha/beta hydrolase-like protein
transcriptional regulator
transcriptional regulator
thioesterase
thioesterase
thioesterase
thioesterase
acetyl-CoA acetyltransferase
acetyl-CoA acetyltransferase
acetyl-CoA acetyltransferase
acetyl-CoA acetyltransferase
acetyl-CoA acetyltransferase
acetyl-CoA acetyltransferase
acetyl-CoA acetyltransferase
acetyl-CoA acetyltransferase
acetyl-CoA acetyltransferase
acetyl-CoA acetyltransferase
acetyl-CoA acetyltransferase
acetyl-CoA acetyltransferase
acetyl-CoA acetyltransferase
acetyl-CoA acetyltransferase
acetyl-CoA acetyltransferase
acetyl-CoA acetyltransferase
pantothenate kinase
pantothenate kinase
pantothenate kinase
pantothenate kinase
UDP-N-acetylmuramoylalanine--D-glutamate ligase
UDP-N-acetylmuramoylalanine--D-glutamate ligase
anti-sigma factor antagonist
anti-sigma factor antagonist
anti-sigma factor antagonist
anti-sigma factor antagonist
anti-sigma factor antagonist
indole-3-glycerol-phosphate synthase
indole-3-glycerol-phosphate synthase
ribosomal RNA large subunit methyltransferase N
2-isopropylmalate synthase
2-isopropylmalate synthase
2-isopropylmalate synthase
2-isopropylmalate synthase
3-demethylubiquinone-9 3-methyltransferase
3-demethylubiquinone-9 3-methyltransferase
50S ribosomal protein L21
50S ribosomal protein L21
50S ribosomal protein L21
50S ribosomal protein L21
50S ribosomal protein L21

50S ribosomal protein L27
50S ribosomal protein L27
50S ribosomal protein L27
GTPase ObgE
GTPase ObgE
GTPase ObgE
gamma-glutamyl kinase
gamma-glutamyl kinase
gamma-glutamyl kinase
gamma-glutamyl kinase
gamma-glutamyl kinase
gamma-glutamyl kinase
gamma-glutamyl kinase
gamma-glutamyl phosphate reductase
gamma-glutamyl phosphate reductase
gamma-glutamyl phosphate reductase
gamma-glutamyl phosphate reductase
gamma-glutamyl phosphate reductase
gamma-glutamyl phosphate reductase
gamma-glutamyl phosphate reductase
gamma-glutamyl phosphate reductase
gamma-glutamyl phosphate reductase
gamma-glutamyl phosphate reductase
gamma-glutamyl phosphate reductase
nicotinate-nucleotide adenylyltransferase
nicotinate-nucleotide adenylyltransferase
thiol peroxidase
thiol peroxidase
thiol peroxidase
thiol peroxidase
cytoplasmic membrane protein
cytoplasmic membrane protein
hypothetical protein LA_0878
hypothetical protein LA_0878
hypothetical protein LA_0879
hypothetical protein LA_0879
NADH dehydrogenase (ubiquinone) chain J
NADH dehydrogenase (ubiquinone) chain H
NADH dehydrogenase (ubiquinone) chain F
NADH dehydrogenase (ubiquinone) chain F
NADH dehydrogenase (ubiquinone) chain F
NADH dehydrogenase (ubiquinone) chain F
NADH dehydrogenase (ubiquinone) chain F
NADH dehydrogenase (ubiquinone) chain F
NADH dehydrogenase (ubiquinone) chain F
NADH dehydrogenase (ubiquinone) chain F
NADH dehydrogenase (ubiquinone) chain F
NADH dehydrogenase (ubiquinone) chain F

NADH dehydrogenase (ubiquinone) chain D
NADH dehydrogenase (ubiquinone) chain D
NADH dehydrogenase (ubiquinone) chain D
NADH dehydrogenase (ubiquinone) chain D
NADH dehydrogenase (ubiquinone) chain D
NADH dehydrogenase (ubiquinone) chain D
NADH dehydrogenase (ubiquinone) chain C
NADH dehydrogenase (ubiquinone) chain C
NADH dehydrogenase (ubiquinone) chain C
NADH dehydrogenase (ubiquinone) chain C
NADH dehydrogenase (ubiquinone) chain C
NADH dehydrogenase (ubiquinone) chain C
NADH dehydrogenase (ubiquinone) chain C
MaoC-like acyl dehydratase
MaoC-like acyl dehydratase
MaoC-like acyl dehydratase
MaoC-like acyl dehydratase
MaoC-like acyl dehydratase
MarR family transcriptional regulator
MarR family transcriptional regulator
MarR family transcriptional regulator
methylase
methylase
methylase
hypothetical protein LA_0908
methylglyoxal synthase
methylglyoxal synthase
methylglyoxal synthase
methylglyoxal synthase
TPR-repeat-containing protein
TPR-repeat-containing protein
TPR-repeat-containing protein
TPR-repeat-containing protein
TPR-repeat-containing protein
TPR-repeat-containing protein
TPR-repeat-containing protein
TPR-repeat-containing protein
NHL repeat protein
NHL repeat protein
NHL repeat protein
NHL repeat protein
NHL repeat protein
NHL repeat protein
NHL repeat protein
NHL repeat protein
NHL repeat protein
enoyl-CoA hydratase
enoyl-CoA hydratase

enoyl-CoA hydratase
enoyl-CoA hydratase
enoyl-CoA hydratase
enoyl-CoA hydratase
enoyl-CoA hydratase
enoyl-CoA hydratase
enolase-phosphatase
enolase-phosphatase
enolase-phosphatase
hypothetical protein LA_0940
hypothetical protein LA_0940
hypothetical protein LA_0940
hypothetical protein LA_0940
hypothetical protein LA_0940
hypothetical protein LA_0940
hypothetical protein LA_0940
transcription elongation factor NusA
transcription elongation factor NusA
transcription elongation factor NusA
transcription elongation factor NusA
transcription elongation factor NusA
transcription elongation factor NusA
transcription elongation factor NusA
transcription elongation factor NusA
transcription elongation factor NusA
polynucleotide phosphorylase
polynucleotide phosphorylase
polynucleotide phosphorylase
polynucleotide phosphorylase
polynucleotide phosphorylase
polynucleotide phosphorylase
polynucleotide phosphorylase
polynucleotide phosphorylase
polynucleotide phosphorylase
polynucleotide phosphorylase
polynucleotide phosphorylase
polynucleotide phosphorylase
polynucleotide phosphorylase
polynucleotide phosphorylase
polynucleotide phosphorylase
polynucleotide phosphorylase
polynucleotide phosphorylase
polynucleotide phosphorylase
polynucleotide phosphorylase
polynucleotide phosphorylase
hypothetical protein LA_0951
hypothetical protein LA_0951
hypothetical protein LA_0954

hypothetical protein LA_0954
hypothetical protein LA_0954
hypothetical protein LA_0954
hypothetical protein LA_0954
hypothetical protein LA_0954
glutamate synthase subunit alpha
glutamate synthase subunit alpha
glutamate synthase subunit alpha
glutamate synthase subunit alpha
glutamate synthase subunit alpha
glutamate synthase subunit alpha
glutamate synthase subunit alpha
glutamate synthase subunit alpha
glutamate synthase subunit alpha
glutamate synthase subunit alpha
glutamate synthase subunit alpha
glutamate synthase subunit alpha
glutamate synthase subunit alpha
glutamate synthase subunit alpha
glutamate synthase subunit alpha
glutamate synthase subunit alpha
glutamate synthase subunit alpha
glutamate synthase subunit alpha
glutamate synthase subunit alpha
glutamate synthase subunit alpha
glutamate synthase subunit alpha
glutamate synthase subunit alpha
glutamate synthase subunit alpha
glutamate synthase subunit alpha
glutamate synthase subunit alpha
glutamate synthase subunit alpha
glutamate synthase subunit alpha
glutamate synthase subunit alpha
glutamate synthase subunit alpha
glutamate synthase subunit alpha
glutamate synthase subunit alpha
glutamate synthase subunit alpha
glutamate synthase subunit alpha
glutamate synthase subunit alpha
glutamate synthase subunit alpha
glutamate synthase subunit alpha
glutamate synthase subunit alpha
TolC-like outer membrane efflux protein
TolC-like outer membrane efflux protein
TolC-like outer membrane efflux protein
ATP-dependent DNA helicase RecQ
exodeoxyribonuclease V subunit gamma
exodeoxyribonuclease V subunit beta

ABC transporter ATP-binding protein
ABC transporter ATP-binding protein
ABC transporter ATP-binding protein
ABC transporter ATP-binding protein
ABC transporter ATP-binding protein
ABC transporter ATP-binding protein
ABC transporter ATP-binding protein
thiamine biosynthesis protein ThiC
thiamine biosynthesis protein ThiC
thiamine biosynthesis protein ThiC
hypothetical protein LA_0994
hypothetical protein LA_0994
hypothetical protein LA_0999
glutathione peroxidase
glutathione peroxidase
hypothetical protein LA_1008
hypothetical protein LA_1019
transcription termination factor Rho
transcription termination factor Rho
transcription termination factor Rho
transcription termination factor Rho
transcription termination factor Rho
transcription termination factor Rho
transcription termination factor Rho
transcription termination factor Rho
transcription termination factor Rho
transcription termination factor Rho
transcription termination factor Rho
transcription termination factor Rho
transcription termination factor Rho
transcription termination factor Rho
transcription termination factor Rho
transcription termination factor Rho
transcription termination factor Rho
transcription termination factor Rho
transcription termination factor Rho
transcriptional regulator
transcriptional regulator
transcriptional regulator
enoyl-CoA hydratase
enoyl-CoA hydratase
enoyl-CoA hydratase
enoyl-CoA hydratase
enoyl-CoA hydratase
enoyl-CoA hydratase
enoyl-CoA hydratase
enoyl-CoA hydratase
enoyl-CoA hydratase

histidine kinase/response regulator hybrid protein
histidine kinase/response regulator hybrid protein
histidine kinase/response regulator hybrid protein
histidine kinase/response regulator hybrid protein
histidine kinase/response regulator hybrid protein
histidine kinase/response regulator hybrid protein
cell shape determination protein
cell shape determination protein
cell shape determination protein
cell shape determination protein
cell shape determination protein
membrane carboxypeptidase/penicillin-binding protein
membrane carboxypeptidase/penicillin-binding protein
membrane carboxypeptidase/penicillin-binding protein
membrane carboxypeptidase/penicillin-binding protein
membrane carboxypeptidase/penicillin-binding protein
diaminopimelate decarboxylase
diaminopimelate decarboxylase
diaminopimelate decarboxylase
diaminopimelate decarboxylase
diaminopimelate decarboxylase
diaminopimelate decarboxylase
diaminopimelate decarboxylase
diaminopimelate decarboxylase
diaminopimelate decarboxylase
diaminopimelate decarboxylase
diaminopimelate decarboxylase
diaminopimelate decarboxylase
hypothetical protein LA_1052
hypothetical protein LA_1052
hypothetical protein LA_1052
hypothetical protein LA_1052
hypothetical protein LA_1052
hypothetical protein LA_1053
hypothetical protein LA_1053
aminopeptidase N
aminopeptidase N
aminopeptidase N
ADP-ribose pyrophosphatase
TPR-repeat lipoprotein
hypothetical protein LA_1087
hypothetical protein LA_1087
hypothetical protein LA_1087
hypothetical protein LA_1087
hypothetical protein LA_1088
hypothetical protein LA_1092
hypothetical protein LA_1092
hypothetical protein LA_1092

hypothetical protein LA_1097
hypothetical protein LA_1099
hypothetical protein LA_1099
hypothetical protein LA_1099
hypothetical protein LA_1099
hypothetical protein LA_1099
hypothetical protein LA_1099
succinyl-CoA synthetase subunit alpha
succinyl-CoA synthetase subunit alpha
succinyl-CoA synthetase subunit alpha
succinyl-CoA synthetase subunit alpha
succinyl-CoA synthetase subunit alpha
succinyl-CoA synthetase subunit alpha
succinyl-CoA synthetase subunit alpha
succinyl-CoA synthetase subunit alpha
succinyl-CoA synthetase subunit alpha
succinyl-CoA synthetase subunit alpha
succinyl-CoA synthetase subunit alpha
succinyl-CoA synthetase subunit alpha
succinyl-CoA synthetase subunit alpha
succinyl-CoA synthetase subunit alpha
succinyl-CoA synthetase subunit alpha
succinyl-CoA synthetase subunit alpha
succinyl-CoA synthetase subunit beta
succinyl-CoA synthetase subunit beta
succinyl-CoA synthetase subunit beta
succinyl-CoA synthetase subunit beta
succinyl-CoA synthetase subunit beta
succinyl-CoA synthetase subunit beta
succinyl-CoA synthetase subunit beta
succinyl-CoA synthetase subunit beta
succinyl-CoA synthetase subunit beta
succinyl-CoA synthetase subunit beta
succinyl-CoA synthetase subunit beta
succinyl-CoA synthetase subunit beta
succinyl-CoA synthetase subunit beta
succinyl-CoA synthetase subunit beta
hypothetical protein LA_1107
hypothetical protein LA_1107
hypothetical protein LA_1107
hypothetical protein LA_1107
hypothetical protein LA_1107
hypothetical protein LA_1107
hypothetical protein LA_1107
adenylosuccinate synthase
adenylosuccinate synthase
adenylosuccinate synthase
adenylosuccinate synthase

adenylosuccinate synthase
adenylosuccinate synthase
ATP phosphoribosyltransferase regulatory subunit
ATP phosphoribosyltransferase regulatory subunit
ATP phosphoribosyltransferase regulatory subunit
ATP phosphoribosyltransferase regulatory subunit
ATP phosphoribosyltransferase regulatory subunit
glycerol-3-phosphate dehydrogenase
glycerol-3-phosphate dehydrogenase
glycerol-3-phosphate dehydrogenase
glycerol-3-phosphate dehydrogenase
glycerol-3-phosphate dehydrogenase
glycerol-3-phosphate dehydrogenase
glycerol-3-phosphate dehydrogenase
glycerol-3-phosphate dehydrogenase
glycerol-3-phosphate dehydrogenase
glycerol-3-phosphate dehydrogenase
glycerol-3-phosphate dehydrogenase
glycerol-3-phosphate dehydrogenase
glycerol-3-phosphate dehydrogenase
glycerol-3-phosphate dehydrogenase
hypothetical protein LA_1118
hypothetical protein LA_1118
hypothetical protein LA_1118
dUTP diphosphatase
dUTP diphosphatase
dUTP diphosphatase
phosphoglycerate dehydrogenase and related dehydrogenase
phosphoglycerate dehydrogenase and related dehydrogenase
phosphoglycerate dehydrogenase and related dehydrogenase
acyl-CoA dehydrogenase
acyl-CoA dehydrogenase
acyl-CoA dehydrogenase
acyl-CoA dehydrogenase
acyl-CoA dehydrogenase
acyl-CoA dehydrogenase
acyl-CoA dehydrogenase
hypothetical protein LA_1132
hypothetical protein LA_1132
hypothetical protein LA_1132
outer membrane lipoprotein-sorting protein
outer membrane lipoprotein-sorting protein
outer membrane lipoprotein-sorting protein
outer membrane lipoprotein-sorting protein
hypothetical protein LA_1137
hypothetical protein LA_1137
hypothetical protein LA_1137
hypothetical protein LA_1137

hypothetical protein LA_1137
hypothetical protein LA_1137
hypothetical protein LA_1137
hypothetical protein LA_1137
2-methylthioadenine synthetase
2-methylthioadenine synthetase
2-methylthioadenine synthetase
2-methylthioadenine synthetase
anthranilate phosphoribosyl transferase
anthranilate phosphoribosyl transferase
anthranilate phosphoribosyl transferase
anthranilate phosphoribosyl transferase
anthranilate phosphoribosyl transferase
anthranilate phosphoribosyl transferase
anthranilate phosphoribosyl transferase
preprotein translocase subunit YajC
preprotein translocase subunit YajC
preprotein translocase subunit YajC
preprotein translocase subunit YajC
pyrimidine deaminase, riboflavin biosynthesis
bifunctional 3,4-dihydroxy-2-butanone 4-phosphate synthase/GTP cyclohydrolase II
bifunctional 3,4-dihydroxy-2-butanone 4-phosphate synthase/GTP cyclohydrolase II
bifunctional 3,4-dihydroxy-2-butanone 4-phosphate synthase/GTP cyclohydrolase II
bifunctional 3,4-dihydroxy-2-butanone 4-phosphate synthase/GTP cyclohydrolase II
bifunctional 3,4-dihydroxy-2-butanone 4-phosphate synthase/GTP cyclohydrolase II
bifunctional 3,4-dihydroxy-2-butanone 4-phosphate synthase/GTP cyclohydrolase II
bifunctional 3,4-dihydroxy-2-butanone 4-phosphate synthase/GTP cyclohydrolase II
bifunctional 3,4-dihydroxy-2-butanone 4-phosphate synthase/GTP cyclohydrolase II
bifunctional 3,4-dihydroxy-2-butanone 4-phosphate synthase/GTP cyclohydrolase II
sulfate ABC transporter substrate-binding protein
sulfate ABC transporter substrate-binding protein
sulfate ABC transporter substrate-binding protein
sulfate ABC transporter substrate-binding protein
sulfate ABC transporter substrate-binding protein
sulfate ABC transporter substrate-binding protein
sulfate ABC transporter substrate-binding protein
acetyl-CoA synthetase
acetyl-CoA synthetase
acetyl-CoA synthetase
ABC transporter substrate-binding protein
ABC transporter substrate-binding protein
ABC transporter substrate-binding protein
recombination factor protein RarA
recombination factor protein RarA
histidinol-phosphate aminotransferase
histidinol-phosphate aminotransferase
histidinol-phosphate aminotransferase
histidinol-phosphate aminotransferase

histidinol-phosphate aminotransferase
histidinol-phosphate aminotransferase
adenylate/guanylate cyclase
adenylate/guanylate cyclase
signal transduction protein
signal transduction protein
signal transduction protein
signal transduction protein
sigma 54 modulation protein
hypothetical protein LA_1190
hypothetical protein LA_1190
hypothetical protein LA_1190
methyl-accepting chemotaxis protein
methyl-accepting chemotaxis protein
methyl-accepting chemotaxis protein
methyl-accepting chemotaxis protein
methyl-accepting chemotaxis protein
methyl-accepting chemotaxis protein
hypothetical protein LA_1192
hypothetical protein LA_1192
hypothetical protein LA_1192
3-hydroxybutyryl-CoA dehydratase
3-hydroxybutyryl-CoA dehydratase
3-hydroxybutyryl-CoA dehydratase
3-hydroxybutyryl-CoA dehydratase
3-hydroxybutyryl-CoA dehydratase
3-hydroxybutyryl-CoA dehydratase
RNA polymerase ECF-type sigma factor
RNA polymerase ECF-type sigma factor
hypothetical protein LA_1206
hypothetical protein LA_1206
hypothetical protein LA_1206
thioesterase superfamily protein
thioesterase superfamily protein
hypothetical protein LA_1209
methyl-accepting chemotaxis protein
metal-dependent hydrolase
metal-dependent hydrolase
metal-dependent hydrolase
membrane carboxypeptidase/penicillin-binding protein
membrane carboxypeptidase/penicillin-binding protein
dihydrolipoamide acetyltransferase
dihydrolipoamide acetyltransferase
dihydrolipoamide acetyltransferase
dihydrolipoamide acetyltransferase
dihydrolipoamide acetyltransferase
dihydrolipoamide acetyltransferase
dihydrolipoamide acetyltransferase

dihydrolipoamide acetyltransferase
dihydrolipoamide acetyltransferase
dihydrolipoamide dehydrogenase
dihydrolipoamide dehydrogenase
dihydrolipoamide dehydrogenase
dihydrolipoamide dehydrogenase
dihydrolipoamide dehydrogenase
dihydrolipoamide dehydrogenase
dihydrolipoamide dehydrogenase
dihydrolipoamide dehydrogenase
dihydrolipoamide dehydrogenase
dihydrolipoamide dehydrogenase
alpha-ketoglutarate decarboxylase
alpha-ketoglutarate decarboxylase
alpha-ketoglutarate decarboxylase
alpha-ketoglutarate decarboxylase
alpha-ketoglutarate decarboxylase
alpha-ketoglutarate decarboxylase
alpha-ketoglutarate decarboxylase
alpha-ketoglutarate decarboxylase
alpha-ketoglutarate decarboxylase
alpha-ketoglutarate decarboxylase
alpha-ketoglutarate decarboxylase
alpha-ketoglutarate decarboxylase
alpha-ketoglutarate decarboxylase
alpha-ketoglutarate decarboxylase
alpha-ketoglutarate decarboxylase
alpha-ketoglutarate decarboxylase
alpha-ketoglutarate decarboxylase
alpha-ketoglutarate decarboxylase
alpha-ketoglutarate decarboxylase
threonyl-tRNA synthetase
threonyl-tRNA synthetase
threonyl-tRNA synthetase
threonyl-tRNA synthetase
threonyl-tRNA synthetase
threonyl-tRNA synthetase
threonyl-tRNA synthetase
threonyl-tRNA synthetase
threonyl-tRNA synthetase
threonyl-tRNA synthetase
threonyl-tRNA synthetase
threonyl-tRNA synthetase
threonyl-tRNA synthetase
threonyl-tRNA synthetase
threonyl-tRNA synthetase
threonyl-tRNA synthetase
translation initiation factor IF-3

translation initiation factor IF-3
50S ribosomal protein L35
50S ribosomal protein L35
50S ribosomal protein L35
50S ribosomal protein L20
50S ribosomal protein L20
50S ribosomal protein L20
50S ribosomal protein L20
hypothetical protein LA_1245
hypothetical protein LA_1245
hypothetical protein LA_1245
hypothetical protein LA_1245
5-formyltetrahydrofolate cyclo-ligase
5-formyltetrahydrofolate cyclo-ligase
chemotaxis protein histidine kinase CheA
chemotaxis protein histidine kinase CheA
chemotaxis protein histidine kinase CheA
chemotaxis protein histidine kinase CheA
chemotaxis protein histidine kinase CheA
chemotaxis protein histidine kinase CheA
chemotaxis protein histidine kinase CheA
chemotaxis protein histidine kinase CheA
chemotaxis protein histidine kinase CheA
chemotaxis protein histidine kinase CheA
chemotaxis-specific methylesterase CheB
chemotaxis-specific methylesterase CheB
two-component response regulator receiver protein
two-component response regulator receiver protein
two-component response regulator receiver protein
two-component response regulator receiver protein
segregation and condensation protein A
segregation and condensation protein B
segregation and condensation protein B
segregation and condensation protein B
bifunctional prephenate dehydrogenase/chorismate mutase
bifunctional prephenate dehydrogenase/chorismate mutase
3-phosphoshikimate 1-carboxyvinyltransferase
3-phosphoshikimate 1-carboxyvinyltransferase
3-phosphoshikimate 1-carboxyvinyltransferase
3-phosphoshikimate 1-carboxyvinyltransferase
30S ribosomal protein S1
30S ribosomal protein S1
30S ribosomal protein S1
30S ribosomal protein S1
30S ribosomal protein S1
30S ribosomal protein S1
30S ribosomal protein S1
30S ribosomal protein S1

30S ribosomal protein S1
30S ribosomal protein S1
30S ribosomal protein S1
30S ribosomal protein S1
30S ribosomal protein S1
30S ribosomal protein S1
30S ribosomal protein S1
30S ribosomal protein S1
30S ribosomal protein S1
30S ribosomal protein S1
30S ribosomal protein S1
30S ribosomal protein S1
hypothetical protein LA_1261
hypothetical protein LA_1261
hypothetical protein LA_1261
hypothetical protein LA_1261
50S ribosomal protein L32
fatty acid/phospholipid synthesis protein
fatty acid/phospholipid synthesis protein
hypothetical protein LA_1273
phosphoglycerol transferase-like protein
phosphoglycerol transferase-like protein
phosphoglycerol transferase-like protein
phosphoglycerol transferase-like protein
phosphoglycerol transferase-like protein
phosphoglycerol transferase-like protein
phosphoglycerol transferase-like protein
phosphoglycerol transferase-like protein
phosphoglycerol transferase-like protein
aspartate/tyrosine/aromatic aminotransferase
aspartate/tyrosine/aromatic aminotransferase
aspartate/tyrosine/aromatic aminotransferase
aspartate/tyrosine/aromatic aminotransferase
GTP-binding protein EngA
GTP-binding protein EngA
glutamine synthetase
glutamine synthetase
glutamine synthetase
glutamine synthetase
glutamine synthetase
glutamine synthetase
glutamine synthetase
glutamine synthetase
glutamine synthetase
glutamine synthetase
glutamine synthetase
glutamine synthetase
glutamine synthetase

glutamine synthetase
glutamine synthetase
glutamine synthetase
phosphoribosylformylglycinamidine (FGAM) synthase
phosphoribosylformylglycinamidine (FGAM) synthase
phosphoribosylformylglycinamidine (FGAM) synthase
phosphoribosylformylglycinamidine (FGAM) synthase
phosphoribosylformylglycinamidine (FGAM) synthase
phosphoribosylformylglycinamidine (FGAM) synthase
phosphoribosylformylglycinamidine (FGAM) synthase
phosphoribosylformylglycinamidine (FGAM) synthase
phosphoribosylformylglycinamidine (FGAM) synthase
isoleucyl-tRNA synthetase
isoleucyl-tRNA synthetase
isoleucyl-tRNA synthetase
isoleucyl-tRNA synthetase
isoleucyl-tRNA synthetase
isoleucyl-tRNA synthetase
isoleucyl-tRNA synthetase
isoleucyl-tRNA synthetase
isoleucyl-tRNA synthetase
isoleucyl-tRNA synthetase
isoleucyl-tRNA synthetase
hypothetical protein LA_1326
hypothetical protein LA_1326
hypothetical protein LA_1326
hypothetical protein LA_1326
hypothetical protein LA_1329
hypothetical protein LA_1329
hypothetical protein LA_1329
hypothetical protein LA_1329
oxidoreductase
signal peptidase II
cAMP-binding protein
cAMP-binding protein
histidine kinase sensor protein
histidine kinase sensor protein
histidine kinase sensor protein
histidine kinase sensor protein
capsule biosynthesis protein CapA
capsule biosynthesis protein CapA
capsule biosynthesis protein CapA
capsule biosynthesis protein CapA
capsule biosynthesis protein CapA
hypothetical protein LA_1359
hypothetical protein LA_1359
hypothetical protein LA_1359
GTP-binding protein BipA

GTP-binding protein BipA
GTP-binding protein BipA
GTP-binding protein BipA
GTP-binding protein BipA
GTP-binding protein BipA
GTP-binding protein BipA
GTP-binding protein BipA
GTP-binding protein BipA
GTP-binding protein BipA
GTP-binding protein BipA
GTP-binding protein BipA
GTP-binding protein BipA
hypothetical protein LA_1384
hypothetical protein LA_1384
hypothetical protein LA_1384
hypothetical protein LA_1384
glycyl-tRNA synthetase
glycyl-tRNA synthetase
glycyl-tRNA synthetase
glycyl-tRNA synthetase
glycyl-tRNA synthetase
glycyl-tRNA synthetase
hypothetical protein LA_1390
hypothetical protein LA_1390
ribosomal protein L11 methylase
ribosomal protein L11 methylase
ribokinase
ribokinase
ribokinase
ribokinase
export protein
export protein
export protein
export protein
export protein
export protein
hypothetical protein LA_1398
hypothetical protein LA_1398
hypothetical protein LA_1398
hypothetical protein LA_1398
serine hydroxymethyltransferase
serine hydroxymethyltransferase
serine hydroxymethyltransferase
serine hydroxymethyltransferase
serine hydroxymethyltransferase
serine hydroxymethyltransferase
serine hydroxymethyltransferase
serine hydroxymethyltransferase

serine hydroxymethyltransferase
serine hydroxymethyltransferase
serine hydroxymethyltransferase
serine hydroxymethyltransferase
serine hydroxymethyltransferase
serine hydroxymethyltransferase
serine hydroxymethyltransferase
serine hydroxymethyltransferase
serine hydroxymethyltransferase
serine hydroxymethyltransferase
serine hydroxymethyltransferase
serine hydroxymethyltransferase
lipoate protein ligase A
hypothetical protein LA_1413
2-methylisocitrate lyase
lactoylglutathione lyase
lactoylglutathione lyase
lactoylglutathione lyase
lactoylglutathione lyase
lactoylglutathione lyase
lactoylglutathione lyase
hypothetical protein LA_1420
hypothetical protein LA_1420
hypothetical protein LA_1420
serine/threonine kinase
serine/threonine kinase
serine/threonine kinase
serine/threonine kinase
serine/threonine kinase
serine/threonine kinase
serine/threonine kinase
hypothetical protein LA_1429
glucose kinase
glucose kinase
glucose kinase
antimicrobial peptide ABC transporter ATPase
hypothetical protein LA_1440
hypothetical protein LA_1440
hypothetical protein LA_1440
heavy metal efflux pump
heavy metal efflux pump
heavy metal efflux pump
heavy metal efflux pump
carboxy-terminal processing protease
carboxy-terminal processing protease
carboxy-terminal processing protease
carboxy-terminal processing protease
ABC transporter ATP-binding protein

UDP-glucose 6-dehydrogenase
UDP-glucose 6-dehydrogenase
UDP-glucose 6-dehydrogenase
UDP-glucose 6-dehydrogenase
UDP-glucose 6-dehydrogenase
UDP-glucose 6-dehydrogenase
UDP-glucose 6-dehydrogenase
UDP-glucose 6-dehydrogenase
UDP-glucose 6-dehydrogenase
phosphoribosylaminoimidazole carboxylase catalytic subunit
UDP-N-acetylmuramyl pentapeptide synthase
UDP-N-acetylmuramyl pentapeptide synthase
hypothetical protein LA_1479
PLP dependent enzyme class III
PLP dependent enzyme class III
PLP dependent enzyme class III
glycolate oxidase
glycolate oxidase
glycolate oxidase
glycolate oxidase
glycolate oxidase
glycolate oxidase
hypothetical protein LA_1495
hypothetical protein LA_1495
hypothetical protein LA_1495
hypothetical protein LA_1495
hypothetical protein LA_1495
hypothetical protein LA_1495
L-aspartate oxidase
L-aspartate oxidase
pyridoxal phosphate biosynthetic protein PdxJ
pyridoxal phosphate biosynthetic protein PdxJ
tRNA isopentenyltransferase
tRNA isopentenyltransferase
host factor I related protein
mannose-1-phosphate guanylyltransferase
mannose-1-phosphate guanylyltransferase
mannose-1-phosphate guanylyltransferase
mannose-1-phosphate guanylyltransferase
mannose-1-phosphate guanylyltransferase
chemotaxis protein CheW
chemotaxis protein CheW
signal transduction protein
hypothetical protein LA_1530
hypothetical protein LA_1530
fructose-bisphosphate aldolase
fructose-bisphosphate aldolase
fructose-bisphosphate aldolase

fructose-bisphosphate aldolase
fructose-bisphosphate aldolase
fructose-bisphosphate aldolase
fructose-bisphosphate aldolase
fructose-bisphosphate aldolase
fructose-bisphosphate aldolase
fructose-bisphosphate aldolase
fructose-bisphosphate aldolase
fructose-bisphosphate aldolase
hypothetical protein LA_1535
hypothetical protein LA_1535
heavy metal efflux pump
heavy metal efflux pump
heavy metal efflux pump
heavy metal efflux pump
DNA methyltransferase
DNA methyltransferase
hypothetical protein LA_1553
hypothetical protein LA_1553
Hsp20/alpha crystallin molecular chaperone
Hsp20/alpha crystallin molecular chaperone
Hsp20/alpha crystallin molecular chaperone
Hsp20/alpha crystallin molecular chaperone
nucleoside-diphosphate-sugar epimerase
nucleoside-diphosphate-sugar epimerase
nucleoside-diphosphate-sugar epimerase
FAD dependent oxidoreductase
FAD dependent oxidoreductase
FAD dependent oxidoreductase
FAD dependent oxidoreductase
phosphoheptose isomerase
phosphoheptose isomerase
phosphoheptose isomerase
nucleoside-diphosphate-sugar epimerase
nucleoside-diphosphate-sugar epimerase
nucleoside-diphosphate-sugar epimerase
nucleoside-diphosphate-sugar epimerase
nucleoside-diphosphate-sugar epimerase
nucleoside-diphosphate-sugar epimerase
nucleoside-diphosphate-sugar epimerase
nucleoside-diphosphate-sugar epimerase
nucleoside-diphosphate-sugar epimerase
nucleoside-diphosphate-sugar epimerase
nucleoside-diphosphate-sugar epimerase
nucleoside-diphosphate-sugar epimerase
nucleoside-diphosphate-sugar epimerase
nucleoside-diphosphate-sugar epimerase
nucleoside-diphosphate-sugar epimerase

nucleoside-diphosphate-sugar epimerase
nucleoside-diphosphate-sugar epimerase
nucleoside-diphosphate-sugar epimerase
nucleoside-diphosphate-sugar epimerase
nucleoside-diphosphate-sugar epimerase
nucleoside-diphosphate-sugar epimerase
nucleoside-diphosphate-sugar epimerase
nucleoside-diphosphate-sugar epimerase
hypothetical protein LA_1587
hypothetical protein LA_1587
hypothetical protein LA_1587
hypothetical protein LA_1587
hypothetical protein LA_1587
SAM-dependent methyltransferase
SAM-dependent methyltransferase
SAM-dependent methyltransferase
SAM-dependent methyltransferase
SAM-dependent methyltransferase
SAM-dependent methyltransferase
ABC transporter ATP-binding protein
ABC transporter ATP-binding protein
ABC transporter ATP-binding protein
ABC transporter ATP-binding protein
glycosyltransferase
glycosyltransferase
hypothetical protein LA_1604
hypothetical protein LA_1604
CMP-N-acetylneuraminic acid synthetase
nucleoside-diphosphate-sugar epimerase
nucleoside-diphosphate-sugar epimerase
nucleoside-diphosphate-sugar epimerase
nucleoside-diphosphate-sugar epimerase
pyridoxal phosphate-dependent aminotransferase
pyridoxal phosphate-dependent aminotransferase
UDP-N-acetylglucosamine 2-epimerase
UDP-N-acetylglucosamine 2-epimerase
UDP-N-acetylglucosamine 2-epimerase
nucleotidyl transferase
nucleotidyl transferase
N-acetylneuraminic (sialic) acid synthetase
N-acetylneuraminic (sialic) acid synthetase
N-acetylneuraminic (sialic) acid synthetase
N-acetylneuraminic (sialic) acid synthetase
N-acetylneuraminic (sialic) acid synthetase
N-acetylneuraminic (sialic) acid synthetase
N-acetylneuraminic (sialic) acid synthetase
N-acetylneuraminic (sialic) acid synthetase
N-acetylneuraminic (sialic) acid synthetase

N-acetylneuraminic (sialic) acid synthetase
pyridoxal phosphate-dependent aminotransferase
pyridoxal phosphate-dependent aminotransferase
pyridoxal phosphate-dependent aminotransferase
pyridoxal phosphate-dependent aminotransferase
pyridoxal phosphate-dependent aminotransferase
pyridoxal phosphate-dependent aminotransferase
pyridoxal phosphate-dependent aminotransferase
alcohol dehydrogenase
alcohol dehydrogenase
alcohol dehydrogenase
alcohol dehydrogenase
alcohol dehydrogenase
alcohol dehydrogenase
alcohol dehydrogenase
molybdenum cofactor biosynthesis protein
molybdenum cofactor biosynthesis protein
molybdenum cofactor biosynthesis protein
molybdenum cofactor biosynthesis protein
molybdenum cofactor biosynthesis protein
molybdenum cofactor biosynthesis protein
molybdenum cofactor biosynthesis protein
methyltransferase-like protein
carbamoyl transferase
carbamoyl transferase
carbamoyl transferase
carbamoyl transferase
carbamoyl transferase
carbamoyl transferase
carbamoyl transferase
carbamoyl transferase
carbamoyl transferase
carbamoyl transferase
carbamoyl transferase
carbamoyl transferase
carbamoyl transferase
carbamoyl transferase
carbamoyl transferase
carbamoyl transferase
acetyltransferase
acetyltransferase
2,4-dihydroxyhept-2-ene-1,7-dioic acid aldolase
2,4-dihydroxyhept-2-ene-1,7-dioic acid aldolase
3-deoxy-manno-octulosonate cytidylyltransferase
3-deoxy-manno-octulosonate cytidylyltransferase
3-deoxy-manno-octulosonate cytidylyltransferase
3-deoxy-manno-octulosonate cytidylyltransferase
oxidoreductase family protein

inositol monophosphatase family protein
inositol monophosphatase family protein
inositol monophosphatase family protein
phosphoglycerate dehydrogenase and related dehydrogenase
phosphoglycerate dehydrogenase and related dehydrogenase
phosphoglycerate dehydrogenase and related dehydrogenase
phosphoglycerate dehydrogenase and related dehydrogenase
phosphoglycerate dehydrogenase and related dehydrogenase
phosphoglycerate dehydrogenase and related dehydrogenase
nucleotidyl transferase
CDP-glucose 4,6-dehydratase
CDP-glucose 4,6-dehydratase
glycosyltransferase
polysaccharide deacetylase-like protein
polysaccharide biosynthesis protein
polysaccharide biosynthesis protein
polysaccharide biosynthesis protein
polysaccharide biosynthesis protein
polysaccharide biosynthesis protein
polysaccharide biosynthesis protein
polysaccharide biosynthesis protein
polysaccharide biosynthesis protein
polysaccharide biosynthesis protein
polysaccharide biosynthesis protein
polysaccharide biosynthesis protein
nucleoside-diphosphate-sugar epimerase
nucleoside-diphosphate-sugar epimerase
nucleoside-diphosphate-sugar epimerase
nucleoside-diphosphate-sugar epimerase
nucleoside-diphosphate-sugar epimerase
nucleoside-diphosphate-sugar epimerase
UDP-N-acetylglucosamine 2-epimerase
UDP-N-acetylglucosamine 2-epimerase
UDP-N-acetylglucosamine 2-epimerase
UDP-N-acetylglucosamine 2-epimerase
UDP-N-acetylglucosamine 2-epimerase
UDP-N-acetylglucosamine 2-epimerase
UDP-N-acetylglucosamine 2-epimerase
UDP-N-acetylglucosamine 2-epimerase
UDP-N-acetylglucosamine 2-epimerase
glycosyltransferase
glycosyltransferase
glycosyltransferase
hypothetical protein LA_1648
glycosyltransferase
hypothetical protein LA_1652
hypothetical protein LA_1652
hypothetical protein LA_1652

hypothetical protein LA_1652
FdtA-like protein
FdtA-like protein
pyridoxal phosphate-dependent aminotransferase
pyridoxal phosphate-dependent aminotransferase
pyridoxal phosphate-dependent aminotransferase
pyridoxal phosphate-dependent aminotransferase
glycosyltransferase
glycosyltransferase
glycosyltransferase
glycosyltransferase
dTDP-4-dehydrorhamnose 3,5-epimerase
dTDP-4-dehydrorhamnose 3,5-epimerase
dTDP-4-dehydrorhamnose 3,5-epimerase
dTDP-4-dehydrorhamnose 3,5-epimerase
dTDP-4-dehydrorhamnose 3,5-epimerase
dTDP-4-dehydrorhamnose reductase
glucose-1-phosphate thymidylyltransferase
glucose-1-phosphate thymidylyltransferase
glucose-1-phosphate thymidylyltransferase
glucose-1-phosphate thymidylyltransferase
glycosyltransferase
poly-gamma-glutamate synthesis protein
poly-gamma-glutamate synthesis protein
poly-gamma-glutamate synthesis protein
poly-gamma-glutamate synthesis protein
poly-gamma-glutamate synthesis protein
30S ribosomal protein S6
30S ribosomal protein S6
30S ribosomal protein S6
30S ribosomal protein S6
30S ribosomal protein S6
30S ribosomal protein S6
single-stranded DNA-binding protein
single-stranded DNA-binding protein
30S ribosomal protein S18
30S ribosomal protein S18
30S ribosomal protein S18
50S ribosomal protein L9
50S ribosomal protein L9
50S ribosomal protein L9
50S ribosomal protein L9
50S ribosomal protein L9
aspartyl-tRNA synthetase
aspartyl-tRNA synthetase
aspartyl-tRNA synthetase
aspartyl-tRNA synthetase
aspartyl-tRNA synthetase

aspartyl-tRNA synthetase
aspartyl-tRNA synthetase
aspartyl-tRNA synthetase
aspartyl-tRNA synthetase
aspartyl-tRNA synthetase
aspartyl-tRNA synthetase
aspartyl-tRNA synthetase
aspartyl-tRNA synthetase
aspartyl-tRNA synthetase
membrane-associated HD superfamily hydrolase
membrane-associated HD superfamily hydrolase
competence-damage inducible protein CinA
competence-damage inducible protein CinA
competence-damage inducible protein CinA
hypothetical protein LA_1690
hypothetical protein LA_1690
triosephosphate isomerase
triosephosphate isomerase
triosephosphate isomerase
phosphoglycerate kinase
phosphoglycerate kinase
phosphoglycerate kinase
phosphoglycerate kinase
phosphoglycerate kinase
phosphoglycerate kinase
phosphoglycerate kinase
phosphoglycerate kinase
phosphoglycerate kinase
phosphoglycerate kinase
phosphoglycerate kinase
phosphoglycerate kinase
phosphoglycerate kinase
phosphoglycerate kinase
phosphoglycerate kinase
phosphoglycerate kinase
phosphoglycerate kinase
glyceraldehyde 3-phosphate dehydrogenase
glyceraldehyde 3-phosphate dehydrogenase
glyceraldehyde 3-phosphate dehydrogenase
glyceraldehyde 3-phosphate dehydrogenase
glyceraldehyde 3-phosphate dehydrogenase
glyceraldehyde 3-phosphate dehydrogenase
glyceraldehyde 3-phosphate dehydrogenase
glyceraldehyde 3-phosphate dehydrogenase
glyceraldehyde 3-phosphate dehydrogenase
glyceraldehyde 3-phosphate dehydrogenase
glyceraldehyde 3-phosphate dehydrogenase
glyceraldehyde 3-phosphate dehydrogenase

glyceraldehyde 3-phosphate dehydrogenase
hypothetical protein LA_1715
hypothetical protein LA_1715
hypothetical protein LA_1715
hypothetical protein LA_1715
hypothetical protein LA_1715
hypothetical protein LA_1715
hypothetical protein LA_1715
hypothetical protein LA_1715
cysteine synthase
cysteine synthase
cysteine synthase
cysteine synthase
cysteine synthase
cysteine synthase
cysteine synthase
cysteine synthase
cysteine synthase
cysteine synthase
cysteine synthase
cysteine synthase
cysteine synthase
cysteine synthase
cysteine synthase
cysteine synthase
cysteine synthase
cysteine synthase
ABC transporter ATP-binding protein
ABC transporter ATP-binding protein
HD family protein
HD family protein
HD family protein
HD family protein
HD family protein
HD family protein
HD family protein
HD family protein
hypothetical protein LA_1728
hypothetical protein LA_1728
hypothetical protein LA_1728
hypothetical protein LA_1728
hypothetical protein LA_1728
hypothetical protein LA_1728
hypothetical protein LA_1728
hypothetical protein LA_1728
hypothetical protein LA_1728
hypothetical protein LA_1728
hypothetical protein LA_1733

hypothetical protein LA_1733
hypothetical protein LA_1733
anti-sigma factor antagonist-like protein
anti-sigma factor antagonist-like protein
anti-sigma factor antagonist-like protein
hypothetical protein LA_1740
hypothetical protein LA_1740
hypothetical protein LA_1740
acyltransferase
two component system sensor histidine kinase\response regulator
ppGpp-regulated growth inhibitor ChpA/MazF
transposase
transposase
hypothetical protein LA_1853
hypothetical protein LA_1853
hypothetical protein LA_1853
hypothetical protein LA_1853
ferric uptake regulator
ferric uptake regulator
hypothetical protein LA_1861
cysteinyl-tRNA synthetase
cysteinyl-tRNA synthetase
cysteinyl-tRNA synthetase
cysteinyl-tRNA synthetase
cysteinyl-tRNA synthetase
acetyl esterase
acetyl esterase
acetyl esterase
acetyl esterase
acetyl esterase
acetyl esterase
acetyl esterase
ınctional 5,10-methylene-tetrahydrofolate dehydrogenase/ 5,10-methylene-tetrahydrofolate cyclohydrol
ınctional 5,10-methylene-tetrahydrofolate dehydrogenase/ 5,10-methylene-tetrahydrofolate cyclohydrol
ınctional 5,10-methylene-tetrahydrofolate dehydrogenase/ 5,10-methylene-tetrahydrofolate cyclohydrol
ınctional 5,10-methylene-tetrahydrofolate dehydrogenase/ 5,10-methylene-tetrahydrofolate cyclohydrol
ınctional 5,10-methylene-tetrahydrofolate dehydrogenase/ 5,10-methylene-tetrahydrofolate cyclohydrol
asparaginyl-tRNA synthetase
asparaginyl-tRNA synthetase
asparaginyl-tRNA synthetase
asparaginyl-tRNA synthetase
asparaginyl-tRNA synthetase
asparaginyl-tRNA synthetase
asparaginyl-tRNA synthetase
hypothetical protein LA_1867
hypothetical protein LA_1867
ATP-dependent helicase
ATP-dependent helicase

ATP-dependent helicase
ATP-dependent helicase
ATP-dependent protease ClpA
ATP-dependent protease ClpA
ATP-dependent protease ClpA
ATP-dependent protease ClpA
ATP-dependent protease ClpA
ATP-dependent protease ClpA
ATP-dependent protease ClpA
ATP-dependent protease ClpA
ATP-dependent protease ClpA
ATP-dependent protease ClpA
ATP-dependent protease ClpA
ATP-dependent protease ClpA
ATP-dependent protease ClpA
ATP-dependent protease ClpA
ATP-dependent protease ClpA
ATP-dependent protease ClpA
ATP-dependent protease ClpA
hypothetical protein LA_1883
hypothetical protein LA_1883
hypothetical protein LA_1883
hypothetical protein LA_1883
hypothetical protein LA_1883
hypothetical protein LA_1883
hypothetical protein LA_1883
hypothetical protein LA_1883
hypothetical protein LA_1883
hypothetical protein LA_1883
hypothetical protein LA_1883
hypothetical protein LA_1883
hypothetical protein LA_1883
hypothetical protein LA_1883
hypothetical protein LA_1883
hypothetical protein LA_1883
hypothetical protein LA_1883
hypothetical protein LA_1883
hypothetical protein LA_1883
anti-sigma factor antagonist
GTP-binding protein LepA
GTP-binding protein LepA
GTP-binding protein LepA
GTP-binding protein LepA
GTP-binding protein LepA
GTP-binding protein LepA
succinate dehydrogenase/fumarate reductase iron-sulfur subunit
succinate dehydrogenase/fumarate reductase iron-sulfur subunit
succinate dehydrogenase/fumarate reductase iron-sulfur subunit

succinate dehydrogenase/fumarate reductase iron-sulfur subunit
succinate dehydrogenase flavoprotein subunit
succinate dehydrogenase flavoprotein subunit
succinate dehydrogenase flavoprotein subunit
succinate dehydrogenase flavoprotein subunit
succinate dehydrogenase flavoprotein subunit
succinate dehydrogenase flavoprotein subunit
succinate dehydrogenase flavoprotein subunit
succinate dehydrogenase flavoprotein subunit
succinate dehydrogenase flavoprotein subunit
succinate dehydrogenase flavoprotein subunit
succinate dehydrogenase flavoprotein subunit
succinate dehydrogenase flavoprotein subunit
succinate dehydrogenase flavoprotein subunit
succinate:quinone oxidoreductase (SQR)-like protein
aldo/keto oxidoreductase
aldo/keto oxidoreductase
aldo/keto oxidoreductase
phosphoglycerate dehydrogenase and related dehydrogenase
phosphoglycerate dehydrogenase and related dehydrogenase
phosphoglycerate dehydrogenase and related dehydrogenase
phosphoglycerate dehydrogenase and related dehydrogenase
phosphoglycerate dehydrogenase and related dehydrogenase
phosphoglycerate dehydrogenase and related dehydrogenase
phosphoglycerate dehydrogenase and related dehydrogenase
phosphoglycerate dehydrogenase and related dehydrogenase
phosphoglycerate dehydrogenase and related dehydrogenase
phosphoglycerate dehydrogenase and related dehydrogenase
phosphoglycerate dehydrogenase and related dehydrogenase
phosphoglycerate dehydrogenase and related dehydrogenase
phosphoglycerate dehydrogenase and related dehydrogenase
TPR-repeat-containing protein
TPR-repeat-containing protein
TPR-repeat-containing protein
hypothetical protein LA_1917
RNA-binding protein
RNA-binding protein
RNA-binding protein
RNA-binding protein
RNA-binding protein
RNA-binding protein
hypothetical protein LA_1924
hypothetical protein LA_1924
hypothetical protein LA_1927
hypothetical protein LA_1927
hypothetical protein LA_1927
thioredoxin
thioredoxin

thioredoxin
CAP family transcription factor
CAP family transcription factor
CAP family transcription factor
CAP family transcription factor
CAP family transcription factor
CAP family transcription factor
CAP family transcription factor
CAP family transcription factor
CAP family transcription factor
CAP family transcription factor
CAP family transcription factor
CAP family transcription factor
CAP family transcription factor
CAP family transcription factor
CAP family transcription factor
CAP family transcription factor
CAP family transcription factor
CAP family transcription factor
CAP family transcription factor
CAP family transcription factor
CAP family transcription factor
CAP family transcription factor
acyl-CoA dehydrogenase
acyl-CoA dehydrogenase
acyl-CoA dehydrogenase
acyl-CoA dehydrogenase
acyl-CoA dehydrogenase
hypothetical protein LA_1935
hypothetical protein LA_1935
hypothetical protein LA_1935
hypothetical protein LA_1935
hydrolase/acyltransferase
hydrolase/acyltransferase
hydrolase/acyltransferase
hydrolase/acyltransferase
hydrolase/acyltransferase
hydrolase/acyltransferase
hydrolase/acyltransferase
hydrolase/acyltransferase
hydrolase/acyltransferase
hypothetical protein LA_1939
hypothetical protein LA_1939
hypothetical protein LA_1939
hypothetical protein LA_1946
hypothetical protein LA_1947
hypothetical protein LA_1947
hypothetical protein LA_1947

hypothetical protein LA_1947
hypothetical protein LA_1947
hypothetical protein LA_1947
two-component response regulator receiver protein
two-component response regulator receiver protein
enolase
enolase
enolase
enolase
enolase
enolase
enolase
enolase
enolase
hypothetical protein LA_1957
hypothetical protein LA_1957
hypothetical protein LA_1957
short-chain dehydrogenase
short-chain dehydrogenase
short-chain dehydrogenase
short-chain dehydrogenase
short-chain dehydrogenase
short-chain dehydrogenase
short-chain dehydrogenase
short-chain dehydrogenase
FAD-binding oxidoreductase
FAD-binding oxidoreductase
FAD-binding oxidoreductase
FAD-binding oxidoreductase
hypothetical protein LA_1978
hypothetical protein LA_1978
hypothetical protein LA_1978
glycosyltransferase
glycosyltransferase
hypothetical protein LA_1981
hypothetical protein LA_1981
hypothetical protein LA_1985
hypothetical protein LA_1985
inosine-5'-monophosphate dehydrogenase
inosine-5'-monophosphate dehydrogenase
inosine-5'-monophosphate dehydrogenase
inosine-5'-monophosphate dehydrogenase
inosine-5'-monophosphate dehydrogenase
inosine-5'-monophosphate dehydrogenase
inosine-5'-monophosphate dehydrogenase
inosine-5'-monophosphate dehydrogenase
inosine-5'-monophosphate dehydrogenase
inosine-5'-monophosphate dehydrogenase

inosine-5'-monophosphate dehydrogenase
inosine-5'-monophosphate dehydrogenase
hypothetical protein LA_1988
hypothetical protein LA_1988
thiamine phosphate pyrophosphorylase
thiamine phosphate pyrophosphorylase
glycosyltransferase
glycosyltransferase
glycosyltransferase
lysyl-tRNA synthetase
lysyl-tRNA synthetase
lysyl-tRNA synthetase
lysyl-tRNA synthetase
lysyl-tRNA synthetase
lysyl-tRNA synthetase
lysyl-tRNA synthetase
lysyl-tRNA synthetase
hypothetical protein LA_1996
hypothetical protein LA_1996
flagellar switch complex protein
flagellar switch complex protein
pyruvate dehydrogenase subunit beta
pyruvate dehydrogenase subunit beta
pyruvate dehydrogenase subunit beta
pyruvate dehydrogenase subunit alpha
pyruvate dehydrogenase subunit alpha
pyruvate dehydrogenase subunit alpha
pyruvate dehydrogenase subunit alpha
response regulator containing a signal receiver domain and a DNA/RNA polymerase-binding domain
endoflagellar filament core protein
endoflagellar filament core protein
endoflagellar filament core protein
endoflagellar filament core protein
endoflagellar filament core protein
endoflagellar filament core protein
endoflagellar filament core protein
endoflagellar filament core protein
endoflagellar filament core protein
endoflagellar filament core protein
endoflagellar filament core protein
endoflagellar filament core protein
endoflagellar filament core protein
endoflagellar filament core protein
endoflagellar filament core protein
flagellin protein
flagellin protein
flagellin protein
flagellin protein

flagellin protein
flagellin protein
hypothetical protein LA_2024
hypothetical protein LA_2024
hypothetical protein LA_2024
hypothetical protein LA_2024
hypothetical protein LA_2024
hypothetical protein LA_2024
hypothetical protein LA_2024
hypothetical protein LA_2024
hypothetical protein LA_2024
hypothetical protein LA_2024
hypothetical protein LA_2024
hypothetical protein LA_2024
hypothetical protein LA_2024
hypothetical protein LA_2024
hypothetical protein LA_2024
hypothetical protein LA_2024
hypothetical protein LA_2024
hypothetical protein LA_2024
hypothetical protein LA_2024
hypothetical protein LA_2027
hypothetical protein LA_2035
methylase of chemotaxis methyl-accepting protein
methylase of chemotaxis methyl-accepting protein
methylase of chemotaxis methyl-accepting protein
S-adenosyl-methyltransferase
undecaprenyldiphospho-muramoylpentapeptide beta- N-acetylglucosaminyltransferase
undecaprenyldiphospho-muramoylpentapeptide beta- N-acetylglucosaminyltransferase
undecaprenyldiphospho-muramoylpentapeptide beta- N-acetylglucosaminyltransferase
undecaprenyldiphospho-muramoylpentapeptide beta- N-acetylglucosaminyltransferase
undecaprenyldiphospho-muramoylpentapeptide beta- N-acetylglucosaminyltransferase
hypothetical protein LA_2053
hypothetical protein LA_2053
D-alanine--D-alanine ligase
D-alanine--D-alanine ligase
methyltransferase
homoserine O-acetyltransferase
O-acetylhomoserine/O-acetylserine sulfhydrylase
O-acetylhomoserine/O-acetylserine sulfhydrylase
O-acetylhomoserine/O-acetylserine sulfhydrylase
O-acetylhomoserine/O-acetylserine sulfhydrylase
O-acetylhomoserine/O-acetylserine sulfhydrylase
O-acetylhomoserine/O-acetylserine sulfhydrylase
O-acetylhomoserine/O-acetylserine sulfhydrylase
O-acetylhomoserine/O-acetylserine sulfhydrylase
O-acetylhomoserine/O-acetylserine sulfhydrylase
O-acetylhomoserine/O-acetylserine sulfhydrylase

O-acetylhomoserine/O-acetylserine sulfhydrylase
O-acetylhomoserine/O-acetylserine sulfhydrylase
O-acetylhomoserine/O-acetylserine sulfhydrylase
O-acetylhomoserine/O-acetylserine sulfhydrylase
O-acetylhomoserine/O-acetylserine sulfhydrylase
O-acetylhomoserine/O-acetylserine sulfhydrylase
cytoplasmic membrane protein
cytoplasmic membrane protein
cytoplasmic membrane protein
hypothetical protein LA_2066
hypothetical protein LA_2066
hypothetical protein LA_2066
hypothetical protein LA_2066
hypothetical protein LA_2066
hypothetical protein LA_2066
hypothetical protein LA_2066
hypothetical protein LA_2066
hypothetical protein LA_2066
hypothetical protein LA_2066
hypothetical protein LA_2066
hypothetical protein LA_2066
hypothetical protein LA_2066
hypothetical protein LA_2066
HflC membrane associated protease
HflC membrane associated protease
HflC membrane associated protease
HflC membrane associated protease
HflC membrane associated protease
HflC membrane associated protease
HflC membrane associated protease
peptidyl-prolyl cis-trans isomerase
peptidyl-prolyl cis-trans isomerase
peptidyl-prolyl cis-trans isomerase
peptidyl-prolyl cis-trans isomerase
peptidyl-prolyl cis-trans isomerase
peptidyl-prolyl cis-trans isomerase
peptidyl-prolyl cis-trans isomerase
peptidyl-prolyl cis-trans isomerase
hypothetical protein LA_2078
flagellar motor switch protein FliM
glycosyltransferase
glycosyltransferase
ABC transporter ATP-binding protein
isopropylmalate isomerase large subunit
isopropylmalate isomerase large subunit
isopropylmalate isomerase large subunit
isopropylmalate isomerase large subunit
isopropylmalate isomerase large subunit

isopropylmalate isomerase large subunit
isopropylmalate isomerase large subunit
isopropylmalate isomerase large subunit
isopropylmalate isomerase large subunit
isopropylmalate isomerase large subunit
3-isopropylmalate dehydratase small subunit
3-isopropylmalate dehydratase small subunit
3-isopropylmalate dehydratase small subunit
3-isopropylmalate dehydratase small subunit
3-isopropylmalate dehydratase small subunit
3-isopropylmalate dehydratase small subunit
3-isopropylmalate dehydratase small subunit
cysteine synthase
cysteine synthase
cysteine synthase
cysteine synthase
cysteine synthase
cysteine synthase
cysteine synthase
gamma-glutamyltranspeptidase
gamma-glutamyltranspeptidase
gamma-glutamyltranspeptidase
gamma-glutamyltranspeptidase
ATP-grasp enzyme
ATP-grasp enzyme
ATP-grasp enzyme
ATP-grasp enzyme
glutaredoxin-like protein
glutaredoxin-like protein
BolA family protein
ABC transporter ATP-binding protein
ABC transporter ATP-binding protein
dihydrolipoamide dehydrogenase
metal-dependent hydrolase
metal-dependent hydrolase
glycerol kinase
glycerol kinase
glycerol kinase
glycerol kinase
glycerol kinase
glycerol kinase
hypothetical protein LA_2121
periplasmic serine protease
periplasmic serine protease
periplasmic serine protease
hypothetical protein LA_2127
DNA or RNA helicase
DNA or RNA helicase

DNA or RNA helicase
DNA or RNA helicase
DNA or RNA helicase
DNA or RNA helicase
DNA or RNA helicase
DNA or RNA helicase
DNA or RNA helicase
DNA or RNA helicase
DNA or RNA helicase
DNA or RNA helicase
DNA or RNA helicase
aspartate aminotransferase
aspartate aminotransferase
aspartate aminotransferase
aspartate aminotransferase
aspartate aminotransferase
aspartate aminotransferase
aspartate aminotransferase
aspartate aminotransferase
hydrolase
hydrolase
hydrolase
hypothetical protein LA_2136
oxidoreductase
oxidoreductase
oxidoreductase
oxidoreductase
oxidoreductase
oxidoreductase
oxidoreductase
oxidoreductase
oxidoreductase
oxidoreductase
oxidoreductase
oxidoreductase
oxidoreductase
malate dehydrogenase
malate dehydrogenase
malate dehydrogenase
malate dehydrogenase
malate dehydrogenase
malate dehydrogenase
malate dehydrogenase
malate dehydrogenase
malate dehydrogenase
malate dehydrogenase
malate dehydrogenase
malate dehydrogenase

malate dehydrogenase
malate dehydrogenase
malate dehydrogenase
malate dehydrogenase
8-amino-7-oxononanoate synthase
8-amino-7-oxononanoate synthase
dethiobiotin synthetase
dethiobiotin synthetase
adenosylmethionine-8-amino-7-oxononanoate aminotransferase
adenosylmethionine-8-amino-7-oxononanoate aminotransferase
adenosylmethionine-8-amino-7-oxononanoate aminotransferase
adenosylmethionine-8-amino-7-oxononanoate aminotransferase
DNA mismatch repair protein MutS
DNA mismatch repair protein MutS
DNA mismatch repair protein MutS
DNA mismatch repair protein MutS
DNA mismatch repair protein MutS
DNA mismatch repair protein MutS
DNA mismatch repair protein MutS
hypothetical protein LA_2150
two-component system signal receiver
two-component system signal receiver
two-component system signal receiver
two-component system signal receiver
isocitrate/isopropylmalate dehydrogenase
isocitrate/isopropylmalate dehydrogenase
isocitrate/isopropylmalate dehydrogenase
isocitrate/isopropylmalate dehydrogenase
isocitrate/isopropylmalate dehydrogenase
isocitrate/isopropylmalate dehydrogenase
acetylornithine aminotransferase
acetylornithine aminotransferase
acetylornithine aminotransferase
acetylornithine aminotransferase
acetylornithine aminotransferase
acetylornithine aminotransferase
acetylornithine aminotransferase
phosphomannomutase
phosphomannomutase
phosphomannomutase
phosphomannomutase
50S ribosomal protein L28
50S ribosomal protein L28
50S ribosomal protein L28
acyl-CoA hydrolase
acyl-CoA hydrolase
glycosyltransferase
glycosyltransferase

lipopolysaccharide core biosynthesis protein
lipopolysaccharide core biosynthesis protein
long-chain-fatty-acid CoA ligase
long-chain-fatty-acid CoA ligase
long-chain-fatty-acid CoA ligase
N-acetyl-gamma-glutamyl-phosphate reductase
N-acetyl-gamma-glutamyl-phosphate reductase
N-acetyl-gamma-glutamyl-phosphate reductase
N-acetyl-gamma-glutamyl-phosphate reductase
N-acetyl-gamma-glutamyl-phosphate reductase
recombinase A
recombinase A
recombinase A
recombinase A
recombinase A
recombinase A
recombinase A
recombinase A
recombinase A
recombinase A
recombinase A
Zn-dependent protease
Zn-dependent protease
Zn-dependent protease
Zn-dependent protease
Zn-dependent protease
hypothetical protein LA_2186
hypothetical protein LA_2186
hypothetical protein LA_2186
hypothetical protein LA_2186
hypothetical protein LA_2186
hypothetical protein LA_2186
hypothetical protein LA_2186
hypothetical protein LA_2186
hypothetical protein LA_2186
DNA helicase UvrD
2,4-dienoyl-CoA reductase
2,4-dienoyl-CoA reductase
2,4-dienoyl-CoA reductase
2,4-dienoyl-CoA reductase
2,4-dienoyl-CoA reductase
2,4-dienoyl-CoA reductase
2,4-dienoyl-CoA reductase
2,4-dienoyl-CoA reductase
2,4-dienoyl-CoA reductase
2,4-dienoyl-CoA reductase
2,4-dienoyl-CoA reductase
2,4-dienoyl-CoA reductase

2,4-dienoyl-CoA reductase
2,4-dienoyl-CoA reductase
isopropylmalate/homocitrate/citramalate synthase
isopropylmalate/homocitrate/citramalate synthase
isopropylmalate/homocitrate/citramalate synthase
isopropylmalate/homocitrate/citramalate synthase
isopropylmalate/homocitrate/citramalate synthase
isopropylmalate/homocitrate/citramalate synthase
isopropylmalate/homocitrate/citramalate synthase
isopropylmalate/homocitrate/citramalate synthase
isopropylmalate/homocitrate/citramalate synthase
isopropylmalate/homocitrate/citramalate synthase
isopropylmalate/homocitrate/citramalate synthase
isopropylmalate/homocitrate/citramalate synthase
isopropylmalate/homocitrate/citramalate synthase
periplasmic serine protease
periplasmic serine protease
periplasmic serine protease
periplasmic serine protease
periplasmic serine protease
periplasmic serine protease
periplasmic serine protease
excinuclease ABC subunit A
excinuclease ABC subunit A
excinuclease ABC subunit A
excinuclease ABC subunit A
excinuclease ABC subunit A
excinuclease ABC subunit A
excinuclease ABC subunit A
excinuclease ABC subunit A
excinuclease ABC subunit A
two component system sensor histidine kinase\response regulator
two component system sensor histidine kinase\response regulator
two component system sensor histidine kinase\response regulator
two component system sensor histidine kinase\response regulator
two component system sensor histidine kinase\response regulator
two component system sensor histidine kinase\response regulator
two component system sensor histidine kinase\response regulator
two component system sensor histidine kinase\response regulator
two component system sensor histidine kinase\response regulator
two component system sensor histidine kinase\response regulator
two component system sensor histidine kinase
two component system sensor histidine kinase
fructose-1,6-bisphosphatase
fructose-1,6-bisphosphatase
fructose-1,6-bisphosphatase
fructose-1,6-bisphosphatase
fructose-1,6-bisphosphatase

fructose-1,6-bisphosphatase
fructose-1,6-bisphosphatase
fructose-1,6-bisphosphatase
hypothetical protein LA_2230
DNA primase
DNA primase
DNA primase
RNA polymerase sigma-70 factor
RNA polymerase sigma-70 factor
RNA polymerase sigma-70 factor
RNA polymerase sigma-70 factor
RNA polymerase sigma-70 factor
RNA polymerase sigma-70 factor
RNA polymerase sigma-70 factor
RNA polymerase sigma-70 factor
RNA polymerase sigma-70 factor
RNA polymerase sigma-70 factor
RNA polymerase sigma-70 factor
RNA polymerase sigma-70 factor
RNA polymerase sigma-70 factor
glycerol-3-phosphate dehydrogenase
glycerol-3-phosphate dehydrogenase
glycerol-3-phosphate dehydrogenase
glycerol-3-phosphate dehydrogenase
glycerol-3-phosphate dehydrogenase
glycerol-3-phosphate dehydrogenase
glycerol-3-phosphate dehydrogenase
glycerol-3-phosphate dehydrogenase
tyrosyl-tRNA synthetase
hypothetical protein LA_2244
hypothetical protein LA_2244
hypothetical protein LA_2244
hypothetical protein LA_2244
hypothetical protein LA_2244
hypothetical protein LA_2244
hypothetical protein LA_2244
DJ-1/PfpI family intracellular protease
DJ-1/PfpI family intracellular protease
hypothetical protein LA_2255
hypothetical protein LA_2255
hypothetical protein LA_2255
hypothetical protein LA_2255
alkylglycerone-phosphate synthase
alkylglycerone-phosphate synthase
alkylglycerone-phosphate synthase
alkylglycerone-phosphate synthase
alkylglycerone-phosphate synthase
alkylglycerone-phosphate synthase

alkylglycerone-phosphate synthase
enoyl-CoA hydratase
enoyl-CoA hydratase
enoyl-CoA hydratase
enoyl-CoA hydratase
enoyl-CoA hydratase
hypothetical protein LA_2266
TPR-repeat-containing protein
hypothetical protein LA_2268
hypothetical protein LA_2268
cysteine desulfhydrase
hypothetical protein LA_2277
endoflagellar biosynthesis chaperone
phosphoribosylglycinamide formyltransferase
bifunctional phosphoribosylaminoimidazolecarboxamide formyltransferase/IMP cyclohydrolase
bifunctional phosphoribosylaminoimidazolecarboxamide formyltransferase/IMP cyclohydrolase
bifunctional phosphoribosylaminoimidazolecarboxamide formyltransferase/IMP cyclohydrolase
bifunctional phosphoribosylaminoimidazolecarboxamide formyltransferase/IMP cyclohydrolase
bifunctional phosphoribosylaminoimidazolecarboxamide formyltransferase/IMP cyclohydrolase
bifunctional phosphoribosylaminoimidazolecarboxamide formyltransferase/IMP cyclohydrolase
bifunctional phosphoribosylaminoimidazolecarboxamide formyltransferase/IMP cyclohydrolase
bifunctional phosphoribosylaminoimidazolecarboxamide formyltransferase/IMP cyclohydrolase
bifunctional phosphoribosylaminoimidazolecarboxamide formyltransferase/IMP cyclohydrolase
translaldolase
translaldolase
translaldolase
translaldolase
translaldolase
translaldolase
translaldolase
translaldolase
translaldolase
pseudouridylate synthase
hypothetical protein LA_2297
hypothetical protein LA_2297
hypothetical protein LA_2297
hypothetical protein LA_2297
hypothetical protein LA_2300
hypothetical protein LA_2300
hypothetical protein LA_2300
hypothetical protein LA_2300
hypothetical protein LA_2301
hypothetical protein LA_2301
hypothetical protein LA_2301
hypothetical protein LA_2301
hypothetical protein LA_2301
hypothetical protein LA_2302
hypothetical protein LA_2302

hypothetical protein LA_2302
short chain dehydrogenase
short chain dehydrogenase
long-chain-fatty-acid CoA ligase
long-chain-fatty-acid CoA ligase
long-chain-fatty-acid CoA ligase
long-chain-fatty-acid CoA ligase
long-chain-fatty-acid CoA ligase
long-chain-fatty-acid CoA ligase
long-chain-fatty-acid CoA ligase
long-chain-fatty-acid CoA ligase
long-chain-fatty-acid CoA ligase
long-chain-fatty-acid CoA ligase
long-chain-fatty-acid CoA ligase
long-chain-fatty-acid CoA ligase
long-chain-fatty-acid CoA ligase
long-chain-fatty-acid CoA ligase
long-chain-fatty-acid CoA ligase
long-chain-fatty-acid CoA ligase
mannosyltransferase
inositol monophosphatase family protein
inositol monophosphatase family protein
hypothetical protein LA_2316
hypothetical protein LA_2316
hypothetical protein LA_2316
hypothetical protein LA_2316
ExbD-like biopolymer transport protein
biopolymer transport protein TolQ
biopolymer transport protein TolQ
biopolymer transport protein TolQ
biopolymer transport protein TolQ
hypothetical protein LA_2323
hypothetical protein LA_2323
hypothetical protein LA_2323
3-ketoacyl-(acyl-carrier-protein) reductase
3-ketoacyl-(acyl-carrier-protein) reductase
3-ketoacyl-(acyl-carrier-protein) reductase
3-ketoacyl-(acyl-carrier-protein) reductase
3-ketoacyl-(acyl-carrier-protein) reductase
3-ketoacyl-(acyl-carrier-protein) reductase
transcriptional regulator
transcriptional regulator
transcriptional regulator
transcriptional regulator
transcriptional regulator
transcriptional regulator
transcriptional regulator
transcriptional regulator

transcriptional regulator
transcriptional regulator
transcriptional regulator
3-polyprenyl-4-hydroxybenzoate decarboxylase
3-polyprenyl-4-hydroxybenzoate decarboxylase
3-polyprenyl-4-hydroxybenzoate decarboxylase
A/G specific adenine glycosylase
A/G specific adenine glycosylase
GTP-dependent nucleic acid-binding protein EngD
GTP-dependent nucleic acid-binding protein EngD
GTP-dependent nucleic acid-binding protein EngD
GTP-dependent nucleic acid-binding protein EngD
GTP-dependent nucleic acid-binding protein EngD
GTP-dependent nucleic acid-binding protein EngD
3-polyprenyl-4-hydroxybenzoate decarboxylase
3-polyprenyl-4-hydroxybenzoate decarboxylase
3-polyprenyl-4-hydroxybenzoate decarboxylase
hypothetical protein LA_2336
chromosome partitioning ATPase
chromosome partitioning ATPase
chromosome partitioning ATPase
chromosome partitioning ATPase
chromosome partitioning ATPase
biotin-requiring enzyme
hypothetical protein LA_2340
ATP-dependent protease ATP-binding subunit
ATP-dependent protease ATP-binding subunit
ATP-dependent protease ATP-binding subunit
ATP-dependent protease ATP-binding subunit
ATP-dependent protease ATP-binding subunit
ATP-dependent protease ATP-binding subunit
ATP-dependent protease ATP-binding subunit
ATP-dependent protease peptidase subunit
ATP-dependent protease peptidase subunit
ATP-dependent protease peptidase subunit
ATP-dependent protease peptidase subunit
peptidyl-tRNA hydrolase
peptidyl-tRNA hydrolase
peptidyl-tRNA hydrolase
isopropylmalate/homocitrate/citramalate synthase
isopropylmalate/homocitrate/citramalate synthase
isopropylmalate/homocitrate/citramalate synthase
isopropylmalate/homocitrate/citramalate synthase
isopropylmalate/homocitrate/citramalate synthase
hypothetical protein LA_2352
hypothetical protein LA_2352
hypothetical protein LA_2352
hypothetical protein LA_2352

permease
exodeoxyribonuclease VII small subunit
exodeoxyribonuclease VII small subunit
hypothetical protein LA_2365
hypothetical protein LA_2365
hypothetical protein LA_2366
actin-like ATPase involved in cell division
actin-like ATPase involved in cell division
actin-like ATPase involved in cell division
actin-like ATPase involved in cell division
actin-like ATPase involved in cell division
actin-like ATPase involved in cell division
actin-like ATPase involved in cell division
actin-like ATPase involved in cell division
type II secretory pathway component protein G
type II secretory pathway component protein F
type II secretory pathway component protein F
type II secretory pathway ATPase protein E
type II secretory pathway ATPase protein E
type II secretory pathway ATPase protein E
type II secretory pathway ATPase protein E
type II secretory pathway ATPase protein E
type II secretory pathway ATPase protein E
type II secretory pathway component protein C
type II secretory pathway component protein C
type II secretory pathway component protein C
type II secretory pathway component protein C
type II secretory pathway component protein C
response regulator with HD-GYP domain
response regulator with HD-GYP domain
response regulator with HD-GYP domain
response regulator with HD-GYP domain
response regulator with HD-GYP domain
response regulator with HD-GYP domain
response regulator with HD-GYP domain
response regulator with HD-GYP domain
response regulator with HD-GYP domain
50S ribosomal protein L19
50S ribosomal protein L19
50S ribosomal protein L19
50S ribosomal protein L19
50S ribosomal protein L19
50S ribosomal protein L19
tRNA (guanine-N1)-methyltransferase
tRNA (guanine-N1)-methyltransferase
tRNA (guanine-N1)-methyltransferase
RNA-binding protein
RNA-binding protein

ribulose-5-phosphate 3-epimerase
ribulose-5-phosphate 3-epimerase
ribulose-5-phosphate 3-epimerase
hypothetical protein LA_2395
hypothetical protein LA_2395
hypothetical protein LA_2395
hypothetical protein LA_2395
hypothetical protein LA_2395
hypothetical protein LA_2395
hypothetical protein LA_2395
hypothetical protein LA_2395
primosomal protein N
primosomal protein N
hypothetical protein LA_2399
hypothetical protein LA_2399
response regulator of two component system
response regulator of two component system
HPr kinase/phosphorylase
HPr kinase/phosphorylase
HPr kinase/phosphorylase
DNA-directed RNA polymerase sigma-54 subunit
DNA-directed RNA polymerase sigma-54 subunit
DNA-directed RNA polymerase sigma-54 subunit
DNA-directed RNA polymerase sigma-54 subunit
hypothetical protein LA_2406
hypothetical protein LA_2406
glycerol-3-phosphate cytidylyltransferase
ADP-heptose synthetase
ADP-heptose synthetase
ADP-heptose synthetase
ADP-heptose synthetase
ADP-heptose synthetase
cell wall-associated hydrolase/lipoprotein
cell wall-associated hydrolase/lipoprotein
cobalamin adenosyltransferase
endoflagellar filament core protein
endoflagellar filament core protein
endoflagellar filament core protein
endoflagellar filament core protein
endoflagellar filament core protein
endoflagellar filament core protein
endoflagellar filament core protein
endoflagellar filament core protein
biotin carboxylase-like protein
biotin carboxylase-like protein
biotin carboxylase-like protein
biotin carboxylase-like protein
biotin carboxylase-like protein

biotin carboxylase-like protein
biotin carboxylase-like protein
biotin carboxylase-like protein
biotin carboxylase-like protein
biotin carboxylase-like protein
biotin carboxylase-like protein
biotin carboxylase-like protein
biotin carboxylase-like protein
biotin carboxylase-like protein
biotin carboxylase-like protein
biotin carboxylase-like protein
biotin carboxylase-like protein
biotin carboxylase-like protein
biotin carboxylase-like protein
biotin carboxylase-like protein
biotin carboxylase-like protein
biotin carboxylase-like protein
biotin carboxylase-like protein
biotin carboxylase-like protein
biotin carboxylase-like protein
biotin carboxylase-like protein
biotin carboxylase-like protein
acetyl-CoA carboxylase carboxyltransferase component
acetyl-CoA carboxylase carboxyltransferase component
acetyl-CoA carboxylase carboxyltransferase component
acetyl-CoA carboxylase carboxyltransferase component
acetyl-CoA carboxylase carboxyltransferase component
acetyl-CoA carboxylase carboxyltransferase component
acetyl-CoA carboxylase carboxyltransferase component
acetyl-CoA carboxylase carboxyltransferase component
acetyl-CoA carboxylase carboxyltransferase component
acetyl-CoA carboxylase carboxyltransferase component
acetyl-CoA carboxylase carboxyltransferase component
acetyl-CoA carboxylase carboxyltransferase component
acetyl-CoA carboxylase carboxyltransferase component
acetyl-CoA carboxylase carboxyltransferase component
acetyl-CoA carboxylase carboxyltransferase component
acetyl-CoA carboxylase carboxyltransferase component
anti-sigma factor antagonist
serine/threonine phosphatase
peptide deformylase
peptide deformylase
peptide deformylase
peptide deformylase
peptide deformylase
heavy metal efflux pump
heavy metal efflux pump
heavy metal efflux pump

heavy metal efflux pump
heavy metal efflux pump
heavy metal efflux pump
heavy metal efflux pump
hypothetical protein LA_2456
SMC-like protein
anti-Sigma factor antagonist
hypothetical protein LA_2465
methyltransferase
hypothetical protein LA_2467
hypothetical protein LA_2467
hypothetical protein LA_2467
hypothetical protein LA_2468
hypothetical protein LA_2468
hypothetical protein LA_2468
hypothetical protein LA_2468
hypothetical protein LA_2468
hypothetical protein LA_2468
hypothetical protein LA_2468
MCP methylation inhibitor
MCP methylation inhibitor
MCP methylation inhibitor
integrase/recombinase XerD
integrase/recombinase XerD
hypothetical protein LA_2484
hypothetical protein LA_2484
thioredoxin reductase
thioredoxin reductase
thioredoxin reductase
imidazoleglycerol-phosphate synthase
imidazoleglycerol-phosphate synthase
imidazoleglycerol-phosphate synthase
imidazoleglycerol-phosphate synthase
imidazoleglycerol-phosphate synthase
imidazoleglycerol-phosphate synthase
imidazoleglycerol-phosphate synthase
aspartyl/glutamyl-tRNA amidotransferase subunit A
aspartyl/glutamyl-tRNA amidotransferase subunit A
aspartyl/glutamyl-tRNA amidotransferase subunit A
aspartyl/glutamyl-tRNA amidotransferase subunit A
aspartyl/glutamyl-tRNA amidotransferase subunit A
aspartyl/glutamyl-tRNA amidotransferase subunit A
aspartyl/glutamyl-tRNA amidotransferase subunit A
hypothetical protein LA_2510
hypothetical protein LA_2510
cytoplasmic membrane lipoprotein LipL31
cytoplasmic membrane lipoprotein LipL31
cytoplasmic membrane lipoprotein LipL31

cytoplasmic membrane lipoprotein LipL31
cytoplasmic membrane lipoprotein LipL31
cytoplasmic membrane lipoprotein LipL31
cytoplasmic membrane lipoprotein LipL31
cytoplasmic membrane lipoprotein LipL31
cytoplasmic membrane lipoprotein LipL31
transcription-repair coupling factor
transcription-repair coupling factor
transcription-repair coupling factor
transcription-repair coupling factor
pantoate--beta-alanine ligase
histidinol dehydrogenase
histidinol dehydrogenase
histidinol dehydrogenase
histidinol dehydrogenase
histidinol dehydrogenase
hydrolase
hydrolase
hydrolase
GGDEF domain receiver component of a two component response regulator
GGDEF domain receiver component of a two component response regulator
GGDEF domain receiver component of a two component response regulator
hypothetical protein LA_2530
peptidyl-prolyl cis-trans isomerase
peptidyl-prolyl cis-trans isomerase
peptidyl-prolyl cis-trans isomerase
peptidyl-prolyl cis-trans isomerase
peptidyl-prolyl cis-trans isomerase
peptidyl-prolyl cis-trans isomerase
adenylate/guanylate cyclase
adenylate/guanylate cyclase
adenylate/guanylate cyclase
adenylate/guanylate cyclase
adenylate/guanylate cyclase
adenylate/guanylate cyclase
adenylate/guanylate cyclase
hypothetical protein LA_2537
hypothetical protein LA_2537
ABC transporter ATPase
ABC transporter ATPase
cAMP-binding protein
cAMP-binding protein
cAMP-binding protein
cAMP-binding protein
cAMP-binding protein
cAMP-binding protein
cAMP-binding protein
alpha/beta hydrolase superfamily protein

FKBP-type peptidyl-prolyl cis-trans isomerase
FKBP-type peptidyl-prolyl cis-trans isomerase
FKBP-type peptidyl-prolyl cis-trans isomerase
FKBP-type peptidyl-prolyl cis-trans isomerase
FKBP-type peptidyl-prolyl cis-trans isomerase
FKBP-type peptidyl-prolyl cis-trans isomerase
RNA-binding protein
RNA-binding protein
ATP-dependent protease ATP-binding subunit
ATP-dependent protease ATP-binding subunit
ATP-dependent protease ATP-binding subunit
ATP-dependent protease ATP-binding subunit
ATP-dependent protease ATP-binding subunit
ATP-dependent protease ATP-binding subunit
ATP-dependent protease ATP-binding subunit
ATP-dependent protease ATP-binding subunit
ATP-dependent protease ATP-binding subunit
ATP-dependent protease ClpP
ATP-dependent protease ClpP
ATP-dependent protease ClpP
ATP-dependent protease ClpP
ATP-dependent protease ClpP
ATP-dependent protease ClpP
ATP-dependent protease ClpP
FKBP-type peptidyl-prolyl cis-trans isomerase
FKBP-type peptidyl-prolyl cis-trans isomerase
FKBP-type peptidyl-prolyl cis-trans isomerase
FKBP-type peptidyl-prolyl cis-trans isomerase
FKBP-type peptidyl-prolyl cis-trans isomerase
FKBP-type peptidyl-prolyl cis-trans isomerase
FKBP-type peptidyl-prolyl cis-trans isomerase
FKBP-type peptidyl-prolyl cis-trans isomerase
FKBP-type peptidyl-prolyl cis-trans isomerase
FKBP-type peptidyl-prolyl cis-trans isomerase
FKBP-type peptidyl-prolyl cis-trans isomerase
FKBP-type peptidyl-prolyl cis-trans isomerase
FKBP-type peptidyl-prolyl cis-trans isomerase
FKBP-type peptidyl-prolyl cis-trans isomerase
FKBP-type peptidyl-prolyl cis-trans isomerase
glycosylhydrolase
glycosylhydrolase
glycosylhydrolase
glycosylhydrolase
glycosylhydrolase
glycosylhydrolase
glycosylhydrolase
acetolactate synthase small subunit
acetolactate synthase small subunit

acetolactate synthase small subunit
acetolactate synthase large subunit
acetolactate synthase large subunit
acetolactate synthase large subunit
acetolactate synthase large subunit
acetolactate synthase large subunit
acetolactate synthase large subunit
acetolactate synthase large subunit
acetolactate synthase large subunit
acetolactate synthase large subunit
acetolactate synthase large subunit
TPR-repeat-containing protein
TPR-repeat-containing protein
TPR-repeat-containing protein
TPR-repeat-containing protein
TPR-repeat-containing protein
TPR-repeat-containing protein
TPR-repeat-containing protein
TPR-repeat-containing protein
hypothetical protein LA_2575
hypothetical protein LA_2575
hypothetical protein LA_2575
hypothetical protein LA_2576
hypothetical protein LA_2576
hypothetical protein LA_2576
hypothetical protein LA_2576
hypothetical protein LA_2576
hypothetical protein LA_2576
hypothetical protein LA_2576
hypothetical protein LA_2576
hypothetical protein LA_2576
hypothetical protein LA_2576
hypothetical protein LA_2576
hypothetical protein LA_2576
hypothetical protein LA_2576
hypothetical protein LA_2576
ferrous iron transport protein B
N-acetylneuraminic (sialic) acid synthetase
hypothetical protein LA_2584
hypothetical protein LA_2584
flagellar assembly protein H
flagellar switch complex protein
flagellar switch complex protein
flagellar MS-ring protein
flagellar MS-ring protein
flagellar MS-ring protein
flagellar protein FlbB
hypothetical protein LA_2596

polymerase
polymerase
RNA polymerase sigma factor WhiG
endoflagellar biogenesis protein
endoflagellar motor switch protein
endoflagellar motor switch protein
endoflagellar motor switch protein
glutathione transferase
glutathione transferase
oligopeptidase A
oligopeptidase A
MaoC family protein
MaoC family protein
MaoC family protein
MaoC family protein
MaoC family protein
MaoC family protein
MaoC family protein
MaoC family protein
MaoC family protein
MaoC family protein
MaoC family protein
alcohol dehydrogenase
hypothetical protein LA_2632
hypothetical protein LA_2632
hypothetical protein LA_2632
hypothetical protein LA_2632
hypothetical protein LA_2632
hypothetical protein LA_2632
hypothetical protein LA_2637
hypothetical protein LA_2637
hypothetical protein LA_2637
hypothetical protein LA_2637
hypothetical protein LA_2637
hypothetical protein LA_2637
hypothetical protein LA_2637
hypothetical protein LA_2637
hypothetical protein LA_2637
hypothetical protein LA_2637
hypothetical protein LA_2637
hypothetical protein LA_2637
hypothetical protein LA_2637
hypothetical protein LA_2637
hypothetical protein LA_2637
hypothetical protein LA_2637
hypothetical protein LA_2637
hypothetical protein LA_2637
acyl-CoA dehydrogenase

acyl-CoA dehydrogenase
acyl-CoA dehydrogenase
nucleoside-diphosphate-sugar epimerase
nucleoside-diphosphate-sugar epimerase
nucleoside-diphosphate-sugar epimerase
nucleoside-diphosphate-sugar epimerase
hypothetical protein LA_2648
co-chaperonin GroES
co-chaperonin GroES
co-chaperonin GroES
co-chaperonin GroES
co-chaperonin GroES
co-chaperonin GroES
co-chaperonin GroES
co-chaperonin GroES
chaperonin GroEL
chaperonin GroEL
chaperonin GroEL
chaperonin GroEL
chaperonin GroEL
chaperonin GroEL
chaperonin GroEL
chaperonin GroEL
chaperonin GroEL
chaperonin GroEL
chaperonin GroEL
chaperonin GroEL
chaperonin GroEL
chaperonin GroEL
chaperonin GroEL
chaperonin GroEL
chaperonin GroEL
chaperonin GroEL
chaperonin GroEL
chaperonin GroEL
chaperonin GroEL
chaperonin GroEL
chaperonin GroEL
chaperonin GroEL
chaperonin GroEL
chaperonin GroEL
chaperonin GroEL
chaperonin GroEL
chaperonin GroEL
chaperonin GroEL
chaperonin GroEL
chaperonin GroEL
chaperonin GroEL

chaperonin GroEL
chaperonin GroEL
chaperonin GroEL
chaperonin GroEL
chaperonin GroEL
chaperonin GroEL
chaperonin GroEL
chaperonin GroEL
chaperonin GroEL
ribonuclease BN
ribonuclease BN
ribonuclease BN
flagellar basal body P-ring protein
flagellar basal body P-ring protein
flagellar basal body P-ring protein
flagellar basal body P-ring protein
hypothetical protein LA_2672
hypothetical protein LA_2672
metal-dependent amidase/aminoacylase/carboxypeptidase
metal-dependent amidase/aminoacylase/carboxypeptidase
metal-dependent amidase/aminoacylase/carboxypeptidase
metal-dependent amidase/aminoacylase/carboxypeptidase
aminopeptidase P
aminopeptidase P
aminopeptidase P
aminopeptidase P
aminopeptidase P
acetyl-CoA acetyltransferase
acetyl-CoA acetyltransferase
acetyl-CoA acetyltransferase
acetyl-CoA acetyltransferase
acetyl-CoA acetyltransferase
acetyl-CoA acetyltransferase
acetyl-CoA acetyltransferase
acetyl-CoA acetyltransferase
N6-adenine-specific DNA methylase
N6-adenine-specific DNA methylase
N6-adenine-specific DNA methylase
N6-adenine-specific DNA methylase
N6-adenine-specific DNA methylase
N6-adenine-specific DNA methylase
N6-adenine-specific DNA methylase
acetylglutamate kinase
D-alanine--D-alanine ligase
L-lysine 2,3-aminomutase
phosphoglycolate phosphatase
phosphoglycolate phosphatase
phosphoglycolate phosphatase

phosphoglycolate phosphatase
phosphoglycolate phosphatase
phosphoglycolate phosphatase
thiamine biosynthesis enzyme
thiamine biosynthesis enzyme
thiamine biosynthesis enzyme
hypothetical protein LA_2719
hypothetical protein LA_2719
hypothetical protein LA_2719
dioxygenase
dioxygenase
peptide chain release factor 3
peptide chain release factor 3
peptide chain release factor 3
peptide chain release factor 3
peptide chain release factor 3
acyltransferase
acyltransferase
acyltransferase
hypothetical protein LA_2738
hypothetical protein LA_2738
hypothetical protein LA_2738
hypothetical protein LA_2738
hypothetical protein LA_2738
hypothetical protein LA_2738
hypothetical protein LA_2738
hypothetical protein LA_2738
hypothetical protein LA_2738
hypothetical protein LA_2738
hypothetical protein LA_2738
pseudouridylate synthase
hypothetical protein LA_2741
sulfate ABC transporter substrate-binding protein
sulfate ABC transporter substrate-binding protein
sulfate ABC transporter substrate-binding protein
sulfate ABC transporter substrate-binding protein
sulfate ABC transporter substrate-binding protein
sulfate ABC transporter substrate-binding protein
6-pyruvoyl-tetrahydropterin synthase
6-pyruvoyl-tetrahydropterin synthase
6-pyruvoyl-tetrahydropterin synthase
transpeptidase/penicillin binding protein
transpeptidase/penicillin binding protein
transpeptidase/penicillin binding protein
rod shape-determining protein MreB
rod shape-determining protein MreB
rod shape-determining protein MreB
rod shape-determining protein MreB

rod shape-determining protein MreB
rod shape-determining protein MreB
rod shape-determining protein MreB
exopolyphosphatase
exopolyphosphatase
exopolyphosphatase
exopolyphosphatase
exopolyphosphatase
exopolyphosphatase
F0F1 ATP synthase subunit epsilon
F0F1 ATP synthase subunit epsilon
F0F1 ATP synthase subunit epsilon
F0F1 ATP synthase subunit delta
F0F1 ATP synthase subunit delta
F0F1 ATP synthase subunit delta
F0F1 ATP synthase subunit delta
F0F1 ATP synthase subunit delta
F0F1 ATP synthase subunit delta
F0F1 ATP synthase subunit B
F0F1 ATP synthase subunit B
signal peptidase I
transcriptional regulator
ABC transporter permease
ABC transporter permease
peroxiredoxin
peroxiredoxin
peroxiredoxin
peroxiredoxin
peroxiredoxin
peroxiredoxin
hypothetical protein LA_2821
hypothetical protein LA_2821
hypothetical protein LA_2821
hypothetical protein LA_2826
signal transduction protein
two-component system signal receiver
two-component system signal receiver
adenylate/guanylate cyclase
adenylate/guanylate cyclase
adenylate/guanylate cyclase
adenylate/guanylate cyclase
adenylate/guanylate cyclase
adenylate/guanylate cyclase
hypothetical protein LA_2836
hypothetical protein LA_2836
hypothetical protein LA_2836
hypothetical protein LA_2836
hypothetical protein LA_2836

ornithine carbamoyltransferase
ornithine carbamoyltransferase
ornithine carbamoyltransferase
response regulator with HD-GYP domain
response regulator with HD-GYP domain
response regulator with HD-GYP domain
response regulator with HD-GYP domain
flagellar hook protein FlgE
flagellar hook protein FlgE
flagellar hook protein FlgE
flagellar hook protein FlgE
flagellar hook protein FlgE
hypothetical protein LA_2859
cytoplasmic membrane protein
cytoplasmic membrane protein
translation initiation factor 2B
translation initiation factor 2B
translation initiation factor 2B
translation initiation factor 2B
translation initiation factor 2B
translation initiation factor 2B
polymerase
hypothetical protein LA_2877
transcriptional regulator
transcriptional regulator
transcriptional regulator
Holliday junction DNA helicase RuvA
Holliday junction DNA helicase RuvA
Holliday junction DNA helicase RuvA
Holliday junction DNA helicase RuvA
flavin reductase-like protein
flavin reductase-like protein
flavin reductase-like protein
ribonuclease D
ribonuclease D
ribonuclease D
alpha-galactosidase
alpha-galactosidase
alpha-galactosidase
alpha-galactosidase
alpha-galactosidase
alpha-galactosidase
alpha-galactosidase
two-component response regulator
two-component response regulator
GGDEF family protein
GGDEF family protein
hypothetical protein LA_2941

serine protease
serine protease
serine protease
serine protease
serine protease
hypothetical protein LA_2950
hypothetical protein LA_2950
two-component hybrid sensor and regulator
two-component hybrid sensor and regulator
two-component hybrid sensor and regulator
acyltransferase
acyltransferase
acyltransferase
methylmalonyl-CoA mutase
methylmalonyl-CoA mutase
methylmalonyl-CoA mutase
methylmalonyl-CoA mutase
methylmalonyl-CoA mutase
dihydroxy-acid dehydratase
dihydroxy-acid dehydratase
dihydroxy-acid dehydratase
dihydroxy-acid dehydratase
dihydroxy-acid dehydratase
dihydroxy-acid dehydratase
dihydroxy-acid dehydratase
dihydroxy-acid dehydratase
dihydroxy-acid dehydratase
hypothetical protein LA_2967
hypothetical protein LA_2967
adenylate/guanylate cyclase
adenylate/guanylate cyclase
adenylate/guanylate cyclase
adenylate/guanylate cyclase
adenylate/guanylate cyclase
adenylate/guanylate cyclase
adenylate/guanylate cyclase
adenylate/guanylate cyclase
hypothetical protein LA_2973
hypothetical protein LA_2973
hypothetical protein LA_2986
uridine 5-monophosphate synthase
uridine 5-monophosphate synthase
NH(3)-dependent NAD(+) synthetase
NH(3)-dependent NAD(+) synthetase
NH(3)-dependent NAD(+) synthetase
hypothetical protein LA_2992
hypothetical protein LA_2992
hypothetical protein LA_2995

alcohol dehydrogenase
single-stranded DNA exonuclease
single-stranded DNA exonuclease
single-stranded DNA exonuclease
tRNA nucleotidyltransferase/poly A polymerase
tRNA nucleotidyltransferase/poly A polymerase
tRNA nucleotidyltransferase/poly A polymerase
tRNA nucleotidyltransferase/poly A polymerase
tRNA nucleotidyltransferase/poly A polymerase
tRNA nucleotidyltransferase/poly A polymerase
tRNA nucleotidyltransferase/poly A polymerase
M23 family metalloendopeptidase
M23 family metalloendopeptidase
prolipoprotein diacylglyceryl transferase
prolipoprotein diacylglyceryl transferase
prolipoprotein diacylglyceryl transferase
prolipoprotein diacylglyceryl transferase
hypothetical protein LA_3028
hypothetical protein LA_3028
hypothetical protein LA_3028
hypothetical protein LA_3028
hypothetical protein LA_3028
hypothetical protein LA_3028
hypothetical protein LA_3028
hypothetical protein LA_3028
hypothetical protein LA_3028
molybdate metabolism regulator
molybdate metabolism regulator
molybdate metabolism regulator
hypothetical protein LA_3039
hypothetical protein LA_3039
hypothetical protein LA_3039
hypothetical protein LA_3039
adenylate/guanylate cyclase
adenylate/guanylate cyclase
glutamate racemase
aminotransferase
aminotransferase
aminotransferase
aminotransferase
aminotransferase
aminotransferase
aminopeptidase
hypothetical protein LA_3055
hypothetical protein LA_3069
hypothetical protein LA_3069
hypothetical protein LA_3069
anti-sigma-factor antagonist

anti-sigma-factor antagonist
anti-sigma-factor antagonist
anti-sigma-factor antagonist
glycosyltransferase
glycosyltransferase
hypothetical protein LA_3079
hypothetical protein LA_3079
adenylosuccinate lyase
adenylosuccinate lyase
adenylosuccinate lyase
adenylosuccinate lyase
adenylosuccinate lyase
adenylosuccinate lyase
adenylosuccinate lyase
adenylosuccinate lyase
adenylosuccinate lyase
nicotinate-nucleotide pyrophosphorylase
nicotinate-nucleotide pyrophosphorylase
nicotinate-nucleotide pyrophosphorylase
nicotinate-nucleotide pyrophosphorylase
nicotinate-nucleotide pyrophosphorylase
ferric uptake regulator
ferric uptake regulator
anti-sigma factor antagonist
anti-sigma factor antagonist
anti-sigma factor antagonist
anti-sigma factor antagonist
anti-sigma factor antagonist
anti-sigma factor antagonist
hypothetical protein LA_3097
hypothetical protein LA_3097
hypothetical protein LA_3097
hypothetical protein LA_3097
hypothetical protein LA_3097
hypothetical protein LA_3097
hypothetical protein LA_3097
hypothetical protein LA_3097
hypothetical protein LA_3097
hypothetical protein LA_3097
hypothetical protein LA_3097
hypothetical protein LA_3097
hypothetical protein LA_3097
hypothetical protein LA_3097
hypothetical protein LA_3097
hypothetical protein LA_3097
hypothetical protein LA_3097
hypothetical protein LA_3097
hypothetical protein LA_3097

serine/threonine kinase
serine/threonine kinase
serine/threonine kinase
hypothetical protein LA_3118
hypothetical protein LA_3118
hypothetical protein LA_3118
hypothetical protein LA_3118
hypothetical protein LA_3118
hypothetical protein LA_3118
hypothetical protein LA_3118
hypothetical protein LA_3118
hypothetical protein LA_3119
hypothetical protein LA_3119
hypothetical protein LA_3119
hypothetical protein LA_3119
zinc-bindin carboxypeptidase
zinc-bindin carboxypeptidase
zinc-bindin carboxypeptidase
acetyl-CoA acetyltransferase
acetyl-CoA acetyltransferase
acetyl-CoA acetyltransferase
acetyl-CoA acetyltransferase
acetyl-CoA acetyltransferase
acetyl-CoA acetyltransferase
acetyl-CoA acetyltransferase
acetyl-CoA acetyltransferase
acetyl-CoA acetyltransferase
acetyl-CoA acetyltransferase
acetyl-CoA acetyltransferase
acetyl-CoA acetyltransferase
acyl-CoA dehydrogenase
acyl-CoA dehydrogenase
acyl-CoA dehydrogenase
acyl-CoA dehydrogenase
acyl-CoA dehydrogenase
acyl-CoA dehydrogenase
acyl-CoA transferase/carnitine dehydratase
acyl-CoA transferase/carnitine dehydratase
acyl-CoA transferase/carnitine dehydratase
acyl-CoA transferase/carnitine dehydratase
acyl-CoA transferase/carnitine dehydratase
Zn-dependent alcohol dehydrogenase
1-hydroxy-2-methyl-2-(E)-butenyl 4-diphosphate synthase
1-hydroxy-2-methyl-2-(E)-butenyl 4-diphosphate synthase
1-hydroxy-2-methyl-2-(E)-butenyl 4-diphosphate synthase
1-hydroxy-2-methyl-2-(E)-butenyl 4-diphosphate synthase
1-hydroxy-2-methyl-2-(E)-butenyl 4-diphosphate synthase
1-hydroxy-2-methyl-2-(E)-butenyl 4-diphosphate synthase

1-hydroxy-2-methyl-2-(E)-butenyl 4-diphosphate synthase
metallophosphoesterase
aldo/keto reductase
aldo/keto reductase
ABC transporter ATPase
hypothetical protein LA_3191
fruiting body developmental protein R
fruiting body developmental protein R
type I restriction enzyme
Zn-dependent hydrolase
hypothetical protein LA_3210
hypothetical protein LA_3214
hypothetical protein LA_3214
geranylgeranyl pyrophosphate synthase
geranylgeranyl pyrophosphate synthase
hypothetical protein LA_3255
hypothetical protein LA_3262
hypothetical protein LA_3262
hypothetical protein LA_3262
hypothetical protein LA_3262
hypothetical protein LA_3263
hypothetical protein LA_3263
hypothetical protein LA_3263
hypothetical protein LA_3263
hypothetical protein LA_3263
hypothetical protein LA_3263
hypothetical protein LA_3263
hypothetical protein LA_3263
hypothetical protein LA_3264
hypothetical protein LA_3264
hypothetical protein LA_3264
hypothetical protein LA_3264
hypothetical protein LA_3264
hypothetical protein LA_3264
hypothetical protein LA_3265
hypothetical protein LA_3265
hypothetical protein LA_3265
hypothetical protein LA_3265
hypothetical protein LA_3265
hypothetical protein LA_3265
hypothetical protein LA_3265
molybdopterin oxidoreductase iron-sulfur binding subunit
molybdopterin oxidoreductase iron-sulfur binding subunit
molybdopterin oxidoreductase iron-sulfur binding subunit
molybdopterin oxidoreductase iron-sulfur binding subunit
molybdopterin oxidoreductase iron-sulfur binding subunit
molybdopterin oxidoreductase iron-sulfur binding subunit
molybdopterin oxidoreductase iron-sulfur binding subunit

molybdopterin oxidoreductase iron-sulfur binding subunit
molybdopterin oxidoreductase iron-sulfur binding subunit
molybdopterin oxidoreductase iron-sulfur binding subunit
molybdopterin oxidoreductase iron-sulfur binding subunit
molybdopterin oxidoreductase iron-sulfur binding subunit
molybdopterin oxidoreductase iron-sulfur binding subunit
molybdopterin oxidoreductase iron-sulfur binding subunit
molybdopterin oxidoreductase iron-sulfur binding subunit
molybdopterin oxidoreductase iron-sulfur binding subunit
molybdopterin oxidoreductase iron-sulfur binding subunit
molybdopterin oxidoreductase iron-sulfur binding subunit
molybdopterin oxidoreductase iron-sulfur binding subunit
molybdopterin oxidoreductase iron-sulfur binding subunit
molybdopterin oxidoreductase iron-sulfur binding subunit
molybdopterin oxidoreductase iron-sulfur binding subunit
molybdopterin oxidoreductase iron-sulfur binding subunit
molybdopterin oxidoreductase iron-sulfur binding subunit
molybdopterin oxidoreductase iron-sulfur binding subunit
molybdopterin oxidoreductase iron-sulfur binding subunit
molybdopterin oxidoreductase iron-sulfur binding subunit
molybdopterin oxidoreductase iron-sulfur binding subunit
hypothetical protein LA_3268
hypothetical protein LA_3268
hypothetical protein LA_3268
1-deoxy-D-xylulose-5-phosphate synthase
1-deoxy-D-xylulose-5-phosphate synthase
adenylate cyclase related protein
adenylate cyclase related protein
adenylate cyclase related protein
adenylate cyclase related protein
adenylate cyclase related protein
adenylate cyclase related protein
adenylate cyclase related protein
adenylate cyclase related protein
adenylate cyclase related protein
tryptophan synthase subunit alpha
tryptophan synthase subunit alpha
tryptophan synthase subunit alpha
tryptophan synthase subunit alpha
tryptophan synthase subunit beta
tryptophan synthase subunit beta
tryptophan synthase subunit beta
tryptophan synthase subunit beta
tryptophan synthase subunit beta
tryptophan synthase subunit beta
tryptophan synthase subunit beta
prolyl-tRNA synthetase
prolyl-tRNA synthetase

prolyl-tRNA synthetase
prolyl-tRNA synthetase
prolyl-tRNA synthetase
prolyl-tRNA synthetase
prolyl-tRNA synthetase
zinc metalloprotease
zinc metalloprotease
zinc metalloprotease
zinc metalloprotease
zinc metalloprotease
zinc metalloprotease
CDP-diglyceride synthetase
undecaprenyl pyrophosphate synthase
undecaprenyl pyrophosphate synthase
ribosome recycling factor
ribosome recycling factor
ribosome recycling factor
ribosome recycling factor
ribosome recycling factor
uridylate kinase
uridylate kinase
uridylate kinase
uridylate kinase
elongation factor Ts
elongation factor Ts
elongation factor Ts
elongation factor Ts
cell shape determination protein
cell shape determination protein
hypothetical protein LA_3301
hypothetical protein LA_3301
hypothetical protein LA_3303
hypothetical protein LA_3303
hypothetical protein LA_3303
hypothetical protein LA_3303
dihydrodipicolinate reductase
dihydrodipicolinate reductase
dihydrodipicolinate reductase
dihydrodipicolinate reductase
dihydrodipicolinate reductase
dihydrodipicolinate reductase
dihydrodipicolinate reductase
dihydrodipicolinate synthase
dihydrodipicolinate synthase
dihydrodipicolinate synthase
dihydrodipicolinate synthase
dihydrodipicolinate synthase
glyoxalase

glyoxalase
dihydrofolate reductase-like protein
hypothetical protein LA_3320
hypothetical protein LA_3323
cytochrome c biogenesis protein
cytochrome c biogenesis protein
cytochrome c-type biogenesis precursor
hypothetical protein LA_3352
hypothetical protein LA_3355
glutathione transferase
glutathione transferase
glutathione transferase
glutathione transferase
glutathione transferase
glutathione transferase
hypothetical protein LA_3361
aminoglycoside phosphotransferase
aminoglycoside phosphotransferase
aminoglycoside phosphotransferase
acyl-CoA dehydrogenase
acyl-CoA dehydrogenase
hydroxyethylthiazole kinase
hydroxyethylthiazole kinase
hydroxyethylthiazole kinase
hydroxyethylthiazole kinase
hydroxyethylthiazole kinase
surface antigen OrfC lipoprotein
surface antigen OrfC lipoprotein
surface antigen OrfC lipoprotein
surface antigen OrfC lipoprotein
surface antigen OrfC lipoprotein
surface antigen OrfC lipoprotein
surface antigen OrfC lipoprotein
surface antigen OrfC lipoprotein
surface antigen OrfC lipoprotein
cAMP-binding protein
cAMP-binding protein
cAMP-binding protein
cAMP-binding protein
cAMP-binding protein
endoflagellar filament sheath protein
endoflagellar filament sheath protein
endoflagellar filament sheath protein
endoflagellar filament sheath protein
endoflagellar filament sheath protein
endoflagellar filament sheath protein
endoflagellar filament sheath protein
metallo-beta-lactamase

metallo-beta-lactamase
metallo-beta-lactamase
metallo-beta-lactamase
Zn-dependent peptidase
Zn-dependent peptidase
Zn-dependent peptidase
Zn-dependent peptidase
Zn-dependent peptidase
Zn-dependent peptidase
Zn-dependent peptidase
Zn-dependent peptidase
Zn-dependent peptidase
Zn-dependent peptidase
Zn-dependent peptidase
Zn-dependent peptidase
Zn-dependent peptidase
Zn-dependent peptidase
Zn-dependent peptidase
Zn-dependent peptidase
Zn-dependent peptidase
Zn-dependent peptidase
hypothetical protein LA_3403
hypothetical protein LA_3403
nucleotide-binding protein
nucleotide-binding protein
nucleotide-binding protein
alanyl-tRNA synthetase
alanyl-tRNA synthetase
alanyl-tRNA synthetase
alanyl-tRNA synthetase
alanyl-tRNA synthetase
alanyl-tRNA synthetase
alanyl-tRNA synthetase
alanyl-tRNA synthetase
alanyl-tRNA synthetase
alanyl-tRNA synthetase
alanyl-tRNA synthetase
50S ribosomal protein L13
50S ribosomal protein L13
50S ribosomal protein L13
hypothetical protein LA_3410
hypothetical protein LA_3410
hypothetical protein LA_3410
hypothetical protein LA_3410
hypothetical protein LA_3410
hypothetical protein LA_3410
hypothetical protein LA_3410
hypothetical protein LA_3410

30S ribosomal protein S7
30S ribosomal protein S7
30S ribosomal protein S7
30S ribosomal protein S7
30S ribosomal protein S7
30S ribosomal protein S7
30S ribosomal protein S7
30S ribosomal protein S12
DNA-directed RNA polymerase subunit beta'
DNA-directed RNA polymerase subunit beta'
DNA-directed RNA polymerase subunit beta'
DNA-directed RNA polymerase subunit beta'
DNA-directed RNA polymerase subunit beta'
DNA-directed RNA polymerase subunit beta'
DNA-directed RNA polymerase subunit beta'
DNA-directed RNA polymerase subunit beta'
DNA-directed RNA polymerase subunit beta'
DNA-directed RNA polymerase subunit beta'
DNA-directed RNA polymerase subunit beta'
DNA-directed RNA polymerase subunit beta'
DNA-directed RNA polymerase subunit beta'
DNA-directed RNA polymerase subunit beta'
DNA-directed RNA polymerase subunit beta'
DNA-directed RNA polymerase subunit beta'
DNA-directed RNA polymerase subunit beta'
DNA-directed RNA polymerase subunit beta'
DNA-directed RNA polymerase subunit beta'
DNA-directed RNA polymerase subunit beta'
DNA-directed RNA polymerase subunit beta'
DNA-directed RNA polymerase subunit beta'
DNA-directed RNA polymerase subunit beta'
DNA-directed RNA polymerase subunit beta'
DNA-directed RNA polymerase subunit beta'
DNA-directed RNA polymerase subunit beta'
DNA-directed RNA polymerase subunit beta'
DNA-directed RNA polymerase subunit beta'
DNA-directed RNA polymerase subunit beta'
DNA-directed RNA polymerase subunit beta'
DNA-directed RNA polymerase subunit beta'
DNA-directed RNA polymerase subunit beta'
DNA-directed RNA polymerase subunit beta'
DNA-directed RNA polymerase subunit beta'
DNA-directed RNA polymerase subunit beta'
DNA-directed RNA polymerase subunit beta'
DNA-directed RNA polymerase subunit beta'
DNA-directed RNA polymerase subunit beta'
DNA-directed RNA polymerase subunit beta'
DNA-directed RNA polymerase subunit beta'

DNA-directed RNA polymerase subunit beta'
DNA-directed RNA polymerase subunit beta'
DNA-directed RNA polymerase subunit beta'
DNA-directed RNA polymerase subunit beta'
DNA-directed RNA polymerase subunit beta'
DNA-directed RNA polymerase subunit beta'
DNA-directed RNA polymerase subunit beta'
DNA-directed RNA polymerase subunit beta'
DNA-directed RNA polymerase subunit beta'
DNA-directed RNA polymerase subunit beta'
DNA-directed RNA polymerase subunit beta'
DNA-directed RNA polymerase subunit beta'
DNA-directed RNA polymerase subunit beta'
DNA-directed RNA polymerase subunit beta'
DNA-directed RNA polymerase subunit beta'
DNA-directed RNA polymerase subunit beta
DNA-directed RNA polymerase subunit beta
DNA-directed RNA polymerase subunit beta
DNA-directed RNA polymerase subunit beta
DNA-directed RNA polymerase subunit beta
DNA-directed RNA polymerase subunit beta
DNA-directed RNA polymerase subunit beta
DNA-directed RNA polymerase subunit beta
DNA-directed RNA polymerase subunit beta
DNA-directed RNA polymerase subunit beta
DNA-directed RNA polymerase subunit beta
DNA-directed RNA polymerase subunit beta
DNA-directed RNA polymerase subunit beta
DNA-directed RNA polymerase subunit beta
DNA-directed RNA polymerase subunit beta
DNA-directed RNA polymerase subunit beta
DNA-directed RNA polymerase subunit beta
DNA-directed RNA polymerase subunit beta
DNA-directed RNA polymerase subunit beta
DNA-directed RNA polymerase subunit beta
DNA-directed RNA polymerase subunit beta
DNA-directed RNA polymerase subunit beta
DNA-directed RNA polymerase subunit beta
DNA-directed RNA polymerase subunit beta
DNA-directed RNA polymerase subunit beta
DNA-directed RNA polymerase subunit beta
DNA-directed RNA polymerase subunit beta
DNA-directed RNA polymerase subunit beta
DNA-directed RNA polymerase subunit beta
DNA-directed RNA polymerase subunit beta
DNA-directed RNA polymerase subunit beta
DNA-directed RNA polymerase subunit beta
DNA-directed RNA polymerase subunit beta

DNA-directed RNA polymerase subunit beta
DNA-directed RNA polymerase subunit beta
DNA-directed RNA polymerase subunit beta
DNA-directed RNA polymerase subunit beta
DNA-directed RNA polymerase subunit beta
DNA-directed RNA polymerase subunit beta
DNA-directed RNA polymerase subunit beta
DNA-directed RNA polymerase subunit beta
DNA-directed RNA polymerase subunit beta
DNA-directed RNA polymerase subunit beta
DNA-directed RNA polymerase subunit beta
DNA-directed RNA polymerase subunit beta
50S ribosomal protein L7/L12
50S ribosomal protein L7/L12
50S ribosomal protein L7/L12
50S ribosomal protein L7/L12
50S ribosomal protein L7/L12
50S ribosomal protein L7/L12
50S ribosomal protein L7/L12
50S ribosomal protein L10
50S ribosomal protein L10
50S ribosomal protein L10
50S ribosomal protein L10
50S ribosomal protein L10
50S ribosomal protein L10
50S ribosomal protein L10
50S ribosomal protein L10
50S ribosomal protein L1
50S ribosomal protein L1
50S ribosomal protein L1
50S ribosomal protein L1
50S ribosomal protein L1
50S ribosomal protein L1
50S ribosomal protein L1
50S ribosomal protein L1
50S ribosomal protein L11
50S ribosomal protein L11
50S ribosomal protein L11
50S ribosomal protein L11
transcription antiterminator
transcription antiterminator
transcription antiterminator
transcription antiterminator
transcription antiterminator
transcription antiterminator
transcription antiterminator
DEAD/DEAH box helicase
DEAD/DEAH box helicase

DEAD/DEAH box helicase
leucyl aminopeptidase
leucyl aminopeptidase
peroxiredoxin
peroxiredoxin
peroxiredoxin
hypothetical protein LA_3453
hypothetical protein LA_3453
hypothetical protein LA_3453
glycerol uptake facilitator protein
polyphosphate kinase
polyphosphate kinase
polyphosphate kinase
polyphosphate kinase
polyphosphate kinase
polyphosphate kinase
polyphosphate kinase
polyphosphate kinase
polyphosphate kinase
polyphosphate kinase
polyphosphate kinase
polyphosphate kinase
polyphosphate kinase
polyphosphate kinase
polyphosphate kinase
polyphosphate kinase
outer membrane receptor for Fe3+-dicitrate/TonB-dependent receptor
outer membrane receptor for Fe3+-dicitrate/TonB-dependent receptor
outer membrane receptor for Fe3+-dicitrate/TonB-dependent receptor
outer membrane receptor for Fe3+-dicitrate/TonB-dependent receptor
outer membrane receptor for Fe3+-dicitrate/TonB-dependent receptor
outer membrane receptor for Fe3+-dicitrate/TonB-dependent receptor
thiol oxidoreductase
thiol oxidoreductase
hypothetical protein LA_3471
hypothetical protein LA_3471
esterase
esterase
esterase
esterase
hypothetical protein LA_3497
hypothetical protein LA_3497
hypothetical protein LA_3497
hypothetical protein LA_3497
hypothetical protein LA_3497
phosphoribosylformylglycinamidine (FGAM) synthase
phosphoribosylformylglycinamidine (FGAM) synthase
phosphoribosylaminoimidazole-succinocarboxamide synthase

phosphoribosylaminoimidazole-succinocarboxamide synthase
phosphoribosylaminoimidazole-succinocarboxamide synthase
phosphoribosylaminoimidazole-succinocarboxamide synthase
serine/threonine phosphatase
serine/threonine phosphatase
hypothetical protein LA_3522
hypothetical protein LA_3522
hypothetical protein LA_3522
hypothetical protein LA_3522
ABC transporter ATP-binding protein
hypothetical protein LA_3524
hypothetical protein LA_3524
hypothetical protein LA_3532
hypothetical protein LA_3532
hypothetical protein LA_3532
hypothetical protein LA_3532
hypothetical protein LA_3532
hypothetical protein LA_3533
hypothetical protein LA_3533
hypothetical protein LA_3535
hypothetical protein LA_3535
hypothetical protein LA_3535
hypothetical protein LA_3535
proton antiporter efflux pump
proton antiporter efflux pump
ribosomal protein serine acetyltransferase
ribosomal protein serine acetyltransferase
ABC transporter ATP-binding protein
ABC transporter ATP-binding protein
ABC transporter ATP-binding protein
hypothetical protein LA_3571
hypothetical protein LA_3571
hypothetical protein LA_3571
flagellar basal body-associated protein FliL
flagellar basal body-associated protein FliL
flagellar motor protein MotB
glycosyltransferase
glycosyltransferase
dioxygenase-like protein
dioxygenase-like protein
gamma-glutamyl carboxylase-like protein
ATP-dependent Lon protease
ATP-dependent Lon protease
ATP-dependent Lon protease
ATP-dependent Lon protease
ATP-dependent Lon protease
ATP-dependent Lon protease
ATP-dependent Lon protease

ATP-dependent Lon protease
ATP-dependent Lon protease
pyridoxal phosphate-dependent aminotransferase
pyridoxal phosphate-dependent aminotransferase
pyridoxal phosphate-dependent aminotransferase
pyridoxal phosphate-dependent aminotransferase
pyridoxal phosphate-dependent aminotransferase
pyridoxal phosphate-dependent aminotransferase
pyridoxal phosphate-dependent aminotransferase
hypothetical protein LA_3606
hypothetical protein LA_3606
hypothetical protein LA_3606
hypothetical protein LA_3606
MaoC-like acyl dehydratase
MaoC-like acyl dehydratase
MaoC-like acyl dehydratase
MaoC-like acyl dehydratase
MaoC-like acyl dehydratase
MaoC-like acyl dehydratase
2-dehydropantoate 2-reductase
2-dehydropantoate 2-reductase
glycosyltransferase
DNA polymerase I
DNA polymerase I
DNA polymerase I
DNA polymerase I
DNA polymerase I
DNA polymerase I
acyl-CoA dehydrogenase
acyl-CoA dehydrogenase
acyl-CoA dehydrogenase
acyl-CoA dehydrogenase
acyl-CoA dehydrogenase
acyl-CoA dehydrogenase
acyl-CoA dehydrogenase
acyl-CoA dehydrogenase
acyl-CoA dehydrogenase
anthranilate synthase component I
anthranilate synthase component I
anthranilate synthase component I
anthranilate synthase component I
anthranilate synthase component I
anthranilate synthase component I
receiver component of a two-component response regulator
FOG:HEAT repeat protein
FOG:HEAT repeat protein
cyclic amidohydrolase
cyclic amidohydrolase

homoserine dehydrogenase
homoserine dehydrogenase
homoserine dehydrogenase
homoserine dehydrogenase
homoserine dehydrogenase
homoserine dehydrogenase
homoserine dehydrogenase
anti-Sigma factor antagonist
enoyl-CoA hydratase
enoyl-CoA hydratase
enoyl-CoA hydratase
enoyl-CoA hydratase
enoyl-CoA hydratase
two-component response regulator
two-component response regulator
two-component response regulator
two-component response regulator
two-component response regulator
two-component response regulator
two-component response regulator
hydrolase
hydrolase/acyltransferase
hydrolase/acyltransferase
hydrolase/acyltransferase
hydrolase/acyltransferase
hydrolase/acyltransferase
acyl-CoA dehydrogenase
acyl-CoA dehydrogenase
acyl-CoA dehydrogenase
ABC transporter ATPase
ABC transporter ATPase
ABC transporter ATPase
ABC transporter ATPase
ABC transporter ATPase
ABC transporter ATPase
ABC transporter ATPase
ABC transporter ATPase
ABC transporter ATPase
ABC transporter ATPase
membrane carboxypeptidase/penicillin-binding protein
membrane carboxypeptidase/penicillin-binding protein
membrane carboxypeptidase/penicillin-binding protein
membrane carboxypeptidase/penicillin-binding protein
heat shock protein GrpE
heat shock protein GrpE
heat shock protein GrpE
heat shock protein GrpE
chaperone protein DnaK

chaperone protein DnaK
chaperone protein DnaK
chaperone protein DnaK
chaperone protein DnaK
chaperone protein DnaK
chaperone protein DnaK
chaperone protein DnaK
chaperone protein DnaK
chaperone protein DnaK
chaperone protein DnaK
chaperone protein DnaK
chaperone protein DnaK
chaperone protein DnaK
chaperone protein DnaK
chaperone protein DnaK
chaperone protein DnaK
chaperone protein DnaK
heat shock protein DnaJ
heat shock protein DnaJ
heat shock protein DnaJ
heat shock protein DnaJ
heat shock protein DnaJ
heat shock protein DnaJ
heat shock protein DnaJ
oxidoreductase
oxidoreductase
oxidoreductase
oxidoreductase
oxidoreductase
oxidoreductase
oxidoreductase
oxidoreductase
oxidoreductase
oxidoreductase
transcriptional regulator
transcriptional regulator
transcriptional regulator
transcriptional regulator
transcriptional regulator
lysophospholipase L1 biosynthesis ABC transporter permease
lysophospholipase L1 biosynthesis ABC transporter permease
lysophospholipase L1 biosynthesis ABC transporter permease
acriflavine resistance protein
hypothetical protein LA_3744
hypothetical protein LA_3744
hypothetical protein LA_3744
threonine synthase
threonine synthase

threonine synthase
threonine synthase
threonine synthase
threonine synthase
threonine synthase
threonine synthase
threonine synthase
threonine synthase
threonine synthase
threonine synthase
threonine synthase
threonine synthase
threonine synthase
hypothetical protein LA_3748
hypothetical protein LA_3748
hypothetical protein LA_3748
hypothetical protein LA_3748
hypothetical protein LA_3748
hypothetical protein LA_3748
hypothetical protein LA_3748
hypothetical protein LA_3748
hypothetical protein LA_3748
hypothetical protein LA_3748
hypothetical protein LA_3748
hypothetical protein LA_3748
hypothetical protein LA_3748
hypothetical protein LA_3749
hypothetical protein LA_3749
hypothetical protein LA_3749
hypothetical protein LA_3749
hypothetical protein LA_3751
hypothetical protein LA_3752
hypothetical protein LA_3752
hypothetical protein LA_3752
signal peptidase I
signal peptidase I
signal peptidase I
signal peptidase I
RNA-binding protein
RNA-binding protein
hypothetical protein LA_3762
hypothetical protein LA_3762
hypothetical protein LA_3762
hypothetical protein LA_3762
hypothetical protein LA_3762
hypothetical protein LA_3762
hypothetical protein LA_3765
hypothetical protein LA_3765

peptidyl-tRNA hydrolase
peptidyl-tRNA hydrolase
hypothetical protein LA_3769
hypothetical protein LA_3769
glyoxalase/bleomycin resistance protein/dioxygenase
glyoxalase/bleomycin resistance protein/dioxygenase
adenosine deaminase
adenosine deaminase
adenosine deaminase
adenosine deaminase
adenosine deaminase
adenosine deaminase
adenosine deaminase
adenosine deaminase
hydrolase
hydrolase
glycosyltransferase
glycosyltransferase
glycosyltransferase
hypothetical protein LA_3796
30S ribosomal protein S20
phosphomannomutase
phosphomannomutase
phosphomannomutase
glucosamine 6-phosphate synthetase
glucosamine 6-phosphate synthetase
glucosamine 6-phosphate synthetase
glucosamine 6-phosphate synthetase
glucosamine 6-phosphate synthetase
glucosamine 6-phosphate synthetase
glucosamine 6-phosphate synthetase
glucosamine 6-phosphate synthetase
glucosamine 6-phosphate synthetase
glucosamine 6-phosphate synthetase
glucosamine 6-phosphate synthetase
acetyl-CoA carboxylase carboxyltransferase component
acetyl-CoA carboxylase carboxyltransferase component
acetyl-CoA carboxylase carboxyltransferase component
acetyl-CoA carboxylase carboxyltransferase component
acetyl-CoA carboxylase carboxyltransferase component
acetyl-CoA carboxylase carboxyltransferase component
nitrogen regulatory protein P-II
nitrogen regulatory protein P-II
nitrogen regulatory protein P-II
hypothetical protein LA_3816
hypothetical protein LA_3816
hypothetical protein LA_3816
hypothetical protein LA_3818

hypothetical protein LA_3818
ATP-dependent Zn protease
ATP-dependent Zn protease
ATP-dependent Zn protease
ATP-dependent Zn protease
ATP-dependent Zn protease
ATP-dependent Zn protease
ATP-dependent Zn protease
ATP-dependent Zn protease
ATP-dependent Zn protease
ATP-dependent Zn protease
ATP-dependent Zn protease
ATP-dependent Zn protease
ATP-dependent Zn protease
ATP-dependent Zn protease
ATP-dependent Zn protease
peptidyl-tRNA hydrolase
peptidyl-tRNA hydrolase
peptidyl-tRNA hydrolase
peptidyl-tRNA hydrolase
peptidyl-tRNA hydrolase
50S ribosomal protein L25
50S ribosomal protein L25
50S ribosomal protein L25
50S ribosomal protein L25
50S ribosomal protein L25
50S ribosomal protein L25
phosphoribosyl pyrophosphate synthetase
phosphoribosyl pyrophosphate synthetase
phosphoribosyl pyrophosphate synthetase
phosphoribosyl pyrophosphate synthetase
phosphoribosyl pyrophosphate synthetase
phosphoribosyl pyrophosphate synthetase
UDP-N-acetylglucosamine diphosphorylase
UDP-N-acetylglucosamine diphosphorylase
UDP-N-acetylglucosamine diphosphorylase
UDP-N-acetylglucosamine diphosphorylase
UDP-N-acetylglucosamine diphosphorylase
UDP-N-acetylglucosamine diphosphorylase
UDP-N-acetylglucosamine diphosphorylase
hypothetical protein LA_3833
hypothetical protein LA_3833
hypothetical protein LA_3838
phospholipid binding protein
phospholipid binding protein
phospholipid binding protein
phospholipid binding protein
cation/multidrug efflux pump

cation/multidrug efflux pump
cation/multidrug efflux pump
cation/multidrug efflux pump
cation/multidrug efflux pump
cation/multidrug efflux pump
cation/multidrug efflux pump
cation/multidrug efflux pump
cation/multidrug efflux pump
cation/multidrug efflux pump
cation/multidrug efflux pump
cation/multidrug efflux pump
cation/multidrug efflux pump
cation/multidrug efflux pump
cation/multidrug efflux pump
cation/multidrug efflux pump
cation/multidrug efflux pump
cation/multidrug efflux pump
cation/multidrug efflux pump
cation/multidrug efflux pump
hypothetical protein LA_3849
hypothetical protein LA_3861
hypothetical protein LA_3861
response regulator containing a signal receiver domain and a DNA-binding domain
response regulator containing a signal receiver domain and a DNA-binding domain
response regulator containing a signal receiver domain and a DNA-binding domain
response regulator containing a signal receiver domain and a DNA-binding domain
response regulator containing a signal receiver domain and a DNA-binding domain
response regulator containing a signal receiver domain and a DNA-binding domain
hypothetical protein LA_3870
hypothetical protein LA_3870
hypothetical protein LA_3870
hypothetical protein LA_3870
hypothetical protein LA_3870
hypothetical protein LA_3870
hypothetical protein LA_3870
hypothetical protein LA_3870
hypothetical protein LA_3870
periplasmic serine protease
periplasmic serine protease
periplasmic serine protease
periplasmic serine protease
acid phosphatase
acid phosphatase
acid phosphatase
glucose-6-phosphate isomerase
glucose-6-phosphate isomerase
glucose-6-phosphate isomerase
glucose-6-phosphate isomerase

glucose-6-phosphate isomerase
glucose-6-phosphate isomerase
nucleoside-diphosphate-sugar pyrophosphorylase
nucleoside-diphosphate-sugar pyrophosphorylase
Ser/Thr protein kinase-like phosphotransferase
Ser/Thr protein kinase-like phosphotransferase
histidine kinase/response regulator hybrid protein
histidine kinase/response regulator hybrid protein
histidine kinase/response regulator hybrid protein
histidine kinase/response regulator hybrid protein
acyltransferase
metallo-beta-lactamase superfamily hydrolase
metallo-beta-lactamase superfamily hydrolase
metallo-beta-lactamase superfamily hydrolase
metallo-beta-lactamase superfamily hydrolase
hypothetical protein LA_3910
hypothetical protein LA_3910
hypothetical protein LA_3912
hypothetical protein LA_3912
hypothetical protein LA_3912
hypothetical protein LA_3912
hypothetical protein LA_3912
hypothetical protein LA_3912
hypothetical protein LA_3912
hypothetical protein LA_3913
hypothetical protein LA_3913
hypothetical protein LA_3917
hypothetical protein LA_3917
methionyl-tRNA synthetase
methionyl-tRNA synthetase
methionyl-tRNA synthetase
methionyl-tRNA synthetase
methionyl-tRNA synthetase
methionyl-tRNA synthetase
methionyl-tRNA synthetase
methionyl-tRNA synthetase
methionyl-tRNA synthetase
hypothetical protein LA_3922
hypothetical protein LA_3922
hypothetical protein LA_3922
NAD(P)H steroid dehydrogenase
NAD(P)H steroid dehydrogenase
NAD(P)H steroid dehydrogenase
NAD(P)H steroid dehydrogenase
response regulator with GGDEF domain
response regulator with GGDEF domain
hypothetical protein LA_3934
flavoprotein

flavoprotein
flavoprotein
hemolysin-like protein
hemolysin-like protein
hypothetical protein LA_3938
hypothetical protein LA_3938
hypothetical protein LA_3938
hypothetical protein LA_3938
hypothetical protein LA_3938
hypothetical protein LA_3938
hypothetical protein LA_3938
glycerol-3-phosphate dehydrogenase
glycerol-3-phosphate dehydrogenase
glycerol-3-phosphate dehydrogenase
glycerol-3-phosphate dehydrogenase
glycerol-3-phosphate dehydrogenase
glycerol-3-phosphate dehydrogenase
ATP-dependent DNA helicase RecG
D-alanyl-D-alanine carboxypeptidase
D-alanyl-D-alanine carboxypeptidase
D-alanyl-D-alanine carboxypeptidase
D-alanyl-D-alanine carboxypeptidase
UDP-glucose 4-epimerase
UDP-glucose 4-epimerase
UDP-glucose 4-epimerase
UDP-glucose 4-epimerase
UDP-N-acetylglucosamine acyltransferase
UDP-N-acetylglucosamine acyltransferase
UDP-N-acetylglucosamine acyltransferase
UDP-N-acetylglucosamine acyltransferase
hypothetical protein LA_3950
hypothetical protein LA_3950
3-deoxy-D-arabino-heptulosonate 7-phosphate synthase
3-deoxy-D-arabino-heptulosonate 7-phosphate synthase
3-deoxy-D-arabino-heptulosonate 7-phosphate synthase
3-deoxy-D-arabino-heptulosonate 7-phosphate synthase
3-deoxy-D-arabino-heptulosonate 7-phosphate synthase
3-deoxy-D-arabino-heptulosonate 7-phosphate synthase
3-deoxy-D-arabino-heptulosonate 7-phosphate synthase
3-deoxy-D-arabino-heptulosonate 7-phosphate synthase
3-deoxy-D-arabino-heptulosonate 7-phosphate synthase
3-methyl-2-oxobutanoate hydroxymethyltransferase
3-methyl-2-oxobutanoate hydroxymethyltransferase
3-methyl-2-oxobutanoate hydroxymethyltransferase
3-methyl-2-oxobutanoate hydroxymethyltransferase
2-amino-4-hydroxy-6-hydroxymethyldihydropteridine pyrophosphokinase
DNA replication protein DnaC
GDP-mannose 4,6-dehydratase

GDP-mannose 4,6-dehydratase
GDP-mannose 4,6-dehydratase
GDP-mannose 4,6-dehydratase
GDP-mannose 4,6-dehydratase
GDP-mannose 4,6-dehydratase
GDP-mannose 4,6-dehydratase
GDP-mannose 4,6-dehydratase
GDP-mannose 4,6-dehydratase
GDP-mannose 4,6-dehydratase
GDP-mannose 4,6-dehydratase
GDP-mannose 4,6-dehydratase
adenosine/AMP deaminase
adenosine/AMP deaminase
adenosine/AMP deaminase
potassium uptake system, NAD-binding component
potassium uptake system, NAD-binding component
potassium uptake system, NAD-binding component
NTP pyrophosphohydrolase
NTP pyrophosphohydrolase
NTP pyrophosphohydrolase
lysine methyltransferase
hypothetical protein LA_3982
hypothetical protein LA_3982
hypothetical protein LA_3982
hypothetical protein LA_3982
hypothetical protein LA_3982
hypothetical protein LA_3982
hypothetical protein LA_3982
hypothetical protein LA_3982
hypothetical protein LA_3982
hypothetical protein LA_3982
hypothetical protein LA_3982
GTPase
cholesterol oxidase
cholesterol oxidase
cholesterol oxidase
cholesterol oxidase
cholesterol oxidase
cholesterol oxidase
cholesterol oxidase
choline dehydrogenase
choline dehydrogenase
adenylate/guanylate cyclase
adenylate/guanylate cyclase
adenylate/guanylate cyclase
adenylate/guanylate cyclase
adenylate/guanylate cyclase
hypothetical protein LA_4027

phenylalanyl-tRNA synthetase subunit beta
phenylalanyl-tRNA synthetase subunit beta
phenylalanyl-tRNA synthetase subunit beta
phenylalanyl-tRNA synthetase subunit beta
phenylalanyl-tRNA synthetase subunit beta
phenylalanyl-tRNA synthetase subunit beta
phenylalanyl-tRNA synthetase subunit beta
phenylalanyl-tRNA synthetase subunit beta
phenylalanyl-tRNA synthetase subunit beta
phenylalanyl-tRNA synthetase subunit beta
phenylalanyl-tRNA synthetase subunit beta
phenylalanyl-tRNA synthetase subunit beta
phenylalanyl-tRNA synthetase subunit beta
phenylalanyl-tRNA synthetase subunit beta
phenylalanyl-tRNA synthetase subunit beta
phenylalanyl-tRNA synthetase subunit beta
phenylalanyl-tRNA synthetase subunit beta
inorganic pyrophosphatase
inorganic pyrophosphatase
inorganic pyrophosphatase
inorganic pyrophosphatase
hypothetical protein LA_4046
short-chain dehydrogenase
short-chain dehydrogenase
lysine decarboxylase-like protein
lysine decarboxylase-like protein
two-component response regulator
isocitrate dehydrogenase
isocitrate dehydrogenase
isocitrate dehydrogenase
isocitrate dehydrogenase
isocitrate dehydrogenase
isocitrate dehydrogenase
isocitrate dehydrogenase
isocitrate dehydrogenase
isocitrate dehydrogenase
isocitrate dehydrogenase
isocitrate dehydrogenase
isocitrate dehydrogenase
isocitrate dehydrogenase
isocitrate dehydrogenase
isocitrate dehydrogenase
isocitrate dehydrogenase
isocitrate dehydrogenase
isocitrate dehydrogenase
isocitrate dehydrogenase
isocitrate dehydrogenase
hypothetical protein LA_4068

UDP-N-acetylglucosamine 1-carboxyvinyltransferase
UDP-N-acetylglucosamine 1-carboxyvinyltransferase
UDP-N-acetylglucosamine 1-carboxyvinyltransferase
UDP-N-acetylglucosamine 1-carboxyvinyltransferase
UDP-N-acetylglucosamine 1-carboxyvinyltransferase
UDP-N-acetylglucosamine 1-carboxyvinyltransferase
Zn-dependent alcohol dehydrogenase
Zn-dependent alcohol dehydrogenase
Zn-dependent alcohol dehydrogenase
Zn-dependent alcohol dehydrogenase
Zn-dependent alcohol dehydrogenase
Zn-dependent alcohol dehydrogenase
ferredoxin
ferredoxin
hypothetical protein LA_4087
MutT/nudix family protein
MutT/nudix family protein
hypothetical protein LA_4097
hypothetical protein LA_4100
hypothetical protein LA_4100
response regulator
response regulator
serine phosphatase RsbU
hypothetical protein LA_4109
hypothetical protein LA_4109
hypothetical protein LA_4109
hypothetical protein LA_4109
hypothetical protein LA_4109
hypothetical protein LA_4109
hypothetical protein LA_4109
acetyl-CoA acetyltransferase
acetyl-CoA acetyltransferase
MaoC-like acyl dehydratase
acyl-CoA dehydrogenase
NAD dependent DNA ligase
NAD dependent DNA ligase
NAD dependent DNA ligase
NAD dependent DNA ligase
NAD dependent DNA ligase
NAD dependent DNA ligase
NAD dependent DNA ligase
NAD dependent DNA ligase
NAD dependent DNA ligase
NAD dependent DNA ligase
peptidase-like protein
peptidase-like protein
peptidase-like protein
peptidase-like protein

peptidase-like protein
peptidase-like protein
NADPH-dependent FMN reductase
3-hydroxyacyl-CoA dehydrogenase
3-hydroxyacyl-CoA dehydrogenase
3-hydroxyacyl-CoA dehydrogenase
3-hydroxyacyl-CoA dehydrogenase
3-hydroxyacyl-CoA dehydrogenase
3-hydroxyacyl-CoA dehydrogenase
3-hydroxyacyl-CoA dehydrogenase
3-hydroxyacyl-CoA dehydrogenase
3-hydroxyacyl-CoA dehydrogenase
3-hydroxyacyl-CoA dehydrogenase
3-hydroxyacyl-CoA dehydrogenase
3-hydroxyacyl-CoA dehydrogenase
3-hydroxyacyl-CoA dehydrogenase
3-hydroxyacyl-CoA dehydrogenase
3-hydroxyacyl-CoA dehydrogenase
3-hydroxyacyl-CoA dehydrogenase
acetyl-CoA acetyltransferase
acetyl-CoA acetyltransferase
acetyl-CoA acetyltransferase
acetyl-CoA acetyltransferase
acetyl-CoA acetyltransferase
acetyl-CoA acetyltransferase
acetyl-CoA acetyltransferase
acetyl-CoA acetyltransferase
acetyl-CoA acetyltransferase
acetyl-CoA acetyltransferase
phenylalanine-tRNA synthetase subunit alpha
phenylalanine-tRNA synthetase subunit alpha
phenylalanine-tRNA synthetase subunit alpha
UDP-N-acetylmuramate--L-alanine ligase
UDP-N-acetylmuramate--L-alanine ligase
UDP-N-acetylmuramate--L-alanine ligase
hypothetical protein LA_4153
hypothetical protein LA_4153
ABC transporter ATP-binding protein
ABC transporter ATP-binding protein
ABC transporter ATP-binding protein
ABC transporter ATP-binding protein
ABC transporter ATP-binding protein
ABC transporter ATP-binding protein
ABC transporter ATP-binding protein
fatty acid synthase subunit beta
fatty acid synthase subunit beta
fatty acid synthase subunit beta
fatty acid synthase subunit beta

fatty acid synthase subunit beta
fatty acid synthase subunit beta
fatty acid synthase subunit beta
fatty acid synthase subunit beta
fatty acid synthase subunit beta
fatty acid synthase subunit beta
fatty acid synthase subunit beta
fatty acid synthase subunit beta
fatty acid synthase subunit beta
hypothetical protein LA_4160
argininosuccinate synthase
argininosuccinate synthase
argininosuccinate synthase
argininosuccinate synthase
argininosuccinate synthase
argininosuccinate synthase
argininosuccinate synthase
argininosuccinate synthase
argininosuccinate synthase
argininosuccinate synthase
argininosuccinate synthase
argininosuccinate synthase
argininosuccinate synthase
argininosuccinate synthase
argininosuccinate synthase
argininosuccinate synthase
pantetheine-phosphate adenylyltransferase
pantetheine-phosphate adenylyltransferase
pantetheine-phosphate adenylyltransferase
nucleoside-diphosphate kinase
nucleoside-diphosphate kinase
nucleoside-diphosphate kinase
nucleoside-diphosphate kinase
nucleoside-diphosphate kinase
nucleoside-diphosphate kinase
nucleoside-diphosphate kinase
isocitrate/isopropylmalate dehydrogenase
isocitrate/isopropylmalate dehydrogenase
isocitrate/isopropylmalate dehydrogenase
isocitrate/isopropylmalate dehydrogenase
isocitrate/isopropylmalate dehydrogenase
isocitrate/isopropylmalate dehydrogenase
isocitrate/isopropylmalate dehydrogenase
isocitrate/isopropylmalate dehydrogenase
isocitrate/isopropylmalate dehydrogenase
isocitrate/isopropylmalate dehydrogenase
GMC oxidoreductase
GMC oxidoreductase

hypothetical protein LA_4182
bifunctional glutamate-tRNA ligase/glutamine-tRNA ligase
bifunctional glutamate-tRNA ligase/glutamine-tRNA ligase
bifunctional glutamate-tRNA ligase/glutamine-tRNA ligase
bifunctional glutamate-tRNA ligase/glutamine-tRNA ligase
bifunctional glutamate-tRNA ligase/glutamine-tRNA ligase
bifunctional glutamate-tRNA ligase/glutamine-tRNA ligase
bifunctional glutamate-tRNA ligase/glutamine-tRNA ligase
bifunctional glutamate-tRNA ligase/glutamine-tRNA ligase
bifunctional glutamate-tRNA ligase/glutamine-tRNA ligase
bifunctional glutamate-tRNA ligase/glutamine-tRNA ligase
bifunctional glutamate-tRNA ligase/glutamine-tRNA ligase
bifunctional glutamate-tRNA ligase/glutamine-tRNA ligase
bifunctional glutamate-tRNA ligase/glutamine-tRNA ligase
bifunctional glutamate-tRNA ligase/glutamine-tRNA ligase
DNA gyrase subunit A
DNA gyrase subunit A
DNA gyrase subunit A
DNA gyrase subunit A
DNA gyrase subunit A
DNA gyrase subunit A
DNA gyrase subunit A
DNA gyrase subunit A
DNA gyrase subunit A
DNA gyrase subunit B
DNA gyrase subunit B
DNA gyrase subunit B
DNA gyrase subunit B
DNA gyrase subunit B
DNA gyrase subunit B
DNA gyrase subunit B
DNA gyrase subunit B
DNA gyrase subunit B
DNA gyrase subunit B
DNA gyrase subunit B
DNA gyrase subunit B
DNA gyrase subunit B
DNA gyrase subunit B
DNA gyrase subunit B
L-amino acid oxidase
L-amino acid oxidase
L-amino acid oxidase
L-amino acid oxidase
L-amino acid oxidase
hypothetical protein LA_4208
hypothetical protein LA_4208
siroheme synthase
sulfate adenylyltransferase subunit 1

sulfate adenylyltransferase subunit 1
sulfate adenylyltransferase subunit 1
sulfate adenylyltransferase subunit 1
sulfate adenylyltransferase subunit 1
sulfate adenylyltransferase subunit 2
sulfate adenylyltransferase subunit 2
sulfate adenylyltransferase subunit 2
sulfate adenylyltransferase subunit 2
sulfate adenylyltransferase subunit 2
sulfate adenylyltransferase subunit 2
sulfate adenylyltransferase subunit 2
phosphoadenosine phosphosulfate reductase
DNA mismatch repair protein ATPase component
polysaccharide deacetylase
polysaccharide deacetylase
polysaccharide deacetylase
polysaccharide deacetylase
polysaccharide deacetylase
polysaccharide deacetylase
methyl-accepting chemotaxis protein
methyl-accepting chemotaxis protein
purine nucleoside phosphorylase
purine nucleoside phosphorylase
purine nucleoside phosphorylase
purine nucleoside phosphorylase
purine nucleoside phosphorylase
purine nucleoside phosphorylase
purine nucleoside phosphorylase
purine nucleoside phosphorylase
methyl-accepting chemotaxis protein
methyl-accepting chemotaxis protein
acetyl-CoA synthetase
acetyl-CoA synthetase
acetyl-CoA synthetase
acetyl-CoA synthetase
acetyl-CoA synthetase
acetyl-CoA synthetase
acetyl-CoA synthetase
GTP cyclohydrolase I
GTP cyclohydrolase I
GTP cyclohydrolase I
mannosyltransferase
mannosyltransferase
hypothetical protein LA_4258
phosphoribosylglycinamide formyltransferase 2
phosphoribosylglycinamide formyltransferase 2
phosphoribosylglycinamide formyltransferase 2
phosphoribosylglycinamide formyltransferase 2

phosphoribosylglycinamide formyltransferase 2
hypothetical protein LA_4272
hypothetical protein LA_4272
hypothetical protein LA_4272
hypothetical protein LA_4272
hypothetical protein LA_4272
hypothetical protein LA_4272
hypothetical protein LA_4272
formate hydrogenase subunit C
metal (Ni/Fe) hydrogenase large subunit
metal (Ni/Fe) hydrogenase large subunit
metal (Ni/Fe) hydrogenase large subunit
metal (Ni/Fe) hydrogenase large subunit
metal (Ni/Fe) hydrogenase large subunit
metal (Ni/Fe) hydrogenase large subunit
metal (Ni/Fe) hydrogenase large subunit
metal (Ni/Fe) hydrogenase small subunit
metal (Ni/Fe) hydrogenase small subunit
metal (Ni/Fe) hydrogenase small subunit
ABC transporter ATP-binding protein
hypothetical protein LA_4291
hypothetical protein LA_4293
hypothetical protein LA_4293
hypothetical protein LA_4293
hypoxanthine phosphoribosyltransferase
hypoxanthine phosphoribosyltransferase
sodium/hydrogen antiporter
glutathione peroxidase
glutathione peroxidase
Hsp31/PfpI family protein
Hsp31/PfpI family protein
Hsp31/PfpI family protein
flagellar assembly protein FliW
flagellar hook-associated protein FlgK
hypothetical protein LA_4315
hypothetical protein LA_4315
hypothetical protein LA_4315
hypothetical protein LA_4332
hypothetical protein LA_4335
hypothetical protein LA_4335
peptide ABC transporter periplasmic
seryl-tRNA synthetase
seryl-tRNA synthetase
seryl-tRNA synthetase
seryl-tRNA synthetase
seryl-tRNA synthetase
Mg-dependent DNase
metalloendopeptidase

metalloendopeptidase
metalloendopeptidase
ParB-like protein
ParB-like protein
ParB-like protein
ParB-like protein
ParB-like protein
ParB-like protein
glucose-inhibited division protein B
glucose-inhibited division protein B
glucose-inhibited division protein B
metallopeptidase
metallopeptidase
metallopeptidase
metallopeptidase
metallopeptidase
hypothetical protein LA_4355
hypothetical protein LA_4355
hypothetical protein LA_4355
hypothetical protein LA_4355
tRNA uridine 5-carboxymethylaminomethyl modification enzyme GidA
tRNA uridine 5-carboxymethylaminomethyl modification enzyme GidA
DNA polymerase III gamma and tau subunits
DNA polymerase III gamma and tau subunits
DNA polymerase III gamma and tau subunits
5,10 methylene tetrahydrofolate reductase
5,10 methylene tetrahydrofolate reductase
hypothetical protein LIC20005
hypothetical protein LIC20005
hypothetical protein LIC20005
porphobilinogen deaminase
porphobilinogen deaminase
delta-aminolevulinic acid dehydratase
delta-aminolevulinic acid dehydratase
delta-aminolevulinic acid dehydratase
delta-aminolevulinic acid dehydratase
delta-aminolevulinic acid dehydratase
glutamate-1-semialdehyde aminotransferase
glutamate-1-semialdehyde aminotransferase
glutamate-1-semialdehyde aminotransferase
glutamate-1-semialdehyde aminotransferase
glutamate-1-semialdehyde aminotransferase
glutamate-1-semialdehyde aminotransferase
glutamate-1-semialdehyde aminotransferase
response regulator
uroporphyrinogen decarboxylase
coproporphyrinogen III oxidase
coproporphyrinogen III oxidase

coproporphyrinogen III oxidase
azoreductase
azoreductase
azoreductase
short-chain dehydrogenase
short-chain dehydrogenase
NADH dehydrogenase
NADH dehydrogenase
NADH dehydrogenase
NADH dehydrogenase
NADH dehydrogenase
hypothetical protein LIC20030
hypothetical protein LIC20032
hypothetical protein LIC20034
hypothetical protein LIC20034
hypothetical protein LIC20035
hypothetical protein LIC20035
hypothetical protein LIC20035
hypothetical protein LIC20035
hypothetical protein LIC20035
hypothetical protein LIC20035
hypothetical protein LIC20035
hypothetical protein LIC20036
methanol dehydrogenase regulator
methanol dehydrogenase regulator
methanol dehydrogenase regulator
methanol dehydrogenase regulator
methanol dehydrogenase regulator
methanol dehydrogenase regulator
heat shock protein 90
heat shock protein 90
heat shock protein 90
heat shock protein 90
heat shock protein 90
heat shock protein 90
hypothetical protein LIC20045
anti-sigma factor antagonist
anti-sigma factor antagonist
hypothetical protein LIC20049
fatty acid desaturase
LAO/AO transport system kinase
methylmalonyl-CoA mutase
methylmalonyl-CoA mutase
methylmalonyl-CoA mutase
methylmalonyl-CoA mutase
methylmalonyl-CoA mutase
methylmalonyl-CoA mutase
methylmalonyl-CoA mutase

methylmalonyl-CoA mutase
thiol-disulfide interchange like protein
phosphoribosyl-ATP pyrophosphatase
phosphoribosyl-ATP pyrophosphatase
phosphoribosyl-ATP pyrophosphatase
3-oxoacyl-(acyl-carrier protein) reductase
3-oxoacyl-(acyl-carrier protein) reductase
3-oxoacyl-(acyl-carrier protein) reductase
acyl carrier protein
acyl carrier protein
acyl carrier protein
hypothetical protein LIC20078
hypothetical protein LIC20078
hypothetical protein LIC20078
haloacid dehalogenase-like protein
4-hydroxybenzoyl-CoA thioesterase
4-hydroxybenzoyl-CoA thioesterase
S-adenosyl-L-homocysteine hydrolase
S-adenosyl-L-homocysteine hydrolase
S-adenosyl-L-homocysteine hydrolase
S-adenosyl-L-homocysteine hydrolase
S-adenosyl-L-homocysteine hydrolase
S-adenosyl-L-homocysteine hydrolase
S-adenosyl-L-homocysteine hydrolase
S-adenosyl-L-homocysteine hydrolase
S-adenosyl-L-homocysteine hydrolase
S-adenosyl-L-homocysteine hydrolase
B12-dependent methionine synthase
B12-dependent methionine synthase
B12-dependent methionine synthase
B12-dependent methionine synthase
B12-dependent methionine synthase
B12-dependent methionine synthase
B12-dependent methionine synthase
B12-dependent methionine synthase
B12-dependent methionine synthase
B12-dependent methionine synthase
B12-dependent methionine synthase
B12-dependent methionine synthase
B12-dependent methionine synthase
B12-dependent methionine synthase
B12-dependent methionine synthase
B12-dependent methionine synthase
B12-dependent methionine synthase
diphosphate--fructose-6-phosphate 1-phosphotransferase
diphosphate--fructose-6-phosphate 1-phosphotransferase
sigma factor regulatory protein
bacterioferritin comigratory protein

bacterioferritin comigratory protein
adenylate/guanylate cyclase
hypothetical protein LIC20096
hypothetical protein LIC20096
hypothetical protein LIC20096
short-chain dehydrogenase
short-chain dehydrogenase
shikimate kinase
hypothetical protein LIC20100
pheromone shutdown protein
pheromone shutdown protein
pheromone shutdown protein
anti-sigma factor antagonist
anti-sigma factor antagonist
anti-sigma factor antagonist
anti-sigma factor antagonist
LipL45 homologue
RNA polymerase ECF-type sigma factor
hypothetical protein LIC20136
capsular polysaccharide biosynthesis protein
capsular polysaccharide biosynthesis protein
hypothetical protein LIC20139
putative permease
heat shock protein HtpX
heat shock protein HtpX
adenine phosphoribosyltransferase
adenine phosphoribosyltransferase
adenine phosphoribosyltransferase
HtrA1
HtrA1
HtrA1-like protein
HtrA1-like protein
hypothetical protein LIC20146
hypothetical protein LIC20153
ATP-dependent protease
hypothetical protein LIC20160
mannosyltransferase
hypothetical protein LIC20162
hypothetical protein LIC20162
hypothetical protein LIC20165
hypothetical protein LIC20165
hypothetical protein LIC20167
hypothetical protein LIC20167
MotA/TolQ/ExbB proton channel family protein
lipoprotein
dnaK suppressor
dnaK suppressor
dnaK suppressor

dnaK suppressor
hypothetical protein LIC20185
hypothetical protein LIC20185
hypothetical protein LIC20185
hypothetical protein LIC20185
hypothetical protein LIC20185
hypothetical protein LIC20185
hypothetical protein LIC20185
hypothetical protein LIC20185
hypothetical protein LIC20186
hypothetical protein LIC20190
hypothetical protein LIC20190
hypothetical protein LIC20190
4-hydroxybutyrate CoA transferase
4-hydroxybutyrate CoA transferase
4-hydroxybutyrate CoA transferase
hypothetical protein LIC20196
hypothetical protein LIC20196
cysteine protease
cysteine protease
cysteine protease
cysteine protease
cysteine protease
cysteine protease
cysteine protease
cysteine protease
cysteine protease
cysteine protease
UTP-glucose-1-phosphate uridyltransferase
UTP-glucose-1-phosphate uridyltransferase
UTP-glucose-1-phosphate uridyltransferase
cysteine desulfurase
cysteine desulfurase
cysteine desulfurase
hypothetical protein LIC20205
hypothetical protein LIC20205
hypothetical protein LIC20205
hypothetical protein LIC20205
hypothetical protein LIC20205
methylmalonyl-COA mutase small subunit
methylmalonyl-COA mutase small subunit
methylmalonyl-COA mutase small subunit
methylmalonyl-COA mutase small subunit
methylmalonyl-COA mutase small subunit
methylmalonyl-COA mutase small subunit
methylmalonyl-COA mutase small subunit
methylmalonyl-CoA mutase
methylmalonyl-CoA mutase

methylmalonyl-CoA mutase
methylmalonyl-CoA mutase
methylmalonyl-CoA mutase
methylmalonyl-CoA mutase
methylmalonyl-CoA mutase
methylmalonyl-CoA mutase
hypothetical protein LIC20214
hypothetical protein LIC20214
ExbB
ExbB
ExbB
hypothetical protein LIC20223
3-oxoacyl-[acyl-carrier protein] reductase
3-oxoacyl-[acyl-carrier protein] reductase
3-oxoacyl-[acyl-carrier protein] reductase
acireductone dioxygenase enzyme ARD and ARD'
acireductone dioxygenase enzyme ARD and ARD'
acireductone dioxygenase enzyme ARD and ARD'
acireductone dioxygenase enzyme ARD and ARD'
acireductone dioxygenase enzyme ARD and ARD'
phosphate ABC transporter periplasmic phosphate-binding protein
S-adenosylmethionine decarboxylase like protein
S-adenosylmethionine decarboxylase like protein
S-adenosylmethionine decarboxylase like protein
S-adenosylmethionine decarboxylase proenzyme
hypothetical protein LIC20240
alanine racemase
alanine racemase
alanine racemase
alanine racemase
alanine racemase
alanine racemase
alanine racemase
alanine racemase
putative acyltransferase
putative acyltransferase
putative acyltransferase
putative acyltransferase
hypothetical protein LIC20243
hypothetical protein LIC20243
hypothetical protein LIC20243
hypothetical protein LIC20243
hypothetical protein LIC20243
hypothetical protein LIC20244
histidine kinase sensor protein
histidine kinase sensor protein
histidine kinase sensor protein
histidine kinase sensor protein

aconitate hydratase
aconitate hydratase
aconitate hydratase
aconitate hydratase
aconitate hydratase
aconitate hydratase
aconitate hydratase
aconitate hydratase
aconitate hydratase
aconitate hydratase
aconitate hydratase
aconitate hydratase
aconitate hydratase
aconitate hydratase
aconitate hydratase
aconitate hydratase
peptidoglycan-associated cytoplasmic membrane protein
response regulator
response regulator
response regulator
response regulator
response regulator
response regulator
pterin-4-alpha-carbinolamine dehydratase
hypothetical protein LIC20257
hypothetical protein LIC20262
pyruvate kinase
pyruvate kinase
pyruvate kinase
pyruvate kinase
pyruvate kinase
hypothetical protein LIC20265
aspartate-semialdehyde dehydrogenase
aspartate-semialdehyde dehydrogenase
aspartate-semialdehyde dehydrogenase
aspartate-semialdehyde dehydrogenase
aspartate-semialdehyde dehydrogenase
aspartate-semialdehyde dehydrogenase
hypothetical protein LIC20272
hypothetical protein LIC20272
hypothetical protein LIC20272
hypothetical protein LIC20272
hypothetical protein LIC20272
putative transcriptional regulator
hypothetical protein LIC20274
hypothetical protein LIC20274
hypothetical protein LIC20274
hypothetical protein LIC20275

hypothetical protein LIC20275
hypothetical protein LIC20275
long-chain-fatty-acid CoA ligase
long-chain-fatty-acid CoA ligase
hypothetical protein LIC10115
phoshomethylpyrimidine kinase protein
phoshomethylpyrimidine kinase protein
HD-GYP hydrolase domain-containing protein
phosphoenolpyruvate carboxykinase
hypothetical protein LIC10291
hypothetical protein LIC10314
hypothetical protein LIC10314
hypothetical protein LIC10337
hemolysin B
hypothetical protein LIC10348
phosphoribosyl pyrophosphate synthetase
phosphoribosyl pyrophosphate synthetase
hypothetical protein LIC10521
lipase
enoyl-CoA hydratase
anthranilate synthase component II
acyl-CoA dehydrogenase
DNA polymerase I
ammonium transporter
DNA-binding stress protein
hypothetical protein LIC10618
anti-sigma factor antagonist
hypothetical protein LIC10640
hypothetical protein LIC10643
hypothetical protein LIC10692
hypothetical protein LIC10712
polyphosphate kinase
polyphosphate kinase
glycerol uptake facilitator protein
30S ribosomal protein S7
hypothetical protein LIC10770
enoyl-CoA hydratase
hypothetical protein LIC10824
hypothetical protein LIC10828
hypothetical protein LIC10828
transketolase alpha subunit protein
monooxygenase
outer membrane protein
FUR family transcriptional regulator
hypothetical protein LIC11017
hypothetical protein LIC11036
periplasmic trypsin-like serine protease
hypothetical protein LIC11051

NH(3)-dependent NAD(+) synthetase
uridine 5-monophosphate synthase
HtrA2
hypothetical protein LIC11137
hypothetical protein LIC11177
hypothetical protein LIC11183
methyl-accepting chemotaxis transducer transmembrane protein
peroxiredoxin
F0F1 ATP synthase subunit gamma
F0F1 ATP synthase subunit epsilon
F0F1 ATP synthase subunit epsilon
periplasmic sulfate-binding protein
phosphoglycolate phosphatase
aminopeptidase P
N-acyl-L-amino acid amidohydrolase
chaperonin GroEL
hypothetical protein LIC11340
transketolase
hypothetical protein LIC11359
hypothetical protein LIC11359
hypothetical protein LIC11369
hypothetical protein LIC11369
hypothetical protein LIC11381
hypothetical protein LIC11390
hypothetical protein LIC11408
glycine rich RNA-binding protein
histidine kinase sensor protein
adenylate/guanylate cyclase
hypothetical protein LIC11468
thioredoxin reductase
acetyl-CoA carboxylase alpha subunit
biotin carboxylase subunit of acetyl CoA carboxylase
biotin carboxylase subunit of acetyl CoA carboxylase
chemotaxis response regulator
ADP-heptose synthase
HPr kinase/phosphorylase
LipL45
lipoyl synthase
hypothetical protein LIC11650
hypothetical protein LIC11653
endonuclease
2,4-dienoyl-coa reductase
N-acetyl-gamma-glutamyl-phosphate reductase
N-acetyl-gamma-glutamyl-phosphate reductase
3-isopropylmalate dehydrogenase
response regulator
aspartate aminotransferase
dihydrolipoamide dehydrogenase

BolA-like protein
BolA-like protein
isopropylmalate isomerase large subunit
hypothetical protein LIC11823
putative methyltransferase
putative lipoprotein
hypothetical protein LIC11909
hypothetical protein LIC11955
hypothetical protein LIC11970
cyclic nucleotide binding protein
succinate dehydrogenase flavoprotein subunit
catalase
cysteine synthase
phosphoglycerate kinase
phosphoglycerate kinase
replicative DNA helicase
dTDP-glucose 4,6-dehydratase
UDP-glucose lipid carrier transferase
glucose-1-phosphate cytidylyltransferase
hypothetical protein LIC12163
alcohol dehydrogenase
alcohol dehydrogenase
alcohol dehydrogenase
transketolase beta subunit
transketolase beta subunit
transketolase
transketolase
transketolase
transketolase
transketolase
transketolase
transketolase
transketolase
hypothetical protein LIC12196
hypothetical protein LIC12196
hypothetical protein LIC12196
hypothetical protein LIC12196
hypothetical protein LIC12196
aminotransferase
aminotransferase
aminotransferase
aminotransferase
aminotransferase
aminotransferase
aminotransferase
aminotransferase
aminotransferase
aminotransferase

aminotransferase
aminotransferase
aminotransferase
aminotransferase
aminotransferase
aminotransferase
UDP-glucose 4-epimerase
heavy metal efflux pump
mannose-1-phosphate guanylyltransferase
hypothetical protein LIC12263
phosphoribosylaminoimidazole carboxylase catalytic subunit protein
beta-ketoacyl synthase
hypothetical protein LIC12332
hypothetical protein LIC12341
capsule biosynthesis protein
capsule biosynthesis protein
isoleucyl-tRNA synthetase
phosphoribosylformylglycinamide synthetase
putative glutamine synthetase protein
hypothetical protein LIC12408
hypothetical protein LIC12413
phosphoglycerol transferase
translation initiation factor IF-3
dihydrolipoamide succinyltransferase
hypothetical protein LIC12548
hypothetical protein LIC12557
hypothetical protein LIC12568
hypothetical protein LIC12639
hypothetical protein LIC12645
hypothetical protein LIC12649
hypothetical protein LIC12652
exodeoxyribonuclease v alpha chain
glutamate synthase (NADPH) alpha chain precursor
glutamate synthase (NADPH) alpha chain precursor
glutamate synthase (NADPH) alpha chain precursor
glutamate synthase (NADPH) alpha chain precursor
metalloprotease
metalloprotease
polynucleotide phosphorylase/polyadenylase
polynucleotide phosphorylase/polyadenylase
hypothetical protein LIC12731
site-specific modification DNA-methyltransferase
putative nicotinate-nucleotide adenylyltransferase
gamma-glutamyl phosphate reductase
gamma-glutamyl phosphate reductase
anti-sigma factor antagonist
acetyl-CoA acetyltransferase
acetyl-CoA acetyltransferase

citrate synthase
preprotein translocase subunit SecY
30S ribosomal protein S8
50S ribosomal protein L16
30S ribosomal protein S19
elongation factor Tu
cytoplasmic membrane protein
cytoplasmic membrane protein
hypothetical protein LIC12923
nitrogen regulatory IIA protein
hypothetical protein LIC12970
hypothetical protein LIC12974
hypothetical protein LIC12980
hypothetical protein LIC13022
acriflavin resistance
hypothetical protein LIC13081
hypothetical protein LIC13083
acyltransferase
amino-sugar biosynthesis protein
hypothetical protein LIC13186
major facilitator superfamily permease
phenylalanyl-tRNA synthetase beta chain
phenylalanyl-tRNA synthetase beta chain
ATP-dependent RNA helicase
hypothetical protein LIC13245
putative lipoprotein
glutamine phosphoribosylpyrophosphate amidotransferase
hypothetical protein LIC13275
NAD dependent DNA ligase
hypothetical protein LIC13293
hypothetical protein LIC13297
acetyl-CoA acetyltransferase
ABC transporter, ATP-binding protein
fatty acid synthase subunit beta
isocitrate dehydrogenase
zinc carboxypeptidase-related protein
sulfite reductase subunit beta
FAD-dependent oxiredutase
phosphoribosylglycinamide formyltransferase 2
hydrogenase subunit
flagellar hook-associated protein FlgK
transcriptional coactivator
hypothetical protein LIC13482
DNA polymerase III >gi|45655586|ref|YP_003395.1| hypothetical protein LIC20001
DNA polymerase III >gi|45655586|ref|YP_003395.1| hypothetical protein LIC20001
DNA polymerase III >gi|45655586|ref|YP_003395.1| hypothetical protein LIC20001
DNA polymerase III >gi|45655586|ref|YP_003395.1| hypothetical protein LIC20001
DNA polymerase III >gi|45655586|ref|YP_003395.1| hypothetical protein LIC20001

DNA polymerase III >gi|45655586|ref|YP_003395.1| hypothetical protein LIC20001
DNA polymerase III >gi|45655586|ref|YP_003395.1| hypothetical protein LIC20001
DNA polymerase III >gi|45655586|ref|YP_003395.1| hypothetical protein LIC20001
DNA polymerase III >gi|45655586|ref|YP_003395.1| hypothetical protein LIC20001
DNA polymerase III >gi|45655586|ref|YP_003395.1| hypothetical protein LIC20001
DNA polymerase III >gi|45655586|ref|YP_003395.1| hypothetical protein LIC20001
valyl-tRNA synthetase
valyl-tRNA synthetase
valyl-tRNA synthetase
valyl-tRNA synthetase
valyl-tRNA synthetase
valyl-tRNA synthetase
valyl-tRNA synthetase
valyl-tRNA synthetase
valyl-tRNA synthetase
valyl-tRNA synthetase
valyl-tRNA synthetase
leucyl-tRNA synthetase
leucyl-tRNA synthetase
leucyl-tRNA synthetase
leucyl-tRNA synthetase
leucyl-tRNA synthetase
leucyl-tRNA synthetase
leucyl-tRNA synthetase
leucyl-tRNA synthetase
leucyl-tRNA synthetase
leucyl-tRNA synthetase
leucyl-tRNA synthetase
F0F1 ATP synthase subunit alpha
F0F1 ATP synthase subunit alpha
F0F1 ATP synthase subunit alpha
F0F1 ATP synthase subunit alpha
F0F1 ATP synthase subunit alpha
F0F1 ATP synthase subunit alpha
F0F1 ATP synthase subunit alpha
F0F1 ATP synthase subunit alpha
F0F1 ATP synthase subunit alpha
F0F1 ATP synthase subunit alpha
F0F1 ATP synthase subunit alpha
F0F1 ATP synthase subunit alpha
F0F1 ATP synthase subunit alpha
F0F1 ATP synthase subunit alpha
F0F1 ATP synthase subunit alpha
F0F1 ATP synthase subunit alpha
F0F1 ATP synthase subunit alpha
F0F1 ATP synthase subunit alpha
F0F1 ATP synthase subunit alpha
F0F1 ATP synthase subunit alpha

F0F1 ATP synthase subunit alpha
F0F1 ATP synthase subunit alpha
F0F1 ATP synthase subunit alpha
F0F1 ATP synthase subunit alpha
bifunctional GMP synthase/glutamine amidotransferase
bifunctional GMP synthase/glutamine amidotransferase
bifunctional GMP synthase/glutamine amidotransferase
bifunctional GMP synthase/glutamine amidotransferase
bifunctional GMP synthase/glutamine amidotransferase
bifunctional GMP synthase/glutamine amidotransferase
bifunctional GMP synthase/glutamine amidotransferase
bifunctional GMP synthase/glutamine amidotransferase
ATP-dependent protease ClpP
ATP-dependent protease ClpP
ATP-dependent protease ClpP
ATP-dependent protease ClpP
ATP-dependent protease ClpP
ATP-dependent protease ClpP
ATP-dependent protease ClpP
ATP-dependent protease ClpP
30S ribosomal protein S15
30S ribosomal protein S15
30S ribosomal protein S15
30S ribosomal protein S15
valine--pyruvate aminotransferase
valine--pyruvate aminotransferase
valine--pyruvate aminotransferase
valine--pyruvate aminotransferase
valine--pyruvate aminotransferase
valine--pyruvate aminotransferase
S-adenosylmethionine synthetase
F0F1 ATP synthase subunit gamma
F0F1 ATP synthase subunit gamma
F0F1 ATP synthase subunit gamma
F0F1 ATP synthase subunit gamma
F0F1 ATP synthase subunit gamma
F0F1 ATP synthase subunit gamma
F0F1 ATP synthase subunit gamma
F0F1 ATP synthase subunit gamma
hypothetical protein LA_0018
hypothetical protein LA_0018
hypothetical protein LA_0018
hypothetical protein LA_0018
hypothetical protein LA_0018
glutaredoxin family protein
TPR-repeat-containing protein
hypothetical protein LA_0070
hypothetical protein LA_0070

hypothetical protein LA_0071
hypothetical protein LA_0071
hypothetical protein LA_0071
dioxygenase superfamily protein
dioxygenase superfamily protein
dioxygenase superfamily protein
dioxygenase superfamily protein
dioxygenase superfamily protein
1-acylglycerol-3-phosphate O-acyltransferase
1-acylglycerol-3-phosphate O-acyltransferase
hypothetical protein LA_0095
hypothetical protein LA_0095
long-chain-fatty-acid CoA ligase
long-chain-fatty-acid CoA ligase
long-chain-fatty-acid CoA ligase
long-chain-fatty-acid CoA ligase
long-chain-fatty-acid CoA ligase
long-chain-fatty-acid CoA ligase
long-chain-fatty-acid CoA ligase
long-chain-fatty-acid CoA ligase
long-chain-fatty-acid CoA ligase
long-chain-fatty-acid CoA ligase
long-chain-fatty-acid CoA ligase
hypothetical protein LA_0129
hypothetical protein LA_0129
hypothetical protein LA_0129
hypothetical protein LA_0129
hypothetical protein LA_0129
hypothetical protein LA_0129
hypothetical protein LA_0136
hypothetical protein LA_0136
hypothetical protein LA_0136
hypothetical protein LA_0136
hypothetical protein LA_0137
hypothetical protein LA_0137
hypothetical protein LA_0137
hypothetical protein LA_0137
hypothetical protein LA_0137
hypothetical protein LA_0137
hypothetical protein LA_0137
hypothetical protein LA_0137
hypothetical protein LA_0137
hypothetical protein LA_0137
hypothetical protein LA_0137
hypothetical protein LA_0137
hypothetical protein LA_0137
hypothetical protein LA_0137
hypothetical protein LA_0172

hypothetical protein LA_0172
hypothetical protein LA_0172
hypothetical protein LA_0172
hypothetical protein LA_0172
malic enzyme
malic enzyme
malic enzyme
malic enzyme
transcriptional regulator
transcriptional regulator
hypothetical protein LA_0234
cytochrome c oxidase subunit II
cytochrome c oxidase subunit II
cytochrome c oxidase subunit II
cytochrome c oxidase subunit II
cytochrome c oxidase subunit II
cytochrome c oxidase subunit II
cytochrome c oxidase subunit II
cytochrome c oxidase subunit II
cytochrome c oxidase subunit II
transcriptional regulator
transcriptional regulator
transcriptional regulator
transcriptional regulator
transcriptional regulator
transcriptional regulator
hypothetical protein LA_0254
DNA polymerase III subunit alpha
DNA polymerase III subunit alpha
DNA polymerase III subunit alpha
RNA polymerase ECF-type sigma factor
RNA polymerase ECF-type sigma factor
hypothetical protein LA_0268
hypothetical protein LA_0268
hypothetical protein LA_0268
hypothetical protein LA_0268
lipoprotein releasing system permease
lipoprotein releasing system permease
lipoprotein releasing system permease
lipoprotein releasing system permease
lipoprotein releasing system permease
lipoprotein releasing system permease
lipoprotein releasing system permease
lipoprotein releasing system permease
exonuclease
exonuclease
exonuclease
Zn-dependent alcohol dehydrogenase

Zn-dependent alcohol dehydrogenase
Zn-dependent alcohol dehydrogenase
Zn-dependent alcohol dehydrogenase
Zn-dependent alcohol dehydrogenase
Zn-dependent alcohol dehydrogenase
Zn-dependent alcohol dehydrogenase
Zn-dependent alcohol dehydrogenase
Zn-dependent alcohol dehydrogenase
Zn-dependent alcohol dehydrogenase
Zn-dependent alcohol dehydrogenase
Zn-dependent alcohol dehydrogenase
Zn-dependent alcohol dehydrogenase
hypothetical protein LA_0323
geranylgeranyl pyrophosphate synthase
geranylgeranyl pyrophosphate synthase
glycerophosphoryl diester phosphodiesterase
glycerophosphoryl diester phosphodiesterase
glycerophosphoryl diester phosphodiesterase
glycine cleavage system aminomethyltransferase T
glycine cleavage system aminomethyltransferase T
glycine cleavage system aminomethyltransferase T
glycine cleavage system aminomethyltransferase T
glycine cleavage system aminomethyltransferase T
glycine cleavage system aminomethyltransferase T
glycine cleavage system aminomethyltransferase T
glycine cleavage system aminomethyltransferase T
glycine cleavage system aminomethyltransferase T
glycine cleavage system aminomethyltransferase T
glycine cleavage system aminomethyltransferase T
phosphoserine aminotransferase
phosphoserine aminotransferase
phosphoserine aminotransferase
phosphoserine aminotransferase
phosphoserine aminotransferase
phosphoserine aminotransferase
phosphoserine aminotransferase
phosphoserine aminotransferase
hypothetical protein LA_0370
hypothetical protein LA_0370
hypothetical protein LA_0370
hypothetical protein LA_0370
hypothetical protein LA_0370
hypothetical protein LA_0370
TPR-repeat-containing protein
TPR-repeat-containing protein
TPR-repeat-containing protein
TPR-repeat-containing protein
TPR-repeat-containing protein

TPR-repeat-containing protein
hypothetical protein LA_0379
hypothetical protein LA_0379
hypothetical protein LA_0379
hypothetical protein LA_0379
hypothetical protein LA_0379
hypothetical protein LA_0379
hypothetical protein LA_0379
hypothetical protein LA_0389
hypothetical protein LA_0389
hypothetical protein LA_0389
hypothetical protein LA_0400
hypothetical protein LA_0400
phosphoglyceromutase
phosphoglyceromutase
phosphoglyceromutase
phosphoglyceromutase
phosphoglyceromutase
phosphoglyceromutase
phosphoglyceromutase
phosphoglyceromutase
phosphoglyceromutase
phosphoglyceromutase
hypothetical protein LA_0444
hypothetical protein LA_0444
rhodanese-like protein
phosphohydrolase
hypothetical protein LA_0478
transcriptional regulator
transcriptional regulator
transcriptional regulator
cation/multidrug efflux pump
cation/multidrug efflux pump
cation/multidrug efflux pump
cation/multidrug efflux pump
cation/multidrug efflux pump
cation/multidrug efflux pump
cation/multidrug efflux pump
cation/multidrug efflux pump
cation/multidrug efflux pump
cation/multidrug efflux pump
cation/multidrug efflux pump
cation/multidrug efflux pump
TonB-dependent outer membrane receptor
bifunctional permease/carbonic anhydrase
bifunctional permease/carbonic anhydrase
bifunctional permease/carbonic anhydrase
bifunctional permease/carbonic anhydrase

bifunctional permease/carbonic anhydrase
bifunctional permease/carbonic anhydrase
bifunctional permease/carbonic anhydrase
bifunctional permease/carbonic anhydrase
transcriptional regulator
transcriptional regulator
transcriptional regulator
transcriptional regulator
transcriptional regulator
transcriptional regulator
transcriptional regulator
transcriptional regulator
SET family protein
hypothetical protein LA_0616
hypothetical protein LA_0616
hypothetical protein LA_0616
hypothetical protein LA_0616
hypothetical protein LA_0616
hypothetical protein LA_0616
hypothetical protein LA_0616
hypothetical protein LA_0616
hypothetical protein LA_0616
hypothetical protein LA_0616
hypothetical protein LA_0616
hypothetical protein LA_0616
hypothetical protein LA_0616
hypothetical protein LA_0616
hypothetical protein LA_0616
hypothetical protein LA_0616
hypothetical protein LA_0616
hypothetical protein LA_0616
hypothetical protein LA_0616
hypothetical protein LA_0616
hypothetical protein LA_0616
hypothetical protein LA_0616
hypothetical protein LA_0616
hypothetical protein LA_0616
hypothetical protein LA_0616
hypothetical protein LA_0616
hypothetical protein LA_0616
hypothetical protein LA_0616
hypothetical protein LA_0616
hypothetical protein LA_0616
hypothetical protein LA_0616
hypothetical protein LA_0616
quinolinate synthetase
quinolinate synthetase
quinolinate synthetase

quinolinate synthetase
quinolinate synthetase
glycosyltransferase
oxidoreductase-like protein
phosphoenolpyruvate-dependent enzyme IIA component of the PTS system
hypothetical protein LA_0639
hypothetical protein LA_0644
hypothetical protein LA_0644
anti-sigma factor antagonist
anti-sigma factor antagonist
anti-sigma factor antagonist
anti-sigma factor antagonist
hypothetical protein LA_0654
hypothetical protein LA_0654
hypothetical protein LA_0654
hypothetical protein LA_0654
endoflagellar motor protein A
methyl-accepting chemotaxis protein
methyl-accepting chemotaxis protein
methyl-accepting chemotaxis protein
methyl-accepting chemotaxis protein
methyl-accepting chemotaxis protein
hypothetical protein LA_0709
Holliday junction endodeoxyribonuclease protein
30S ribosomal protein S17
30S ribosomal protein S17
30S ribosomal protein S5
30S ribosomal protein S5
30S ribosomal protein S5
30S ribosomal protein S5
30S ribosomal protein S5
30S ribosomal protein S5
30S ribosomal protein S5
30S ribosomal protein S5
30S ribosomal protein S5
hypothetical protein LA_0777
rRNA methylase
rRNA methylase
hypothetical protein LA_0818
hypothetical protein LA_0818
methionine sulfoxide reductase B
methionine sulfoxide reductase B
biotin--[acetyl-CoA-carboxylase] ligase
biotin--[acetyl-CoA-carboxylase] ligase
biotin--[acetyl-CoA-carboxylase] ligase
biotin--[acetyl-CoA-carboxylase] ligase
biotin--[acetyl-CoA-carboxylase] ligase
dolichyl-phosphate-mannose-protein mannosyltransferase

dolichyl-phosphate-mannose-protein mannosyltransferase
hypothetical protein LA_0858
aldehyde dehydrogenase
aldehyde dehydrogenase
aldehyde dehydrogenase
aldehyde dehydrogenase
aldehyde dehydrogenase
aldehyde dehydrogenase
aldehyde dehydrogenase
aldehyde dehydrogenase
NADH dehydrogenase (ubiquinone) chain E
NADH dehydrogenase (ubiquinone) chain E
4-hydroxybenzoate transporter transmembrane protein
4-hydroxybenzoate transporter transmembrane protein
hypothetical protein LA_0941
translation initiation factor IF-2
translation initiation factor IF-2
translation initiation factor IF-2
translation initiation factor IF-2
translation initiation factor IF-2
translation initiation factor IF-2
translation initiation factor IF-2
translation initiation factor IF-2
translation initiation factor IF-2
translation initiation factor IF-2
translation initiation factor IF-2
translation initiation factor IF-2
translation initiation factor IF-2
translation initiation factor IF-2
translation initiation factor IF-2
translation initiation factor IF-2
translation initiation factor IF-2
translation initiation factor IF-2
translation initiation factor IF-2
translation initiation factor IF-2
translation initiation factor IF-2
translation initiation factor IF-2
Zn-dependent peptidase
Zn-dependent peptidase
Zn-dependent peptidase
hypothetical protein LA_0958
hypothetical protein LA_0958
hypothetical protein LA_0958
hypothetical protein LA_0960
hypothetical protein LA_0960
exodeoxyribonuclease V subunit alpha
drug proton antiporter
hypothetical protein LA_1090

lipid-a-disaccharide synthase
cytoplasmic membrane protein
cytoplasmic membrane protein
cytoplasmic membrane protein
cytoplasmic membrane protein
hypothetical protein LA_1122
CDP-diacylglycerol--glycerol-3-phosphate 3- phosphatidyltransferase
hypothetical protein LA_1141a
hypothetical protein LA_1141a
hypothetical protein LA_1141a
preprotein translocase subunit SecD
preprotein translocase subunit SecD
preprotein translocase subunit SecD
preprotein translocase subunit SecD
preprotein translocase subunit SecD
preprotein translocase subunit SecD
preprotein translocase subunit SecD
preprotein translocase subunit SecD
preprotein translocase subunit SecD
preprotein translocase subunit SecD
preprotein translocase subunit SecD
preprotein translocase subunit SecD
preprotein translocase subunit SecD
preprotein translocase subunit SecF
preprotein translocase subunit SecF
sulfate ABC transporter permease
sulfate ABC transporter permease
ABC transporter ATP-binding protein
ABC transporter ATP-binding protein
ABC transporter ATP-binding protein
hypothetical protein LA_1196
hypothetical protein LA_1196
hypothetical protein LA_1200
intramembrane serine protease
carbamoyl phosphate synthase small subunit
carbamoyl phosphate synthase small subunit
carbamoyl phosphate synthase small subunit
carbamoyl phosphate synthase small subunit
carbamoyl phosphate synthase small subunit
bifunctional prephenate dehydratase/chorismate mutase
bifunctional prephenate dehydratase/chorismate mutase
bifunctional prephenate dehydratase/chorismate mutase
bifunctional prephenate dehydratase/chorismate mutase
bifunctional prephenate dehydratase/chorismate mutase
bifunctional prephenate dehydratase/chorismate mutase
bifunctional prephenate dehydratase/chorismate mutase
ATP phosphoribosyltransferase
ATP phosphoribosyltransferase

ATP phosphoribosyltransferase
ATP phosphoribosyltransferase
transcriptional regulator
transcriptional regulator
transcriptional regulator
transcriptional regulator
hypothetical protein LA_1295
hypothetical protein LA_1295
hypothetical protein LA_1295
hypothetical protein LA_1295
hypothetical protein LA_1295
hypothetical protein LA_1295
hypothetical protein LA_1295
hypothetical protein LA_1295
hypothetical protein LA_1295
hypothetical protein LA_1295
hypothetical protein LA_1295
hypothetical protein LA_1295
chromosome segregation protein
hypothetical protein LA_1312a
anti-sigma factor antagonist
hypothetical protein LA_1328
hypothetical protein LA_1328
hypothetical protein LA_1328
hypothetical protein LA_1328
hypothetical protein LA_1328
hypothetical protein LA_1328
hypothetical protein LA_1328
ankyrin repeat-containing protein
two-component system signal receiver
two-component system signal receiver
two-component system signal receiver
transcriptional regulator
transcriptional regulator
hypothetical protein LA_1366
pirin-like protein
pirin-like protein
pirin-like protein
hypothetical protein LA_1389
hypothetical protein LA_1389
hypothetical protein LA_1396
hypothetical protein LA_1396
hypothetical protein LA_1396
hypothetical protein LA_1412
hypothetical protein LA_1412
hypothetical protein LA_1412
hypothetical protein LA_1412
hypothetical protein LA_1412

hypothetical protein LA_1412
adenylate/guanylate cyclase
adenylate/guanylate cyclase
adenylate/guanylate cyclase
adenylate/guanylate cyclase
adenylate/guanylate cyclase
adenylate/guanylate cyclase
short chain dehydrogenase
short chain dehydrogenase
transcriptional repressor LexA
transcriptional repressor LexA
oligo-1,6-glucosidase
membrane-bound proton-translocating pyrophosphatase
dehalogenase-like hydrolase
pyrroline-5-carboxylate reductase
pyrroline-5-carboxylate reductase
hypothetical protein LA_1507
hypothetical protein LA_1507
hypothetical protein LA_1507
hypothetical protein LA_1507
hypothetical protein LA_1507
hypothetical protein LA_1507
carboxy-terminal processing protease
carboxy-terminal processing protease
carboxy-terminal processing protease
carboxy-terminal processing protease
carboxy-terminal processing protease
carboxy-terminal processing protease
carboxy-terminal processing protease
carboxy-terminal processing protease
carboxy-terminal processing protease
carboxy-terminal processing protease
carboxy-terminal processing protease
carboxy-terminal processing protease
carboxy-terminal processing protease
carboxy-terminal processing protease
carboxy-terminal processing protease
hypothetical protein LA_1515
hypothetical protein LA_1515
hypothetical protein LA_1517a
thymidylate synthase
thymidylate synthase
thymidylate synthase
thymidylate synthase
thymidylate synthase
thymidylate synthase
thymidylate synthase
sodium/hydrogen antiporter

sodium/hydrogen antiporter
sodium/hydrogen antiporter
sodium/hydrogen antiporter
ADP-heptose synthetase
ADP-heptose synthetase
ADP-heptose synthetase
hypothetical protein LA_1586
hypothetical protein LA_1586
hypothetical protein LA_1586
hypothetical protein LA_1586
hypothetical protein LA_1586
hypothetical protein LA_1620
dTDP-4-dehydrorhamnose epimerase
dTDP-4-dehydrorhamnose epimerase
dTDP-4-dehydrorhamnose epimerase
dTDP-4-dehydrorhamnose epimerase
hypothetical protein LA_1639
hypothetical protein LA_1639
dTDP-D-glucose 4,6-dehydratase
dTDP-D-glucose 4,6-dehydratase
dTDP-D-glucose 4,6-dehydratase
dTDP-D-glucose 4,6-dehydratase
hypothetical protein LA_1686
arginyl-tRNA synthetase
arginyl-tRNA synthetase
arginyl-tRNA synthetase
arginyl-tRNA synthetase
arginyl-tRNA synthetase
arginyl-tRNA synthetase
signal peptidase I
signal peptidase I
signal peptidase I
signal peptidase I
carbonic anhydrase/acetyltransferase
histidine kinase/response regulator hybrid protein
histidine kinase/response regulator hybrid protein
histidine kinase/response regulator hybrid protein
hypothetical protein LA_1827
hypothetical protein LA_1827
catalase
catalase
transcriptional regulator
hypothetical protein LA_1950
hypothetical protein LA_1950
hypothetical protein LA_1958
hypothetical protein LA_1958
hypothetical protein LA_1958
preprotein translocase subunit SecA

preprotein translocase subunit SecA
preprotein translocase subunit SecA
preprotein translocase subunit SecA
preprotein translocase subunit SecA
preprotein translocase subunit SecA
preprotein translocase subunit SecA
preprotein translocase subunit SecA
preprotein translocase subunit SecA
preprotein translocase subunit SecA
preprotein translocase subunit SecA
preprotein translocase subunit SecA
preprotein translocase subunit SecA
preprotein translocase subunit SecA
preprotein translocase subunit SecA
preprotein translocase subunit SecA
preprotein translocase subunit SecA
preprotein translocase subunit SecA
preprotein translocase subunit SecA
preprotein translocase subunit SecA
preprotein translocase subunit SecA
hypothetical protein LA_1965
cation efflux system protein
cation efflux system protein
cation efflux system protein
cation efflux system protein
cation efflux system protein
cation efflux system protein
cation efflux system protein
hypothetical protein LA_1968
hypothetical protein LA_1968
hypothetical protein LA_1968
hypothetical protein LA_1968
hypothetical protein LA_1999
hypothetical protein LA_1999
hypothetical protein LA_1999
hypothetical protein LA_1999
hypothetical protein LA_1999
hypothetical protein LA_2004a
hypothetical protein LA_2025
hypothetical protein LA_2025
hypothetical protein LA_2025
hypothetical protein LA_2025
hypothetical protein LA_2025
hypothetical protein LA_2025
hypothetical protein LA_2025
hypothetical protein LA_2025
hypothetical protein LA_2026
hypothetical protein LA_2030

hypothetical protein LA_2030
hypothetical protein LA_2031
hypothetical protein LA_2031
hydrolase
hydrolase
hydrolase
HIT family hydrolase
HIT family hydrolase
HIT family hydrolase
UDP-N-acetylmuramoylalanyl-D-glutamate--2,6- diaminopimelate ligase
UDP-N-acetylmuramoylalanyl-D-glutamate--2,6- diaminopimelate ligase
UDP-N-acetylmuramoylalanyl-D-glutamate--2,6- diaminopimelate ligase
UDP-N-acetylmuramoylalanyl-D-glutamate--2,6- diaminopimelate ligase
UDP-N-acetylmuramate--L-alanine ligase
UDP-N-acetylmuramate--L-alanine ligase
UDP-N-acetylmuramate--L-alanine ligase
UDP-N-acetylmuramate--L-alanine ligase
hypothetical protein LA_2080
hypothetical protein LA_2083
hypothetical protein LA_2083
hypothetical protein LA_2083
7-cyano-7-deazaguanine reductase
7-cyano-7-deazaguanine reductase
7-cyano-7-deazaguanine reductase
FHA domain-containing protein
ATP-dependent protease ClpP
ATP-dependent protease ClpP
gamma-glutamylcysteine synthetase
gamma-glutamylcysteine synthetase
gamma-glutamylcysteine synthetase
gamma-glutamylcysteine synthetase
biotin synthase
SGNH-hydrolase
SGNH-hydrolase
SGNH-hydrolase
endonuclease III
endonuclease III
endonuclease III
endonuclease III
exopolysaccharide production protein
hypothetical protein LA_2174
hypothetical protein LA_2174
hypothetical protein LA_2174
hypothetical protein LA_2174
hypothetical protein LA_2174
hypothetical protein LA_2174
hypothetical protein LA_2174
alpha-beta hydrolase family esterase

Zn-dependent protease
Zn-dependent protease
Zn-dependent protease
Zn-dependent protease
Zn-dependent protease
peptidyl-prolyl cis-trans isomerase
peptidyl-prolyl cis-trans isomerase
hypothetical protein LA_2213
hypothetical protein LA_2213
endoflagellar motor protein B
endoflagellar motor protein B
endoflagellar motor protein B
endoflagellar motor protein B
magnesium transporter
magnesium transporter
magnesium transporter
magnesium transporter
magnesium transporter
magnesium transporter
methyl-accepting chemotaxis protein
methyl-accepting chemotaxis protein
methyl-accepting chemotaxis protein
endonuclease
transcriptional regulator
transcriptional regulator
transcriptional regulator
deoxyguanosinetriphosphate triphosphohydrolase
deoxyguanosinetriphosphate triphosphohydrolase
hypothetical protein LA_2278
hypothetical protein LA_2289
MBOAT family D-alaninealginate export/acetyltransferase membrane protein
hypothetical protein LA_2295
hypothetical protein LA_2295
hypothetical protein LA_2295
hypothetical protein LA_2295
hypothetical protein LA_2295
hypothetical protein LA_2295
hypothetical protein LA_2295
hypothetical protein LA_2295
hypothetical protein LA_2295
hypothetical protein LA_2295
hypothetical protein LA_2295
hypothetical protein LA_2295
hypothetical protein LA_2295
hypothetical protein LA_2295
hypothetical protein LA_2295
hypothetical protein LA_2295
hypothetical protein LA_2295

UDP-3-O-[3-hydroxymyristoyl] N-acetylglucosamine deacetylase
UDP-3-O-[3-hydroxymyristoyl] N-acetylglucosamine deacetylase
UDP-3-O-[3-hydroxymyristoyl] N-acetylglucosamine deacetylase
UDP-3-O-[3-hydroxymyristoyl] N-acetylglucosamine deacetylase
UDP-3-O-[3-hydroxymyristoyl] N-acetylglucosamine deacetylase
nucleoside-diphosphate-sugar epimerase
nucleoside-diphosphate-sugar epimerase
nucleoside-diphosphate-sugar epimerase
nucleoside-diphosphate-sugar epimerase
nucleoside-diphosphate-sugar epimerase
nucleoside-diphosphate-sugar epimerase
nucleoside-diphosphate-sugar epimerase
nucleoside-diphosphate-sugar epimerase
glucose/sorbosone dehydrogenase
glucose/sorbosone dehydrogenase
glucose/sorbosone dehydrogenase
glucose/sorbosone dehydrogenase
hypothetical protein LA_2353
hypothetical protein LA_2353
hypothetical protein LA_2353
hypothetical protein LA_2353
ribonucleotide reductase subunit alpha
ribonucleotide reductase subunit alpha
ribonucleotide reductase subunit alpha
ribonucleotide reductase subunit alpha
ribonucleotide reductase subunit alpha
ribonucleotide reductase subunit alpha
ribonucleotide reductase subunit alpha
ribonucleotide reductase subunit alpha
ribonucleotide reductase subunit alpha
ribonucleotide reductase subunit alpha
ribonucleotide reductase subunit alpha
ribonucleotide reductase subunit alpha
ribonucleotide reductase subunit alpha
ribonucleotide reductase subunit alpha
ribonucleotide reductase subunit alpha
ribonucleotide reductase subunit alpha
ribonucleotide reductase subunit alpha
type II secretory pathway component protein D
type II secretory pathway component protein D
type II secretory pathway component protein D
type II secretory pathway component protein D
type II secretory pathway component protein D
type II secretory pathway component protein D
type II secretory pathway component protein D
hypothetical protein LA_2378
ribonuclease H II
30S ribosomal protein S16

30S ribosomal protein S16
methionyl-tRNA formyltransferase
methionyl-tRNA formyltransferase
methionyl-tRNA formyltransferase
methionyl-tRNA formyltransferase
methionyl-tRNA formyltransferase
ABC transporter ATP-binding protein
ABC transporter ATP-binding protein
ABC transporter ATP-binding protein
2-dehydro-3-deoxyphosphooctonate aldolase
2-dehydro-3-deoxyphosphooctonate aldolase
2-dehydro-3-deoxyphosphooctonate aldolase
2-dehydro-3-deoxyphosphooctonate aldolase
2-dehydro-3-deoxyphosphooctonate aldolase
2-dehydro-3-deoxyphosphooctonate aldolase
CTP synthetase
CTP synthetase
CTP synthetase
CTP synthetase
CTP synthetase
CTP synthetase
CTP synthetase
CTP synthetase
CTP synthetase
endoflagellar hook-filament protein
endoflagellar hook-filament protein
endoflagellar hook-filament protein
endoflagellar hook-filament protein
endoflagellar hook-filament protein
penicillin tolerance protein
penicillin tolerance protein
penicillin tolerance protein
histidine kinase/response regulator hybrid protein
hypothetical protein LA_2436
hypothetical protein LA_2436
hypothetical protein LA_2443
methionyl aminopeptidase
methionyl aminopeptidase
methionyl aminopeptidase
methionyl aminopeptidase
methionyl aminopeptidase
hypothetical protein LA_2458
hypothetical protein LA_2458
hypothetical protein LA_2458
hypothetical protein LA_2458
polymerase
polymerase
hypothetical protein LA_2497

hypothetical protein LA_2497
hypothetical protein LA_2497
hypothetical protein LA_2497
hypothetical protein LA_2497
hypothetical protein LA_2497
hypothetical protein LA_2497
hypothetical protein LA_2497
glycosyltransferase
glycosyltransferase
exodeoxyribonuclease
exodeoxyribonuclease
hypothetical protein LA_2531
hypothetical protein LA_2531
hypothetical protein LA_2531
hypothetical protein LA_2531
phosphate sodium symporter
phosphate sodium symporter
phosphate sodium symporter
phosphate sodium symporter
phosphate sodium symporter
phosphate sodium symporter
phosphate sodium symporter
phosphate sodium symporter
phosphate sodium symporter
acyl-CoA thioesterase
acyl-CoA thioesterase
protein kinase
sodium:alanine symporter/phosphatidylserine decarboxylase
sodium:alanine symporter/phosphatidylserine decarboxylase
sodium:alanine symporter/phosphatidylserine decarboxylase
short-chain dehydrogenase
short-chain dehydrogenase
short-chain dehydrogenase
short-chain dehydrogenase
short-chain dehydrogenase
short-chain dehydrogenase
short-chain dehydrogenase
short-chain dehydrogenase
S-adenosylmethionine synthetase
S-adenosylmethionine synthetase
S-adenosylmethionine synthetase
S-adenosylmethionine synthetase
S-adenosylmethionine synthetase
S-adenosylmethionine synthetase
S-adenosylmethionine synthetase
S-adenosylmethionine synthetase
S-adenosylmethionine synthetase
S-adenosylmethionine synthetase

S-adenosylmethionine synthetase
S-adenosylmethionine synthetase
S-adenosylmethionine synthetase
S-adenosylmethionine synthetase
S-adenosylmethionine synthetase
adenylate/guanylate cyclase
biotin carboxylase
biotin carboxylase
biotin carboxylase
biotin carboxylase
flagellar basal body rod protein FlgG
flagellar basal body rod protein FlgG
lipopolysaccharide heptosyltransferase II
lipopolysaccharide heptosyltransferase II
bacterioferritin
bacterioferritin
bacterioferritin
Fe-S oxidoreductase
Fe-S oxidoreductase
Fe-S oxidoreductase
Fe-S oxidoreductase
Fe-S oxidoreductase
Fe-S oxidoreductase
Fe-S oxidoreductase
Fe-S oxidoreductase
Fe-S oxidoreductase
Fe-S oxidoreductase
Fe-S oxidoreductase
Fe-S oxidoreductase
Fe-S oxidoreductase
Fe-S oxidoreductase
hypothetical protein LA_2726
hypothetical protein LA_2726
hypothetical protein LA_2726
acetyl-CoA carboxylase biotin carboxyl carrier protein subunit
acetyl-CoA carboxylase biotin carboxyl carrier protein subunit
hypothetical protein LA_2742
hypothetical protein LA_2746
hypothetical protein LA_2746
hypothetical protein LA_2746
rod shape-determining protein
rod shape-determining protein
rod shape-determining protein MreC
cGMP-specific phosphodiesterase
cGMP-specific phosphodiesterase
cGMP-specific phosphodiesterase
cGMP-specific phosphodiesterase
F0F1 ATP synthase subunit beta

F0F1 ATP synthase subunit beta
F0F1 ATP synthase subunit beta
F0F1 ATP synthase subunit beta
F0F1 ATP synthase subunit beta
F0F1 ATP synthase subunit beta
F0F1 ATP synthase subunit beta
F0F1 ATP synthase subunit beta
F0F1 ATP synthase subunit beta
F0F1 ATP synthase subunit beta
F0F1 ATP synthase subunit beta
F0F1 ATP synthase subunit beta
F0F1 ATP synthase subunit beta
F0F1 ATP synthase subunit beta
F0F1 ATP synthase subunit beta
F0F1 ATP synthase subunit beta
F0F1 ATP synthase subunit beta
F0F1 ATP synthase subunit beta
F0F1 ATP synthase subunit beta
F0F1 ATP synthase subunit beta
ATP synthase C chain
F0F1 ATP synthase subunit A
F0F1 ATP synthase subunit A
hypothetical protein LA_2800
hypothetical protein LA_2800
hypothetical protein LA_2800
methyl-accepting chemotaxis protein
methyl-accepting chemotaxis protein
methyl-accepting chemotaxis protein
methyl-accepting chemotaxis protein
methyl-accepting chemotaxis protein
methyl-accepting chemotaxis protein
two component system sensor histidine kinase\response regulator
citrate lyase
citrate lyase
citrate lyase
citrate lyase
citrate lyase
citrate lyase
citrate lyase
citrate lyase
citrate lyase
citrate lyase
citrate lyase
citrate lyase
MhpC-like protein
MhpC-like protein
MhpC-like protein
MhpC-like protein

dihydrolipoamide dehydrogenase
pyruvate kinase
pyruvate kinase
pyruvate kinase
pyruvate kinase
pyruvate kinase
pyruvate kinase
phosphoribosylaminoimidazole synthetase
phosphoribosylaminoimidazole synthetase
phosphoribosylaminoimidazole synthetase
phosphoribosylaminoimidazole synthetase
phosphoribosylaminoimidazole synthetase
hypothetical protein LA_2936
hypothetical protein LA_2936
thiosulfate sulfurtransferase
thiosulfate sulfurtransferase
thiosulfate sulfurtransferase
thiosulfate sulfurtransferase
thiosulfate sulfurtransferase
thiosulfate sulfurtransferase
thiosulfate sulfurtransferase
thiosulfate sulfurtransferase
thiosulfate sulfurtransferase
thiosulfate sulfurtransferase
antimicrobial peptide ABC transporter ATPase
antimicrobial peptide ABC transporter ATPase
hypothetical protein LA_3017
Fe-S metabolism protein
Fe-S metabolism protein
nickel ABC transporter periplasmic protein OppA
nickel ABC transporter periplasmic protein OppA
nickel ABC transporter periplasmic protein OppA
nickel ABC transporter periplasmic protein OppA
nickel ABC transporter periplasmic protein OppA
nickel ABC transporter periplasmic protein OppA
nickel ABC transporter periplasmic protein OppA
nickel ABC transporter periplasmic protein OppA
nickel ABC transporter periplasmic protein OppA
nickel ABC transporter periplasmic protein OppA
nickel ABC transporter periplasmic protein OppA
nickel ABC transporter periplasmic protein OppA
guanosine polyphosphate pyrophosphohydrolase/synthetase
guanosine polyphosphate pyrophosphohydrolase/synthetase
hypothetical protein LA_3091
hypothetical protein LA_3091
hypothetical protein LA_3091
hypothetical protein LA_3091
hypothetical protein LA_3091

OmpL1
OmpL1
OmpL1
OmpL1
OmpL1
OmpL1
OmpL1
OmpL1
OmpL1
OmpL1
alpha/beta hydrolase superfamily protein
alpha/beta hydrolase superfamily protein
alpha/beta hydrolase superfamily protein
hypothetical protein LA_3162
serine/threonine phosphatase
serine/threonine phosphatase
serine/threonine phosphatase
serine/threonine phosphatase
serine/threonine phosphatase
serine/threonine phosphatase
hypothetical protein LA_3200b
hypothetical protein LA_3200b
hypothetical protein LA_3219
hypothetical protein LA_3219
hypothetical protein LA_3219
hypothetical protein LA_3219
hypothetical protein LA_3219
hypothetical protein LA_3219
hypothetical protein LA_3219
hypothetical protein LA_3219
hypothetical protein LA_3219
Fe-S-cluster-containing hydrogenase
Fe-S-cluster-containing hydrogenase
Fe-S-cluster-containing hydrogenase
Fe-S-cluster-containing hydrogenase
Fe-S-cluster-containing hydrogenase
Fe-S-cluster-containing hydrogenase
Fe-S-cluster-containing hydrogenase
1-deoxy-D-xylulose 5-phosphate reductoisomerase
1-deoxy-D-xylulose 5-phosphate reductoisomerase
1-deoxy-D-xylulose 5-phosphate reductoisomerase
30S ribosomal protein S2
30S ribosomal protein S2
30S ribosomal protein S2
30S ribosomal protein S2
30S ribosomal protein S2
30S ribosomal protein S2
30S ribosomal protein S2

30S ribosomal protein S2
30S ribosomal protein S2
30S ribosomal protein S2
30S ribosomal protein S2
30S ribosomal protein S2
30S ribosomal protein S2
30S ribosomal protein S2
hypothetical protein LA_3319
Zn-dependent alcohol dehydrogenase
Zn-dependent alcohol dehydrogenase
Zn-dependent alcohol dehydrogenase
enoyl-CoA hydratase
enoyl-CoA hydratase
enoyl-CoA hydratase
transcript cleavage factor/unknown domain fusion protein
transcript cleavage factor/unknown domain fusion protein
transcript cleavage factor/unknown domain fusion protein
transcript cleavage factor/unknown domain fusion protein
transcript cleavage factor/unknown domain fusion protein
transcript cleavage factor/unknown domain fusion protein
transcript cleavage factor/unknown domain fusion protein
transcript cleavage factor/unknown domain fusion protein
transcript cleavage factor/unknown domain fusion protein
transcript cleavage factor/unknown domain fusion protein
transcript cleavage factor/unknown domain fusion protein
transcript cleavage factor/unknown domain fusion protein
transcript cleavage factor/unknown domain fusion protein
transcript cleavage factor/unknown domain fusion protein
transcript cleavage factor/unknown domain fusion protein
transcript cleavage factor/unknown domain fusion protein
transcript cleavage factor/unknown domain fusion protein
transcript cleavage factor/unknown domain fusion protein
transcript cleavage factor/unknown domain fusion protein
transcript cleavage factor/unknown domain fusion protein
transcript cleavage factor/unknown domain fusion protein
transcript cleavage factor/unknown domain fusion protein
transcript cleavage factor/unknown domain fusion protein
transcript cleavage factor/unknown domain fusion protein
transcript cleavage factor/unknown domain fusion protein
transcript cleavage factor/unknown domain fusion protein
transcript cleavage factor/unknown domain fusion protein
transcript cleavage factor/unknown domain fusion protein
transcript cleavage factor/unknown domain fusion protein
transcript cleavage factor/unknown domain fusion protein
transcript cleavage factor/unknown domain fusion protein
transcript cleavage factor/unknown domain fusion protein
transcript cleavage factor/unknown domain fusion protein
transcript cleavage factor/unknown domain fusion protein

transcript cleavage factor/unknown domain fusion protein
transcript cleavage factor/unknown domain fusion protein
transcript cleavage factor/unknown domain fusion protein
transcript cleavage factor/unknown domain fusion protein
transcript cleavage factor/unknown domain fusion protein
transcript cleavage factor/unknown domain fusion protein
transcript cleavage factor/unknown domain fusion protein
transcript cleavage factor/unknown domain fusion protein
transcript cleavage factor/unknown domain fusion protein
transcript cleavage factor/unknown domain fusion protein
transcript cleavage factor/unknown domain fusion protein
transcript cleavage factor/unknown domain fusion protein
flagellar filament sheath protein
flagellar filament sheath protein
flagellar filament sheath protein
flagellar filament sheath protein
flagellar filament sheath protein
flagellar filament sheath protein
hypothetical protein LA_3397
hypothetical protein LA_3397
hypothetical protein LA_3397
hypothetical protein LA_3397
hypothetical protein LA_3405
hypothetical protein LA_3405
hypothetical protein LA_3405
30S ribosomal protein S9
30S ribosomal protein S9
30S ribosomal protein S9
30S ribosomal protein S9
30S ribosomal protein S9
hypothetical protein LA_3433
hypothetical protein LA_3433
hypothetical protein LA_3433
riboflavin biosynthesis protein
riboflavin biosynthesis protein
riboflavin biosynthesis protein
FAD/FMN-containing dehydrogenase
FAD/FMN-containing dehydrogenase
FAD/FMN-containing dehydrogenase
short chain dehydrogenase
short chain dehydrogenase
short chain dehydrogenase
short chain dehydrogenase
short chain dehydrogenase
short chain dehydrogenase
short chain dehydrogenase
short chain dehydrogenase
hypothetical protein LA_3492

methyl-accepting chemotaxis transducer transmembrane protein
thioesterase-like protein
hypothetical protein LA_3534
hypothetical protein LA_3534
hypothetical protein LA_3534
hypothetical protein LA_3534
activator of Hsp90 ATPase 1 family protein
activator of Hsp90 ATPase 1 family protein
selenocysteine lyase
selenocysteine lyase
selenocysteine lyase
selenocysteine lyase
selenocysteine lyase
selenocysteine lyase
Fe-S cluster assembly ABC transporter permease
Fe-S cluster assembly ABC transporter permease
hypothetical protein LA_3584
DNA-binding ferritin-like protein
DNA-binding ferritin-like protein
DNA-binding ferritin-like protein
lysophospholipase
lysophospholipase
anthranilate synthase component II
anthranilate synthase component II
multidrug ABC transporter ATPase/permease
multidrug ABC transporter ATPase/permease
multidrug ABC transporter ATPase/permease
ketosteroid isomerase-like protein
ketosteroid isomerase-like protein
ketosteroid isomerase-like protein
hypothetical protein LA_3664
hypothetical protein LA_3664
hypothetical protein LA_3664
uracil-DNA glycosylase
uracil-DNA glycosylase
uracil-DNA glycosylase
hypothetical protein LA_3669
multidrug ABC transporter ATPase/permease
multidrug ABC transporter ATPase/permease
hypothetical protein LA_3697
hypothetical protein LA_3697
antimicrobial peptide ABC transporter ATPase
antimicrobial peptide ABC transporter ATPase
antimicrobial peptide ABC transporter ATPase
phosphoribosylamine--glycine ligase
phosphoribosylamine--glycine ligase
phosphoribosylamine--glycine ligase
phosphoribosylamine--glycine ligase

phosphoribosylamine--glycine ligase
phosphoribosylamine--glycine ligase
transcriptional regulator
transcriptional regulator
transcriptional regulator
transcriptional regulator
pirin-like protein
pirin-like protein
pirin-like protein
pirin-like protein
pirin-like protein
pirin-like protein
pirin-like protein
acriflavine resistance protein
acriflavine resistance protein
acriflavine resistance protein
hypothetical protein LA_3911
hypothetical protein LA_3911
hypothetical protein LA_3911
acyl-CoA dehydrogenase
acyl-CoA dehydrogenase
acyl-CoA dehydrogenase
acyl-CoA dehydrogenase
acyl-CoA dehydrogenase
phosphoesterase
phosphoesterase
phosphoesterase
hypothetical protein LA_3961
hypothetical protein LA_3961
hypothetical protein LA_3961
hypothetical protein LA_3961
hypothetical protein LA_3961
hypothetical protein LA_3961
hypothetical protein LA_3961
hypothetical protein LA_3961
hypothetical protein LA_3961
hypothetical protein LA_3961
hypothetical protein LA_3961
hypothetical protein LA_3961
hypothetical protein LA_3961
hypothetical protein LA_3961
hypothetical protein LA_3961
hypothetical protein LA_3961
hypothetical protein LA_3961
hypothetical protein LA_3961
hypothetical protein LA_3961
aldo/keto reductase
potassium/proton antiporter

potassium/proton antiporter
hypothetical protein LA_4000
hypothetical protein LA_4000
hypothetical protein LA_4000
hypothetical protein LA_4025
hypothetical protein LA_4025
hypothetical protein LA_4025
hypothetical protein LA_4025
hypothetical protein LA_4025
organic radical activating enzyme
lauroyl/myristoyl acyltransferase
lauroyl/myristoyl acyltransferase
lauroyl/myristoyl acyltransferase
DEAD/DEAH box helicase
DEAD/DEAH box helicase
DEAD/DEAH box helicase
DEAD/DEAH box helicase
DEAD/DEAH box helicase
DEAD/DEAH box helicase
DEAD/DEAH box helicase
DEAD/DEAH box helicase
DEAD/DEAH box helicase
ankyrin repeat-containing protein
ankyrin repeat-containing protein
apolipoprotein n-acyltransferase
apolipoprotein n-acyltransferase
apolipoprotein n-acyltransferase
apolipoprotein n-acyltransferase
apolipoprotein n-acyltransferase
apolipoprotein n-acyltransferase
hypothetical protein LA_4084
hypothetical protein LA_4084
amidophosphoribosyltransferase
amidophosphoribosyltransferase
amidophosphoribosyltransferase
amidophosphoribosyltransferase
amidophosphoribosyltransferase
ribonuclease D
ribonuclease D
ribonuclease D
ribonuclease D
ribonuclease D
ribonuclease D
bifunctional ornithine acetyltransferase/N-acetylglutamate synthase
bifunctional ornithine acetyltransferase/N-acetylglutamate synthase
bifunctional ornithine acetyltransferase/N-acetylglutamate synthase
bifunctional ornithine acetyltransferase/N-acetylglutamate synthase
bifunctional ornithine acetyltransferase/N-acetylglutamate synthase

bifunctional ornithine acetyltransferase/N-acetylglutamate synthase
two component system sensor histidine kinase
two component system sensor histidine kinase
thioesterase
thioesterase
hypothetical protein LA_4154
hypothetical protein LA_4154
hypothetical protein LA_4154
hypothetical protein LA_4154
hypothetical protein LA_4154
hypothetical protein LA_4154
hypothetical protein LA_4154
hypothetical protein LA_4154
hypothetical protein LA_4154
hypothetical protein LA_4154
hypothetical protein LA_4154
hypothetical protein LA_4154
hypothetical protein LA_4154
hypothetical protein LA_4154
hypothetical protein LA_4154
endoflagellar motor protein
hypothetical protein LA_4185
hypothetical protein LA_4185
hypothetical protein LA_4185
hypothetical protein LA_4185
hypothetical protein LA_4185
hypothetical protein LA_4185
hypothetical protein LA_4185
hypothetical protein LA_4185
hypothetical protein LA_4212
ferredoxin-like sulfite reductase
ferredoxin-like sulfite reductase
ferredoxin-like sulfite reductase
ferredoxin-like sulfite reductase
ferredoxin-like sulfite reductase
ferredoxin-like sulfite reductase
ferredoxin-like sulfite reductase
ferredoxin-like sulfite reductase
ferredoxin-like sulfite reductase
ferredoxin-like sulfite reductase
ferredoxin-like sulfite reductase
ferredoxin-like sulfite reductase
ferredoxin-like sulfite reductase
ferredoxin-like sulfite reductase
ketol-acid reductoisomerase
ketol-acid reductoisomerase
ketol-acid reductoisomerase
ketol-acid reductoisomerase

ketol-acid reductoisomerase
ketol-acid reductoisomerase
ketol-acid reductoisomerase
ketol-acid reductoisomerase
ketol-acid reductoisomerase
ketol-acid reductoisomerase
redox protein OsmC
redox protein OsmC
dihydroorotate dehydrogenase 2
hypothetical protein LA_4314a
hypothetical protein LA_4318
carboxypeptidase I
carboxypeptidase I
carboxypeptidase I
carboxypeptidase I
carboxypeptidase I
ferredoxin--NADP reductase
ferredoxin--NADP reductase
ferredoxin--NADP reductase
ferredoxin--NADP reductase
ferredoxin--NADP reductase
ferredoxin--NADP reductase
ferredoxin--NADP reductase
ferredoxin--NADP reductase
ferredoxin--NADP reductase
ferredoxin--NADP reductase
ferredoxin--NADP reductase
ferredoxin--NADP reductase
DNA polymerase III subunit gamma
DNA polymerase III subunit gamma
DNA polymerase III subunit gamma
DNA polymerase III subunit gamma
DNA polymerase III subunit gamma
DNA polymerase III subunit gamma
DNA polymerase III subunit gamma
DNA polymerase III subunit gamma
branched-chain amino acid aminotransferase
branched-chain amino acid aminotransferase
branched-chain amino acid aminotransferase
branched-chain amino acid aminotransferase
branched-chain amino acid aminotransferase
branched-chain amino acid aminotransferase
glucose-methanol-choline oxidoreductase
hypothetical protein LIF_A0038
hypothetical protein LIF_A0038
NAD(P)(+) transhydrogenase alpha subunit
NAD(P)(+) transhydrogenase alpha subunit
NAD(P)(+) transhydrogenase alpha subunit

OmpA family protein
hypothetical protein LIF_A0060
hypothetical protein LIF_A0060
hypothetical protein LIF_A0060
hypothetical protein LIF_A0060
enoyl-CoA hydratase
long-chain-fatty-acid CoA ligase
hypothetical protein LIF_A0106
hypothetical protein LIF_A0106
hypothetical protein LIF_A0115
hypothetical protein LIF_A0115
hypothetical protein LIF_A0117
hypothetical protein LIF_A0117
LipL45-like lipoprotein
putative lipoprotein
putative lipoprotein
putative lipoprotein
putative lipoprotein
hypothetical protein LIF_A0126
hypothetical protein LIF_A0126
hypothetical protein LIF_A0126
hypothetical protein LIF_A0126
transcriptional regulator
hypothetical protein LIF_A0139
NADH dehydrogenase (ubiquinone) chain G
NADH dehydrogenase (ubiquinone) chain G
hypothetical protein LIF_A0146
preprotein translocase subunit YidC
preprotein translocase subunit YidC
cytochrome c oxidase subunit II
cytochrome c oxidase subunit II
integral membrane protein with PIN domain
integral membrane protein with PIN domain
hypothetical protein LIF_A0229
hypothetical protein LIF_A0232
hypothetical protein LIF_A0232
lipoprotein releasing system, LolD ATPase component
lipoprotein releasing system, LolD ATPase component
cAMP-binding protein
Zn-dependent alcohol dehydrogenase
NAD(P)(+) transhydrogenase beta component
NAD(P)(+) transhydrogenase beta component
glycerophosphoryl diester phosphodiesterase
putative lipoprotein
hypothetical protein LIF_A0297
hypothetical protein LIF_A0297
hypothetical protein LIF_A0321
hypothetical protein LIF_A0331

ATP-dependent protease ClpA
electron transfer flavoprotein subunit beta
putative lipoprotein
putative lipoprotein
hypothetical protein LIF_A0374
hypothetical protein LIF_A0374
hypothetical protein LIF_A0378
riboflavin synthase subunit beta
hypothetical protein LIF_A0398
hypothetical protein LIF_A0398
hypothetical protein LIF_A0398
hypothetical protein LIF_A0398
hypothetical protein LIF_A0402
hypothetical protein LIF_A0402
hypothetical protein LIF_A0402
hypothetical protein LIF_A0402
putative lipoprotein
hypothetical protein LIF_A0449
cation/multidrug efflux pump
putative periplasmic solute-binding protein
putative periplasmic solute-binding protein
bifunctional permease/carbonic anhydrase
bifunctional permease/carbonic anhydrase
bifunctional permease/carbonic anhydrase
bifunctional permease/carbonic anhydrase
LipL41
LipL41
oxidoreductase-related protein
oxidoreductase-related protein
hypothetical protein LIF_A0534
methyl-accepting chemotaxis protein
methyl-accepting chemotaxis protein
methyl-accepting chemotaxis protein
methyl-accepting chemotaxis protein
methyl-accepting chemotaxis protein
methyl-accepting chemotaxis protein
elongation factor Tu
50S ribosomal protein L22
30S ribosomal protein S3
30S ribosomal protein S3
30S ribosomal protein S3
50S ribosomal protein L16
50S ribosomal protein L14
30S ribosomal protein S8
30S ribosomal protein S8
30S ribosomal protein S8
50S ribosomal protein L6
30S ribosomal protein S5

30S ribosomal protein S5
50S ribosomal protein L15
adenylate kinase
L,L-diaminopimelate aminotransferase
putative Zn-dependent hydrolase
hypothetical protein LIF_A0647
hypothetical protein LIF_A0647
trypsin-like serine protease
hypothetical protein LIF_A0671
dolichyl-phosphate-mannose-protein mannosyltransferase
aldehyde dehydrogenase
aldehyde dehydrogenase
hypothetical protein LIF_A0717
hypothetical protein LIF_A0717
NADH dehydrogenase (ubiquinone) chain J
transcription elongation factor NusA
translation initiation factor IF-2
translation initiation factor IF-2
30S ribosomal protein S15
hypothetical protein LIF_A0776
ATP-binding protein of an ABC transporter complex
membrane carboxypeptidase/penicillin-binding protein
membrane carboxypeptidase/penicillin-binding protein
lipid-a-disaccharide synthase
lipid-a-disaccharide synthase
lipid-a-disaccharide synthase
hypothetical protein LIF_A0898
hypothetical protein LIF_A0898
hypothetical protein LIF_A0924
hypothetical protein LIF_A0924
hypothetical protein LIF_A0924
hypothetical protein LIF_A0924
preprotein translocase subunit SecD
preprotein translocase subunit SecD
preprotein translocase subunit SecF
ABC transporter ATP-binding protein
ABC transporter ATP-binding protein
methyl-accepting chemotaxis protein
hypothetical protein LIF_A0978
hypothetical protein LIF_A0978
hypothetical protein LIF_A0978
hypothetical protein LIF_A0978
hypothetical protein LIF_A0978
membrane carboxypeptidase/penicillin-binding protein
membrane carboxypeptidase/penicillin-binding protein
dihydrolipoamide dehydrogenase
alpha-ketoglutarate decarboxylase
50S ribosomal protein L20

hypothetical protein LIF_A1010
hypothetical protein LIF_A1010
hypothetical protein LIF_A1010
chemotaxis protein histidine kinase CheA
chemotaxis protein histidine kinase CheA
chemotaxis protein histidine kinase CheA
chemotaxis protein histidine kinase CheA
30S ribosomal protein S1
hypothetical protein LIF_A1024
transcriptional regulator
phosphoglycerol transferase-related protein
phosphoglycerol transferase-related protein
glutamine synthetase
phosphoribosylformylglycinamidine (FGAM) synthase
phosphoribosylformylglycinamidine (FGAM) synthase
putative lipoprotein
hypothetical protein LIF_A1067
histidine kinase sensor protein
histidine kinase sensor protein
transcriptional regulator
GTP-binding protein BipA
putative lipoprotein
putative lipoprotein
export protein
hypothetical protein LIF_A1130
hypothetical protein LIF_A1130
hypothetical protein LIF_A1130
adenylate/guanylate cyclase
UDP-glucose 6-dehydrogenase
UDP-glucose 6-dehydrogenase
UDP-glucose 6-dehydrogenase
hypothetical protein LIF_A1202
hypothetical protein LIF_A1211
hypothetical protein LIF_A1211
carboxy-terminal processing protease
carboxy-terminal processing protease
chemotaxis protein CheW
chemotaxis protein CheW
heavy metal efflux pump
heavy metal efflux pump
heavy metal efflux pump
Hsp20/alpha crystallin molecular chaperone
ABC transporter ATP-binding protein
ABC transporter ATP-binding protein
N-acetylneuraminic (sialic) acid synthetase
N-acetylneuraminic (sialic) acid synthetase
glucose-1-phosphate thymidylyltransferase
50S ribosomal protein L9

50S ribosomal protein L9
signal peptidase I
HD family protein
hypothetical protein LIF_A1397
hypothetical protein LIF_A1401
hypothetical protein LIF_A1401
antisigma factor antagonist-related protein
antisigma factor antagonist-related protein
antisigma factor antagonist-related protein
ATP-dependent protease ClpA
putative lipoprotein
putative lipoprotein
putative lipoprotein
putative lipoprotein
putative lipoprotein
putative lipoprotein
putative lipoprotein
putative lipoprotein
succinate dehydrogenase flavoprotein subunit
succinate dehydrogenase flavoprotein subunit
succinate dehydrogenase flavoprotein subunit
succinate dehydrogenase flavoprotein subunit
aldo/keto oxidoreductase
hypothetical protein LIF_A1547
CAP family transcription factor
acyl-CoA dehydrogenase
hydrolase
hypothetical protein LIF_A1568
ATP-dependent protease ClpP
preprotein translocase subunit SecA
preprotein translocase subunit SecA
cation efflux system protein
short-chain dehydrogenase
short-chain dehydrogenase
short-chain dehydrogenase
FAD-binding oxidoreductase
FAD-binding oxidoreductase
hypothetical protein LIF_A1600
hypothetical protein LIF_A1604
glycosyltransferase
hypothetical protein LIF_A1636
hypothetical protein LIF_A1637
putative lipoprotein
putative hydrolase
putative hydrolase
undecaprenyldiphospho-muramoylpentapeptide beta- N-acetylglucosaminyltransferase
hypothetical protein LIF_A1659
hypothetical protein LIF_A1672

HflC membrane associated protease
HflC membrane associated protease
peptidyl-prolyl cis-trans isomerase
hypothetical protein LIF_A1682
flagellar motor switch protein FliM
flagellar motor switch protein FliM
gamma-glutamyltranspeptidase
glycerol kinase
oxidoreductase
oxidoreductase
oxidoreductase
SGNH-hydrolase
DNA mismatch repair protein mutS
isocitrate/isopropylmalate dehydrogenase
putative esterase of the alpha-beta hydrolase
putative esterase of the alpha-beta hydrolase
putative esterase of the alpha-beta hydrolase
putative esterase of the alpha-beta hydrolase
long-chain-fatty-acid CoA ligase
long-chain-fatty-acid CoA ligase
long-chain-fatty-acid CoA ligase
long-chain-fatty-acid CoA ligase
serine protease
serine protease
serine protease
excinuclease ABC subunit A
endoflagellar motor protein B
magnesium transporter
sensor histidine kinase and response regulator of a two component complex
sensor histidine kinase and response regulator of a two component complex
sensor histidine kinase and response regulator of a two component complex
sensor histidine kinase and response regulator of a two component complex
RNA polymerase sigma-70 factor
RNA polymerase sigma-70 factor
RNA polymerase sigma-70 factor
glycerol-3-phosphate dehydrogenase
glycerol-3-phosphate dehydrogenase
glycerol-3-phosphate dehydrogenase
glycerol-3-phosphate dehydrogenase
hypothetical protein LIF_A1828
methyl-accepting chemotaxis protein
hypothetical protein LIF_A1837
alkylglycerone-phosphate synthase
alkylglycerone-phosphate synthase
alkylglycerone-phosphate synthase
alkylglycerone-phosphate synthase
PurH
LipL45

LipL45
LipL45
hypothetical protein LIF_A1878
long-chain-fatty-acid CoA ligase
long-chain-fatty-acid CoA ligase
long-chain-fatty-acid CoA ligase
transcriptional regulator
3-polyprenyl-4-hydroxybenzoate decarboxylase
3-polyprenyl-4-hydroxybenzoate decarboxylase
glucose/sorbosone dehydrogenase
glucose/sorbosone dehydrogenase
actin-like ATPase
actin-like ATPase
type II secretory pathway component protein G
type II secretory pathway component protein G
type II secretory pathway component protein D
type II secretory pathway component protein D
50S ribosomal protein L19
hypothetical protein LIF_A1958
endoflagellar filament core protein
heavy metal efflux pump
heavy metal efflux pump
heavy metal efflux pump
heavy metal efflux pump
hypothetical protein LIF_A2014
hypothetical protein LIF_A2016
SMC-like protein
SMC-like protein
hypothetical protein LIF_A2026
hypothetical protein LIF_A2026
hypothetical protein LIF_A2026
putative lipoprotein
amidase
hypothetical protein LIF_A2057
hypothetical protein LIF_A2057
hypothetical protein LIF_A2070
hypothetical protein LIF_A2070
adenylate/guanylate cyclase
adenylate/guanylate cyclase
LipL45-related protein
LipL45-related protein
glycosyl hydrolase
hypothetical protein LIF_A2106
hypothetical protein LIF_A2106
hypothetical protein LIF_A2106
hypothetical protein LIF_A2106
hypothetical protein LIF_A2106
hypothetical protein LIF_A2113

flagellar MS-ring protein
flagellar MS-ring protein
short-chain dehydrogenase
LipL32
LipL32
LipL32
LipL32
adenylate/guanylate cyclase
adenylate/guanylate cyclase
chaperonin GroEL
flagellar basal body P-ring protein
flagellar basal body P-ring protein
Fe-S oxidoreductase
Fe-S oxidoreductase
Fe-S oxidoreductase
Fe-S oxidoreductase
acyltransferase
acyltransferase
putative lipoprotein
protein MreC
protein MreC
actin-like ATPase
actin-like ATPase
actin-like ATPase
actin-like ATPase
cGMP-specific phosphodiesterase
F0F1 ATP synthase subunit beta
ATP synthase F1 subunit delta
ATP synthase F1 subunit delta
methyl-accepting chemotaxis protein
hypothetical protein LIF_A2300
alpha-galactosidase
adenylate/guanylate cyclase
prolipoprotein diacylglyceryl transferase
hypothetical protein LIF_A2478
glycosyltransferase
OppA
OppA
OppA
guanosine polyphosphate pyrophosphohydrolase/synthetase
guanosine polyphosphate pyrophosphohydrolase/synthetase
anti-sigma factor antagonist
LipL71
LipL71
LipL71
LipL71
putative lipoprotein
alpha/beta hydrolase superfamily protein

alpha/beta hydrolase superfamily protein
alpha/beta hydrolase superfamily protein
metallophosphoesterase
metallophosphoesterase
metallophosphoesterase
putative lipoprotein
putative lipoprotein
putative lipoprotein
putative lipoprotein
hypothetical protein LIF_A2617
Fe-S-cluster-containing hydrogenase
molybdopterin oxidoreductase iron-sulfur binding subunit
molybdopterin oxidoreductase iron-sulfur binding subunit
hypothetical protein LIF_A2620
adenylate cyclase-like protein
adenylate cyclase-like protein
adenylate cyclase-like protein
tryptophan synthase subunit beta
zinc metalloprotease
zinc metalloprotease
zinc metalloprotease
hypothetical protein LIF_A2650
glutathione transferase
glutathione transferase
glutathione transferase
surface antigen OrfC lipoprotein
surface antigen OrfC lipoprotein
transcript cleavage factor/hypothetical protein
transcript cleavage factor/hypothetical protein
transcript cleavage factor/hypothetical protein
transcript cleavage factor/hypothetical protein
transcript cleavage factor/hypothetical protein
transcript cleavage factor/hypothetical protein
50S ribosomal protein L13
30S ribosomal protein S7
30S ribosomal protein S12
50S ribosomal protein L7/L12
50S ribosomal protein L1
DEAD/DEAH box helicase
outer membrane receptor for Fe3+-dicitrate/TonB-dependent receptor
hypothetical protein LIF_A2802
hypothetical protein LIF_A2804
hypothetical protein LIF_A2804
hypothetical protein LIF_A2804
hypothetical protein LIF_A2828
hypothetical protein LIF_A2858
hypothetical protein LIF_A2858
hypothetical protein LIF_A2858

hypothetical protein LIF_A2871
ATP-dependent Lon protease
ATP-dependent Lon protease
MaoC-like acyl dehydratase
2-dehydropantoate 2-reductase
2-dehydropantoate 2-reductase
2-dehydropantoate 2-reductase
ATPase and permease components of ABC-type multidrug transport system
homoserine dehydrogenase
anti-Sigma factor antagonist
enoyl-CoA hydratase
hydrolase
hydrolase
membrane carboxypeptidase/penicillin-binding protein
ATPase and permease components of ABC-type multidrug transport system
ATPase and permease components of ABC-type multidrug transport system
ATPase and permease components of ABC-type multidrug transport system
DnaK protein
DnaK protein
heat shock protein DnaJ
heat shock protein DnaJ
oxidoreductase
ATPase component of ABC-type antimicrobial peptide transport system
hypothetical protein LIF_A3008
putative hydrolase
glycosyltransferase
30S ribosomal protein S20
hypothetical protein LIF_A3053
ATP-dependent Zn protease
ATP-dependent Zn protease
ATP-dependent Zn protease
50S ribosomal protein L25
transcriptional regulator
cation/multidrug efflux pump
cation/multidrug efflux pump
hypothetical protein LIF_A3092
hypothetical protein LIF_A3092
hypothetical protein LIF_A3092
serine protease
serine protease
NAD(P)H steroid dehydrogenase
NAD(P)H steroid dehydrogenase
hypothetical protein LIF_A3166
hypothetical protein LIF_A3166
hypothetical protein LIF_A3182
adenylate/guanylate cyclase
adenylate/guanylate cyclase
adenylate/guanylate cyclase

| |
|---|
| hypothetical protein LIF_A3213 |
| apolipoprotein n-acyltransferase |
| apolipoprotein n-acyltransferase |
| apolipoprotein n-acyltransferase |
| apolipoprotein n-acyltransferase |
| putative lipoprotein |
| Zn-dependent alcohol dehydrogenase |
| Zn-dependent alcohol dehydrogenase |
| serine phosphatase RsbU, regulator of sigma subunit |
| acetyl-CoA acetyltransferase |
| hypothetical protein LIF_A3311 |
| hypothetical protein LIF_A3311 |
| hypothetical protein LIF_A3311 |
| ATP-binding protein of an ABC transporter complex |
| ATP-binding protein of an ABC transporter complex |
| GMC oxidoreductase |
| GMC oxidoreductase |
| hypothetical protein LIF_A3337 |
| DNA gyrase subunit A |
| DNA gyrase subunit B |
| DNA gyrase subunit B |
| DNA gyrase subunit B |
| polysaccharide deacetylase |
| methyl-accepting chemotaxis protein |
| acetyl-CoA synthetase |
| acetyl-CoA synthetase |
| hypothetical protein LIF_A3408 |
| sodium/hydrogen antiporter |
| metallopeptidase |
| Total Conserved Peptides |

| Sequence |
| --- |
| (R)IIEAVAK(E) |
| (R)LLLGALNDIYLYK(K) |
| (K)AIHAVEGVITTR(E) |
| (K)ETFLSATDLEISIK(T) |
| (K)FSTSISKEEFQVSLR(Q) |
| (K)IKVNTSEFLK(A) |
| (K)LIEGNFPNYEQVIPK(N) |
| (K)SILSNLK(I) |
| (R)FIFNGLYMIPNGDK(L) |
| (R)FIFNGLYMIPNGDKLIFVGTDGR(R) |
| (R)KTSYAIAHEDQR(F) |
| (R)TLPSPLQFK(D) |
| (K)FITVLLTPMDLK(I) |
| (K)EGLTAVISIK(I) |
| (K)GIMQTLSSEGLTLFFEENPNITK(K) |
| (K)GIMQTLSSEGLTLFFEENPNITKK(I) |
| (K)GLVLVVEDHRR(G) |
| (K)ILSSEEIR(V) |
| (K)IMIMTDADIDGSHIR(T) |
| (K)IPQPQFNSQTK(E) |
| (K)ISTIEVVMTILHAGGK(F) |
| (K)KHPTGLSGEDVK(E) |
| (K)TVLEGGGLPGK(L) |
| (K)VIHFER(N) |
| (R)FRELAFLNK(G) |
| (R)GYLYVAQPPLYLIK(H) |
| (R)NEFQFSGGIVSFVEHINENK(H) |
| (R)NTQAILPLK(G) |
| (R)TLLLTFFFR(Y) |
| (R)TLNDFLK(K) |
| (R)VLVSALGTGIGEDEFNIDKIR(Y) |
| (K)AAMTHDNIMFFSNIGK(V) |
| (K)EILTAYAAHR(R) |
| (K)IVGEVIGNYHPHGDASVYEALVR(M) |
| (K)KDANAQVILNQLYK(M) |
| (K)LIIDLK(D) |
| (K)SGIIAIGLR(E) |
| (K)VESMTDKDEVMIFSK(K) |
| (K)VLSYNIAGKPDIADALK(N) |
| (R)AHILEGLK(I) |
| (R)ETIDAVIAVIR(N) |
| (R)EVIVVTEIPYQVNKK(V) |
| (R)ILHAMNER(A) |
| (R)MVQEFSLR(V) |
| (K)ILQVATESLK(K) |
| (K)ILQVATESLKK(A) |

(K)KEDQVSLVYGMEEVTGLENVEAEVTTK(K)
(K)KQEAQILGNR(Y)
(K)LNNYYDVWIK(T)
(K)TISTSLSKDPLLGETVK(L)
(K)TISTSLSKDPLLGETVKLLK(K)
(R)YANFDQWIYK(V)
MVGETVESASGVSDGEATASVIVSQSQGVVK(G)
(R)LQGASDVVKK(M)
(K)SGLEFFKPISTGK(G)
(-)MNDLFNVK(N)
(K)KGGNIINIASILGMR(G)
(K)NAGAIVYGTGSSQESIK(K)
(K)NAGAIVYGTGSSQESIKK(F)
(K)TVLVTGSTR(G)
(R)ALAVEWIGSGYR(V)
(R)LDVLVNNAGIASNKPAAFLK(E)
(R)YFAEGFK(N)
(-)MILSLHSINPEK(R)
(K)ITPGPFTFITSANK(H)
(K)IYIFPTDTVYALLADSQSK(L)
(K)ATQQLVPR(V)
(K)VVIASAK(G)
(K)VVIASAKGETTENPAPR(A)
(R)GLLASR(T)
(R)TNTLLSIPmLFLmGAAR(N)
(R)TNTLLSIPmLFLmGAAR(N)
(R)VLWWFR(W)
(R)IVEGTYPPGYIPK(R)
(-)MEHPLAPPIDFPLEEVKR(R)
(K)FIVEYLR(Q)
(K)HFLSGNSEVVVSNGK(V)
(K)SVQSVSGIFIESALK(L)
(K)TFSFSTEIEER(D)
(R)AMLVPSFPTNR(K)
(R)AMLVPSFPTNRK(E)
(R)DGIQRTIGTRR(Q)
(-)MHNQDSLIYGILPDVHFR(Y)
(R)TMIGAFFLADQIKEDTK(V)
(K)IESLNAFLQLNATK(I)
(K)NIIINSNQK(L)
(K)VVTDSEHNVIEIAVQDR(L)
(K)VVVVSAGAIEGPALLQR(S)
(R)LGVHPVPQNLINANNNILMK(G)
(K)ALEFGER(T)
(K)AVSLSLPK(K)
(K)FQQAETLLEGLVK(D)
(K)MLYYANLNQAVAYHK(M)
(K)SYTAHNTVQPLIGLTK(L)

(K)FAVFPNSIHELTK(F)
(K)AILAMNEIK(E)
(K)NLPITSQEITIPINSVLISR(T)
(K)ETYWGNVNPIGIR(S)
(K)FEFIR(H)
(K)IVHKPLPQDDPAR(R)
(K)TNVLGMMNMLGLAK(R)
(K)VSLVEGIR(K)
(K)VSLVEGIRK(T)
(R)IFNTYGPR(M)
(R)ILQASTSEVYGNPLEHPQK(E)
(R)RKPDLTLAK(Q)
(R)VVSNFIVQALK(K)
(R)VVSNFIVQALKK(E)
(K)ADVIITTALIPGK(K)
(K)AGASLVSR(Q)
(K)AVLLAASHLAR(F)
(K)EQVHSLGAK(F)
(K)FVEVEGASHSAAAGGYAVEQTEEYK(K)
(K)FVEVEGASHSAAAGGYAVEQTEEYKK(R)
(K)HGQNVVTPK(G)
(K)IGATVLVEK(G)
(K)IGATVLVEKGAGEQSYYHDEDYKK(A)
(K)KAPLLITK(K)
(K)KVEVLSLDAIAR(I)
(K)MKPGTIYLGMFQPAmNAQTVQK(L)
(K)MKPGTIYLGMFQPAmNAQTVQK(L)
(K)NLAGSIPADASKMFSK(N)
(K)NVLNFLK(L)
(K)SNLIVSIHFADPTTLGK(M)
(R)HKLTLDALGSK(S)
(R)LGAVVDVFDTRPEVK(E)
(R)LGAVVDVFDTRPEVKEQVHSLGAK(F)
(R)VSVTPDIVDALKK(I)
(K)LLDSFLK(N)
(K)TIFLPIMDESLFVDTFK(L)
(R)VAIRPEmTPTLAR(M)
(R)WFSIPNLWR(Y)
(R)YVLFLGESEIR(T)
(K)IEISSPGFHPQEDILDLR(G)
(K)IEQPIENQKPNSAALK(V)
(K)LLDPLVGYLLK(N)
(K)EIIPMDSNSNFVSQKPAR(R)
(K)LTAAHPTLPLGSIIR(V)
(K)ILGNITIGK(N)
(K)VFSILLER(I)
(R)FITGIDIHPGAK(I)
(K)IILDESPEYFAGHK(D)

(K)SMQGAGGALVMGVVMNSIMAR(S)
(K)SSLVNPLNNLLSGVEK(V)
(K)YFSYFKPAETR(H)
(-)MILTGK(E)
(K)LVIPESGLLLKPGILYLGR(T)
(R)GIQTSMLYK(D)
(R)LHEELLVYTELPLDMK(K)
(R)TLESTETHNLVPMLEGR(S)
(K)ALITESK(Y)
(K)TIDAQTALDFGIANSIWHDSSLPAAK(M)
(K)YYNNTLNTEDRLEALK(A)
(R)IGISK(A)
(R)LGIIPGGGGTQR(L)
(K)SFLYSILSSEEVGK(E)
(K)NVFSGIVQNSGIK(K)
(K)TMNLNNTLFAVLK(K)
(R)IIDQKPNWEAIGYEISEK(A)
(K)IETGAGILEVDLK(I)
(K)RVNVEFVTIR(G)
(K)VDLVSVDMGKPVLVPSQIPVVWK(N)
(K)YIYDHGLTSSK(N)
(R)GIGPLIER(H)
(R)GKDHLYQR(T)
(R)HSIFPK(R)
(R)IQWQESGNILMTGPAR(E)
(R)LTPEQIQK(I)
(R)NFGIGSDGVIFIR(N)
(R)VNVEFVTIR(G)
(K)RRDPSQPQELGR(D)
(R)ANHNFHELSEVYQR(F)
(K)LSNVTLVDFFPR(T)
(K)ALSTLTALAEK(N)
(K)AESKKEEAAPASESSTPAGEEQ(-)
(K)EEAAPASESSTPAGEEQ(-)
(K)KEEAAPASESSTPAGEEQ(-)
(R)SIFDLNK(L)
(K)IIFR(N)
(K)GILQEIFR(E)
(K)INTPQFIISSK(K)
(K)KGILQEIFR(E)
(K)KINTPQFIISSK(K)
(K)VFWTGAIDSFQNFQK(V)
(R)FVAHSYIAR(I)
(R)LGWLYVDNIR(I)
(R)LPNIHSGVLSIELSTSLEK(F)
(R)TYNFPQGR(C)
(K)FLSLLTDLR(G)
(K)SIFGLNTEQKPNPSSTYLR(I)

(R)EPLILNLFL(-)
(R)NAGNVVVIQPEWR(L)
(R)TPTATWK(K)
(R)VIVLGENPSETDLKEWATK(S)
(R)YGSSTPGVNSR(E)
(K)VQTYEPSPLFTEYMLDAVNLYLK(E)
(K)HGLIDLNLWLR(G)
(K)LIGFYDPVRMKAER(K)
(R)GTGQYQFDTEIPFFEHMLSHISK(H)
(K)VPHIGWNR(L)
(K)AGIEHIVFTDIAQDGTLAGPNLEAYR(E)
(K)ISLYGVITGK(A)
(R)EILNSYPFQVIASGGIASLK(D)
(R)FILGTAAVTNPDLLK(Y)
(K)INSTPTLFINSKPLPK(S)
(K)IRDQINR(E)
(K)SFLSEYNGIIK(V)
(K)SGTPNVDFLHQLIR(Q)
(K)VSESAYSAIR(N)
(-)MDNENIKPVTLTIAGSDSGGGAGIQADLK(T)
(K)FKVPTLLK(G)
(K)QILAVLDYFPVK(A)
(K)TGMLFSTSIIETTSSILSK(R)
(K)MTFLVGNSGVGK(S)
(K)STLINLLTQTQVQK(T)
(K)ELLEQIQNILAIK(I)
(K)LNKELLEQIQNILAIK(I)
(K)SYDIFQALLTDALK(L)
(R)YAFTAGILMDVPR(I)
(K)AIPLYQEAFQK(T)
(K)ANIAINTYPTAPGYYLAGVSEYK(L)
(K)GISMLENPPPGTR(N)
(K)KVNLSGENVLVEIVSGK(K)
(K)KVNLSGENVLVEIVSGKK(S)
(K)KYEEAEVYFEEAR(K)
(K)LIGVPVIINNSVILNYGTSVESLDKDR(G)
(K)NPEFSDAHYQISYIYYK(R)
(K)NQTLANQYTK(E)
(K)SGVLFSIAQK(D)
(K)VNLSGENVLVEIVSGK(K)
(R)EAINAFLLR(K)
(R)ILSGEYEAALK(A)
(R)NEGNLFALIR(I)
(R)YLGSYETGIFFLSGK(K)
(K)IFYQESK(V)
(K)MEIVLGR(E)
(K)TDMLIIDAQYSSEEAEKK(V)
(K)TFHLLEK(N)

(K)VTPFLLR(H)
(R)GSLPTPLSESEYR(E)
(R)LVLTHHEPDHKDEDIFK(I)
(K)NIHELYPMLETVLVSNK(S)
(K)EMIASAIHYNSSR(A)
(K)GVYLPGEGVTGK(V)
(K)LFEMIHLVSDSR(A)
(K)NNPENVSFLAAPIK(S)
(R)ASVLITGESGTGK(E)
(R)ERSDDIPLLINHFIAK(Y)
(R)LNVVNMVTPPLR(E)
(R)SDDIPLLINHFIAK(Y)
(K)IEGLAVNLAIK(T)
(K)IVLDQVSVLLGNDVEIHPLKEEK(I)
(K)LFYGYHVSDR(A)
(K)TTLNTQNFYK(Y)
(R)EVHFVDSADGGYALAAAQLK(E)
(R)TGIFYFLR(R)
(-)MLLVPTYIADSPIGGFGLFAGR(D)
(K)FDFTEEIIQHFK(I)
(K)INSVIPVDFYNR(N)
(K)KLDQMNLMMSSYLADVGYTQMK(I)
(K)LILNNSFFAYNEK(T)
(K)LVNAELTVDLAK(N)
(K)NHPIISYLMIANLPDLDDNIK(T)
(K)TVLVADIQK(Q)
(R)IGTIKPNFSNMGK(L)
(K)TIGSPIALIVDNQNTISK(D)
(K)TIGSPIALIVDNQNTISKDYENLR(T)
(K)VSTFGESHGTSVGVVVEGVPAGIPIR(L)
(R)AAFKPTSTIFK(K)
(R)AINPQWFQK(W)
(R)RPGQSNLTTPR(D)
(R)TNHSGGLQGGISNGEELVIR(A)
(R)TTFRPSHADYTYQMK(Y)
(K)DFLMTTDFRPNTR(G)
(K)DVGVDIPFFCYHPK(L)
(K)GLLNSGSVFR(R)
(K)NGLNQSFQK(V)
(K)SVIVEAR(V)
(K)TILQSVESFEK(S)
(R)NVPQEEVGSNLIINHNR(C)
(K)AVTIEFPEK(K)
(K)ERmmQLVGGPIIGER(Q)
(K)FYFYSIGK(G)
(K)GLWITLK(H)
(K)KFEIDLLR(C)
(R)MMQLVGGPIIGER(Q)

(K)GIPESSLNK(I)
(K)MNFPILFQSR(K)
(R)ELLQFVLK(N)
(R)MLPDSEVSVTGDKK(Y)
(K)KMQELSPQLK(T)
(K)LVFYPLNQK(Q)
(K)SFIVTDSYIVEFSSLGGK(I)
(K)SFNQGITTPFR(N)
(K)SYQAVELSR(D)
(K)TDKESGVDFAGTGSR(Y)
(R)NGIIWILK(Q)
(R)TFGSLGPYPK(D)
(R)YFIAVADPLDHKPHGIVLDNRPK(N)
(K)GGFFGISQK(K)
(K)IIHGVVLTVLK(K)
(K)LLEPMNPFER(R)
(K)SVAATVAK(T)
(R)ELSLVR(L)
(R)IIHMALQENEK(V)
(R)KIVLDIESYR(D)
(K)AIAEWEEDTEKER(I)
(K)GIQINLSDSK(F)
(K)GVENYFLFNQSPFALTK(I)
(K)KPEFSALADTLR(R)
(K)VLMLQYNPGAFK(D)
(R)ELQIANVIIRPASLK(A)
(R)GIYEFLK(E)
(R)SYEVLLQTVR(T)
(K)AQSSNDTFPTAMHIAAAEQLNQK(L)
(K)AVLPSVYR(L)
(K)ESGIELGLLTSEQFDQWVLPEK(M)
(K)FEALAAHDSLVETSGVLK(T)
(K)KKTDEFQNIIK(I)
(K)KPIHPNDDVNK(A)
(K)LIPALIQLK(E)
(K)LNEHLNNSLmLVTALNPHIGYDNAAK(I)
(K)LNEHLNNSLmLVTALNPHIGYDNAAK(I)
(K)SAAVVNAELGLLSEDK(K)
(R)AIEIAGGVK(G)
(R)GIIPNKEK(L)
(R)LALGGTAVGTGLNTHPQFAVK(A)
(K)AIEWETSFATPK(-)
(K)GGVNASGGYSAVK(S)
(K)IAATPVNVSIGYVK(D)
(R)NYMDLLHDMGLMVLK(Q)
(R)LTLLAAPYGGLIEK(L)
(R)GISGFPYIFVRR(V)
(R)HKFLETQYDSPSNLPSDGTNLWR(D)

(R)VDLIDTLIAYDEEGKR(I)
(K)ADVTPGDFSEWSKTNAPVIK(E)
(K)GAGSSEPVSGLDAKDAK(N)
(K)GNIFYSELR(A)
(K)KADVTPGDFSEWSK(T)
(K)KADVTPGDFSEWSKTNAPVIK(E)
(K)KEESAAPEPSTQEQSAAANR(N)
(K)KGNIFYSELR(A)
(K)LPDSYALEITGHTDAIGPEQAEGAK(K)
(K)LPDSYALEITGHTDAIGPEQAEGAKK(G)
(K)QGIPANR(I)
(K)TNAPVIK(E)
(R)IVTKGAGSSEPVSGLDAK(D)
(R)NVDVNSPEAIADSLNEKLK(D)
(R)VTFR(F)
(R)YPDGLTRPGFSYK(K)
(R)HFGLNLFEEIRK(S)
(R)HIYLDLSNTKEIEFKLSNILK(E)
(K)IRYEHFLNFVPEVMK(F)
(K)WIIDPLDGTINYSHR(L)
(R)YEHFLNFVPEVMK(F)
(K)SILLSSFFK(R)
(K)GIWSWLTTIDHK(R)
(K)TLEWTIPSPPTHENFEK(T)
(K)TPVITGGPYEYR(-)
(K)LIGILLESVGLMER(N)
(K)SMGKPGLPPEDQDQYR(S)
(K)EPGLGIFGGGGSGNGFGVGIEKEEVR(D)
(K)VLNLNTLANALKPVVLPGEELGIQVIK(E)
(K)VTVTSIIQTAAGK(M)
(R)EIQLISDSSDPIKR(A)
(R)EIQLISDSSDPIKRAR(G)
(R)GLEMLNK(L)
(R)ILDIADTHFLDGPLILPNFVLR(E)
(K)DKYFVDEPSSNGNIWWSHINFK(V)
(K)ELGLEPSEIFHNLSYDEIYEHEK(K)
(K)ELGLEPSEIFHNLSYDEIYEHEKK(N)
(K)GGHPKTIIFLTYDAFGVLPPVSK(L)
(K)GIFSVmNYK(L)
(K)GIFSVmNYK(L)
(K)IIDEILNGNIEK(S)
(K)KGIFSVMNYK(L)
(K)KLGEMFIK(N)
(K)KLYVFDGYAGANPETR(V)
(K)LGEMFIK(N)
(K)LSIEQAMYHFLSGYTAK(V)
(K)RMNLPSTR(K)
(K)SEFVTHPVFQVAYPK(T)

(K)TEPDIYEAIRK(D)
(K)TIIFLTYDAFGVLPPVSK(L)
(K)VINLDPK(T)
(K)YFVDEPSSNGNIWWSHINFK(V)
(R)AYLmNTGLVGGSYGVGK(R)
(R)AYLmNTGLVGGSYGVGK(R)
(R)AYLMNTGLVGGSYGVGKR(M)
(R)KIIDEILNGNIEK(S)
(R)MNLPSTR(K)
(R)VSYPIFHINNIQVPSK(G)
(R)KPEVMMNGDEGNEEGLFER(F)
(K)FSLDGIEAILEAHK(N)
(R)TTLSQTLEPMIEPYLK(L)
(K)GKADPELVNQMLLEK(L)
(K)LIHSADPNIPQSYVDLNR(A)
(K)TSNWVKDEILGIVNK(E)
(K)TVFEELLTSDKDAETIVTEK(N)
(R)ALGAIVGYVMK(V)
(R)SKLPELPNER(K)
(K)FTYQLVQETPETAFLSVK(I)
(K)YTVEFQGK(E)
(R)FILSNQFNNEGIANFSGLR(E)
(R)IFESLVESLQINTR(V)
(K)TLETEILHELK(I)
(K)YLHLNIDPK(L)
(K)AWFYTYLNR(S)
(R)EHAAQWGIER(G)
(K)IMIQLLK(S)
(K)KPVETLEQFIPSGAR(V)
(K)LETPEGQIRPLPGTVVASK(N)
(K)SDDFMEQLIHYFNGK(D)
(K)ILDHLDLDVEEGEIVSVEGK(S)
(R)FSMVIVTHDMELASLVHK(R)
(R)NLMALILELQK(E)
(K)GLPIESLR(V)
(K)LEAALLSFR(V)
(R)QTSFLLLGDR(L)
(K)SQSIEVVGNYLYK(G)
(K)ANFEGMVK(A)
(K)KDSPSFFIIR(E)
(K)MVGLSYPKDENLVLDLLTK(N)
(K)VLYNFEIGTK(D)
(K)YLQYLPNGPFATQAK(L)
(R)IWTAYK(Q)
(R)LITLLSER(I)
(R)MYADNEMIFCEHEPGR(E)
(R)SASAIAWGHVQLLAINK(A)
(K)VIQYVEQHLIPFLDQT(-)

(R)DGLQQLGRPILGGGLR(F)
(R)FVLPPLNNQDPEDHGIEIK(I)
(R)NIIHVGLSDLFLPITVR(S)
(R)AAAGFNETHADSLIQLLSK(I)
(K)IIPSIK(N)
(K)GFWTLPAGFLENR(E)
(K)IIVGSIPIWENR(I)
(R)KGFWTLPAGFLENR(E)
(K)IVIHNIADFQR(I)
(K)VADVFLMLYEK(M)
(R)LLILLMDDIQGK(V)
(R)SATAIAISEVK(V)
(K)VALLPPEKDSISQK(S)
(K)YLQHYSGTTYAGR(A)
(R)NPAFELMNVAEVFYK(N)
(R)NSPYPLNMPPLVFEGSTSR(V)
(K)LLESLVVSLGYK(K)
(R)LNLNGLYVYPGLINGHDHLLSTYLPR(V)
(R)VAPSKPYLNWLPWDNDLK(S)
(K)AEFSMEFSR(Y)
(K)ANPQILEPImK(V)
(K)ANPQILEPImK(V)
(K)AYYFEGK(D)
(K)EIPDDLKELAQK(K)
(K)GIVDLVTMK(A)
(K)GMGELHLEVYIER(M)
(K)GMTIYNMSNNK(K)
(K)GMTIYNMSNNKK(H)
(K)HLNNLAK(A)
(K)HNAVPVQIPIGLENDLK(G)
(K)LKHNAVPVQIPIGLENDLK(G)
(K)LLDGVLDYLASPVDVK(N)
(K)MTPVFmGSAFK(N)
(K)MTPVFmGSAFK(N)
(K)NHTINIIDTPGHVDFTVEVER(S)
(K)REYGVELITGAPQVAYR(E)
(K)VSMESMFVPAPVISLTIEAK(E)
(R)FTKEDPTFQTHVDQESGQTIIK(G)
(R)GITIQSAATYCQWK(N)
(R)GSLIGFPIIGVR(C)
(R)IHAIHEVR(G)
(R)ILFYTNR(I)
(R)NIGISAHIDSGK(T)
(R)NVAEELMK(K)
(R)RYNVPR(V)
(R)TGTIELK(M)
(R)VAFINK(L)
(R)VAFINKLDR(T)

(R)VAGYMEPIPLEETLDYDFVNK(V)
(R)YAPVPR(N)
(K)VIPANVSFLK(E)
(K)VNSYPSFVILDR(S)
(K)LNISPPLIGK(T)
(K)LNISPPLIGKTFEFDNLNEALK(Y)
(K)YFQSGTSMGK(V)
(R)FGGYSSHLNIDSR(Y)
(R)MIVYGAAEMMGEGSSVNWAR(I)
(R)DSYGVPHIR(S)
(R)STTGGAILANDPHIGYGNPGTWYEVHLR(T)
(K)TGWLIDYAAVSK(I)
(R)FDAAHLLPNVPDGHK(C)
(K)ATQEIVTALK(E)
(K)STSAEEVAMLLDMAQR(V)
(K)TVIVIK(R)
(R)GIDVEFAIHPVAGR(M)
(R)MPGHMNVLLAEADIPYDK(M)
(R)SLPNVLFGGLGAAVDNSK(G)
(R)SLPNVLFGGLGAAVDNSKGGEDIYAGK(T)
(R)VVIVPGYGMAVAQAQHAVR(D)
(R)VVIVPGYGMAVAQAQHAVR(D)
(K)MVFDPNSVDAHQVMIASEK(A)
(K)KTNAEEYFEKN(-)
(R)NHLQIFPQGLAK(Y)
(K)IQITALNTETVNLIR(A)
(K)IVEEEDKIQYIR(S)
(K)KLDENSVEYHYLK(G)
(K)VFHMFPSR(S)
(-)MNSTLQNQTKTNLEK(V)
(K)DLAALmITYPSTHGVFEESVK(E)
(K)DLAALmITYPSTHGVFEESVK(E)
(K)FTSIFANVLK(N)
(K)NSPHTAAMVTSDR(W)
(K)NSPHTAAMVTSDRWDHLYPR(E)
(R)FGLPLGFGGPHAGYFATK(D)
(R)HIGPNLQQTAEMLK(E)
(R)NSILNANYIAK(R)
(K)ALVIGFEDGHSSNHK(L)
(K)FVTPQSIGISK(R)
(K)GHYNNELQVLDPTKDDTIIR(G)
(K)ILFLK(T)
(K)LILIDQETLKPVLFAEDNIFWR(S)
(K)NSDILATYILYYNAIHR(K)
(K)NSDILATYILYYNAIHRK(D)
(K)RYSEWPGK(T)
(K)TDKELQELNKDPVK(N)
(K)THTQGGISVTR(I)

(K)TQILIPLVEDVLGK(D)
(K)VGSGLAESLK(K)
(K)VGSGLAESLKK(E)
(R)FGADVISVLEDSDFGHINSIQR(I)
(R)YSEWPGK(T)
(K)IGIASDHGGFELK(E)
(R)HNNANVLVLGGR(I)
(R)RVNQLETLNS(-)
(R)TWINTSFEGGR(H)
(K)FGGYAEPGGFNK(G)
(R)MNSAFTNLNEELR(H)
(K)IYDSSYHLVYSSESTK(E)
(K)FLAELYHLDIVK(N)
(K)ALANFLFGNDDAMLR(V)
(K)HNYVGTEHILLALLR(D)
(K)ILNNLNINLNELR(K)
(K)ILNNLNINLNELRK(E)
(K)LEFLETPWTDYKPVDPK(T)
(K)NNPVLVGESGVGK(T)
(K)RVINEIAQDEAKR(L)
(K)TAIVEGLALAIVEK(S)
(K)VAEPSVDDAIQILQGLKK(A)
(R)LGSDYIGPEHILLGLLK(E)
(R)LGSDYIGPEHILLGLLKEEDSVAIK(I)
(R)LLGAPPTSSVGVSSTTQSGPQPR(Q)
(R)RFQVVK(V)
(R)RPTGSFIFLGPTGVGK(T)
(R)VDMSEYMEPHAVSR(L)
(R)VIQILSR(K)
(R)VLSLDLASLIAGTK(Y)
(R)HIGNPEYLSPTIVFR(I)
(R)IFAVTHGSGYQFLR(H)
(K)IYHSPYER(T)
(K)SFLFSPGSALAINVPR(E)
(K)GYIDFR(E)
(K)ISIPGFLTGEEFLTFSGK(L)
(R)KIPAELVALGANILK(Y)
(R)LFGLPFSTR(L)
(R)LGLASALISDPELLILDEPGSGLDPK(G)
(K)VFSIAFLTLR(E)
(R)LLSLIPSLGYNTPR(I)
(K)FLTPGIGYGDLK(K)
(K)LHLGNFFSVIR(K)
(K)SNIYAIHSLFLDAK(G)
(R)EVGPVFK(I)
(R)ILTGVQPSGK(L)
(K)INILVENHLLAK(Q)
(R)FLWFYSPAR(A)

(R)GTSGGVFGLLSGYEEVTQVGGSIR(L)
(R)MSEISSLR(A)
(K)AQNPAVVLLPHTSQGK(D)
(K)AQNPAVVLLPHTSQGKDYSPR(V)
(K)IVSSDLSGGSKVQLTEASIIVSGGR(G)
(K)KPIYSGK(A)
(K)VATYGIVGDLFEVVPAVTAEFK(K)
(K)VATYGIVGDLFEVVPAVTAEFKK(V)
(K)VGAGIIADVVGFSVDGGK(V)
(K)VGAGIIADVVGFSVDGGKVVAK(K)
(K)VIALLIGNGVEK(H)
(K)VQLTEASIIVSGGR(G)
(K)VTSPIQIFTVRPNSQEITQK(A)
(K)YIVAINK(D)
(K)YIVAINKDGDAPIFK(V)
(R)AAVDAGWISHSHQVGQTGK(T)
(-)MKIIVLVKQVPDTETSIK(V)
(K)HGGEVIAVSLGPDR(V)
(K)IIVLVK(Q)
(K)IIVLVKQVPDTETSIK(V)
(K)WIISPYDEFAIEEGIR(L)
(R)EKHGGEVIAVSLGPDR(V)
(R)KLEAADANAFASQLVK(A)
(R)TAYAMGADR(A)
(K)EHDDYETFNLSLFR(R)
(K)LSEFGQIQR(L)
(K)LVATQMAER(V)
(K)NIVADLK(K)
(K)NIVADLKK(I)
(K)QYITNGNVGSVFLVYAK(M)
(K)RTTSFIVESSFPGFSVGK(K)
(R)ALVYQVASGIHPESR(N)
(R)TTSFIVESSFPGFSVGK(K)
(R)TTSFIVESSFPGFSVGKK(E)
(K)LGSALETLQVLIDSK(S)
(K)AIMPILIK(K)
(K)TTITFENKPQYYNSPSGNVVPK(A)
(K)FLHVLSR(S)
(K)GPFYVLSHER(I)
(K)IPNGLSLELK(I)
(R)NVGLTFEALQK(E)
(K)HPFTDLHR(Y)
(K)HTAMLTSIFR(H)
(K)ILEFSSLLK(Q)
(K)ISYDYGIVNLNYHSSWK(H)
(K)KSELLQNLLDHSSSQELDLNR(I)
(K)LAGEVLAPLLK(Y)
(K)SELLQNLLDHSSSQELDLNR(I)

(K)SVIHF(-)
(K)YLPLVAGEGSQSHFMAGQYVTER(V)
(K)YVIGISR(L)
(R)HFHTPETLK(L)
(R)IKDDGTLNGHHILR(K)
(R)LHNDSIINETWEGTHLLLSEHVLR(G)
(K)EILYEIPGYSPELIHQIVDR(E)
(K)LSEALNQYYFER(N)
(K)EALLLR(Y)
(K)MTVAEINAAIQTSK(E)
(K)YILHLNAR(L)
(R)LEELAVK(G)
(K)ATLEGR(L)
(K)DLSAVDLGVLVSK(T)
(K)ELGLNPEITNVNGGAVAIGHPLGASGAR(V)
(K)KPLAIIR(G)
(K)KPVILEKDEFIK(G)
(K)MGIGPALAIPAALK(K)
(K)MGIGPALAIPAALKK(A)
(K)TGLPLGVPALTLNR(L)
R)EGGTVTAGNASGLNDGAAVTIVASSSYAK(K)
(R)LKEEIFTVTIPGK(K)
(R)TGVSPEAIGESIFGNVIPTGK(E)
(R)TPFGNFGGTLK(D)
(R)VTITLAYELK(R)
(K)AAPGMDETTIHVSWEGNNDSILK(N)
(K)SLVNLIETLSK(K)
(K)SLNLPEWVLESNIK(L)
(R)LANQVIELEPAHPAAR(K)
(R)LFQMGNTDLYR(K)
(K)GAEAAATAVEMVNLLSLL(-)
(K)IGSIGVQHSIPVIFGVLTTDTIEQAIER(A)
(R)FNEFITESLLK(G)
(R)MHGVEDVTVVR(V)
(R)FVNGVLDAILK(N)
(K)SIALTVLGVIHDESGR(Y)
(R)NSSSGSEFAGAFNK(D)
(K)NAVPALLVLAR(N)
(K)HLPFFMDVTLR(D)
(R)IHIVYPVSAFTIQNVLK(I)
(R)LVSYLSEIPIYEK(A)
(R)RPWNLEQK(E)
(R)YRPWLFVSMVK(A)
(K)EKSAIPELIQLLNR(N)
(K)IAEPGDSTIALKNK(I)
(K)IATAAAISLGK(I)
(K)IATAAAISLGKIAEPGDSTIALK(N)
(K)IISSENGDIVYASLASLLNITTK(N)

(K)SAIPELIQLLNR(N)
(K)SLSSGSDSEKYESAVALGK(N)
(R)RSDEFVADLLDLIK(K)
(R)SDEFVADLLDLIK(K)
(R)SDEFVADLLDLIKK(K)
(R)ILVENLFPK(E)
(K)GKPSLWIFPEAR(M)
(K)LLVMVR(T)
(K)NQTAFVLQPEK(L)
(K)TDLWTWVR(G)
(K)TSLVLFEELIR(K)
(R)IVAHVEVDLYGK(G)
(R)MLENFTQLQK(G)
(K)GYTNWELASSR(A)
(R)ISAASMGASRPALPNTSPENR(S)
(K)ALVVVVDNTAPTVK(I)
(K)NVTYTISAIDALSGVK(E)
(K)VSFAVDVNPPTTQIR(K)
(K)VSFAVDVNPPTTQIRK(V)
(K)YTSPITILK(E)
(K)FIELLSLFEK(K)
(K)NLAEILGVELK(G)
(K)DIGTDYYVLGIHTPAFQTPK(H)
(K)GIPVIEDISEVLGAQSATFIPGK(Q)
(K)TEIEFHKPTLSR(E)
(R)FIILAPGSYEQVER(Y)
(K)YLFLDVGDTILHLK(K)
(K)ATTHWMSLDVLK(L)
(K)IFLFAQSK(D)
(K)LFPVQISSER(F)
(R)TLTEFLVQNSK(D)
(K)IFLPEHGLFAELQDQVSGSSLR(Y)
(R)FLLDYLK(G)
(R)GTTRPFETFGAPYINEENIR(I)
(K)LANVLDQISGILK(T)
(K)LELFK(L)
(K)TMVADALAAFR(W)
(K)VMMWQDLSIHR(G)
(K)YLNALGSLPDPLRENLEFFR(V)
(K)YLNALGSLPDPLRENLEFFRV(-)
(R)IFFIGENSLVEK(F)
(R)IVLPIFAQGDLK(T)
(R)KLELFK(L)
(R)LTSGSPATHFPILTK(F)
(R)NVPKPVELMLK(Y)
(K)AVPYFPK(I)
(R)FVVEIIR(E)
(K)ILQILSPTDR(S)

(K)ETDYLFFLLK(G)
(K)FIILASVVER(E)
(K)IVSPYNTYLNK(G)
(K)LVNFTIPEGYNNR(Q)
(K)RQDFLLAASEPQLLR(E)
(K)YFLYLVR(F)
(R)IFEKDLK(I)
(R)NEERPLMAGVFNNR(L)
(K)ALTVSAGVIEEVTK(A)
(K)DKWLPLLASGER(M)
(K)GFYFQmEGFAGGR(I)
(K)GFYFQmEGFAGGR(I)
(K)GHFGAYGLDEDHEMFR(E)
(K)GLSILLAEKPTFTGHEFTYTQPEGGK(I)
(K)IFNDEINQFLENAmAIQNYNEIAEK(I)
(K)IFNDEINQFLENAmAIQNYNEIAEK(I)
(K)KALTVSAGVIEEVTK(A)
(K)VAEWVTR(E)
(K)VSVDKMDENQLVQYQIAWLTSEQR(I)
(K)WLPLLASGER(M)
(R)AHGVMQAALEAALR(Y)
(R)EAMQIHGGMGYAEEYAVSR(Y)
(R)GGLGIAGSLITRPEImSK(A)
(R)GGLGIAGSLITRPEImSK(A)
(R)GMHSFEVSFDNYFVPAENLLGGEAGR(G)
(R)GTGDLEQEMAVVFAAETVNHIR(S)
(R)HDDIIPEDIIGGLK(E)
(R)MAVILQASR(Q)
(R)VFSIFEGAEEVMALR(V)
(K)AALNMLTHTSASDFAK(D)
(R)LKDQFLDSDPSLNYTNSK(S)
(R)SIVSSPFTEVAGWIQLANR(K)
(R)VSELFWTFVPK(F)
(-)MTNLQLVIGDK(K)
(K)IWDTNSIVEYLAETFPEK(N)
(K)LSSWSMRPWILLK(E)
(R)SIVAEMHSGFSDLRK(N)
(R)ARPGGITGLAEK(A)
(R)IIAALDLGTSLTK(V)
(R)VIEAAQAIR(V)
(R)VTVIATGFHK(R)
(K)TFGIVGFPLSHSLSPLIHNSIYK(D)
(K)DFLKDPWAQTVPFK(E)
(K)FALTVK(D)
(K)FLIQDFGNR(N)
(K)GTIDSLKPFGVFVK(L)
(K)ITAIVNSAK(K)
(K)KAEIPVLGQFVSEGDSGADVK(I)

(K)LDDHFNGLVPGR(E)
(K)QISLSIQK(A)
(K)ITFQPMQYVR(S)
(K)LFDELDLEILENKK(Q)
(R)FEFFLK(N)
(K)AIQNIADSFQK(G)
(K)GIYDVLIGINLLR(E)
(K)HFITSGPKVKEAVENIR(A)
(K)IHSAYQPAGDQVK(A)
(K)VAYLHSEVETLDR(V)
(K)VVEQIIRPTGLLDPIVDVRPTK(N)
(R)INPVTAQTIFK(L)
(R)KQTLVDFGFR(L)
(K)EPVFQILTVLQGK(I)
(K)LSVQVHPDDVYAETYDPESSGKK(E)
(K)SFPLLIK(I)
(R)FHPIYK(E)
(R)IHAIGAGILLMEVQQSSDSTYR(V)
(K)AYLTHFGVWK(N)
(K)INAQGLVFQIER(K)
(K)ELFTAMQVIGK(L)
(K)HSLIHEDMINLFDGFPGK(G)
(K)IVGQPFVYPLDK(H)
(K)LFHDFYK(K)
(K)LPGWLAHWR(E)
(K)LPTEQQLK(D)
(K)SYQFPLISGTDGKK(G)
(R)KSVPEYFEK(F)
(R)KYIPLSER(-)
(R)QVAAVGLIEDILK(S)
(K)FLSPEFLESLLR(Q)
(R)YLSQYSYSENLPPGIVPTILSR(E)
(K)KLEQEVVNSSEITAER(W)
(K)KPFQDLGYLTLISPK(G)
(R)FIPYLHR(G)
(R)YWVMQVSIPNSIYK(E)
(K)GQQVAVIVSAMGHTTDELVDLAAK(I)
(K)IHDVFGLSS(-)
(K)KDVHEAKPILEGFSK(S)
(K)QITYEEMLELASLGAGVLHSR(S)
(K)SHVGVAAGMFK(A)
(K)YGGTSVGTPER(I)
(R)ITIAGVPDKPGLAAGLFGELSSK(H)
(R)IACETmSRALADLRGHK(N)
(R)AVLGLEILK(T)
(K)AISWVVPEELPPILIQK(K)
(K)LGGSLATSGSVSR(L)
(R)YIPLVSSEIADKEVAEK(I)

(K)AELLSYIK(D)
(K)ALGHPIVK(Y)
(K)ASPISQVLLEESVLGWK(E)
(K)ATGFPIAK(I)
(K)DLSELGFNLIATSGTHK(F)
(K)EVVLPFNKFPGVDTILGPEMR(S)
(K)IAALLSIGYTLDEIKNDITR(V)
(K)IGYPVLVRPSYVLGGR(A)
(K)IHLIINTPLSR(V)
(K)ILGTSPDSIDRAEDR(K)
(K)KQILDLLDGNLR(V)
(K)NEILYIIEVNPR(A)
(K)SVMILGGGPNR(I)
(R)AMLIINEEK(E)
(R)FVEVLEK(L)
(R)IYQNEKPEGVIIQFGGQTPLK(L)
(R)TVPFVSK(A)
(R)VPASGLANNLK(D)
(R)VPASGLANNLKDAVEIK(N)
(K)IPLGSLLPDFR(L)
(R)TVAINPNIHPDYPDDSPEMMLVK(I)
(K)NQFFPVFGGAALHGDGVR(E)
(R)ASASTENLLGFAGVLR(N)
(R)ELLAVLELLSK(T)
(K)ADMLAADERAEMIEMVEMDVR(E)
(K)ALEGDESEIGMPAILK(L)
(K)AVAYDQIDNAPEEK(A)
(K)AVAYDQIDNAPEEKAR(G)
(K)EHILLAR(Q)
(K)FAAEVYVLTK(D)
(K)FAIR(E)
(K)LLDQAEAGDNIGALLR(G)
(K)LMEALDTFVPNPK(R)
(K)LMEALDTFVPNPKR(V)
(K)NKAVAYDQIDNAPEEK(A)
(K)NMITGAAQMDAAILVVSATDGPMPQTK(E)
(K)RVIDKPFLMPVEDVFSITGR(G)
(K)RVIDKPFLMPVEDVFSITGR(G)
(K)TTLTAAITTTLAK(A)
(K)TVVTGIEMFR(K)
(K)TVVTGIEMFRK(L)
(K)VNDEVEIIGIRPTTK(T)
(K)YSFPGDTTPIVHGSAVK(A)
(R)AEMIEMVEMDVR(E)
(R)GITIATSHQEYETANR(H)
(R)GQVLAKPGSITPHK(K)
(R)KLLDQAEAGDNIGALLR(G)
(R)QVGVPYVIVFINK(A)

(R)TIGSGVVAEITE(-)
(R)VEQGVLK(V)
(R)VIDKPFLmPVEDVFSITGR(G)
(R)VIDKPFLmPVEDVFSITGR(G)
(K)LQLPAGVSVDIK(S)
(K)LQLPAGVSVDIKS(-)
(K)RTGATVSGPIPLPTK(K)
(R)TGATVSGPIPLPTK(K)
(K)NLLFVSGAVPGTANTVITIEK(I)
(K)NLLFVSGAVPGTANTVITIEKI(-)
(K)SVENDGYEAIQLAFQDIKEIQTSK(A)
(K)AFDSIFK(N)
(K)LSVASIYEAIK(A)
(K)LSVASIYEAIKAENANLR(S)
(K)NTGVIGLLVQGENDFLK(K)
(K)NTGVIGLLVQGENDFLKK(S)
(K)VVEDLDPKEYNTK(A)
(R)AVLSILGK(K)
(R)NLVITEAALK(E)
(R)NLVITEAALKEMLVQYGAQK(-)
(-)MNLQDVILTPIVTEKSQDLETIGANSK(K)
(K)VTPSSVNVQVYR(G)
(K)DHNLVSIGK(A)
(K)FKPVTSASR(Y)
(K)IGNAMPIGK(I)
(K)IPPGTNVHNVELQIGK(G)
(K)LPSSEVR(K)
(K)SVLDFAEITETEPYKPLTLTLNYK(A)
(K)VGDTVQSGAGSEIKIGNAMPIGK(I)
(R)DGEYILLKLPSSEVR(K)
(R)GVVmNPVDHPHGGGEGR(T)
(R)GVVmNPVDHPHGGGEGR(T)
(R)HPVSPWGQPTK(G)
(R)IIDFK(R)
(R)IIDFKR(R)
(R)KANIPAVVK(S)
(R)SAFISLICYK(D)
(R)TAGSFGTIAGR(D)
(K)KIYVDEGPIMK(R)
(K)RLSHITVVLSD(-)
(K)VILSASANASVLNNK(V)
(R)AIEPLTK(V)
(R)GYAVNEALDILK(F)
(R)LSHITVVLSD(-)
(K)INVNLHTAKPGIVIGQK(G)
(K)TMTDKPVNLNIIEVK(K)
(K)TTFGQIGVK(V)
(K)VNPIGLR(I)

(K)VWTYSGDFIQSK(E)
(K)VWTYSGDFIQSKEESEEDKYAVK(R)
(R)FNNAGVVK(V)
(R)GWDSIWFSQSDYKK(N)
(R)IPLHTLR(A)
(K)GNPEFWIAEIRPGR(I)
(K)LPIHTEFVK(R)
(R)GSSVSFGEFGLK(A)
(R)IFPHTPITK(K)
(K)LLTIQR(E)
(K)LQELKDSEILEQLEEAR(K)
(-)MIQQETLLQVADNSGIKK(V)
(R)RPDGSYIR(F)
(R)VGFETVK(G)
(R)VIVEGLNKR(K)
(R)VSRPDKK(E)
(K)ALEAAVEELALITGQRPVK(T)
(K)EIVPELNKK(F)
(K)FSSIMQVPR(L)
(K)INTLYGINMTFVTNSK(S)
(K)IVLNVGmGEAHTNPK(A)
(K)IVLNVGmGEAHTNPK(A)
(K)SNEEAYSLLAAFGMPYR(N)
(K)VDKINTLYGINMTFVTNSK(S)
(R)GNYNMSIK(E)
(R)LVNVALPR(V)
(K)LASGAQIPGVVK(S)
(K)LASGAQIPGVVKSSW(-)
(K)TSITVR(H)
(K)EEGFIKDYESVK(V)
(K)GIMTGK(N)
(K)GIMTGKNAR(K)
(K)NNIGTLIVSTSK(G)
(K)SILDLMK(E)
(K)SILDLMKEEGFIK(D)
(K)GVSQGWEK(N)
(K)NLEITGVGYR(A)
(K)QLVGQVAADIR(S)
(K)RGEDLVMSLGFSHEVVYK(A)
(K)VSGIDKQLVGQVAADIR(S)
(K)VVALHGLTR(A)
(R)GEDLVMSLGFSHEVVYK(A)
(R)LVFIK(S)
(R)SGMIFHGR(I)
(-)MENIIVTQVK(S)
(K)LTLHALGLR(R)
(K)AIHVIADGFSQSAK(E)
(K)AIHVIADGFSQSAKEK(I)

(K)ILDKETTLFK(I)
(K)ILGTGEIKK(A)
(K)KAIHVIADGFSQSAK(E)
(R)GFEGGQMPIHR(R)
(R)LEQASAFGK(D)
(R)RGFEGGQMPIHR(R)
(K)VPLQYGK(Q)
(K)AINLQVPDAELLKR(L)
(K)KLSQVNGVGSLEEVTSLIQK(E)
(K)KTLPLLDFYATR(K)
(K)LSQVNGVGSLEEVTSLIQK(E)
(K)NGFLLDGFPR(T)
(K)NIIFMGPPGAGK(G)
(K)NIIFMGPPGAGK(G)
(K)NRLDNYNKK(T)
(K)RYMDAGDLVPDSVVIGIIK(D)
(K)TLPLLDFYATR(K)
(R)LSIPQISTGDILR(E)
(R)TVEQAEALDTLLKNEGK(S)
(R)YMDAGDLVPDSVVIGIIK(D)
(R)ILPGDKVTVELSPYDLSK(G)
(R)MHYIR(I)
(R)IVVGLTYIFGIGNSLSK(L)
(R)SEIQLNIKR(L)
(R)VKDLNESQEAAIR(K)
(K)KSTPYAAQIAAGNAAEK(A)
(K)STPYAAQIAAGNAAEK(A)
(K)EQVIVELYSK(-)
(K)GPPGMPTK(R)
(K)RAYGLLEK(Q)
(K)SSYTFNK(D)
(K)TSSFIADNIK(L)
(K)VSEYGAQLR(E)
(R)AYGLLEK(Q)
(R)HHIDLPVK(E)
(R)KGPPGMPTK(R)
(R)LGFAITR(R)
(R)NFIAHR(H)
(R)REGVDLFLK(S)
(R)RLDNVVYR(L)
(R)YRGPVVK(I)
(R)YYEEASHAHGVTGEILLQLLER(R)
(K)ASLSKHVEELELSVR(S)
(K)DIEVLGTIPVDSIFSPVQK(V)
(K)FVAEPFER(G)
(K)GAGYFR(A)
(K)HVEELELSVR(S)
(K)IIHLELK(G)

(K)KDIEVLGTIPVDSIFSPVQK(V)
(K)KIEFNTEANTPNYGK(F)
(K)KKDIEVLGTIPVDSIFSPVQK(V)
(K)LSTLGLSFGMR(D)
(K)LTLEVWTDGSVSPDDAVAQAAK(I)
(K)VVFEVSETR(V)
(R)GFATTIGNSLR(R)
(R)GYVPAEEK(K)
(R)IEGVNHEFSFIEGVAEDVTR(I)
(R)IILNLK(Q)
(R)RTLMSSIEGAAISAIR(I)
(R)SLEIDFIGDLVK(R)
(R)SLEIDFIGDLVKR(S)
(R)SLNVLR(S)
(R)TLMSSIEGAAISAIR(I)
(R)TLMSSIEGAAISAIR(I)
(K)NLVTDLKPEVQLHNK(R)
(K)NLVTDLKPEVQLHNKR(E)
(R)DALINNMITSLFK(Y)
(R)EVVVKLFEDIAK(R)
(K)AGYEVFGGVNAPYIWLK(T)
(K)AGYLFPEISK(R)
(K)AQVVGTPGSGFGPAGEGYFR(L)
(K)EIQTSIAYYMANASK(I)
(K)EMGTVGGFHGYGPEQGYSFLLK(S)
(K)IRDGLKK(A)
(K)KAGYEVFGGVNAPYIWLK(T)
(K)KEIQTSIAYYMANASK(I)
(K)KNNSIILYDSAYEAFISEPGVPR(S)
(K)SIADHDYGSLGIK(I)
(R)LGIGDVTLPIVPSVVDAMVEASK(E)
(R)YSNLIYMPATK(E)
(K)AFGILTGK(L)
(K)ETTVLHPLPR(V)
(K)GTLDGLTVAFIGDLK(Y)
(K)KETTVLHPLPR(V)
(K)MVEAYADIAVIR(H)
(K)NVLDTEQFSKPDLDFLIGK(I)
(R)LFLISPPELALPESYK(K)
(R)TIHSLINLLR(H)
(R)VISTVGFQFSSISK(G)
(K)DKLPALR(V)
(K)LPIEPENLPGVDIVVISHAHR(D)
(K)NFFSVLR(W)
(R)FAQGFSATILPIGAFKPR(W)
(R)MGLTDMNQYHWGSYAFEFENIR(I)
(R)MHILTDPIFTGVPPFVK(R)
(R)GFILDPPTFGR(G)

(K)ELEETALHDPYFVER(K)
(K)FPTIAAFAYK(K)
(K)LYPNVDFYSGIIYR(A)
(R)ALGIPVNMFTVMFAMGR(L)
(R)HGGANQEVLEMLQEIQASGLPVK(K)
(R)HGGANQEVLEMLQEIQASGLPVKK(I)
(R)HGGANQEVLEMLQEIQASGLPVKK(I)
(R)HISMIR(L)
(R)KLYPNVDFYSGIIYR(A)
(R)LPGWIAQWK(E)
(R)LSGFGHR(V)
(R)QIYTGSTETSYK(E)
(R)QIYTGSTETSYKEAK(K)
(R)AALFLPFLNPSEK(K)
(R)QESFFKENAVSVSNARGLmQImGPTGK(E)
(K)IFFEELLER(A)
(K)ILEYFSGALNEVHSPFSSLR(I)
(R)ILTLIESLSSEEKESFFK(T)
(R)IFIAEAYEMFQK(F)
(K)DLIDLEYSKDSLSVEK(I)
(K)FFINGSLK(I)
(K)SLQAEQLYIGVTGK(V)
(R)TPELIIQNGVILEGR(C)
(R)VMLLPTSK(I)
(R)VMLLPTSKILGDIR(T)
(K)DLQVLPLTTHVPLVK(V)
(K)IGKEEVEILEPMIQFLK(K)
(K)ISERLIAEASSQILK(K)
(R)GVNLTLGLPFIR(V)
(R)LIAEASSQILK(K)
(K)ALVESAITPGQK(Q)
(K)AIMEELKTTGK(V)
(K)KLNLSGGAVVVQIMNDSPADR(A)
(K)RPAQAWLGVGVDYLHEDDAK(K)
(K)RPAQAWLGVGVDYLHEDDAKK(L)
(K)TFTAELIGSDPVIDLALLK(I)
(K)VGDWAIAIGAPLGYEQSLTAGIVSAVGR(T)
(R)AGIQLmDVITEISGTK(I)
(R)AGIQLmDVITEISGTK(I)
(R)ISIQLK(E)
(R)ITVTVVR(Q)
(R)MIASQSGGSVGIGFAIPINEAK(A)
(R)QAITIQQAFEEVYQTASPSVVSIATER(I)
(K)TTLAGIISNELGTR(L)
(R)LLTSMGENEILFIDEIHTLPK(K)
(R)LTITSAPVITK(G)
(K)HLSANGHPVYTVPLGFQVGNIR(K)
(K)LLETFLIEK(N)

(R)KDQFILPSENSR(L)
(K)TLSDLQSLMDRYPDLGEFLR(A)
(R)AWVILK(E)
(K)IHPLESQIR(F)
(K)VSTWIHSFEK(I)
(R)FFHPVPAGR(K)
(R)NIFGEYNPAYLWR(I)
(K)ALGPYPGHHLGK(I)
(K)ELGLTIEAVVSGWHMK(G)
(K)GVHPNNMGIGQAVATK(A)
(K)INPNGGSIAIGHPFGATGIR(L)
(K)IQDVDYVEIHEAFAATAVGALEQIK(M)
(K)LAFESFKR(A)
(K)LKPSQIDGVVVGEGFSNAPNSAR(V)
(K)SHGPTVSIFNASPR(S)
(K)YSPYIIPMKDDEGNELTIDEAVGLR(E)
(R)AMLmFDNPGMK(F)
(R)AMLmFDNPGMK(F)
(R)LIANAIMDLK(E)
(R)LIANAIMDLKEDSSANK(V)
(R)RTPFAQIAK(A)
(R)SDGAAGVIVTTVEK(A)
(R)VIANLVGMR(D)
(K)GQDYQVIGTGGLVTVIDAAHPGIFDK(I)
(K)LPFSISYPRPYEIGADR(L)
(R)MFQDWFK(I)
(R)NTSQLPPIVFQSPGK(I)
(K)LGISFINDSK(S)
(K)STNLHSMLAGMATWK(N)
(K)GRLDVSIANEVEENLNDLIDNQGHKK(V)
(K)ISNIKPAVK(R)
(R)DGVLIVYLK(G)
(R)LDVSIANEVEENLNDLIDNQGHK(K)
(R)LDVSIANEVEENLNDLIDNQGHKK(V)
(R)ADYRPVQIAK(T)
(R)KAWLNLF(-)
(K)FSLPELLQAAK(D)
(K)LAGHFHDTYGMAIANVEK(A)
(K)LLEVLLK(E)
(K)SGISTGIDPNLLWEASLFMEK(S)
(R)TISESIEGFKDIFNFSK(I)
(K)DKFGVSWQIIPSILGDYLQDKDPK(K)
(K)LYASTFSDWK(L)
(K)IHGYVYK(R)
(K)VHIGQPELK(T)
(K)VLEDVKGDKIHGYVYK(R)
(K)VLLFAESNNK(V)
(K)VTQDQEFLTEKTGK(N)

(K)DHTLFALVTGK(V)
(K)FRPGNNVGLGK(D)
(R)FGGESVLAGNILVR(Q)
(-)MESFVDEVAIEVFAGHGGAGSVHFR(R)
(K)IAGYAFTTLSPNLGVVK(R)
(R)NELSTYNPELLNRPYLIVLNK(I)
(K)EIIR(K)
(K)ISKEDLGLAGGPSGPGTGGMFTK(L)
(K)QALAAMGQNR(L)
(K)SAGLLSEAGIPTAILNGK(K)
(K)VILVSSGAIAR(G)
(K)VVPFLEK(I)
(R)LVNLYDSFFSK(V)
(K)LFHEILIK(E)
(K)LSSALMDR(L)
(K)TSSGHTEAIVTEDLNTAR(I)
(K)TVSSLEEALAFIEK(T)
(K)VIPIVINSK(V)
(R)FHDGGEFGLGAEVGISTGK(L)
(R)GGSEAFYSNEILIK(L)
(R)GITLPNGLELVTR(R)
(R)IFSMASAVR(E)
(R)LLLNEKR(I)
(R)RVPLGVVMVIYESRPNVTIDVGALSFK(S)
(K)EVPLNSHLNSLFEFK(F)
(R)SGPSYTIQTIQELK(T)
(K)AAGISGVTTLVISGDLPFAMGR(F)
(K)AYGTHIADGPLK(G)
(K)GNPVQLEGK(I)
(R)AVFVLDK(S)
(K)EIGQLK(K)
(K)VLMLTGNQLTSLPK(E)
(K)EIEKEDIIELELVQPK(N)
(R)ILPESIFSASK(K)
(K)GLNGIYFGMSIDEVK(S)
(K)LYSITTSNPSIQLFGKPIIGESIETIR(E)
(K)FVGYSFQISK(N)
(R)AGFWGLLQPLADGIK(F)
(K)ALQMKPDDIIAEVK(K)
(K)GFFYIR(G)
(K)KALQMKPDDIIAEVKK(S)
(K)LEFYESVHGYDGMK(K)
(K)LIENLPHQIIEGMIIGAR(A)
(K)LLTKYIDNPQSTK(L)
(K)VKPIVPDHPR(R)
(R)KLIENLPHQIIEGMIIGAR(A)
(R)LKPPFPAVSGLYR(S)
(R)SPTVVNNVETFSAIPHILDK(G)

(K)GLKPAIEEFNSLLLK(N)
(K)VYNNMEELIYHFK(I)
(R)IGGLEKDIYPDFVKEVK(L)
(R)IIEQLVDGIPSGPWHADLPHAYLPEK(H)
(R)IVEAESVIGYVHR(C)
(R)MNYVSTPLNNIGWILAVEK(M)
(K)FPHFLDSQEEVISNVPVFFLK(V)
(K)NDPTLHYNFLNDLTAVDWLGK(K)
(K)VESFLK(E)
(R)EVYDLFGISFIGHPNLDR(I)
(R)FEVNYLLR(S)
(R)ILMPDNFQGHPLR(K)
(R)ILMPDNFQGHPLRK(D)
(K)AAAVGEVIYLYQFA(-)
(K)EIGVSDYHTVTQEQVNK(F)
(K)FADATLDHQWIHTDPDK(A)
(K)MGINYGMEK(L)
(K)VVLSSFAELAAYAGK(E)
(K)LEVLGLVAR(T)
(K)NLSPLTTSHADILFALVK(K)
(R)SLLGNLYR(G)
(K)FLGQFFTPER(V)
(R)LGHFVPLTEFGK(F)
(R)VAHFLVDWVLGAER(I)
(K)NFLQAMDILER(Y)
(-)MKEVSVPAIK(R)
(R)FLSGPLGGDQQIGAK(I)
(R)IVLIAHDNR(K)
(R)IVLIAHDNRK(E)
(K)AAPYTGTLPYTESTYLK(I)
(K)ILASIPPSGSVLK(V)
(K)LVSIYPQNPLYLYLK(A)
(K)NLSLNPTITLSEVLESAR(N)
(K)SFLGDYPDLALLISSSVR(K)
(R)KNLSLNPTITLSEVLESAR(N)
(R)NYTAAYQSFSEASR(L)
(R)YLNLLLFLR(K)
(K)FILIPGEGSEK(N)
(K)NLYNSFLSHK(D)
(K)SGLVIYNTIDNTWR(L)
(K)VLNHDYPDISVFLR(V)
(R)GFSYFNNSQYSSAR(E)
(R)HRVEIFSPSNQLSSNMDVVLEK(V)
(R)ISLSLVYENTSEVK(A)
(R)IVLVLVSGNSYPDSFTQISSEK(I)
(R)KLNQPFSSTFDYTGTQFIADFNR(H)
(K)ELFGTHIANR(L)
(K)IIEGLLETPEQNKPR(V)

(K)ILTLSFNNPETR(N)
(K)ILTLSFNNPETRNSmTREmGLEFK(K)
(K)LGIHPGMGSSYIVK(E)
(R)ATEIAIALSESAPLALSELK(K)
(R)ATEIAIALSESAPLALSELKK(N)
(R)VVILTGK(N)
(K)ILFFTDIK(E)
(K)SAVYSSGSIQAQK(L)
(K)SSMFSDVPSFLKK(I)
(K)ETIPSTILHFSDSGK(L)
(K)FILGTLK(E)
(K)LLNTIYVEGISGNTPFGYIER(M)
(K)SGYLEIHK(W)
(K)VLNGVPTNLPQTIPVVSSK(L)
(K)YVVSSFSIFDK(K)
(R)LETILPHFEGK(Y)
(K)AIDASAEIGSVISFR(E)
(K)KSGLEESSELEKPSPVTVEFASGK(D)
(K)KVVSGNPSSALEISLEDAK(A)
(K)LASQLAGYK(I)
(K)LQGIGHTTAGQILK(L)
(K)SEGIKDLETLIEYSQDDLAK(L)
(K)SGLEESSELEKPSPVTVEFASGK(D)
(K)VEGIMPR(R)
(R)IVGILK(S)
(-)MTHTISGQYGR(D)
(K)ASSTLAGTAPR(I)
(K)AYLHELEYEIVR(E)
(K)EVVEFFKDTIEDSDKIK(D)
(K)GITAFQMDLK(V)
(K)HIAEFVTLQEEYAK(K)
(K)LVLPKPDEFPYVIR(V)
(K)MVDGFFAEVEVGK(I)
(K)RIADFGAFVEILPGK(E)
(K)SFMLHYNFPAFSVGEVR(R)
(K)SFMLHYNFPAFSVGEVR(R)
(K)THIVMIEGEAK(E)
(K)VKEYAFAK(L)
(K)VTGVSFDVLESVFEQAQR(G)
(R)FHILDIMEK(H)
(R)GQTQSLGVVTLGTGSDNQR(Y)
(R)IADFGAFVEILPGK(E)
(R)KGITAFQMDLK(V)
(R)SISVEINPLPGPHGSAVFTR(G)
(R)VNGELILNPTTK(E)
(K)RKPDESYIVLEPVQEK(F)
(R)KPDESYIVLEPVQEK(F)
(K)AWPHILK(I)

(K)IFEEYPNTSSLHQK(L)
(K)LILTEILEK(F)
(K)LSDLFLPLLQK(L)
(K)NFMNTHAGIK(N)
(R)SYIVSNIQK(T)
(K)AATSGTNILILSDK(K)
(K)AATSGTNILILSDKK(L)
(K)AAVDNDYEAFKEATK(I)
(K)AMLVPNAWSK(N)
(K)ANADVILISGHVGGTGAAPITSIK(Y)
(K)EIHPIIRPHMSDSASLDNAMEAIVR(S)
(K)GVQITLHGDANDYVGK(G)
(K)GWDIQPLLNK(E)
(K)IAQGAKPGEGGQLPGK(K)
(K)KLFATYIEEMGHR(R)
(K)LFATYIEEMGHR(R)
(K)LFLIQK(K)
(K)LTSSDLSEGILKDFEANK(N)
(K)LVSEAGVGTIAAGVAK(A)
(K)NLTMSEELK(S)
(K)NMGAGMTGGTAYFFQK(G)
(K)SSMALSYLVR(N)
(K)SVIKPQAHQGK(E)
(K)YAGSPWELGLSETHQVLVmNGLR(D)
(K)YAGSPWELGLSETHQVLVmNGLR(D)
(K)YFPGTYSR(I)
(R)APIPMELAVAAVHNHLIR(N)
(R)DRLDSLDLNPILVR(L)
(R)EKGVTSLYTR(L)
(R)FAQVTNPPIDYMR(E)
(R)FGVTSEYLNSATEIEIK(I)
(R)FLTPGMSHGALSIEAHTDLAIAMNR(L)
(R)IGLYTYFR(Q)
(R)ILAHNGEINTIAGNR(I)
(R)ISGIGIGGIEQNILR(N)
(R)IWMLAR(E)
(R)LGPGEMIGINLK(E)
(R)QILFAYSPFK(Q)
(R)RPDEDSIGVGMIFLPR(E)
(R)SLVESALMEFNFK(L)
(R)YSTNTFPSWALAQPFR(I)
(R)YVPVNPEVLGPK(A)
(K)DYIYALDHR(T)
(K)IQLNIDILR(Y)
(R)YAAIVADKDIFEGAK(K)
(K)ILLATNAFGMGVDKPNIR(T)
(K)IVSIPGTISK(N)
(K)LISSGLTKEAR(E)

(K)DLNTTQSSLEEIFVQLVSK(-)
(K)GLEIIGNGK(Q)
(K)QLLYTYDSHGAQTGISTLLQDLK(K)
(K)SPIVSVNRLSKYYANGFQALK(D)
(K)YYANGFQALK(D)
(R)ILFLDEPTAGVDVELR(K)
(R)ILFLDEPTAGVDVELRK(D)
(K)NFLVKINANIGNSTFSSSIEEEVEK(L)
(K)YGVSISLGDGLRPGSIADANDK(A)
(R)YIPLTTNR(I)
(K)IERIPK(L)
(K)IQTDHSMSLISGK(N)
(K)IPLIGR(I)
(K)DIHPLYSYLIQNSPNPGEVEWNFEK(I)
(K)VVMVVNVASK(C)
(K)TPVILSLEMLER(D)
(K)NYPESAVAALLQAVR(D)
(K)DVVILLDSITR(L)
(K)GTGNMEIHLDR(K)
(K)ILSGGVDSNALHKPK(R)
(K)KQNLIFAILQAQAER(D)
(K)LFGLR(T)
(K)QNLIFAILQAQAER(D)
(K)RFFGAAR(N)
(K)RGGYEGPAPAPIDLVELK(K)
(K)VLSPMSITESMELLLEK(M)
(R)ALIVAPPR(T)
(R)AYNQVIPTSGK(I)
(R)DGQVHAAGVMEK(L)
(R)FFAMLR(V)
(R)HVQVAEMVIEK(A)
(R)IFPAIDINK(S)
(R)KRGGYEGPAPAPIDLVELK(K)
(R)KVLSPMSITESMELLLEK(M)
(R)LVEHGKDVVILLDSITR(L)
(R)TGDTVEGQIRPPK(E)
(K)ANQDNISHTALQLGVPR(T)
(K)LIGLLSR(K)
(K)YFLLTPSTHLPVVDNSGK(L)
(K)FFDLILK(M)
(K)GINAVYDSPKPSIAAVQK(H)
(K)TAILYLNRPEK(R)
(K)TAILYLNRPEKR(N)
(R)KFFDLILK(M)
(R)KRPVYNQS(-)
(R)LPSIIGQGHTR(E)
(R)NAMNWPFWR(D)
(R)YATVDASISLR(E)

(K)NGSSHEIQFQSSPIFGGEEK(L)
(K)NNLTIISSIFSLR(S)
(K)RNPSSLGLSLVESLAK(Q)
(R)AVALLHEILYESK(D)
(R)IKNNLTIISSIFSLR(S)
(R)NPSSLGLSLVESLAK(Q)
(K)IVEESFGGTR(T)
(K)KIVEESFGGTR(T)
(R)EVTVSGTMIGNIK(A)
(R)IDGKFEGEIK(T)
(R)LLGDIIAPALHLAK(G)
(K)IYSTLNIQHQTQAEK(A)
(K)QVFPLSVLYVEDGSGKVIK(D)
(R)AYAVIASGGK(Q)
(R)FFVIHFR(D)
(R)GAGFIPEPEPTTHPTQPK(K)
(K)HPLVVVSK(E)
(K)IGPPVVELFQEFAK(K)
(K)IGPPVVELFQEFAKK(H)
(K)TADYVYVGHCCESGDLFTQAEGGGPITR(I)
(K)TSSFGIWYEDINQVK(E)
(K)VHTHIGSGSDPEVWK(V)
(R)AIHYGFKPEQIMLTSQELAK(G)
(R)FNPGLGSGQTK(K)
(R)FNPGLGSGQTKK(T)
(R)QLETFGNLFPGK(E)
(R)SIAGQFGTPVFVYSR(E)
(R)TVLEIMK(R)
(K)IEPISINLFLR(N)
(K)LAAESISNIAGATK(I)
(K)STHTGIVFIQIK(Y)
(K)YVVSGNNTSLSVVR(T)
(R)NTPVSQMGFGLPLILGVK(E)
(K)EYILSQVGIK(G)
(K)TWVFLLPDYK(G)
(K)IIIPGIK(I)
(K)LFPDALEHFYVR(E)
(K)TDSFLDLPVNFLNVGMNEIK(I)
(K)LVSWNITSPDKK(I)
(K)LLIQIESYK(E)
(K)GNLFQEPIR(T)
(K)ILQELPLGLK(I)
(R)NFLPVYHLWK(N)
(R)NYLLDLR(G)
(R)KGEETPIGYFLFR(E)
(K)AYLEYGINLR(K)
(K)IVLEWDAHEGGYESQNELLTTGGYK(T)
(K)QLSGYFTTGGASGPLKPGTEVIWK(F)

(K)TFLQSLFLPEGR(F)
(K)EASPPNGFQGEYHPTEK(I)
(K)LISSNPLPLYNEYNQALLK(S)
(K)SNWEDVILEVLSK(K)
(R)FFSFLPEGSFLK(T)
(R)GSWNQIVDLR(N)
(R)TGPGTENPISFQFK(G)
(K)EGSFHATQMLAYGTK(V)
K)EGSFHATQmLAYGTKVVAGVTPGKGGSK(W)
(K)GGSKWEDKVPVFNTIK(D)
(K)GIVMIGEIGGTSEEEAAEYIK(N)
(K)KPVVGFIAGQTAPPGK(R)
(K)KPVVGFIAGQTAPPGKR(M)
(K)LGIMPGFIHSPGSVGIVSR(S)
(K)LVGPNCPGVITPR(A)
(K)VPVFNTIK(D)
(K)VVAGVTPGK(G)
(K)VVVQGITGK(E)
(K)VYSVLR(N)
(K)WEDKVPVFNTIK(D)
(R)MGHAGAIISGGLGTATSK(M)
(R)MGHAGAIISGGLGTATSK(M)
(R)SGTLTYESVAQITK(Q)
(K)ANVPFGVVIDK(K)
(K)ANVPFGVVIDKK(E)
(K)AVDLKVPLVVR(L)
(K)EYYLSILLDR(S)
(K)GIFVNIFGGIVR(C)
(K)IAIDPGIGLQVNQAR(Q)
(K)TGGSVVVVK(A)
(K)TIIMASTEGGMEIEEVAETHPEK(I)
(K)VPLVVR(L)
(K)VYLEQGIDIAK(E)
(R)DITEEDPLEVQASEFNLNYVK(L)
(R)HKANVPFGVVIDK(K)
(R)LQGTNSELGR(E)
(R)QLAFELGLPAESHK(S)
(K)ANEALQAILEFESK(V)
(K)ATEQALNYSHIEEIQLDK(I)
(K)EFPIQIWK(H)
(K)ELLALHER(L)
(K)IEVLEHPVHPR(N)
(K)NAIIWHITSGK(E)
(R)VNIELSK(V)
(K)GGEFGATTGRPR(R)
(K)INLVSTGPDRK(D)
(K)KNIINTAYYLDTELKK(G)
(K)NIINTAYYLDTELK(K)

(K)VIDFLSK(D)
(R)YQGGANAGHTVVVHGK(K)
(K)NASGYALMVNTLESSLK(T)
(R)IRDLSGNEISPSIDLTVQAVK(G)
(R)RTLLETISGVLK(K)
(R)TLLETISGVLK(K)
(R)TLLETISGVLKK(K)
(K)APFFNVVK(G)
(K)EFFENIHLFK(R)
(K)FLYLSSVTPMPPVMEVFK(E)
(K)ILPTTSLLFNQVNGK(L)
(K)ISGFEIAASNATLIPR(V)
(K)IVAIFPEGTR(S)
(K)KQFLIDNLALLVGSNLNK(H)
(K)QFLIDNLALLVGSNLNK(H)
(K)SIAEQLVFIAGR(L)
(K)VIIPISLEK(T)
(R)EFMMEFLSPIMK(Q)
(R)FFTEMTTIGTK(M)
(R)KISGFEIAASNATLIPR(V)
(R)LMPFVETVYHYVANK(V)
(K)LYELYSAR(K)
(K)NVPPMGTR(F)
(K)SNQLRPANWTSADAAGLPILPGLVR(Y)
(K)LRPNAELPLLQTK(Q)
(R)GELMIPLLNLGDSSFIIEHGMR(I)
(R)IAQLLIR(K)
(K)HSILTPHVAGYSQLGR(I)
(K)SGTIFINTSR(G)
(K)TESRPLSEVLDASVLSFHVPLTK(D)
(K)AAIVSTFGK(M)
(K)DTGNYVFFAVNSGHK(N)
(K)FMNNYFFEPNSSLPVVPK(I)
(K)LLLDGEIFAFGLSEK(E)
(K)NMYFEMAAK(D)
(K)YALQLYSK(R)
(R)NQTWYADFLEFQK(S)
(K)MMTYIPNTDEIHIYPKPPVK(K)
(K)VIDEVVFSELWK(L)
(K)VIDEVVFSELWKLGEER(K)
(K)ADFQIQTVSNKK(S)
(K)SNKSGPIFSNFTDEGLSRIFR(K)
(K)TLYIYIAR(L)
(K)YFVLNLEQR(E)
(K)GGAVNTAVLAFNVYPELTVYK(F)
(K)GVSFSVSNQQCK(L)
(K)IEESQAPLEELTRPTASYYTK(I)
(K)ITLGRPGK(L)

(K)KLDIPENIDFLSR(S)
(R)GEKIEESQAPLEELTRPTASYYTK(I)
(R)IISQAVTENSAK(V)
(R)SIPENR(S)
(K)IAPYLESPLQHVSSK(I)
(K)IENYSKPYAYVK(V)
(K)VNLFSYSPQEGTK(G)
(R)YPDNIHSEIPEVDLFFGTGK(Y)
(K)GEVSEILLSSFLTAMR(F)
(K)HLTTLEAESFMNHVMK(G)
(K)VLNSESIAGTHAVALNAGAGLFVMGK(I)
(R)GFTFLFAPMWHPSMK(H)
(R)GFTFLFAPMWHPSMK(H)
(R)LGYQTETTQEEVEAHLVNR(G)
(R)SVSSHTGSSDILTR(L)
(K)DTNVSFTASTVLKK(K)
(K)GDEVVTSSGIHGK(V)
(K)KGDEVVTSSGIHGK(V)
(K)VVEIKDNNEVVVLNIAK(D)
(R)VFIIFEEK(N)
(-)MIQPIENAIEEIK(S)
(K)HGLNIYTIEDLIR(Y)
(K)HGTAFTVSVDAK(H)
(K)IHIALIK(G)
(K)INFMAAFGR(G)
(K)LGFAPDLR(D)
(K)TVSADLMRPGHLFPLQAVPGGVLR(R)
(R)AVTVQVLLDDK(T)
(R)VPIEIQPGSDNHDYLMTK(K)
(K)LFITHWK(K)
(K)NQNVEIVFPSVSILAEPSVAVVTK(N)
(K)NYYRPTDKK(V)
(K)SLYSNVLVLDSGAR(G)
(R)AVIDGLEADVVTLALAHDIETIAEK(T)
(R)GIGDVLISWENEAHLALQETAK(N)
(R)WNYLAAWGYAK(E)
(K)ILFTTNLYSFK(G)
(R)IVLFLK(M)
(R)TVLSTGSPLTTSGFR(Y)
(K)FSGAYLVATPAALIR(K)
(K)SIASNFRPLLEPVQR(E)
(R)NFEFFLQELSR(T)
(K)DYIYPHDFGGFK(E)
(R)EGTILLFLDEIHR(F)
(K)IHSTDSVTHDLNAFLDLAK(L)
(K)MRPPFSVTNLSALAATEALK(N)
(K)SYEAGKPIELVVR(E)
(R)ITFAMYK(I)

(R)ITIGRPEQNDLVLEALEKEFH(-)
(R)SILNSLK(S)
(R)IILVR(E)
(R)LLSEVFQILPLSMQTQK(D)
(K)FIQGIHLSR(T)
(K)IMLTGQASADAVGNALNHANLYR(Y)
(R)VNVSIGVATHQHSLYYKAR(I)
(R)YLSKPWDSNDLILTIR(E)
(K)INYNWKNVDHSK(A)
(K)ALLLFR(E)
(K)EILVYYNNLIR(L)
(R)EGLHFQDPSALLSLIR(E)
(K)DVLMISVAKPLR(R)
(K)GFAVVAEEISK(L)
(K)IIFEIK(G)
(K)LLLSLNQMK(N)
(R)FIPYLNK(S)
(R)INEILSIITDISEK(T)
(K)NYLSLYISHEYFK(E)
(R)GLSFSGEMANR(R)
(R)SNQYSGIAAPIER(E)
(K)EGQIAILTIQRPSALNALNR(E)
(K)KTIQQGLDVSLK(E)
(K)LGLPEVSLGLIPGFGGTQR(L)
(K)SIANSILK(K)
(K)TIQQGLDVSLK(E)
(R)EVLIQIGQEVDALEKDENIR(V)
(K)HLILQDGK(G)
(K)KIELSDESNYLISK(I)
(K)ELSHINLSQEFSIR(L)
(K)FRIPLFLR(K)
(R)TGTLSLFGQSETK(S)
(K)LWDTSDTSSQTGPVITHIQVEYR(K)
(K)VAWGDMDAFGHVNHVVYAK(Y)
(R)ETILQFLEK(F)
(R)ITSAISEALSATK(S)
(-)MIVQLYGTR(G)
(K)TLVLTHHEPVYSDDVLDLILEEAR(A)
(K)VTQILEIVR(D)
(K)MLTNVAMAGTAAMGLR(D)
(R)GQAASVLAVPIWGR(M)
(K)ATLTLPEIPK(A)
(K)ATSSVSNASVNVGTPAAVK(A)
K)ATSSVSNASVNVGTPAAVKATLTLPEIPK(A)
(K)EAVTFLVK(V)
(K)FKEAHNVSLGFMSFFTK(A)
(K)GLVVPVVR(D)
(K)RAVVVNDQIVIRPmMYLALSYDHR(I)

(K)TIPAINAEIR(G)
(R)KLIEDNGLNPASITGSGK(N)
(K)DLEADVVLVAVGR(R)
(K)HIVVATGSVPIDIPGLTVDGK(N)
(K)LEVHGISVGK(V)
(K)LLGAFVFGPR(A)
(K)SLFRPNAR(S)
(R)IQVDAHFK(T)
(R)LGTSVTVVEFLPGLISNVDR(Q)
(R)RPFLEGVGLEEAGVAFTQR(K)
(R)SFHAHPTLSEVIK(E)
(R)SLTSQGMEFLFEHK(V)
(K)IRVDTMQGVWSK(F)
(K)LDPLGIQKPNR(T)
(K)LFQADYFETFLAK(K)
(K)LHLQEQDQLVLDAFQA(-)
(K)LHNISPADIDTVVDSETLGR(V)
(K)LLAEQMHGIVQALTSVPQGFTPNSK(L)
(K)LMALYGENGALLEELYNQYK(L)
(K)NQGAWFFVR(E)
(K)YHLGYSNSR(M)
(K)YIPLNHISSK(Q)
(R)EmGIINLLNAYR(R)
(R)EmGIINLLNAYR(R)
(R)FPASLSPVEDILQGAFR(E)
(R)HAVLVDTNTNEK(Y)
(R)KPLVIVTPK(S)
(R)LGHNETDEPAFTQPK(M)
(R)LSGLIMLLPHGYEGQGPEHSSAR(L)
(R)STLYATDLAK(G)
(R)VEQIYPFPAK(E)
(-)MYQLTLPDK(S)
(K)ALAVR(L)
(K)EGPGFPFWHPK(G)
(K)ELPFLK(N)
(K)FGFSEFK(T)
(K)GTILWNSLAEYLR(S)
(K)KANYLLILGEK(E)
(K)NSNIEVLTYSEK(L)
(K)SQGKDEDWEFATSTLK(Q)
(K)TFIATRPEK(S)
(R)AIYGSLER(F)
(R)DFIEKELPFLK(N)
(R)FAEFGSVHR(H)
(R)GPHVPNSGILK(S)
(R)ILTVTEK(V)
(R)IYGVAFPTKK(E)
(K)FELLKK(S)

(K)SATDKLFNHR(V)
(R)GLTLVHNSDWK(S)
(R)HILTK(K)
(R)LMPYGVR(-)
(K)LGIHMDR(K)
(K)LGIHMDRK(S)
(R)ENGLSYSVFMNSLK(K)
(R)KSLAELAFNDREVFNSLVEK(I)
(-)MLTMETIEELESK(V)
(K)ALELIADLR(A)
(K)INQLLGR(L)
(R)LESENETLRAENDQMK(L)
(K)LEFILPEAWESGPFGILEPK(G)
(K)LIGLTYEELFPASFAK(E)
(K)KIDTPFMDMIQK(L)
(K)NLSIINDIFR(A)
(K)TDREEFPVVIVQYK(G)
(K)VIDALGEPLLHLIR(N)
(K)VNLVLSGESTELDKK(V)
(K)YVIANVSQGK(K)
(R)FSLIVQNLK(N)
(R)LEAEEVKELEEEIRK(S)
(R)MVQISTVFR(R)
(R)SHITEIALDQSASEDEQKR(A)
(K)SGIIGVIMTGMGK(D)
(R)IPADISLPILVVQHMPAGFTK(A)
(K)DFVVKPFSPER(L)
(K)TLKPDLVTMDITMR(E)
(R)IIMVTALGQEDLLAK(A)
(R)LQQAADK(A)
(K)ILFTLR(E)
(K)EVPGRPALYGTTK(E)
(K)GLIEALLFVSGEPLK(L)
(K)SSLDTLAIIAYK(Q)
(K)LISELPTDFSGIITDLGSTKK(E)
(K)MWAAIFGSNQDEIFR(S)
(K)KIPIQSYMDHR(I)
(K)NIGLNPAR(T)
(R)IAMSFLIFK(T)
(R)TGILTALQSMGAK(I)
(K)EMVIR(G)
(K)FGAFVELK(E)
(K)GYIVNVEGVELFLPASQLSYK(F)
(K)ISLSIK(D)
(K)IVSVDNDYVYVAIEGLK(Q)
(K)IVSVDNDYVYVAIEGLKQEGR(I)
(K)KGQEVEALVLDIDFK(N)
(K)LALGLK(Q)

(K)LLDEVNEEKWDALLLK(L)
(K)QYFQVGQEVELVILELDKENNK(L)
(K)STFAEVFK(Q)
(K)VANPTSQLK(K)
(R)ESLGSFLNTSLR(-)
(R)GNVLEGVITGITK(Y)
(R)GNYVSAIVK(R)
(R)KGWEIVK(E)
(R)LSLGLK(Q)
(R)NEHPVGTIVEGK(V)
(R)RGNVLEGVITGITK(Y)
(R)RLSLGLK(Q)
(K)AADKLEWAGK(K)
(K)AYYFYLAGNLR(E)
(K)GTIALEILEK(K)
(K)IELALTK(F)
(K)TGIVLEPK(V)
(K)KLPINFVGNVEGR(D)
(K)LIFNILNPK(V)
(R)FLYHSILDEEEPAADR(L)
(K)AYLNLSYDLLNR(N)
(K)ELFYLR(F)
(K)ETAYAGAEFISDKYPILR(V)
(K)ETNPGTPPNVVLIMLENWTGK(F)
(K)FISPISNGLVEGK(E)
(K)LGAWGYDDADVLQLLHER(I)
(K)NTIFVFVADHTHHR(Y)
(R)FFDGKEDLSYNINPPR(E)
(R)NLLAPER(T)
(K)AFELAGTLK(N)
(K)IIIYSSPSNPTGK(I)
(K)SEGAFYFFIK(I)
(R)KAFELAGTLK(N)
(K)NLITTADHGLLNYLR(R)
(R)VQKLMDLSFDLASR(S)
(K)ANNVLFYDFR(F)
(K)GIQQMPHTLR(E)
(K)GYQNLSDTAMHYIGGVLSHAK(S)
(K)GYQNLSDTAMHYIGGVLSHAK(S)
(K)IDPGPPREEDLFELSLDEIR(E)
(K)IDPGPPREEDLFELSLDEIREK(G)
(K)KGDVFTEEFIQTYK(A)
(K)NGVNLFAGK(G)
(K)RLLPGFEAPSILAYSAQNR(S)
(K)TLHQIGmETFVVHHEVAQGQGEIGVK(F)
(K)TLHQIGmETFVVHHEVAQGQGEIGVK(F)
(K)YLESSGLADTAYFGPENEFFIFDNIK(V)
(R)FTDIK(G)

(R)IPFVNGDK(A)
(R)LLPGFEAPSILAYSAQNR(S)
(R)TPSEVIAYAK(A)
(K)EIGVELAVLGNFRPSLGGSEYLK(K)
(K)FYPIGK(T)
(K)IESHNHPTAVEPYQGAATGVGGIMR(D)
(K)ISFQELSGPYEK(G)
(K)LIFNFPK(E)
(K)LLMEASLEAIQK(G)
(K)RPSYLDAVK(T)
(K)TVLFSGLGIESDLTSLRR(N)
(R)KPITEQYDSEVGLVK(L)
(K)EFVLHDGPPYANGNFHLGHSLNK(I)
(K)ETLEILFVVSQVHEK(N)
(K)FQHPFMDR(I)
(K)GIEIWYSEK(A)
(K)LSQFAEDVIANYEAYQFHQIYQK(L)
(K)REPAQIQSWK(S)
(K)SLEAGLEIVSK(N)
(K)SLSAIDGVQWIPSWGITR(I)
(R)ATPQWFFK(M)
(R)GWFQSSLWPSMALR(G)
(R)LWVSSLDFR(D)
(K)AISLSHISDGTEIADNGTVQFQTVR(N)
(K)ILVLISK(Y)
(K)VELFETLIAPGPFTHPTQLTPEQWK(D)
(R)TTALIWGEK(D)
(K)GIIIGGSMFR(K)
(K)HINRPVSNEQK(S)
(R)FGIVAGK(E)
(R)SLLSTSSGINWK(V)
(K)YKGFKSLTPISISEK(S)
(R)YLEVFGSFFR(M)
(K)TTLLGFFQPDLK(E)
(R)HAFFGELSLFTEER(R)
(K)FLLQEIK(D)
(K)FLLQEIKDEELR(L)
(K)QLLFNLISNAFK(Y)
(R)LHHTDQFEGTGVGLAIVK(R)
(K)DISVAWIGFSHLK(S)
(K)ILPLQLDSASIPEYDPEKK(T)
(K)TIDLMK(K)
(R)LIAYSLGNFMGYR(A)
(R)SFLFGKVESYLK(G)
(K)LIIEMQTIEEYK(S)
(K)SMFEEMWPK(A)
(R)FSILIDADVK(T)
(K)FLDANMR(K)

(K)LYNFEGLKR(N)
(K)MALGMNVIQLAAK(K)
(K)QGWAVHNLSESPGTNLDPLLDTVLK(H)
(K)SLQLGHRPILVINK(I)
(K)TTLLDGILR(Q)
(R)EGFEIQVSRPEVILK(T)
(R)GELVIGPNTSVYPGMIIGIHSR(D)
(R)GIIGFR(G)
(R)KFSLEQNIEFLDDDELLEVTPTSLR(L)
(R)KGELQLMDAHPSGMTR(V)
(R)NIAIIAHVDHGK(T)
(R)VEFVIPTR(G)
(K)GKPVPPNPNLAGFYISSETQR(W)
(K)IEVLSIFPK(S)
(K)SDPNVNKEELIR(E)
(R)TLIFSNILFTK(I)
(K)IPFGIAQIGK(S)
(K)KYVPFVVEPALGVNR(L)
(K)YNFGWGELWGIASR(T)
(K)YVPFVVEPALGVNR(L)
(R)KIPFGIAQIGK(S)
(R)LFLAVVTDAYEEEKLPDGETR(T)
(K)LHPIFWPDVK(I)
(R)ISMFVGR(T)
(K)ILIFLK(A)
(R)YREIILSIPK(E)
(K)SSSISYIEGYLWDGQGTK(E)
(R)IVQEVGPR(L)
(R)SGGSAANTMIALANSGGTGTYTGK(V)
(R)TMLTHLGISITLQK(S)
(K)ASFLKPEQR(K)
(K)LTVNSNNLDLLPK(N)
(K)NGIQVGYTGTYR(L)
(K)RDIFDEYNISPDK(G)
(R)LIVNSLNTYSASQFK(N)
(R)LPLFLK(T)
(K)AIALIHVK(Q)
(K)EFEEAISEFIIHR(S)
(K)SIDPSIKK(N)
(R)SFPIAHK(Y)
(K)AVAFQEALQPDYKK(Y)
(K)FAEIAK(E)
(K)FPMNQFR(L)
(K)GGIQEMTQK(F)
(K)GGIQEMTQKFPMNQFRLD(-)
(K)IYKPIPYGVDSK(T)
(K)LIVAGASAYAR(T)
(K)NAIPFDKNPPAVASGIR(L)

(K)NPPAVASGIR(L)
(K)TLAEVFVK(R)
(K)TLAEVFVKR(G)
(K)VKGGIQEMTQK(F)
(K)YAEGYPGKR(Y)
(K)YIETVLANAK(T)
(R)AVLEAYTSTLTNK(Y)
(R)GGLILSTLENEK(V)
(R)LGTPALTTR(G)
(R)VFPGIQGGPLMHVIAAK(A)
(R)VFPGIQGGPLMHVIAAK(A)
(R)VVSGGTDNHLVLLDVSVK(G)
(K)FSQDLSHVFAESLGLSK(I)
(K)DILWTISDHFPQIGTYR(M)
(K)NNFTAADLLSLGVR(R)
(K)GAYAVEVDIMQPIDVNK(S)
(K)KLETFWVDILGLEK(A)
(K)LETFWVDILGLEK(A)
(K)LNHIGLWVDDIHK(A)
(R)FTPGGIRK(G)
(R)SEKENVDEDILR(I)
(-)MESLHIQQTK(T)
(K)VIMDILDSLQK(Y)
(K)VLWLYK(E)
(K)AANILANYSSSFVK(H)
(K)YIPILDELHPAVVNLR(N)
(K)YLLVEAELAR(L)
(R)ESYIEALLNEFKR(M)
(R)GNPFFINELLK(Q)
(R)MTETGRPSIVLIAGYSGVGK(S)
(R)SEIPEALSHVILK(L)
(K)GAGLGFLEILR(K)
(K)SFLGIDIGAGSIK(A)
(R)ELIFPIFR(T)
(R)NLIHTINPEK(I)
(K)LSGGQIQMTAIAR(S)
(K)ALALAALGNYVDAR(V)
(K)ISDIFLR(D)
(R)FLSENSSLLNVISGK(D)
(K)GMVANSGSEPR(K)
(R)GEVAYVGSVLDSIKR(T)
(R)GVYVSSNLVLR(S)
(R)IKLENYGLTPAEIAGIER(G)
(K)GIQPDVIVKPIEPTEDDRFYIR(K)
(K)GSVQNIYSLSHNTGIALTIQK(Y)
(K)NPNYSEANFVLLEK(Y)
(R)GLISSLGDPHSR(F)
(K)NIPIKQYSSGMVMR(L)

(K)AGDLIAQLYAPFVLNGNPILR(M)
(K)EGAAIEDFMRPER(V)
(K)GADALLLLTEWR(E)
(K)GIIPIYEPGLSELVLNNWK(E)
(K)STVPVGTAAK(V)
(K)TFAVWGLSFKPGTDDmR(E)
(K)TIIANETKEEFDVVSNPEFLK(E)
(R)EAPSIPLLLK(L)
(R)VVIGSETQK(A)
)TLNGLDSLLSIVQMPGGVPVGTLAIGTAGAK(N)
(K)FLQTIPEGSIVLAK(A)
(K)LFYPNTGEYSK(I)
(K)TQEVSTNVPKEETNVYKEEPK(K)
(K)ILYFLQANLTGENTK(H)
(K)LSMGMSGDYK(I)
(K)NTSLVLHHIGPVQSGTLRK(L)
(K)AGIPLPGFAPVWSLSDTR(D)
(K)MLTFQDNINALR(E)
(R)DALEFLNFPLVMKPADNMGAR(G)
(R)IIEREPYFIEMGHNMPSALSPSILK(E)
(R)KIEGVNDLFFMNK(I)
(R)LSGGFMSAFTFPLSSGINLNR(A)
(K)IAQHLLR(L)
(K)LLLHGEGIK(I)
(K)SSEDHFTIAYNSAPYQFR(Y)
(K)VEAGNTTTEVGDKVELFK(K)
(R)ILLEYASPLIVR(S)
(R)KFALLAMIASK(T)
(K)NTMSNYVGIVR(S)
(R)ETILATGGAGQVYSHTTNPK(I)
(K)LSVNINK(I)
(R)FLDLVLK(T)
(K)TSLITELDPTRFEILSFDSR(Q)
(R)VLSMSIEEKK(V)
(K)DKLDLTIYLLNGVPLK(G)
(K)IIPSEPVDIAIMEKSSR(I)
(K)NTAPIIALASLYFQER(Y)
(K)SILIQEK(N)
(R)TSIILEEFK(T)
(R)YGDPLQVVLSADAWIHPVK(E)
(K)TYFAGIMDIR(G)
(K)VANVVLFPPESINPPPPSVK(G)
(R)HMPILENDTLIGIISIGDAVK(A)
(K)LGILYLNNNQLTTLPK(E)
(K)LTSIPDELGQLKK(L)
(K)DGVALLNAIQDVYLSK(D)
(K)IDYHLATDLTGQANHLGATIQADIVK(Q)
(K)INHNELLSYPNK(F)

(K)IPFIVK(I)
(K)LPEVFAGGFK(D)
(K)QKLPEVFAGGFK(D)
(K)SMKDGVALLNAIQDVYLSK(D)
(K)TALGAEADSLLNHTCK(T)
(R)AGGMGLISGR(K)
(R)AHELGLVTILWAYLR(N)
(R)MYTQLTADNPIDMAR(Y)
(R)NYQAILNTGR(L)
(K)ITIEQR(L)
(K)IYNGTIAGGSNR(L)
(K)FIPSSTISLSQPIENR(V)
(K)NTAVTTAGDGIPLLLGQIAEVEIGPALR(F)
(R)VLGLPIVEIK(V)
(R)YGVQAEEILATVEALR(L)
(K)SFLHFNQNLQTPVVTK(Q)
(R)IHFRDSR(E)
(K)APIAALEYPSFGNGILIAGGLSNVIR(K)
(K)NNIVGLAPNTLDR(E)
(K)DVQVQLEKDQLIISGK(T)
(K)NGVLNLTLPK(R)
(R)TFTLTESVEEDRISAVYK(N)
(R)VLELPTEVDSEKANAEFK(N)
(K)EVVGYQGLLTFDLTKPDGTPR(K)
(K)NLPEVVVWGTGKPLR(E)
(K)VNEFFEVNRPEYVFLAAAK(V)
(R)GYELSLEFFNR(F)
(R)LFFDNKPITDFVIK(K)
(R)NSGVLHSGIYYAENSQK(L)
(R)NVFFNSLK(N)
(-)MLDHYNAYIGR(L)
(K)ANKGDVLIVLSGSGNSPNVVK(A)
(K)GDVLIVLSGSGNSPNVVK(A)
(K)FPALSLR(F)
(K)ISIETGIGELLK(N)
(K)LAGKPFTVVGDGK(Q)
(K)NIDYWR(E)
(K)VGEIYNVGSGATISVNR(I)
(R)AFNLNHVK(E)
(R)FFNVYGPR(S)
(R)IVELLK(G)
(R)IVELLKGEVTYIPK(R)
(R)MGEELVMHWAQVYK(F)
(R)TSGTYGAVFGVFLAQK(L)
R)TSGTYGAVFGVFLAQKLAGKPFTVVGDGK(Q)
(K)AFDSGLLPNSLTDEKYFNIK(R)
(K)LVTTPTNDNR(S)
(K)SINLDAFRPLVEISK(K)

(K)SINLDAFRPLVEISKK(H)
(K)SIYITGGAGYVGAMLVPR(L)
(K)VAGEIFNAGYLNFTVSEIANMVK(E)
(K)VTVLDLMIYGEDVLK(E)
(K)VTVLDLMIYGEDVLKEHPNLTK(I)
(R)FIYASSSSVYGIK(D)
(R)LDVVVNILTNLAYHK(R)
(R)LLSEGYK(V)
(K)SVFTPYGDK(K)
(K)SVFTPYGDKK(L)
(K)YGEVLLFDQGYPHGNR(V)
(R)DILGLSKEETSDILLNQIHK(K)
(R)LKVIQSmNSLK(D)
(K)EYGFDYWDGDRR(F)
(K)GFLLYEMLLIEPK(L)
(K)LFELK(S)
(K)NGYLMLESYR(N)
(K)NIVTPLHK(A)
(K)TLIEDYNLRPGSK(V)
(K)ILEQGSFAQLSVKPEGILEK(M)
(K)QNTETGNIAMGVLSEIVSSAR(I)
(R)IGYVPQDPVLFHSSIR(E)
(R)KPELLILDEATSSLDSESETLIQR(S)
(-)MIGLSIVIPVYK(G)
(K)FTNWAANFLLDKPK(N)
(K)VIFFEQENR(-)
(R)LIRAVTWNFPNmGLVGYK(D)
(K)LAEILLLDKV(-)
(-)MILITGADGFIGSHLTETLVR(Q)
(K)VIHTSTSEVYGTAR(F)
(R)AVIPTIITQIAK(G)
(R)FVPITEEHPLQGQSPYSASK(I)
(-)MLSEQIVSVLR(S)
(R)ISAIVPMHTFGHPVDIDGLFAVAK(D)
(K)LDLQIIATGMHLSPEFGLTYK(E)
(K)NNLLITFHPVTLEESTAK(V)
(R)IIFELVK(E)
(K)GLAISVNEVMSSKPK(V)
(K)QINIFPIHEYWLDIGR(M)
(K)ASFIPEIWQGHK(N)
(K)FIFSRPWGLPVR(Y)
(K)LESYGIPAYK(I)
(K)NNGEGFLISLTR(L)
(K)VEMDLVEFHFSYK(D)
(K)VSLLPGEFR(E)
(R)ISIIPLINENGILEAIAK(E)
(R)SLIEIQK(V)
(R)SLIEIQKVIDITR(R)

(R)YHDLEQILSK(V)
(K)AIVVVHLYGMIIPDILEIAK(F)
(K)FINSFNEFEELSFQK(Q)
(K)IQVIVQYYPLYR(Y)
(K)NITTMGEGGILTLK(N)
(K)NVSLADKVPGLR(H)
(K)TKAIVVVHLYGmIIPDILEIAK(F)
(R)IALLTEER(R)
(K)AIVGIGGGSTMDVAK(A)
(K)FGINSDHSMFDAIILDSSLIK(N)
(K)IGIPTVAGSGAEASR(T)
(K)IHKIELPK(N)
(K)KNNGDYYIFIIDDVHQK(G)
(K)MERPLTNALGENWK(D)
(R)INELLPK(K)
(K)KYNTPSVSFQGDNEPFLNK(E)
(K)LSLVDLWNSK(E)
(K)MTTESITK(I)
(K)VDFVVIQDFMTPDVEGDFESLAGK(G)
(R)FSLDAINEDTYKK(I)
(R)IKGPVFEEYR(K)
(R)KELNSELPIVGVNFVR(M)
(R)YPLSNHLYWLSK(G)
(K)AFQHLTGR(G)
(K)ALDILR(N)
(K)IGSSELDGAAIASFSSPIDDIMLFK(S)
(K)KALDILR(N)
(K)KFEIIENPENKR(I)
(K)SRDFWMPFAPVVMDTFVER(Y)
(K)SVLEIFPEK(K)
(K)VMGLAPYGK(E)
(K)YDTDREQILAEFLSIDR(T)
(R)DFWMPFAPVVMDTFVER(Y)
(R)LYLGPSASLK(E)
(R)LYLGPSASLKEEER(V)
(R)NKPILALTVDGWGDGK(N)
(R)SILADPIHLK(I)
(R)WKPFLVNGENALK(S)
(R)YMTLMLGMKPNEHEFK(V)
(K)DISIPIMNQGHR(G)
(K)NVESYSIVGGVPAK(L)
(R)GVGYSNANLFGK(Y)
(R)VIQIENIK(A)
(K)NWDLLYASR(E)
(K)QLGVIAFR(N)
(K)TIAVLPAR(M)
(K)VVVDKNWDLLYASR(E)
(R)EIAGGGILLDQGIHMVDLIR(L)

(K)IIQYLSK(N)
(K)IQFLR(F)
(K)TLNSFVSEFQK(Y)
(K)LTPLEVSEFAK(E)
(K)MILINDLK(D)
(R)FTEKEEIYSSSDIVSLHIPLSHK(T)
(R)ILSEFRPK(M)
(R)KLTPLEVSEFAK(E)
(R)VGLNVIR(I)
(K)ETFMVTYGDGVANVDIKK(L)
(-)MFQNIYK(N)
(R)SPLATRPWQHVLEPLSGYLLLGEK(L)
(R)FLFIPEVLGEYTIHNSNQSAALER(H)
(K)ILAGSHIPLR(I)
(K)ALLELLDKPHHPVEIIGTR(H)
(K)ASAATIEVLSK(A)
(K)AVYPINAMGISK(A)
(K)FFVGDVR(D)
(K)ISTLEDYNSHNTKR(L)
(K)KISTLEDYNSHNTK(R)
(K)SGKPITITDPNMTR(F)
(K)SLLITGGTGSFGK(A)
(R)GSVIPLFIEQVK(S)
(R)GVEYVFHAAALK(Q)
(R)YGNVMASR(G)
(K)KTPILFSSSIQAALDNEYGK(S)
(K)TPILFSSSIQAALDNEYGK(S)
(K)TQDAGQFSFFTAHPGVTR(G)
(R)GVFVEMLK(T)
(R)KDLVIPNVGTGLIR(A)
(R)LQSENGNPVFIFR(L)
(K)IPIFHMEAGNR(C)
(K)TGSPMFEVLNYYLEGIK(K)
(K)TGSPMFEVLNYYLEGIKK(S)
(K)VSTIIGTRPEIIR(L)
(K)YFVVSAHR(E)
(R)IIHSYTDYVNR(V)
(R)IVDHTADINLTYSNIAR(E)
(R)KPDYFLNAAGTSGAATIGNVIIK(V)
(R)VLQVLQILEK(Q)
(K)GDMSFIGPRPESMELSEWYEK(D)
(K)VVFSQKR(M)
(R)FALVPLGEALEFR(E)
(K)SASVITDYLFK(F)
(K)SIISGVTELVTK(H)
(K)FSPPIFWDLLK(F)
(K)GMIFELK(K)
(K)INVFLASSSFPYIK(D)

(K)NLSWNIVEQPNFVQAGK(K)
(R)DGNLIILESLKDIPFEIK(R)
(R)VYYINNLENSVSVR(G)
(K)LLETIESGWYILGK(E)
(K)TEVHYPIPPHK(Q)
(K)TGTFGEMSGFSFYPTK(N)
(K)YKNDLVGYNSR(L)
(R)LYTENIEVLFK(R)
(R)SIETYLPLYLQLFK(N)
(K)IVSGNYPFTYHVFR(K)
(R)LVSNPFSEFIQTISDYK(I)
(K)KFQIEGPVLIEPK(V)
(K)NIFWVPPGFAHGFLTLEDK(T)
(K)VIDVVVDVR(V)
(R)GFFLETFK(A)
(R)GMHLQIPPYDQGK(L)
(K)IFGVVPHWR(E)
(K)GIILAGGSGTR(L)
(R)AVSIEEKPSKPK(S)
(R)IYLERGILNVQVMGR(G)
(R)LYPVTYVVSK(Q)
(K)FEYFHFPENLGYGVANNR(S)
(K)KLPEVLEPLNLNLK(G)
(R)IHSVTAIAEQTHYATATR(S)
(R)MINLETPIVASK(T)
(R)SGVAPFLFPSLR(A)
(R)VELVPIFGK(F)
(K)EFLINQNILR(S)
(K)HEEQGIFHHYK(C)
(K)HSVSVTAEEDWGQRK(L)
(K)LWHPIK(H)
(K)VEKEFLINQNILR(S)
(R)NYELTTITR(V)
(K)SINGTSLVNFSLANGR(T)
(R)IVVENFQLLGSK(D)
(R)ITGTSARYQRVLAR(E)
(R)SERPEQGDLSAEMEGKPQRK(Q)
(R)SIGLLPYK(V)
(K)KLGELKR(E)
(K)LFGAVTPIDVASILK(K)
(K)LFGAVTPIDVASILKK(N)
(R)LAEGIQPTITLHVK(K)
(R)NFLFPK(R)
(K)EVFNIQLSIPFPR(M)
(K)HGTEGLESTITKR(F)
(K)HWIQENYK(N)
(K)SIRDVIAFPK(T)
(K)TPPFSLDEFDEVSEELKLK(Y)

(K)VRSEYVLAVR(G)
(R)EIVNHSLGALR(L)
(R)FRDQGGVIFIDLR(D)
(R)HEFIFAIR(N)
(R)LNPNQFYALPQSPQIFK(Q)
(R)MLMLLTGGK(S)
(R)MQTGTIEVVLDQLEILNVAK(T)
(R)MTYNTAMEEYGSDKPDLR(F)
(R)SEYVLAVR(G)
(K)IPNAGFFVENQHLIPK(K)
(K)LMELADTGHPLLQDLLTK(A)
(K)IIILSTGSELTSGR(S)
(K)VGLVYFGIGQK(N)
(R)ALVDFLLK(K)
(K)GEEIFSFDLDTIYPHK(L)
(K)NASVVLETK(K)
(K)TLEVLSSQIR(E)
(K)VATPSQAQEVHSFIR(K)
(K)VSMVFPSTLHLENVSK(I)
(K)AGVAGIDLQLPVDHIIGDQFNEK(A)
(K)EILELSALLHKPARPFVAIIGGSK(V)
(K)FASGTMAIAK(A)
(K)GQVNPEFSLAPVVETFQSLVK(S)
(K)ITHISTGGGASLEFMEGR(K)
(K)MGILDGWMGMDIGSK(T)
(K)NAGTIFWNGPMGVFEMDK(F)
K)NAGTIFWNGPMGVFEMDKFASGTMAIAK(A)
(K)TLPTIELLIK(K)
(K)TLPTIELLIKK(G)
(K)VNHLLIGGGMAYTFLK(S)
(R)AIPIGNSLVEK(E)
(R)AIPIGNSLVEKEFEVQAFQLIEK(A)
(R)IIIASHLGRPK(G)
(R)KLPGVEALK(K)
(R)KLPGVEALKK(K)
(R)LLPAYAGLLMHK(E)
(K)DKDIPTFVMGVNNEK(Y)
(K)DPNLEFVAINDLVTPDNLAYLLK(Y)
(K)KVVISAPAK(D)
(K)LTGMSFR(V)
(K)LVSWYDNEMGYSNR(V)
(K)VDYVIESTGLFTDR(V)
(K)VVISAPAK(D)
(R)AGIKDPNLEFVAINDLVTPDNLAYLLK(Y)
(R)FQGTVEHTEKELIVDGK(K)
(R)GAmQNIIPASTGAAK(A)
(R)GAmQNIIPASTGAAK(A)
(R)IAINGFGR(I)

(R)VLDLIR(Y)
(K)KSEIPAILSLIEK(G)
(K)LPLTSSSVTINSPYMHLVR(F)
(K)SEIPAILSLIEK(G)
(R)GYAATPGDSNVHISTIYAVFR(G)
(R)IFSHAVIPLGSLEVLK(N)
(R)LLMEESLQELESILK(E)
(R)LLNELYSQVGIK(L)
(R)VKDFFPEFLSK(K)
(K)AWMPQQFENEANIK(I)
(K)AWMPQQFENEANIK(I)
(K)DFPDGLDYVITGVGTGGHITGVAQVLK(Q)
(K)DSIIVEPTSGNTGIGLALVAAVK(G)
(K)EEGLFIGVSSGAALAAVAK(K)
(K)GMPGAIEK(A)
(K)KLPEIPEGSR(I)
(K)LLLVMPESmSIER(R)
(K)LLLVMPESmSIER(R)
(K)QIVAENPK(A)
(K)SILETIGNTPHVK(I)
(R)AAKEEGLFIGVSSGAALAAVAK(K)
(R)IALSMIEDAEK(T)
(R)IALSMIEDAEKTGK(L)
(R)ILTFSYDTGER(Y)
(R)IMAAYGAEFELTPR(E)
(R)IMAAYGAEFELTPR(E)
(R)YLSIEGLFPVPSNA(-)
(K)ILFLR(S)
(K)KDQLYNTLSSGEK(K)
(K)ASFFTIDETILHK(Y)
(K)FILLSLNQK(L)
(K)VNTIVQAVK(V)
(R)ATLIVPQQIVPIEITTK(L)
(R)IFFLK(K)
(R)NKVNTIVQAVK(V)
(R)NLLYVSGVR(K)
(R)VADIAAAGALLHDLGK(F)
(K)AVWGEEK(Y)
(K)KDQVLISNFVDQTEEER(K)
(K)NISFFR(R)
(K)VVVLIFNPLK(E)
(K)VVVLIFNPLKEK(N)
(K)WRGFDLENNTFVINFQNLmPQK(L)
(R)FIDIGGIK(L)
(R)FIDIGGIKLLN(-)
(R)GFDLENNTFVINFQNLMPQK(L)
(R)GFDLENNTFVINFQNLMPQKLEPK(A)
(K)EIFLTNEEGIVLAHSNPEYLVENLK(K)

(K)SFFFSK(F)
(K)TETNLSQITIK(T)
(K)INPISNPTSPEYSAK(S)
(R)GIFIPEIK(T)
(R)NSPVQFYSSSSSR(K)
(R)ALLVEGGGmK(G)
(R)ATSSNIFDLLK(A)
(R)IHTISPENPLPVSLVTTNANK(L)
(K)VLYNGNMLVIFPEQTTVPYK(M)
(K)GAFSFITKPFQPEILINK(V)
(K)GYPFEVLIK(S)
(R)LESITLR(V)
(R)YSVVIPFVFYHGER(T)
(K)IMHIALPIGK(Y)
(K)IVISAESKEEADK(L)
(R)DKYGIEWMVDFDPK(Y)
(R)FKDLASPEFPVAK(K)
(K)GIVNLLELK(S)
(K)SGITLYDSNVVHHHHAIDEK(T)
(K)IVLIHGWDFQEK(N)
(-)MIEIQFYNSLSGR(K)
(K)SIIEFTAPWTK(A)
(K)TWLHSEHLLVDGQK(M)
(R)AFLFVDVLR(R)
(R)ILGVLNFESK(K)
(K)GLTETGVAQLILDLR(G)
(K)IKVPTLVSVGMEDR(I)
(K)RGDLPVVVYFHDYGK(D)
(K)SMGASLAAFGAAFTNR(I)
(R)AVEFLR(L)
(R)GDLPVVVYFHDYGK(D)
(R)MQVYPTEGNEAGFKDEK(Q)
(-)MNPVLLDGKK(L)
(K)LATILVGNNPASETYVSMK(V)
(K)NAVVVGR(S)
(R)NADIIIGAVGKPEFIK(A)
(R)SSFYTPVPGGVGPMTIAVLLLQTLYSAK(E)
(K)ALEILQSK(G)
(K)EHGIDFLISQR(H)
(K)VLERDPAPLLAYLEK(D)
(K)VLERDPAPLLAYLEKDFPVIDYTK(A)
(K)YGSVPHSGFGLGSER(L)
(R)EQLLTVEFGGPVFIQK(Y)
(R)HLWLR(S)
(K)GSSVLTPLLEAYLLK(E)
(K)TVLDLISVYK(I)
(K)ESALVLEMLAGR(L)
(K)LLQQIWLPNK(N)

(K)NALLYVPK(E)
(R)IILDEAHNFPDIIGSSFR(R)
(K)ARWDLEK(S)
(K)ITLENR(K)
(K)LLLMEDVLK(T)
(K)LVDLQK(E)
(K)NNPVLIGEPGVGK(T)
(K)NTVIILTSNIGSEVLGSSEYTSEEKER(L)
(K)TAIVEGLANK(I)
(K)TLYTLDLGSMIAGAK(Y)
(K)VIGQDHALR(L)
(R)AGIADPNRPIGTFLFLGPTGVGK(T)
(R)FLPDKAVDLIDEASSK(M)
(R)IDEVILFHSITDSVIHK(I)
(R)IDMSEYMEAHSVAR(L)
(R)ILDSALIAAATLSNR(Y)
(R)LLKEEVSEEDIANIVSR(W)
(R)RPYSLILFDEIEK(A)
(R)WTGIPVSK(M)
(K)ADPDKPQVNEER(A)
(K)DLIAEFNKIAPILQK(R)
(K)FIKDLTSETILFEDQK(G)
(K)FSFKDLIAEFNK(I)
(K)FYSELAVNER(A)
(K)GSPEEDKNDQLSFYAQIYLPIR(I)
(K)IAETNNQNTNIVTK(D)
(K)IAETNNQNTNIVTKDEYDR(I)
(K)IVEEIGIQKIAETNNQNTNIVTK(D)
(K)KQSIFQDYISK(L)
(K)LITSGMLEFAFGR(I)
(K)LYAYSKPSSTNPNER(I)
(K)QSIFQDYISK(L)
(K)TLFQEIQK(L)
(K)TLPLLGR(S)
(R)ANQTAEHYGNR(F)
(R)IKQELVESK(K)
(R)KGSPEEDKNDQLSFYAQIYLPIR(I)
(R)SVLFMLIR(N)
(-)MILNEIIISTEK(I)
(K)ATVITPNEYVGNIMSLAMDK(R)
(K)ATVITPNEYVGNIMSLAMDKR(G)
(K)MDILVNGEPVDALSMIVHR(S)
(K)QIGNVEIPQEAFLAVLK(T)
(R)HQFMIPLQAAVGGK(I)
(R)VGYLGLLHMEIVQER(L)
(K)AFPVLK(D)
(K)AFPVLKDLVVDR(S)
(K)ITHLGLLPQGK(V)

(R)IIQAGGFISVNTGGAPDANALPIPK(V)
(K)GQTGQQLLLGAYSALSR(Q)
(K)IREEFWQNVNVPGSGTELNQSLEK(A)
(K)LEFENVHLATR(S)
(K)LTLMSESLR(N)
(K)YPSYGNLSPR(D)
(R)AHSIAAQGGINAAK(N)
(R)DRYENLFQMYEQITGENPYK(Q)
(R)KFNIIVVGSGLAGASASATLAELGYNVK(T)
(R)KYPSYGNLSPR(D)
(R)NPADIPESERDYYLER(K)
(R)TEMVELIVVDGHAK(G)
(R)YENLFQMYEQITGENPYK(Q)
(R)YENLFQMYEQITGENPYKQPMR(I)
(K)NTVQATLASR(T)
(K)SEFLNPYFSFR(G)
(R)LGLETVDVFLLHNPEYFLMDR(E)
(R)TVTEPYLNQFQNQNHLNQFLER(T)
(K)DKIQVLLLENVHQDAFDMFQK(D)
(K)ELLDEIENIHVLGIR(S)
(K)FINNGSTTFSVNFPNLEITPLPAGQYR(I)
(K)GIPVFNAPYSNTR(S)
(K)HPFSIK(T)
(K)IQVLLLENVHQDAFDMFQK(D)
(K)KGIPVFNAPYSNTR(S)
(K)NSDFISFHVPETPQTMNLYGK(K)
(K)TNVTSAVLEK(A)
(K)VIYYDIQTVLPLGNATPVSSYEELLK(N)
(R)MISFPK(D)
(R)RVPDHIR(N)
(R)SKTNVTSAVLEK(A)
(K)AATLDPAHYLASMELGENAIR(L)
(K)GLLELIK(I)
(K)YLNLYGIEYTR(V)
(K)FLTSPGK(E)
(-)MNIYIGNLAYQATEDDLR(K)
(-)MNIYIGNLAYQATEDDLRK(A)
(K)AFESFGEVTSVR(I)
(K)VNEALPK(K)
(R)GLAFVEMANK(E)
(R)GLAFVEMANKEEGNAAIDGLNGTQIR(G)
(K)ILNLDVSLFPR(M)
(K)VLETSALLAITR(K)
(K)IPEFLPVQR(L)
(K)NIFDFR(A)
(R)LEPYYQIALSVNAIK(M)
(K)KLNVDDNQDTAQSLGISSIPTLLLYK(D)
(K)VIGALPK(A)

(R)MVAPVLEELSGELDGLVK(I)
(K)AEMAYFR(G)
(K)ELQELFHFQPIIIGK(A)
(K)FEGLNQDETGQGPIKR(Y)
(K)FFHVEK(G)
(K)FLQFIR(N)
(K)FYIIASGNVK(F)
(K)GISTAELEFPIYYNFFLR(Q)
(K)IEVPGEVGFNIK(Y)
(K)ITIHATETVMDSFLR(K)
(K)ITVYHIAR(K)
(K)KELQELFHFQPIIIGK(A)
(K)KNPGVYMR(M)
(K)KSPLVFILPNK(F)
(K)MYAQGILPESR(W)
(K)NFVSNYTFR(A)
(K)VHYGNVIIGK(L)
(K)VNEGSILVR(E)
(K)VNVYQNGNLLAELTDGDFVGEIYNISK(N)
(R)GLTSHQITQLELIMTLHK(V)
(R)IIYHEAGIPPLHTR(I)
(R)ISYLASLPPEVQEK(I)
(R)KIEVPGEVGFNIK(Y)
(K)FFETEVAPNHESWEK(V)
(K)ITLETIAGR(S)
(K)TFISNGQLANLIITAVK(H)
(R)IQTIYAGTTEIMK(E)
(R)KFFETEVAPNHESWEK(V)
(K)ISDISLTNVGFAVFLK(T)
(K)ISIEEIIDNTFYAK(I)
(K)TQLEILQESLNNALR(T)
(R)ANAPIYLAK(K)
(K)FLLGLHSSLR(T)
(K)FVALTFTNNQPVFNYAVFEAIQDK(N)
(K)GNPIGMIWGIR(R)
(K)IVASATHFK(M)
(K)IVVMGHSMGGMIPR(Y)
(R)ALPSSNNQIVK(F)
(R)HLLSGTMNYAAGIPLFVK(G)
(R)NFQLPIYSVLGTLDK(I)
(R)TVSFELSTK(V)
(K)AANTLNAASDK(F)
(K)AANTLNAASDKFAK(E)
(K)NASVQALETYLNIVSK(D)
(K)SSIPVFDLVEFK(E)
(K)AVPILFEILK(T)
(K)HLTDTGDMSVLEILEYK(A)
(K)LTQYEESILK(L)

(K)SISSMQTIKEELEK(I)
(R)AQIALFFGK(N)
(R)ELEGFPPEHSGILIDQLGK(I)
(K)IMLTGQAGLDSAIHAINYGGLSR(Y)
(R)YVEKPWNIEELTGDIR(F)
(K)GISSGVGNSILIK(V)
(K)LGANAILGTSLAVAK(A)
(K)VNQIGSLSETLSSIEMAK(K)
(K)YVLGAENNK(E)
(K)YVLGAENNKEFSSAELVDYYANLVSK(Y)
(R)GNPTVEVDVILLDGSFGR(A)
(R)MGAEIFHSLK(S)
(R)SGETEDVTISHIAVATNAGQIK(T)
(R)YIGGNFAR(E)
(K)INLSFIVLPPEK(-)
(R)ETPGFLIR(F)
(R)LSSFPYTNSIFPAEVEFR(F)
(K)AGMNTYLEALR(N)
(K)ELATLLLEQGHTVTLVAR(R)
(K)GLDEIYYASGVMHNIKPDEFDVEK(D)
(K)GLFWLISAK(E)
(K)IIGISSIAGDR(G)
(K)IIVVGASSGIGK(E)
(K)SILASFNSSGK(M)
(R)LSVLGVQVLTVKPGFIDTAMTK(G)
(K)KVPIEIQLPEPTK(V)
(K)NIFSYADQK(G)
(K)VEAWGMNHHSISPVLFPEKEEDFK(N)
(R)GIITWLAVFK(K)
(K)NLVILETSWK(Y)
(K)YLNSEELESLFMR(S)
(R)ASSVTSLGDLWNWMK(D)
(K)GAEFVIGSR(L)
(R)NSFQLLDLEK(E)
(K)ILFGAIKPYTEIQNEFQSR(K)
(K)ILNFTLK(T)
(K)ERFPIVNISTGGLK(V)
(R)IVPPEGTVWITNIR(T)
(K)LIGIVTNR(D)
(K)SIPEIHK(A)
(K)VAQGVSGSVVDR(G)
(K)VMTTNLITGK(E)
(R)AQAENLIR(A)
(R)AQATAIYQTAK(H)
(R)DFLVLPGFIDFHPSEVELETR(L)
(R)GSILNFIPYLSQGLR(L)
(R)KGFTGIPVTEDGTR(N)
(R)RSESAQAQGSVHGLYSFSAPTmR(A)

(R)RSESAQAQGSVHGLYSFSAPTmR(A)
(R)SESAQAQGSVHGLYSFSAPTMR(A)
(K)LKPEFFQNAIWIIGPEGGFR(E)
(R)IFLPEIVSPVSLK(E)
(K)SASINELEFIVK(S)
(R)NTLFKSK(L)
(K)ATMILAEGAGLQWAATR(L)
(K)GLIWLWR(I)
(R)ISPIALMMDLVR(L)
(K)EYSGIDFSQIISLEEAKK(K)
(K)GEITLHVTSVELLAK(C)
(K)QGINPYPVR(F)
(K)SLDLGDIIGLEGYLFK(T)
(R)FEPYVAGR(E)
(R)IAPELYLK(R)
(R)LVMLLTNSHSIR(D)
(R)TTYVDIIK(E)
(K)LEPYPTGAK(N)
(R)TFYQILPGK(-)
(K)SALLLLSLSK(E)
(R)NHFLSAMSR(N)
(K)MNYMSAGQFPIPIVFR(G)
(R)ALMYVLPAAEK(L)
(R)LAAQHSQSFESWYAHIPGLK(V)
(K)ALMAELFGK(R)
(R)DHVSVAVER(A)
(R)RGEGPTLMEISTYR(F)
(R)TGISSGYGGSMHFFDK(N)
(R)VLGLELGADDYMVKPFSPR(E)
(-)MIINHNLAAINSHR(V)
(K)FQNNEVAK(N)
(K)GLMVAYENIQASESR(I)
(K)GLMVAYENIQASESR(I)
(K)MALLQGDFAR(G)
(K)NMETLSSGMR(I)
(K)NQILVQSGTAMLAQANVRPQSVLQLLR(-)
(K)NQILVQSGTAMLAQANVRPQSVLQLLR(-)
(K)SLNLIK(A)
(R)AGDDASGLAVSEKMR(T)
(R)ANLGAYFNR(L)
(R)IRDTDMAEETVAFTK(N)
(R)LEHASKGLMVAYENIQASESR(I)
(R)NTEDGMSLIQTTEGYLQETNDIIQR(I)
(R)VLAIQSSNGIYSAEDR(Q)
(-)MIINHNLSAVNAHR(S)
(K)GLMGAYENMQASESR(I)
(K)LFEGQFAR(G)
(K)QILVQSGTAMLAQANMKPNSVLK(L)

(R)IRDADMAEEVVSLTTK(Q)
(R)VLAIQTSNGIYSNEDR(Q)
(K)AISEWAASVSK(K)
(K)ASDGESLGSSLLSKTDAFVK(S)
(K)DSLFQEELVYKK(D)
(K)EEWFNLTENNPIVLKFTGLNADEATK(F)
(K)FKDDLTEFTATK(E)
(K)KKEFYEK(E)
(K)KVNNQLKEEWFNLTENNPIVLK(F)
(K)VGGAPVFPATPGNFGEAEIIK(A)
(K)VGGAPVFPATPGNFGEAEIIKALK(K)
(K)VNNQLKEEWFNLTENNPIVLK(F)
(K)VTVIGEAPIYNGDKQIAK(Q)
(K)VVLLWGDGK(I)
(K)VVLLWGDGKIVDSGTADGR(G)
(R)GFSFLATK(S)
(R)GFSFLATKSLEVK(S)
(R)GVVTLEFKPLK(-)
(R)TIYNTAATGTYK(V)
(R)VAVLVLGK(V)
R)VAVLVLGKVGGAPVFPATPGNFGEAEIIK(A)
(R)YPIQSYPSK(I)
(K)ILEILK(C)
(K)FLGHTVYQK(K)
(K)NLIDFR(K)
(R)GELTYHAKPQLK(N)
(K)LLLDLLR(L)
(K)EHYEWANFVIAR(S)
(K)IGYENLYLYSLVR(N)
(K)TFFIFK(K)
(K)YFELNGAAIVVDQKDEDESHLFR(V)
(R)VLTGSALYEEVSKK(S)
(K)ELILPIK(G)
(R)DLLSGHYLQYNIGFK(T)
(K)LETLGFPQFLKPVEGGSSVSVYK(I)
(R)ANQIFLQSGQK(V)
(K)VIVAFPNFGHWNVR(W)
(K)GSSGPLSIHPETSTPYGSR(F)
(K)DLYGAILGFGIK(G)
(K)FEPFGGVNIAYIIK(A)
(K)FIDGLELFSLLANVGDAK(S)
(K)FLGGHGTSIGGIIVDSGK(F)
(K)FWEVFGK(F)
(K)HSENALAVAEYLTK(H)
(K)KDLYGAILGFGIK(G)
(K)KFIDGLELFSLLANVGDAK(S)
(K)LFGLQEFGNIYTR(I)
(K)VHFVDPSDPENFR(K)

(K)VHFVDPSDPENFRK(A)
(K)VSWVNYPGLK(M)
(R)AFYAETLGNPK(L)
(R)AVPIYQTTSYVFK(N)
(R)KHSENALAVAEYLTK(H)
(R)NYKPETIALHGGQSPDPSTLSR(A)
(K)DIYVQIKPEYR(N)
(K)YTPGEITYSAYLGK(L)
(R)LINNIWFGQYGK(Y)
(K)DNYPEYGRPETPAEK(G)
(K)EFYIK(H)
(K)IFDYNDQLGNENYKENVDALKDSLR(Y)
(K)IFELNQR(L)
(K)NVQGALAK(Y)
(K)SQPSSAQLGQSILETER(K)
(K)SQPSSAQLGQSILETERK(L)
(K)YILAGVENTK(T)
(K)YMEIYFEGQNSNDPEPR(T)
(R)GPNTQPSHNDKVEVFFQK(D)
(R)HTVLMK(M)
(R)TVLFK(G)
(R)VEVYDFIRDEER(G)
(R)VLPFR(T)
(K)KTEIVLPLNLANFR(S)
(K)SILEAMEK(E)
(K)TEIVLPLNLANFR(S)
(K)VNRYEIVNITPPK(S)
(R)YEIVNITPPK(S)
(K)ALEIELIAAAK(A)
(K)TTILPAELANIAGVFEGLSK(V)
(K)KLSVLEILFR(T)
(K)KPFYDGLTFHR(V)
(K)LSVLEILFR(T)
(K)TVQNFIDLAQGEKEFLSR(N)
(K)VLIVDK(R)
(K)VLIVDKR(N)
(K)VVANELQIK(N)
(K)VVANELQIKNR(E)
(R)IAEMIIEDAK(G)
(R)SIPNPTTLAVINMDPLR(G)
(K)GNAIWVGENPHDYISIQGK(G)
(K)LSVAIITYNEEK(N)
(R)FFLLGQDVRYFTEREWQNVR(G)
(K)DIILAIIGK(I)
(K)GVEWDLAVK(K)
(K)IFTAAGFEWR(N)
(K)IGTAGATGYVIEYR(G)
(K)VQAIVVPGSGR(V)

(K)YMDLK(S)
(R)GSAIQALSMEAR(M)
(R)HLIHEVTSPQAFEGLK(L)
(R)THLVGPEMAAAAAIEGHFVDIR(N)
(R)TVILHADEIAPMVTWGTSPSQVVSIK(G)
(K)GASILITR(D)
(K)IYYFEVDSFR(K)
(K)NGMLPVILK(S)
(K)SEEVEELFHLVSTNVGAK(V)
(R)EHAPWALEDYGFR(A)
(R)TGFGVHLFHDWR(Y)
(R)YKGASILITR(D)
(K)DLQTFGAHVEIVPGAK(G)
(K)ETILQMIGNTPLIR(L)
(K)NTIILNEYK(D)
(R)ELSVAKPVK(E)
(R)IVVLSPDRF(-)
(R)LNQIGSHIPNVEFYLK(A)
(R)TALSMLLSSER(R)
(K)IANSIVNAMK(K)
(K)ISTDLLVVPSHQK(T)
(R)IHHQFFPDAVFIEK(S)
(R)LISETGDKEFYHGK(I)
(K)MVVKPVTTNFGIGISVLPPR(T)
(K)TALDSYITFLVMENK(T)
(R)GVGHVTPLEK(I)
(R)ILELNFNPVLYIHNYPYEGK(N)
(K)VFLFMK(G)
(R)SLGIQFGSFNVLSDETIR(Q)
(R)MVYSILKPWIDR(G)
(K)FALQIENLVK(T)
(K)STTIGILSSLVNK(T)
(K)NSYSASATGMAR(L)
(K)AFPVTESISAISK(S)
(K)YQHSFTHFPVPYEGFR(A)
(K)ELTQILGIPDSILPETHNSSSLFGR(T)
(K)GLESFFR(K)
(K)SIALQSYELVEAMENDTGSKLK(V)
(R)GFFPSVVDLK(K)
(R)TLIYNIEK(K)
(R)VVRPSNVDTTVLGAAFLAGLER(G)
(K)ILNLIPLEER(D)
(K)INSPGGSVTASDIIYR(E)
(K)LLQSIVDDLFEK(F)
(R)IFTASQALQHGLIDK(I)
(R)IFNEEILTLK(T)
(K)AIGLIMK(K)
(K)FNIPLITGK(T)

(K)GFLQEIGNHR(A)
(K)GHDNTAVFYSLISR(D)
(K)HSESHLLVIGQYINQLEEISK(K)
(K)HSESHLLVIGQYINQLEEISKK(F)
(K)KFNIPLITGK(T)
(K)KYDVPWK(E)
(K)SPEYLHTYR(I)
(K)YGFNLRPTSISGK(K)
(R)AVQPFIESTFPDKIYIK(K)
(R)ISPLSLWNAASIK(M)
(R)LGLTATLVR(E)
(K)IVYDGFVFSNLAMLSPELK(K)
(K)IVYDGFVFSNLAMLSPELKK(Q)
(K)SQVIVGTGGK(Q)
(R)DLIVSLLNAIPGVK(C)
(R)LNVIEPSPTLVITAK(A)
(R)LSYAMGENDIRR(G)
(R)RDLIVSLLNAIPGVK(C)
(R)YTAVSGTVELK(E)
(K)FYLIPGGDHYIHFTK(S)
(K)NPNILSLLFIEDIAPR(N)
(K)YTNYPYTGR(T)
(R)FIFTITK(I)
(-)MKDIIEK(L)
(K)AEIENHLISQK(K)
(K)DVEKENPR(I)
(K)ELPPLR(D)
(K)VKELPPLRDNTILAGNNPSIGPENAK(V)
(K)VLFDNSGNLPKDR(V)
(K)VVSLAAK(E)
(K)YGVSGTPAFFINGVMIEGAQPIEAFIK(V)
(R)AVYEQYK(N)
(R)GLGLDMK(V)
(R)NNFFGQLR(N)
(R)NQYNISVK(V)
(R)NVSEKELLEPSVSAPSIEEMR(A)
(K)EINWALLVGSVPR(K)
(K)EVTHLGIWGNHSATQYPDFYNTK(I)
(K)FKITHEELVSER(N)
(K)FKITHEELVSERNEVK(E)
(K)GLIFGFPLK(S)
(K)GLIFGFPLKSDGK(K)
(K)INGGIFVNQGK(A)
(K)ISGKPVTDVISDHEWLK(G)
(K)ITHEELVSER(N)
(K)ITHEELVSERNEVK(E)
(K)KVEIIQGISLNDFAK(E)
(K)VAVTGAAGQIGYSLLFR(I)

(K)VEIIQGISLNDFAK(E)
(K)VTVSSDLDTAFK(E)
(R)GASSAASAANGVVDTVR(G)
(R)IASGQMFGADTAVEIQMLELEAAIPAAK(G)
(R)LNHASILDGIR(L)
(R)TFIYSTAPMPAIAHAVPTSISIVK(S)
(K)IIIEGAGGLLVPLNR(R)
(K)TFFSSLILGK(Y)
(K)GLSAGVSAVAVTLTTDR(I)
(K)GVLLRPLGNVIYLTPPYNIENSSLEK(I)
(R)TGSVFAYQR(A)
(R)VFQGLMFPTK(E)
(K)AIAVLTGPNMAGK(S)
(K)ETANILNHYTEDSLILFDEVGR(G)
(K)IASSILDIALTK(R)
(K)TIFATHYHELTELSR(L)
(K)TVLAPYITALK(D)
(K)VSYPFLIPIEELKK(I)
(R)LIAELEK(F)
(K)ILLDLESFLK(F)
(K)AGVAPNGRPYQVLIAENSR(F)
(K)NLIDEGAMDYIPKPIK(R)
(K)QLAQILESEGYQVIGFAENGK(E)
(R)IVIVSEENTPAVLK(N)
(K)EFSDVQLNHLYVDNAAMQLIVNPK(Q)
(K)GVANPIAQVLSAALMLR(Y)
(K)TGHPLPPETLK(L)
(R)ELTGGIYFGQPK(G)
(R)TRDIAEVGSTIVGTKEIGQLIESFL(-)
(R)YSFSMEEEANKIETAVR(K)
(K)GYGELLK(G)
(K)LAELLILNSFPGK(V)
(K)LFHTSNLFYSEEASK(L)
(K)SIVDPIILSLEK(S)
(K)VFLTNSGTEAIEGAFK(L)
(R)SQADKLFHTSNLFYSEEASK(L)
(R)TGTLFAFETMGFSPDAMTLAK(G)
(K)IVYSPLHGTGGK(S)
(K)IYSSFQSLK(A)
(K)SLTLEGSAGK(E)
(R)AALGFISYLSK(K)
(K)AAIKDNGGSLGVLAPK(K)
(K)VNLIK(K)
(R)WVTIR(L)
(K)SPGLLGDIIQIFSK(I)
(R)YSNIVTVSMADVVFK(S)
(K)LIHDEIPDSTLTIIGDGPALPSLK(V)
(R)SGYLPSYPDIK(V)

(K)ILSQWGQGLEESVK(I)
(R)GILGLYR(L)
(K)ALQDGWLDSGDILTWTHTGELK(F)
(K)NLGVLIVPFFDR(V)
(R)SFTWGIHQIQEFIPGSYNDR(T)
(K)GAAGQALQNINLMTGAK(E)
(K)ILSHQHEPEIR(E)
(K)SLVVLAVPNEVSVESAPK(F)
(K)TISEVFPEVSFPK(N)
(K)VQHTNFLDISLR(K)
(K)AEIEGDMGDSHMGLQAR(L)
(K)AGAWYSYNTEK(I)
(K)IGVMFGSPETTTGGNALK(F)
(K)KLAIEQAMSQIEK(Q)
(K)LAIEQAMSQIEK(Q)
(K)NNPEIALTIENMVR(D)
(K)TTLTLSAIAEAQK(R)
(K)TTLTLSAIAEAQKR(G)
(R)GGVAAFIDAEHALDPSYAK(K)
(R)mKIGVmFGSPETTTGGNALK(F)
(R)SNAIDLIVIDSVAALVPK(A)
(K)AQLILEAGLSR(K)
(K)GFEFFR(D)
(K)TILIENGILK(N)
(R)FVASENPGAQK(G)
(R)SLLMLSAGYIEGK(K)
(K)EPVIEVPIESSFAPNVFVSVLAVR(G)
(K)FSLFPFEIR(C)
(K)GHWDITPANALGILAFQSYSK(Q)
(K)IIVLDALSR(F)
(K)ILPTDILISLR(N)
(K)SVAGNFLNEGEK(F)
(K)TGPSGTLITK(G)
(K)VYLVLEK(G)
(R)VGWKPFEVPVR(I)
(K)LLVNPLDSVSWIR(T)
(K)EAGADALNIGIGWHESR(I)
(K)EVGFYTSLSYK(E)
(K)HVVVVGSGPGGLESAR(I)
(K)IGGQLNLAAQIPGK(S)
(K)IGSGLGATTAWALLQELESK(E)
(K)KTASYADYLSGK(F)
(K)SKVPEIPSYLIGGAK(D)
(K)YSEIFQPIQIGSIVLPNR(V)
(R)FDGGASFITTGGIAVNHEGR(G)
(R)MIAFYGR(R)
(R)MNFAIEVMK(N)
(R)MSGIDLIPGNPTFEEVILLAEK(L)

(R)VNMPETMSR(I)
(R)YNVTSYTNAQIQK(E)
(K)AIVGANAFAHESGIHQDGVIK(H)
(K)FLLSNVR(G)
(K)LGFSPQVEELEAAYQR(F)
(K)TITGMLVQPNK(A)
(K)TITGMLVQPNK(A)
(R)AGNTAMEEVVMAMR(T)
(R)AYISALNR(L)
(R)AYISALNRL(-)
(R)ETYEIMKPETVGLSSNR(M)
(R)IHTFIASSPIHMK(Y)
(R)ILIDGHVR(E)
(R)KDTFGIQTQIK(T)
(R)LVISPVTEGQDAMAEASVTLEYKGDR(V)
(K)ENFIYINYQNENEDQKPSSK(E)
(K)SFGETFQR(D)
(K)TFAEPFLSAK(V)
(R)DLLSEYGPIHPESK(K)
(R)GIVLEGFALTGGLK(S)
(R)LIFQLVR(W)
(R)YLPSLFTFQSFLK(S)
(K)LQILAPVISGK(K)
(K)LVVITGVSGSGK(S)
(K)STLINDILYNAAAHK(V)
(K)TLYILDEPTTGLHFEDVR(R)
(R)EHNLKNINVDIPRDQLVVITGLSGSGK(S)
(R)LMGVLYILDEPSIGLHQR(D)
(R)QFLGQMEKPDLDLIEGLSPAISIEQK(T)
(R)SNPATYTGLFTPIR(E)
(R)YVESLSAYAR(Q)
(K)AILEKPDIIVTDIMMPVMSGDQMLK(E)
(K)FSFTLPK(L)
(K)KNYLFQVDELPNALANGAALK(Q)
(K)MVQDVIATVLQNYPK(K)
(K)QVWMNLISNAVK(Y)
(K)TEEFVPALMSSINELKK(S)
(K)TIETLENANADMESFSYSVSHDLR(A)
(K)VQIPIYAPIHIEETEQPVK(T)
(R)TPLTLILGPTR(A)
(R)YIASHFDSITK(E)
(K)EHGGSLNFESEPGR(Y)
(R)IILENSIVEENHK(K)
(K)GGIFLYPNDTK(S)
(K)VFNHTLTR(S)
(R)ILDLTPK(E)
(R)KAGLLENILGATETVNVQGETQMK(L)
(R)KSPAGTPGTLSDLLQQGSGQR(A)

(R)LLYEAAPMAFIAEQAGGMAVTVYGER(I)
(R)TTLVVGSK(K)
(R)YIGSLVADFHR(N)
(K)KEPHLSTLR(L)
(K)SLQNLYSFIQTLGR(E)
(R)FFTPEIFTELSFYR(K)
(R)IMFPVLDLSGR(T)
(K)FSTYATWWIR(Q)
(K)IIEETILSSSILR(A)
(K)MYALNAEEAHKLEELFFK(N)
(K)VSLISGETEVFLAK(R)
(R)GMHFFDLIQEGNIGLIK(A)
(R)IKEIDR(H)
(R)LFIQEFGR(D)
(R)LFIQEFGRDPNNEEIAER(L)
(R)LGWPVQK(V)
(R)LVVSIAK(R)
(R)NTLEPASTLVPK(D)
(R)RMEQEAGSTVQEIK(D)
(R)VPVHMIEQVNK(V)
(K)KAEESAAMELIESLR(K)
(K)MVAPVAMVLSK(Q)
(R)FYERPYYGIGLTLYDILASK(G)
(R)IELVSFIK(K)
(R)ILFVDLSLAQK(M)
(R)LNVLLAR(S)
(R)TFNVLSPSQGIHLVFSK(E)
(R)YLAQFHFK(L)
(K)AGFDPTAPDLHLGHFVQLK(K)
(K)EGTDMGGYIHFEMTER(E)
(K)FEGDISGTINAPVKK(V)
(K)ITISEVSEVSSIPIRAIFmIVGK(D)
(K)KELEILNNVFP(-)
(K)KVFQHLLNLEEIPK(Y)
(K)VFQHLLNLEEIPK(Y)
(R)AIFMIVGK(D)
(R)AGIEVLSASLK(E)
(R)FTAFPGIISNNSGYTGSR(L)
(K)EFLFEELKDYLK(K)
(K)SYLSNFTFNPK(M)
(K)TPYEIMIPILQK(L)
(R)VVLPAELLK(K)
(K)NQVQNLILK(W)
(K)VVTPMGTVETLK(E)
(K)VVTPMGTVETLKEPASSTGPELNR(I)
(R)IFAGSEGLLGIITEATIK(V)
(R)LYQTLGTLGK(K)
(R)NHLMENGIGVDTMETSTTYDR(V)

(R)WNSWGAYGQDFFK(V)
(K)FFSNGFNPEIFVDK(S)
(K)NMAIGSVVGIK(V)
(K)SVILTSK(N)
(K)VSLRDPVR(I)
(R)LGFPESQIGINFPAVAGFMLK(E)
(K)TANSGMQVLPMGDILLK(D)
(R)AILYLTSLSDTIR(N)
(K)VVAPNVLEALDYYK(R)
(R)LALIIQSPGYDPK(T)
(K)INPSPIFILQVANESGVIQPLEK(V)
(R)LISTPPTLISIVPIATGYELR(L)
(K)LIVMLYDGAIR(F)
(R)IINIHPSLLPAFPGLNAQK(Q)
(K)AFSHTAMYDAAISSWFYK(Q)
(K)EALDELDLRPIHHGLLIQDR(D)
(K)FLNQNGVEIISTGGTLK(L)
(K)FTQEALEVFSK(K)
(K)IDLVVVNLYPFLK(T)
(K)TVSKPEVQLEEAIENIDIGGPSMIR(S)
(R)KAFSHTAMYDAAISSWFYK(Q)
(R)RTDPISAFGGVIGIK(G)
(R)YGENPHQAASFYEPLFTK(S)
(K)AGATFISPFIGR(V)
(K)EALELVEIAENVVIK(V)
(K)TVVELTK(R)
(K)TVVELTKR(N)
(K)VPLIPEGLK(T)
(R)GPIHLK(E)
(R)ILAASIR(G)
(R)NIPTNVTLCFSAPQALLAAK(A)
(R)VDDTSWDGMELISEIR(E)
(R)IDPEIDVVSYLGDVVKPIVESK(K)
(K)DYFIFSFDHIPLSELGDVK(A)
(K)SIPIEAIVK(Q)
(K)VYTITGGSVITSLK(K)
(K)VYTITGGSVITSLKK(K)
(K)GFFSGEGLFFLK(V)
(R)AESGAMVAMSPTVK(M)
(R)ALLGGESFFQNTFK(S)
(R)NQNSFVSWANQWR(R)
(-)MNIEIISKPSYSFAK(V)
(K)AESGSMMSMSSGITIQTHK(A)
(K)SFFLGGEGFVAR(F)
(K)VLLNGGESIK(A)
(R)NVPSLGSWFR(N)
(-)MNYEIIHKPSYSFLK(V)
(K)STFFGGEGLVMEFTGHGR(V)

(R)VLIQTR(V)
(R)GISVGIFHPGMVATR(M)
(R)IFEGMDVLNSNSIR(D)
(K)AISDGWMNTGDMGMINFKK(T)
(K)ENGINESNNEKLIENPK(I)
(K)GVVFIK(G)
(K)HSNMMHQVNYVSPMLDIK(A)
(K)IYDLYR(K)
(K)LAYFFSR(K)
(K)LIIGSVGVIVPK(T)
(K)LQEWAK(E)
(K)NLGAIVVPDFEKLQEWAK(E)
(K)SQLTNVKTLImmDPTSNSPGVLK(M)
(K)TLIMMDPTSNSPGVLKMQELIEK(G)
(R)AATGGQLEASISGGGALPR(H)
(R)IAAIDPEDLFTLIYTSGTTGLPK(G)
(R)LLSILPIWHVFER(V)
(R)LWENIYNGIYTR(I)
(R)QDLATVKPTFMGSAPR(L)
(R)NEVAEFFHIPLEK(M)
(K)ILSDFKPIQK(V)
(K)IVLEIYHSDFK(V)
(K)GTYTVYVK(F)
(K)NLLSAVK(Y)
(K)SQGVVEEFNKVEEYNK(N)
(K)YLFFYSFK(M)
(K)INVFLGPLEK(R)
(K)KKEDTIAEMENLSGQALK(Y)
(K)NLEAAEEEMLSAGFAQILK(M)
(K)SGPSILVLQAGIESSLK(N)
(K)WKPVSQEDWR(S)
(K)VITEILTK(V)
(R)VEIPFSANYISIR(S)
(R)YIEMLYTITK(E)
(K)KWEALIPIGR(L)
(K)QFGSLDVAILNAGILR(D)
(K)TLGTEVYSFK(A)
(K)TVLVTGSASGLGK(A)
(K)WEALIPIGR(L)
(R)VAGIAPGFIK(T)
(K)EHSVGELVDLLGISQSAASQHLSK(M)
(K)EHSVGELVDLLGISQSAASQHLSKMK(I)
(K)GIAHPDR(L)
(K)GIAHPDRLQILFFLSQK(E)
(K)INGILSSR(K)
(K)KNQLESAIR(G)
(K)NQLESAIR(G)
(K)YKDLIR(T)

(R)KESNQVFYYLK(D)
(R)LQILFFLSQK(E)
(R)TIVKIYG(-)
(K)IATTIQHILPPTPAK(I)
(K)VWAYEESGVHSLAAAVVK(E)
(R)FSVVTNLYGSER(R)
(K)LILELR(K)
(R)VTAMLPIYETFLK(R)
(K)FLSHIR(E)
(K)SFQLITLKPVMYVANIADKDAAK(K)
(K)VVPAIIEFVDIAGLVK(G)
(R)AWTTPFNSTGPK(A)
(R)FEEEISGLDRNEQLEFLK(E)
(R)LAEIAKPQK(V)
(K)LLGMELNLYPPWTPK(N)
(K)MIASINAGLTENLIER(A)
(R)NLIIVPR(E)
(K)IIASNVVVSFLK(I)
(K)NVIAIGSGK(G)
(K)STVTVNIAAMAASLGYK(V)
(R)ASAMFSQVK(V)
(R)ASAmFSQVKVPILGIVENmSEFICPK(C)
(R)IQIGLEAQIR(Q)
(R)KGEVLGILELSESE(-)
(K)FFHLELK(G)
(K)KAVAIALR(N)
(K)NIIMIGPTGVGK(T)
(K)QKAEEALLDILLPFPGENK(H)
(R)FPIRVELEK(L)
(R)KKLDPEMR(E)
(R)LLEEVSFEGPDLPESQRK(V)
(R)VEEMGIIFLDEIDKIAGR(E)
(K)KVQEFGGSLSR(S)
(K)VAIGGDGQVSMGNTVMK(N)
(K)VQEFGGSLSR(S)
(R)ALYDHTNLSPK(E)
(K)LLLVGIGNPGQK(Y)
(R)FGVGKPSITSEVPDYVLSNFLPNEK(E)
(R)TDEDGVTIFYLKPLEFMNLSGK(S)
(K)GNLYANPILPER(F)
(K)KGNLYANPILPER(F)
(K)SLVEHLSK(E)
(K)TMGVHPDQTVAAVHATEK(M)
(R)IFLPDTLGVLSPEETFQGVDSLIQK(Y)
(K)FAGASFQSLFDQMSYK(L)
(K)LSTLFETPQQK(L)
(K)LTILLR(D)
(R)TLLSTIQSHSITTESLHLK(Y)

(R)FILDNLPFSK(Q)
(K)ISFEDALMELEQIAEK(L)
(K)SKISFEDALMELEQIAEK(L)
(K)TSLFGLIK(K)
(R)LSENMSFSQLNLR(I)
(R)SVTLDNIFR(L)
(K)NILNLDQFR(H)
(K)VTNKPTSEESF(-)
(R)FINSYFPGETSILLNLPLDR(L)
(R)GIESFFNDSLGLITHR(Y)
(R)GLFVDSVSLSSVITQHSNK(E)
(R)IPFPMETVEVTGNIWR(I)
(R)SEIMSISHGEEEEYR(H)
(R)SIVSMNETERPEVLYLSGGGGK(I)
(K)DAYELQSHLER(Y)
(K)IPILGSLAR(K)
(R)ELFPFLAK(Y)
(K)ETYKPTTSELESIGISK(K)
(K)IGLTFATGLR(A)
(K)MVNVILSQAVNER(A)
(K)SYHAGISSR(I)
(R)IGIYELLLVNSPLK(Q)
(R)SLIYEPHGIILVTGPTGSGK(S)
(-)MNAIFLELR(K)
(K)MLEIWGAVK(T)
(K)VGLETTNR(S)
(R)SISLNYVVLEK(S)
(R)SLSSYEEMVQGNLIR(G)
(K)FAVSELKPGMR(F)
(K)HTILGYQILSQK(M)
(K)ILPHEAIK(S)
(K)ITFGNLFR(E)
(K)TMDEKPLEVTPVR(E)
(R)IGVVLGPNLDKPIRPFIR(I)
(R)IKITFGNLFR(E)
(R)IYSISDNFSALVTNRPHR(K)
(R)NLILVDLLK(E)
(K)IELIRKGK(V)
(K)LYYLR(N)
(R)IFPLFSPR(I)
(R)RVSYDVGVER(I)
(R)TQNFTVGDTVK(V)
(R)VQIYEGVVISVANEANGK(T)
(K)AIESGVFSVNIIQLR(N)
(K)FNFITLFPDK(I)
(K)GIVILTSPSGIPFNQSIAMDLK(K)
(K)YIVASLVEFPEEIVIR(E)
(R)AILTAASVK(A)

(K)ELIGSRPIEIEVDGGVNDTNIQELR(K)
(K)ISASILATK(L)
(K)NGVDIVVVGAGLYK(T)
(K)RYPDKTDGIILYQSIRPGR(E)
(K)SYPEVHNELGR(L)
(K)TDGIILYQSIRPGR(E)
(K)TKEEFSPTSFQGASFPLVQK(S)
(K)VFLLVTK(V)
(K)VIVPDLIGK(S)
(K)VIVPDLIGKSYPEVHNELGR(L)
(R)VIVPDVR(G)
(K)KEIRPLK(L)
(R)SAVFAPVSNLGLVIIDEDHDSSFK(E)
(K)ESILSQLAIFQNR(L)
(R)TLDFLPGVPMIVLR(S)
(K)FAIAQAASTNAR(V)
(K)KIESEAVSILQNHFWPGNIR(E)
(K)FITLISGILDR(S)
(K)GEWAYITSR(T)
(K)LGIPLMISEVSTHK(F)
(K)ILPSSQRK(K)
(K)KLEPYPATLYTPNKPDYVIPDVIVR(E)
(K)SSLSEHLLWQLR(L)
(R)GIMELK(W)
(K)IIFFPR(E)
(K)ITTDPDPILIAKDRYLTGK(Q)
(K)GHITYLSQAR(E)
(K)EIFHIPTVAK(E)
(K)ITLGGAGNVVK(N)
(K)IVTVDPQVSHFFLYK(G)
(K)NLAGLLHHENTDK(D)
(R)IISEVSKICVDKK(K)
(K)ITHVGLVINEK(E)
(R)YTLASDLLSQLK(S)
(R)LSDYFFVAAR(F)
(-)MIINHNISAIFAHR(T)
(K)FNSESMGKDIEK(L)
(K)GLMNAYENIQAAESR(I)
(K)LSSGMRINR(A)
(R)IRDTDMAEQMTSFTR(Y)
(R)LEHAAKGLMNAYENIQAAESR(I)
(R)TQILGLR(R)
(R)YQILTQAATAMLAQANMKPQTVLQLLK(-)
(K)AIQPHAGGVIMNWSKPLADEIR(D)
(K)ALSFYQELAK(R)
(K)DAHLSAGFIGFHLNR(S)
(K)DAMFKPSTSFMISYLAGVGALEK(I)
(K)DVDLEIAWKK(I)

(K)GAVQEILSESK(V)
(K)IEWLR(N)
(K)IIKDVDLEIAWK(K)
(K)IISEASADLKK(V)
(K)IVPDEVIHIETEAEITER(K)
(K)LEVDEKLEAVIPEEFRK(T)
(K)LLELSLTQELLEK(E)
(K)LLPYAGLNSGFYK(L)
(K)LTLITRPIGELIK(D)
(K)NFLIELNIENTR(H)
(K)SLNGIEAGLLSSVLASHNGYQSGLELLK(L)
(K)VAGAYDSNIALLISHGENR(K)
(K)VAGAYDSNIALLISHGENRK(D)
(K)YTHIFAGYGFMAEDSEFISAIEK(S)
(R)EYGILLSEKDSVVYPMALAPELR(G)
(R)FPNNIHR(V)
(R)IEQIISIAK(D)
(R)IQFHQSSSPWIR(S)
(R)KLEVSVTPGVDNISSLALLAK(A)
(R)NPDMGLFVHYK(V)
(R)SFSLESIK(C)
(R)VTEMVYSLK(F)
(K)DYVYKDEVSSIQK(E)
(K)EAIVIR(D)
(K)KLQTLSTQYEK(E)
(K)LQTLSTQYEK(E)
(K)SLDGASLVTGILNINGR(D)
(K)VHGQSGVVDIVTGDELGSLR(T)
(R)DVAVYGHDFTLR(A)
(R)FIYEEEILFK(K)
(R)FIYEEEILFKK(T)
(R)IAVMGPAGK(D)
(R)IVLPGTTR(N)
(R)KISGVVPSLMLMFGFNAGGGAYLPR(Q)
(R)KLLDSIIDIR(T)
(R)LLSYLPDNNHSFAPFHATSDPTNR(F)
(R)NILFQNLDYLIR(H)
(R)NLVTAFGR(I)
(R)LAGIKPAVK(K)
(K)GSGPPLGVLPSASYK(G)
(K)QIVVVGSEDNERYPGTPDVPER(I)
(R)GYVERPNQIR(M)
(R)HAEGVGLAAPQIGILK(Q)
(R)IILNPVITPLTK(D)
(R)MQWMDEK(G)
(K)LFPGSVDVFQIK(L)
(K)LVTKPLEEAIQEVDGIK(E)
(K)SLVEFFLSK(S)

(K)TIALTVIK(R)
(K)VAQAINTAFQGTVATK(I)
(K)YLTSQDIIFALK(N)
(R)NINFPGGNIAGNQK(E)
(K)IKDWIEPGDLDQEIVVK(N)
(K)ATVLNLYHNFQWPAETIAEK(L)
(K)VTIDTVVIDGYDKIFDEVTQSASK(G)
(K)IFSLKAEK(V)
(K)FGEAYLSWANSVPAFIPK(F)
(K)GNYQNESGPNLR(R)
(K)ILFFDEGIVLIPSK(T)
(K)KNQVLESSPSESFYEEITEEK(S)
(K)ATGFSASETGIREELSDEAR(R)
(K)DQSLLDLWELKSGNQNIR(L)
(K)IASLLGANIQELKDQSLLDLWELK(S)
(K)NASIIYDTLLNSDLKK(T)
(K)YPIER(R)
(R)ESVNGAGYSDDEVRKPEVIYK(Q)
(R)SLVVTEQIAK(A)
(K)IAGVLMPGMSEQEIK(N)
(K)IAGVLMPGMSEQEIKNEYK(D)
(R)AELVNPFLEAATIVFR(D)
(K)SVQELLGHIDIATTQIYTHMANK(T)
(R)LLNHYIK(R)
(K)TSEIPAFLPPAR(R)
(R)AESSLPGDSSLLLYLLEIHLQEK(D)
(K)ELVVVGGGDSAVEEASHLTK(F)
(K)IEIIWNSQVK(E)
(K)IVIIGSGPAGHTAAIYAAR(A)
(K)IFIPFTVGGGIR(T)
(K)NFSSSLGIPIIASGGAGNPEHMVEAILR(G)
(K)VSINTAAFQNPK(L)
(R)DRYEVFLHGGR(T)
(R)EILLHLVER(I)
(R)GKADAVLAASIFHFGEYSIR(E)
(R)NGFDIHLLK(N)
(K)AFLYLDEK(K)
(K)GLPIGLQITTPHFQEGK(L)
(K)SPFHATVVEK(L)
(K)SYVELK(D)
(K)TKDPIQMYK(A)
[illegible]IPGGSSGGSAAAVAASMVPLALGSDTGGSVR(Q)
(R)YGLVAYASSLDQIGPFSR(E)
(R)FHNFGVGTTAVTNQVDR(G)
(R)NLLYYILNPADK(S)
(K)DKSQEILPK(V)
(K)ENLLEFISKDISEVPEQMR(A)
(K)FQEMQIVSQLYVMHLVESK(I)

(K)HNDKFDLDAFLK(K)
(K)KNFYDQYR(D)
(R)ALNYQFQK(K)
(R)IKEQVAIK(H)
(R)SKEPQISSLPIDR(C)
(R)VQNIEKENLLEFISK(D)
(K)ILSGEPVLIFTSVSGFLK(T)
(K)IVLTSSFEAQTK(R)
(K)TLPPIQTMQGR(A)
(R)LQGLFEK(E)
(R)SSQGLALSSR(N)
(K)NIQIVTPPR(K)
(K)SFVLEISK(L)
(R)IYSSLGVSTFLK(R)
(R)KGTLPDGLIAAAK(I)
(R)VTWQEVSK(K)
(K)FIVESLK(S)
(K)LIFFFHGFPDDAGSMK(E)
(R)AGIPEAALLK(N)
(K)AFDSGAIDYVVKPFDAIELLAR(V)
(R)LFEEmTRRK(E)
(R)NILENIENLGIPHSASK(T)
(K)AFVSLGSSQSTFAK(E)
(K)DGFYNGLTFHR(V)
(K)FTLSMANAGPNTGGSQFFINVR(D)
(K)SVIIETITVSP(-)
(K)TNFGNFSVYLDEEKAPITAGNFIK(L)
(R)HAVFGR(V)
(R)VTEGMDIVETISETETGFQDKPTK(S)
(K)LGFGGAAVVSIVPENK(A)
(K)LLQSNTNFMNLPIVESMVK(S)
(K)SLHSIPEIPPGEFSVSIHIAR(E)
(K)TITKPVLNLLR(A)
(K)TNSPSSATTQELSTSQVK(S)
(R)ATTEIAQGNFK(I)
(R)VGIVWEPPK(K)
(K)FTIPVSR(N)
(R)SLIVLGELK(S)
(K)STLLDMIVGR(E)
(R)VLSAADmLELFLFDTR(L)
(K)AFFVVIYGTLK(V)
(K)FYQAISHFLAK(R)
(K)GDDVNVTTIATGDHFGELPFLDPGKR(S)
(K)VYEQGDSIFHEGNEAK(A)
(R)IPYDHLK(A)
(R)MLTHDLAYAR(E)
(R)SASVEAMER(S)
(K)ILPYSQIHSPVLVIQAGK(Y)

(K)EAFSGSNVTVHYVGTLTNGK(K)
(K)LTIPPELGYGSR(G)
(K)NPFTFNLGAGEVIK(G)
(R)GAGAAIPPNSTLIFEVELLK(V)
(R)KLTIPPELGYGSR(G)
(R)KNPFTFNLGAGEVIK(G)
(K)IFSEFGTVEEVHIK(K)
(K)ISVGNLPQELTEDELKK(I)
(K)FGLIPEFIGR(L)
(K)HPHQEYLQVDTK(N)
(K)KAEVGIIYIDEVDK(I)
(K)SILDQYVIGQDHAK(K)
(K)SNILLIGPTGSGK(T)
(K)TLLAQTLAR(I)
(R)IQSENKDVLMEQVIPEDLIK(F)
(R)LPIVATLQELNVDMLR(Q)
(R)VAPTLILK(K)
(K)EILNSIYHK(H)
(K)LKEILNSIYHK(H)
(K)NYGIIDTVIQIDRK(Q)
(K)RSALPNAR(I)
(K)TVEQIQK(D)
(R)ImMHQPMGGATGQASDIEIQAR(E)
(R)ImMHQPMGGATGQASDIEIQAR(E)
(K)ALYANILPAVDDDLASEFDGSESLNVLK(D)
(K)HYFTR(Q)
(K)IKLPEVSVSDLDVSEEVEK(V)
(K)IPGFRPGK(A)
(K)KIDNILFDLVEK(K)
(K)KIDNILFDLVEKK(Y)
(K)LASSYQISLSDLK(K)
(K)LASSYQISLSDLKK(E)
(K)LEHPMIR(F)
(K)LMEQYR(E)
(K)LPHMTMEK(Y)
(K)NSGNTSNDYHLGHENNLK(G)
(K)TFEYSVTIK(A)
(K)YVFPESYLR(E)
(R)HLGDSVASDAINTLIVDGMTSILSK(L)
(K)ILFSFSPTLESK(K)
(K)LDPDFPATLSSKILK(G)
(K)MSPEELSGQVIHVSIPGK(T)
(K)NFLEIFSSNLNAEISR(A)
(K)NIPNVNYSK(I)
(K)STIPVVDLILK(G)
(K)VGFVTSYQLR(K)
(K)IADLTHSSLTIEYSGNSR(K)
(K)ILVNNHPGVMSHVSGLFTR(R)

(R)ELVLLVVK(L)
(K)EHPLDFDNSGNEIKPQDFLER(L)
(K)HILVR(H)
(K)ILVFNNNFLGMVR(Q)
(K)LAEAYSIPAMK(I)
(K)VFPMIPAGK(S)
(R)HEQGAVHMAEGYAR(A)
(R)HFEEATLIALGGRPGPVLLDFPK(D)
(R)IQVDYLLHGDLK(D)
(R)KEWLTHLQNLK(K)
(R)SQKPLLYVGGGAINAGASK(E)
(K)DPHNTYGLVGMGDLFFK(K)
(K)ELLEKDPHNTYGLVGMGDLFFK(K)
(K)LKELLEKDPHNTYGLVGMGDLFFK(K)
(K)LLDSHQFPK(A)
(K)LLLIQPDNIK(I)
(K)NFFALYGLAESYR(G)
(R)HITITDASILSR(V)
(R)KNFFALYGLAESYR(G)
(K)IKELHLR(K)
(R)LQAIAPYR(K)
(R)LQAIAPYRK(W)
(K)AFTELK(D)
(K)GLVLAK(S)
(K)LGEYFFQTGELSHK(T)
(K)LLNIVDIK(G)
(K)LNLIVNLTR(-)
(K)LVVHEVNEAR(M)
(K)RTEQELLNPDIGIFPEK(S)
(K)SQIYQMELILPVLK(L)
(K)VNIPPINVEMELNLLR(K)
(K)YEQIVPALIAK(G)
(K)YHAFEEIAGLVPLEQNLK(G)
(R)FLSLSK(L)
(R)LTNNLDKYHAFEEIAGLVPLEQNLK(G)
(R)TKDQNTMELLLGQIK(G)
(K)GLPGLSLFSETPSK(L)
(K)VLVDIFK(K)
(K)LLQPSIFFLK(N)
(R)FLAEELGLPFR(V)
(K)LVFKPIQISDMK(D)
(K)LEPALSASIR(L)
(R)EILSSFLNSLSK(I)
(K)ASVILHYIPGVESLSR(K)
(K)GAIEQSLMTLR(S)
(R)LREEELAAQQQMMR(E)
(R)FAEELDELEKR(K)
(K)LFSIIDGVK(I)

(K)DVMVQEGVLHSFVSAGGK(I)
(K)VTILPPRPGGR(D)
(R)SIDWIPR(S)
(R)GEMSVLSSLIIGPNK(Q)
(K)SGSFLLYSTLSGSVNGR(V)
(K)TGGGVNGSPSETR(N)
(R)VVLAMSAENAVK(I)
(K)LHGASISNYVNK(V)
(R)EISTIIETYLDIPARR(I)
(K)VANSVPTVLK(F)
(R)LSGIGLKPEIK(K)
(K)ALANLGYYNVQFLKPVYPGDTLSAK(T)
(K)ASTGSDTYFENFQPGQIYIHQNGR(T)
(K)GNPKPVIK(D)
(K)GSDKPGIVSVR(T)
(K)IFEIER(K)
(K)IPNKPFAELAPNTTVSK(D)
(R)AFAQEFATTFMDANPLFLSAPYAK(A)
(R)KIMIYR(S)
(R)SNGKPK(G)
(R)TITDEHFPWTYR(V)
(R)YLEEFTEGEIFEHPR(E)
(K)IWIPFAK(Q)
(K)EGIQVTLLNMATIKPIDK(E)
(K)ELLDYFGLR(S)
(K)QIIHAIADYK(G)
(R)AWEIVR(N)
(R)NSVVYPFLNVK(L)
(R)VGRPNVPIIER(E)
(K)AKPVQKLDDDDDGDDTYKEER(H)
(K)AYYLYVWIPAVIAEMGVR(M)
(K)AYYLYVWIPAVIAEMGVR(M)
(K)IPNPPK(S)
(K)IPNPPKSFDDLK(N)
(K)KLLVR(G)
(K)LDDDDDGDDTYK(E)
(K)LDDDDDGDDTYKEER(H)
(K)QAIAAEESLK(K)
(K)SFDDLKNIDTK(K)
(K)SMPHWFDTWIR(V)
(K)SSFVLSEDTIPGTNETVK(T)
(R)IKIPNPPK(S)
(R)ISFTTYKPGEVK(G)
(R)MISPTGEIGEPGDGDLVSDAFK(A)
(R)MISPTGEIGEPGDGDLVSDAFKAATPEEK(S)
(R)MSAIMPDQIAK(A)
(R)MSAIMPDQIAKAAK(A)
(R)AILLEASR(S)

(R)ILIGLLLEK(G)
(R)YSMAMMNGAR(L)
(K)IALVAGATGLIGK(C)
(K)IVNPFLWGGIR(K)
(K)SFVFYNR(V)
(R)SFLVVSALGADPK(S)
(K)TYTTFK(E)
(K)EYLIIR(E)
(K)IGSIFVPDTAK(E)
(K)VGDTVLYGKYSGTEIK(S)
(K)VVEIGSGK(Y)
(K)VVEIGSGKYEDGK(L)
(K)VVEIGSGKYEDGKLIPLEVK(V)
(K)YSGTEIK(S)
(R)VLVEPR(Q)
(K)APGFGDR(R)
(K)AQEAVGSLK(L)
(K)AQEAVGSLKLDGDEATGAK(I)
(K)ARVEDALSATR(A)
(K)AVTAAVESIQK(R)
(K)AVTAAVESIQKR(A)
(K)DIANVASISANNDNTIGNLIADAMDK(V)
K)DIANVASISANNDNTIGNLIADAMDKVGK(D)
(K)DLIHILEK(V)
(K)EIELEDPLENMGAQMVK(E)
(K)FGAPTITK(D)
(K)FGAPTITKDGVTVAK(E)
(K)GNEGFNALTMVWEDMIQAGVVDPAK(V)
(K)GNEGFNALTMVWEDMIQAGVVDPAK(V)
(K)ISSMKDLIHILEK(V)
(K)KDIANVASISANNDNTIGNLIADAmDK(V)
(K)KDIANVASISANNDNTIGNLIADAmDK(V)
(K)KFGAPTITK(D)
(K)KGNEGFNALTmVWEDMIQAGVVDPAK(V)
(K)KGNEGFNALTmVWEDMIQAGVVDPAK(V)
(K)KQIEDTTSEYDREK(L)
(K)LAGGVAVIHVGAATEVEMK(E)
(K)LENTTLQMLGR(A)
(K)LLEGVNK(L)
(K)LLEGVNKLANAVK(V)
(K)NVTAGANPMSLK(R)
(K)NVTAGANPmSLKR(G)
(K)SIETTLDVVEGMQFDR(G)
(K)SMLEDIAILTGGQVISEDLGMK(L)
(K)TISCVAVK(A)
(K)TNDVAGDGTTTATILAQSIINEGLK(N)
(K)VTLGPKGR(N)
(K)VTVDKENTTIIEGK(G)

(R)AAVEEGIVPGGGLTLLK(A)
(R)AAVEEGIVPGGGLTLLKAQEAVGSLK(L)
(R)ALEEPIR(M)
(R)ANKVTVDKENTTIIEGK(G)
(R)KLLEGVNK(L)
(R)KSMLEDIAILTGGQVISEDLGMK(L)
(R)MITSNAGLEGSVIVEHAK(A)
(R)NVVIDKK(F)
(R)SALQNAASIGSMILTTEVTITDKPDK(D)
(K)KVPSSHVELSSVSGLKEEEIPGLTK(K)
(K)TINGEIFLAGER(G)
(R)YIAPLHSLEEMNTSPSNEFR(K)
(K)DIIAILEALKK(S)
(K)HGIGPESVVPISPSEINIVLGK(S)
(K)SLEGGVLLQSPLK(T)
(K)TVGVVHGGAIVEQELDQNFYASER(V)
(K)RNPWEPVWK(S)
(K)SDKEEQLDTIYLEQMIK(E)
(K)ENIQFILPK(G)
(K)GFVYPHHSSKFDIDEDSLSIGLSVLK(E)
(K)TGVVSLIDSGKPGK(T)
(K)YNVDAALALHVWNHIPIGK(I)
(K)EGVEFASIHNQAVK(T)
(K)LDEILLNQHTLFHFFGK(N)
(K)LIEWIYSLNQR(S)
(R)ESKPGMFEYELEAILESEYLK(H)
(R)TSHYLGMDVHDVGSYYK(D)
(K)GFAMSMAQGGGMIATR(Y)
(K)TELDYHSLSYQTAIQLLER(N)
(K)THPEGQLPINASGGLK(T)
(R)GHPVGVSGLAQIAELQSWMFK(K)
R)GIGHGIGNLSNPPGDLSFSSSVTAFQGAYK(E)
(R)GIIIATEVMSR(L)
(R)YLQQYGYDRR(D)
(R)YTGEVFVSAR(I)
(K)ILNSEHSLR(I)
(K)ISEGFIVTNPPYGVR(L)
(K)LLYGGHTNYR(S)
(K)LVAYTLGR(-)
(K)NAGVDHLIHFFESDAK(I)
(R)EILASALIR(Y)
(R)SLASSLPFQK(L)
(R)FPILFWR(A)
(K)IARLDFETDRFLYSK(N)
(K)IGGMGIPQFVNDLPGGGGK(I)
(K)FEPILVLDNKR(I)
(K)GEGEFYPNVLETIEILAQK(G)
(K)KGEGEFYPNVLETIEILAQK(G)

(K)TIFFNLLPQLNEEQR(D)
(R)IMAEIGKPVK(T)
(R)KPYIQTILEVSGILPK(F)
(K)ITGLPLK(E)
(R)AFSPVEIINLEK(I)
(R)TGGFLSFIPWTFQPQTK(R)
(K)KPIAILIEDFSLQK(L)
(K)SFPASIEEIIR(L)
(R)SFMELLPK(K)
[illegible])AGADALIAVAQGAGGHAGNISPFSLYPYLK(K)
(K)VELLITSLGSPR(T)
(K)IAPPPLFFPLK(D)
(K)NLFSLLDEIEK(V)
(R)GIPIVTFINK(M)
(R)GLNVLHGR(L)
(R)TFAIIAHPDAGK(T)
(K)AATIAVSVR(N)
(K)GLDTTSFVVYPEGHNSYR(E)
(K)LAWENK(I)
(K)FLGLAK(E)
(K)LKYPYDNYYLFK(S)
(K)NSFIVR(D)
(K)RVELLLYLR(A)
(K)YPYDNYYLFK(S)
(R)AIEPIVR(N)
(R)DFHYVTNK(Y)
(R)LYQEGLQYFFENNHVNAYR(R)
(R)MSPVFDR(R)
(R)NLVDIAIEWSK(N)
(R)VELLLYLR(A)
(R)SLAKVASLK(G)
(R)ALTTIPVYLALGYK(I)
(K)AHKDHFADGASFDQLYVK(-)
(K)DKYEVVIPSLSILAEPPVAIVEK(N)
(K)SLPNNSTPYTSTIVFVVR(K)
(K)SLYSEAAQEIIAK(H)
(K)YEVVIPSLSILAEPPVAIVEK(N)
(R)WNYLAAWAFAQK(K)
(K)INPTSENIAR(W)
(K)LKELVGR(L)
(R)IVIHEGPENLAFFEPTSLS(-)
(K)KPIVLLEGISTAQQER(I)
(K)TLVALAALESQHK(I)
(R)GQIYQPYVVSEIRDPVDNSIINR(T)
(K)LTIGNAFPEKK(A)
(K)YIKPGIR(-)
(R)GIVLTGGGCLLR(G)
(R)GLEMYLSK(E)

(R)IVIGVPSGITEVER(R)
(R)IVIGVPSGITEVERR(A)
(R)TPGEIVAIRPMK(D)
(-)MVQEKPLAAIDLGTNSFHMIIVR(V)
(R)DLKPDLVVGSSGTIQSIAGMILASR(G)
(R)ELLEAASLLHEIGQSISHSAYHK(H)
(R)LIYFGILQGIPVFDR(K)
(R)LYIEEMLLPFR(K)
(R)LYIEEMLLPFRK(I)
(K)VLSVEGGFVEIK(D)
(K)VSVISPEK(I)
(R)ILVASR(-)
(K)ISDITLNFLGVLLNR(G)
(K)KLPVGAIYEN(-)
(K)NFFLSPTVSNEEK(E)
(K)SIASQLGEIK(K)
(R)IIHLPEIQK(Q)
(R)SYPSLEPAQAAK(L)
(K)FAWDVILK(A)
(R)AETVQNDIKK(A)
(K)KPGFEHYVLEGYEEDR(K)
(K)EAILEGIIESHDK(I)
(K)GVKTPmPLQAYFK(V)
(K)TPMPLQAYFK(V)
(K)AEAVLGK(E)
(K)EGGIGEIKYPLIADLTK(S)
(K)KEGGIGEIKYPLIADLTK(S)
(K)LGTEVLGVSVDSAFTHLAWK(N)
(R)DYNVLTEGGVALR(G)
(R)GTFIIDPAGVIR(Q)
(K)INEAMNFK(T)
(K)TTTGELTIR(F)
(R)FLQPAFVNQPLHLR(G)
(R)SVIDTLLK(Y)
(K)LLTGLISEQKNESAHSFTSGKR(I)
(K)YPKPGIILLDLNMPR(M)
(R)KPVEFEAFLETIR(A)
(K)ALQQFNELR(Q)
(K)FIGDAIMAIFPAR(A)
(K)LLGVFAVESAEMNAFK(L)
(K)SQIAIPLLVK(D)
(R)AENAVEAALAMMK(A)
(R)YAGQIGQSMGR(E)
(K)FELSELFDQSPGIK(A)
(K)FMTVMVPK(V)
(K)LIAVTVAHMANFK(T)
(K)YIIRPSVAVK(F)
(R)DAELKPHFAR(A)

(R)NVSVIMAR(L)
(R)SLAMLFQK(T)
(R)VNYAGHMSGR(S)
(K)IMAISDIFDALTDKDRPYK(R)
(K)LSLISPYEFGFLTIK(K)
(K)SSVAGYVAATGK(I)
(K)TFLNAFTQLQMITDQFDLQHK(V)
(K)IMTQGSLQTTGK(N)
(R)MDVIGNNISNVNTHGFK(T)
(R)RGHVTTINTFDDQGIQR(E)
(R)SLYSGVSGLK(N)
(R)VATAVFNNPAGLDK(A)
(K)IAEIISR(Y)
(K)ILPQEIGNLR(K)
(R)NQLISLLPEIGNLK(N)
(-)MQESGLKPILWK(N)
(K)ALNPSFDVTPASLITGIITEK(G)
(K)GAEELALFMLSEDLENNLTLSK(Y)
(K)GIVSPVTEENLK(K)
(K)IQSFLITDNMAGWVMSSR(K)
(R)VLPGTTSYITAK(T)
(K)GVAPVPGENGWVR(F)
(K)NLNELVESLQK(E)
(K)IANGLNVPFSELLK(E)
(K)LIRHTK(G)
(R)AMLSQIEQGK(S)
(K)EVQLQIFHSINER(G)
(K)ILSFFSAEDLYR(I)
(K)LELFLSGSSK(E)
(K)LFGFLTEQDKEFFK(V)
(K)FVVNILAQGQDSISNQFASGK(T)
(K)NALSHFPSGVTVITYSHLGK(H)
(K)VISAGSDDGLRPLLYYR(R)
(K)IFHFAR(M)
(K)SLFENPEIVK(I)
(K)YLISLEQK(L)
(K)EIGDWLTTNDKFPGGMR(L)
(K)ISLTPIGISR(F)
(K)KLPIQFFQIDDGYQK(E)
(K)LTPGIWLAPFLVR(K)
(R)ALINDINR(S)
(R)EKLTPGIWLAPFLVR(K)
(R)FTIEPESYLK(K)
(K)AYDYYVILK(E)
(R)TSIPSK(E)
(K)LNQGLLTLSFQNR(Y)
(K)VLVELASILTK(T)
(R)VPTYSVYR(H)

(K)SSSFLSGGFLFQPVNR(A)
(K)VLLDGVVIIR(S)
(R)FTDRDILLSGIYPDPLNSK(Y)
(R)GVNLPIILDQDTIDKINIR(I)
(R)ILTNAHVVSNSSYLK(V)
(K)GFHDFQDQILESVNDQTVR(S)
(K)LLYLYESFNK(K)
(K)NLNQYVNLVMR(S)
(R)ILFLDDEEMIRDLFR(E)
(R)LGAVDFFIKPFR(M)
(K)SAAAYYEDLENYMVELIQK(G)
(R)AGVPIIPSGIK(G)
(R)TETVLPGSFK(A)
(K)SVPVLGITGTGGAGK(S)
(K)SVSLSNGNIFQELMETSKK(V)
(K)VSLGQMTHALYEVGGQYR(R)
(R)FITAASLFDGHDASINIMR(R)
(R)FLIDFPNK(T)
(K)ALINLIQK(G)
(K)IKPGDVIIIR(Y)
(K)ISITGPAK(V)
(K)NLLQVEISQEEIDKR(L)
(K)SEALHPSGPLVILK(G)
(K)SWKPIEPR(Y)
(K)TPHLADLKPGGK(Y)
(K)VGGVHGVMK(Y)
(R)EMLAVTSALVGK(G)
(K)ILLASGPQEPR(T)
(K)QFIIVLR(Y)
(K)GREEPVDVYELGK(I)
(K)IPETQIGIGLHTGEAMTGNVGSEAR(K)
(K)KPTMDPLSYEILK(S)
(K)LLVTQAVYDDIKDTVPGR(H)
(K)NAVEASLELLK(K)
(K)NESFSEFKR(V)
(K)NKVVGMFGQYVSPDVVDR(L)
(R)SLISAMSAVQEKNK(V)
(K)FIVGIPENK(N)
(R)LFVHGVTLIQNSGEETPLTLNQDGK(F)
(R)YILDVDLLSHQVTR(K)
(K)NVYPLIVR(K)
(R)YSEQPFTLASGR(K)
(K)AVALGLFDYLMYSNTK(G)
(K)SLLEISVSK(E)
(R)SWNSLIQLLPHTENK(I)
(R)SYYQNMEK(Q)
(R)TATHDQIR(S)
(K)LQAEIQTITR(K)

(K)VGVGILAAAVDTILSK(F)
(K)ENVLNLLK(K)
(K)IFQELSIFEPFGHENPIPLYSIK(N)
(R)EWFYQEFLK(Y)
(K)ENMHLVPAIK(N)
(K)FAEILGLNIEK(T)
(K)FLETNIGK(R)
(K)FLETNIGKR(L)
(K)IVHILFR(G)
(K)NSLELIFR(R)
(K)VIEVSTFR(S)
(K)RGDLIGLVGSTGK(A)
(R)ILTYEVIPEFHLK(K)
(K)FLFNR(E)
(K)YIYPLTHWYGFPGR(M)
(R)IPVPFLNPLFK(F)
(R)LLVEFLR(V)
(K)IDKPASLTSETFHQFLK(V)
(K)ITQISNLTK(E)
(K)LETLALSNNR(L)
(K)LSEFPDFLLNLPSLK(S)
(K)LTQEVIIQTLK(A)
(K)NNQFIQIPEILK(E)
(K)NSTSTETSVSQTK(S)
(K)SISQLESLK(N)
(K)SPIGIFDIR(W)
(K)GYIEANAPAPPK(G)
(K)KGYIEANAPAPPK(G)
(K)TAVSQLPELAPFLEK(L)
(K)GFVSTEFLSK(K)
(K)KYVLISEGK(L)
(K)LFGSVHTDTQGSR(F)
(K)VLFQLEK(G)
(K)LLSPFFTFTSR(G)
(R)LLHNILPVSIAAELKK(N)
(K)EFSIPIFGMEPAIKPAILQNPGKK(V)
(K)EFPWPGGR(C)
(K)IFGFMR(R)
(K)KLEALGLQITYENIGDPILK(G)
(K)SWAYTATQGYEPTR(K)
(K)SWAYTATQGYEPTRK(F)
(R)NIVHSGADALIYEIR(Q)
(K)LLLSNHE(-)
(K)VITIQTLR(T)
(K)LLFDLPGIQIR(D)
(K)SSPIPVNLDLK(S)
(R)ILQLIDVIR(F)
(K)FIIDLK(K)

(K)FIIDLKK(V)
(K)LASIFEIYK(T)
(R)MVFTNLNSDIENLMEITK(L)
(K)LLTAHVFYR(I)
(R)KEDLEILFVNDGSR(D)
(K)NSILLGTTSIYK(R)
(R)ILLPDLQTK(D)
(K)FALFFEELNR(N)
(K)IWELENKFEIWK(E)
(K)MLFVIK(N)
(K)VHHDVIAFLTNMNSYIGPAGR(H)
(K)VLLALIEK(G)
(R)ANVNVGLQDVALWHER(D)
(R)DRHAFYLSVLGVTASSLDR(M)
(R)SHGIHAEPMTLGLK(F)
(R)VVLPDSTIALEYILDK(M)
(K)APNVLIEFSGGITPEK(L)
(K)IEGSLVQILR(I)
(K)LEALSEFSGAGVSMGYLTHTTR(F)
(R)FLDLGLDIERH(-)
(R)ILLNFIQYLSGISTTTGEVVK(K)
(K)YYEHIIGHK(H)
(R)ILSIMVSAGLLQEHNFGK(D)
(-)MEITR(R)
(K)LTSFFEIFDNESEAVSAFK(-)
(K)VSYIDSSGIGALISSLSNLK(K)
(K)VSYIDSSGIGALISSLSNLKK(Y)
(K)YYTIINLEK(V)
(R)KVFELTK(L)
(K)ASEENLTETKK(S)
(K)IGEDGLPEGWKR(Y)
(K)IKEGYSEIDDIRK(K)
(K)IKNPNLIYPK(Q)
(K)IYAEK(Q)
(K)KKPATEEVEAIEENR(K)
(K)KPATEEVEAIEENR(K)
(K)NLLTAHQK(A)
(K)NLNDADKYAEGKDPEVSETR(N)
(K)QVLLIPPR(K)
(K)SALYAR(A)
(K)VLALSQK(Q)
(K)VLALSQKQQLIDSFADIDK(N)
(K)YAEGKDPEVSETR(N)
(K)YAPSELEEARK(N)
(R)KPTTHSSEDSEAGESNNK(K)
(R)LDKVESAPSR(K)
(R)TLESYPK(E)
(R)VAAEDLYSSEKYEDSISR(S)

(K)FATSIFLKKTSGNPLAIIQLLGICQK(K)
(K)MNRIEEAVLAGLSGMRR(F)
(R)SSAIVYSGYGmIINQVLK(D)
(K)EALELIQVSFSDPEVYKK(V)
(K)NGITVVDKEPVVTEDK(L)
(K)NGITVVDKEPVVTEDKLPK(T)
(K)NSILNLPVSESSLVVR(T)
(K)VGIIYFSNAEEYFAYPQEFK(N)
(R)GNLLGNITLTGIGNTLK(K)
(R)GNLLGNITLTGIGNTLKK(I)
(R)SLQVMVEGGTVDKK(N)
(K)QPVTGISWYTAK(E)
(K)SGSWWWPAHYAR(G)
(K)TEKPHLMPTANVGTR(A)
(R)YGKPYLGPK(Q)
(K)FGFPSPGISVVK(Q)
(K)IAASVTSLNPEKEFEAK(K)
(K)IWIVPIVNPDGAR(H)
(K)FGISR(E)
(K)HYNLVPQGISADLIATK(F)
(K)INHIHTLGNSSGIVDGAASVLLASDEGIK(K)
(K)KSIIPVK(T)
(K)RGALASIHPQELSAATLNAIQER(N)
(K)SIIPVK(T)
(K)SIIPVKTEDGTIVDSDENPR(I)
(K)SLGIPIEK(I)
(R)EEADRFAESSQIK(A)
(R)GALASIHPQELSAATLNAIQER(N)
(R)IESTFEWLSDLAPVFK(T)
(R)YAVMSALWPNSVPGYTVNR(F)
(K)EITHEVAIHWDEENKHPEELINR(M)
(K)ISSFQAVQHMLADMSIGLETSR(Q)
(K)NEIAAYVIR(E)
(K)QWITGGGSAGAYTVFAYTDK(G)
(R)DTPGLTVGKK(E)
(R)QVTYLAAR(M)
(K)ANGAPSLYLMLNR(N)
(K)TIEEVIKDPVFQESGMVIEK(K)
(R)NFAGHDVNYLSLSGVLSQTGK(T)
(R)TLGYTVEEIETMKK(E)
(R)WVALGALEDMFFK(T)
(K)ISERPDPVPVQGEVLVR(F)
(K)ASNPQVMVQAYR(M)
(K)IVQFEAGENHPVR(V)
(K)LLADKFNKK(I)
(K)NVLFSLDSK(E)
(R)IAESLGYYDIIVSMK(A)
(R)ISEVFSPLVLR(C)

(R)YNQTPFGYQR(R)
(R)LGAPSEISVIQLK(K)
(K)IQLEAYSPLAHGQK(I)
(R)LGIDFVDLYLIHFPVTSK(R)
(K)STLFQVILGNVEADSGTISIPK(G)
(R)IQVR(T)
(K)GLALDGLASLR(R)
(R)AVSTRPWIGDVSFNVASVGAQR(T)
(K)SGGEFFTPQHVSK(L)
(K)LIDQGQVQILEVDPPYEKYR(W)
(K)TTAESMVLR(F)
(K)KHEDMGFHEGWGTALDQLVVYVK(K)
(R)IIDVPR(E)
(K)LIHSLFAKPER(S)
(K)TYTIQSGGK(R)
(R)NPVELMEFLSK(E)
(K)DKVPLAELAPELK(K)
(K)DKVPLAELAPELKK(-)
(K)SVFTWGYR(K)
(K)VEGWMNPR(C)
(K)DVSHTQFNAR(L)
(K)FVFPFLLLLNRPNK(R)
(K)GYLGNLVNENHIHDIGK(F)
(K)KPLLNVPFFVAR(M)
(K)RYESAPLIPVGDPR(L)
(K)VEQNLINFK(L)
(R)LDQALHHHQ(-)
(R)LTGGWEVISYSLPFIK(F)
(K)GLGNPFK(D)
(K)GLGNPFKDDLANLQR(G)
(K)IQGYSDGQIYHVVTAGWGR(M)
(K)SLIFLLVAVLFWNCESK(T)
(R)GAGNGTIVGPAPR(I)
(R)IGFPVPAVISPK(I)
(K)FALWIPSSSK(G)
(K)FEETSSGVFGLFDTPAEIISAAAK(T)
(K)FEETSSGVFGLFDTPAEIISAAAKTK(E)
(K)GYKEDEVLNFIK(G)
(K)VLHPDITTDK(F)
(K)YVEVVK(-)
(R)KVLHPDITTDK(F)
(K)AANNAHLQELPDPVTK(L)
(K)ALWSNK(G)
(K)APSEIPK(I)
(K)DALNGATLSALVSELGVSEENIR(K)
(K)GIFSIQQPAIRPLFNSR(S)
(K)GISSNDVLVLK(T)
(K)HYEISLTSAEEVVSK(G)

(K)IVPYVDLNK(T)
(K)IVPYVDLNKTDENSQYDFVK(H)
(K)LSLFETIAIGDGK(A)
(K)NAYILSENGVYSGISVTSLEK(T)
(K)NAYILSENGVYSGISVTSLEKTGK(K)
(K)NLLHQAALVVSLSDR(A)
(K)QNNLADVNLFIAAESVPTMTGSNADLR(L)
(K)QTIELPAQIQPGMHK(D)
(K)QTIELPAQIQPGMHKDAIAIAVGYGR(T)
(K)TVVVTK(L)
(K)TVVVTKLLDSPSTQSIIGDFLR(T)
(R)ADETALSSNVLATTTHFLESWGDAEVTK(G)
(R)AQNPATIEGGIEVK(S)
(R)EMHWLR(I)
(R)FNWMQHWYNDFGWK(N)
(R)LAIRPGDQTK(L)
(R)SLVVAGSLAATTK(D)
(R)TAAGAVGTGVGK(N)
(R)TGTTVTASER(K)
(R)VLEYLNVGPQVAYLTR(V)
(R)YLGLNPEVAVR(G)
(K)LTQSYNDNKPIEWIK(V)
(K)YVGYAPDQPIPFNHK(I)
(K)YVGYAPDQPIPFNHKIHAGDNK(I)
(K)GPILLHIITQK(G)
(R)LVWDVGHQTYPHK(I)
(K)ASGLGFANIK(K)
(K)DIMNRPNEK(R)
(K)FKLENVEDPGDAGR(T)
(K)FPQPPIEPVAEK(A)
(K)RIEILR(K)
(K)SLDLPQMDIGIGLNSGPAVVGNMGSSHR(M)
(K)YYANIIDIFK(N)
(R)IYELLDLVNEEDLKLLR(K)
(R)YNPPPGAR(Q)
(K)SLASGFIYYVTSYGVTGER(G)
(K)VISTYADGVIIGSAVQR(I)
(R)IDFIHLVTPATTEDR(I)
(R)SLAHPFSMDK(I)
(K)LFGVEAGGYSSDPGSHSATIQFGR(T)
(K)NYIGRPSPLTYAER(L)
(K)VVGVSSGTATLK(D)
(R)DWALNVSNTHYIVGSSIGPHPFPTIVR(D)
(R)IEGIIPALETAHAFR(Y)
(R)YSPEILHDALVELETTYK(K)
(R)YSPEILHDALVELETTYKK(L)
(K)FFESGEISIYNR(K)
(K)FRDEIRPR(F)

(K)GIEVGHIFK(L)
(K)LKDSELIGFPIR(I)
(K)SSAGLYFYLPLGLK(V)
(R)HDLSYALGPTHEESFSFLLKPLLK(S)
(R)KSSAGLYFYLPLGLK(V)
(K)FNPEPMQR(D)
(K)GEPGELLSTPPLKR(M)
(K)ILGER(E)
(R)KYPELNLESYQFASYDPELGQK(L)
(R)SSGIPTIGVEPFGER(R)
(R)VVATFSYPEQFQHWLSSR(L)
(R)GLEVFLISAILSFVSVIGDLLESAMKR(D)
(K)FLYTSILPPVDLLIR(T)
(R)AAQELFLER(K)
(K)AIQTSGLGLQPTNDGVVIR(I)
(K)TIDLVK(K)
(R)ANPSLVEDIR(V)
(R)IVIPELTGER(R)
(R)MLVISPYDK(G)
(K)RYSQISFMESINR(R)
(R)IVIFAGGTGNPYFTTDTTASLR(A)
(R)VQSAIEINSIAESYIR(R)
(R)YSQISFMESINRR(L)
(K)AITWLR(E)
(K)FGENITIAR(F)
(K)IGVLVELNSETDFVSK(N)
(R)VVSYIHGNGK(I)
(K)AGTVIVGGEITGNVIATEK(L)
(R)TSRPITEYGAISTVLGR(E)
(K)AIELTPDDGPSKK(Y)
(K)QTIQFYNEGLNLYK(T)
(K)ILPIEVK(F)
(K)TNHFVSPSVVHVSGPPGVLENLGK(I)
(R)VNIFASASNAGETLLLGIPIK(C)
(R)VSILFK(I)
(K)EIGIHTFR(A)
(K)STLSSSVVSQSSVFLGMDSGLHSGIK(Q)
(K)TFAVGSVHAAEFLVGR(K)
(K)TQIALIGASGR(M)
(K)VMGEISDIEIQDIHHR(H)
(R)AGEVIGDHTVYFFTPEER(I)
(R)KPGLYDMFAVLGL(-)
(K)ALVQLVESFQQGK(I)
(K)DGVDGILSVNPYYNKPTQEGLFQHFK(S)
(K)IQVVAGTGSNSTK(E)
(K)SIAEHSTVPVMLYNIPGR(T)
(R)TSVNLLPETVLR(L)
(K)VTGIGGIFFFSDNPQETK(E)

(R)DVNKPDEINYLQWSLFK(K)
(K)NSVFLESLADIKK(L)
(K)VLFLNNNQITILPNEIAK(L)
(K)SLNLSYNQIK(T)
(K)NGLNIQNVIVSHEATFQVK(R)
(K)TAYPNENYFFSGFR(M)
(K)MVHGFGEEALSDPIIQK(F)
(R)SATQNIPKPANSSETIPK(F)
(K)GIHASFPFHEESGEVR(F)
(-)MIELYSASTPNGR(K)
(K)IPFAINR(Y)
(K)ISIMLEELGIPYTVHPINLDK(L)
(K)ISIMLEELGIPYTVHPINLDKLEQK(Q)
(K)RPAFIK(G)
(R)LYSVLDRR(L)
(-)MQDKPTSTDLIESIQDFLMK(E)
(K)GIELAAIGR(R)
(K)IIANGSIYSLR(L)
(R)YIVKDPSLNR(I)
(K)DLGLWTPHLPK(S)
(R)TTLFLVPTNTPGYTMVR(E)
(K)ELLNFKPTVIR(G)
(K)GSGSFAVNFLDQLNLLSTEK(-)
(K)IASGLLINIGTVTSNDAITMK(I)
(K)IPWVLDPVAVGALGFR(T)
(R)ATASASAVFAIAGTR(S)
(K)FSIHPYK(G)
(K)GLVLGK(V)
(K)ILLYDPIR(D)
(K)LIDTIGPPGNKR(H)
(K)LSSPGFQNSSYR(I)
(K)NIALVEMAIPVYKDEK(E)
(K)SIPVFDKPNTIALSPDGK(Y)
(K)VLEMDKNIALVEmAIPVYK(D)
(R)YLVISDFLDHQIR(V)
(K)IPLNHFIR(W)
(K)LLVELK(E)
(K)VTESMIEK(L)
(R)DSLFK(Y)
(R)TNPGFSMK(I)
(K)LILENFEEAEDWR(A)
(K)LTATIPGFVPQSTR(F)
(K)NHILGVK(T)
(R)DVFDENTVPDNGGDQIEK(N)
(R)LDYFGWR(K)
(R)QLSIWALGR(K)
(R)VEVFPPHEYVVK(G)
(K)IFVPAPLKEPMQK(I)

(K)IVLIHFSK(R)
(R)EWGHTHLDEILNNLSSFK(N)
(R)FHPFLP(-)
(K)IPPLTFEFPEVK(V)
(K)IQAVTLGDLK(R)
(K)MGILHNFLR(F)
(K)TAELVYKGTVLGK(S)
(K)YFVSSSVK(T)
(R)FIFQFVDK(M)
(R)GLAYSSGSSTHFEK(D)
(R)IVLLTGDLQK(E)
(K)FVSVVHPSTPSK(I)
(K)KYFVPENR(T)
(K)LADVLSLYQLLYGDWR(E)
(K)YFVPENR(T)
(R)FSNLFSIYITNNQNADQK(K)
(R)FYSLLELHR(K)
(R)IKNQILGGYLR(A)
(R)KFVISNEDNYIYSK(N)
(R)LEIWAK(L)
(R)TFQMENGLNVLMMK(R)
(K)KTTTVMEESNESSGDEFIK(A)
(R)ALNLLIAEK(G)
(K)KKDDLQEVIHLLK(N)
(K)LESVIDVLVSK(M)
(K)SKIEPATGGTVR(M)
(K)GFQEELEKDR(Q)
(K)GNITHLFYNSPK(S)
(K)ILILSFIPGPDEIR(E)
(K)ILILSFIPGPDEIREK(L)
(K)IWVTVYTDDDEAEKIWLSK(G)
(K)TLELGLEK(I)
(R)EIFLNYFK(D)
(R)EKNLLVILGSR(N)
(R)TGEIGYFFIK(K)
(R)TLLGDHVLQK(G)
(R)VALLLQEVDSVYDTNELRK(I)
(K)IFSGAEHNLQAQKPVK(L)
(K)IFSGAEHNLQAQKPVKLDI(-)
(K)VIYEAVK(G)
(K)FTIDPNTVNSGNNDRDAK(I)
(K)GIIGLTLNGVTK(K)
(K)LWPEVELTISTK(L)
(K)TGVGGSFDK(I)
(K)TGVGGSFDKIEVVGTTTGNSFENTFK(G)
(K)TNLNLGDFK(A)
(K)VISVELSSDK(T)
(K)VISVELSSDKTSGK(G)

(K)AFSHYR(W)
(K)LAAEFIEAQK(G)
(R)EALENAKPQVEVK(S)
(R)KITPDPVYNDVQVAK(F)
(R)LALGIR(W)
(R)RLALGIR(W)
(R)VGGVTYQVPVEVRPER(R)
(R)LTTGIEVTAYIPGEGHNLQEHNVVLIR(G)
(K)ASLNTESFFSAASFQETTK(V)
(K)DKGGFNPVFIMAASGAR(G)
(K)EHPVMLNR(A)
(K)ENVIIGHMIPAGTGTK(K)
(K)FVAGIGADAIK(E)
(K)FVAGIGADAIKELLAR(I)
(K)GGFNPVFImAASGAR(G)
(K)GGFNPVFImAASGAR(G)
(K)GVTALQVYLVQEVQEVYR(L)
(K)KMALELFKPFIMK(R)
(K)KPETINYR(T)
(K)KVEAEDKEVFDVLEYVVK(E)
(K)MALELFKPFIMK(R)
(K)RGDQIDDGNLDPHDILR(V)
(K)RLEVLEAFR(D)
(K)RMLQEAVDALFDNSR(R)
(K)SVLYFEK(Y)
(K)TGTIVNVK(V)
(K)TVLMLDDIKK(L)
(K)VEAEDKEVFDVLEYVVK(E)
(K)VIEIWTK(T)
(K)VKVTIPIGK(Q)
(K)VLTDAAIK(G)
(K)VSYTGIVNNINGR(L)
(K)VTIPIGK(Q)
(K)YHEMGLPK(K)
(K)YKDIAVFK(S)
(R)EEYSVPVDAIISVQDGDKVK(A)
(R)EGLSVLEFFISTHGAR(K)
(R)FRQNLLGK(R)
(R)GLMAKPSGEIIELAIR(S)
(R)IAVISGGSR(E)
(R)IAVISGGSREEYSVPVDAIISVQDGDK(V)
(R)IKEWSYGEVK(K)
(R)ILGEEAVIPVK(T)
(R)KVLITDSGDTSFVNQQIDR(L)
(R)LGIQAFLPVLVEGK(A)
(R)LIFNTILPEGYAYVNRPLSDK(E)
(R)LIFNTILPEGYAYVNRPLSDKETNR(I)
(R)LIPAEIGEAVGTIAAQSIGQPGTQLTMR(T)

(R)LIQQYKTEELSNLR(V)
(R)LVFNEENKR(V)
(R)mGHIELAAPVSHIWYYR(S)
(R)mGHIELAAPVSHIWYYR(S)
(R)MGLLLDMTVNQLK(S)
(R)MLQEAVDALFDNSR(R)
(R)MLQEAVDALFDNSRR(K)
(R)NTLITR(E)
(R)RGSIVIQR(L)
(R)SHNDFESITIR(L)
(R)SVIVVGPELK(Y)
(R)TFHIGGAASAK(V)
(R)TSNILLK(V)
(R)VKGVTALQVYLVQEVQEVYR(L)
(R)YATLFAPTISIEDIR(V)
(K)EKIEVTGENGELKEYVLQIGSK(Q)
(K)FEFNNPK(E)
(K)FHSLMR(Q)
(K)FPATLLVK(S)
(K)GIHSIKPGIPESFNVLVQELR(G)
(K)GKDNPILINALEKDGVNDYEDAILK(F)
(K)HDGVVTSVDAENIVVER(K)
(K)IEVTGENGELKEYVLQIGSK(Q)
(K)ITNLDYLPNLIQIQK(R)
(K)KFPATLLVK(S)
(K)LGPEQITR(D)
(K)LLHSIFGEK(A)
(K)RGSTLAGQVVVGEK(L)
(K)SLGHGTNEEVLR(L)
(K)VKEVELIEFPK(G)
(R)ALMGSNMQR(Q)
(R)DDVFSSIHIEEFEIQAR(E)
(R)DVHYSHYGR(M)
(R)ETFPIESPNNDMIMEYSHYILGEPK(R)
(R)GSTLAGQVVVGEK(L)
(R)GSTLAGQVVVGEKLDEMGNILVK(G)
(R)GSWLEFEMDNK(G)
(R)HQGLEAVFR(E)
(R)HRGDFPFR(N)
(R)IGAEVKPGDILVGMVTPK(G)
(R)IVRDDVFSSIHIEEFEIQAR(E)
(R)LFFSPK(T)
(R)LNALGPGGLSR(D)
(R)MNLGQIFETQLGFAASK(L)
(R)MNLGQIFETQLGFAASK(L)
(R)NVLAAFMPWEGYNFEDAILISER(I)
(R)RLNALGPGGLSR(D)
(R)RTINDIINMETGEVMLEAGSK(I)

(R)SFDWFLQADVK(D)
(R)SFDWFLQADVKDETK(R)
(R)SFDWFLQADVKDETKR(R)
(R)SPGIFFSYDmER(D)
(R)SPGIFFSYDmER(D)
(R)STGPYSLVTQQPLGGK(A)
(R)SVGELISNQLK(T)
(R)TINDIINMETGEVMLEAGSK(I)
(R)VLRPADIIETVR(Y)
(R)VVVSQLHR(S)
(R)YILNLFSETENYYPDDIDHLGNR(R)
(R)YILNLFSETENYYPDDIDHLGNRR(I)
(K)GLVEAGGK(A)
(K)IEVIK(L)
(K)KFEGAGAQIELK(A)
(K)KIEVIK(L)
(K)KKFEGAGAQIELK(A)
(K)LTLVEAADLVK(K)
(R)EITGLGLADAKGLVEAGGK(A)
(K)DKNITFGSEYQGPLAAIFAK(D)
(K)IEAVALLK(G)
(K)NITFGSEYQGPLAAIFAK(D)
(K)NITFGSEYQGPLAAIFAKDNLPAIAK(V)
(K)NNLFLR(A)
(R)AIQATAEK(N)
(R)EQLLAQIAGGINGPAR(T)
(R)TIASGINQIIASLAR(A)
(K)FFSIDK(A)
(K)FFSIDKAVELAK(S)
(K)GGVVHLGVGK(V)
(K)LIENIR(T)
(K)SLQNIR(G)
(K)TFSISPTMGVGVK(V)
(R)GTISLPHGTGK(K)
(R)TVVQTLMR(D)
(K)AIGLETGSATPHTHK(V)
(K)LPVVITVFSDR(S)
(K)SPPAALLVK(K)
(R)SFTFITK(S)
(K)IIDGPFANFTGLVDEIFPDK(G)
(K)IIDGPFANFTGLVDEIFPDKGR(L)
(K)KWYALQTYSGHENK(V)
(K)VEIFGR(S)
(K)WYALQTYSGHENK(V)
(R)FLIQSLPSVSTFVGSK(D)
(R)VKVEIFGR(S)
(K)GIQGIAALVLAPTR(E)
(K)TVAFLIPVIHNILTK(G)

(R)LIDMIK(S)
(K)IIYLGLGETSK(I)
(R)LVLGDVLSYVGK(K)
(K)GTVLYFYPK(D)
(K)TGFNVVGVSK(D)
(R)EFMGIVR(T)
(K)LFIMDLIAR(T)
(K)WIGSVLDSASNLLHLK(L)
(R)AGDSIEYELPGTLDEILPFLK(L)
(R)LAHFILPIAGK(G)
(K)DLLITMKDELR(N)
(K)GISENITVLSIVGR(F)
(K)LHVDDLFYGMTVR(E)
(K)MNSLVDPHIILSLYK(A)
(K)MNSLVDPHIILSLYKASQAGVK(V)
(K)MPTLNLLSASPHNLK(S)
(K)QITEDVATIFNTITSYAK(M)
(K)SLNLAMVLSTDDEK(T)
(K)STFISLIDK(E)
(R)FFPLEEIIK(L)
(R)FLRPDGHYVK(K)
(R)IEVLFPIIESK(N)
(R)ISSEDPKVLGIK(M)
(R)RIEVLFPIIESK(N)
(R)SLNGDSASEILSDLSDKVR(V)
(R)YVHLGTGNYNSTTSK(Y)
(K)EFLEQTKPVDSMEVLR(R)
(K)GSGAILFGPSTIGGVVNFITK(R)
(K)LPIDLIYTR(A)
(K)SYDTEPFVNRPGDTVYMLDSVGHR(N)
(R)IPGSATVIEK(E)
(R)NTGSNFNTIPSNVIK(S)
(K)LLGVPGR(R)
(R)NWTQPDVVR(G)
(K)SLSYLVQFQNSALDAR(V)
(R)VGVPAGLTVSGTTQYPTK(L)
(K)GEMIAFQGGHEIPDVVLK(G)
(K)GMNLYLR(N)
(K)KLEQLLIGAGWK(G)
(K)LEQLLIGAGWK(G)
(K)LALSLGKPK(K)
(K)LGFQESDYVPLLKPLTGNPDYK(E)
(K)TTLVSLGMQMSSAALNR(F)
(K)YGGSQYLNLLEDLLPADIQDAR(L)
(R)YGLGGVLLR(E)
(K)IVGMMPHPER(A)
(R)SGAMAGFSPVMK(S)
(K)MPPAPNLPAELIQELR(E)

(K)SISIFLR(A)
(R)ISAFDVVFPQLVPDKGK(V)
(R)NYLETTSWNK(M)
(K)LLPILFITGINR(E)
(R)FKLLPILFITGINR(E)
(K)HSLLVILRPELEK(V)
(R)IDIMIALYK(D)
(R)KNLYQIDI(-)
(R)LIFTPPDVLASQVK(L)
(R)NVLSFSGGELQR(A)
(K)LGGAIFADFR(Y)
(K)LWSLNEMEK(T)
(K)LLGAIAITAPR(F)
(R)FPLLK(E)
(R)GPEVVAGITK(L)
(R)LEGIWHL(-)
(R)RLEGIWHL(-)
(K)LILHHTGIPAGEIR(D)
(R)ETIFDAWTIPEQLMR(W)
(K)DFETGLNNIKTIVEEK(-)
(K)IFTFVNDYHNWPSWSPWEK(L)
(K)LDFISPFEAHNTAEFTFVAK(D)
(K)TNVTWAMYGPNAFISK(L)
(K)ELPEDIALSLLHSAK(H)
(K)GTLGAAVSVAK(E)
(K)ISTEAMYLMMENAFK(L)
(R)LGLSFEGIFR(Q)
(R)MLNYITPDFVHVFAK(G)
(R)NEILQMSLLKPK(L)
(R)SILLITHYQR(M)
(K)SEKPVETTSAPIATETASTLSPELQK(G)
(K)TLNNGIPNSPMVAYK(F)
(K)YVVHLSQN(-)
(K)NVALVKPPPPLANYNFTEEFR(I)
(K)SKDELINIEDQLDLR(E)
(K)GRLEEMGMNIESLPSQTTGK(A)
(K)GVITDPDSIHFSYIHSPMR(Y)
(R)WYLPLFPTAIESLDLK(G)
(K)LAAPSTEHLEPIFYVLGAMKPEEK(V)
(K)YRPSGSTELAQQIQK(L)
(R)IWLLR(N)
(K)AIVALIDSYSR(E)
(K)GTILLLVGPPGVGK(T)
(K)SRPVFPGIITPLIVPSGK(F)
(K)VLLFLK(K)
(K)VNLPDNAVNILVNTIR(R)
(R)ILEFLAVK(K)
(R)NYLDILESLPWEPAPVR(E)

(R)TLLVMTR(E)
(R)TYIGSMPGK(I)
(K)AAIIVHLYGWGSSR(L)
(K)EIGFGNIYPGAMSDQPGAKPYIK(G)
(K)FKGASIYK(D)
(K)RFEPGLLEEWIEK(V)
(K)YYDILPNLGIQVIHPPK(D)
(R)ISHYAYGDVGWNSR(L)
(R)LDTLQAAFLNLNLK(H)
(K)GETIVEQEMSIFIR(K)
(K)KAGYEDTPAPPTYPTVIQFWGYPK(I)
(K)YTYLKPIYPGR(V)
(R)ILHLK(E)
(K)LITVAVEATDDSGEVK(C)
(K)MEVGQELPPLKTEVITHANLVR(Y)
(K)TEVITHANLVR(Y)
(K)TGLDGTIAHGMYVMAQLGR(L)
(R)KTGLDGTIAHGMYVMAQLGR(L)
(R)YAGASGDFNPIHNDPDFAR(K)
(K)LIFNLNNSLNALSGLTLK(A)
(K)NILGILWGK(L)
(K)NLFSIWK(Y)
(K)NGYVQTLTGR(R)
(K)VVNFSVIYGVTPYGLSR(N)
(R)EHLGPVLSSQVPK(V)
(R)IHTSYNQTIAATGR(L)
(R)KPMPEDLRPQIQEVMDTLEK(I)
(R)YMTQYPGVK(S)
(K)AEHHDHTGEYPLAVLK(K)
(K)AWEIGLMNIHIEPR(F)
(K)IAMGAFDHTRPGVAIGAVGVAR(A)
(K)IFQIYEGTSQIQR(M)
(K)TPGIIMGK(K)
(K)VPAWQMIGR(E)
(R)AAMEHSIQYANTR(N)
(R)NTFGRPIMENQGISFMIAEMAR(D)
(R)NTYQASIAK(M)
(K)FKPDVPPFQVYR(G)
(K)IATGSLELNPNIPDEEYKK(N)
(K)TVYDLIR(A)
(R)AALPAGTVSGAPK(I)
(R)LPPFAGGAVGFLSFAAVR(Y)
(R)TVNPSPYMYYLK(L)
(R)AVNAAQGWEIYSK(D)
(K)GILTVFR(A)
(K)NIIEAIGQNTSLYGTYSFIR(I)
(K)FLKEEIK(S)
(R)SNIHITLYAGIGPDTKPLKK(S)

(K)GAGSLPTASSVVSDILYYGAR(R)
(K)IIDGLSLIR(H)
(K)ISILGSLAFSEK(I)
(K)VLGDHGVSISSVR(Q)
(R)IGLIGAGTVGSGVIEILK(K)
(R)IGLIGAGTVGSGVIEILKK(E)
(R)QNETEKEPVEVVVITHPATEESIK(N)
(K)IVIGGLNHFLENVIR(L)
(K)TQSSIIPNLLEAMTANK(E)
(R)IGFAEPLVGLPITSALIIR(M)
(R)IPASVTVETK(R)
(R)IPASVTVETKR(A)
(R)QIMIPSSVSEAAMEGALYKPSDAVR(N)
(K)EYPFEILPFTHTDK(G)
(K)IRYDLILTDNR(M)
(K)LVQEIAFFLK(E)
(K)VNPEFFYFK(S)
(R)MTPIIFLTVSNEK(E)
(R)MTPIIFLTVSNEKETIIQAAHNK(K)
(R)YNFDVDHVVDGVAAEIK(L)
(K)LAFISASPR(Y)
(K)NADKDIFIPIVSWLDKR(N)
(K)TSDDWDLTLEHFLPALGTTSK(K)
(K)TVIQLQGEIHHPK(T)
(R)DAGYPSLFFGDKK(F)
(R)IANLVTIGSSGILDSPSASIK(S)
(K)TVLETVGDLAGNILSPHVADLDR(E)
(K)TVLETVGDLAGNILSPHVADLDREGLK(F)
(K)VIEAGVQAYGFSR(K)
(-)MISTSGLTLNFGK(K)
(K)IAIIGTNGVGK(T)
(K)IKLDDVKPSSR(V)
(K)ILFENVTVK(F)
(K)SYDRPIFK(D)
(K)TIHWLEEFLTNYR(G)
(R)DAIYSKPEMSDEDGIRVSELEEK(Y)
(R)EGILEDADTLVDWLYR(Y)
(R)LIIGR(M)
(R)VLLAQVLFQKPDILLLDEPTNHLDIK(T)
(K)NEVTNYYEVSQIRPIR(I)
(R)GMNVYTTLDYDKQEAAEK(S)
(R)IAFAQSINTIAVK(L)
(R)TDNLAEHGNIIWALLSSEDR(E)
(K)FTLRPAR(V)
(K)SLVSGFLNPIDNLER(V)
(R)SAQEFVSIRK(E)
(R)VGATQTNSEELKPFVDGVTMILK(E)
(K)ASDINEVILVGGSIR(I)

(K)AVITVPAYFNDEQR(Q)
(K)GETLIGQFAK(N)
(K)HLDMTLTR(A)
(K)IELSGTMSTQINLPFITADASGPK(H)
(K)IRIESSSGLSEDEIQK(M)
(K)NQAITNAVNTIR(S)
(K)NQTGIDISADKNTVQR(L)
(K)VVDADYTVVDDEKK(-)
(R)AKFDQLTK(S)
(R)DAGLKASDINEVILVGGSIR(I)
(R)IAGLEVER(I)
(R)IINEPTAAALAYGFDK(K)
(R)IINEPTAAALAYGFDKK(N)
(R)IPAVQELVK(Q)
(R)NTTIPTK(K)
(R)SGNEGVKFETSAGEFTPQEISAR(V)
(R)TTPSIVAFTAK(G)
(K)GHGMPYLGAYGK(G)
(K)IPPGVETGSR(L)
(K)ISLAQAILGAEIEVPTIDGK(K)
(K)KHELFER(Q)
(K)VSGEGEAGPNGGPHGDLYVVTHIK(K)
(R)SYYDILGVSKSANDEEIK(S)
(R)TINIK(I)
(K)DNPLKQEHEHFIR(C)
(K)HVLVEKPIAETTNQAK(E)
(K)LGVIGTGHMGQYHVNVAK(T)
(K)NLVLLVGHVER(F)
(K)TDAVVIAVPTFLHHEIAK(K)
(K)TDAVVIAVPTFLHHEIAKK(A)
(K)TLAFSTIDELISK(T)
(R)FNGAVLELSK(I)
(R)NSDVSTLEIAYQILSEIHGTSNK(-)
(R)TLNITQK(D)
(K)FHVFQGYSGWGAGQLETEMNRK(S)
(K)ILISNSSIVMDYFNR(T)
(K)ISQPGIEIIPGLYLAR(S)
(K)SWVIHEASK(D)
(R)SFDTLLELLK(S)
(K)LIVAPVIGAR(L)
(R)EYFLSYR(N)
(R)RPNLFVLDIR(A)
(K)KVFGLFmISLILSR(F)
(K)GPSGVLLQAFEERPVR(L)
(R)NVVGEFIGIAAGK(G)
(R)SGAVVALVTK(Y)
(K)ADLFGLYNDPHTGVALAALEK(L)
(K)AGIPAIIFLPAGK(V)

(K)DLGMTVLLSQVK(Q)
(K)FPNSSGIWNK(R)
(K)FPNSSGIWNKR(E)
(K)GESVVVISTAHGLK(F)
(K)IVNSIHKVEPK(A)
(K)NFESFEPVSAK(T)
(K)TISVEITQQLGWK(V)
(K)TTLASAIQIGNPVSIQK(A)
(K)VSQAQLIQPVSNGAK(V)
(R)EAGIYLANSMNSLR(I)
(R)EWVLPHIEDK(N)
(R)IVLAQAEHANPLYLSYLK(N)
(R)LTESLGLGSFYVK(Q)
(K)ALDVFGPGTHTLK(T)
(K)LLKPMVVTR(L)
(K)LLKPMVVTR(L)
(K)LVNLPFGGQTPFTAEVVYVNK(S)
(K)NGVPITQISQHLEEASSAGK(T)
(K)NGVPITQISQHLEEASSAGKTK(T)
(K)SFVNTVVGTQHK(F)
(K)TGNIPVLEK(L)
(K)VLTDKFEIETMGNMYQQK(R)
(K)VLTDKFEIETMGNMYQQKR(M)
(K)YHITLGVR(A)
(R)LSDFISEVVLK(N)
(R)NDISYFGQLVVNESQEAVFFK(E)
(-)MQQETSTGQNAVGQFLAQSPVQNLISR(Y)
(K)IQILGINSNSSIQEVR(D)
(K)LIHTIQSAGYGLNGLGTGLK(A)
(K)LPLYSNALK(D)
(K)NIGNIEVFYGIFQEK(-)
(K)KLDLNFR(K)
(K)LDPLHEAFFLPGTGFAK(E)
(K)MPMILPSQSK(T)
(K)TIAPSLSMIPMTAAK(G)
(R)ILINDSPLQETFLKPGFK(T)
(R)IPPGYFLMLGDNR(E)
(R)VVGLPGDYFR(F)
(K)GFGFVEMPNKEEADK(A)
(K)VNVAKPK(N)
(K)GAELLAK(L)
(K)GAELLAKLAPIQGK(L)
(K)KGAELLAK(L)
(K)KKGAELLAK(L)
(K)KPDAKELLAK(I)
(K)LAPIQGK(L)
(K)GQYMIPYEVLDHLSTSR(I)
(K)NQNYLFALIPSLPIK(E)

(R)SYVFHPYNLVK(D)
(R)TDHETGNVAAVMDGDIEPFILAYLK(T)
(K)NLLPEILDMAMR(L)
(R)ELNFIWHNVSDHVSPAYAVGK(D)
(K)AVHFYEQGLGFPK(M)
(R)GFTIAYNGQSK(E)
(K)LRHPEVIGIGLGGAELMGPAR(D)
(K)SRLPADRPYSSSLQISFDK(Q)
(R)ANNILYTEVFFAPSK(F)
(R)HPEVIGIGLGGAELMGPAR(D)
(R)IIDRDVTELNR(L)
(R)KEQNHPVR(Y)
(R)LPADRPYSSSLQISFDK(Q)
(R)VVAHSGEDDGPWAIWEAVELLK(A)
(K)GLNIQAITLLNGGIFPESHRPK(F)
(K)ILAHDLGDTVTQELLAR(F)
(K)LANQIIFHEINQGK(G)
(R)IFEVGISYYGR(T)
(R)NILETVK(K)
(K)AAESYQNLVFGIALEK(I)
(K)AAMTLFIEYTSLLDK(A)
(K)GAISEEYTLPLSFLSLTLEK(I)
(R)DSLSGIAHILNVMAATGK(K)
(R)GIIPTGLSPEVIFNSLR(A)
(K)GGYPHYMIK(E)
(K)GHVFQSLTDTEVLPHLLEESK(K)
(K)GHVFQSLTDTEVLPHLLEESKK(N)
(K)GKGEYFLASDISPLTR(N)
(K)ILIEEIK(L)
(K)MVSNIQEIK(A)
(K)VVSLVNNVNSTIAR(E)
(R)EFPAPGNVGIGHTR(W)
(R)IIIQAAGTSYYAGMIGK(H)
(R)KGIIISIVTEGDQEAK(S)
(R)TYNHPVAMEGALK(L)
(K)IFYNQANLSALK(I)
(K)MVNAVSTSIVPK(Y)
(K)YYGTTLVTGFAK(I)
(R)DILGITVYANHSQK(L)
(R)ISVMGGEQAANVLLTVK(M)
(R)NIVSTFHHAGNVTQR(G)
(K)IFITPLEDVIR(I)
(K)LIVAIIQPHKLEEVK(S)
(R)LEIAVNDEFVKPTVDAILK(A)
(K)ILTAIPSFALEEQK(A)
(K)KGKPEYIEK(I)
(K)RPIQVSPELISYLK(E)
(-)MNYWLFK(T)

(R)ANPLSVVGIAEVVKPGYPDHFAFDPSHK(Y)
(K)ALLAKETISHDELMTIVHPANEEGTK(K)
(K)EELVEIIEFLKDPK(K)
(K)ETISHDELMTIVHPANEEGTK(K)
(K)EVIAYHEAGHAILGTLLPYTEPVHK(V)
(K)IQIEK(D)
(K)IQIEKDVIEGYYIPSEYADSK(T)
(K)SFFISEK(E)
(K)VMMGPER(K)
(K)VPMTSDISLHSIAR(G)
(R)ADVLDPALLRPGR(F)
(R)ALGLTQSLPKEDK(H)
(R)GTPGFTGADLANLINEGALLAAR(K)
(R)IPTGVLLVGPPGTGK(T)
(R)TTVAPLDK(D)
(R)TTVAPLDKDLISSLR(R)
(K)ILDVIANNINVEIK(T)
(K)LIVGLGNPGDR(Y)
(R)IGIQNDGFNLK(N)
(R)SISLGDIDDVIEKYHL(-)
(R)SPNFIR(I)
(K)IGIETTGIAK(G)
(K)TGGQFEHIIHEIR(V)
(K)TIPEDLLENLTIDVTDLDVGDSIK(I)
(K)VPTSWEILINGDPIVTSVNK(T)
(R)SSGMVPVNIIGGGVATSGAVNEK(E)
(R)SSGMVPVNIIGGGVATSGAVNEKELEK(M)
(K)ANVSEVMNVIGEIEGK(N)
(K)SLSVAPLFANAIQR(I)
(R)ASVSSISVVIPYYGYGR(Q)
(R)IHTNQSVSTLFD(-)
(R)ILTMDLHADQIQGFFR(V)
(R)INSTQFSEVVLANTIAIPESK(K)
(K)ENEFSATILSAVVEKPTGYGR(I)
(K)LKNHLESHGVNSPEDLQMLSALIK(G)
(K)NHLESHGVNSPEDLQMLSALIK(G)
(K)QHKENEFSATILSAVVEKPTGYGR(I)
(K)VAVELNGKPLLLHVLDHLK(G)
(K)VAVVLAAGK(G)
(R)NSSGEVTAIVEEKDSSTEEK(L)
(K)SGNLNLNAVGWSK(K)
(R)FVPEFHR(K)
(R)FGSPNIEDFAVSIFK(L)
(K)DKIPADQNSSGALIGFYINQLK(I)
(K)IPADQNSSGALIGFYINQLK(I)
(K)SPELTSVK(L)
(R)YYFTVYALK(D)
(K)AVFLMVYK(Q)

(K)DVDNVGGGQRPFTLTVSGQK(R)
(K)EHMIPASVVSNR(L)
(K)IGYSGQAESFK(E)
(K)IVSMFNTNR(S)
(K)MNTTQFK(A)
(K)MSQIALQVDQK(I)
(K)NSILLVDFTNQLLSQGK(E)
(K)QSGSNTVAVAQSVK(K)
(K)VGNHAHNSSESIPEITTK(A)
(K)YAEQQVR(D)
(R)DLKENFYSSFVPNFNNR(L)
(R)EKEVLLGVPGTIIGTELR(T)
(R)LASGGMNIPAGK(V)
(R)LIPIQNVAK(A)
(R)LNRNKAVEIYADVNPK(G)
(R)RIDPADQPIVIIALR(A)
(R)TGAPEFR(V)
(R)TINEFQSPEEIRDTPISLFGNEVPIK(I)
(R)TLVEGTTPAVYR(E)
(K)VLPVSNPPTEK(Q)
(K)IKDLNPFIDAFLDR(E)
(K)SMTENIHQIILLNR(L)
(K)IFLVDDHAILR(E)
(K)KLDLHK(V)
(K)LLELGINGYVLK(D)
(K)LLELGINGYVLKDDAGNDLLR(A)
(K)VADLVMYAIR(A)
(R)TIILSR(H)
(K)FSGSAANQVMGYHGTIGYNFHISGSK(Y)
(K)IQLFYQVIHTNTAK(N)
(K)KEFYEDNDTGLIYTKPGPNR(T)
(K)LSLGAAVAQTTQLK(T)
(K)YIMPVFK(G)
(R)IQYNVFGGLIK(K)
(R)ITLGQINVPFNR(E)
(R)LENAINSQFLR(V)
(R)NSAFSGNEDFHTADANFR(R)
(K)GILIEINSPGGTVGASQEVYNELMR(L)
(K)IYSGQDAFR(N)
(K)SVQNLAEGK(I)
(R)TTGGTFFSTAPIGAALIK(I)
(K)EHNTYLIAPLR(E)
(R)GVNMGHDVHYSGTVGAAR(H)
(R)SATSMALSIYDSMR(V)
(K)EYLGWLYLPK(E)
(K)GIFPASMDFTTDLHSLGQYIQEGKR(I)
(K)NMFALLNKPGFEQEGETLR(K)
(R)AVLEATLPFFK(K)

(R)FASSFIQSSK(L)
(R)LLWELLR(K)
(K)ELETFSLFPLVFSEEKK(E)
(K)QNAEVAIINIHYLGEKIR(K)
(K)ALGSYFYLIADKK(M)
(R)SYKALGSYFYLIADK(K)
(K)AIQITWSQLVK(L)
(K)IYQPYFTTK(G)
(K)NHHPVVSVLDDGVGMDEEVIQK(I)
(R)LIFPNKV(-)
(K)SILMKLIYGSFLNYYHWK(K)
(K)IMLDLLK(S)
(K)MIPVPGHTQGHSVLLYK(N)
(R)FLFTGDHLAYDPLKDR(L)
(R)FVSSLADKIESLGGIR(Y)
(K)GLNSIVIGPDSLLFQR(I)
(R)LIFEFR(T)
(K)AAILNAIVNIMK(V)
(K)FTVAQALAVIDHVDAIKPTIEEYK(K)
(K)FTVAQALAVIDHVDAIKPTIEEYKK(L)
(K)LAVRPLINALR(G)
(K)SLENHPYLK(F)
(R)KDLASVLGFK(L)
(R)KETVNFLVSTLEK(E)
(K)ESISILR(E)
(K)MSHDLEAPIPGATEIR(T)
(K)TSIIGELSEVFENPLPVK(T)
(K)VLPGHEFYEK(F)
(K)EGLALIEEILYQTVK(F)
(K)KVETSENPVPEER(L)
(K)STSLETINPSHLK(T)
(K)VLPYEMISK(R)
(K)VVVVANLKPR(K)
(K)YFYVWLDAPIGYMASSK(N)
(R)FLAIYLYPVVPK(I)
(R)LEISIDDLSK(V)
(R)VGQIVEAGPVDGADKLVNVK(V)
(K)IPILK(I)
(K)LSIHGFTR(D)
(K)VNIQFSDFNLERPSLLFIK(T)
(K)IFITGASGFVGEATTR(T)
(K)TVLPILLK(M)
(R)LIWGPGDK(T)
(R)NFLESLLATQK(V)
(K)ILAFR(D)
(K)TIIAVPMIFR(G)
(R)LIAEIELLWK(I)
(K)FVGPITFEALTGK(K)

(K)MILDLYQR(N)
(K)NSASVIAIVPATANIIGK(M)
(K)IDQEEFQIIQNTFQFSEHQAK(D)
(R)MMHSPEELMIIIEEQNK(Q)
(K)GALDQAVSIMILR(R)
(K)ILIISENLSGR(I)
(K)MATEITFLAQSK(G)
(K)NYLPVVYGVR(E)
(K)SHIPVLSVFVR(D)
(R)EFTDAAIQYYAVHGHTMELLGFLSR(L)
(R)NLLIQFFK(N)
(K)DMIVSSPPSHALSEVLR(E)
(K)HLPNFTLPEK(L)
(K)TTQSAYELSQK(L)
(K)YHSYLSYLSGPSFAK(E)
(R)KVQEIFSFLYFR(T)
(R)LGKGETLEQILSSMNEVAEGVK(T)
(K)TLVALLTALR(Y)
(K)FYNWMFK(N)
(K)LLELFQAYQR(E)
(K)WHWSYAPISNK(L)
(R)HHWGTDVDLNALENSYFEK(G)
(K)HPVRPENYYGYTK(L)
(K)LLASSAMAQR(L)
(R)LLQWVPEYSEAK(T)
(R)TPANLLPIIMEAAVGIR(K)
(K)IHPTAIIDPK(A)
(K)TVIGDHNIFR(E)
(K)VIYHSGISTR(K)
(R)FHQGAVIGVMPQDLGFNQQLLTK(T)
(-)MLVDWNR(L)
(R)VNNIAHFTK(E)
(K)AETGLPYVTEVMDHTMAEEVAK(H)
(K)HADMIQIGTR(N)
(K)HIFIVGPDSPQTYEQTLTIAK(Q)
(K)IWNVSIPYK(N)
(K)KYNILDR(I)
(R)GAAVSDTIHFIGDTR(K)
(R)NTPDLNVITHVK(N)
(R)TGKPVILKR(G)
(R)TRPTDWR(G)
(K)FLIADLPFLSYQTSIEEGIR(S)
(K)FSNLHSIVK(E)
(K)GKEKISVVTCYDFSFAR(I)
(R)GAPDKFLIADLPFLSYQTSIEEGIR(S)
(K)IETSLNPEELLEVVLR(I)
(K)LTILTSNTSPAEWK(D)
(K)ALITGITGQDGSYLTEFLLQK(G)

(K)FYRPTEVDILIGDPAK(A)
(K)GYQVHGIVR(R)
(K)ITLGVANLLAK(K)
(K)KGGPIQLGNMDAK(R)
(K)LYAYWAVVNYR(E)
(K)SGQLLVEVNPK(F)
(K)VQAIPQTETTPFYPR(S)
(R)EAFGIHASNGILFNHESPR(R)
(R)FYQASTSELYGK(V)
(R)GNSNLVLHYGDLTDSSNLNR(I)
(R)KITLGVANLLAK(K)
(-)MKPYADLHNHLYGSITSEFLYELGK(S)
(K)FTISTDDPGIFGTSIKEEFSK(A)
(K)LVISLHR(D)
(K)EILILFGK(S)
(K)ILQMIGIENLFNPEER(A)
(R)AAASMAEQLSYQGVK(K)
(K)IQNFLQDIGNR(S)
(K)NTLNTVVPFK(K)
(K)NVGMVVFNSR(G)
(K)DFEIKPSSIPGIGMGLFPK(E)
(K)EVTSVAGKNYEETR(A)
(K)IIENYNK(I)
(K)IIENYNKITEEIK(N)
(K)IPEGQIEAVK(A)
(K)IRETVDK(V)
(K)NKIPEGQIEAVK(A)
(K)QILDVLNAGLGLIK(A)
(K)TYmELVTK(G)
(K)TYmELVTK(G)
(K)TYMELVTKGASDNSEATVK(I)
(K)VINDIK(E)
(K)STIINSLLGGEIQK(T)
(K)GILHILPEDFAK(Q)
(K)VGFHTAEILDALGIK(D)
(K)VVNSEGQDVYVPHLDR(L)
(R)FFNSVLALQPQSLFSHDVNPVSR(R)
(R)GQLPEIFAIASK(L)
(R)SLGVNPLLTICAISER(A)
(R)YGLGEMFGVSNIK(A)
(K)LIGPIFWK(S)
(R)VILSAGTLGTNR(I)
(K)LDTSHEEFLDQILNR(S)
(K)LIVEVER(A)
(K)SLPYLYGPSLVLK(G)
(R)DIVYNTLLHSTR(E)
(R)LINVDTLNHLLPVEFR(S)
(K)FLQEVVLK(G)

(K)ASEPVGFLHIPHK(E)
(K)DGLETIVGNLGYVHPAILDSFELK(K)
(K)GLEATTTLPVIR(R)
(K)HPSADKLQIVQVFDGSSK(S)
(K)IFAFAVSLDK(K)
(K)LERIPELK(E)
(K)SQIVTGATNVK(I)
(K)TFFAIALK(Y)
(K)VLENQNAHSYYASSISGVSVFPSK(R)
(K)YHEVFNYSFQSYK(E)
(R)IHTDIHFINAK(L)
(R)IKEISDQLVILAGK(N)
(R)NQGKPVAIAGVMGGK(E)
(R)NSLLPSLLK(N)
(R)SGKDGLETIVGNLGYVHPAILDSFELK(K)
(R)VINNVVDVSNYVMLEMGQPTHFFDK(K)
(R)VIYGSFEFER(I)
(-)MVHPWHDISPGDQNPEIVNGVIEIK(R)
(K)IVLDSLELYKK(T)
(K)TVVIEEFQNAILAR(K)
(R)AKYEVDKEYGILK(L)
(R)LGGEYDLGSGDPNRKDGK(V)
(K)SFLVTGASSGIGR(A)
(R)IDGMIHSAGISMR(G)
(K)GGGVSGIIPDFLSIK(E)
(R)SGNNPVYVEAAR(N)
(K)IFLITSGNIETNR(I)
(K)AGIEYSYYLIDDAVAQLmK(N)
(K)AGIEYSYYLIDDAVAQLmK(N)
(K)ALDTFQFMEAIQK(R)
(K)ALDTFQFMEAIQKR(L)
(K)DTELYIPEAGKVELVYTGK(D)
(K)FEYEAAHGTVTR(H)
(K)IDLWFATK(D)
(K)KAGIEYSYYLIDDAVAQLMK(N)
(K)KPITVGR(H)
(K)NIPPAVR(S)
(K)TPLVELDGDEMTR(I)
(K)VELVYTGK(D)
(K)YQQGETTSTNSVASIFAWTGALIK(R)
(R)ALIHDFEGAGVIMGQHNLDK(S)
(R)HAYGDLYK(D)
(R)HAYGDLYKDTELYIPEAGK(V)
(R)IIWK(E)
(R)KPIIVK(N)
(R)KYQQGETTSTNSVASIFAWTGALIK(R)
(R)SILDGTVFR(K)
(R)VSLPISVGISSILPIAR(Q)

(K)ISGIGSNILTIEGVTSLKPPAK(E)
(K)TPLHGTVVPQGNK(N)
(R)HAASEPHVQR(L)
(R)HLGMEITEEEPGTYIFK(H)
(R)LGIEVRPHENGILVPSDQK(M)
(R)VFLPKPGGDK(I)
(K)ALVQTAAASALGK(M)
(K)AVEAIDHYAVNMTK(G)
(K)KLPIVPGFEGSGNVVSSGGGFYASYLK(G)
(K)LEGFWLSTWVPQQTPYK(I)
(K)LPIVPGFEGSGNVVSSGGGFYASYLK(G)
(R)AIVYGALSEK(E)
(K)IEHLIIK(E)
(K)YYIQSEQIQK(I)
(K)FFQAYSSFSENLAESLK(L)
(K)THPGQISFPGGAYSPK(D)
(R)LIIPQENFIGIPIPPIGQEK(S)
(K)LLETLDLILK(H)
(K)ADNFYFLISHLESIK(A)
(R)IFPFLQK(M)
(K)ILNLFMTNPGVAFTRDK(L)
(K)YSMPIIMVTAK(S)
(K)ILVLIPADK(F)
(K)ENLIAFLPPWSGIK(G)
(K)YWSADGGKYPQLGIAR(F)
(R)FPWNMAK(W)
(R)IELIFAVK(I)
(R)LSFLESIFGK(H)
(R)SESWVPHLIGELIR(F)
(R)TVYLYLQAEQNPEFGIK(S)
(R)SHLMDLGGASVTGALGQAR(I)
(R)YVMVNVFNSK(G)
(R)IAHGGLAASLLAPVLGMK(L)
(K)LSLWDLTR(T)
(K)AMDLIVYYGGR(K)
(K)IESLLEIPGIGPSTIQAFQENFSDKR(I)
(K)KIESLLEIPGIGPSTIQAFQENFSDK(R)
(K)LPVLSLINTYNDNELLEWVNK(T)
(K)LTFPVPPNTAFVSGSK(I)
(K)SVNIILK(G)
(K)TTHLLAGPGAGSK(L)
(R)DALGYTSHSPR(W)
(R)FLLPSLGLSELGHK(V)
(R)GEVFMTFK(D)
(K)IGGVGPGDKLEDLLK(L)
(K)IKPVIDMPSFR(D)
(K)SGPYFGYSQNGNTFVK(Y)
(K)SSGNEFYIYSNK(E)

(K)SSPDVQAYLQGIGNSIHQK(I)
(R)FNSDKQELYSLLATLEK(T)
(K)ALQSLLTTLR(A)
(K)AFDEDGQKHYFGTHFFNPPYK(M)
(K)AKPGKPFYELK(E)
(K)AVSESDWVFELVAESYEVKEPINK(R)
(K)AVSESDWVFELVAESYEVKEPINKR(I)
K)EMADLAMGTGFNWAPASAFVDFLGGPK(D)
(K)FEIDFIR(Q)
(K)GGIALMDAImSGYTGR(A)
(K)GGIALMDAImSGYTGR(A)
(K)LVYNIGADLYEPVPK(F)
(K)MPDYFQK(L)
(K)TVTVLGANGTMGAGSAAIVASFGK(A)
(R)AMAPLDTADFVGLDVHK(A)
(R)AVVYTNDTPAFAGNR(I)
(R)IGFQLINEVAQIAEK(Y)
(R)RPGTIVSTVSSGLSIER(L)
(R)YVSYSLSIVGEVVDTK(E)
(K)EHEAIEEGVIDFK(G)
(K)FILPWTR(Q)
(K)GDKFILPWTR(Q)
(K)GVQFPFPK(L)
(R)EAVQDGLEAVGITSDEIKK(L)
(R)FMGALAEISR(I)
(R)GGEYNMAVGGR(L)
(R)MMLDLYK(Q)
(R)VAPITFEAK(V)
(R)YKLPEQR(F)
(K)DISAANLIDTMQVLLSR(I)
(K)EWTGFAFGLGLDR(L)
(R)YGIHDIR(Y)
(K)IISLFEPR(S)
(K)MIPQFTGQK(I)
(K)VIVVAGTHGK(T)
(K)ISTSLQLNENSPK(I)
(K)TNINSIDYRPAIR(I)
(K)KYLSSFPGVNVDK(I)
(K)VSGLEVTLSETDVASGKK(Y)
(R)FLFLHQGR(M)
(R)IVILDEPVSGLDPKQIVEIR(N)
(R)KQEVTLEQIFMNQV(-)
(R)LNFELGEGEIVGLLGLNGAGK(T)
(R)VGIAQALLGNPR(I)
(K)ANEEVGDKQPAPMVAVIGYSQK(E)
(K)AVKPFVTATNVTGIDFGPSVVSQK(L)
(K)FVYLR(T)
(K)GSDLKIPVYSIFDGR(N)

(K)IIYGGYDFESWVK(N)
(K)IPVYSIFDGR(N)
(K)LFLQFGGQGSPFLK(E)
(K)LTQANMGTSENK(I)
(K)QPAPMVAVIGYSQK(E)
(R)EMLIK(T)
(R)IGFNFK(G)
(R)TTAPFHSPHMEDTNK(T)
(R)VKQTNDSLGLSGSK(A)
(K)VFTELFEK(I)
(K)DKLSIEFAELIYNGHWFSSR(M)
(K)GIPVPVTAEKPYSMDR(N)
(K)KMNPLEIMETLNTIAGK(H)
(K)MNPLEImETLNTIAGK(H)
(K)MNPLEImETLNTIAGK(H)
(K)SKGIPVPVTAEKPYSMDR(N)
(K)SQVSLYNPEMATFEKEELYNQK(D)
(K)TIIAPWR(I)
(K)VYIQDLR(L)
(R)AFITETQR(Y)
(R)FELGVK(S)
(R)GVYETPGGTILFLAHR(D)
(R)IWSFGGR(S)
(R)KSQVSLYNPEMATFEKEELYNQK(D)
(R)NLMHISYEGGILEDPYKEPDEK(M)
(R)YLLGTSLARPLIAK(A)
(K)HLAIYPGSFDPLTK(G)
(R)AVTDFDYEYAISLMNKK(L)
(R)LGFIR(E)
(K)EAAAGTIR(A)
(K)EANAVHGSDSDDNAALEISFFFK(G)
(K)HVGNILSR(I)
(R)ALYAESK(E)
(R)DNAVLHWR(E)
(R)TFImIKPDGVK(N)
(R)TFImIKPDGVK(N)
(K)APITTPQGGGYK(S)
(K)DLVGVDIFLDWVGKDPNELGNK(L)
(K)EFSDAVIGNLGHLPGK(F)
(K)HPSLDIVIIR(E)
(K)IEPIEIEIGEK(V)
(K)INNAWLLTIEEGIHTGDIFKPGVSR(I)
(K)KIIIPEYK(R)
(K)NLANPSGLLNAAVMMLVHIGQPDIAAK(I)
(K)TENLYYFNGK(R)
(K)VYPDGQPETFLIDHWR(C)
(K)QLPHIGGTIGWIDDVPSELGNISVSASGK(R)
(K)SLSWEGNPVPQAR(K)

(K)SNAYLLANENPETIKK(A)
(K)IFDVHR(C)
(K)LIGDFIIVK(S)
(K)NVNNPILTSIVESVR(V)
(K)RAGATSILAFR(D)
(K)SAGLELPILFPLLGK(E)
(K)SPSTFDVFK(K)
(K)TLIINDIIQGK(V)
(K)VLENLLPFLMDR(K)
(K)VVNEFYEILK(N)
(R)AGATSILAFR(D)
(R)FAPSPSGFLHVGGAR(T)
(R)GVGHLSNTPR(Q)
(R)TALFNYLYAK(S)
(R)VYLDNLTQAPDYVAEFFVTDLK(I)
(K)EImALFNKEDVQTLAMNVITYDGK(L)
(K)KLTEFMIIELEELQNK(W)
(K)LTEFMIIELEELQNKWDKV(-)
(K)VAGLAMGYHPHGDK(S)
(K)VISLLDDILERK(D)
(K)VPIVLLNNIQGIATGFR(C)
(K)YFEWLSTIPVYR(M)
(R)AIPHEIDGLKPVQR(R)
(R)ILWAMWNSDAR(N)
(K)AIANEELK(N)
(K)AIANEELKNIVAIVGLPLDQK(V)
(K)EDHGTSVEFELNPK(Y)
(K)GLDEIFSHSDLVYYK(M)
(K)LIVQLSSDR(K)
(K)LSNVEHVR(M)
(K)MDYVAPGSASQLHLETYLVIGQNK(N)
(K)NIVAIVGLPLDQK(V)
(K)NLTWVNSIFAPQGGSAIEYLENR(I)
(K)SQDKSYLINDLNEDIR(N)
(K)SQIVALEK(K)
(K)TGLLEEIK(L)
(R)GIPATNAEGVYLHLMYGENFDDHVK(Q)
(R)VFLYFDEHGK(F)
(R)VLFVAEGDSAIAGLRPAR(N)
(K)FIPTGIATFPPGSYGSIISLLKPMDR(I)
(K)VLSDLVNFPK(Y)
(R)IFFAGEHTAELNGTVEGALASAIR(A)
(R)KFGTWDISPK(S)
(R)TVTDVSGNFLDLGAEWIQAEHR(T)
(K)MNVILSLSEK(S)
(R)LNLTYQSGK(V)
(K)DLLQTLKDDYFR(I)
(K)FIIADAPGHVQYTR(N)

(R)FPVQYVIRPLSDEHHDYR(G)
(R)GNNGQINLALLTDGLK(A)
(R)NMVTGASNSNLAIILIDAR(K)
(R)TAKPVSFDEYR(I)
(K)DSITLVHLALK(A)
(K)ENIALPSLYFSHK(R)
(K)FITIDANDRVEDK(I)
(K)LQRPELWNIYNGR(I)
(R)ETASQFERPALLFSGGK(D)
(R)NGIQTVTLLDTIAEFK(F)
(R)VFPISNWTELDVWEYIR(K)
(K)YHPLLDWSLER(I)
(K)FSLPVLK(I)
(K)ADDYPEIQETFAVLPNKK(N)
(K)DNVVTALEAFFK(S)
(K)FQHEQVPQISGNASGIFLSGK(K)
(K)KFQHEQVPQISGNASGIFLSGK(K)
(R)QGQLIPGNIYTITLSK(V)
(R)YGLIFGTNYTGNK(A)
(K)LIAQRSSKMK(E)
(R)GFAVVAQEVSK(L)
(K)IGLEIHLDK(T)
(K)KLLSELIHVLGNGDDLSLK(N)
(K)LIAFLPR(H)
(K)LLSELIHVLGNGDDLSLK(N)
(R)EEIKPLDFVLPSQIIDR(T)
(R)NSTYFGNGVVAHAPFAEPFSPNLSK(R)
(R)YSIITAPEK(R)
(R)YSIITAPEKR(N)
(K)LSAEITK(I)
(K)MESQAASLEEVNAVITSLSKSSEK(N)
(K)IARPDVIHWAPGLPK(T)
(K)RFFETYFSQFK(G)
(K)YGVNIFYTAPTAIR(A)
(R)FFETYFSQFK(G)
(R)IGAIHSVVFGGFSPEALQSR(I)
(R)LIWFK(K)
(R)LLGSVGEPINPEAWEWYFK(N)
(K)AHVAYLPNKK(I)
(K)AYEFLTSGYR(A)
(R)IVDVFAR(R)
(K)LIYNGIDHSVFYPATSTEK(K)
(K)SIVTIHDIIPFR(M)
(K)IQFIDISKPNYIYYK(H)
(K)HKPDLIVPEIEAIK(T)
(K)ILLLGSGELGK(E)
(R)AILGIPILEITLER(K)
(R)KGASAVILASTENK(T)

(R)MGVTLSYSTK(G)
(K)KWIPQTTPSSTADDVLAQNR(K)
(K)LWVNDWIK(L)
(K)NLSSWNANITYK(N)
(K)YSGQFHVVQTGYTFAEK(L)
(R)MLGGANWTINGLSYDGAR(V)
(R)NSPLIYNTTTNEFELNSAAFMK(Q)
(R)VWGGASPANVGDIR(S)
(R)VGAPVFQIFYDTWR(M)
(K)GTYYSAVEGWR(G)
(R)FWLSNEGIEHEIVEYSEK(E)
(R)GIPLGVMEALTGNR(F)
(R)GLIDLIK(G)
(R)LGFQK(R)
(R)LVLLELER(V)
(R)SVTEEVVGHFER(A)
(K)IIDSGFVHVYSTDR(E)
(K)QPDLFIGGDPPRPDVIVSAFR(Y)
(K)VSPLNPNAR(G)
(K)TLNDIFLSMTGR(G)
(R)NNPLGIVYAEQNHQFASNSNR(F)
(K)FPFLVTVK(R)
(K)GWSFSELGFGK(N)
(R)TEVGVNYVFIR(T)
(R)NPASLEIVTLLLK(E)
(R)SYSGTVSTGK(I)
(R)ILIVDFNPVFR(Q)
(R)YSPITTPENIEK(E)
(R)YSPITTPENIEKEIQNLLK(R)
(K)AENFAGLILPGGHAPGMK(E)
(K)SQEMAAWNLTR(L)
(R)LWLGSYYR(T)
(K)EYKPLIPEQELLGIGINEIK(E)
(R)GLTAHQQALQTTGHNISNADNK(H)
(K)LPDSFLFHISESLVK(G)
(R)MDGVFWTYYDNLRPR(Y)
(R)NSNIQIQLSLPEDFYFHNPK(N)
(K)NFSEILSNMGSFR(E)
(K)TSAFFGHEVNVNLR(W)
(R)VVFTPGTGVTVNK(D)
(R)YFNQNNWVK(-)
(K)KLELPYR(V)
(K)MLSHAENILK(K)
(K)THFEIGEALHWIDFEK(G)
(R)ALMNFMLDVHTK(E)
(R)YMEISSVSNFR(D)
(K)SGLPVVIHSR(D)
(K)ISNELTQDIISILK(K)

(K)TEVIGFVGK(T)
(R)LTVMVIPHSEQK(T)
(K)AHGVIQPIVVK(Q)
(K)ISEIRPNPGQPR(K)
(K)MTIVYPNLEAMEK(I)
(R)GLGNLIPVNESK(T)
(R)KKIEQLAYQIAEK(G)
(R)LLQLPDVVQDLIK(N)
(K)FTVFLK(E)
(K)LEFSEDLVPLEFLGMR(H)
(R)FPTEADEISSFFDWDLVHK(F)
(K)EIFNGYEFVHNEGGIGTK(D)
(K)GIIWLDLK(S)
(R)AMTSNTVSITGIDTHQAGINVITSK(T)
(R)NPLLGIVLNGVIR(S)
(R)SRNPLLGIVLNGVIR(S)
(K)IVYSALYEYNSSGK(L)
(K)LLTRLNVQGNQER(K)
(K)NQLNYSSNNLLQSFTFHVK(Q)
(K)YIFGNNSNGPVK(E)
(R)NLTPLLFK(R)
(R)SESYIGVLIDDLVHK(G)
(K)IIGIDSIALSRLFFK(L)
(R)FIFIVNNLDK(L)
(R)YISKPETIPPLLIFHGPDGTGK(E)
(K)VGIQVPVIPGIMPITSFSQIDR(F)
(R)SSGILNLMALR(G)
(K)HIPLGAIAFQR(E)
(K)ILIPGELTTANLLLK(L)
(R)EVVDSHIDLYVNQFTR(S)
(K)TIFPTELILK(D)
(R)LLSEDFFNSDELEYQEIRK(F)
(K)AGVTHFILFLIPELK(S)
(K)KLIQPIFIVEGLK(E)
(K)LIQPIFIVEGLK(E)
(R)AGASYLITYAAR(R)
(R)DKEEGADFLMVKPGMTSIDLIGPIR(E)
(K)NIAALIIEPLPANYGLLIQR(K)
(K)VSPGGVHSPVR(S)
(K)YGTLVVFDEVISGFR(V)
(R)FVNSGTEAVMSALR(V)
(R)KYGTLVVFDEVISGFR(V)
(R)RIDLIPEGHKESFAK(V)
(R)VGFQGMSGLLGIWPDLVTYGK(I)
(R)FIYKDFMLDFQGFQIK(R)
(K)TFVEIVR(D)
(K)LGYTSVNFDLIYGLPK(Q)
(K)NLEILLDSILK(K)

(R)SLILQLSTSGK(V)
(K)IAIIAGPHR(K)
(K)IWKPIEAELK(N)
(R)LGSLGHQSWILDLGNHK(L)
(R)GLPGSASYSSSK(A)
(R)YGISVSVIHPGFIDTPINNR(M)
(K)GLERPLPGVSPVAMQQGR(Y)
(K)LLISFEK(A)
(K)NVTVVLGEATK(I)
(K)VVVIGAGFGGLQAVK(K)
(R)YVASLIQNDLK(N)
(K)VYAVLVTVQKPTFNSEEGSQMK(F)
(R)VWNAVTVNEELIHWLADK(V)
(K)ANLANSFDPIFILK(Y)
(K)GNAISPAGAYVEK(D)
(K)ASYKPDIPFTYYK(D)
(K)FNWSNGNPAYK(G)
(K)GKDLEVQILPPNIR(V)
(K)TGEGPIMVGTNKK(N)
(K)TYYDNGSIQMEGGYKPK(L)
(K)VSVLIR(T)
(K)VYYINGNENK(R)
(K)SEFLSGVFTK(G)
(K)INVDYPTLEEEVSILDQHGK(I)
(K)TSLVEILR(H)
(R)IQFTPDLLPADLVGTVVFNPK(T)
(R)KGPVFTGVLLADEINR(A)
(R)SLASALDLNFK(R)
(R)SLASALDLNFKR(I)
(K)ISAHIVK(K)
(K)ITLHLDGDSGEYLDQWK(L)
(K)QTPLWSEAPSSVTK(E)
(K)YINQIAFSSAEEFVK(K)
(R)LNEMNMLGGQKPLDLLK(N)
(R)SYLQSDPLVK(K)
(R)YVMEPYQVQLIFQK(E)
(K)TEIIDPPHPNVVILNLNELK(Y)
(R)SIFELK(H)
(R)LFPNGDFIFR(K)
(K)GFLDMMWK(T)
(K)SSLLGELATQFLK(S)
(K)EPAWIFR(T)
(K)INNNEMVIVGK(N)
(K)IVVGKPGLDGHSNGAEmIAVSAK(H)
(K)SGKPSKEPAWIFR(T)
(R)ALQLPAWNEALSLPRPWDQQWSLR(L)
(R)GTYIFPPAQSIR(I)
(R)ILIEALGVTMSR(D)

(R)SVFGEYRPATGVEGQK(L)
(K)GYPSILFLDR(N)
(-)MEFLLQLENILK(K)
(-)MEFLLQLENILKK(R)
(K)SYTADLFR(G)
(K)AGANLVIADLNEESSKATADEISK(Q)
(K)AVALEMASR(K)
(K)NPNGGSIINLSSIAGVNGNIGQTNYSASK(A)
(K)FIDNLK(S)
(K)FIDNLKS(-)
(K)IQTVGDVTKFIDNLK(S)
(K)KSSITSEWLTSTGYTIDDIK(Y)
(K)LLSASTPEQYIELSIK(S)
(R)TSIPAVNHIR(R)
(K)EWNYQILNEIFQK(V)
(K)FLEAIGLEKPV(-)
(K)GHSTQIAIR(W)
(K)AGIPVFAWK(G)
(K)EMPGLMALR(Q)
(K)KGELKVPAFNVNDSVTK(S)
(K)QADYLGVPINGPFKPDHYR(Y)
(K)QADYLGVPINGPFKPDHYRY(-)
(K)SIIVLAEGR(L)
(K)VAALHLEQLGVR(L)
(K)VPAFNVNDSVTK(S)
(K)YELGVYTLPK(H)
(R)GISEETTTGVK(S)
(K)AAIFAIEQVFEDLQVR(I)
(K)FLIATVK(G)
(K)GRYPAILESETTGK(Q)
(K)IAPVYDHPVVHVVDASR(V)
(K)LLTVFHTLR(Q)
(K)NTGITLTEHFAMWPASSVSGLYFAHPK(S)
(K)SVAYLLPFMEEEKNQIENSTARPK(F)
(K)VSGGLSNVSFSFR(G)
(K)YGRPLTVIEGPLMDGMK(I)
(R)AVTFDDLVATFYEQAR(A)
(R)ENRDPIDWSTTVIDKPSFIGIK(V)
(R)FAEAFAEYMHYK(V)
(R)GIRPAAGYPASPDHTEK(R)
(R)GIRPAAGYPASPDHTEKR(A)
(R)HFLNLIAGEPDIAK(V)
(R)IGLEIPLLIGGATTSSAHTAVK(I)
(R)VVNVVNQLLHPDLHEAYSQK(V)
(K)LKDIGLFLK(D)
(R)ILGFPYGYQGLVK(R)
(R)IIETGNPILVQNISTVEDWFRPER(Y)
(K)VYGISLDALDSHGQFIQK(Y)

(K)YSLNFPLLSDPDHK(L)
(R)FVPVQFLNVLGK(N)
(K)VDYISYDHLHTQDIR(K)
(R)DLPHPVDR(L)
(R)LFIVQYK(T)
(K)IAEEAFAAGHSILITDINEK(E)
(R)SGHIVNIASLAGVAPISGISLYSTSK(F)
(K)SIAVVFGLSRPTEILVEK(I)
(K)VGSDLAFTGGLLHNIGK(V)
(K)SEDVLKDLFSQLPK(R)
(K)VFAVVGAGHLQGILNHVQEER(D)
(R)AWWNIGIFNR(L)
(K)DELSVEIISNSHVNHLLKPHMTIVK(T)
(K)GALIVYAIPSPINK(I)
(K)IFELTR(L)
(K)LITMSIDLDEALDK(A)
(K)GLSNSWVEYSPQK(S)
(K)IFDFLYK(Y)
(R)IVQLYETLEK(I)
(K)ILPSLGSLFFDSPK(L)
(R)EQIANGGPVTVTHPDVIR(Y)
(K)FGTFYLHALPHPQLVIGK(R)
(R)SIAGILENAGVK(E)
(K)MMMGVQVIDPR(S)
(R)TANLPMPEVGIYHSPEVNAFATGPSK(S)
(K)GKLPSETVSEEYDLEYGKDVIEVHK(D)
(K)ILLMDDLIATGGTMIAAVK(L)
(K)LPSETVSEEYDLEYGKDVIEVHK(D)
(K)GENIGYLIPTNVIR(H)
(R)SGNTSGGSQLLYR(F)
(K)IVLLSQVLPDESNNGFHDLSFK(I)
(R)MIPSFIIQK(F)
(R)FIFPGEFLADHWR(D)
(K)VPFVNHSIAAVK(V)
(K)YSVYIPTANLR(D)
(R)FGYPLQELNELVVK(G)
(K)FLFTIGTLEPR(K)
(K)FTDFLLIPK(V)
(R)IVIVSPDFK(T)
(K)ILNLPTLAMISEFNR(L)
(K)VQSIAIPGTELIVQDLAILK(T)
(K)VILWSPLLYEGLETR(V)
(R)SNLVPLFR(K)
(R)TFLPVLK(R)
(K)GILLPFLTLLNQDAGASTAK(S)
(K)AIPWTR(Y)
(K)KVLQEISELLHEK(K)
(K)VLQEISELLHEKK(N)

(K)YAHWEDNSKPSGLK(E)
(K)FKAIVSQEILNGDVILTGPK(S)
(K)GEISVQR(G)
(K)KINDADYLSVAK(G)
(K)LNVPYLHNHSEIENSLVGAQNK(M)
(K)MNLHTPTTTAGVR(G)
(K)MTPEQFQIIYELAK(K)
(K)TIYITPSDLQK(L)
(R)IELENHQNHSK(K)
(R)SSLHLFEPK(H)
(K)IIPIDLILTGSYSIQK(I)
(K)LAELLTEHSLEYSGLLDSDQAR(K)
(R)YIVYLLNIQSETNK(Q)
(K)ELISIAHPNHR(E)
(R)GASLSEGGKPIIALPSTTSK(G)
(R)IVSLLKPGANVVTTR(A)
(K)GNESSLIVLYEPHPFAFK(E)
(K)LPPKFEIGFmIFPELLGK(W)
(K)AQLSEGKPVVAGVLVYENFFNLK(G)
(K)ETLFLYSVTSK(T)
(K)GIVEIYVVSAK(L)
(K)IFSPAFIYNQINGGR(D)
(K)ISITWDPVKEASEYYVFR(K)
(K)KGIVEIYVVSAK(L)
(K)TPPAKPLGLIASR(G)
(K)VLINWEVVPGAVGYEIHR(K)
(R)TRPSQAAIEAASK(Y)
(R)YAYYSVTAVNTEGK(N)
(K)LNGGLGTSMGLSGPK(S)
(K)MTSEGLSSAFIQDFLKK(T)
(R)TFSTNNLWIDLVALR(Q)
(K)GVVIGFIPMASTPDQFLENLK(S)
(K)IGFHSVMER(I)
(K)YIAFPAWK(W)
(-)MNIKPLK(E)
(K)DAHEALELAIR(E)
(K)ETTFLEAVAAAIQHEK(D)
(K)FITELYEQAEGSELPNLKQLAAIHK(F)
(R)LAEEVDDHIK(F)
(K)GIEFFIHVK(S)
(K)HSIPVLK(E)
(K)QWLTSNLPGTFPFAR(S)
(K)TPTVLLLPMGDLK(M)
(K)VVDPAAGSYYLESLTDSITKK(A)
(R)ILWAEILK(E)
(R)SPEIFSWLPK(D)
(K)AAADILELLIK(D)
(K)AGVAIDSILDMK(I)

(K)AVEEKPLDILDIDNTAVR(E)
(K)TLVPQVIAELK(K)
(R)GPYSTMYVQQPWTIR(Q)
(R)NTQIFLQEETNIHR(V)
(R)NTYIYPPAPSMK(I)
(R)VVDPWGGSYYVESLTHSLAHR(A)
(K)GTTVFGGAGNHFNFPLDTR(F)
(R)NPNIYKPDAIMTQVATGSEQR(L)
(K)AFYGLGTLGSTGAEFVMR(S)
(K)FSQFSAEHYEVGHEALSELQEGK(I)
(K)SIASENHGNIYAK(F)
(K)GNSVGVYIDELSALFR(Y)
(K)LILVAGAGSGIGK(S)
(K)SVLENLNLLNQAAIGVSR(T)
(R)VITVYPGAVYTDIWK(G)
(K)FLVHVYKDDFISVPR(N)
(K)VPHNASNLTDK(E)
(R)DLIVLHPNVSGLNEMLAK(F)
(R)YIVDGSGVFGFAFK(D)
(R)YIVDGSGVFGFAFKDEK(F)
(K)TDIAAASRPLTETELK(E)
(K)AVHKDELAGLSGLSTPHR(V)
(K)RFGETVVVHFGEDER(V)
(R)DFYLVSPLHDDGIDSIHLIR(Q)
(R)GVLNLGVDLHHKPVGN(-)
(K)VVQQFPVPLQNLLK(A)
(K)FEEAIFLAK(S)
(K)IPILIMGEIQNPEK(R)
(K)NFQLNPILSWK(S)
(R)IGVNSIEEALLIR(N)
(R)ILSSFLPAPK(L)
(R)IVHIQYHPADSYIGYGSTFQTSYPTK(V)
(R)SLSNNLDNFR(S)
(R)THTINYEVMTR(I)
(K)LFSSHLEDTFQNILK(K)
(K)TNLIPIFLK(Y)
(R)APYPTVIMGNHVEEGDVPALSVIHR(V)
(R)NLFLFPEGTYNHDGYLNQIK(K)
(K)IAELIHNSSESETGK(I)
(K)IFFSLESLNGIK(I)
(K)IFISNTLK(I)
(K)ILNQSPIQEMFSLGLEK(I)
(K)LNPLASMFQAAGFEK(Q)
(K)VPTIELIPIVR(E)
(K)IEAGEIPFTTWLDLHLK(K)
(R)AIEHLTDPPSVLLEALDLAAK(D)
(R)LPILPLK(G)
(R)SSAIENVLMMQGLEMDKR(N)

(K)AGSALNLISEEQNK(K)
(K)ANHTFNSQQIAWFK(A)
(K)ILYSHLWEGEPK(T)
(K)KQGMLALTFADK(S)
(K)NQLSGAYEPVPQTAR(A)
(K)NSIITSFNR(N)
(K)QGMLALTFADK(S)
(K)RNDGLAGTHAFVASPEIVTAFAIAGK(L)
(K)VAGILTVK(G)
(K)VTVAVDPK(S)
(R)GHLDNISNNLLIGAVNSFNDK(T)
(R)GHLDNISNNLLIGAVNSFNDKTNEVK(N)
(R)IHETNLKK(Q)
(R)LQLLSPFIPWEK(T)
(R)NDGLAGTHAFVASPEIVTAFAIAGK(L)
(R)NVVGRPLTLTEK(I)
(R)NYIAGNGISK(D)
(K)AGIKPNVGASGEIIR(G)
(K)NSAQPEWIETIWGIGYR(F)
(K)SLDSAHYSTTLQTSGEEGLK(F)
(R)IMGLELGADDYITKPFSVR(E)
(R)KEFDILQLMAASPGK(V)
(R)KSLDSAHYSTTLQTSGEEGLK(F)
(K)FRTEIPLLSK(T)
(K)LAARRNR(K)
(K)EMNIPLDFKPEQK(E)
(K)DVLKNPIAILLDTQGPEIR(T)
(K)HINLPGIR(V)
(K)MIQALAEAGMNVAR(L)
(R)GTTALNVAGFHPHYPLIYAFTNMTTVR(R)
(R)VIVATHLLESMIHNPSPTR(A)
(R)SVFYYNIHTEILNR(I)
(K)NYANAGIHIISNSK(N)
(K)VAVLGATGSVGQR(F)
(K)YVVLSHNTIR(G)
(R)GAAGAALLNAELLYK(K)
(R)LRPDPILDWK(Y)
(R)MDPTVPILSAEVNSSHLEVLTSQK(T)
(K)GIILWEWK(L)
(K)MTYVVFTQK(G)
(R)AADIMDSGNLNIHSGNPVFLAR(I)
(R)IAFLGSQFNLSHFR(L)
(R)LAELILEMK(I)
(K)GILVIGFLPEQISK(L)
(K)FSVDGIVGLQQTITSIK(K)
(K)TTTSLNLSMGLAR(R)
(R)IFETSISDGVAVGESHLAK(K)
(K)ILPSENQPR(Q)

(R)ENLSPYEEIEAMSHLK(T)
(R)SYMTELLGISSLSK(E)
(K)KPFEVGDELTNLLK(M)
(R)VWENVYANIYNK(V)
(K)FYPVYTGLYDDNEAGVMHTAVTVTR(L)
(K)EENHFFLVIDPVMVATSGAK(L)
(R)KKEENHFFLVIDPVMVATSGAK(L)
(K)DLQKFDFTEEIIQHFK(I)
(K)LSIEQAMYHFLSGYTAKVAGTER(G)
(K)TVFEYTIATK(D)
(K)FGADVISVLEDSDFGHINSIQR(I)
(K)KEPDKTHTQGGISVTR(I)
(K)SAGSHWSEALSMKPGDPAVSLDYSR(F)
(K)ENIMSIIDIMVTDTNKR(L)
(R)FEEIRDNILNHFIK(K)
(K)KVNGSLAIIDKR(R)
(R)VPVDNLHFAPVLAEYVNTK(K)
(R)NLLIYSGGPVDPTFISVLHEDNK(I)
(K)IPTLLISGR(R)
(R)NGLLSEVGASLEELRK(K)
(K)HLYGVQFHPESIMTGAGLDLIR(N)
(K)KAWEIGLMNIHIEPR(F)
(K)LIQDYTPSHIAMTFDPGGPLER(G)
(K)GLNVTEHGAGSSWIDLIHSLK(D)
(K)LTSIHEEGGVPHADDMIR(H)
(K)ELRPLREEGTLILGSGNIVHNLHK(A)
(R)KIEFAGLNEHFRK(V)
(K)ESLSYVLHDPSDQIELTVTPIRK(T)
(K)DIQELETFVSHVENPKPIELPK(E)
(K)AGEIGLFWKPFGLSDIGLHK(E)
(R)RPTFTISNWNFGAPALVHQSPR(T)
(K)STFISLIDKEKENALSGKPAR(I)
(R)LLHHPYQSFSAIEDLLR(I)
(R)IAHFILPIAGK(G)
(R)RVGGVTYQVPVEVRPER(R)
(K)IDLDSNHFFSPHQTFHR(V)
(R)ILEYHYALYSFPKPVVTILK(G)
(K)LVFR(T)
(K)EIGQLQNLQVLFLSYNQFK(T)
(K)SLFLSNNQLTTFPK(E)
(K)GVSFMENNPGWHGTPPKPEEEKK(A)
(K)NLALSHHPTLNSDLLDFIR(H)
(K)LITLDR(T)
(R)IFSLHNHFTAEGLLEEFKDQR(D)
(K)LSDPTAFLDVLFSVSK(N)
(K)SIQSVLQDPNTKEELK(I)
(R)SAILGVMATVPLPDHR(I)
(K)DVSLSGNQISELPEFLSEMTALR(E)

(K)SWNSLIQLLPHTENK(I)
(K)LVEGSAHLVK(Q)
(R)LALPGTNVTLSVFR(T)
(K)IFDKSIATIGK(N)
(K)RNSSSGQSQSNEKPQTSVSENVPEK(S)
(K)ISSPTLLFYYYK(N)
(K)TIYIFMQR(L)
(K)LAEFKKLGTEVLGVSVDSAFTHLAWK(N)
(K)IKELVSSLASLGGVVHSPYLR(R)
(K)DNSISILTDHGALKEDIDLEVEK(K)
(K)DNSISILTDHGALKEDIDLEVEKK(N)
(K)LDLITVDGHFNGWQK(A)
(K)LYHLKPNQILMIGDR(L)
(R)LKDGEVLIVFAASHLIR(N)
(K)GFVYPHHSSK(F)
(K)NVTAGANPMSLK(K)
(K)EFWPFYLGEHSHPVNR(M)
(K)EGIQVTLLNMATIKPIDKETILK(Y)
(R)GTDFGIPAGAIHLQIIGLK(N)
(R)VGNTHPLHYDK(L)
(K)DGVTSIFDLLDLR(K)
(K)DGVTSIFDLLDLRK(R)
(K)LFGHEPWHIR(V)
(K)ILEILK(E)
(-)MENPNVEKEEEDKQFAVIK(G)
(K)KIFSEFGTVEEVHIKK(D)
(R)MLGYDSIEEVIGKPVLQFVHPR(S)
(K)ILVLILR(L)
(K)DKDWFLIDPIKR(S)
(K)VDFSSRPFK(L)
(R)IGGHVVAFVANNSAVSSGQIDIGAAR(K)
(R)GYDLQTNLLVHYGLIHWILGK(D)
(R)KLTLITRPIGELIK(D)
(R)AWLTKPFSPEELVQTIHK(L)
(K)IVTVDPQVSHFFLYK(R)
(R)INMSEINRPGLSLTGFYESFAHDR(I)
(R)VVVLEGLSDEEIAKDEDLKK(I)
(R)HVVITSVNRDDLEDGGAAHFAK(T)
(K)GIFYDPFLK(Y)
(K)FLSLSTNPQNDEKVEIVYEDKEK(T)
(R)LQALSFVVHFIGDLHQPLHTAER(N)
(R)RFDGGASFITTGGIAVNHEGR(G)
(K)KVQHTNFLDISLR(K)
(R)GNTLVVVSALDNLVK(G)
(K)HFDLFANLRPAIIYPELK(N)
(K)NLIDEGAMDYIPKPIKR(E)
(K)KKGEDIVSFGAGEPDFETPSHIR(D)
(R)TPIEYPPVPHAVFTHPQIAK(V)

(K)VTILDPYKDGVHIK(A)
(R)MVYETLKEELKQEVHALALETR(I)
(R)RPEATFATMDHNVSTR(T)
(R)MYGDQFHSSPYANPEGVHVSR(S)
(K)ERPDFAYILNLIPSGSR(V)
(K)LIQDEATLAAIIAHEIAHAEK(R)
(R)IDSTLDYLSTFTEKDFLEANDRK(V)
(R)MNQNSEFDIVTLIELAK(R)
(K)FDQGHLGILFHMPTVR(E)
(K)ILEENKITIHATETVMDSFLR(K)
(R)EKLEFENVHLATR(S)
(K)TLTTAGGHPVSQNQHSLTAGPR(G)
(R)SNPGGSIKDRIALSMIEDAEK(T)
(K)KLAALADIYVNDAFGAAHR(A)
(K)LAALADIYVNDAFGAAHR(A)
(R)SGPIGSFHLAFRPELSR(F)
(R)VALFQGLPGETYNIGTR(N)
(R)HVVRPGISGWAQVEQGYAAEVDGMK(V)
(K)GEVIKDYFLNYHAR(M)
(R)NKPILALTVDGWGDGK(D)
(K)HASEDMVEWFPASVK(E)
(R)KFGINSDHSMFDAIILDSSLIK(N)
(R)LKHASEDMVEWFPASVK(E)
(K)SVYQLALQDPR(V)
(R)LIANGGGIVYAPLGPTHLAVEDIAILR(A)
(K)GFAMAEGNPQWHHK(N)
(K)GFFPLDVLK(T)
(K)HSLSNLAVIIDYNK(L)
(K)SFGFAIEEVDGHNIK(E)
(K)TFGKPDSILGGHPER(G)
(K)TKPTAIIAHTIK(G)
(R)GHIGPALSLIEILR(V)
(R)KLIVEMMESEKR(G)
(K)EFLILSPSDFGFHNMLK(N)
(K)GMFAIFGDFLEK(K)
(K)IGLIPENWFGTK(L)
(K)SFLEMDLIPFK(D)
(K)TGFIQNVYFHDTK(E)
(-)MINIPYINLVEQWK(N)
(K)AIMPVHLTGR(V)
(R)IADKYSIPVIEDAAQSIGSK(Y)
(R)ILTLPIHQYLK(D)
(R)MDAIQAAILNFR(L)
(R)NTVGEFGFVTR(M)
(K)AGTWGNTGAFSLHPLK(N)
(K)AIVPVHFTGYMTDMR(K)
(K)NLNVWSDGGVIVTDDEELAK(N)
(K)VHYPIPIYR(Q)

(K)YAIGVNSGTDAIK(L)
(R)FVPTGDFTLGKPLIEFENQFSK(L)
(R)IENANYYDQHLR(S)
(R)LDTIQAVVGNWLIPNAVAISNK(R)
(R)LDTIQAVVGNWLIPNAVAISNKR(I)
(R)SISQITLPPRPK(D)
(R)LDVVVNILTNLAYHKR(E)
(R)VLGLPLVEIK(V)
(K)EHLVLLGIKPNRPEIGYGYIEAGK(S)
(K)DKPIDILEIHDDNYGIITSGR(I)
(K)VAIIMGSHSDWETMKEAEIILK(E)
(K)ILYNGLARNK(W)
(K)SMHPNGAFFYSDEQEIKPLFDR(W)
(K)IGESDRKEFEEAISEFIIHR(S)
(K)AQLVFISFHGGAEGGPALHVK(N)
(R)SFLFGK(V)
(K)IVEVFGELFQK(G)
(R)HDQMASATTGGQVGNSVYIVGATTGR(D)
(R)ESVEHMLLDKEFLK(K)
(R)HIGEEESFFHSILQSEYDTAR(N)
(K)KPYLSLYFQRPVDPSR(N)
(K)KPILGLALTLTTHYPYR(T)
(R)MIEDLKEHGTAERDPIQDGK(Q)
(R)AVVVNDQIVIRPMMYLALSYDHR(I)
(K)mMTYIPNTDEIHIYPKPPVKK(L)
(K)TLGVHRPETAAITGIEAVNLWK(Q)
(K)GVVPENGKIEVLEHPVHPR(N)
(K)LVVLFHDKEGFTHNIESLTPADVVPA(-)
(K)VISLLNADTADPVFEYFLGK(N)
(K)YGAKTDLAVK(R)
(K)LKGAELSDLK(K)
(K)LFQFALDTLPEHEPAFVMSVHK(S)
(K)ERAPIPMELAVAAVHNHLIR(S)
(K)IAQGAKPGEGGQLPGKK(N)
(K)LTSSDLSEGILKDFEANKNYFVK(V)
(R)APIPMELAVAAVHNHLIR(S)
(-)MMQEPSQIVHR(K)
(R)EFHNASSASLFTHILGGGTSSR(L)
(K)REAKPPEHEILISR(I)
(R)EAKPPEHEILISR(I)
(R)TPPVLLMFDLLDK(S)
(R)KLDPQIQNELKDLVLDLK(Q)
(-)MNSSILTGIFGGSFDPPHEGHSEILK(S)
(K)ILLSLADLLEK(R)
(K)ILLSLADLLEKR(K)
(K)GRLDVSIANEVEENLNDLIDNQGHK(K)
(K)AKELGLTIEAVVSGWHMK(G)
(K)KINPNGGSIAIGHPFGATGIR(L)

(K)KWNDELTMHTLIHEDLKR(L)
(K)YGGFIPGIRPGSHTK(E)
(K)SILDLMKEEGFIKDYESVK(V)
(K)ALSLASYKLPIHTEFVK(R)
(K)KGPFIDDHLMK(K)
(R)VEQGVLKVNDEVEIIGIRPTTK(T)
(R)VLNLAGNQFTSLPK(E)
(R)VLNLAGNQLTSLPK(E)
(K)NINHYDVEIFYHSPDLR(Q)
(K)AIDTHQIDTENKEEILESLLAR(E)
(K)ASSPEFSTTIGMIK(Y)
(K)LLINEYQMNFPK(F)
(K)SPNIIIELWQK(V)
(R)IGMITNQSAFGPDGEYHFQSIHK(R)
(R)STNMFVEMVPSR(D)
(K)IFASGIRGR(I)
(K)TQIQMVGNTLK(F)
(R)VLIPDIPGFGK(S)
(R)NIGSSLHFIPLYEHPFYSSR(F)
(R)FITPGLGQVVADFEIPKEEIER(I)
(R)AIVGLFAPESK(S)
(K)FLENQGGISLEVSFAK(K)
(K)VLENQNAHSYYASSISGVSVFPSKR(K)
(R)LLPHQIESTYIVVNSLRPR(F)
(K)LTQYIIQTTK(N)
(R)VATTVGPNSTVGQK(D)
(K)YLRVDSIAYLSVESMNR(A)
(R)KDPEALYLDFTSILFR(D)
(R)NIPLRLPEPITVYLR(G)
(R)YIAGVLSHELSHYYNK(H)
(R)AEEISNLIIQLATK(Y)
(K)KPINPSGGLIGVGHPVGASGVR(M)
(K)LFASASSSPR(L)
(K)AIYVSLYNTPDSNIVSGSPESLLEFR(K)
(K)HPSLDIVIIRENEEDLYTGIEHR(Q)
(K)LGIIVLPILNPGGVALK(Q)
(K)LDKDKPLPNWETPSYLGWNER(Q)
(K)IAVIGSGIAGLMTAHDLLK(H)
(K)HKPDLIVPEIEAIKTER(F)
(R)THLELTMVHEAMLLEASGSR(R)
(K)LQVR(S)
(K)GIAIPPELYDEIKEAVIEAENEVKS(-)
(K)MKEEINSLHEYVEYYYGR(L)
(K)ETSKPGEEFLLIDLGSISR(V)
(K)GIPSLGETGSGK(A)
(K)ISLGQLEVGISGIVK(I)
(K)TGQWNFSLVQAK(Y)
(K)VKGNTEAAYTNISPPPENHSPIYGR(N)

(R)GFGGIFHSR(S)
(R)ISHPDLIK(I)
(R)LAVDGENSEGAAVQSNDRR(L)
(R)LHSNSQEIAFFPDTFR(F)
(R)NATTSYPGIVEFATLGNSVPGK(V)
(R)NTGLIGYGEK(F)
(K)IKPLADLAVQAVQSGQVEFIPK(M)
(K)ITPAHDANDFEAGQR(L)
(K)LNLLQEIVTK(I)
(K)LSNPGFLSK(A)
(K)QVLSDVLIR(A)
(K)STLWLPGMDHAGIATQMVVER(E)
(K)VLIHGLVR(D)
(R)FFLAATLPEGK(D)
(R)FILMNLEESFVPIGITPDIEK(D)
(R)GGAVIEPYLSTQWFVK(I)
(R)QIPLLFDSFVDKEFGSGLVK(I)
(-)MQYPFQEVESFWQK(F)
(K)IPVWISDYVLYGYGTGAIMAVPAHDQR(D)
(K)LIHQGLILGEDK(R)
(K)LRDWLFAR(Q)
(K)SEGLEITFPFLKPLQSGLEGIR(I)
(K)TGVFTGTFVFNPADPSQK(I)
(R)FSNQGSNGLDYR(Q)
(R)GVEGVFR(F)
(R)LFEMFMGPLEMVKPWSTR(G)
(R)QIQMIGLSYDWSR(E)
(R)YAMQTGIHPAITTK(D)
(K)AIDAMIPIGR(G)
(K)ASTVASTVEMLR(N)
(K)ATLVVYDDLSKQAVAYR(Q)
(K)DLGVEEVGTVLEIGDGIAR(V)
(K)KATLVVYDDLSK(Q)
(K)KDLGVEEVGTVLEIGDGIAR(V)
(K)LELAQFR(D)
(K)LGEVLSKVAEEFLR(K)
(K)LTRPVESPAPGIAMR(Q)
(K)MKLELAQFR(D)
(K)QPVSSPYPVEEQVVEIFAVTR(G)
(K)TSIALDTILNQK(G)
(K)VAEEFLR(K)
(K)YLLNTIK(E)
(K)YLLNTIKEQYSEVLDSIR(K)
(K)YLLNTIKEQYSEVLDSIRK(E)
(R)DLEAFAQLGTELDPATQAQLDR(G)
(R)EAYPGDVFYLHSR(L)
(R)GFMDKIPVAK(V)
(R)IVQMLK(Q)

(R)QPVGEPmQTGIK(A)
(R)VFGLK(N)
(R)VGSAAQIK(A)
(R)VVNPLGEPIDGKGPINAK(L)
(K)ILPIASVGVK(G)
(K)IVGNLFLEAR(D)
(K)IWQMPVVLLPIGEK(E)
(K)IWQMPVVLLPIGEKENEK(S)
(K)SIVLRPVESQEAMTANFFR(M)
(K)TWGIDQFLK(E)
(R)HPFPGPGLVVR(M)
(R)VLGFLIDTGFMR(K)
(K)IAEVFEELTGSK(I)
(K)IFLWGPVTDESSKDLVGK(L)
(K)ITFYINSPGGVVTSGMTVFDTIK(M)
(K)LEEDTDRDYYMDAEEAIK(Y)
(K)LLYLEMK(D)
(K)VMIHQPSIGGQIVAPATDLK(I)
(K)VMIHQPSIGGQIVAPATDLK(I)
(R)KIFLWGPVTDESSK(D)
(K)LIETLGLR(K)
(K)LLDYLK(R)
(K)LLDYLKR(T)
(R)KAGDTGSTEVQIALIDAR(I)
(K)ILGGYESPSGILELR(E)
(K)TFFPGTDSNWSHR(E)
(K)YFISLENFDSIPSWK(K)
(R)ANNVGIPLLVDNAYGFPFPNVVFGK(N)
(R)ANSVINLTSANPGQFIALDLFR(S)
(R)NLSLSVSDLYPILK(E)
(R)HGGGAFSGKDPSKVDR(S)
(K)EEVDVVEIASTVYYSSASQKPEVTR(V)
(K)ELVSSLASLGGVVHSPYLR(R)
(K)GISFFK(F)
(K)TVALLIITANR(G)
(K)VLESLLPLVVK(T)
(R)EAEEFANLFLELFAK(E)
(R)GLCGGYNSNVNR(M)
(R)TMEMVSTAK(S)
(K)AEYGNATIAFENIFK(S)
(K)SIISFEESIR(K)
(K)TTQLSQELAPLIVTIR(L)
(R)SILPVLEK(Y)
(R)VSLLNFGK(K)
(K)TMLQYLGNESK(K)
(R)EFTIEKIIQNCKTSFmR(R)
(K)GNIEQIEQLIYK(E)
(K)LLALLSFK(D)

(K)IVWLK(E)
(K)IVWLKEPTYPADSK(E)
(K)VGFMMIGK(E)
(-)MIIVEGIDYFLIPAENPEASAK(F)
(K)FYSDIFDFESLDEK(S)
(K)SGEYVIMGLDSINIK(L)
(R)GPETNQSGEFLHFLDPSGNVLEISYK(D)
(R)GPETNQSGEFLHFLDPSGNVLEISYKD(-)
(K)LILVSNHR(S)
(R)NKVDILPSVIVPIEESYSDIPIPSLVR(S)
(K)IGLTIPER(H)
(R)NFPEIANR(F)
(K)LAYFFSK(H)
(K)RIEEIKPEDLFTLIYTSGTTGMPK(G)
(K)SLVIGIR(S)
(K)VLGAIIVPDMEHLGVWCK(E)
(K)YGDRPVFWSK(G)
(R)AKDTVVLLGGENVEPVPIENK(M)
(R)GTDVTESELEYILNHSEAK(I)
(R)IEEIKPEDLFTLIYTSGTTGMPK(G)
(R)INEYGAISSGIQTYYTK(V)
(R)LEWILTDYAVQFSGAANVPR(G)
(R)VWENVYANIYNK(I)
(K)KHTTTELTPEEIHNIGLSEVSR(I)
(K)KIDQLIEGILFR(D)
(K)NMPIPIAMSELR(K)
(K)SLFFR(M)
(K)TLTYHETIPGHHLQIAIMQELEK(L)
(R)LVVDTGLHYK(R)
(K)TTEFTVSTPTALAGVR(G)
(R)FAVIEGTIAVSK(K)
(R)IVWLLGDVK(I)
(R)VVTGPNGGAEImVADSGIIK(M)
(K)IENFNSVLQEPTFK(S)
(K)SLKEEEAILGGSSDSDYK(I)
(K)SSLVPIWEK(S)
(K)TDNLISEPEFWKLSGEK(G)
(K)TISLEYEK(E)
(R)IITDVK(E)
(R)ILSFPEALQVALDGLK(M)
(R)KTGNTIPVFVLSPIQTPEGMDSK(L)
(R)LNVDAVIK(T)
(R)LSVYSFPSNGNNVGTYK(F)
(R)NYPLPEGLQQIQITRPGQK(D)
(R)SGQTISIYQEWK(E)
(R)SGQTISIYQEWKEDR(T)
(R)SNLTPGAMQLELVR(E)
(K)FAIPYLK(E)

(K)LASLEAEFSTLLK(K)
(K)SIHPSYLLTLDK(L)
(R)IIEFEQTHIQMVEPGR(K)
(R)LSIQIISR(L)
(K)FTFGPEYLIPKPFDKR(V)
(K)mNIPVFHDDQHGTAIITVAGLLNSFK(L)
(R)SDNPNQVNNVLGFPFIFR(G)
(R)VLFHVAPAVAEAAVASGSAR(I)
(K)LIEILGLSQAEFAR(S)
(R)SIDLKPAFISDLINQR(A)
(R)FSNENSNYPSPFLPLR(L)
(K)GDIEIHVTAR(Q)
(K)IPPTELGK(K)
(K)LNTLFQPNGIYVPVGK(T)
(K)SILQPTAQVVK(G)
(K)YPNGVTIVSPNATEK(L)
(R)KSILQPTAQVVK(G)
(R)LVMTSQDVLHSFYVPAFR(N)
(R)QWQWTFK(Y)
(R)YTTLTFTPTEK(G)
(K)LYESAYNLVK(M)
(K)VGDYVVYPIHGVGEILEISK(K)
(K)VGDYVVYPIHGVGEILEISKK(N)
(K)VIALLK(K)
(R)IRPIIDK(K)
(R)IRPIIDKK(E)
(R)LEDYTKPESPER(E)
(K)AIAGLYLSGHPLDKYEK(Q)
(K)MDILGLK(N)
(R)AHDTYSIQPINELFLR(L)
(K)IFHLSEDDAGDFFIYAFER(L)
(R)NMLIDWQR(T)
(K)EALNFAIGAAK(S)
(K)YIEDALHSVEGVAGQVNSK(V)
(R)KYIEDALHSVEGVAGQVNSK(V)
(R)KYIEDALHSVEGVAGQVNSKVEEVK(S)
(K)ESLWQGGPHITVENK(F)
(K)IIAWLSR(N)
(K)TGYYNYDTHYVFLSLPVAQK(F)
(R)ALPVENIHDLVGNK(L)
(R)FSLGK(G)
(R)GSLIFLVTSR(Y)
(R)VAGLLSLK(S)
(R)YNVENQVLIGK(E)
(-)MIRFLHTADLHLSLKEK(V)
(K)IGSLLIAYPGSPR(I)
(K)ISGIVEDEHIVSETLDNFSK(S)
(K)AIWDIAGK(G)

(K)ENKHPAGNMSILVGAEK(T)
(K)FHIGGSDASGIVYK(V)
(K)FNHWGALTSEINK(T)
(K)FNHWGALTSEINKTEK(F)
(K)GLNDLVIDKK(V)
(K)HLTWEESAAYMLVGATAYR(M)
(K)HPAGNMSILVGAEK(T)
(K)KMYAQVVRPER(Y)
(K)LGAAGVINR(K)
(K)MYAQVVRPER(Y)
(K)TFAWNETAHSHQLMK(E)
(R)YLWMR(Q)
(K)LYLFGTQAISDWK(E)
(K)ITYPMLFGMDR(C)
(K)STFENFLNSQTLSILSK(Q)
(K)EHVNLLVQAIENAK(V)
(K)ISVIPWTVNDVEDMKK(L)
(R)TTIQSFYLPALTIVK(E)
(K)GIGLALIQSEK(I)
(K)IKDGEPIQIEIR(E)
(K)IVSFALTEAGVPR(E)
(K)MIPFGGWDMPVQYSGIIAEHNATR(E)
(K)QAIITMKPFIPGSIR(K)
(K)TTSGTFSPSLK(K)
(K)TTSGTFSPSLKK(G)
(K)VDNQSLQWHQIALQGPK(A)
(K)YPLYGHELNDQWTPIESGIGWIVK(E)
(R)EAAGLFDVSHMGEIFITGNPK(S)
(R)FMLLPYQGEEIIVSR(T)
(K)EGESFDVAHTGIWTK(K)
(K)IPILLDYSVMVK(N)
(K)LVFEWLLK(L)
(K)MNVVFLLK(D)
(K)NIGPSGLSLAIIR(N)
(K)YLHITSNNTIYGTQYPEIPK(I)
(R)KIPILLDYSVMVK(N)
(R)SLYNTPSTYSIYIAK(L)
(K)AVVPTSSALELYLR(V)
(K)FLGITEDKTDLLPFR(Y)
(K)SNPEKDVINGGGYFIHYGIHPDR(M)
(R)AAVPASQNLTR(I)
(R)IISLNSEILLEK(N)
(R)VLLYFNPNDR(E)
(K)AAALFQEATDKYPDSPVGR(K)
(K)QYYGAIDTFK(K)
(K)VAYSMGNLLLQLNR(D)
(R)DEEGKGHLEEVLR(I)
(R)KNYVLALQEDPENVK(A)

(R)SNALSAIADYYMK(K)
(K)AIPNATVYTVYR(S)
(K)LGEVNHPDTEAQIK(M)
(K)LTELSHPESTFLDQDLNK(S)
(K)SQTIYYGVSVR(S)
(R)NFTTIPVVIEHALTPTENK(I)
(R)SAEPMSSLSLMR(K)
(R)YFYIGNPNK(S)
(K)AEVSLK(W)
(R)FLLNNGREDDGLLVIAR(A)
(R)LLFGTALLYNGK(A)
(K)RLNDIINVGIGAVQTSR(E)
(K)VNLAWGLVR(F)
(K)FDRGPLPNVLYAGImQYDGDLKLPER(F)
(K)LVSNSIASGDIFNGQAWK(E)
(K)QNIVLLVTADHGNADEMFQLDKK(G)
(K)TSHTLNPVPISILDPENK(I)
(K)VLLIILDGVGYSPK(G)
(R)AIEISLAFTEK(N)
(R)FLVAPPAIDR(T)
(R)GPLPNVLYAGIMQYDGDLKLPER(F)
(R)LHILLDGR(D)
(R)VWNQFPTLHIQAHGK(A)
(K)LNSTLPEYK(E)
(K)TSNFYVETPSAVAAVR(G)
(K)VHNVEGGILLYSDEVDSSIIKY(-)
(K)IAVVSDLHYGFLNPEFWVR(W)
(-)MEVPLTNLIHSAEK(L)
(K)ILPESVAINFR(K)
(R)HSPMSFTK(I)
(R)LSALLFNLLTR(H)
(K)AVELFDKFQTWLER(Q)
(K)ELPPPPGIR(Y)
(K)ETKPALFIDVYK(Q)
(K)LIPLSAISVGK(E)
(K)LISYQMPTVVIANR(L)
(K)LNEGIQNLEGKPK(I)
(K)MQLVGVLPGVAGSELR(Y)
(K)NSILLVDYAMQAIR(E)
(K)TAGLNVPVGK(F)
(R)GIEYVVK(M)
(R)LEQVQGVGSVK(L)
(R)NLRMAYAQTKVPNIANK(L)
(K)ILILFLK(D)
(K)ELSGILDENEKHER(L)
(K)ILDLNIDIDKDFR(D)
(K)YHGLIDAWLR(H)
(K)YIDPDVLEIIEDFK(I)

(R)ENTFSEILTAFYR(F)
(R)GNDLQLHGWVYDLK(Q)
(R)IWVEEK(L)
(R)LSVNEMTGTSAGELFVHR(N)
(K)FFGEVLPK(L)
(K)FIYAVYK(D)
(K)KYLIGIGTR(T)
(K)TTDKFIYAVYK(D)
(K)VWTNSDIKR(R)
(K)YLELTSLK(G)
(K)YLIGIGTR(T)
(R)TKNENEMGSNGNIPK(L)
(K)VLTVEEAESLPDSEKDIFMK(Y)
(K)ANLATYYFSTGDFEK(S)
(K)ANLATYYFSTGDFEKSIK(L)
(K)DEDEEVKELLQEGYEEIVGETPSFK(K)
(K)DVNTGNEPVSKPTGVR(M)
(K)ELLQEGYEEIVGETPSFK(K)
(K)ELLQEGYEEIVGETPSFKK(A)
(K)FLGTIR(N)
(K)GQSWGAKANLATYYFSTGDFEK(S)
(K)IDAVAAGLKVAGFAASMATGK(D)
(K)KAVVSSPAK(I)
(K)KSLWEAIFGEGSSFIDQMPSK(V)
(K)LYEEAMKLK(D)
(K)LYEEAMKLKDADK(S)
(K)RADAINEASLSLTGITK(N)
(K)RVEATFAVDESNAK(-)
(K)SLWEAIFGEGSSFIDQMPSK(V)
(K)VAGFAASmATGK(D)
(K)VAGFAASmATGK(D)
K)VAGFAASMATGKDVNTGNEPVSKPTGVR(M)
(K)VFEAFDKESYYK(L)
(K)VFEAFDKESYYKLTDLSK(R)
(K)VFVKDEDEEVK(E)
(R)ADAINEASLSLTGITKNR(A)
(R)KRVEATFAVDESNAK(-)
(R)LSPIVK(T)
(R)MMLIPLDATLIK(V)
(R)MMLIPLDATLIKVETGEVK(K)
(R)MMLIPLDATLIKVETGEVK(K)
(R)MMLIPLDATLIKVETGEVKK(A)
(R)NVGLAVEAPK(K)
(R)NVGLAVEAPKK(S)
(R)VEATFAVDESNAK(-)
(-)MKTLEEVAK(A)
(K)VAADTDADVILFNGVHFMAETAK(L)
(K)VLIADLK(A)

(R)MLDLSFKK(-)
(R)YLAANVQNLTQK(K)
(K)AEIPAMISAYK(S)
(K)ILYLGPETLYHLYR(A)
(-)MNQLLTLLQPETVIFNLESGSK(E)
(R)SVVLWLVSPK(F)
(K)IQEGKPAFINVFTEDPFIDLK(N)
(K)NLNILLSSGTGNLHVYPGIGGWK(E)
(K)QLNYVQDLNGSFFIANMKPAIEK(V)
(K)TFFENSIHK(D)
(K)TFFENSIHKDNK(N)
(K)VAGLTSYFK(T)
(K)ILPYEQGQIK(D)
(K)LESVVFWSR(K)
(K)MRPQYEEISILK(N)
(R)TNLLEIIR(R)
(R)AVEEILFESAMAMEDKETNSAK(I)
(K)AVLSMK(E)
K)LASQTSTSVQEIGSLVNSTNTAVLNGNTK(V)
(K)VAEAFNILK(K)
(R)FIPYVFQSGEK(G)
(R)TNLLALNAAIEAAR(A)
(R)EVVPFKNENELHRLEK(L)
(K)LLLNVELLK(G)
(K)ILAIETRPLSK(E)
(K)TVVILVETR(K)
(K)ANEVPDAIRK(S)
(K)ATLDALEQLETPILAAKK(R)
(K)GHTIPHEVIGK(F)
(K)SWGSSNPVNIVK(A)
(K)VGIGFGK(A)
(R)FSFNALSVVGDQR(G)
(R)RFSFNALSVVGDQR(G)
(R)SIVEKAGIQDVLTK(S)
(R)VILKPSTAGTGIIAGASVR(S)
(K)LILEWSSK(I)
(K)IALVFGSEQYGLSTYAR(N)
(K)NLKPILVIEGVEKPGNLGTILR(T)
(K)LAYSGFNDFLK(E)
(K)SIAILEELYPK(S)
(K)GTEMAFTGALYK(N)
(K)YESGSGWPSFYQPVK(D)
(-)MQNVLLQPEK(G)
(K)ELILLNELLLWK(G)
(K)GIFLDSVTSTNTVIK(G)
(K)LVPFLNESILLWK(D)
(K)TILSVYPELTR(E)
(K)LFLFGVPASK(R)

(R)YLIVQDK(W)
(K)FTSDYTIFDPLHGLTWK(A)
(K)EFYGNSLK(E)
(K)IISEVFPK(E)
(K)IVmTAAAK(H)
(K)LIWGK(V)
(K)LPNSNQPPSVNK(A)
(K)SQIIYEPR(G)
(K)TPMTLFGSK(S)
(R)QAALSSIDLMYPPYTNFVK(R)
(R)IGDPISLAQVYGVATFYTLYNK(K)
(R)IGDPISLAQVYGVATFYTLYNKKPVGK(Y)
(K)GLGPAMSALILGLTPGDR(S)
(R)ATLINVNIPK(N)
(R)LVFR(S)
(K)AAEVPILVAINK(I)
(K)AAEVPILVAINKIDLPAANPEK(I)
(K)IMQELANHGLQSEEWGGETMYAK(I)
(K)KKGDDPSTPSPAASPK(K)
(K)KMNLKPGDVIGK(L)
(K)LAMEGLLEPEKIEEVIGTAEIR(E)
(K)LLDTIR(R)
(K)MNLKPGDVIGK(L)
(K)TIKETLQGSADAGKR(K)
(K)VTLENMNEFIK(Q)
(K)VTLENMNEFIK(Q)
(K)VVGVSGVSVPK(E)
(K)VVGVSGVSVPKEITLLENVQVGELAK(K)
(K)YYNIIYQVVDEIK(L)
(R)GLITFLDTPGHEAFTSmR(A)
(R)GLITFLDTPGHEAFTSmR(A)
(R)GPGQGGGGYQGNR(G)
(R)GSVATVLIQNGTLR(V)
(R)IGNAGAATGTSSK(V)
(R)SGGTGTRPMPITSAEVELSQSR(G)
(R)SSVIDTESGGITQHIGAYQVR(T)
(R)TGPSRPNSGGGYQGNR(G)
(-)MQEPSQIVHR(K)
(R)KVLPGGITVLFQK(A)
(R)VGGFTNAATSR(E)
(K)ISAESFALDIVSYLHR(D)
(R)LSFLGVNSDYYR(E)
(R)YYHSSGNPNALEMVENTLLAMK(R)
(R)EVIINQPKNVVFTELR(F)
(R)FQKPFEGSFNSYITTQSVNEK(Q)
(K)GAVLSDFLSVLESK(R)
(R)SFLFGK(F)
(R)LINSSTELNRLGSGVLK(Y)

(R)ILNDLLQSAGQLADHYENNNK(K)
(K)LIPHLD(-)
(K)VNFNIDLLR(Y)
(R)ALESNYNLQNLR(Y)
(R)SSQSILPFNQANFFTGTK(I)
(K)AHLATSPESSVYLYLFPDAYK(K)
(R)ILINQAYATGK(A)
(K)KSSSSIPNNLEETQK(K)
(K)SSNTAGASSGTSTPK(K)
(K)SSSSIPNNLEETQKK(N)
(K)FFEITSQNK(G)
(K)FVVLEK(A)
(K)INQISQENPELILEAKNK(L)
(K)LRPTLVEEWITK(G)
(K)QSDWVISPSPSELKDESFLLVK(G)
(K)VVSAPTIR(S)
(R)EYKQSDWVISPSPSELKDESFLLVK(G)
(R)FIGPTLGIESIEVGMK(A)
(R)FITSAKINQISQENPELILEAK(N)
(R)LTSQNLTEPQVR(I)
(R)LVMDLLVNR(L)
(R)NLAIVWGDKVVSAPTIR(S)
(R)VFAYWAR(G)
(K)IEAVLLLLDK(E)
(K)IEAVLLLLDKEK(H)
(K)TEILPLLIVTK(L)
(R)VIFPELVSPLVTGAAMAFAR(G)
(K)IEQVGTPDEVYNHPK(N)
(K)VVILR(S)
(R)EPIEAQTIPAEIQYIHSTGR(N)
(K)LLNQTANEWITQK(E)
(K)SLPNSVNTILDR(S)
(K)NTLIPLSIAYFNSDR(R)
(R)IGPELTPVVR(T)
(K)IQASGLIVK(E)
(K)LAVYDYGVK(T)
(K)SFPGIINADLAK(V)
(K)SGYPLLTVQYHPESAPGPNDSR(Y)
(K)YIFGTHTGKK(F)
(K)GLEIGYLGPAGSFSNQAVR(S)
(K)IFRDQIDSLDREIVK(S)
(K)NWIAANLPHVEIVETSSTAK(A)
(R)FGASINALEFNSIPDVFR(A)
(R)FLIIGK(N)
(R)INISLLGLEHDLSK(I)
(R)NSWEYNFFIDFHGHQK(D)
(-)MLTLALPK(G)
(K)GWLSSKPDPNSK(E)

(R)EFFFLK(G)
(R)LSLAAPNGFTLEAHHR(K)
(K)IITIPLENIDFSSPGKR(L)
(K)ISNDKAEDILISFYDDIPNETR(S)
(K)LAADIETTHGISGLWLIK(G)
(R)SLIYQMIVAVSK(N)
(K)GVEVAFLVSK(S)
(K)IAGLNIPVSK(S)
(K)IAYTNQIADPK(E)
(K)IVVMISDFR(G)
(K)KLLSEVPLPLK(L)
(K)LLSEVPLPLK(L)
(K)NALIYYINHK(R)
(R)DFQIELLLGESGLK(M)
(R)EFWNFYVEPFR(K)
(R)GFVWSDGPLTSAIR(S)
(R)IDLIPIKR(L)
(R)KPLPFDITR(F)
(K)QIEDENLSVLEK(E)
(K)ILFEIWK(S)
(K)TGNPEIISFFLGK(V)
(K)AFLTWLFFK(E)
(K)FYLVVLR(N)
(K)LEGKPTTIYNTK(K)
(K)NIPLLSNSMEAVLGILPEEKPGHR(Q)
(R)ALQEFDYLFGTLFEK(F)
(R)TLPLDQLR(K)
(R)VTGFSSSDLLPSFLSEFNLSGK(K)
(R)NAEGITPLLK(S)
(K)MLPVVILTSSAEEKDIIESYK(L)
(K)TRVPSLILLDLK(M)
(R)VPSLILLDLK(M)
(K)SAITDLIDTKPNYTISISSSQWIR(I)
(R)QESVVLTLYEALSQK(K)
(K)LVNEMFYK(L)
(R)HLWWNFVSTSLER(I)
(R)LIAGEFLGK(R)
(R)YVGPFVFVDHMGPVSIQTGK(E)
(K)GLNGGVVSGTETPTQK(E)
(R)GPESFMEYWR(R)
(K)EFIIYPFR(S)
(K)VNPYFFLLQSLLDK(L)
(K)VQAIFQLK(I)
(K)MPIPLSFR(D)
(K)MVPPVPVLR(S)
(R)AIFSKPQDMEISFIGASK(Q)
(R)AILPGSSELQISYTIPASNLSTVTFK(D)
(R)SGIQEIIVYEK(T)

(R)VIALQQGMIPIK(E)
(K)FSLIEGR(Y)
(K)IILTSVYENTK(S)
(K)SESLLLNILPK(S)
(K)STVLFADIVGFSK(L)
(R)LVSSYQNLK(Q)
(R)TFFLLGK(K)
(K)TEILDELFHLAK(D)
(K)VAIVTGGSTGIGK(A)
(K)GNVPMYALR(V)
(K)LIGLYR(I)
(K)YIVSENDHDGYFQSR(V)
(K)AAVVGDTVGDPFKDTSGPSINILIK(L)
(K)KPSQLGEFFR(D)
(K)NLPTGSNVVR(V)
(K)VTSPGGTTIAGLVELEK(N)
(K)IIGANGGNVNSVR(F)
(R)FGLVLDTR(D)
(R)MFAQYFNTTK(N)
(R)NLWSTEGGITGLGGLR(T)
(R)SPQANLSGEHIFFGGLVR(A)
(R)VWDDEHNVGLK(D)
(K)ALILDLR(N)
(K)DFDNVVSHVSR(Y)
(K)EVVVENPLEGRPAVNAGIR(S)
(K)GLIVSTK(S)
(K)GVPGTLHIEVVR(D)
(K)ISTDDSFILFDPDYAEVEK(I)
(K)LIEGHEHIGYIK(L)
(K)LMPLGNDYLIK(L)
(K)NKDITNLPLAVLINAK(S)
(K)VALTIQR(K)
(R)EAAIYALLSLPHSIYLYPESYFK(E)
(R)GILLGER(T)
(R)KGVPGTLHIEVVR(D)
(R)VLAFIETNLDK(Y)
(R)VLAFIETNLDKYK(D)
(K)VATSLNPLGK(K)
(K)VEIIK(K)
(K)TIFPWK(Y)
(K)LVSHSSNGPEILAQSVR(S)
(K)VHPQIAK(W)
(R)ALFNVQYSFQK(R)
(R)AVITEMINQVR(A)
(R)FYESGDLLNLHHK(W)
(R)HLTSTLNESSMSPITR(A)
(R)YLLPLGTFTYLYHSVNGLTLHR(Y)
(K)IAYGLFPEK(K)

(K)LIELFFTK(R)
(R)GLELLK(I)
(R)IILEK(G)
(K)NLQEQGIDVQLVGDSSRPTTFK(I)
(R)EFVGGAGVVAAHVK(A)
(R)IALGNHEDGVEWIANTLLEK(T)
(K)AIVMQEGK(D)
(K)VISKPEFGFEIGK(A)
(K)VSAIVTVEEHTR(I)
(K)YGSQESLLNYFGINK(D)
(R)LMPLTLDWPHPIYIR(L)
(R)SIFIEK(Y)
(K)ALIANFTDIPHDPSEAER(L)
(K)IQEIILGRPENYQR(V)
(K)LILFDPR(E)
(R)LPEHGSFIPFTWG(-)
(K)MLNGLLLQLNDIAK(R)
(K)NVILVEDAAHVLKPTK(G)
(K)ADIANKEEVSSIFQEHK(F)
(K)EILSGQYK(E)
(K)FAFESALK(E)
(K)TNVLGTFYLLDAAR(L)
(R)SILVSTMDLK(K)
(K)AFTSFYLAK(D)
(K)INLEFVSANPTGPLNIVSAR(A)
(K)VIISQQVNLLESGQK(V)
(R)LLFWIAR(F)
(R)LSGAVQSLGYK(K)
(R)VTNYLQSFAK(A)
(K)VVFSR(I)
(R)ISGKPGDTVQMK(N)
(R)NNHPEDISGVGSGFTLQFEDKR(G)
(R)SNLYLGDLVLVK(H)
(K)KPQIHESVFLAPGSQVVGDVVIGK(N)
(K)YKPHAIILDLDLPGIK(G)
(K)YSESIIIKGIK(S)
(R)LLDETALFLHR(M)
(K)NFFPLTNDLMFK(I)
(R)SYLDIR(A)
(R)DPLKFPDFIHSQK(R)
(R)LTSAIASTMHNLPKPLLEANLK(H)
(R)ALGVSPSYLSK(I)
(-)MNQNSEFDIVTLIELAK(R)
(R)AVAGFHTLDR(I)
(K)IQVPTILMK(K)
(K)TKPFVLYYNFIGPISEEGNLER(L)
(R)TNSIITGTPETLLFK(T)
(-)MIQNILR(V)

(K)EAEIVANAGKPGAVTIATNMAGR(G)
(K)ESQTLASITFQNYFR(M)
(K)FQAILTEIRELQSK(K)
(K)IYNLDVIVIPPNVSVQR(K)
(K)KLIPIVR(Q)
(K)KQPVLVGTISIEK(S)
(K)KVGQEITGGFQEFSGGNLNR(S)
(K)LAGMTGTADTEAEEFKK(I)
(K)LIPIVR(Q)
(K)MLASAGIQHNVLNAK(F)
(K)MVSNAIAR(A)
(K)NEIVNFIFR(V)
(K)NHEEVLAAGGLHILGTER(H)
(R)ASEILSSVK(I)
(R)NIFLDILDHR(W)
(R)NPLVEYK(L)
(R)NVLLTEK(G)
(R)QNLEYAESLMQK(M)
(R)RYSDGLHQAIEAK(E)
(R)SQSNGSSVTVTTSSGGGTER(K)
(R)GGQNSYPSGSSFPGYQHR(D)
(K)FLNLLIESALR(Y)
(K)LLQVSGIADVVTFGGLPK(Q)
(K)TVNGVPVYVR(D)
(R)ALFGIAVR(F)
(R)EASILSAVSQLRPR(L)
(R)YSVTVADVIEAIK(S)
(R)YVVESTAGTHTPMELR(T)
(K)KPAADTVAPAPPSQTIELEEK(K)
(K)NFAVNAVALTIQK(A)
(K)SSPWGFR(I)
(K)TFTVTPVWNFTENLLIK(L)
(-)mLFEITIQQFSK(M)
(K)KFDVEVLLNSR(L)
(K)NYLGEMPFKS(-)
(K)TLSELQTR(I)
(R)LAPDQFHFIR(Q)
(K)LFSFLTHLLKNR(C)
(K)GRGEMIDKR(F)
(K)LIPSEIEFYSNILPGLSGK(N)
(K)NAELPTILK(G)
(K)NVILITNPSGIGR(S)
(K)SPVTVDEFFNLPIYHIYR(V)
(R)GTTAPFVYFGAPWMTEPEK(L)
(R)LIVLDHVNPALYLK(G)
(R)VKNAELPTILKGADAILFDVQDMGMR(C)
(K)SSVSPVLALNPSLK(V)
(K)RMIQFLSTHPLNDERIGENR(K)

(R)MIQFLSTHPLNDER(I)
(K)ILQPFLTYLNK(S)
(K)INFGASIGEVGK(D)
(K)LYGQEDIADEVHFAK(T)
(K)MLDLK(F)
(R)KmLDLK(F)
(K)NLFSIHK(L)
(K)NLFSIHKLDYARGK(R)
(R)IVIYEPYEMQK(E)
(K)ASLILLR(T)
(K)IPQIPGR(F)
(K)TSLTNILFSLAK(D)
(R)FPDLLIYALDDIYR(K)
(K)GIPLIHR(S)
(K)LILNGANVFHK(S)
(K)LVTLGAGNVR(D)
(K)NSDIAGAEKTSKVLYGIDPDK(I)
(R)ILNTESVSPGLAK(V)
(K)KEDLQGGVFTFIK(G)
(K)KTGLSEFILPEDKIETTK(D)
(K)TGLSEFILPEDKIETTK(D)
(K)SFKEYILSYR(N)
(K)TGLPDFGVILVSYIPNKR(C)
(R)NVGIFHEFLVNK(I)
(R)VPGSLSNYNTGANLDLVFQK(L)
(R)ISEEILFGVLK(S)
(R)SGLSEPGKPVGSFLFAGPTGVGK(T)
(R)MFSYLEYSPGTLMK(M)
(R)MQTISGVHYNFSFSK(V)
(R)SNLAFTQLILLDSLLK(V)
(R)VNPDGTLSGTTHPIHLGSSLTNHYIK(T)
(R)VISIPVNFFIPVK(G)
(K)LKPILLPYL(-)
(K)NVEFLDGWQFLR(E)
(K)SANIVFVGDSLIQLFPK(E)
(K)LLNDFDGKIPK(T)
(K)NDPVQVEKDLmSLLPEKYWR(D)
(K)TANVVLSEVHGLVEGIVVDTHVNR(L)
(K)TIPELITLPGFGR(K)
(K)NPFTGVGPGNYNR(E)
(K)NQIDTIQWHVEDEIR(R)
(K)RKLEVLLEQR(K)
(R)ISSEFQNVPK(K)
(R)MSILELK(I)
(R)RPDLPDKELVGFK(R)
(R)RPDLPDKELVGFKR(Q)
(R)TDLVEILDDRISSEFQNVPK(K)
(K)IPFVTSAVDLHSGEEYVMDR(G)

(K)IPDILIR(E)
(K)SGSYPILLSNR(I)
(K)SNLVSAGVALVATDGK(T)
(R)ISPQIFGMFSSPFSADSVQK(G)
(R)SGLDLDKINPELIVSTATER(S)
(K)AYGIKDPDLIFDVPR(S)
(R)VVTFHYTLHDTEGNLIDSSEGK(T)
(K)LLNDISLGKNIVSMGVSETGWKGR(I)
(K)NIVSMGVSETGWK(G)
(K)EILGNYPGNLIIIEGHTDNVPIR(T)
(K)IKEILGNYPGNLIIIEGHTDNVPIR(T)
(R)ALSVLHYFLESK(I)
(R)RVDIVVVPR(-)
(K)AEQNAFLDLFLK(Q)
(K)ANPSSQEWIEFFSEK(I)
(K)LSSSVLTSAR(R)
(K)NLFLAPLNTK(V)
(K)TGFTSIHYDTDQEEIAK(I)
(R)KYDLVSVAVLDDLGR(I)
(K)SISEIVNQSR(K)
(K)TNLLALNASIEAAR(A)
(R)GFAVVANEVSK(L)
(R)TIGIIAQQLLINSK(K)
(K)SLIADTIK(E)
(R)EIAAELDVSTGTLYHYFATK(E)
(R)IESLFQMVQAR(E)
(K)IIESLFDYFLK(H)
(R)TLTNFLVSDLIQNIAHR(L)
(K)ITEGNPIFLK(G)
(K)VYLIWPK(V)
(K)LIGFELPENFK(A)
(K)AAATKGNSPSAVVVFSVGEAK(I)
(K)AAVLEAER(K)
(K)ASKEDQFSVVTPTAIAGVR(G)
(K)GNSPSAVVVFSVGEAK(I)
(K)ILHADLTEEK(A)
(K)ILHADLTEEKAALGASLK(T)
(K)INQADVQEVIYDFQTKAK(F)
(K)IQQTVASSEIVLEK(N)
(K)IQQTVASSEIVLEKNQASVVK(A)
(K)KINQADVQEVIYDFQTK(A)
(K)NITGVVK(K)
(K)QVLISAPK(E)
(R)ISENSVIDFDALSINSHGNSDTR(L)
(R)KKLESEVAAR(Q)
(R)LALVSGK(V)
(R)TIVTVDKDTTEK(M)
(R)TIVTVDKDTTEKMVR(L)

(K)ASISAELSNVVDTSNATTLGDGIHK(I)
(K)GQSITVSLDR(E)
(K)ILDLFGDISIAGRPIIGHYLASK(A)
(K)VTYTIDFNHPLLK(G)
(R)RIPLFK(R)
(K)HMFDLYAQR(R)
(K)ITAYLAFGDHNGIYNLGR(G)
(K)KTFLEHTTR(N)
(K)LLAEESLK(N)
(K)LSINIEYIEMPETLK(S)
(K)TFLEHTTR(N)
(K)YFNVFGYGEGHK(G)
(R)LGIDDILVVDHLGTSSK(W)
(K)TLLGSMLR(I)
(K)TLVEVLGAPDKYK(A)
(R)IWALDLPDESTQPAKK(V)
(R)VSEWIASSPK(E)
(K)LTLQNHPVR(N)
(K)TVMMVESQNK(K)
(K)TVMMVESQNKK(V)
(K)VITIQIEALENGSK(I)
(K)EIFDSPGFSLLEHLGFTK(E)
(K)EIFDSPGFSLLEHLGFTKEDINK(A)
(K)FEPNGIVSGNK(H)
(K)FNLGENFLTK(N)
(K)GHATLNGAPVINTQALK(E)
(K)IINQSVPYGLK(K)
(K)MSTSVIDYIFR(E)
(K)SQGHYYVDPSTGK(L)
(K)SSGLMSFLK(I)
(K)TINLPEEAVIEDIKNAYFLSWK(M)
(K)VASLVTGSMLNNR(H)
(K)VEASLPLAFDINFAFNK(F)
(K)VFEANDILVPEDWSQVAVDILAQK(Y)
(K)YGSGTGTNFSNLR(G)
(R)HFTVPQNGESSTIQWTK(R)
(R)IMAAAQPFISGAISK(T)
(R)VIDLAR(Q)
(K)GEAFGQFNSGLSK(E)
(K)LLVLVSEFDVK(I)
(K)NIAEIALAGGNPTFNR(R)
(K)SVLESLGFGVVEEPDLISIVK(I)
(R)NAGTGGTNAVTVDNYEYRPTGIK(L)
(R)TITQVIPIENVKPEELEPILK(R)
(R)TITQVIPIENVKPEELEPILKR(L)
(R)IYVESTPSMVK(I)
(K)LINRFTQSKFPLK(L)
(K)FVDIVGHYHPAQIK(E)

(K)NGAQPTGTVLNLFK(N)
(K)ALEYNIPVFQYESIKK(E)
(K)IGYFGTPEHSAK(L)
(K)ITDAGIESILQLLK(T)
(R)ALYPDMIATTTFR(D)
(R)SKIPEPGPVKK(K)
(K)VFIDGQDVTDSPMHIR(A)
(R)ILISGSTHDLVNDPETR(R)
(R)LGVGYLAQEASIFR(K)
(K)GQFLAPADTR(H)
(K)IPYIFK(S)
(K)KGQFLAPADTR(H)
(K)YNVPVLTDIHETSQISPLK(D)
(R)EFIPSILR(S)
(R)GTSFGYGNLIFDGR(A)
(K)NSVSTGQIYNTVIQR(E)
(K)QTVQIAVVGK(Y)
(K)SILIHER(H)
(K)VHGILVPGGFGDR(G)
(R)KNSVSTGQIYNTVIQR(E)
(R)SIYESLSHGGIAHDTK(V)
(R)SIYESLSHGGIAHDTKVEFIK(V)
(R)TVQVVPHITNEIR(N)
(R)YEHGSSNVLFVHLTLVPTITAAGEAK(T)
(-)MIINHNISALR(T)
(K)QSNKPIQLQVGPNQNEK(L)
(K)SAEFNHIKPLSGDHSK(Q)
(K)SGSAMLAQANMRPDQVVK(L)
(R)ELSIQTSNGIYSNEDRK(L)
(K)DKIDPNKPLTYLMQTTLSVADTK(N)
(K)IFYPNANVELFPGSR(D)
(K)ILGLTAGASTPQVLVDEIISK(L)
(K)VDKEILDLLLTNNR(V)
(R)IKELENVNKK(L)
(R)MYEGIIQYSGK(E)
(K)EIARLQNLRSLLLNQNR(F)
(K)AMFIGIEQVRPGNR(V)
(R)DLMGHGIGR(G)
(R)GFHEDPQIPHFR(Q)
(R)TFIVGGNTSSEVK(T)
(R)VHDIANAIDDFLTPK(G)
(K)IHQLIDDAVK(R)
(K)IHQLIDDAVKR(T)
(K)SKGFMTSGDAIDGLNEK(I)
(R)STVRPTDF(-)
(K)NIQIIQSVAK(D)
(R)GIKFGIRNEVLNLLSEEPEYGK(K)
(K)DKDWFLIDPIK(R)

(K)EIIFQDFITIK(K)
(K)FYAIGNYEVK(K)
(K)LAFVVLNGENWKPEDGAEFVK(I)
(K)WNGNVYIQNTSSSR(K)
(R)KLDFQILEMALE(-)
(R)SQGIAQSNHLQFTSSNLNEILNR(G)
(R)SVTINFR(K)
(K)ERPDLVVYTLEPSEGDYLK(E)
(R)SVLVVGVSETSK(R)
(R)DKPFSDHAPLEFEIKL(-)
(R)YLYPDKQEYSWWTFR(A)
(K)LEAYSELSEALKDYIQK(D)
(K)SILPTKPAILSELNR(I)
(K)TKLEAYSELSEALK(D)
(K)TKLEAYSELSEALKDYIQK(D)
(K)NSFEIVR(M)
(K)SATNLMELIADSDKFPDLLEK(A)
(K)SLLAFK(T)
(K)YLEEEDLSSIHPITESINHLDR(I)
(R)ITESIWNILR(T)
(R)KSATNLMELIADSDKFPDLLEK(A)
(R)MHETEAFDLLR(A)
(R)TISSMLASLNVAR(K)
(R)TSSNGISSFHEISKDEKEEVK(E)
(K)IAKEENLILVPFFLDGVAGIK(N)
(K)ILLLGMK(T)
(K)LNAILFEFR(E)
(R)FILIPILK(A)
(R)LFGLLASSR(L)
(R)LITYLQTEYGK(V)
(K)AVVEVLVNNAGFGTNGK(F)
(K)AVVEVLVNNAGFGTNGKFDR(M)
(K)AYVLSFSEAVYEDVKK(D)
(K)ILAQSVR(I)
(K)ILNVASIAGFQPGPNMANYYATK(A)
(K)SVVIAGALNK(I)
(K)TAIITGGTVGIGYELSK(L)
(K)VKVDILSLDLADPK(S)
(K)FLRPDAK(S)
(K)HLAGLR(H)
(K)IIVDTYGGYGR(H)
(K)KVIPANLLNNTK(Y)
(K)NVVASGLADK(C)
(K)SQVTVEYK(D)
(K)SQVTVEYKDGKPVR(I)
(K)VIPANLLNNTK(Y)
(K)YFINPTGQFIIGGPHGDAGLTGR(K)
(K)YRETASYGHFGR(K)

(R)ETASYGHFGR(K)
(R)GIIESLK(L)
(R)HGGGAFSGKDPSK(V)
(R)IDTVVISTQHSPDVTHK(Q)
(R)KIIVDTYGGYGR(H)
(K)YIGDAVMATFGTPHPSPSAEVDAR(N)
(K)LLVGEK(I)
(K)TVSEIPLQGNILGIGTR(S)
(R)SIGILSPQENHQTLAQWK(E)
(R)TFHDLEEWENFLQSK(K)
(K)LFEIGSFQATGNK(L)
(K)VGADIRPIVIGQVELYR(F)
(K)GTESVLEANPYIDSLIPLDKK(E)
(K)TNLPELSFLVSK(A)
(K)ILISEEEHIDWIESQQEIIR(Q)
(K)INVGNNIEEILK(V)
(R)GIVIAVK(N)
(K)AAGTENFLENPNWIFR(I)
(K)AIIADLKHALLDK(M)
(K)FPAELQNAFVNMENNSNPWGVGAHTR(A)
(K)HAHLIFAPFNYFLQSDTPK(G)
(K)HALLDK(M)
(K)IRTEETDAAVAAEK(V)
(K)MPEVAK(I)
(K)NEYPQFGGNFEVVHHSEFINQLVK(E)
(K)SVGAVYDSPTENASFLAAGIGSIWR(S)
(K)VLNPKAIIADLK(H)
(R)GGNEYLYQTLAQANVDTMNGYNVKK(V)
(R)HLVLAESK(F)
(R)INSFIQNVILQK(K)
(R)RGGNEYLYQTLAQANVDTMNGYNVK(K)
(R)AVPEWFSQGSTSIQYIPVHIDFK(-)
(R)FLFMEIYK(N)
(R)RAVPEWFSQGSTSIQYIPVHIDFK(-)
(K)KGSVLAIVEAMK(M)
(R)IGVSVGTTVK(K)
(K)SVFPPLVFPVLYK(A)
(K)FFIAQTGYTFFDR(F)
(K)LWFGDIFR(T)
(R)VNPWAPNINDR(Y)
(K)IWLALDGK(D)
(R)LTAFLNPEEFK(Q)
(K)GAIIEALVGK(V)
(K)AIQDNLSEVDFHYR(I)
(K)IIGEIAGSVYK(R)
(R)IYDTELLHQENQNLK(E)
(R)VFASNLLTPEHVGSFR(L)
(K)DLQDIIAILGMDELSEDDKVLVAR(A)

(K)ETIIAEVQTHIGGK(A)
(K)FLSQPFHVAEVFTGAPGK(Y)
(K)QHGGFSVFAGVGER(T)
(K)TGLFGGAGVGK(T)
(K)TVLCYGQMNEPPGAR(L)
(K)TVLIQELINNIAK(Q)
(K)VIDLLAPYIK(G)
(K)VIDLLAPYIKGGK(T)
(R)AAPSFDELTSKTEVFETGIK(V)
(R)AIALSSTDGLIR(G)
(R)AISDKGIYPAVDPLDSTSR(V)
(R)FSQAGSEVSALLGR(M)
(R)GQEVSNTGKPISVPVGDATLGR(I)
(R)IFNVLGK(T)
(R)IFNVLGKTIDEGPAITVK(E)
(R)MPSAVGYQPTLSTEMGALQER(I)
(R)VALSALTMAEHFR(D)
(R)VMNAQVLGEEHYLVAR(E)
(R)YKDLQDIIAILGMDELSEDDKVLVAR(A)
(R)IGGSATEGISR(Q)
(K)ETIFTDHETGK(R)
(R)FANTVEVFVNFLR(K)
(K)ASPLGGEFLR(S)
(K)FLNASVGNFAETMADIK(S)
(R)VGQFVSSEGNK(F)
(K)DELYLLHLAFR(L)
(K)GLNTVSSAVK(N)
(K)VNLLALNAAIEAAR(A)
(R)GFAVVASEITR(L)
(R)IDPVTDKIEELIQLQLK(I)
(R)VYAEILSGLEHLK(E)
(K)GKNPGLGLPMVFGFVK(R)
(K)ALELQNK(L)
(K)ANLVAAALANGVIPAHNVTLDLK(N)
(K)IAYITIPKPTR(A)
(K)LTGISIPEEANKR(F)
(K)RAHDDFGFLR(M)
(K)SPGQFEHELLR(R)
(K)SPGQFEHELLRR(G)
(R)AAQVEEMITYIQK(A)
(R)EIPIHVLIETNGALQEVEK(I)
(R)IHDYTNSFWK(Q)
(R)LQNSELNK(H)
(R)YFWEILQK(A)
(K)ANLYYVR(L)
(K)SVPLYSNFDSYYR(E)
(K)VEFGNHVLFSK(Y)
(R)NPASAFWYR(D)

(K)EYGINLSDPKPDFAAIIR(R)
(K)GINLPGTPISAPALSEK(D)
(K)LNQAGKPVITATQMLESMIENPRPTR(A)
(K)VVILSGAPGTTVR(S)
(R)GVVPFVMPFFTR(L)
(R)SGYSALITSEMRPK(V)
(R)SYLEGTYTGLIAK(I)
(K)IELAR(L)
(K)ILGLESSGPHSNGFSLIR(K)
(K)IMESSGEVVHEIGEIVSGNKEEVQFV(-)
(K)YKHPILLSGTDGVGTK(I)
(R)ILPQGTQSK(F)
(K)GQSLIVHTPTVVAAVR(G)
(R)VAAKEELAAMEQIDQMK(-)
(K)GAYSFPFIK(K)
(K)KMTGPLEEILK(L)
(K)MTGPLEEILK(L)
(K)QAVSVYGQTGAGGAAPR(V)
(R)AAGFQNAFLYGNR(W)
(R)EVGYLDVHTYQGSWFEWR(K)
(R)LPGAFLWDWR(I)
(R)MVYLLR(A)
(R)SASGYQEATLK(G)
(R)SSLLALMLR(E)
(K)NAENVIQLLVNLQK(Q)
(K)RPSLILADEPTGNLDTK(N)
(R)ITVAR(N)
(R)GMIALLIR(V)
(R)NGLYSMVNILR(N)
(K)GQPNLSGFK(N)
(K)ILTAEVPYVLLWNTSATR(I)
(K)IYNSKPIGFQGLVFNIR(R)
(K)LIEQQRAEFSVPKR(N)
(K)NNNTAVFR(I)
(K)NSEVDKLIEQQR(A)
(K)VYNEEPIAFQAFK(K)
(K)YLTPIQER(A)
(R)AEFSVPK(R)
(R)FEKPVVLNER(E)
(R)IMYWNR(F)
(R)NFDKENFDFPIVSGPYSMLSAK(K)
(K)GATILDFAFR(I)
(K)TNMYQSLHTTVIGPDGKPLEVQIR(T)
(K)IHKIEPAALSTDFEIISAQR(Q)
(K)LIQFESR(G)
(K)SPAGGVYTDLK(K)
(K)SPAGGVYTDLKK(-)
(K)VHPFSITNDQVAEFAWNTMK(F)

(K)DGLDAATHYGPVR(S)
(K)GAMVGGNLMVGYESDFGK(Y)
(K)GGYDILTAAGAGAVANLLSDGTDPVTTR(E)
(K)TQSVGGATNLSPFPAYPIVVGGQIYR(F)
(K)YFFWR(V)
(R)KAIPAENR(L)
(R)LITLDR(T)
(R)STKGAMVGGNLMVGYESDFGK(Y)
(R)TSGIAPNFLIGTQAR(V)
(R)VAAEYTQK(I)
(K)VIAHDPVTLNFLQITYSR(I)
(R)SSLEIWPK(T)
(R)VIALDFAGHGESDSTLNFR(D)
(K)NVETLTNLGFTDLFK(N)
(K)FIPTTIPYIK(N)
(K)VIGTLEILNK(T)
(R)ILEFLEAR(T)
(R)LVLYTDGISEHYSEDR(T)
(R)SFDSSDLNYLTSLSEVAAIAIQNAK(I)
(R)TGTIYLADHTNQTLR(I)
(K)ITPSPGTITISGANYSNR(A)
(K)LDSGGTNFVTWLTTLSTGTKPGNAK(C)
(K)DVMNLEPLKEK(F)
(K)GSPTIILFK(T)
(K)ILNHKPSDPDWEERDR(L)
(K)MVTAANSGHPGGPLGLADIYAVLYK(K)
(K)MVTAANSGHPGGPLGLADIYAVLYKK(I)
(R)YAAMAHSGYFPLEDLmTFR(K)
(R)YAAMAHSGYFPLEDLmTFR(K)
(R)YAAMAHSGYFPLEDLmTFR(K)
(R)YAAMAHSGYFPLEDLMTFRK(L)
(K)HLENMNK(V)
(K)LYDVLALGWVGSNK(A)
(K)TVMPHKDGGHH(-)
(R)DVTEDILRPVEAFPTSLWWK(A)
(R)GPLWVNFR(S)
(R)IMPVIAVAEVK(T)
(R)KLYDVLALGWVGSNK(A)
(K)IILTASGGAFR(D)
(K)RYPGLGLAFEAGK(V)
(K)VVPVDSEHNALFQLLESLNPNSVEK(I)
(K)DMATIPEIMFVIDPK(K)
(K)DMATIPEIMFVIDPKK(E)
(K)DMATIPEIMFVIDPKKEEIAVK(E)
(K)FIMDEDPDSKK(T)
(K)IFAVVDTNCDPELIDYPIPGNDDAIR(A)
(K)MAPYVFTAR(N)
(K)NLLETGVHFGHQTR(K)

(K)TLGGIKDMATIPEIMFVIDPK(K)
(K)VLFVGTK(K)
(R)AISLFLETMSNAVIEGTGGVVEQPR(F)
(R)FSEDLDSEALALEYQGEYDESGK(F)
(R)NGIHIIDLQK(T)
(R)SNMFFINNR(W)
(R)WPGGLLTNWNTVK(R)
(K)YGMWWQVTPK(I)
(K)FQIPFILIEK(M)
(K)MISVSGSYGSDR(R)
(R)AIPELIELYLK(G)
(K)AILFRPEIR(F)
(K)GISNNYFIGNLYENLK(N)
(R)FGGPPLITTLAR(K)
(K)AITYLK(Q)
(K)ELLTSFNNR(M)
(K)EVPYFTDLEKER(L)
(K)FDLLNSFIK(S)
(K)FKIFDDLIEFYK(S)
(K)FLPVAEWSK(W)
(K)GYVNLVR(K)
(K)HAHNPEEVLTGILFEVPIK(V)
(K)HIISYQSPLAK(S)
(K)IAELIK(S)
(K)IEEYLSEHEDSISAR(Y)
(K)INIGVTAK(L)
(K)ITSISPNGDSIFVDFKDK(K)
(K)IVAFLYLQTASEELGDEEIPR(H)
(K)KGYVNLVR(K)
(K)KILEHEASSNK(A)
(K)KKDELVYR(E)
(K)KLDIAMEALDNR(E)
(K)LKNESTGEVVAYSILGAWDADTEK(H)
(K)LSIQMAITSLKPLK(R)
(K)LSIQmAITSLKPLKR(D)
(K)LSIQmAITSLKPLKR(D)
(K)LTSLFNEEIYVR(T)
(K)LYALYK(E)
(K)NNIPDFFK(E)
(K)QLEDWDKVIYLLK(K)
(K)SAIILDLTNVK(T)
(K)SLPVSSLIEVSTK(I)
(K)TDKINIGVTAK(L)
(K)VIYLLK(K)
(K)VWEGEWLLSSK(Q)
(K)WAIIEHITDQILK(Y)
(K)YALSILEENK(E)
(K)YVNHSLLEDFLK(M)

(R)EDAEGAIEAFNHFYYEEEEAADPVR(K)
(R)FSLQSQEN(-)
(R)GEISDSGLLKNLLESFK(V)
(R)IPEGAILVTR(R)
(R)KIEEYLSEHEDSISAR(Y)
(R)KKEEFEHLLNVEMPENSK(D)
(R)KLYALYK(E)
(R)LVGYLYPIVEPYK(Q)
(R)MLTADIK(S)
(R)NIVFDTNNYVLHR(N)
(R)NWGVGK(I)
(R)YLLGILSLER(G)
(K)FQFTYPGENAVTIRPPR(I)
(K)IYGVEFPGVSK(A)
(K)LISGKPGDIK(N)
(K)TIVFK(Q)
(R)IPEYEILR(T)
(R)SRPDTSGETVYLFFDELR(V)
(K)EYGISILSNLKPEEESK(N)
(K)LQIAYYQMIQADR(M)
(K)VKNDIHALATVTK(N)
(R)YKDSLMHFR(I)
(K)IIHPANFGVQK(D)
(K)IILNPR(N)
(K)KKEYFASVDPISLVVTGK(G)
(K)AIIEEAIRPLSLLNVQDK(Y)
(K)DIKDYLQNR(K)
(K)EIWAVGR(R)
(R)IKPEFRPAVK(K)
(R)KAIIEEAIRPLSLLNVQDK(Y)
(K)NGGTLILDFR(K)
(K)STLQEILSGMK(F)
(R)KSTLQEILSGMK(F)
(K)FSSVETLISQLK(Q)
(K)FSSVETLISQLKQDEILTR(N)
(K)VDPVYLNDTK(L)
(K)DFLHFSR(T)
(K)LFGFLETITEK(G)
(R)VLLVAVSDFASILSLFQETHLVK(V)
(K)FFLSDQSNYITGTYLR(V)
(K)KLTDPAFIPLR(R)
(K)LDFLVNNAGVLK(D)
(K)LDFLVNNAGVLKDNLFIR(M)
(K)LTDPAFIPLRRPGTQQEIANAVK(F)
(K)VALVTGAASPR(G)
(R)IVNISSVSGLTGQPGQANYSSSK(A)
(R)RPGTQQEIANAVK(F)
(K)LIFVPQGK(D)

(R)IQIDR(L)
(K)MREENIGPMILR(S)
(K)FIMHGPDGTDYPNLIVFLEVVRPEK(L)
(K)HGSDMKDHPGDFHVTVLFSEQNGK(T)
(K)TILDMTMLFNTAQQR(N)
(R)MWTDPEHVIHWWGPK(G)
(R)FTMHGPDGTDYANHSVFVEIIKPEK(L)
(R)MLFESLQEFEK(A)
(K)LVAISQMSNVTGTIHDILPVQK(R)
(K)MLGPTGVGVLYAK(E)
(K)STYAELPSK(L)
(R)ASLYLYNTK(D)
(R)GGVISFNFPGVHPHDVGTILDEEGIAIR(V)
(R)IRTDFPILGTK(M)
(K)IPDLVK(L)
(K)IPLSNFHPEDFTEVPDFAAVVWK(I)
(K)ALLLKPVDQQLLFETALAYANASK(D)
(K)LLADTYILYFK(T)
(R)ALGFYAPSSSHQLGK(L)
(R)HLVSGHETVIR(T)
(K)IYEGLYHETMNER(I)
(R)VIVVQHGIGEHSGR(Y)
(K)GISSPFVATR(Y)
(K)IVSAPSIMHGK(I)
(K)ITASPLSQNIEFK(N)
(K)NLFFSYPGTGSAVLK(D)
(R)FQLQMTLPEQR(I)
(K)GLDLYLK(R)
(K)VTWEAYYTFSK(T)
(R)GMWQMLISR(G)
(K)ASFTVSSSIPEEFK(F)
(K)DIKPGAEFVFPGEEK(T)
(K)TTEETLSLLISSLK(E)
(K)AIVTLGNPSTR(F)
(K)VMDLLGWK(R)
(R)GTWGSFFGIPVMPTYHPSFVIR(N)
(K)EIFLGGVSLGGQAILNSLK(A)
(R)LSLLNGHLQEVISGIR(V)
(R)LSTVQIANRIFTMEDGK(I)
(K)IVFDPTSSYHSLR(L)
(R)ILLSPILEEDLYSAFR(G)
(K)IGFIFQNFQLIK(S)
(K)ILFADEPTANLDKK(N)
(K)SLNALENVSLPLVLNSK(L)
(K)NIPLNLPETSGQNVHLFHAGTLK(K)
(K)NIPLNLPETSGQNVHLFHAGTLKK(D)
(K)NSVQSFLK(Q)
(K)SIPIVIK(A)

(K)VKEQIFDPMFDLFR(K)
(R)GLLYAGLMISPNGEPK(V)
(K)IISIVPIAGQEALSVYK(T)
(K)IREENWFLETILPFGK(N)
(K)LDNVVYHLSK(A)
(K)NVILERPTHLAK(R)
(K)AIGAPEHPHR(G)
(K)LKPGDVQWMTAGSGLVHSELPSNEFQK(N)
(K)NGGWMHGFQLWVNLPSSQK(M)
(K)TIRPSLR(T)
(R)GFETVTYLLSGEMEHR(D)
(R)TMEGGGFPVR(R)
(R)YGPFVMNTQEEIYQAFHDFQSGK(M)
(K)MDLIESPELLAHK(D)
(K)SIPGVFSVR(D)
(R)AVDNAMAELPAQVTEKPK(M)
(K)ILQSMIALGELK(L)
(K)TITLEDVAEMIIK(G)
(R)LVLGGIGANYLTR(D)
(K)LVFNQLIDWK(E)
(K)VIFPK(A)
(K)YGGMGLPATVQSFMMEVAAR(A)
(R)IQFGKPIEQIPAVR(K)
(R)TISQAYNLDSIAITSANLIK(L)
(K)AFFSTDR(I)
(K)IPNYGNVGSQGIR(R)
(R)SLFHQAAK(T)
(K)AINHLDEIKNPDLR(E)
(K)AIQEQEALWK(N)
(K)AIQEQEALWKNPDFK(G)
(K)GELVPEDDDKNLTEEQK(R)
(K)GELVPEDDDKNLTEEQKR(R)
(K)HIVNLVNK(D)
(K)HIVNLVNKDEVISVRP(-)
(K)IDELLKGELVPEDDDKNLTEEQK(R)
(K)KAIQEQEALWK(N)
(K)LVAKQQALNYFTK(A)
(K)NVHLLR(A)
(K)QQALNYFTK(A)
(K)RLLSDTYR(S)
(K)YLASCYAFEENMIK(K)
(K)YLASCYAFEENMIKK(N)
(R)EAIEQYRK(E)
(R)ERPEFK(R)
(R)LALSNYQSGVNTILK(M)
(R)LALSNYQSGVNTILKMR(E)
(K)KEGVSLLPYSPIAGGVLSGK(Y)
(K)TKLEEGDVLLILTPK(G)

(R)ASYPFEFENR(E)
(K)NVSGNHLDIGVGTGYLLQK(T)
(R)ANILEPIHITEK(F)
(R)LSNIAGQFK(A)
(K)GGFTDIEVDNLHASIAGNILK(I)
(K)INNLLGNETYKK(A)
(K)LNEAVNPLPYNK(Q)
(K)TYLYQSK(I)
(K)VLTLEEKEELR(Q)
(K)HALVGNLLASPVQGELSPEILAEWIK(Y)
(K)LFPEQYFWVHR(R)
(K)SVLHIGNLVAGTLFAPR(L)
(R)IAYENISHAFPEYTESQK(K)
(K)DIIGHAQTGTGK(T)
(K)FQNHPQIIDVTHQK(L)
(K)GIAFSFIVGK(Q)
(K)LMGDDYTALDIAAALFK(M)
(K)NPQIVIATPGR(M)
(K)TAAFAIPTIELLEVESK(H)
(R)GYFAEALHGDLNQK(Q)
(R)KGSIEILVATDVAGR(G)
(R)SIVDAGHIGK(Y)
(K)GEAILIDWLK(D)
(K)LIQVGANILQLNASGK(T)
(K)ANVFFNEIDAGFK(G)
(K)SLNVLIVQPDAPLSFR(D)
(R)FDSLMFLLANR(Y)
(R)FEPGLTHNLIR(Y)
(R)STNSGISANIDHLGR(F)
(R)YYNNNVLYDPNGDRR(D)
(K)FQVLSLETQK(A)
(K)IIIPALQK(K)
(K)ELQGNAAIGHNR(Y)
(K)LKELQGNAAIGHNR(Y)
(K)NGMNSYYPFPK(A)
(R)SGETDFLSALSSALKK(V)
(R)YAGMGLVAHIFTETK(L)
(K)GLIPINK(K)
(K)MNLFSEWK(V)
(K)VIHYGPSILAALENVPYESNIK(G)
(R)FIYDTLVFR(D)
(R)FIYDTLVFRDDK(S)
(R)LLDHEQYSLTYLVDYYHK(I)
(K)LIELTISGAK(S)
(K)NNFPGAPVIVGK(E)
(K)SGQGTIYGIAK(G)
(K)SLLKPGNLEEVAEAIMTTDTR(K)
(K)VFDEPIPFEGLQIYFGTLPVK(E)

(R)SGVLQAIVINSK(N)
(R)ILAISEVHNYLYK(S)
(R)LQIEDNGSGISDSK(T)
(K)GIFDVPFILAR(I)
(K)SIEIHHQVEVQTSVIR(I)
(K)EVDVVAFYPRPLENSPSSEK(A)
(K)GLGIAEGWPTR(I)
(K)GPISQVQGQAGMVLAK(S)
(K)GQYSFSPAAIK(I)
(K)GTENFGWLLEK(S)
(K)HPIEEALTR(K)
(K)IFITIDPK(G)
(K)IVQAVVNITTEEK(N)
(K)NIYFTQSNGER(F)
(K)NLEAYPLMESGGDAILDKNNNGK(L)
(K)VGILSNSLSFLNFK(V)
(R)FSPIFQNLPNEK(V)
(R)IRPDLEIYLDQLK(S)
(R)KIVQAVVNITTEEK(N)
(R)TKKPLAEVTGK(Q)
(R)NVWEYNSYR(A)
(K)FSFSLLNIGPLHYDVSK(S)
(K)GVPGEVVLWFK(N)
(K)IIELLFSADEK(K)
(K)ILDGLMTLK(D)
(K)YISSTVPLVEITK(E)
(R)FSLTPDKDGIPSVLFR(S)
(R)MYSPGSNVFSSSGK(Y)
(R)VAVPSLLSLEAPK(E)
(K)YGGVVIAALVIPR(L)
(K)AFVLWK(N)
(K)DFPGKPLK(S)
(K)DKPLPNWETPSYLGWNER(Q)
(K)ELQLLDGVSQILSDHFK(Y)
(K)IAEGLDQNIDTYEEDEKQLLK(F)
(K)KELQLLDGVSQILSDHFK(Y)
(K)SALKEIIGAYK(L)
(K)TNAYAEVWLGDK(Q)
(R)AASLLGWVPENALIPVAEAIVTAHR(D)
(R)FREALGSM(-)
(R)IDGYFVFAGGGFGMTHNKPETFAR(A)
(R)IKDFPGKPLK(S)
(R)NVTQAVNPLGK(K)
(R)SFPEVLNGIQK(V)
(K)ADIIQILAPDTIQADLYKK(D)
(K)GDALVFSHGFNIHYDFIKPPK(E)
(K)GPGHLVR(R)
(K)LITDLIYEGGLAR(M)

(K)NAGFEVYSVSEASQK(A)
(K)NASHPIESVGKK(L)
(K)TIAVIGYGSQGHAQAQNMK(D)
(R)ALAHAAGVGGGR(A)
(R)DKNASHPIESVGKK(L)
(R)MKEVLNDIQK(D)
(K)ISLSWK(K)
(K)LHGSSTPETYGK(A)
(-)MFLPHLR(Q)
(K)HLGLNYTK(D)
(K)TPGWFYSQSYTDVR(G)
(K)DFSSLLNWLR(Q)
(K)IQVYELPEIWNSK(M)
(R)LLSQELVEEFSITTSKAHSIWAKAR(K)
(R)LWENQVGR(S)
(R)TAYQEIYTFR(N)
(K)FTGNITLVYGAPYSDELVMMDYLK(G)
(K)GLADVGYTVR(L)
(K)HHLEGAHQLFVETY(-)
(K)ISGNTGTYEEFK(H)
(K)VISNVLLTPETGTGK(R)
(R)EPQINLFK(K)
(R)EPQINLFKK(S)
(R)LYSIASPSYSFGMK(E)
(R)LYSIASPSYSFGMKEDNIEFIIK(R)
(R)LYSIASPSYSFGMKEDNIEFIIKR(D)
(R)MYISHR(V)
(R)VREQAEAVKK(I)
(K)FLWDSIEFTHTLNLIR(D)
(K)FRDVIHQDLAIGALQNALK(S)
(K)IGHAYIFFGPR(G)
(K)MIGYHGIEFLTSFIK(S)
(K)MIGYHGIEFLTSFIKSLVDPDNHSK(S)
(K)TLEEPPAHIVFVLATTEFHK(I)
(R)DMLSFMEQAIVFTDSK(L)
(R)DVIHQDLAIGALQNALK(S)
(K)IITPGINSSLLEGITR(R)
(K)ISIQTHALQYGTTVFGGLR(G)
(K)TMVSSWR(R)
(K)VSGGYVNSALAK(S)
(R)HWLTPVY(-)
(R)SVLQIAK(D)
(K)MLAEVQAAAGAK(E)
(K)SFLETK(G)
(K)VNNSSGAYYLLAK(C)
(K)ADVIITTALIPGKK(A)
(K)APLLITK(K)
(K)KIGATVLVEK(G)

(K)IYDGTKPANVITSK(R)
(K)LLGLLSFK(G)
(K)VDLQNTLIYEEIASLKEDR(F)
(K)YEQALQASLK(L)
(R)SIDGLGHHVAFMK(Y)
(K)EIAVLLLNRPEK(R)
(R)HVITEK(Y)
(R)FGIFYLDEFKNPEFQEK(V)
(R)VIPSGSFLDK(T)
(K)NGLNMNQFR(S)
(R)LVSGAANGEKSISEQLK(E)
(K)TAPGAYYDEPALDGSRPGIFYANLR(D)
(R)EFHSVVLNNGSLPLNILEK(L)
(K)ELIVLHK(N)
(K)EGTIRLYLGYSK(V)
(K)ITDPVKEIVYGSVDGSFQSPK(I)
(K)NPSQLSQELK(R)
(K)TISLEYEKER(L)
(K)EAASHFQTAIQNDPK(N)
(K)GISLYHSGNK(K)
(K)LNSFNVEASDIEGILPGKK(D)
(R)VASALENGNQAMR(K)
(K)GAFNEEHLEIISTLANQAAQIFK(N)
(R)FESQGIYFLK(S)
(K)IKIDGIEYEVDEK(K)
(K)VTEGLSIFTK(N)
(K)NILPNQEKELEEK(F)
(K)KSFIVTDSYIVEFSSLGGK(I)
(K)TKESNSADPVVQK(E)
(R)IFADLR(S)
(R)LVGPSYK(G)
(K)IVTNDFNLNK(V)
(K)IVTNDFNLNKVAELQGVK(V)
(K)YIEDALHSVEGVAGQVNSKVEEVK(S)
(K)SENIESVQLSVSTHK(D)
(K)YVLSPVSGIQISTLKPGDK(I)
(K)KLVLADEPTGNLDR(E)
(R)HDNFPSQLSGGESAR(V)
(K)VLNTLSK(S)
(K)LGAFTIGTVGTSSK(I)
(K)KATQEIVTALK(E)
(K)KATQEIVTALKET(-)
(R)GLKPENTWPAFEEAIR(Y)
(K)KKEELILGDWVK(I)
(R)AIELKPNFR(A)
(R)INLALAYFETGNFLR(A)
(R)IFWDAPKETGEIIVAR(S)
(K)WANSLVPNNANVLNNLALVYEK(A)

(-)MLEFTK(R)
(K)SINETGIK(W)
(K)GQTIQVSSITTNVK(Y)
(K)NLGASLPGDIR(V)
(K)LFSIYLDNPNEVPKEK(L)
(R)ASIEIYEPLTEHK(T)
(K)LLQDLFFQGAK(E)
(R)GATAHFDLVAGESAK(I)
(K)SIALTVLGVIHDESGRYPQAIER(L)
(R)AFQSSIDLDPSLSEAYENKR(A)
(R)LAALSHSYLGNIAYNK(Q)
(R)VIELNPK(N)
(K)EAFEYADKNR(R)
(K)IISSENGDIVYASLASLLNITTKNEK(L)
(K)NKIISSENGDIVYASLASLLNITTK(N)
(K)SLSSGSDSEKYESAVALGKNK(E)
(R)TWYGPGNR(K)
(K)YLNALGSLPDPLR(E)
(R)LRPDQR(N)
(K)GFPPGPISNPGFPALEAAFYPK(E)
(K)ILQVITEGK(V)
(K)DRENTFSEILTAFYR(F)
(K)IQNKPLDLVLQQDYQK(L)
(K)LDKVPDNAHLIIDGSR(S)
(K)SPLSVSGPAAGLTVIVFDSIK(T)
(K)IGNLIGAEAILYIGYQKPYTECSTENK(I)
(K)VETGEVKK(A)
(K)YEYPLGYPQYFVK(K)
(R)KTVLEEPFTLFPESGALYLK(E)
(K)VSGAGATNSPLK(L)
(K)GFAVVAEEIGK(L)
(K)LITAGLLK(A)
(R)SPLIFTSPPR(E)
(K)EAILSILFK(A)
(K)SHYATDPYYYEIK(G)
(R)FPLLFSAPPR(N)
(K)VNDEVEIIGIRPTTKTVVTGIEMFR(K)
(R)GYAVNEALDILKFTNK(R)
(K)KPETVAQCIAESIALQIEQR(Q)
(K)VVIER(F)
(R)IGITR(G)
(R)ILFEMSGIDEETAKK(A)
(R)IFGPVAR(E)
(K)SAEIRPYK(N)
(K)VFLK(Y)
(R)VSTPGR(R)
(R)ALLMNCVK(G)
(K)AGIQDVLTKSWGSSNPVNIVK(A)

(K)LFGKD(-)
(K)EFYPVNLR(D)
(K)NQTAMGIEAK(R)
(K)FNGVSYVTQK(G)
(K)LFPEVTVHLPSGMGEFAR(D)
(K)LLENLLIK(F)
(K)SVLYFFEK(K)
(K)TGKTFTAELIGSDPVIDLALLK(I)
(R)AGQYVSSLNLIR(K)
(K)EIGIWIR(K)
(R)LVSFTK(K)
(R)VSGYIHEAVEK(G)
(K)KGDEVNLSLSSGK(V)
(R)AISNEMTR(L)
(R)FFIFEKPIK(K)
(K)VSMEQEEEEELTPLEDLPGLSAR(I)
(K)KGDDPSTPSPAASPK(K)
(K)STSDRDFSGAENTK(F)
(-)MIATEQK(K)
(K)VLELK(K)
(R)SYIR(K)
(R)VLEHTGISNLMPYLEK(I)
(R)VPESNQYEKPSVLYGLGEK(N)
(K)IELAK(S)
(K)SKFPNLTIEYLFDR(S)
(R)ILNDLLQSAGQLADHYENNNKK(I)
(K)GVVPENGK(I)
(K)KATEQALNYSHIEEIQLDK(I)
(K)ILSYTIPEISSLR(R)
(K)VGDGSAVEMIQNGKPK(I)
(R)GLVQTGETR(S)
(R)VGQILR(E)
(R)LSEIESYFELSPSRK(L)
(R)NLAIVWGDK(V)
(R)TVVEFDQNMDRK(K)
(R)ALAIEPR(F)
(R)IIAGLEDADKGQVIFEGR(E)
(K)IHSSASSLMNFSLQIEEAVQEQR(R)
(K)FQSGLNEELLR(Q)
(K)TIQPEPENGWK(R)
(R)IPLFLR(K)
(R)IWENVLSNR(G)
(R)QISSTSEAR(K)
(K)KHDDLVSGITVNYSGGADR(G)
(K)MLTNVAMAGTAAMGLRDPEK(G)
(K)TSVPGIYAIGDAIDGPMLAHK(A)
(K)LSLAFNPSHLECVDPVVTGSVR(A)
(R)AVNGTIHK(N)

(K)TTNELNDLKER(E)
(K)VVKALELIADLR(A)
(R)AENGRLESENETLR(A)
(K)IHNDQITTLEGNEIINLR(G)
(R)GVGMNVVNNLIQEFK(G)
(R)KIGFMVDELVGK(H)
(R)LEAEEVKELEEEIR(K)
(K)EGLEGLIHTSELAWSK(K)
(K)VDPYTDFQGSKIELALTK(F)
(K)GQGSPEVNSSLNENSGTVKK(M)
(K)HIGYEGFVFLGK(D)
(K)STLMPHWGFDTVLGEK(E)
(K)KIDPGPPREEDLFELSLDEIR(E)
(K)IPAESLVLGGGAPR(Y)
(K)TWKPDEIPDVTK(G)
(R)NLIYNSEEVAYFHISPSK(V)
(K)LYYVYPFQGSYVK(A)
(K)FSYMEDILLIK(K)
(K)LIDDLLEYSRLGR(R)
(R)SGNTAIMDR(I)
(R)INVVDTPGHADFGGEVER(V)
(K)IVEIFQQSGILEPIDGKK(N)
(R)LSFIASENREEWR(I)
(K)GHLVFIYPK(V)
(K)YYTYYPNDPAIGKPDR(T)
(R)HSLPLMPADPISTQFNLK(R)
(R)TIDYIFYSSNLK(Q)
(K)IALAGIDMIQGLK(D)
(K)FYGESGLSGK(T)
(K)TIIANETK(E)
(R)LEFTVSLK(E)
(K)DLYQATHEQYLQNSR(I)
(R)GMFVEATYEK(V)
(R)LRGDLIYDTNPNTLYYGIGER(S)
(K)YKDSPLKDPFGESEEK(S)
(R)DKYEEKDEIFPGK(T)
(K)VSGQSVGIIVDK(V)
(R)YITGIGK(K)
(K)KFPEVVGVVSR(M)
(K)LVEGPAAIYR(E)
(R)LAVHNLTLSQIYDK(L)
(R)VDIYSDEENIYLLADLPGVEEK(D)
(K)MTAFAGESGAGK(S)
(R)IILGFGR(Q)
(K)IASADFTNHELIR(L)
(K)YLYVTESSK(R)
(R)GILNVQVMGR(G)
(K)AMEDVGGNLNGKEYEILVK(T)

(K)TGGGEKLFGAVTPIDVASILK(K)
(R)SFLFFPFTLETK(D)
(K)NPALSVDLLK(L)
(K)HTYTIYSAK(Y)
(K)EIFLTNEEGIVLAHSNPEYLVENLKK(R)
(R)VAAIHMIYDR(G)
(K)SESIGSFGLASQSQR(L)
(K)TLLSFLGFGR(S)
(R)SISFFDDSNRAEEFLSSIK(I)
(R)FQPVYVK(E)
(K)EGITEEEKNSAK(F)
(K)FFQNELDKLK(T)
(K)GTFILK(Q)
(K)IVEEIGIQK(I)
(K)TLFQEIQKLK(S)
(K)YVSKEGITEEEK(N)
(R)EISEFITKNTDEIQR(K)
(R)IMNFVEPQVAFNDYRK(K)
(K)IPNGSIDK(K)
(K)KALNDAEETTKK(L)
(R)IWVPK(N)
(R)TVDSFHR(Q)
(R)TVIPILQQLIAQIEK(I)
(K)VVGADFEGSLAENKDLLAK(I)
(R)EKEFYGDAYIIR(S)
(R)LVLSIAAVEATALVQR(I)
(K)VILQFIR(S)
(R)LHENFNDPEIR(A)
(K)IAEVFEELTGSKISK(N)
(R)KHLLEYDDVMNR(Q)
(R)VEGHNFDIR(K)
(K)NEQVNMILSQYGR(N)
(K)HDVTNFDQVETVFQK(A)
(K)MNAFVIK(H)
(R)WGLVGLIIR(C)
(K)NNFAVGPIGDHVLEFTFMTPDGK(V)
(R)FVNFAK(F)
(K)YYQTVMFAEK(G)
(K)VDVFGTIGDKFDLVK(K)
(R)NYSIFMLGGK(E)
(K)IDTQPMLKLTVR(C)
(R)LEVVSPK(N)
(R)IGGEILTLANSR(S)
(K)EGILSALFR(F)
(K)NAIENVSDGVTEQFMHWIETK(R)
(K)SIVIVAGGTGGHISPGVALAEVLTELK(E)
(R)FYVNEAK(A)
(K)ENVDALKDSLR(Y)

(K)AQISLSEGDRDAK(I)
(R)SVGEREEAINISEGEK(M)
(R)FEDEINGKSLGLDQVK(A)
(R)NQPYFFYEEK(H)
(R)LAIQIGER(V)
(R)LATTGLSAQLR(A)
(K)TASSSSSFLK(T)
(K)TGLVLDAYFSGTK(I)
(K)ELPPLRDNTILAGNNPSIGPENAK(V)
(K)SFDQVKAEIENHLISQK(K)
(R)VLSEFASK(K)
(K)IVILGIPPVR(N)
(K)IAGVPEPIVK(R)
(K)WETLPPEK(Q)
(K)FLSGISLFQK(L)
(K)FPIIHLYFQQVGLKPEFDK(L)
(K)TEIISYFR(S)
(R)AITLEGR(E)
(K)ILFLENEK(V)
(K)TSLYHLIR(D)
(R)DVASVYSDRPMYWIR(E)
(R)TILFLPPWHIAER(L)
(K)AETFQSGPYK(S)
(K)LLSQVPNLK(K)
(R)KFITGFLNEEDFK(E)
(R)LGQPATTFSGGEAQR(I)
(K)VLENQIAVLK(T)
(R)LMNTVFASAVETDTVRK(A)
(K)TEGTMASIYIADNGPGIPIHLR(D)
(K)YSNANLSEVIR(Y)
(R)HGGQVFAEGEVNK(G)
(R)TFLETIER(N)
(-)MENLQSMPEVQKIISLGK(A)
(K)VLQEAVSK(I)
(K)VYDLIR(V)
(K)DILSVFVGIRPLISPEGNQDTK(N)
(K)FVLPAYR(F)
(R)LLENAPHLVKPLK(F)
(R)LQNYYGTEVFEVLGK(K)
(K)ITISEVSEVSSIPIR(A)
(K)LAEFTSSNAK(S)
(K)MLANQADAIQIVK(Q)
(K)KPAEQWFK(M)
(K)SSFTQTQIR(D)
(R)ILHSAGR(S)
(R)YNMPFLR(N)
(K)AIIQPGGSVR(D)
(K)KIQQTVASSEIVLEK(N)

(K)KLESEVAAR(Q)
(K)SKVDIQFADGSAIR(I)
(K)SLFLSGEGLVAVFSGSGK(L)
(K)LIENPK(I)
(K)LQEWAKENGINESNNEK(L)
(R)IEWIIADYGVILTGAANVPR(G)
(K)ESNQVFYYLK(D)
(R)IQIYGPMETGMHIQIHR(G)
(R)YQGEAFTGALR(I)
(K)FYLNYVLK(V)
(R)LIVADVGQDLWEEVSIVER(G)
(R)ELHIPLTTVK(A)
(R)VTILNILSDGK(V)
(K)KAAINDPWGTTYK(L)
(K)NADFNILNEDEYPSDFR(R)
(K)ITLELFQEIK(N)
(K)NILLDESLK(G)
(-)MNQLLR(E)
(K)IVLTVNTGLDR(V)
(K)FNSESMGK(D)
(K)GQLLALDFEK(L)
(K)IIEEFK(K)
(K)ISKEEIENYVTR(V)
(R)EKADAILVVDEAKK(I)
(R)LLTDAVILPGSTLKEEISR(D)
(R)ETLIVTSKVK(A)
(K)GLLEEFKNEYSIQK(D)
(K)YILDVK(K)
(K)ATGFSASETGIR(E)
(K)QILNSAPMQYR(I)
(R)KNASIIYDTLLNSDLK(K)
(K)GLVLSEIR(F)
(K)GFILFPR(T)
(K)IKISEEEAMEECQK(L)
(R)DMMITTIAAEKDGFTK(R)
(R)IHQSFFNGK(E)
(R)IHQSFFNGKEVIVEPLIASLQQETAR(D)
(K)SPSSFQSFPIDAIEIDHLEDTQK(L)
(K)TSDRDAENAVNGALMMR(E)
(K)VSLLGSNGENLISSK(T)
(R)SLSISLNSPTER(K)
(K)IPVLVK(Q)
(K)NVLTLGEISR(K)
(K)YHAFEEIAGLVPLEQNLKGDK(Y)
(K)YVQTHPILPSVK(K)
(R)GKYEQIVPALIAK(G)
(R)SYVVTSR(G)
(K)IETIPQLSLK(K)

(K)IISDFEEDLEKER(L)
(R)SAQFASEDEELRK(Q)
(K)IFDFAK(K)
(K)AATPEEKSMPHWFDTWIR(V)
(K)QAIAAEESLKK(A)
(K)YNSLTR(I)
(R)IKIPNPPKSFDDLK(N)
(K)GKQEPLQLYEVVWR(-)
(K)SMPDSVLDVLQKEER(S)
(K)VTLGPK(G)
(K)NLGVEANLKPDQTR(N)
(R)SGLNLSVTDK(N)
(K)LAEPSDHHTK(G)
(K)YTYHDSCYIGR(Y)
(R)AILYR(L)
(R)KAAGTENFLENPNWIFR(I)
(K)IPVAIVLK(S)
(R)AMGSQVFETTK(A)
(K)ASSLTVLGGTTSTTHYYSVYR(L)
(K)IGKPVYTSGGGGVFPSGIPVGIITEEGPR(N)
(K)TPNTNQTQTNETK(N)
(K)ALKEPTDEILDGIK(R)
(K)LTIGNAFPEK(K)
(R)GLEMYLSKETGVPVFR(A)
(R)NQYNLVVGER(T)
(R)ANELIISLAR(D)
(K)TEVFETGIKVIDLLAPYIK(G)
(K)LGSILSEK(F)
(K)NFFLSPTVSNEEKEK(V)
(K)ITDTIYIR(A)
(K)FQGQPDVIHPGIISTILDEIMVK(I)
(K)EFGYSYLK(L)
(R)SLISAMSAVQEK(N)
(K)FSIGAQYLVLHGFAR(L)
(R)LKPLSDFLSTIPGIPK(M)
(K)NRPLYVIENVYSTNFQK(Q)
(K)GNPDSIPLALR(T)
(K)KGDIDIYPTYTASFWVK(E)
(R)IYSNQFPK(S)
(R)IAQETLSLYAPIAGR(L)
(R)TLSFQPPEK(Q)
(K)IINVSGSVR(K)
(K)IKEGYSEIDDIR(K)
(K)SFPSSVDDARK(E)
(R)KPTTHSSEDSEAGESNNKK(K)
(R)YVVR(K)
(K)LAIEGK(I)
(K)TETHITIPDPHEVCSPNTAK(K)

(R)IFHNPLAQVAIK(R)
(R)LGVNGEAILR(C)
(K)ASLTLAGVTDLK(A)
(K)SFLESVVK(R)
(K)VISVEVIEK(N)
(K)DYVHFYSPDDHEHYPNGYEFK(D)
(K)LAQVTNHK(N)
(K)VPLAELAPELK(K)
(K)IVLYVR(K)
(K)FALWIPSSSKGYKEDEVLNFIK(G)
(K)EALDIQPLVTGGKSVR(D)
(K)HYEISLTSAEEVVSKGQAASYGK(A)
(K)VGVLFVNDVNLVYQAGEEWK(N)
(K)VHDMPDHVQFNHSR(H)
(K)EYGTHIIISEYTYEK(V)
(R)FTLLR(A)
(R)TWLKFPQPPIEPVAEK(A)
(K)INNTIGQVLIAK(A)
(K)ILGEYQNR(K)
(R)EGKPMEWNVTPR(I)
(R)KVNIVVDR(K)
(K)ISIPPISLK(D)
(K)QEWFLK(I)
(R)IPAIIDK(D)
(R)LYSVLDR(R)
(R)HIVSGNTENK(I)
(R)LSPPEKYK(K)
(K)ILEHEASSNK(A)
(K)QAIETFAK(T)
(K)YALSILEENKEKAITYLK(Q)
(K)YVFSILEEEGKFDLLNSFIK(S)
(K)YVNHSLLEDFLKMSEIGNNR(K)
(K)YYENKDEITELFK(N)
(R)LASGIAAR(L)
(K)SVAEQLFYDALEIIQK(K)
(R)GTLDTLGVDKR(R)
(K)AEADDLKK(K)
(K)AVELAK(S)
(R)KYGVPVTGISSELDQK(K)
(R)FSVIPGIR(Y)
(K)SFESPNSK(L)
(K)ILNFENR(E)
(K)QALRDQFLFLIETEECK(S)
(R)AFQEFMAK(K)
(R)LLESSLR(S)
(K)INKPVSSLETSGKDWLFR(L)
(K)LSYSITSGFENR(S)
(K)TQHIVSGFFGK(K)

(K)FLGDENLKLLAK(Y)
(K)GGILLTGMLGK(T)
(K)GMYFFPVSR(Y)
(K)EFGVTFK(N)
(K)LLAAGNPVTFYGR(A)
(R)FAEIRPIR(S)
(R)SQTIPIVVSFQNGVR(N)
(K)LSDNQFANNGVLP(-)
(R)VQPVMIPIK(H)
(K)VTNSNSFEISR(K)
(K)INVLNR(I)
(K)NADKDIFIPIVSWLDK(R)
(K)TLFIISK(A)
(K)QGGSTITQQLAK(L)
(K)GLSFNNVTYMYPGAK(N)
(K)NPAIQEINLEIAK(G)
(R)LVQEALESLYK(N)
(K)VTKAVITVPAYFNDEQR(Q)
(R)IPAVQELVKQVFGK(E)
(K)GTIISNPCR(S)
(R)KISLAQAILGAEIEVPTIDGK(K)
(K)IVKDPLLIESR(R)
(K)ALTWLEK(V)
(K)FILGK(S)
(K)GENILLIHGFPTASFDWEK(I)
(R)VVYYWHR(L)
(K)TNLIHSK(N)
(K)IETTVHDFMEDYTKPAIK(A)
(K)NEGVIVMAATNR(A)
(K)VTIIPR(G)
(R)DLFDQGKK(N)
(R)HVDFYK(V)
(K)TNIQNAIQNEK(S)
(K)IGQLGEVKDTVEDETSR(A)
(K)TKGATTTLEEIAYSK(I)
(R)QNLPQLLAPIYYGR(I)
(R)SIVTNMLPQFDTGAMIAIHPLEAIDKK(Y)
(R)YLSLHGFIGNGHGGGGDYGYGR(R)
(K)IEGEIHSGHSTFESTGSESILQK(L)
(R)LCQYDGEIPLYEEDESPFDR(L)
(K)ANSSEMQTLSIRPR(L)
(K)TDLVISNAGGKPVR(C)
(K)AINHLDEIK(N)
(K)DSPEYKHIVNLVNKDEVISVRP(-)
(K)VINDIKEVTSVAGK(N)
(K)DTLELIRPYLPSGIVR(R)
(K)TLETLAQFEK(T)
(R)LMEGASNYSAAK(N)

| |
|---|
| (K)ASLVLGYFMHLK(Y) |
| (K)KPDLIVLPEAGVPFFSAHK(T) |
| (R)HLGWTDTETFNHEAVR(S) |
| (R)SYYETTR(T) |
| (R)YYTLAEK(E) |
| (K)EFLLAK(I) |
| (K)GEVLIR(M) |
| (K)VINIVR(K) |
| (K)LDSNTMLLALGDVSGHGLGTGYLVSAIR(G) |
| (K)TVSSSVGGDFLGTAAYYEKEAK(G) |
| (K)SGYEFVK(G) |
| (K)SNSIGSGNER(L) |
| (R)NVILGMVLK(T) |
| (K)YLSSFPGVNVDK(I) |
| (R)GNTFLVSTSTER(E) |
| (K)VIMIEEGGYYHTGTFDNHELNMAGK(V) |
| (R)NIPSAFGVEEFR(L) |
| (K)GEWYLLGK(F) |
| (K)LTEFMIIELEELQNK(W) |
| (K)LSPLLK(K) |
| (K)TNMQELIEK(A) |
| (K)YEIVHEELEDVPAK(L) |
| (R)ALGSADLNKDNVVTALEAFFK(S) |
| (K)AQTLLEVIAEISDHSK(G) |
| (K)SVAEAAVVGFPHDIK(G) |
| (R)TGNESGLTWK(D) |
| (R)EAFVMIK(K) |
| (R)IAQGLLEGIER(E) |
| (R)HLEPGTISPVDSK(Y) |
| 7755 |

**Appendix 3**: Conserved proteins present in three serovars of *Leptospira interrogans.*

| Accession Number | Protein identification | Protein Length (Amino Acids) | Canicola | | Pomona | | Copenhageni | |
|---|---|---|---|---|---|---|---|---|
| | | | Mean No. of peptides | Mean % Coverage | Mean No. of peptides | Mean % Coverage | Mean No. of peptides | Mean % Coverage |
| P61442 | Hypothetical protein LIC10524 | 646 | 26 | 23 | 20 | 34 | NA | NA |
| Q72M74 | ABC transporter, ATP-binding protein | 309 | 5 | 18 | 6 | 21 | NA | NA |
| Q72MM7 | Hypothetical protein LIC13166 | 306 | 18 | 32 | 11 | 37 | NA | NA |
| Q72MR9 | Hypothetical protein LIC13123 | 291 | 5 | 17 | 14 | 45 | NA | NA |
| Q72MY9 | Hypothetical protein LIC13050 | 366 | 3 | 8 | 11 | 34 | NA | NA |
| Q72N71 | LipL41 | 355 | 56 | 46 | 8 | 29 | NA | NA |
| Q72NF9 | Elongation factor Tu | 401 | 121 | 52 | 16 | 48 | NA | NA |
| Q72NN7 | Acetyl-coa acetyltransferase | 441 | 27 | 32 | 9 | 21 | NA | NA |
| Q72NX2 | Transcription elongation factor nusa | 460 | 9 | 19 | 8 | 16 | NA | NA |
| Q72P55 | Hypothetical protein LIC12621 | 136 | 7 | 26 | 9 | 61 | NA | NA |
| Q72PA2 | Succinyl-coa synthetase beta subunit | 390 | 17 | 23 | 9 | 26 | NA | NA |
| Q72PL6 | Response regulator | 120 | 10 | 36 | 7 | 47 | NA | NA |
| Q72PR0 | Putative glutamine synthetase protein | 473 | 22 | 25 | 6 | 9 | NA | NA |
| Q72PY9 | Hypothetical protein LIC12326 | 123 | 4 | 19 | 6 | 31 | NA | NA |

| | | | | | | | | |
|---|---|---|---|---|---|---|---|---|
| Q72Q79 | Fructose-bisphosphate aldolase | 351 | 12 | 20 | 10 | 35 | NA | NA |
| Q72QN1 | Cysteine synthase | 309 | 47 | 40 | 12 | 34 | NA | NA |
| Q72QS2 | Hypothetical protein LIC12040 | 146 | 8 | 25 | 7 | 34 | NA | NA |
| Q72QV5 | Succinate dehydrogenase/fumarate reductase iron-sulfur subunit | 243 | 7 | 18 | 5 | 19 | NA | NA |
| Q72QV6 | Succinate dehydrogenase flavoprotein subunit | 637 | 13 | 21 | 8 | 13 | NA | NA |
| Q72QX3 | RNA-binding protein | 91 | 17 | 72 | 7 | 51 | NA | NA |
| Q72R01 | ATP-dependent Clp protease-like, proteolytic subunit | 197 | 7 | 28 | 6 | 31 | NA | NA |
| Q72R58 | Flagellin protein | 281 | 33 | 45 | 11 | 33 | NA | NA |
| Q72R63 | Putative lipoprotein | 412 | 16 | 30 | 5 | 18 | NA | NA |
| Q72RA0 | Hypothetical protein LIC11848 | 276 | 14 | 28 | 12 | 46 | NA | NA |
| Q72RC5 | Isopropylmalate isomerase small subunit | 206 | 8 | 33 | 3 | 11 | NA | NA |
| Q72RG3 | Hypothetical protein LIC11782 | 348 | 14 | 20 | 11 | 33 | NA | NA |
| Q72RT8 | Putative translaldolase | 214 | 14 | 30 | 12 | 56 | NA | NA |
| Q72RU5 | LipL45 | 390 | 18 | 32 | 11 | 37 | NA | NA |
| Q72S54 | Flagellin protein | 285 | 2 | 14 | 7 | 30 | NA | NA |
| Q72S55 | Flagellin protein | 282 | 13 | 22 | 11 | 46 | NA | NA |

| | | | | | | | | |
|---|---|---|---|---|---|---|---|---|
| Q72S74 | Putative polypeptide deformylase protein | 178 | 6 | 27 | 9 | 52 | NA | NA |
| Q72SC8 | LipL31 | 241 | 9 | 29 | 7 | 17 | NA | NA |
| Q72SG6 | ATP-dependent Clp protease, proteolytic subunit | 198 | 10 | 24 | 9 | 37 | NA | NA |
| Q72SG7 | FKBP-type peptidyl-prolyl cis-trans isomerase | 451 | 14 | 22 | 7 | 16 | NA | NA |
| Q72SM7 | LipL32 | 272 | 49 | 49 | 7 | 28 | NA | NA |
| Q72SR5 | Bacterioferritin | 178 | 6 | 20 | 7 | 47 | NA | NA |
| Q72SW4 | Hypothetical protein LIC11258 | 340 | 6 | 17 | 12 | 39 | NA | NA |
| Q72SX9 | F0F1 ATP synthase subunit beta | 467 | 21 | 43 | 20 | 49 | NA | NA |
| Q72SY1 | F0F1 ATP synthase subunit alpha | 503 | 25 | 30 | 22 | 47 | NA | NA |
| Q72T03 | Peroxiredoxin | 193 | 8 | 28 | 8 | 43 | NA | NA |
| Q72T25 | Hypothetical protein LIC11196 | 155 | 8 | 26 | 9 | 58 | NA | NA |
| Q72T27 | Putative citrate lyase | 330 | 15 | 26 | 11 | 16 | NA | NA |
| Q72TL5 | LipL71 | 555 | 13 | 15 | 11 | 17 | NA | NA |
| Q72U13 | Elongation factor Ts | 199 | 7 | 23 | 6 | 32 | NA | NA |
| Q72U26 | Glyoxalase | 141 | 4 | 19 | 5 | 29 | NA | NA |
| Q72U74 | Flagellar filament sheath protein | 315 | 9 | 19 | 8 | 34 | NA | NA |
| Q72UA9 | 50S ribosomal protein L7/L12 | 127 | 11 | 37 | 6 | 37 | NA | NA |

| | | | | | | | | |
|---|---|---|---|---|---|---|---|---|
| Q72UC6 | Cytosol aminopeptidase protein | 495 | 7 | 16 | 7 | 16 | NA | NA |
| Q72UI5 | Hypothetical protein LIC10672 | 229 | 4 | 11 | 8 | 27 | NA | NA |
| Q72UQ6 | Hypothetical protein LIC10601 | 150 | 13 | 35 | 9 | 51 | NA | NA |
| Q72UQ7 | Fatty acid synthase subunit beta | 138 | 11 | 45 | 7 | 60 | NA | NA |
| Q72V20 | Hypothetical protein LIC10483 | 330 | 13 | 29 | 11 | 31 | NA | NA |
| Q72V90 | Hypothetical protein LIC10411 | 157 | 43 | 58 | 9 | 50 | NA | NA |
| Q72VD7 | Electron transport flavoprotein beta subunit | 253 | 11 | 20 | 6 | 33 | NA | NA |
| Q72VI3 | Hypothetical protein LIC10314 | 557 | 23 | 20 | 4 | 11 | NA | NA |
| Q72VM5 | Elongation factor G | 706 | 46 | 25 | 10 | 24 | NA | NA |
| Q72VV5 | Peptidoglycan associated cytoplasmic membrane protein | 195 | 14 | 46 | 10 | 56 | NA | NA |
| Q72WD5 | DNA polymerase III beta subunit | 373 | 16 | 24 | 12 | 31 | NA | NA |
| Q75FE0 | Response regulator | 243 | 13 | 37 | 11 | 55 | NA | NA |
| Q75FJ7 | Hypothetical protein LIC20185 | 233 | 10 | 33 | 5 | 28 | NA | NA |
| Total Conserved Proteins | | | | | 60 | | | |

**Note: NA represents not applicable due to data being unavailable**

**Appendix 4:** Proteins conserved between three serovars of *Leptospira interrogans* grouped by their cellular content.

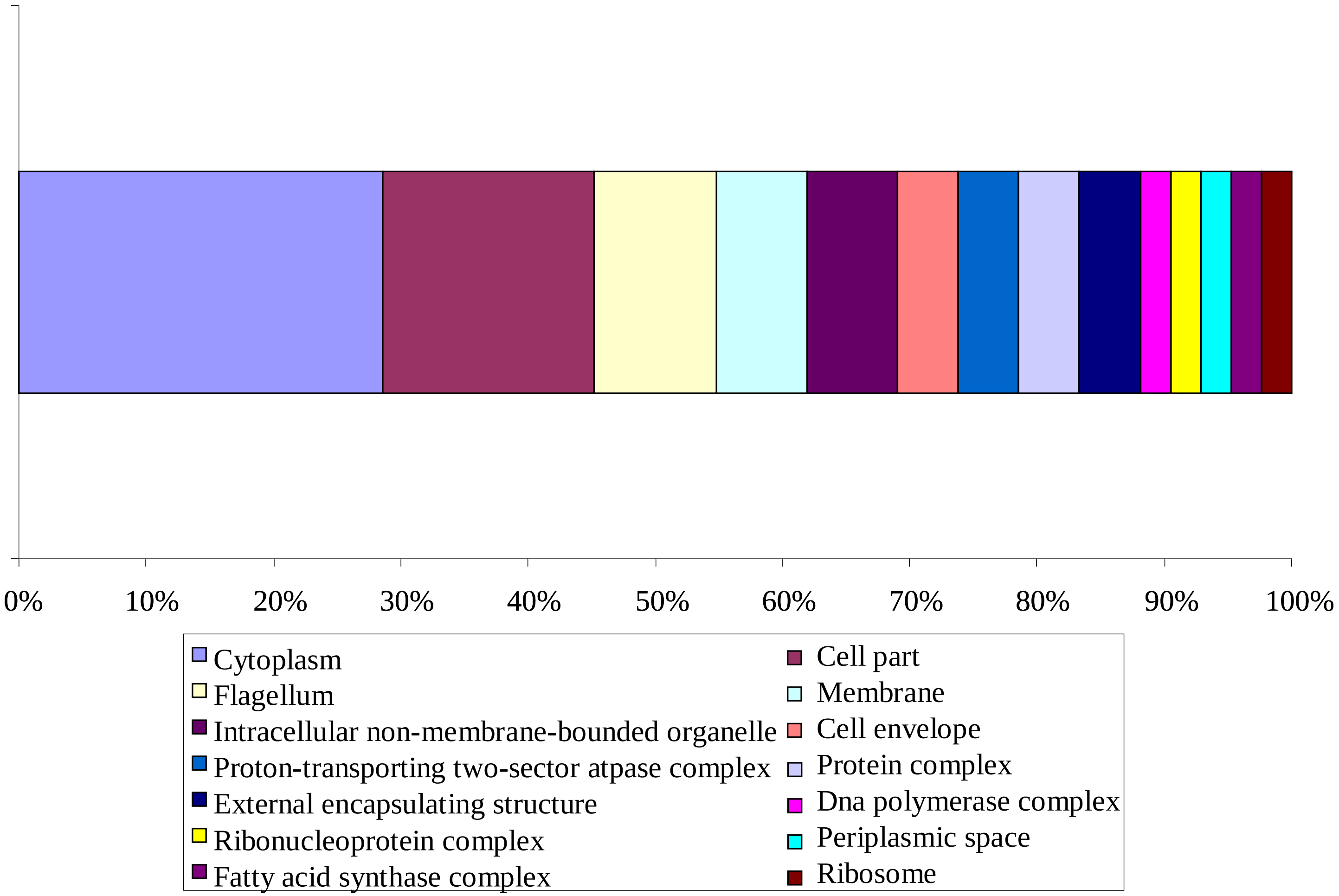


Note: Legend reads left to right.

**Appendix 5:** Proteins conserved between three serovars of *Leptospira interrogans* grouped by functional annotation.

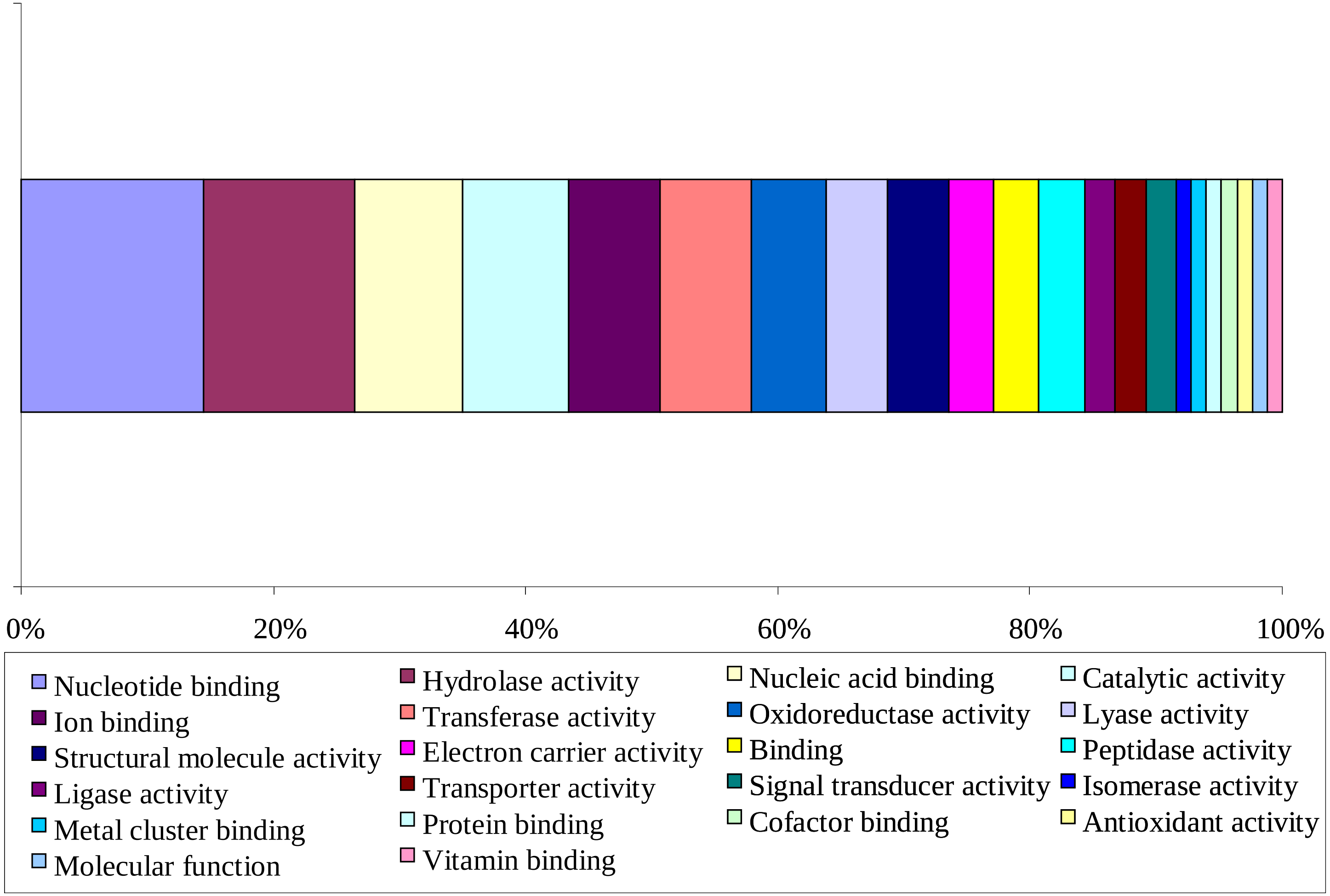


Note: Legend reads left to right.
